# Supplementary material for: Exploring the Epidemiology of Cancer after Solid Organ Transplantation (EpCOT): an observational cohort study
Source: BMJ Open. 2021 Apr 8;11(4):e043731. doi: 10.1136/bmjopen-2020-043731 (PMC8039244; doi:10.1136/bmjopen-2020-043731)
Supplement: Supplementary data [file bmjopen-2020-043731supp003.pdf]

HOSPITAL EPISODE STATISTICS DATA

Hospital Episode Statistics Admitted Patient Care

Period

1997/98
1998/99
1999/00
2000/01
2001/02
2002/03
2003/04
2004/05
2005/06
2006/07
2007/08
2008/09
2009/10
2010/11
2011/12
2012/13
2013/14
2014/15
2015/16
2016/17
2017/18
2018/19

Other fields

[ACTIVAGE Age at activity date,
[ADMIAGE Age on admission,
[ADMIDATE Date of admission,
[ADMIFLAG Admission episode flag,
[ADMIMETH Method of admission,
[ADMISORC Source of admission,
[ADMISTAT Psychiatric history on admission,
[CLASSPAT Patient classification,
[DIAG_3_NN Diagnosis - 3 characters,
[Diag_4_01 Primary Diagnosis - 4 characters,
[DIAG_4_NN Diagnosis - 4 characters,
[DIAG_NN All Diagnosis codes,
[DISDATE Date of discharge,
[DISDEST Destination on discharge,
[DISFLAG Discharge episode flag,
[DISMETH Method of discharge,
[DISREADYDATE Discharge ready date,
[DOMPROC Trust derived dominant procedure,
[ENCRYPTED_HESID Encrypted HESID,

[EPIEND Date episode ended,
[EPIORDER Episode order,
[EPISTART Date episode started,
[EPISTAT Episode status,
[EPITYPE Episode type,
[ETHNOS Ethnic category,
[FAE Finished Admission Episode,
[FCEFLAG Finished consultant episode flag,
[HRGNHSVN Version No. of Trust derived HRG,
[IMD04 IMD Index of Multiple Deprivation,
[IMD04_DECILE IMD Decile Group,
[IMD04C IMD Crime Domain,
[IMD04ED IMD Education Training and Skills Domain,
[IMD04EM IMD Employment Deprivation Domain,
[IMD04HD IMD Health and Disability Domain,
[IMD04HS IMD Barriers to Housing and Service Domain,
[IMD04I IMD Income Domain,
[IMD04IA IMD Income affecting Adults Domain,
[IMD04IC IMD Income affecting Children Domain,
[IMD04LE IMD Living Environment Domain,
[IMD04RK IMD Overall Rank,
[LSOA01 Lower Super Output Area (LSOA01),
[LSOA11 Lower Super Output Area (LSOA11),
[MAINSPEF Main specialty,
[OPDATE_NN Date of operation,
[OPERSTAT Operation status code,
[OPERTN_4_01 Primary Operative procedure codes 4 character,
[OPERTN_4_NN All secondary Operative procedure codes 4 character,
[PCON_ONS Westminster parliamentary constituency (ONS),
[PROCODE3 Provider code - 3 character,
[PROCODE5 Provider code - 5 character,
[PROCODET Provider code,
[PROVSPNOPS Pseudonymised hospital provider spell number,
[RESGOR Government office region of residence,
[SEX Sex of patient,
[SITETRET Site code of treatment,
[STARTAGE Age at start of episode,
[SUSHRG SUS generated HRG,
[SUSHRGVERS SUS generated HRG version number,
[TRETSPEF Treatment specialty

Hospital Episode Statistics Outpatients

Periods

2003/04
2004/05
2005/06
2006/07

2007/08
2008/09
2009/10
2010/11
2011/12
2012/13
2013/14
2014/15
2015/16
2016/17
2017/18
2018/19

Other fields
[APPTDATE Appointment date,
[ATTENDED Attended or did not attend,
[ATTENDID Attendance identifier,
[CHAPTER Primary diagnosis chapter,
[DIAG_3_01 Primary diagnosis - 3 character,
[DIAG_3_NN Secondary diagnoses - 3 character,
[DIAG_4_01 Primary diagnosis - 4 character,
[DIAG_4_CONCAT 4 character concatenated diagnosis,
[DIAG_4_NN Secondary diagnosess - 4 character,
[DIAG_COUNT Count of diagnoses,
[DIAG_NN Diagnosis,
[DNADATE Last DNA or patient cancelled date,
[ENCRYPTED_HESID Encrypted HESID,
[ETHNOS Ethnic category,
[HRGNHS Trust derived HRG value,
[HRGNHSVN Version No. of Trust derived HRG,
[IMD04 IMD Index of Multiple Deprivation,
[MAINSPEF Main specialty,
[MYDOB Date of Birth - month and year,
[NODIAGS Number of Diagnosis,
[OPERTN_4_NN All Operative procedure codes,
[OPERTN_NN Operative procedure,
[PROCODE3 Provider code (3 character),
[PROCODE5 Provider code (5 character),
[SEX Sex of patient,
[STAFFTYP Medical staff type seeing patient,
[SUSHRG SUS generated HRG,
[SUSHRGVERS SUS generated HRG version number,
[TRETSPEF Treatment specialty,
[WAITING Days waiting

Civil Registration (Deaths) - Secondary Care Cut
Periods

Future 31/12/2016
Sensitive fields
[cause_of_death] Original Underlying Cause of Death,
[cause_of_death_non_neonatal] Non-neonatal cause of death,
[death_record_used] Death Record Used,
[dod] Date of Death,
[subsequent_activity] Subsequent activity
