# Supplementary material for: Exploring the Epidemiology of Cancer after Solid Organ Transplantation (EpCOT): an observational cohort study
Source: BMJ Open. 2021 Apr 8;11(4):e043731. doi: 10.1136/bmjopen-2020-043731 (PMC8039244; doi:10.1136/bmjopen-2020-043731)
Supplement: Author's manuscript [file bmjopen-2020-043731.draft_revisions.pdf]

BMJ Open is committed to open peer review. As part of this commitment we make the peer review history of every article we publish publicly available.

When an article is published we post the peer reviewers' comments and the authors' responses online. We also post the versions of the paper that were used during peer review. These are the versions that the peer review comments apply to.

The versions of the paper that follow are the versions that were submitted during the peer review process. They are not the versions of record or the final published versions. They should not be cited or distributed as the published version of this manuscript.

BMJ Open is an open access journal and the full, final, typeset and author-corrected version of record of the manuscript is available on our site with no access controls, subscription charges or pay-per-view fees (http://bmjopen.bmj.com).

If you have any questions on BMJ Open's open peer review process please email info.bmjopen@bmj.com

BMJ Open

Protocol: observational cohort study exploring the Epidemiology of Cancer after solid Organ Transplantation (EpCOT)

Journal:	*BMJ Open*
Manuscript ID	bmjopen-2020-043731
Article Type:	Protocol
Date Submitted by the Author:	12-Aug-2020
Complete List of Authors:	Sharif, Adnan; University Hospitals Birmingham NHS Foundation Trust, Renal Medicine<br>Winter, David; University of Birmingham<br>Reulen, Raoul; University of Birmingham<br>Hawkins, Mike; University of Birmingham, School of Health and Population Sciences
Keywords:	TRANSPLANT SURGERY, TRANSPLANT MEDICINE, Transplant surgery < SURGERY, ONCOLOGY, Adult oncology < ONCOLOGY

SCHOLARONE™
Manuscripts

I, the Submitting Author has the right to grant and does grant on behalf of all authors of the Work (as defined in the below author licence), an exclusive licence and/or a non-exclusive licence for contributions from authors who are: i) UK Crown employees; ii) where BMJ has agreed a CC-BY licence shall apply, and/or iii) in accordance with the terms applicable for US Federal Government officers or employees acting as part of their official duties; on a worldwide, perpetual, irrevocable, royalty-free basis to BMJ Publishing Group Ltd ("BMJ") its licensees and where the relevant Journal is co-owned by BMJ to the co-owners of the Journal, to publish the Work in this journal and any other BMJ products and to exploit all rights, as set out in our licence.

The Submitting Author accepts and understands that any supply made under these terms is made by BMJ to the Submitting Author unless you are acting as an employee on behalf of your employer or a postgraduate student of an affiliated institution which is paying any applicable article publishing charge ("APC") for Open Access articles. Where the Submitting Author wishes to make the Work available on an Open Access basis (and intends to pay the relevant APC), the terms of reuse of such Open Access shall be governed by a Creative Commons licence – details of these licences and which Creative Commons licence will apply to this Work are set out in our licence referred to above.

Other than as permitted in any relevant BMJ Author's Self Archiving Policies, I confirm this Work has not been accepted for publication elsewhere, is not being considered for publication elsewhere and does not duplicate material already published. I confirm all authors consent to publication of this Work and authorise the granting of this licence.

For peer review only

Title. Protocol: observational cohort study exploring the Epidemiology of Cancer after solid Organ Transplantation (EpCOT)

Author. Adnan Sharif[1,2], David L Winter[3], Raoul C Reulens[3], Michael M Hawkins[3]

Address.

[1]Department of Nephrology and Transplantation, Queen Elizabeth Hospital, University Hospitals Birmingham, Edgbaston, Birmingham, United Kingdom

[2]Institute of Immunology and Immunotherapy, University of Birmingham, Edgbaston, Birmingham, UK

[3]Institute of Applied Health Research, University of Birmingham, Edgbaston, Birmingham, UK

Corresponding author contact information.

Dr. Adnan Sharif

Department of Nephrology and Transplantation

Queen Elizabeth Hospital, University Hospitals Birmingham

Mindelsohn Way, Edgbaston

Birmingham, B15 2GW, United Kingdom

Phone: (0121 371 5861), Fax: (0121 371 5858), Email: adnan.sharif@uhb.nhs.uk, Twitter: @AdnanSharif1979, Orchid ID: 0000-0002-7586-9136

Word count. 3,879

For peer review only

For peer review only - http://bmjopen.bmj.com/site/about/guidelines.xhtml

Abstract

Introduction. Solid organ transplant patients are counselled regarding increased risk for cancer (principally due to their need for lifelong immunosuppression) and it ranks as one of their biggest self-reported worries. Post-transplantation cancer is common, associated with increased healthcare costs and emerging as a leading cause of post-transplant mortality. However, epidemiology of cancer post-transplantation remains poorly understood, with limitations including translating data from different countries and national data being siloed across different registries and/or data warehouses.

Methods and analysis. Study methodology for EpCOT involves record linkage between the UK Transplant Registry (from NHS Blood and Transplant), Hospital Episode Statistics (for secondary care episodes from NHS Digital), national cancer registry (from cancer registration data hosted by Public Health England) and the national death registry (from NHS Digital). Deterministic record linkage will be conducted by NHS Digital, with a fully anonymised linked dataset available for analysis by the research team. The study cohort will consist of 85,410 solid organ transplant recipients, who received a solid organ transplant in England between January 1st 1985 and December 31st 2015, with up-to-date outcome data.

Ethics and dissemination. This study has been approved by the Confidentiality Advisory Group (reference: 16/CAG/0121), Research Ethics Committee (reference: 15/YH/0320) and institutional review board (reference: RRK5471). The results of this study will be presented at national and international conferences, and manuscripts with results will be submitted for publication in high-impact peer-reviewed journals. The information produced will also be

For peer review only

For peer review only - http://bmjopen.bmj.com/site/about/guidelines.xhtml

used to develop national evidence-based clinical guidelines to inform risk stratification to enable risk-based clinical follow up.

Registration. This observational study is registered with clinicaltrials.org (NCT02991105).

Strengths and limitations of this study

- Population-based cohort linked dataset of all solid organ transplant recipients in England to provide comprehensive coverage
- The largest national linked dataset of solid organ transplant recipients at risk for developing cancer available internationally
- Robust and collaborative data linkage to explore important clinical outcomes of interest post-transplantation
- Missing variables and confounding data may limit interpretation of the analysis

For peer review only

For peer review only - http://bmjopen.bmj.com/site/about/guidelines.xhtml

Introduction

Solid organ transplantation is established therapy for patients with end-stage organ dysfunction, providing lifesaving (e.g. heart, lung, liver) or life enhancing (e.g. kidney, pancreas) treatment. However, cancer is now emerging as one of the leading complications after solid organ transplantation. Transplant recipients have an increased risk of cancer compared with that expected from the general population [1-3] and comparable risk to immunodeficiency states such as HIV/AIDS [4], with similar preponderance for cancers with a viral component [5]. Multi-factorial aetiology underlies cancer risk after solid organ transplantation but immunosuppression, compulsory to prevent allograft rejection, contributes significantly to cancer risk [6, 7]. Standardised incidence ratio for development of all post-transplantation cancers (excluding non-melanoma skin cancers) remain constant and above 2-fold expected up to 15-years post-transplantation in the UK [3], meaning the absolute excess risk of cancer increases with time. Transplant recipients are achieving better long-term survival [8] and, among kidney transplant patients with more than 20-years graft function, *de novo* cancer develops in 37% of patients [9]. In summary, post-transplantation cancer is now the leading cause of death after kidney transplantation in the UK [10], death from cancer post-transplantation is in excess of that expected from the general population [11-14], it ranks as one of the biggest worries for post-transplant patients [15] and development of post-transplantation cancer is associated with increased healthcare cost [16].

Most publications on cancer epidemiology after solid organ transplantation focus on historical eras and practice has changed over the last decade. Immunosuppression is significantly different, especially since 2006 when dissemination of the SYMPHONY study

For peer review only - http://bmjopen.bmj.com/site/about/guidelines.xhtml

results led to switches from ciclosporin to tacrolimus-based immunosuppression [17]. This is important as tacrolimus may increase risk for post-transplantation cancer compared to ciclosporin [18], with an observed dose-effect relationship between tacrolimus and post-transplantation cancer [19]. Cancer risk appears decreased with sirolimus [20, 21], increased with azathioprine [22-24] but some studies suggest no difference in cancer-risk between immunosuppressant agents [25]. Induction immunosuppression is also more common and T-cell depletion induction has been linked with increased risk for post-transplantation cancer [26]. Evolving immunosuppression, changing transplant practice and selection of marginal donors/recipients justifies a more contemporary and time-dependent analysis of post-transplantation cancer.

Data related to the epidemiology of post-transplantation cancer predominantly comes from outside the UK (e.g. the US) but this may not be directly translatable. Immunosuppression use, the biggest modifiable risk factor for post-transplantation cancer [27], differs between the US and UK. For example, T-cell depletion therapy is the predominant induction agent in the US versus non T-cell depletion (e.g. basiliximab) in the UK [28]. Cumulative exposure to immunosuppression will be different, with financial coverage limitations in the US extending to immunosuppression provision [29] and this contributes to inferior post-transplant outcomes in the US compared to Europe [30, 31]. Kidney allograft recipients in England have more pre-transplant cancer history, more post-transplant cancer occurrence but superior overall graft survival and all-cause mortality when compared to contemporaneous recipients in New York State [32], suggesting post-transplantation cancer risk (and outcomes) are not comparable. Study cohorts from Australia and/or New Zealand are more aligned to UK

For peer review only - http://bmjopen.bmj.com/site/about/guidelines.xhtml

practice from an immunosuppression perspective. However, they have different demographics (e.g. less minority ethnic recipients) and burden of medical co-morbidities (e.g. cause of kidney failure) that limits direct translation of outcomes [33]. With only two epidemiology studies focused on post-transplantation cancer in the UK [3, 13], further studies in the UK are urgently warranted to inform patients and professionals.

Absence of relevant epidemiological data impacts upon patient counselling regarding increased risk for post-transplantation cancer, which is accepted by transplant candidates as a recognised complication from immunosuppression. However, individual risk stratification is not possible and this has implications in the contemporary era of consent [34]. Personalised counselling is difficult as we cannot predict individual post-transplant cancer risk, but gaining a clearer understanding of post-transplantation cancer risk, morbidity and mortality for specific transplant groups/cohorts will aid transplant discussions. KDIGO (Kidney Disease Improving Global Outcomes) guidelines for the care of (kidney) transplant patients report very poor level evidence when it comes to cancer, with either level 2C or non-graded evidence [35]. This translates into lack of consensus on cancer screening advice post-transplantation [36], lack of evidence-based guidance on how best to manage development of post-transplant cancer [37] and overall poor outcomes post-transplantation if transplant recipients develop cancer [11-13].

To understand the epidemiology of post-transplant cancer in the UK, it is imperative to bring together multiple sources of information relating to the same solid organ transplant recipient who may develop cancer contained across different registries or datasets. Big data linkage,

For peer review only - http://bmjopen.bmj.com/site/about/guidelines.xhtml

the process of bringing together and linking large volumes of healthcare records, can help identify factors and associations that would otherwise be difficult to robustly determine. The ability to link data can significantly increase the value that can be derived from individual datasets – which are often collected for specific regulatory purposes with considerable effort and expense – and maximise research benefit for patients. To that effect, Epidemiology of Cancer after solid Organ Transplantation (EpCOT) has the aim of exploring prespecified research questions of importance to the solid organ transplant community utilising big data record linkage of national healthcare datasets. The study protocol is registered with clinicaltrials.org (NCT02991105) and study results will be reported in accordance with STROBE guidelines [38].

Methods and Analysis

This epidemiological study will undertake record linkage between national datasets to link up the pathway from solid organ transplantation and subsequent risk for developing cancer. Deterministic record linkage will be undertaken by NHS Digital using identifiable patient data (specifically NHS number, date of birth, sex and postcode) between the following datasets:

(i) *UK Transplant Registry (UKTR)*

The UKTR is maintained by NHS Blood and Transplant and holds data for all solid organ transplants performed in the UK, with data submission mandatory for transplant centres. UKTR will provide data for the central study cohort of 85,410 patients who received a solid organ transplant between 1st January 1985 and 31st December 2015 in England, with donor-, recipient- and transplant-specific variables. An overview of the cohort is provided in Table 1,

For peer review only - http://bmjopen.bmj.com/site/about/guidelines.xhtml

with key demographics shown in Table 2.

(ii) *Civil registration deaths (NHS Digital)*

National death registration data is held by NHS Digital. For those patients that have died, the date and underlying cause of death from death certificate codes will be obtained for any death occurring in the United Kingdom (any deaths occurring outside of the United Kingdom will be missed but date of emigration [loss to follow up] will be provided). For date of death only a flag indicating that death has occurred and the months from transplantation to death (to calculate time interval) will be processed in line with legal approval.

(iii) *National cancer registry - National Cancer Registration and Analysis Service (NCRAS) from Public Health England (PHE)*

NCRAS collects information about every patient diagnosed with cancer in England. All variables of cancer-related interest will be linked to our cohort (between 1st January 1986 and 31st December 2017 unless stated otherwise) and include; cancer site (C00c-C097x and D00-D48), histology, treatment, chemotherapy (between 1st April 2012 and 31st March 2018) and radiotherapy (between 1st April 2009 and 31st March 2019).

(iv) *Hospital Episode Statistics (HES)*

HES is an administrative dataset collating information on all secondary care events in England, held by NHS Digital. From HES we can determine adverse health outcomes or procedures requiring hospitalisation (e.g. inpatient, outpatient and A&E attendance), for which the risk

For peer review only - http://bmjopen.bmj.com/site/about/guidelines.xhtml

may be increased as a result of having had a solid organ transplant. Period at risk of adverse health outcomes would be from 1997 until the most recent date for which HES is known to be complete.

Record linkage methodology

Data linkage methodology will involve UK Transplant Registry and NCRAS sending to NHS Digital only a list of identifying details for their respective cohorts. NHS Digital will link the UK Transplant Registry and NCRAS cohorts, determining which patients are common in both data sets. NHS Digital will then provide a list of pseudonymous IDs of patients common in both data sets to UK Transplant Registry and NCRAS respectively. This will facilitate extraction of clinical/health data from their data sets respectively, for provision to the University of Birmingham identifiable by the pseudonymous ID only. NHS Digital will facilitate linkage to HES and 'Civil Registration – Deaths' to extract records and supply anonymised records to University of Birmingham with the common pseudonymous ID. The University of Birmingham will then facilitate linkage between the NHS Digital, UKTR and NCRAS datasets using the pseudonymous ID common to all datasets. See Figure 1 for a summary of data flows for the EpCOT study.

Data security and governance

There will be no need, requirement or possibility to re-identify individuals after record linkage by the University of Birmingham (acting as Data Processor). Received data will be stored on a separate array of disks and accessed on a mapped drive as part of the University of Birmingham campus network. Access to this mapped drive will be limited to the named University researchers who can only access the University of Birmingham campus network

For peer review only - http://bmjopen.bmj.com/site/about/guidelines.xhtml

with a personalised username/password combination. The personal computers used to access the data will have additional virus checking/data security software installed (Malwarebytes). The separate array of disks will reside in a controlled environment with access only by accredited University IT staff. The data from this disk array will be backed up under a separate storage policy, which once deleted will make the data unavailable for restore.

The linkage, processing and analysis of the data from UKTR, NCRAS and NHS Digital will only be carried out the specified researchers within the University of Birmingham as the Data Processor who are appropriately trained in data protection and confidentiality. There will be no attempt made to link any data requested under this application to any held under other Data Sharing Agreements. This database of the pseudonymised data will be accessible only to named personnel within the project and will be kept in auditable documents. In addition, analysts fulfilling database administrator role (who are substantive members of the University of Birmingham) will also have access to the database but undertake no processing. At the end of the project, the data will be destroyed in line with strict policies and procedures on the destruction of data. No record level information will leave the Data Processor or aggregated data without small number suppression in line with the NHS Digital HES Analysis Guide.

Prespecified research questions

1. To compare observed and expected risks of specific causes of deaths, in particular cancer-related death, by linking the UKTR with the national death registry to obtain underlying causes of death and determine factors related to increased risk of specific causes of death post-transplantation. General population mortality rates will be used to calculate

For peer review only - http://bmjopen.bmj.com/site/about/guidelines.xhtml

expected number of deaths from specific causes and identify subgroups of post-transplant patients (e.g. age, sex, ethnicity, organ type, etc.) at excess risk compared with expected risk.

2. Investigate survival and causes of death after cancer in post-transplant patients versus individuals from the general population with a similar *de novo* cancer of the same age, sex, and calendar year of diagnosis.

3. Compare observed and expected risks of specific cancer types post-transplantation by linking the UKTR with the national cancer registry to obtain observed numbers of cancers and determine factors related to increased risk of specific types of cancer. General population cancer incidence rates will be used to calculate expected numbers of cancers of specific type and identify subgroups of post-transplant patients at excess risk of specific cancers compared with expected.

4. Estimate risk of morbidity requiring hospitalisation both generally and that associated with development of post-transplantation cancer by linking the UKTR with Hospital Episode Statistics (HES). Risk of hospital admissions and procedures (e.g. surgery) for specific morbidities will be investigated. We will calculate expected risks for specific conditions requiring hospitalisation, enabling identification of specific subgroups of post-transplant patients at excess risk compared with expected.

Detailed research plan

1: Compare observed and expected risks of specific causes of death

For peer review only - http://bmjopen.bmj.com/site/about/guidelines.xhtml
For peer review only

Each of the 85,410 patients in the UKTR enters risk at the date of transplantation and contributes person-years until the exit date. Standardised mortality ratios (SMRs) and absolute excess risks (AERs) will be calculated as O/E and [(O – E)/ py]*1000, where 'O' and 'E' are the observed and expected numbers of deaths respectively and 'py' is the total person-years at risk. To examine variation in SMRs or AERs across factors (e.g. age, sex, ethnicity, organ type, period of follow-up, calendar year of diagnosis), multivariable Poisson regression models will be utilised. Cumulative incidence of deaths from specific causes, in particular cancer, will be estimated (treating other causes of death as competing risks). All statistical analyses will use STATA 16 unless otherwise stated.

2: Investigate survival and causes of death after cancer in transplant patients

Comparators will be individuals from the general population with a similar *de novo* cancer of the same age, sex and calendar year of diagnosis. On one hand, heightened surveillance among solid organ transplant survivors should detect any cancers at an early stage and, therefore, reduce cancer-related mortality. On the other hand, previous treatment with immunosuppressants may limit treatment options for cancer and hence increase cancer-specific mortality. Survival comparisons will use Cox regression. We shall compare all-cause and cause-specific mortality of patients who have undergone a solid organ transplant and developed a cancer with that of individuals who developed a similar *de novo* cancer using Cox regression and/or Poisson regression analysis. An (anonymous) sample of patients from the general population who developed cancer will be obtained from NCRAS.

For peer review only - http://bmjopen.bmj.com/site/about/guidelines.xhtml

3: Compare observed and expected risks of specific cancer types

Data from NCRAS will identify diagnosis of post-transplantation cancers in terms of date and site/type. The initial analysis will involve constructing a table with rows corresponding to variables relating to characteristics (e.g. age, sex, organ etc.) of all transplant patients and the columns corresponding to the cancer site/types. Observed and expected numbers in each cell of this table will identify any evidence of an excess. Poisson regression as described above will be used to determine subgroups of patients at a substantially increased risk of specific cancers.

We will also compare cancer risk for transplant patients treated before/after 2006 (introduction of tacrolimus-based regimens) to evaluate whether more recently treated patients have increased/decreased post-transplantation cancer risk.

4: Estimate risk of morbidity requiring hospitalisation

Two types of analyses will be possible using UKTR-HES linked data:

A) Internal analyses: Risk of specific adverse outcomes post-transplantation will be compared over the period at risk using Poisson regression, to identify particular subgroups (e.g. in terms of attained age, sex, type of transplant, age at diagnosis, period of follow-up and calendar year of diagnosis) at greatest risk of the specific adverse health outcomes.

B) External analyses: Obtaining general population HES events classified by age, sex and calendar year and dividing by appropriate general population estimates will enable external comparisons. We will use Poisson regression to compare risk of adverse health

For peer review only - http://bmjopen.bmj.com/site/about/guidelines.xhtml

outcomes, over the period at risk, between transplant patients and the general population adjusting for attained age, sex and calendar year in the model. Patient sub-groups (defined in terms of attained age, sex, type of transplant, age at diagnosis, period of follow-up and calendar year of diagnosis) at substantial excess risk compared with expected will thus be identified.

Complementary to our analyses specified above, a data-driven methodology incorporating latest developments in Artificial Intelligence (e.g. machine learning) will be undertaken, enabling us to rank the importance of predictors for post-transplantation cancer. By developing and applying models to approximate complex and yet-unknown interactions between clinical features, better prediction of patient outcomes could inform certain streams of post-transplantation care.

For peer review only

Analytical considerations

Adequate power for study events

In previous work by Collett et al. [3], 37,617 solid organ transplant recipients in the UK were analysed, whilst in the current proposal we include more than double (N=85,410). Using a conservative approach, assuming number of expected cancers will be twice that from previous work by Collett and colleagues (although expected number will likely be much greater as the cohort has aged), then we will be able to detect SIRs of at least the following magnitudes with 80% power when using a 5% significance level (see Table 3).

For peer review only - http://bmjopen.bmj.com/site/about/guidelines.xhtml

Thus, apart from cancer sites of anus, lip and Kaposi sarcoma, we should be able to detect an SIR of 2.0 or less with at least 80% statistical power. Similar logic would apply to our analyses relating to hospitalisations and mortality.

Missing data

Missing data is a reality of both administrative and registry data. Examples of the degree of missing data from the UKTR has been indicated in Table 2, but the final amount after record linkage is undertaken will not be known and in some instances could be reduced (e.g. missing ethnicity data in UKTR may be available from HES or NCRAS which also contain this data field).

Our approach for dealing with missing data will be to only analyse the available data (i.e. ignoring the missing data) and conduct sensitivity analyses to explore whether missing data could have biased our results; or, where appropriate, to impute missing data and account for the fact that missing values were imputed with uncertainty (e.g. multiple imputation).

Quality assurance for submitted data and record linkage

Under our section 251 approval, record linkage will be facilitated by NHS Digital. The research team is therefore reliant on robust quality assurance mechanisms from both data senders and NHS Digital Data Linkage and Extract Services to ensure a robust and comprehensive record linkage process. We have worked very closely with all partners, carefully selecting fields from the available data dictionaries and checking their suitability and viability for record linkage. Cost recovery frameworks have been factored into our budget to ensure robust data

For peer review only - http://bmjopen.bmj.com/site/about/guidelines.xhtml

extraction and quality assurance processes are in place. NHS Digital charges also includes list cleaning, a validation process using demographic data to ensure accuracy and improve linkage outcomes. A record linkage report will be available to check record linkage success rate.

Patient and Public involvement (PPI)

Patient involvement has been critical in the design of our research aims, to ensure our research questions are both relevant and appropriate. We have developed our research aims after discussion with key stakeholders like patients to ensure we are seeking answers to questions that they wish to be answered and further study into post-transplantation cancer meets those needs. Our departmental PPI forum involving transplant candidates and patients, hosted as part of the Trust's initiative to ensure adequate patient and public representation in research drives, has confirmed qualitative study findings that post-transplantation cancer is a leading concern and an area where preventative strategies would be overwhelmingly welcomed. Patients were very receptive to our strategy of record linkage, both using anonymised national data resources and also using linked primary/secondary care data after informed consent if required for any future extension work.

For peer review only

Ethics and dissemination

Approvals for record linkage

The EpCOT study research plan has been reviewed and approved by our data contributing partners NHS Digital, the UK Transplant Registry and Public Health England. Their support, collaboration and advice has been critical for the development of data flows and we plan to

For peer review only - http://bmjopen.bmj.com/site/about/guidelines.xhtml

work in close partnership at various stages of the project. Our wide consortium of partners has demonstrated their strong support to ensure the success of this project over many years of development.

The EpCOT project has attained institutional (*RRK5471*) and ethical (*15/YH/0320*) approval. The project also has section 251 approval from the Confidentiality Advisory Group (*16/CAG/0121*), under Regulation 5 of the Health Service (Control of Patient Information) Regulations 2002, for the record linkage to proceed by NHS Digital.

Dissemination plan

The aim of this project is to produce targeted studies looking at the epidemiology of post-transplantation cancer, and this data will be disseminated through presentations at national/international congresses and submitted for publication in leading medical journals. The aim is also to develop Standards of Care guidelines, working with the Standards Committee of the British Transplantation Society to provide evidence-based clinical evidence for direct patient benefit. These will be made freely available to the community at their website (https://bts.org.uk/guidelines-standards/). It is also the aim to plan patient-focused dissemination in plain English through various channels of communication for patients and the general public.

Outputs will only contain aggregate data with small numbers suppressed in line with the NHS Digital HES Analysis Guide. Both NHS Blood and Transplant and Public Health England (who will provide the initial data cohorts on this project) have mechanisms for dissemination of

For peer review only - http://bmjopen.bmj.com/site/about/guidelines.xhtml

information to both professional and general population and we will use these to share the results from EpCOT widely. We also aim to do similar research presentation at Research Open Days at both University Hospitals Birmingham and the University of Birmingham. Summary research findings are actively disseminated via social media channels (e.g. @AdnanSharif1979; @UHBResearch; @ImmunologyUoB), while tagging our collaborative partners (@NHSBT_RD; @PHE_uk; @NHSDigital). Project partners will be encouraged to disseminate their involvement through all social media channels.

Discussion

Large epidemiological studies from the US (using data from the Transplant Cancer Match Study) have recently reported post-transplant cancer epidemiology outcomes for a cohort of 221,962 solid organ transplant recipients, of whom 15,012 developed cancer [39]. One of the main findings from Noone and colleagues is cancer-related mortality increases steadily with post-transplantation time duration, reaching 15.7% of deaths (810 per 100,000 person-years) after 10 or more years after transplantation. While a large cohort, one of the limitations of this dataset is the incomplete national coverage; a total of 17 cancer registries provided data regarding incident cancers (excluding nonmelanoma skin cancer) that covers approximately one-half of the US transplant population with varying years of coverage data. To the best of our knowledge, EpCOT is the largest population-cohort study exploring cancer epidemiology after solid organ transplantation using record linkage of national datasets with extended years of coverage.

For peer review only - http://bmjopen.bmj.com/site/about/guidelines.xhtml

In order to fulfil its regulatory mandate, NHS Blood and Transplant (NHSBT) collects data for all solid organ transplant recipients including long-term follow-up information. The collection of national data enables NHSBT to fulfil its statutory obligations with regard to the effective use of organs, equitable organ offering and performance monitoring of transplant centres in terms of patient and graft outcomes. Such data has tremendous analytical potential and is available for research purposes with appropriate approval. However, the exploration of certain outcomes like cancer is limited as this information is not routinely collected by NHSBT. To overcome this issue, record linkage between the transplant registry and national cancer data is essential. The methodology of EpCOT facilitates this, with the inclusion of all secondary care episodes and death registrations, to obtain a robust, comprehensive and informative population-cohort study of post-transplantation cancer. From an epidemiological perspective, this allows detailed analyses to assess the true impact of cancer for solid organ transplant recipients and provides coverage of the full patient journey from transplantation to cancer-related outcomes.

In summary, cancer is a leading cause of morbidity and mortality after solid organ transplantation. With incidence of cancer projected to increase, improving our understanding of post-transplant cancer epidemiology is essential to improve long-term patient outcomes. EpCOT will be one of the largest record linkages of solid organ transplant recipients, with contemporary and comprehensive national cancer-related outcomes, to provide answers to questions of importance to both transplant professionals and organ recipients respectively.

For peer review only - http://bmjopen.bmj.com/site/about/guidelines.xhtml

References:

1. Engels EA, Pfeiffer RM, Fraumeni JF, Jr., Kasiske BL, Israni AK, Snyder JJ, Wolfe RA, Goodrich NP, Bayakly AR, Clarke CA *et al*: **Spectrum of cancer risk among US solid organ transplant recipients**. *JAMA* 2011, **306**(17):1891-1901.
2. Kasiske BL, Snyder JJ, Gilbertson DT, Wang C: **Cancer after kidney transplantation in the United States**. *Am J Transplant* 2004, **4**(6):905-913.
3. Collett D, Mumford L, Banner NR, Neuberger J, Watson C: **Comparison of the incidence of malignancy in recipients of different types of organ: a UK Registry audit**. *Am J Transplant* 2010, **10**(8):1889-1896.
4. Grulich AE, van Leeuwen MT, Falster MO, Vajdic CM: **Incidence of cancers in people with HIV/AIDS compared with immunosuppressed transplant recipients: a meta-analysis**. *Lancet* 2007, **370**(9581):59-67.
5. Morath C, Mueller M, Goldschmidt H, Schwenger V, Opelz G, Zeier M: **Malignancy in renal transplantation**. *J Am Soc Nephrol* 2004, **15**(6):1582-1588.
6. Vajdic CM, van Leeuwen MT: **Cancer incidence and risk factors after solid organ transplantation**. *Int J Cancer* 2009, **125**(8):1747-1754.
7. Geissler EK: **Post-transplantation malignancies: here today, gone tomorrow?** *Nat Rev Clin Oncol* 2015, **12**(12):705-717.
8. **UK Transplant Activity Report 2019-2019. Accessed 5th September 2019 at; https://nhsbtdbe.blob.core.windows.net/umbraco-assets-corp/16469/organ-donation-and-transplantation-activity-report-2018-2019.pdf** Accessed 29th May 2018.
9. McCaughan JA, Courtney AE: **The clinical course of kidney transplant recipients after 20 years of graft function**. *Am J Transplant* 2015, **15**(3):734-740.
10. Steenkamp R, Rao A, Fraser S: **UK Renal Registry 18th Annual Report (December 2015) Chapter 5: Survival and Causes of Death in UK Adult Patients on Renal Replacement Therapy in 2014: National and Centre-specific Analyses**. *Nephron* 2016, **132 Suppl 1**:111-144.
11. Na R, Grulich AE, Meagher NS, McCaughan GW, Keogh AM, Vajdic CM: **De novo cancer-related death in Australian liver and cardiothoracic transplant recipients**. *Am J Transplant* 2013, **13**(5):1296-1304.
12. Acuna SA, Fernandes KA, Daly C, Hicks LK, Sutradhar R, Kim SJ, Baxter NN: **Cancer Mortality Among Recipients of Solid-Organ Transplantation in Ontario, Canada**. *JAMA Oncol* 2016, **2**(4):463-469.
13. Farrugia D, Mahboob S, Cheshire J, Begaj I, Khosla S, Ray D, Sharif A: **Malignancy-related mortality following kidney transplantation is common**. *Kidney Int* 2014, **85**(6):1395-1403.
14. Kiberd BA, Rose C, Gill JS: **Cancer mortality in kidney transplantation**. *Am J Transplant* 2009, **9**(8):1868-1875.
15. Howell M, Tong A, Wong G, Craig JC, Howard K: **Important outcomes for kidney transplant recipients: a nominal group and qualitative study**. *Am J Kidney Dis* 2012, **60**(2):186-196.
16. Dharnidharka VR, Naik AS, Axelrod D, Schnitzler MA, Xiao H, Brennan DC, Segev DL, Randall H, Chen J, Kasiske B *et al*: **Clinical and Economic Consequences of Early Cancer**

For peer review only - http://bmjopen.bmj.com/site/about/guidelines.xhtml

After Kidney Transplantation in Contemporary Practice. *Transplantation* 2017, **101**(4):858-866.

17. Ekberg H, Tedesco-Silva H, Demirbas A, Vitko S, Nashan B, Gurkan A, Margreiter R, Hugo C, Grinyo JM, Frei U *et al*: **Reduced exposure to calcineurin inhibitors in renal transplantation**. *N Engl J Med* 2007, **357**(25):2562-2575.
18. Wimmer CD, Angele MK, Schwarz B, Pratschke S, Rentsch M, Khandoga A, Guba M, Jauch KW, Bruns C, Graeb C: **Impact of cyclosporine versus tacrolimus on the incidence of de novo malignancy following liver transplantation: a single center experience with 609 patients**. *Transpl Int* 2013, **26**(10):999-1006.
19. Carenco C, Assenat E, Faure S, Duny Y, Danan G, Bismuth M, Herrero A, Jung B, Ursic-Bedoya J, Jaber S *et al*: **Tacrolimus and the risk of solid cancers after liver transplant: a dose effect relationship**. *Am J Transplant* 2015, **15**(3):678-686.
20. Yanik EL, Siddiqui K, Engels EA: **Sirolimus effects on cancer incidence after kidney transplantation: a meta-analysis**. *Cancer Med* 2015, **4**(9):1448-1459.
21. Sharif A: **Sirolimus after kidney transplantation**. *BMJ* 2014, **349**:g6808.
22. Jiyad Z, Olsen CM, Burke MT, Isbel NM, Green AC: **Azathioprine and Risk of Skin Cancer in Organ Transplant Recipients: Systematic Review and Meta-Analysis**. *Am J Transplant* 2016, **16**(12):3490-3503.
23. Na R, Laaksonen MA, Grulich AE, Meagher NS, McCaughan GW, Keogh AM, Vajdic CM: **Iatrogenic immunosuppression and risk of non-Hodgkin lymphoma in solid organ transplantation: A population-based cohort study in Australia**. *Br J Haematol* 2016, **174**(4):550-562.
24. Na R, Laaksonen MA, Grulich AE, Meagher NS, McCaughan GW, Keogh AM, Vajdic CM: **High azathioprine dose and lip cancer risk in liver, heart, and lung transplant recipients: A population-based cohort study**. *J Am Acad Dermatol* 2016, **74**(6):1144-1152 e1146.
25. Gallagher MP, Kelly PJ, Jardine M, Perkovic V, Cass A, Craig JC, Eris J, Webster AC: **Long-term cancer risk of immunosuppressive regimens after kidney transplantation**. *J Am Soc Nephrol* 2010, **21**(5):852-858.
26. Hall EC, Engels EA, Pfeiffer RM, Segev DL: **Association of antibody induction immunosuppression with cancer after kidney transplantation**. *Transplantation* 2015, **99**(5):1051-1057.
27. Gutierrez-Dalmau A, Campistol JM: **Immunosuppressive therapy and malignancy in organ transplant recipients: a systematic review**. *Drugs* 2007, **67**(8):1167-1198.
28. Hardinger KL, Brennan DC, Klein CL: **Selection of induction therapy in kidney transplantation**. *Transpl Int* 2013, **26**(7):662-672.
29. Gill JS, Tonelli M: **Penny wise, pound foolish? Coverage limits on immunosuppression after kidney transplantation**. *N Engl J Med* 2012, **366**(7):586-589.
30. Ojo AO, Morales JM, Gonzalez-Molina M, Steffick DE, Luan FL, Merion RM, Ojo T, Moreso F, Arias M, Campistol JM *et al*: **Comparison of the long-term outcomes of kidney transplantation: USA versus Spain**. *Nephrol Dial Transplant* 2013, **28**(1):213-220.
31. Gondos A, Dohler B, Brenner H, Opelz G: **Kidney graft survival in Europe and the United States: strikingly different long-term outcomes**. *Transplantation* 2013, **95**(2):267-274.
32. Jackson-Spence F, Gillott H, Tahir S, Nath J, Mytton J, Evison F, Sharif A: **Cancer-related outcomes in kidney allograft recipients in England versus New York State: a**

For peer review only

comparative population-cohort analysis between 2003 and 2013. *Cancer Med* 2017, **6**(3):563-571.

33. Au EH, Chapman JR, Craig JC, Lim WH, Teixeira-Pinto A, Ullah S, McDonald S, Wong G: **Overall and Site-Specific Cancer Mortality in Patients on Dialysis and after Kidney Transplant**. *J Am Soc Nephrol* 2019.
34. Lee A: **'Bolam' to 'Montgomery' is result of evolutionary change of medical practice towards 'patient-centred care'**. *Postgrad Med J* 2017, **93**(1095):46-50.
35. Kidney Disease: Improving Global Outcomes Transplant Work G: **KDIGO clinical practice guideline for the care of kidney transplant recipients**. *Am J Transplant* 2009, **9 Suppl 3**:S1-155.
36. Acuna SA, Huang JW, Scott AL, Micic S, Daly C, Brezden-Masley C, Kim SJ, Baxter NN: **Cancer Screening Recommendations for Solid Organ Transplant Recipients: A Systematic Review of Clinical Practice Guidelines**. *Am J Transplant* 2017, **17**(1):103-114.
37. Ajithkumar TV, Parkinson CA, Butler A, Hatcher HM: **Management of solid tumours in organ-transplant recipients**. *Lancet Oncol* 2007, **8**(10):921-932.
38. von Elm E, Altman DG, Egger M, Pocock SJ, Gotzsche PC, Vandenbroucke JP, Initiative S: **Strengthening the Reporting of Observational Studies in Epidemiology (STROBE) statement: guidelines for reporting observational studies**. *BMJ* 2007, **335**(7624):806-808.
39. Noone AM, Pfeiffer RM, Dorgan JF, Magder LS, Bromberg JS, Lynch CF, Morris CR, Pawlish KS, Engels EA: **Cancer-attributable mortality among solid organ transplant recipients in the United States: 1987 through 2014**. *Cancer* 2019, **125**(15):2647-2655.

For peer review only

For peer review only - http://bmjopen.bmj.com/site/about/guidelines.xhtml

Authors' Contributions: Adnan Sharif[1-3], David L Winter[2,3], Raoul C Reulens[2,3], Michael M Hawkins[2,3]

[1] Wrote first draft

[2] Reviewed manuscript

[3] Approved final version

Funding Statement: This work was supported by a Wellcome Trust Institutional Strategic Support Fund: Digital Health Pilot Grant and the Kildare Trust.

Competing Interests Statement: No competing interests.

For peer review only

For peer review only - http://bmjopen.bmj.com/site/about/guidelines.xhtml

Table 1. Snapshot of the UK Transplant Registry cohort

	01/01/1985-31/12/1994	01/01/1995-31/12/2005	01/01/2006-31/12/2015	Total
**Kidney**	14341	16172	23436	53949
**Pancreas**	76	451	1835	2362
**Heart**	3271	2533	1457	7261
**Lung**	1160	1593	1683	4436
**Liver**	3296	6846	6925	17067
**Pancreas islets**	0	0	120	120
**Abdominal/intestinal**	4	47	164	215
**Total**	22148	27642	35620	85410

For peer review only

For peer review only - http://bmjopen.bmj.com/site/about/guidelines.xhtml

<u>**Table 2. Demographics of UK Transplant Registry cohort**</u>

		Kidney (n=53949)	Pancreas (n=2362)	Heart (n=7261)	Lung (n=4436)	Liver (n=17067)	Pancreas islets (n=120)	Abdominal / Intestinal (n=215)	Missing or unreported data
First graft		46347	2230	7115	4305	15241	74	201	0
Part of multi-organ transplant		2199	1994	1136	1027	441	0	137	0
Donor	Living	12475	0	0	24[1]	380	0	0	1
	DBD[2]	35310	2039	7246	4199	15551	110	215	
	DCD[3]	6164	323	14	224	1136	10	0	
Female recipient		20510	1006	2008	2062	7517	87	93	48
Ethnicity of recipient	White	33556	2060	3963	3237	12073	117	181	19029
	Asian	4641	115	269	75	1476	1	15	
	Black	2369	69	77	26	438	2	5	
	Chinese	300	5	11	2	107	0	0	
	Mixed	122	5	15	1	23	0	1	
	Other	545	19	39	9	408	0	3	
Recipien	0-9	1329	37	611	86	2028	0	98	22

For peer review only - http://bmjopen.bmj.com/site/about/guidelines.xhtml

t age (in years)	10-19	2860	20	733	392	946	0	15
	20-29	6546	168	746	734	1176	0	21
	30-39	9719	781	813	607	1745	10	23
	40-49	12285	912	1556	864	3390	42	26
	50-59	11972	397	2265	1288	4812	48	20
	60-69	7733	47	531	464	2861	17	11
	70-79	1464	0	3	1	106	3	1
	80+	25	0	0	0	0	0	0

[1]Partial solitary lung, [2]Donation after brain death, [3]Donation after cardiac death

For peer review only

Table 3. Adequate power for EpCOT study events

Site of cancer development	Estimated expected number of cancers	Minimal SIR that can be detected
All cancers excluding non-melanoma	1650	1.1
Skin: non-melanoma	294	1.1
Lung and Bronchus	252	1.1
Breast	243	1.1
Colorectal	200	1.2
Prostate	197	1.2
Bladder	64	1.3
Non-Hodgkin's lymphoma	56	1.3
Skin – malignant	53	1.4
Stomach	49	1.4
Oesophagus	44	1.4
Kidney	39	1.5
Pancreas	38	1.5

For peer review only - http://bmjopen.bmj.com/site/about/guidelines.xhtml

Leukaemia	37	1.5
Ovary	35	1.5
Uterus	31	1.5
Multiple myeloma	21	1.7
Cervix	18	1.8
Oral cavity	16	1.8
Liver	16	1.8
Hodgkin's lymphoma	10	1.9
Thyroid	9	2.0
Anus	5	3.0
Lip	2	4.0
Kaposi sarcoma	1.4	5.5

For peer review only

Figure legend

Figure 1. Flow of study data for the EpCOT project between data providers, NHS Digital and the University of Birmingham as Data Processor.

For peer review only - http://bmjopen.bmj.com/site/about/guidelines.xhtml

Figure 1. Flow diagram of data linkage process

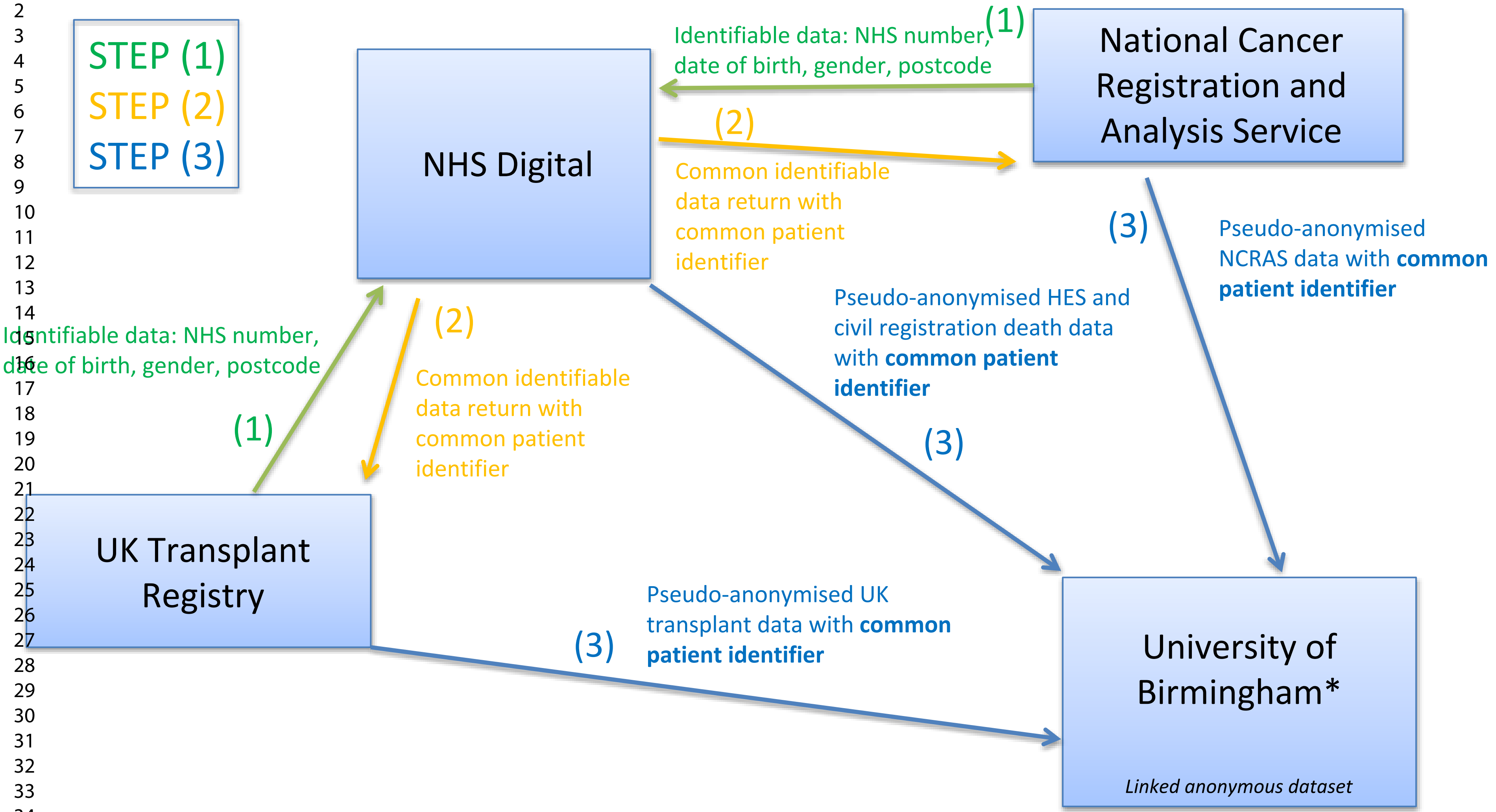

*University of Birmingham is the Data Processor. University Hospitals Birmingham NHS Foundation Trust is the Data Controller but will not receive (or access) any data at any time point.

BMJ Open

Protocol: observational cohort study exploring the Epidemiology of Cancer after solid Organ Transplantation (EpCOT)

Journal:	*BMJ Open*
Manuscript ID	bmjopen-2020-043731.R1
Article Type:	Protocol
Date Submitted by the Author:	13-Jan-2021
Complete List of Authors:	Sharif, Adnan; University Hospitals Birmingham NHS Foundation Trust, Renal Medicine<br>Peracha, Javeria; University Hospitals Birmingham NHS Foundation Trust, Renal Medicine<br>Winter, David; University of Birmingham<br>Reulen, Raoul; University of Birmingham<br>Hawkins, Mike; University of Birmingham, School of Health and Population Sciences
**Primary Subject Heading**:	Epidemiology
Secondary Subject Heading:	Surgery, Renal medicine, Oncology
Keywords:	TRANSPLANT SURGERY, TRANSPLANT MEDICINE, Transplant surgery < SURGERY, ONCOLOGY, Adult oncology < ONCOLOGY

SCHOLARONE™
Manuscripts

I, the Submitting Author has the right to grant and does grant on behalf of all authors of the Work (as defined in the below author licence), an exclusive licence and/or a non-exclusive licence for contributions from authors who are: i) UK Crown employees; ii) where BMJ has agreed a CC-BY licence shall apply, and/or iii) in accordance with the terms applicable for US Federal Government officers or employees acting as part of their official duties; on a worldwide, perpetual, irrevocable, royalty-free basis to BMJ Publishing Group Ltd ("BMJ") its licensees and where the relevant Journal is co-owned by BMJ to the co-owners of the Journal, to publish the Work in this journal and any other BMJ products and to exploit all rights, as set out in our licence.

The Submitting Author accepts and understands that any supply made under these terms is made by BMJ to the Submitting Author unless you are acting as an employee on behalf of your employer or a postgraduate student of an affiliated institution which is paying any applicable article publishing charge ("APC") for Open Access articles. Where the Submitting Author wishes to make the Work available on an Open Access basis (and intends to pay the relevant APC), the terms of reuse of such Open Access shall be governed by a Creative Commons licence – details of these licences and which Creative Commons licence will apply to this Work are set out in our licence referred to above.

Other than as permitted in any relevant BMJ Author's Self Archiving Policies, I confirm this Work has not been accepted for publication elsewhere, is not being considered for publication elsewhere and does not duplicate material already published. I confirm all authors consent to publication of this Work and authorise the granting of this licence.

For peer review only

Title. Protocol: observational cohort study exploring the Epidemiology of Cancer after solid Organ Transplantation (EpCOT)

Author. Adnan Sharif[1,2], Javeria Peracha[1], David L Winter[3], Raoul C Reulen[3], Mike M Hawkins[3]

Address.

[1]Department of Nephrology and Transplantation, Queen Elizabeth Hospital, University Hospitals Birmingham, Edgbaston, Birmingham, United Kingdom

[2]Institute of Immunology and Immunotherapy, University of Birmingham, Edgbaston, Birmingham, UK

[3]Institute of Applied Health Research, University of Birmingham, Edgbaston, Birmingham, UK

Corresponding author contact information.

Dr. Adnan Sharif

Department of Nephrology and Transplantation

Queen Elizabeth Hospital, University Hospitals Birmingham

Mindelsohn Way, Edgbaston

Birmingham, B15 2GW, United Kingdom

Phone: (0121 371 5861), Fax: (0121 371 5858), Email: adnan.sharif@uhb.nhs.uk, Twitter: @AdnanSharif1979, Orchid ID: 0000-0002-7586-9136

Word count. 4,065

For peer review only - http://bmjopen.bmj.com/site/about/guidelines.xhtml

Abstract

Introduction. Solid organ transplant patients are counselled regarding increased risk for cancer (principally due to their need for lifelong immunosuppression) and it ranks as one of their biggest self-reported worries. Post-transplantation cancer is common, associated with increased healthcare costs and emerging as a leading cause of post-transplant mortality. However, epidemiology of cancer post-transplantation remains poorly understood, with limitations including translating data from different countries and national data being siloed across different registries and/or data warehouses.

Methods and analysis. Study methodology for EpCOT involves record linkage between the UK Transplant Registry (from NHS Blood and Transplant), Hospital Episode Statistics (for secondary care episodes from NHS Digital), national cancer registry (from cancer registration data hosted by Public Health England) and the national death registry (from NHS Digital). Deterministic record linkage will be conducted by NHS Digital, with a fully anonymised linked dataset available for analysis by the research team. The study cohort will consist of up to 85,410 solid organ transplant recipients, who received a solid organ transplant in England between January 1st 1985 and December 31st 2015, with up-to-date outcome data.

Ethics and dissemination. This study has been approved by the Confidentiality Advisory Group (reference: 16/CAG/0121), Research Ethics Committee (reference: 15/YH/0320) and institutional review board (reference: RRK5471). The results of this study will be presented at national and international conferences, and manuscripts with results will be submitted for publication in high-impact peer-reviewed journals. The information produced will also be

For peer review only

For peer review only - http://bmjopen.bmj.com/site/about/guidelines.xhtml

used to develop national evidence-based clinical guidelines to inform risk stratification to enable risk-based clinical follow up.

Registration. This observational study is registered with clinicaltrials.org (NCT02991105).

Strengths and limitations of this study

- Population-based cohort linked dataset of all solid organ transplant recipients in England to provide comprehensive coverage
- A large, retrospective analysis using national linked datasets of solid organ transplant recipients at risk for developing cancer
- Robust and collaborative data linkage of routinely collected administrative data, for the purpose of exploring important clinical outcomes of research interest post-transplantation
- Missing variables and confounding data may limit interpretation of the analysis
- Data is England-specific and may not be translatable to other cohorts

For peer review only

For peer review only - http://bmjopen.bmj.com/site/about/guidelines.xhtml

Introduction

Solid organ transplantation is established therapy for patients with end-stage organ dysfunction, providing lifesaving (e.g. heart, lung, liver) or life enhancing (e.g. kidney, pancreas) treatment. However, cancer is now emerging as one of the leading complications after solid organ transplantation. Transplant recipients have an increased risk of cancer compared with that expected from the general population [1-3] and comparable risk to immunodeficiency states such as HIV/AIDS [4], with similar preponderance for cancers with a viral component [5]. Multi-factorial aetiology underlies cancer risk after solid organ transplantation but immunosuppression, compulsory to prevent allograft rejection, contributes significantly to cancer risk [6, 7]. Standardised incidence ratio for development of all post-transplantation cancers (excluding non-melanoma skin cancers) remain constantly increased starting from 2-years post-transplantation in the UK [3], meaning the relative excess risk of cancer increases with time. Transplant recipients are achieving better long-term survival [8] and, among kidney transplant patients with more than 20-years graft function, *de novo* cancer develops in 37% of patients [9]. This is likely due to improved post-transplant longevity leading to increased cumulative exposure to cancer risks such as immunosuppression. In summary, post-transplantation cancer is now the leading cause of death after kidney transplantation in the UK [10], death from cancer post-transplantation is in excess of that expected from the general population [11-14], it ranks as one of the biggest worries for post-transplant patients [15] and development of post-transplantation cancer is associated with increased healthcare cost [16].

For peer review only - http://bmjopen.bmj.com/site/about/guidelines.xhtml

Some reports on cancer epidemiology after solid organ transplantation focus on historical eras and practice has changed over the last decade. Immunosuppression is significantly different, especially since 2006 when dissemination of the SYMPHONY study results led to switches from ciclosporin to tacrolimus-based immunosuppression [17]. This is important as tacrolimus has been shown in liver transplant recipients to increase risk for post-transplantation cancer compared to ciclosporin [18], with an observed dose-effect relationship between tacrolimus and post-transplantation cancer [19]. Cancer risk appears decreased with sirolimus after kidney transplantation [20, 21], increased with azathioprine after solid organ transplantation [22-24] but some studies (limited to kidney transplantation) suggest no difference in cancer-risk between immunosuppressant agents [25]. Induction immunosuppression is also more common used; while T-cell depletion induction has been linked with increased risk for post-transplantation cancer [26], induction therapy can lead to reduced exposure to maintenance immunosuppression further confounding cancer risk assessment [27]. Evolving immunosuppression, changing transplant practice and selection of solid organ donors/recipients with different characteristics justifies a more contemporary and time-dependent analysis of post-transplantation cancer.

Data related to the epidemiology of post-transplantation cancer predominantly comes from outside the UK (e.g. the US) but this may not be directly translatable. Immunosuppression use, the biggest modifiable risk factor for post-transplantation cancer [28], differs between the US and UK. For example, T-cell depletion therapy is the predominant induction agent in the US versus non T-cell depletion (e.g. basiliximab) in the UK [29]. Cumulative exposure to immunosuppression will be different, with financial coverage limitations in the US extending

For peer review only - http://bmjopen.bmj.com/site/about/guidelines.xhtml

to immunosuppression provision [30] and these factors could possibly contribute to inferior post-transplant outcomes observed in the US compared to Europe [31, 32]. Kidney allograft recipients in England have more pre-transplant cancer history, more post-transplant cancer occurrence but superior overall graft survival and all-cause mortality when compared to contemporaneous recipients in New York State [33], suggesting post-transplantation cancer risk (and outcomes) are not comparable. Study cohorts from Australia and/or New Zealand are more aligned to UK practice from an immunosuppression perspective. However, they have different demographics (e.g., less minority ethnic recipients) and burden of medical co-morbidities (e.g., cause of kidney failure) that limits direct translation of outcomes [34]. With only two epidemiology studies focused on post-transplantation cancer in the UK [3, 13], further studies in the UK are urgently warranted to inform patients and professionals.

Absence of relevant epidemiological data impacts upon patient counselling regarding increased risk for post-transplantation cancer, which is accepted by transplant candidates as a recognised complication from immunosuppression. However, individual risk stratification is not possible and this has implications in the contemporary era of consent [35]. Personalised counselling is difficult as we cannot predict individual post-transplant cancer risk and screening is only available for a minority of cancers. Gaining a clearer understanding of post-transplantation cancer risk, morbidity and mortality for specific transplant groups/cohorts will aid transplant discussions. This is especially important for cancers of greater incidence and risk of adverse outcomes after solid organ transplantation (e.g., post-transplant lymphoproliferative disorders). KDIGO (Kidney Disease Improving Global Outcomes) guidelines for the care of (kidney) transplant patients report very poor level evidence when it

For peer review only - http://bmjopen.bmj.com/site/about/guidelines.xhtml

comes to cancer, with either level 2C or non-graded evidence [36]. This translates into lack of consensus on cancer screening advice post-transplantation [37], lack of evidence-based guidance on how best to manage development of post-transplant cancer [38] and overall poor outcomes post-transplantation if transplant recipients develop cancer [11-13].

To understand the epidemiology of post-transplant cancer in the UK, it is imperative to bring together multiple sources of information relating to the same solid organ transplant recipient who may develop cancer contained across different registries or datasets. Big data linkage, the process of bringing together and linking large volumes of healthcare records, can help identify factors and associations that would otherwise be difficult to robustly determine. The ability to link data can significantly increase the value that can be derived from individual datasets – which are often collected for specific regulatory purposes with considerable effort and expense – and maximise research benefit for patients. To that effect, Epidemiology of Cancer after solid Organ Transplantation (EpCOT) has the aim of exploring prespecified research questions of importance to the solid organ transplant community utilising big data record linkage of national healthcare datasets. The study protocol is registered with clinicaltrials.org (NCT02991105) and study results will be reported in accordance with STROBE guidelines [39].

Methods and Analysis

This epidemiological study will undertake record linkage between national datasets to link up the pathway from solid organ transplantation and subsequent risk for developing cancer. Deterministic record linkage will be undertaken by NHS Digital using identifiable patient data (specifically NHS number, date of birth, sex and postcode) between the following datasets:

For peer review only - http://bmjopen.bmj.com/site/about/guidelines.xhtml

(i) UK Transplant Registry (UKTR)

The UKTR is maintained by NHS Blood and Transplant and holds data for all solid organ transplants performed in the UK, with data submission mandatory for transplant centres. UKTR will provide data for the central study cohort of 85,410 patients who received a solid organ transplant between 1st January 1985 and 31st December 2015 in England, with donor-, recipient- and transplant-specific variables. An overview of the cohort is provided in Table 1, with key demographics shown in Table 2 and the data dictionary in Supplementary File 1. The final cohort for analysis will be determined after excluding any repeat transplant recipients, non-English residents who received their transplant in England and anyone who refused consent for their data to be used.

(ii) Civil registration deaths (NHS Digital)

National death registration data is held by NHS Digital. For those patients that have died, the date and underlying cause of death from death certificate codes will be obtained for any death occurring in the United Kingdom (any deaths occurring outside of the United Kingdom will be missed but date of emigration [loss to follow up] will be provided). For date of death only a flag indicating that death has occurred and the months from transplantation to death (to calculate time interval) will be processed in line with legal approval.

(iii) National cancer registry - National Cancer Registration and Analysis Service (NCRAS) from Public Health England (PHE)

For peer review only - http://bmjopen.bmj.com/site/about/guidelines.xhtml

NCRAS collects information about every patient diagnosed with cancer in England. All variables of cancer-related interest will be linked to our cohort (between 1st January 1986 and 31st December 2017 unless stated otherwise) and include; cancer site (C00c-C097x and D00-D48), histology, treatment, chemotherapy (between 1st April 2012 and 31st March 2018) and radiotherapy (between 1st April 2009 and 31st March 2019). The data dictionary is provided in Supplementary File 2.

(iv) *Hospital Episode Statistics (HES)*

HES is an administrative dataset collating information on all secondary care events in England, held by NHS Digital. From HES we can determine adverse health outcomes or procedures requiring hospitalisation (e.g. inpatient, outpatient and A&E attendance), for which the risk may be increased as a result of having had a solid organ transplant. Period at risk of adverse health outcomes would be from 1997 until the most recent date for which HES is known to be complete. The data dictionary is provided in Supplementary File 3.

Record linkage methodology

Data linkage methodology will involve UK Transplant Registry and NCRAS sending to NHS Digital only a list of identifying details for their respective cohorts. NHS Digital will link the UK Transplant Registry and NCRAS cohorts, determining which patients are common in both data sets. NHS Digital will then provide a list of pseudonymous IDs of patients common in both data sets to UK Transplant Registry and NCRAS respectively. This will facilitate extraction of clinical/health data from their data sets respectively, for provision to the University of Birmingham identifiable by the pseudonymous ID only. NHS Digital will facilitate linkage to

For peer review only - http://bmjopen.bmj.com/site/about/guidelines.xhtml

HES and 'Civil Registration – Deaths' to extract records and supply anonymised records to University of Birmingham with the common pseudonymous ID. The University of Birmingham will then facilitate linkage between the NHS Digital, UKTR and NCRAS datasets using the pseudonymous ID common to all datasets. See Figure 1 for a summary of data flows for the EpCOT study.

Data security and governance

There will be no need, requirement or possibility to re-identify individuals after record linkage by the University of Birmingham (acting as Data Processor). Received data will be stored on a separate array of disks and accessed on a mapped drive as part of the University of Birmingham campus network. Access to this mapped drive will be limited to the named University researchers who can only access the University of Birmingham campus network with a personalised username/password combination. The personal computers used to access the data will have additional virus checking/data security software installed (Malwarebytes). The separate array of disks will reside in a controlled environment with access only by accredited University IT staff. The data from this disk array will be backed up under a separate storage policy, which once deleted will make the data unavailable for restore.

For peer review only

The linkage, processing and analysis of the data from UKTR, NCRAS and NHS Digital will only be carried out the specified researchers within the University of Birmingham as the Data Processor who are appropriately trained in data protection and confidentiality. There will be no attempt made to link any data requested under this application to any held under other Data Sharing Agreements. This database of the pseudonymised data will be accessible only to

For peer review only - http://bmjopen.bmj.com/site/about/guidelines.xhtml

named personnel within the project and will be kept in auditable documents. In addition, analysts fulfilling database administrator role (who are substantive members of the University of Birmingham) will also have access to the database but undertake no processing. At the end of the project, the data will be destroyed in line with strict policies and procedures on the destruction of data. No record level information will leave the Data Processor or aggregated data without small number suppression in line with the NHS Digital HES Analysis Guide.

Prespecified research questions

1. To compare observed and expected risks of specific causes of deaths, in particular cancer-related death, by linking the UKTR with the national death registry to obtain underlying causes of death and determine factors related to increased risk of specific causes of death post-transplantation. General population mortality rates will be used to calculate expected number of deaths from specific causes and identify subgroups of post-transplant patients (e.g. age, sex, ethnicity, organ type, etc.) at excess risk compared with expected risk.

2. Investigate survival and causes of death after cancer in post-transplant patients versus individuals from the general population with a similar *de novo* cancer of the same age, sex, and calendar year of diagnosis.

3. Compare observed and expected risks of specific cancer types post-transplantation by linking the UKTR with the national cancer registry to obtain observed numbers of cancers and determine factors related to increased risk of specific types of cancer. General population cancer incidence rates will be used to calculate expected numbers of cancers

For peer review only - http://bmjopen.bmj.com/site/about/guidelines.xhtml

of specific type and identify subgroups of post-transplant patients at excess risk of specific cancers compared with expected.

4. Estimate risk of morbidity requiring hospitalisation both generally and that associated with development of post-transplantation cancer by linking the UKTR with Hospital Episode Statistics (HES). Risk of hospital admissions and procedures (e.g. surgery) for specific morbidities will be investigated. We will calculate expected risks for specific conditions requiring hospitalisation, enabling identification of specific subgroups of post-transplant patients at excess risk compared with expected.

Detailed research plan

1: Compare observed and expected risks of specific causes of death

Each of the patients in the UKTR enters risk at the date of transplantation and contributes person-years until the exit date. Standardised mortality ratios (SMRs) and absolute excess risks (AERs) will be calculated as O/E and [(O – E)/ py]*1000, where 'O' and 'E' are the observed and expected numbers of deaths respectively and 'py' is the total person-years at risk. To examine variation in SMRs or AERs across factors (e.g. age, sex, ethnicity, organ type, period of follow-up, calendar year of diagnosis), multivariable Poisson regression models will be utilised. Cumulative incidence of deaths from specific causes, in particular cancer, will be estimated (treating other causes of death as competing risks). All statistical analyses will use STATA 16 unless otherwise stated.

2: Investigate survival and causes of death after cancer in transplant patients

For peer review only - http://bmjopen.bmj.com/site/about/guidelines.xhtml

Comparators will be individuals from the general population with a similar *de novo* cancer of the same age, sex and calendar year of diagnosis. On one hand, heightened surveillance among solid organ transplant survivors should detect any cancers at an early stage and, therefore, reduce cancer-related mortality. On the other hand, previous treatment with immunosuppressants may limit treatment options for cancer and hence increase cancer-specific mortality. Survival comparisons will use Cox regression. We shall compare all-cause and cause-specific mortality of patients who have undergone a solid organ transplant and developed a cancer with that of individuals who developed a similar *de novo* cancer using Cox regression and/or Poisson regression analysis. An (anonymous) sample of patients from the general population who developed cancer will be obtained from NCRAS.

3: Compare observed and expected risks of specific cancer types

Data from NCRAS will identify diagnosis of post-transplantation cancers in terms of date and site/type. The initial analysis will involve constructing a table with rows corresponding to variables relating to characteristics (e.g. age, sex, organ etc.) of all transplant patients and the columns corresponding to the cancer site/types. Observed and expected numbers in each cell of this table will identify any evidence of an excess. Poisson regression as described above will be used to determine subgroups of patients at a substantially increased risk of specific cancers.

We will also compare cancer risk for transplant patients treated before/after 2006 (introduction of tacrolimus-based regimens) to evaluate whether more recently treated patients have increased/decreased post-transplantation cancer risk.

For peer review only - http://bmjopen.bmj.com/site/about/guidelines.xhtml

4: Estimate risk of morbidity requiring hospitalisation

Two types of analyses will be possible using UKTR-HES linked data:

A) Internal analyses: Risk of specific adverse outcomes post-transplantation will be compared over the period at risk using Poisson regression, to identify particular subgroups (e.g. in terms of attained age, sex, type of transplant, age at diagnosis, period of follow-up and calendar year of diagnosis) at greatest risk of the specific adverse health outcomes.

B) External analyses: Obtaining general population HES events classified by age, sex and calendar year and dividing by appropriate general population estimates will enable external comparisons. We will use Poisson regression to compare risk of adverse health outcomes, over the period at risk, between transplant patients and the general population adjusting for attained age, sex and calendar year in the model. Patient sub-groups (defined in terms of attained age, sex, type of transplant, age at diagnosis, period of follow-up and calendar year of diagnosis) at substantial excess risk compared with expected will thus be identified.

Complementary to our analyses specified above, a data-driven methodology incorporating latest developments in Artificial Intelligence (e.g. machine learning) will be explored, enabling us to rank the importance of predictors for post-transplantation cancer if possible. By developing and applying models to approximate complex and yet-unknown interactions between clinical features, better prediction of patient outcomes could inform certain streams of post-transplantation care.

For peer review only - http://bmjopen.bmj.com/site/about/guidelines.xhtml

Analytical considerations

Adequate power for study events

In previous work by Collett et al. [3], 37,617 solid organ transplant recipients in the UK were analysed, whilst in the current proposal we include more than double (N=85,410, although final cohort after exclusions still to be determined). Using a conservative approach, assuming number of expected cancers will be twice that from previous work by Collett and colleagues (although expected number will likely be much greater as the cohort has aged), then we will be able to detect SIRs of at least the following magnitudes with 80% power when using a 5% significance level (see Table 3).

Thus, apart from cancer sites of anus, lip and Kaposi sarcoma which are the commonest occurring, we should be able to detect an SIR of 2.0 or less with at least 80% statistical power. Similar logic would apply to our analyses relating to hospitalisations and mortality.

Missing data

Missing data is a reality of both administrative and registry data. Examples of the degree of missing data from the UKTR has been indicated in Table 2, but the final amount after record linkage is undertaken will not be known and in some instances could be reduced (e.g. missing ethnicity data in UKTR may be available from HES or NCRAS which also contain this data field).

Our approach for dealing with missing data will be to only analyse the available data (i.e. ignoring the missing data) and conduct sensitivity analyses to explore whether missing data

For peer review only - http://bmjopen.bmj.com/site/about/guidelines.xhtml

could have biased our results; or, where appropriate, to impute missing data and account for the fact that missing values were imputed with uncertainty (e.g. multiple imputation).

Quality assurance for submitted data and record linkage

Under our section 251 approval, record linkage will be facilitated by NHS Digital. The research team is therefore reliant on robust quality assurance mechanisms from both data senders and NHS Digital Data Linkage and Extract Services to ensure a robust and comprehensive record linkage process. We have worked very closely with all partners, carefully selecting fields from the available data dictionaries and checking their suitability and viability for record linkage. Cost recovery frameworks have been factored into our budget to ensure robust data extraction and quality assurance processes are in place. NHS Digital charges also includes list cleaning, a validation process using demographic data to ensure accuracy and improve linkage outcomes. A record linkage report will be available to check record linkage success rate.

Patient and Public involvement (PPI)

Patient involvement has been critical in the design of our research aims, to ensure our research questions are both relevant and appropriate. We have developed our research aims after discussion with key stakeholders like patients to ensure we are seeking answers to questions that they wish to be answered and further study into post-transplantation cancer meets those needs. Our departmental PPI forum involving transplant candidates and patients, hosted as part of the Trust's initiative to ensure adequate patient and public representation in research drives, has confirmed qualitative study findings that post-transplantation cancer is a leading concern and an area where preventative strategies would be overwhelmingly

For peer review only - http://bmjopen.bmj.com/site/about/guidelines.xhtml

welcomed. Patients were very receptive to our strategy of record linkage, both using anonymised national data resources and also using linked primary/secondary care data after informed consent if required for any future extension work.

Ethics and dissemination

Approvals for record linkage

The EpCOT study research plan has been reviewed and approved by our data contributing partners NHS Digital, the UK Transplant Registry and Public Health England. Their support, collaboration and advice has been critical for the development of data flows and we plan to work in close partnership at various stages of the project. Our wide consortium of partners has demonstrated their strong support to ensure the success of this project over many years of development.

The EpCOT project has attained institutional (*RRK5471*) and ethical (*15/YH/0320*) approval. The project also has section 251 approval from the Confidentiality Advisory Group (*16/CAG/0121*), under Regulation 5 of the Health Service (Control of Patient Information) Regulations 2002, for the record linkage to proceed by NHS Digital.

Dissemination plan

The aim of this project is to produce targeted studies looking at the epidemiology of post-transplantation cancer, and this data will be disseminated through presentations at national/international congresses and submitted for publication in leading medical journals.

For peer review only - http://bmjopen.bmj.com/site/about/guidelines.xhtml

The aim is also to develop Standards of Care guidelines, working with the Standards Committee of the British Transplantation Society to provide evidence-based clinical evidence for direct patient benefit and counselling. These will be made freely available to the community at their website (https://bts.org.uk/guidelines-standards/). It is also the aim to plan patient-focused dissemination in plain English through various channels of communication for patients and the general public.

Outputs will only contain aggregate data with small numbers suppressed in line with the NHS Digital HES Analysis Guide. Both NHS Blood and Transplant and Public Health England (who will provide the initial data cohorts on this project) have mechanisms for dissemination of information to both professional and general population and we will use these to share the results from EpCOT widely. We also aim to do similar research presentation at Research Open Days at both University Hospitals Birmingham and the University of Birmingham. Summary research findings are actively disseminated via social media channels (e.g. @AdnanSharif1979; @UHBResearch; @ImmunologyUoB), while tagging our collaborative partners (@NHSBT_RD; @PHE_uk; @NHSDigital). Project partners will be encouraged to disseminate their involvement through all social media channels.

For peer review only

Discussion

Large epidemiological studies from the US (using data from the Transplant Cancer Match Study) have recently reported post-transplant cancer epidemiology outcomes for a cohort of 221,962 solid organ transplant recipients, of whom 15,012 developed cancer [40]. One of the main findings from Noone and colleagues is cancer-related mortality increases steadily with post-transplantation time duration, reaching 15.7% of deaths (810 per 100,000 person-years)

For peer review only - http://bmjopen.bmj.com/site/about/guidelines.xhtml

after 10 or more years after transplantation. While a large cohort, one of the limitations of this dataset is the incomplete national coverage; a total of 17 cancer registries provided data regarding incident cancers (excluding nonmelanoma skin cancer) that covers approximately one-half of the US transplant population with varying years of coverage data. To the best of our knowledge, EpCOT is one of the largest population-cohort studies exploring cancer epidemiology after solid organ transplantation. Using record linkage of national datasets with extended years of coverage, this project will provide detailed information for solid organ transplant recipients with cancer-related coverage at a countrywide level.

In order to fulfil its regulatory mandate, NHS Blood and Transplant (NHSBT) collects data for all solid organ transplant recipients including long-term follow-up information. The collection of national data enables NHSBT to fulfil its statutory obligations with regard to the effective use of organs, equitable organ offering and performance monitoring of transplant centres in terms of patient and graft outcomes. Such data has tremendous analytical potential and is available for research purposes with appropriate approval. However, the exploration of certain outcomes like cancer is limited as this information is not routinely collected by NHSBT. To overcome this issue, record linkage between the transplant registry and national cancer data is essential. The methodology of EpCOT facilitates this, with the inclusion of all secondary care episodes and death registrations, to obtain a robust, comprehensive and informative population-cohort study of post-transplantation cancer. From an epidemiological perspective, this allows detailed analyses to assess the true impact of cancer for solid organ transplant recipients and provides coverage of the full patient journey from transplantation to cancer-related outcomes.

For peer review only - http://bmjopen.bmj.com/site/about/guidelines.xhtml

For peer review only

In summary, cancer is a leading cause of morbidity and mortality after solid organ transplantation. With incidence of cancer projected to increase, improving our understanding of post-transplant cancer epidemiology is essential to improve long-term patient outcomes. EpCOT will be one of the largest record linkages of solid organ transplant recipients, with contemporary and comprehensive national cancer-related outcomes, to provide answers to questions of importance to both transplant professionals and organ recipients respectively.

References:

1. Engels EA, Pfeiffer RM, Fraumeni JF, Jr., Kasiske BL, Israni AK, Snyder JJ, Wolfe RA, Goodrich NP, Bayakly AR, Clarke CA *et al*: **Spectrum of cancer risk among US solid organ transplant recipients**. *JAMA* 2011, **306**(17):1891-1901.
2. Kasiske BL, Snyder JJ, Gilbertson DT, Wang C: **Cancer after kidney transplantation in the United States**. *Am J Transplant* 2004, **4**(6):905-913.
3. Collett D, Mumford L, Banner NR, Neuberger J, Watson C: **Comparison of the incidence of malignancy in recipients of different types of organ: a UK Registry audit**. *Am J Transplant* 2010, **10**(8):1889-1896.
4. Grulich AE, van Leeuwen MT, Falster MO, Vajdic CM: **Incidence of cancers in people with HIV/AIDS compared with immunosuppressed transplant recipients: a meta-analysis**. *Lancet* 2007, **370**(9581):59-67.
5. Morath C, Mueller M, Goldschmidt H, Schwenger V, Opelz G, Zeier M: **Malignancy in renal transplantation**. *J Am Soc Nephrol* 2004, **15**(6):1582-1588.
6. Vajdic CM, van Leeuwen MT: **Cancer incidence and risk factors after solid organ transplantation**. *Int J Cancer* 2009, **125**(8):1747-1754.
7. Geissler EK: **Post-transplantation malignancies: here today, gone tomorrow?** *Nat Rev Clin Oncol* 2015, **12**(12):705-717.
8. **UK Transplant Activity Report 2019-2019. Accessed 5th September 2019 at; https://nhsbtdbe.blob.core.windows.net/umbraco-assets-corp/16469/organ-donation-and-transplantation-activity-report-2018-2019.pdf** Accessed 29th May 2018.
9. McCaughan JA, Courtney AE: **The clinical course of kidney transplant recipients after 20 years of graft function**. *Am J Transplant* 2015, **15**(3):734-740.
10. Steenkamp R, Rao A, Fraser S: **UK Renal Registry 18th Annual Report (December 2015) Chapter 5: Survival and Causes of Death in UK Adult Patients on Renal Replacement Therapy in 2014: National and Centre-specific Analyses**. *Nephron* 2016, **132 Suppl 1**:111-144.

For peer review only - http://bmjopen.bmj.com/site/about/guidelines.xhtml

11. Na R, Grulich AE, Meagher NS, McCaughan GW, Keogh AM, Vajdic CM: **De novo cancer-related death in Australian liver and cardiothoracic transplant recipients**. *Am J Transplant* 2013, **13**(5):1296-1304.
12. Acuna SA, Fernandes KA, Daly C, Hicks LK, Sutradhar R, Kim SJ, Baxter NN: **Cancer Mortality Among Recipients of Solid-Organ Transplantation in Ontario, Canada**. *JAMA Oncol* 2016, **2**(4):463-469.
13. Farrugia D, Mahboob S, Cheshire J, Begaj I, Khosla S, Ray D, Sharif A: **Malignancy-related mortality following kidney transplantation is common**. *Kidney Int* 2014, **85**(6):1395-1403.
14. Kiberd BA, Rose C, Gill JS: **Cancer mortality in kidney transplantation**. *Am J Transplant* 2009, **9**(8):1868-1875.
15. Howell M, Tong A, Wong G, Craig JC, Howard K: **Important outcomes for kidney transplant recipients: a nominal group and qualitative study**. *Am J Kidney Dis* 2012, **60**(2):186-196.
16. Dharnidharka VR, Naik AS, Axelrod D, Schnitzler MA, Xiao H, Brennan DC, Segev DL, Randall H, Chen J, Kasiske B *et al*: **Clinical and Economic Consequences of Early Cancer After Kidney Transplantation in Contemporary Practice**. *Transplantation* 2017, **101**(4):858-866.
17. Ekberg H, Tedesco-Silva H, Demirbas A, Vitko S, Nashan B, Gurkan A, Margreiter R, Hugo C, Grinyo JM, Frei U *et al*: **Reduced exposure to calcineurin inhibitors in renal transplantation**. *N Engl J Med* 2007, **357**(25):2562-2575.
18. Wimmer CD, Angele MK, Schwarz B, Pratschke S, Rentsch M, Khandoga A, Guba M, Jauch KW, Bruns C, Graeb C: **Impact of cyclosporine versus tacrolimus on the incidence of de novo malignancy following liver transplantation: a single center experience with 609 patients**. *Transpl Int* 2013, **26**(10):999-1006.
19. Carenco C, Assenat E, Faure S, Duny Y, Danan G, Bismuth M, Herrero A, Jung B, Ursic-Bedoya J, Jaber S *et al*: **Tacrolimus and the risk of solid cancers after liver transplant: a dose effect relationship**. *Am J Transplant* 2015, **15**(3):678-686.
20. Yanik EL, Siddiqui K, Engels EA: **Sirolimus effects on cancer incidence after kidney transplantation: a meta-analysis**. *Cancer Med* 2015, **4**(9):1448-1459.
21. Sharif A: **Sirolimus after kidney transplantation**. *BMJ* 2014, **349**:g6808.
22. Jiyad Z, Olsen CM, Burke MT, Isbel NM, Green AC: **Azathioprine and Risk of Skin Cancer in Organ Transplant Recipients: Systematic Review and Meta-Analysis**. *Am J Transplant* 2016, **16**(12):3490-3503.
23. Na R, Laaksonen MA, Grulich AE, Meagher NS, McCaughan GW, Keogh AM, Vajdic CM: **Iatrogenic immunosuppression and risk of non-Hodgkin lymphoma in solid organ transplantation: A population-based cohort study in Australia**. *Br J Haematol* 2016, **174**(4):550-562.
24. Na R, Laaksonen MA, Grulich AE, Meagher NS, McCaughan GW, Keogh AM, Vajdic CM: **High azathioprine dose and lip cancer risk in liver, heart, and lung transplant recipients: A population-based cohort study**. *J Am Acad Dermatol* 2016, **74**(6):1144-1152 e1146.
25. Gallagher MP, Kelly PJ, Jardine M, Perkovic V, Cass A, Craig JC, Eris J, Webster AC: **Long-term cancer risk of immunosuppressive regimens after kidney transplantation**. *J Am Soc Nephrol* 2010, **21**(5):852-858.

For peer review only - http://bmjopen.bmj.com/site/about/guidelines.xhtml

26. Hall EC, Engels EA, Pfeiffer RM, Segev DL: **Association of antibody induction immunosuppression with cancer after kidney transplantation**. *Transplantation* 2015, **99**(5):1051-1057.
27. Wiseman AC: **Induction Therapy in Renal Transplantation: Why? What Agent? What Dose? We May Never Know**. *Clin J Am Soc Nephrol* 2015, **10**(6):923-925.
28. Gutierrez-Dalmau A, Campistol JM: **Immunosuppressive therapy and malignancy in organ transplant recipients: a systematic review**. *Drugs* 2007, **67**(8):1167-1198.
29. Hardinger KL, Brennan DC, Klein CL: **Selection of induction therapy in kidney transplantation**. *Transpl Int* 2013, **26**(7):662-672.
30. Gill JS, Tonelli M: **Penny wise, pound foolish? Coverage limits on immunosuppression after kidney transplantation**. *N Engl J Med* 2012, **366**(7):586-589.
31. Ojo AO, Morales JM, Gonzalez-Molina M, Steffick DE, Luan FL, Merion RM, Ojo T, Moreso F, Arias M, Campistol JM *et al*: **Comparison of the long-term outcomes of kidney transplantation: USA versus Spain**. *Nephrol Dial Transplant* 2013, **28**(1):213-220.
32. Gondos A, Dohler B, Brenner H, Opelz G: **Kidney graft survival in Europe and the United States: strikingly different long-term outcomes**. *Transplantation* 2013, **95**(2):267-274.
33. Jackson-Spence F, Gillott H, Tahir S, Nath J, Mytton J, Evison F, Sharif A: **Cancer-related outcomes in kidney allograft recipients in England versus New York State: a comparative population-cohort analysis between 2003 and 2013**. *Cancer Med* 2017, **6**(3):563-571.
34. Au EH, Chapman JR, Craig JC, Lim WH, Teixeira-Pinto A, Ullah S, McDonald S, Wong G: **Overall and Site-Specific Cancer Mortality in Patients on Dialysis and after Kidney Transplant**. *J Am Soc Nephrol* 2019.
35. Lee A: **'Bolam' to 'Montgomery' is result of evolutionary change of medical practice towards 'patient-centred care'**. *Postgrad Med J* 2017, **93**(1095):46-50.
36. Kidney Disease: Improving Global Outcomes Transplant Work G: **KDIGO clinical practice guideline for the care of kidney transplant recipients**. *Am J Transplant* 2009, **9 Suppl 3**:S1-155.
37. Acuna SA, Huang JW, Scott AL, Micic S, Daly C, Brezden-Masley C, Kim SJ, Baxter NN: **Cancer Screening Recommendations for Solid Organ Transplant Recipients: A Systematic Review of Clinical Practice Guidelines**. *Am J Transplant* 2017, **17**(1):103-114.
38. Ajithkumar TV, Parkinson CA, Butler A, Hatcher HM: **Management of solid tumours in organ-transplant recipients**. *Lancet Oncol* 2007, **8**(10):921-932.
39. von Elm E, Altman DG, Egger M, Pocock SJ, Gotzsche PC, Vandenbroucke JP, Initiative S: **Strengthening the Reporting of Observational Studies in Epidemiology (STROBE) statement: guidelines for reporting observational studies**. *BMJ* 2007, **335**(7624):806-808.
40. Noone AM, Pfeiffer RM, Dorgan JF, Magder LS, Bromberg JS, Lynch CF, Morris CR, Pawlish KS, Engels EA: **Cancer-attributable mortality among solid organ transplant recipients in the United States: 1987 through 2014**. *Cancer* 2019, **125**(15):2647-2655.

For peer review only

For peer review only - http://bmjopen.bmj.com/site/about/guidelines.xhtml

Authors' Contributions:

Adnan Sharif: Substantial contributions to the conception, design of the work, seeking of funding, acquisition, analysis, interpretation of data, drafting the work, revising it critically and final approval of the version published

Javeria Peracha: Substantial contributions to the design of the work, analysis of data, interpretation of data, critical revision and final approval of the version published

David L Winter: Substantial contributions to the design of the work, acquisition of data, storage of data, critical revision and final approval of the version published

Raoul C Reulen: Substantial contributions to the conception, design of the work, acquisition, analysis, interpretation of data, revising it critically and final approval of the version published

Mike M Hawkins: Substantial contributions to the conception, design of the work, acquisition, analysis, interpretation of data, revising it critically and final approval of the version published

Funding Statement: This work was supported by a Wellcome Trust Institutional Strategic Support Fund: Digital Health Pilot Grant (419195-419186-42231786) and the Kildare Trust (no award number applicable).

Competing Interests Statement: No competing interests.

For peer review only

Table 1. Snapshot of the UK Transplant Registry cohort

	01/01/1985-31/12/1994	01/01/1995-31/12/2005	01/01/2006-31/12/2015	Total
**Kidney**	14341	16172	23436	53949
**Pancreas**	76	451	1835	2362
**Heart**	3271	2533	1457	7261
**Lung**	1160	1593	1683	4436
**Liver**	3296	6846	6925	17067
**Pancreas islets**	0	0	120	120
**Abdominal/intestinal**	4	47	164	215
**Total**	22148	27642	35620	85410

For peer review only

For peer review only - http://bmjopen.bmj.com/site/about/guidelines.xhtml

Table 2. Demographics of UK Transplant Registry cohort

		Kidney (n=53949)	Pancreas (n=2362)	Heart (n=7261)	Lung (n=4436)	Liver (n=17067)	Pancreas islets (n=120)	Abdominal / Intestinal (n=215)	Missing or unreported data
First graft		46347	2230	7115	4305	15241	74	201	0
Part of multi-organ transplant		2199	1994	1136	1027	441	0	137	0
Donor	Living	12475	0	0	24[1]	380	0	0	1
	DBD[2]	35310	2039	7246	4199	15551	110	215	
	DCD[3]	6164	323	14	224	1136	10	0	
Female recipient		20510	1006	2008	2062	7517	87	93	48
Ethnicity of recipient	White	33556	2060	3963	3237	12073	117	181	19029
	Asian	4641	115	269	75	1476	1	15	
	Black	2369	69	77	26	438	2	5	
	Chinese	300	5	11	2	107	0	0	
	Mixed	122	5	15	1	23	0	1	
	Other	545	19	39	9	408	0	3	
Recipien	0-9	1329	37	611	86	2028	0	98	22

For peer review only - http://bmjopen.bmj.com/site/about/guidelines.xhtml

For peer review only

**t age (in years)**	**10-19**	2860	20	733	392	946	0	15	
	**20-29**	6546	168	746	734	1176	0	21	
	**30-39**	9719	781	813	607	1745	10	23	
	**40-49**	12285	912	1556	864	3390	42	26	
	**50-59**	11972	397	2265	1288	4812	48	20	
	**60-69**	7733	47	531	464	2861	17	11	
	**70-79**	1464	0	3	1	106	3	1	
	**80+**	25	0	0	0	0	0	0	

[1]Partial solitary lung, [2]Donation after brain death, [3]Donation after cardiac death

For peer review only

Table 3. Adequate power for EpCOT study events

Site of cancer development	Estimated expected number of cancers	Minimal SIR that can be detected
All cancers excluding non-melanoma	1650	1.1
Skin: non-melanoma	294	1.1
Lung and Bronchus	252	1.1
Breast	243	1.1
Colorectal	200	1.2
Prostate	197	1.2
Bladder	64	1.3
Non-Hodgkin's lymphoma	56	1.3
Skin – malignant	53	1.4
Stomach	49	1.4
Oesophagus	44	1.4
Kidney	39	1.5
Pancreas	38	1.5

For peer review only - http://bmjopen.bmj.com/site/about/guidelines.xhtml

Leukaemia	37	1.5
Ovary	35	1.5
Uterus	31	1.5
Multiple myeloma	21	1.7
Cervix	18	1.8
Oral cavity	16	1.8
Liver	16	1.8
Hodgkin's lymphoma	10	1.9
Thyroid	9	2.0
Anus	5	3.0
Lip	2	4.0
Kaposi sarcoma	1.4	5.5

For peer review only

Figure legend

Figure 1. Flow of study data for the EpCOT project between data providers, NHS Digital and the University of Birmingham as Data Processor.

For peer review only - http://bmjopen.bmj.com/site/about/guidelines.xhtml

Figure 1. Flow diagram of data linkage process

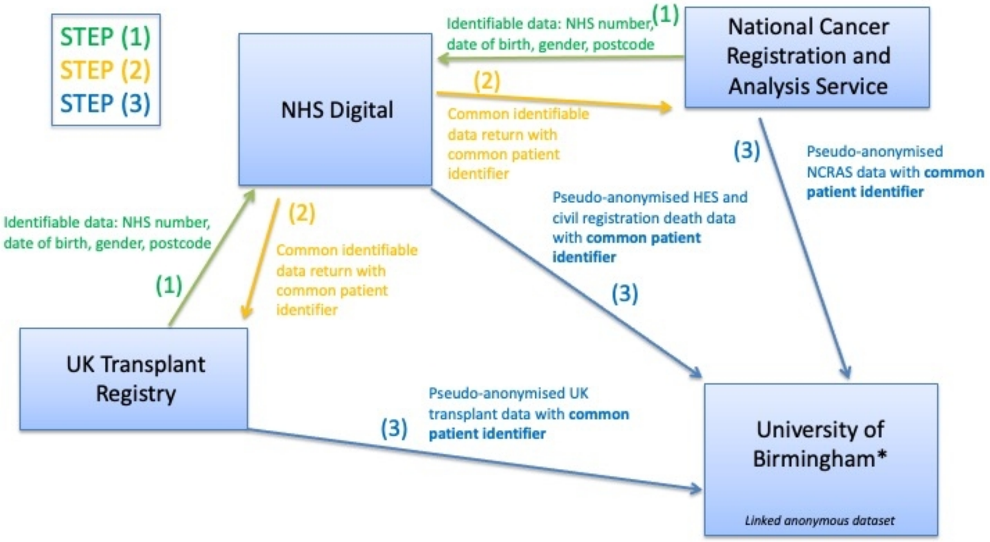

*University of Birmingham is the Data Processor. University Hospitals Birmingham NHS Foundation Trust is the Data Controller but will not receive (or access) any data at any time point.

254x190mm (72 x 72 DPI)

	**Activity/Organ(s):**
	**Geographical area:**
	**Mark data items required with an X**
**X**	**Variable**
	ARECIP_ID
	NHS_GRP
	RIMD
	RIMD_RANK
X	rhosp
X	RSEX
X	RAGE
	RAGE_GRP
	RAGE_GRP2
	PAED_AT_REG
X	RBG
	RRHESUS
x	RWEIGHT
x	RHEIGHT
X	RBMI
X	RETHNIC
X	PRD
	FPRD
	DIAB
X	WAIT_TIME
X	RCMV
X	RHCV
X	RHBV
	RHIV
X	DIAL_AT_REG
X	DIAL_AT_TX
X	DIAL_AT_TX_TYPE
	ATX_ID
X	TX_YR
	F_YR
X	TX_TYPE
	ORGAN
X	MULTI_TX
X	GRAFT_NO
X	HSP
X	CRF_TX
	rethosp
	LOCAL
	ADONOR_ID
	dd_yr
	DIMD
	DIMD_RANK
X	DTYPE
	DGRP
	DCD_CATEGORY
	RLTNSHIP
	dutilised
X	DSEX
X	DAGE

For peer review only

	DAGEMTHS
	DAGE_GRP
	DAGE_GRP2
x	DBG
X	DHEIGHT
X	DWEIGHT
X	DBMI
X	DETHNIC
x	DCOD
X	CIT_MINS
	CIT_HRS
	DURINE_OUTPUT_24HRS
	DURINE_OUTPUT_HR
	DURINE_PERIOD
	DURINE_PERIOD_OUTPUT
	dhosp
X	DRET_CREAT
X	DCMV
	DHBSAG
	DHBCAB
	DHCV
	DHIV
X	DEBV
	DHTLV
	DTOXO
	DSYPHILIS
	DHBCAG
	DPAST_ALCOHOL_ABUSE
	DPAST_CARDIO_DISEASE
	DPAST_DIABETES
	DPAST_FAMILY_DIABETES
	DPAST_FAMILY_DIABETES_TYPE
	DPAST_DRUG_ABUSE
	DPAST_HYPERTENSION
	DPAST_LIVER_DISEASE
	DPAST_OTHER
x	DPAST_SMOKER
x	DPAST_SMOKER_AMOUNT
x	DPAST_TUMOUR
	DPAST_UTI
	DPAST_ALLERGY
	PERSATE
	PERFQUAL
	MACHPER
	D_A_HOM
	D_B_HOM
	D_DR_HOM
	R_A_HOM
	R_B_HOM
	R_DR_HOM
	AMM_1998
	BMM_1998
	DRMM_1998

For peer review only

	AMM
	BMM
	DRMM
	mgrade
x	HLA_GRP
	matchpts
x	MATCHBTY
x	AITX_TYPE
	AITX_GRP
	AITX_FLAG
x	DGF
x	A_0
x	ALI_0
x	C_0
x	M_0
	O_0
	OKI_0
x	P_0
x	T_0
x	serum3
x	A_3
x	C_3
x	M_3
x	O_3
x	P_3
x	T_3
x	REJECTION_COUNT3
	malignancy3
	RECURRENT_DISEASE3
x	serum12
x	A_12
x	C_12
x	M_12
x	O_12
x	P_12
x	T_12
x	REJECTION_COUNT12
	malignancy12
	RECURRENT_DISEASE12
x	serum60
	A_60
	C_60
	M_60
	O_60
	P_60
	T_60
	REJECTION_COUNT60
	malignancy60
	RECURRENT_DISEASE60
x	GRAFT_OUTCOME

er review only

For peer review only - http://bmjopen.bmj.com/site/about/guidelines.xhtml

x	COF
	FCOF
	RCOD
	RCOD2
	FRCOD
	FRCOD2
	VTL_STAT
x	LAST_FUP
x	LOST_FUP
	tsurv
	tcens
x	gsurv
x	gcens
	PSURV
	PCENS

For peer review only

Kidney
United Kingdom
**Description**
Anonymised ODT recipient identification number
NHS group status
Recipient 2010 IMD deprivation score based on postcode (England)
Recipient 2010 IMD deprivation ranking order, 1 being the most deprived (England)
Anonymised unit that received and transplanted the organ
Recipient sex
Recipient age
Recipient age group, adult or paediatric
Recipient age group2, ten year increments
Does the patient have paediatric priority from registration
Recipient blood group
Recipient blood rhesus allele
Recipient weight at registration (kg)
Recipient height at registration (cm)
Recipients BMI
Recipient ethnicity
Primary renal disease
Primary renal disease in text
Was the recipient diabetic
Recipient waiting time (days)
Recipient CMV test result
Recipient HCV status
Recipient HBV status
Recipient HIV status
Dialysis status at transplant
On dialysis prior to transplant
Status of dialysis prior to transplant type
Anonymised transplant identification number
Year of transplant
Financial year of transplant
Type of transplant
Organ transplanted
Multi-organ transplant indicator
Number of kidney transplants
Flag indicating whether recipient was highly sensitised or not at time
Calculated reaction frequency at transplant
Anonymised unit that retrieved the organ
Kidney used by local centre
Anonymised donor identification number
Year of donation
Donor 2010 IMD deprivation score based on postcode (England)
Donor 2010 IMD deprivation ranking order, 1 being the most deprived (England)
Type of donor
Donor type group
Patient code for DCD donors
Relationship of donor to recipient
Donor utilised
Donor sex
Donor age

Month part of donor age (if under 3)
Donor age group, adult or paediatric
Donor age group, ten year increments
Donor blood group
Donor height (cm)
Donor weight (kg)
Donors BMI
Donor ethnicity
Donor cause of death
Cold ischaemic time (minutes)
Cold ischaemic time (hours)
Donor urine output within the last 24 hours (ml)
Donor urine output within the last hour (ml)
Donor, period or urine output (hr)
Donor urine output (ml)
Anonymised donation centre
Donor creatinine at retrieval
Donor CMV test result
Donor HBS AG test result
Donor HBCAB test result
Donor HCV test result
Donor HIV test result
Donor EBV test result
Donor HTLV test result
Donor Toxo test result
Donor syphilis test result
Donor HBCAG test result
Donor history of alcohol abuse
Donor cardio disease
Donor diabetes
Donors family history of diabetes
Donors family history type of diabete
Donor history of drug abuse
Donor hypertension
Donor liver disease
Donor past other
Donor smoker
Donor smoker amount
Donor tumour
Donor UTI
Donor allergy
Perfusate used
Perfusion quality
Machine perfusion
Donor homozygous at A locus
Donor homozygous at B locus
Donor homozygous at DR locus
Recipient homozygous at A locus
Recipient homozygous at B locus
Recipient homozygous at DR locus
Number of mismatches at A locus 1998
Number of mismatches at B locus 1998
Number of mismatches at DR locus 1998

Number of mismatches at A locus
Number of mismatches at B locus
Number of mismatches at DR locus
Matchgrade
HLA mismatch groups
Points score based on current matchability points band
Matchability
Type of antibody incompatible transplant
Group of antibody incompatible transplant
Source of antibody incompatiable transplant
Function of kidney post transplant
Azathioprine immunosuppression at transplant
ALG/ATG antibody at transplant
Ciclosporin immunosuppression at transplant
Mycophenolate immunosuppression at transplant
Other monoclonal antibody at transplant
OKT3 antibody at transplant
Predniscolone / predniscone immunosuppression at transplant
Tacrolimus immunosuppression at transplant
Serum creatinine at 3 months
Azathioprine immunosuppression at three months follow up
Ciclosporin immunosuppression at three months follow up
Mycophenolate immunosuppression at three months follow up
Other immunosuppression at three months follow up
Predniscolone / predniscone immunosuppression at three months follow up
Tacrolimus immunosuppression at three months follow up
Number of treated rejection episodes at 3 month follow up
Has the recipient developed a malignancy in the first 3 months post-transplant
Has the recipient developed evidence of a primary or recurrent renal disease (excluding infection, large vessel disease, outflow obstruction and chronic allograft nephropathy) in the graft during the frist 3 months post
Serum creatinine at 1 year
Azathioprine immunosuppression at one year follow up
Ciclosporin immunosuppression at one year follow up
Mycophenolate immunosuppression at one year follow up
Other immunosuppression at one year follow up
Predniscolone / predniscone immunosuppression at one year follow up
Tacrolimus immunosuppression at one year follow up
Number of treated rejection episodes at 1 year follow up
Has the recipient developed a malignancy in the first twelve months post-transplant
Has the recipient developed evidence of a primary or recurrent renal disease (excluding infection, large vessel disease, outflow obstruction and chronic allograft nephropathy) in the graft during the frist year post transplant.
Serum creatinine at 5 years
Azathioprine immunosuppression at five years follow up
Ciclosporin immunosuppression at five years follow up
Mycophenolate immunosuppression at five years follow up
Other immunosuppression at five years follow up
Predniscolone / predniscone immunosuppression at five years follow up
Tacrolimus immunosuppression at five years follow up
Number of treated rejection episodes within the last 12 months at 5 year follow up
Has the recipient developed a malignancy in the last 12 months at 5 years post-transplant
Has the recipient developed evidence of a primary or recurrent renal disease (excluding infection, large vessel disease, outflow obstruction and chronic allograft nephropathy) in the graft in the last 12 months at 5 years post
Last known outcome of graft

Cause of failure
Cause of failure in text
Primary recipient cause of death
Secondary recipient cause of death
Primary recipient cause of death in text
Secondary recipient cause of death in text
Recipient vital status
Last reported follow up period
Lost to follow up
Transplant survival time (days)
Transplant censoring indicator
Graft survival time (days)
Graft censoring indicator
Patient survival time (days)
Patient censoring indicator

For peer review only

Reference
1=Group 1, 2=Group 2, 8=Not reported, 9=Unknown
1=No, 2=Yes, 8=Not reported, 9=Unknown
1=Positive, 2=Negative, 8=Missing, 9=Unknown, 10=Not tested
see reference renal disease
0=No, 1=Yes
1=Negative, 2=Positive, 6=results awaited, 7=Not tested, 8=Not reported, 9=Unknown
1=Negative, 2=Positive, 6=results awaited, 7=Not tested, 8=Not reported, 9=Unknown
1=Negative, 2=Positive, 6=results awaited, 7=Not tested, 8=Not reported, 9=Unknown
1=Negative, 2=Positive, 6=results awaited, 7=Not tested, 8=Not reported, 9=Unknown
1=Haemodialysis, 2=Peritoneal dialysis, 3=Not on dialysis, 8=Not reported, 9=Unknown
1=No, 2=Yes, 8=Not reported, 9=Unknown
1=Haemodialysis, 2=Peritoneal dialysis, 3=Not on dialysis, 8=Not reported, 9=Unknown
10=Kidney, 13=En-bloc kidney, 14=Double kidney, 110=Liver & kidney,115=Liver, kidney & pancreas, 130=Kidn
10=Unspecified kidney, 11=Left kidney, 12=Right kidney
1=Kidney only, 2=Multiple organ transplant
1=No, 2=Yes, 8=Not reported, 9=Unknown
0=Imported, 1=Used by local centre
1=DBD, 2=DCD, 5=Living related, 6=Living, unrelated, 7=Domino 13=Living unrelated - Altruistic
11=After withdrawl of treatment in a patient not certified dead by brain stem tests, 12=After withdrawl of treatmer
see reference relationship
1=No, 2=Yes, 8=Not reported, 9=Unknown
1=Negative, 2=Positive, 6=results awaited, 7=Not tested, 8=Not reported, 9=Unknown

see reference donor COD

1=Negative, 2=Positive, 6=results awaited, 7=Not tested, 8=Not reported, 9=Unknown
1=Negative, 2=Positive, 6=results awaited, 7=Not tested, 8=Not reported, 9=Unknown
1=Negative, 2=Positive, 6=results awaited, 7=Not tested, 8=Not reported, 9=Unknown
1=Negative, 2=Positive, 6=results awaited, 7=Not tested, 8=Not reported, 9=Unknown
1=Negative, 2=Positive, 6=results awaited, 7=Not tested, 8=Not reported, 9=Unknown
1=Negative, 2=Positive, 6=results awaited, 7=Not tested, 8=Not reported, 9=Unknown
1=Negative, 2=Positive, 6=results awaited, 7=Not tested, 8=Not reported, 9=Unknown
1=Negative, 2=Positive, 6=results awaited, 7=Not tested, 8=Not reported, 9=Unknown
1=Negative, 2=Positive, 6=results awaited, 7=Not tested, 8=Not reported, 9=Unknown
1=Negative, 2=Positive, 6=results awaited, 7=Not tested, 8=Not reported, 9=Unknown
1=No, 2=Yes, 8=Not reported, 9=Unknown
1=No, 2=Yes, 8=Not reported, 9=Unknown
1=No, 2=Yes, 8=Not reported, 9=Unknown
1=No, 2=Yes, 8=Not reported, 9=Unknown
3=Type 1, 4=Type 2, 5=Type 1 & 2, 8=Not reported, 9=Unknown
1=No, 2=Yes, 8=Not reported, 9=Unknown
1=No, 2=Yes, 8=Not reported, 9=Unknown
1=No, 2=Yes, 8=Not reported, 9=Unknown
1=No, 2=Yes, 8=Not reported, 9=Unknown
1=No, 2=Yes, 8=Not reported, 9=Unknown

1=No, 2=Yes, 8=Not reported, 9=Unknown
1=No, 2=Yes, 8=Not reported, 9=Unknown
1=No, 2=Yes, 8=Not reported, 9=Unknown
0=No, 1=Yes
see reference perfusate used
1=No, 2=Yes Hypothermic, 3=Yes Normothermic, 8=Not reported, 9=Unknown
0=Heterozygous, 1=Homozygous, 9=Unknown
0=Heterozygous, 1=Homozygous, 9=Unknown
0=Heterozygous, 1=Homozygous, 9=Unknown
0=Heterozygous, 1=Homozygous, 9=Unknown
0=Heterozygous, 1=Homozygous, 9=Unknown
0=Heterozygous, 1=Homozygous, 9=Unknown
0=Zero mismatches, 1=One mismatch, 2=Two mismatches, 7=Not tested, 9=Unknown
0=Zero mismatches, 1=One mismatch, 2=Two mismatches, 7=Not tested, 9=Unknown
0=Zero mismatches, 1=One mismatch, 2=Two mismatches, 7=Not tested, 9=Unknown

0=Zero mismatches, 1=One mismatch, 2=Two mismatches, 7=Not tested, 9=Unknown
0=Zero mismatches, 1=One mismatch, 2=Two mismatches, 7=Not tested, 9=Unknown
0=Zero mismatches, 1=One mismatch, 2=Two mismatches, 7=Not tested, 9=Unknown
000= zero zero zero mismatches, Fave=Favourable mismatch (100,010,110), Non-fave=Non-favourable mismat
see reference Table A: HLA mismatch groups

1=HLAi, 2=ABOi, 3=Both HLAi/ABOi, 8=Not reported
HLA=HLA and HLA/ABO
FORM- Antibody incompatible form received, KTRF=Identified on kidney transplant form, ABO=Calculated from
1=Immediate, 2=Delayed, 3=Primary non-function, 8=Not reported, 9=Unknown
1=No, 2=Yes, 8=Not reported, 9=Unknown
1=No, 2=Yes, 8=Not reported, 9=Unknown
1=No, 2=Yes, 8=Not reported, 9=Unknown
1=No, 2=Yes, 8=Not reported, 9=Unknown
1=No, 2=Yes, 8=Not reported, 9=Unknown
1=No, 2=Yes, 8=Not reported, 9=Unknown
1=No, 2=Yes, 8=Not reported, 9=Unknown
1=No, 2=Yes, 8=Not reported, 9=Unknown

1=No, 2=Yes, 8=Not reported, 9=Unknown
1=No, 2=Yes, 8=Not reported, 9=Unknown
1=No, 2=Yes, 8=Not reported, 9=Unknown
1=No, 2=Yes, 8=Not reported, 9=Unknown
1=No, 2=Yes, 8=Not reported, 9=Unknown
1=No, 2=Yes, 8=Not reported, 9=Unknown

1=No, 2=Yes, 8=Not reported, 9=Unknown

1=No, 2=Yes, 8=Not reported, 9=Unknown

1=No, 2=Yes, 8=Not reported, 9=Unknown
1=No, 2=Yes, 8=Not reported, 9=Unknown
1=No, 2=Yes, 8=Not reported, 9=Unknown
1=No, 2=Yes, 8=Not reported, 9=Unknown
1=No, 2=Yes, 8=Not reported, 9=Unknown
1=No, 2=Yes, 8=Not reported, 9=Unknown

1=No, 2=Yes, 8=Not reported, 9=Unknown

1=No, 2=Yes, 8=Not reported, 9=Unknown

1=No, 2=Yes, 8=Not reported, 9=Unknown
1=No, 2=Yes, 8=Not reported, 9=Unknown
1=No, 2=Yes, 8=Not reported, 9=Unknown
1=No, 2=Yes, 8=Not reported, 9=Unknown
1=No, 2=Yes, 8=Not reported, 9=Unknown
1=No, 2=Yes, 8=Not reported, 9=Unknown

1=No, 2=Yes, 8=Not reported, 9=Unknown

1=No, 2=Yes, 8=Not reported, 9=Unknown

1=Functioning, 2=Failed, 3=Died with a functioning graft, 4=Died unknown graft function, 9=Unknown

see reference renal causes of failure

see reference recipient COD
see reference recipient COD

1=Alive, 2=Dead, 8=Not reported, 9=Unknown

1=No, 2=Yes, 8=Not reported, 9=Unknown

0=Censored, 1=Event (Graft failure or death)

0=Censored, 1=Event (Graft failure)

0=Censored, 1=Event (Death of recipient)

For peer review only

	**Activity/Organ(s):**
	**Geographical area:**
	**Mark data items required with an X**
**X**	**Variable**
	ARECIP_ID
	NHS_GRP
x	RSEX
X	RAGE
	RAGE_GRP
	RAGE_GRP2
X	RBG
	RRHESUS
X	RWEIGHT
X	RHEIGHT
X	RBMI
X	RETHNIC
X	WAIT_TIME
	PKPD
	FPKPD
X	RCMV
X	RHCV
	SURFACE_AGHBSAG
	HBSAB
	HIV
	ABOmatch
	MM_COMPLETE
	AMM
	BMM
	DRMM
	MGRADE
	HLA_GRP
X	SENSITISATION
X	DIALYSIS
	DIALYSIS_START_DATE
	MYOCARDIAL_INFARCTION
	CVA
	REG_BLIND
	LIMB_AMPUTATION
	DIABETES_CAUSE
	DIAGNOSIS_YR
	INSULIN_DOSE
	SERUM_CREATININE
	PRETX_egfr
	PIPECIGARS
x	CIGARETTES
	NODAY
	YRS_SINCE_STOP
	EXPOSURE
	ADONOR_ID
	DD_YR
X	DTYPE
X	DGRP
X	DSEX

For peer review only

X	DAGE
	DAGE_GRP
	DAGE_GRP2
X	DBG
x	DCOD
	FDCOD
X	DHEIGHT
X	DWEIGHT
	DGIRTH
X	DBMI
X	DETHNIC
X	DCMV
	DHG
	DGLUCOSE
	DGLUCOSE_ON_ADMISSION
	DRET_CREAT
	DHCV
	DREGION
	DNATION
	DCOUNTRY
	DHBSAG
	DHBCAB
	DHIV
X	DEBV
	DHTLV
	DTOXO
	DSYPHILIS
	DHEV
	DURINE_OUTPUT_24HRS
	DURINE_OUTPUT_HR
	DURINE_PERIOD
	DURINE_PERIOD_OUTPUT
	DINSULIN
	DINSULIN_DOSE
	DCURRENT_GLUCOSE
	DINSULIN_DATE
	DINSULIN_RATE
	DPAST_ALCOHOL_ABUSE
	DALCOHOL_UNITS_AMOUNT
	DPAST_CARDIO_DISEASE
	DPAST_DIABETES
	DPAST_FAMILY_DIABETES
	DPAST_FAMILY_DIABETES_TYPE
	DPAST_DRUG_ABUSE
	DPAST_HYPERTENSION
	DPAST_LIVER_DISEASE
	DPAST_OTHER
x	DPAST_SMOKER
x	DPAST_SMOKER_AMOUNT
x	DPAST_TUMOUR
	DPAST_UTI
	DPAST_ALLERGY
	MACHINE_PRESERVATION_USED

For peer review only

	WIT
	CIT
X	CIT_MINS
	ASYSTOLIC_PERIOD
	DUCT_MANAGEMENT
	QUALITY
	SENSITISATION_AT_TX
	HSP
	ATX_ID
x	TX_YR
	FYR
X	TX_TYPE
x	TX_GRP
X	PTX_NO
	HBA1C
	IFCC_HBA1C
	TMF_INITIAL_GRAFT_FUNCTION
	TMF_REJECTIONS
	TMF_RENAL_DISEASE
	TMF_INSULIN_INDEPENDENT
	TMF_INSULIN_DOSE
	TMF_BLOOD_GLUCOSE
	TMF_HBA1C
	TMF_IFCC_HBA1C
	TMF_CREATININE
x	AF1Y_REJECTIONS
x	AF1Y_RENAL_DISEASE
x	AF1Y_INSULIN_INDEPENDENT
	AF1Y_BLOOD_GLUCOSE
	AF1Y_HBA1C
	AF1Y_IFCC_HBA1C
	AF1Y_CREATININE
	AF2Y_REJECTIONS
	AF2Y_RENAL_DISEASE
	AF2Y_INSULIN_INDEPENDENT
	AF2Y_BLOOD_GLUCOSE
	AF2Y_HBA1C
	AF2Y_IFCC_HBA1C
	AF2Y_CREATININE
	AF5Y_REJECTIONS
	AF5Y_RENAL_DISEASE
	AF5Y_INSULIN_INDEPENDENT
	AF5Y_BLOOD_GLUCOSE
	AF5Y_HBA1C
	AF5Y_IFCC_HBA1C
	AF5Y_CREATININE
X	GRAFT_OUTCOME
X	COF
	FCOF
	COF_NOTE
	RCOD1
	RCOD2
	FRCOD1

review only

	FRCOD2
	GRAFT_FUNCTION
	RCOD_NOTE
	TSURV
	TCENS
X	GSURV
X	GCENS
	PSURV
	PCENS
	KID_DGF
	KID_OUTCOME
	KID_LFUP
	KID_GSURV
	KID_GCENS
	KID_PSURV
	KID_PCENS

For peer review only

Pancreas
**United Kingdom**
**Description**
Anonymised recipient identification number
NHS group
Recipient sex
Recipient age at transplant
Recipient paediatric or adult
Recipient age in 10 year increments
Recipient blood group
Recipient blood rhesus allele
Weight of recipient (kg)
Height of recipient (cm)
Recipient BMI
Recipient ethnicity
Waiting time for transplant (days)
Best known pancreas/renal disease at registration
Pancreas/renal diease at registration in text
Recipient CMV test result at registration
Recipient HCV test result at registration
Recipient Hepatitis B - Surface Ag(HBsAG)
Recipient if negative Hepatitis B - Surface Ag(HBsAG) then HBsAb
Recipient HIV
Donor vs Recipient blood group
Mismatch recipient
Number of mismatches at A locus
Number of mismatches at B locus
Number of mismatches at dr locus
Matchgrade
HLA mismatch groups
% sensitised against general donor population
Dialysis status of recipient at transplant
Date current dialysis started
Pre-transplant complications- Myocardial infarction
Pre-transplant complications- CVA
Pre-transplant complications- Registered blind
Pre-transplant complications- Limb amputation
Cause of diabetes
Year of diagnosis
Insulin dose prior to transplant (units/24hr)
If not on dialysis, serum creatinine prior to transplant
Pre-transplant Estimated GFR
Does the recipient smoke pipe and cigars at transplant
Does the recipient smoke cigarettes at transplant
If the recipient smokes cigarettes, how many a day at transplant?
If ex smoker how many years since stopped smoking at transplant
Total exposure in pack years at transplant
Anonymised donor identification number
Year of donation
Donor type
Donor type group
Donor sex

Donor age
Donor paediatric or adult
Donor age in 10 year increments
Donor blood group
Donor cause of death
Donor cause of death in text
Donor height (cm)
Donor weight (kg)
Donor Girth (cm)
Donor BMI
Donor ethnicity
Donor CMV test result
Donor haemoglobin at retrieval
Donor glucose at retrieval
Donor glucose on admission
Donor creatinine at retrieval
Donor HCV at retrieval
Region of donor hospital
Nation of donor hospital
Country of donor hospital
Donor HBS AG test result
Donor HBCAB test result
Donor HIV test result
Donor EBV test result
Donor HTLV test result
Donor Toxo test result
Donor syphilis test result
Donor HEV test result
Donor urine output last 24 hours (ml)
Donor urine output last hour (ml)
Donor urine last for (hrs)
Donor urine output in relation to durine_period time
Insulin adminstered to the donor
If yes what was the dose of insulin given to the donor (mg)
Donor blood glucose level mmol/l
If yes the date and time insulin was given to the donor
Donor rate of insulin infusion (ml/hr)
Donor history of alcohol abuse
Donor units of alcohol (available post January 2016)
Donor cardio disease
Donor diabetes
Donors family history of diabetes
Donors family history type of diabete
Donor history of drug abuse
Donor hypertension
Donor liver disease
Donor past other
Donor smoker
Donor smoker amount
Donor tumour
Donor UTI
Donor allergy
Machine preservation used?

Warm ischaemia time (minutes)
Cold ischaemia time (hh:mm)
Cold ischaemia time (minutes)
If donor DCD then duration of asystolic period (mins)
Type of duct management
Quality of cold perfusion
Sensitisation at transplant (%)
Flag indicating whether recipient was highly sensitised at transplant
Anonymised transplant identification number
Year of transplant
Financial year of transplant
Transplant type
Type of pancreas transplant
Number of pancreas transplants
Pre-transplant HbA1c (%) - as recorded prior to 29/1/13
Pre-transplant IFCC-HbA1c (mmol/mol) - as recorded since 29/1/13
Recipient initial graft function at 3 month fup
Number of treated rejection episodes in the first 3 months post transplant
Recurrent primary disease in pancreatic graft within the first 3 months
Has the recipient been insulin independent at all since transplant at 3 month fup
What is the insulin dose for recipient at 3 months
If insulin independent then state blood glucose (mmol/l)
If insulin independent then state HbA1c (%) - as recorded prior to 29/1/2013
If insulin independent then state IFCC-HbA1c (mmol/l) - as recorded since 29/1/2013
Serum creatinine measured between 10 and 14 weeks post-transplant
Number of treated rejection episodes in the last 12 months at 1 year fup
Recurrent primary disease in pancreatic graft within the last 12 months at 1 year fup
Has the recipient been insulin independent within the last 9 months at 1 year fup
If insulin independent then state blood glucose (mmol/l) at 1 year fup
If insulin independent then state HbA1c (%) at 1 year fup - as recorded prior to 29/1/13
If insulin independent then state IFCC-HbA1c (mmol/mol) at 1 year fup - as recorded since 29/1/13
Serum creatinine measured between 11 and 13 months post-transplant
Number of treated rejection episodes in the last 12 months at 2 year fup
Recurrent primary disease in pancreatic graft within the last 12 months at 2 year fup
Has the recipient been insulin independent within the last 12 months at 2 year fup
If insulin independent then state blood glucose (mmol/l) at 2 year fup
If insulin independent then state HbA1c (%) at 2 year fup - as recorded prior to 29/1/13
If insulin independent then state IFCC-HbA1c (mmol/mol) at 2 year fup - as recorded since 29/1/13
Serum creatinine measured at 2 years
Number of treated rejection episodes in the last 12 months at 5 years fup
Recurrent primary disease in pancreatic graft within the last 12 months at 5 years fup
Has the recipient been insulin independent within the last 12 months at 5 years fup
If insulin independent then state blood glucose (mmol/l) at 5 years fup
If insulin independent then state HbA1c (%) at 5 years fup - as recorded since 29/1/13
If insulin independent then state IFCC-HbA1c (mmol/mol) at 5 years fup - as recorded since 29/1/13
Serum creatinine measured at 5 years
Last known outcome of graft
Cause of failure
Cause of failure in text
Additional cause of failure information
Recipient primary cause of death
Recipient secondary cause of death
Recipient primary cause of death in text

Recipient secondary cause of death in text
Pancreas function at last assessment
Recipient cause of death notes
Transplant survival time (days)
Transplant censoring indicator
Graft survival time (days)
Graft censoring indicator
Patient survival time (days) from first graft only
Patient censoring indicator from first graft only
Function of kidney post transplant
Last known outcome of kidney graft
Kidney lost to fup
Kidney graft survival time (days)
Kidney graft censoring indicator
Kidney patient survival time (days)
Kidney patient censoring indicator

For peer review only

Reference

1=Group 1, 2=Group 2, 9=Unknown

1=Positive, 2=Negative, 8=Missing, 9=Unknown, 10=Not tested

see reference pancreas/renal disease

1=Negative, 2=Positive, 6=results awaited, 7=Not tested, 8=Not reported, 9=Unknown
1=Negative, 2=Positive, 6=results awaited, 7=Not tested, 8=Not reported, 9=Unknown
1=Negative, 2=Positive, 6=results awaited, 7=Not tested, 8=Not reported, 9=Unknown
1=Negative, 2=Positive, 6=results awaited, 7=Not tested, 8=Not reported, 9=Unknown
1=Negative, 2=Positive, 6=results awaited, 7=Not tested, 8=Not reported, 9=Unknown
1=Identical, 2=Compatible, 3=Incompatible, 9=Unknown match

0=Zero mismatches, 1=One mismatch, 2=Two mismatches, 7=Not tested, 9=Unknown
0=Zero mismatches, 1=One mismatch, 2=Two mismatches, 7=Not tested, 9=Unknown
0=Zero mismatches, 1=One mismatch, 2=Two mismatches, 7=Not tested, 9=Unknown
000= zero zero zero mismatches, Fave=Favourable mismatch (100,010,110), Non-fave=Non-favourable mismat
see reference Table A: HLA mismatch groups

1=Haemodialysis, 2=Peritoneal dialysis, 3=Not on dialysis, 8=Not reported, 9=Unknown

1=No, 2=Yes, 8=Not reported, 9=Unknown
1=No, 2=Yes, 8=Not reported, 9=Unknown
1=No, 2=Yes, 8=Not reported, 9=Unknown
1= No amputation, 2=Major limb amputation, 3=Digital amputation
1=Type 1, 2=Type 2, 3=Other

1=No, 2=Yes, 3=Ex-smoker, 8=Not reported, 9=Unknown
1=No, 2=Yes, 3=Ex-smoker, 8=Not reported, 9=Unknown

1=DBD, 2=DCD, 5=Living related, 6=Living, unrelated, 7=Domino 13=Living unrelated - Altruistic

see reference donor COD

1=Negative, 2=Positive, 6=results awaited, 7=Not tested, 8=Not reported, 9=Unknown

1=Negative, 2=Positive, 6=results awaited, 7=Not tested, 8=Not reported, 9=Unknown

1=Negative, 2=Positive, 6=results awaited, 7=Not tested, 8=Not reported, 9=Unknown
1=Negative, 2=Positive, 6=results awaited, 7=Not tested, 8=Not reported, 9=Unknown
1=Negative, 2=Positive, 6=results awaited, 7=Not tested, 8=Not reported, 9=Unknown
1=Negative, 2=Positive, 6=results awaited, 7=Not tested, 8=Not reported, 9=Unknown
1=Negative, 2=Positive, 6=results awaited, 7=Not tested, 8=Not reported, 9=Unknown
1=Negative, 2=Positive, 6=results awaited, 7=Not tested, 8=Not reported, 9=Unknown
1=Negative, 2=Positive, 6=results awaited, 7=Not tested, 8=Not reported, 9=Unknown
1=Negative, 2=Positive, 6=results awaited, 7=Not tested, 8=Not reported, 9=Unknown

1=No, 2=Yes, 8=Not reported, 9=Unknown

1=No, 2=Yes, 8=Not reported, 9=Unknown
1=Non-Drinker Alcohol, 2=Drinks Rarely/ Occasionally - <1 u/day, 3=Light Drinker - 1-2 u/day, 4=Moderate Drink
1=No, 2=Yes, 8=Not reported, 9=Unknown
1=No, 2=Yes, 8=Not reported, 9=Unknown
1=No, 2=Yes, 8=Not reported, 9=Unknown
3=Type 1, 4=Type 2, 5=Type 1 & 2, 8=Not reported, 9=Unknown
1=No, 2=Yes, 8=Not reported, 9=Unknown
1=No, 2=Yes, 8=Not reported, 9=Unknown
1=No, 2=Yes, 8=Not reported, 9=Unknown
1=No, 2=Yes, 8=Not reported, 9=Unknown
1=No, 2=Yes, 8=Not reported, 9=Unknown

1=No, 2=Yes, 8=Not reported, 9=Unknown
1=No, 2=Yes, 8=Not reported, 9=Unknown
1=No, 2=Yes, 8=Not reported, 9=Unknown
1=No, 2=Yes - warm, 3=Yes - cold

1=Enteric side to side, 2=Enteric - roux, 3=Bladder drainage
1=Good, 2=Fair, 3=Poor, 4=Patchy, 8=Not reported, 9=Unknown

1=No, 2=Yes, 8=Not reported, 9=Unknown

50=Pancreas only, 51=Pancreas islets, 115=Pancreas, Liver & kidney, 130=Pancreas/Kidney, 140=Pancreas &
PAK=Pancreas after kidney, PTA=Pancreas transplant alone, SPK=Simultaneous pancreas/kidney, IAK= Islets a

1=Immediate function, 2=Partial function (Insulin required), 3=Primary non-function

1=No, 2=Yes, 8=Not reported, 9=Unknown
1=No, 2=Yes, 8=Not reported, 9=Unknown

1=No, 2=Yes, 8=Not reported, 9=Unknown
1=No, 2=Yes, 8=Not reported, 9=Unknown

1=No, 2=Yes, 8=Not reported, 9=Unknown
1=No, 2=Yes, 8=Not reported, 9=Unknown

1=No, 2=Yes, 8=Not reported, 9=Unknown
1=No, 2=Yes, 8=Not reported, 12=Unknown

1=Functioning, 2=Failed, 3=Died with a functioning graft, 4=Died unknown graft function, 9=Unknown
see reference cause of failure

see reference recipient cause of death
see reference recipient cause of death

1=Insulin independent, 2=Partial function, 3=No function

0=Censored, 1=Event (Graft failure or death)

0=Censored, 1=Event (Graft failure)

0=Censored, 1=Event (Death of recipient)
1=Immediate, 2=Delayed, 3=Primary non-function, 8=Not reported, 9=Unknown
1=Functioning, 2=Failed, 3=Died with a functioning graft, 4=Died unknown graft function, 9=Unknown
1=No, 2=Yes, 8=Not reported, 9=Unknown

0=Censored, 1=Event (Graft failure)

0=Censored, 1=Event (Death of recipient)

For peer review only

	**Activity/Organ(s):**
	**Geographical area:**
	**Mark data items required with an X**
**X**	**Variable**
	ARECIP_ID
	AREGISTRATION_ID
	RPC2
	RIMD
	RIMD_RANK
X	RSEX
X	RAGE
	RAGE_GRP
X	rbg
X	RWEIGHT
X	RHEIGHT
X	RETHNIC
X	RCSPLD1
X	RCSPLD2
	RCSPLD3
X	RFAIL_GRADE
	RLIFE
	RIN_PAT
	RVENT
	RREN_SUP
	RASCITES
	RDIURETIC
	RENCEPH
	RAB_SURGERY
	RICP_MON
	ROES_VARICES
	ROES_SHUNT
	RPYREXIAL
	RSEPSIS
	RSEPSIS_SITE
	RHB
	RWBC
	RPLATELETS
	RCREAT
	RCREAT_CORR
X	RALBUMIN
	RUREA
	RINR
	RBILIRUBIN
	RSODIUM
X	UKELD
X	MELD
	RPOTASSIUM
	RPH
	ROXYGEN
X	RCMV
X	RHCV
	RHBV_DNA
	RHBS_AG

review only

	RHBE_AG
	RHCV_RNA
	RANTI_HBCLGM
	RANTI_HBS
	RANTI_HBE
	RANTI_HCV
	RANTI_DELTA
	RANTI_CMV
	RANTI_HSV
	RANTI_HIV
	ATX_ID
x	TX_YR
	REREG
	rereg_yr
	RE_TXD
	nxttx_yr
X	TX_TYPE
X	MULTI_TX
	TX_METHOD
	ORGAN
	ORG_TXD
	sm_bowel
	URGENT
	su_flag
X	LIVER
	ALLTX_NO
X	GRAFT_NO
	TXNATION
	TXCOUNTRY
	ADONOR_ID
	dd_yr
X	DTYPE
	DGRP
X	DSEX
X	DAGE
	DAGE_GRP
	dbg
X	DBMI
X	DCOD
	DNATION
	DCOUNTRY
X	DCMV
	KIDDONOR
	PANDONOR
	SMBDONOR
	HRTDONOR
	LNGDONOR
	MULTIDON
	PRES_FLUID
	ORG_APP
	STEATOSIS
	CAPSULAR DAMAGE
	HEP_ARTERY

review only

CIT
VV_TIME
REP_TIME
BA
AA
RANTI_FIB
IO_D
IO_BLOOD
IO_PLASMA
IO_PLATELETS
IO_CRYO
PO_BLOOD
PO_PLASMA
PO_PLATELETS
PO_CRYO
COMMON_BILE_DUCT
COMMON_HEP_ARTERY
HEPATIC_ARTERY
ILIAC_ARTERY
ILIAC_VEIN
LEFT_AND_RIGHT_HEPATIC
LEFT_HEPATIC_GASTRIC
LYMPH_NODE
MAIN_PORTAL_VEIN
NORMAL_ANATOMY
PATCH
RIGHT_HEPATIC_SMA
SPLEEN
VENA_CAVA
TMF_LIFESTYLE
TMF_REJECTION_EPISODES
TMF_DAYS_VENTILATED
TMF_DAYS_IN_ITU
TMF_RENAL_STATUS
TMF_HEPATIC_ART_THROMBOSIS
TMF_PORTAL_VEIN_THROMBOSIS
TMF_IVC
TMF_HAEMORRHAGE
TMF_BILIARY_TRACT_LEAK
TMF_BILIARY_TRACT_STRICTURE
TMF_BILIARY_STRICTURE_TREAT
TMF_CMV_INFECTION
TMF_FUNGAL_INFECTION
TMF_SPUTUM_SEPSIS
TMF_BLOOD_SEPSIS
TMF_URINE_SEPSIS
TMF_ASCITIES_SEPSIS
TMF_WOUND_SEPSIS
TMF_OTHER_SEPSIS
x TMF_CYCLOSPORIN
x TMF_AZATHIOPRINE
x TMF_MYCOPHENOLATE
x TMF_PREDNISOLONE

review only

x	TMF_FK506
x	TMF_IMMUNO_DRUG_OTHER
x	TMF_IMMUNO_DRUG_OTHER_TEXT
	AF1Y_LIFESTYLE
	AF1Y_DAYS_INPATIENT
	AF1Y_READMISSIONS
	AF1Y_PROTOCOL
	AF1Y_ACUTE_REJECTION
	AF1Y_CHRONIC_REJECTION
	AF1Y_BILIARY_COMP
	AF1Y_VASCULAR_COMP
	AF1Y_DISEASE_RECUR
	AF1Y_INFECTION
	AF1Y_READMIT_OTHER
	AF1Y_BILIRUBIN
	AF1Y_ALK_PHOSPHATE
	AF1Y_ALT_SGOT
	AF1Y_AST_SGPT
	AF1Y_BLOOD_UREA
	AF1Y_DYSFUNCTION
	AF1Y_TUMOUR
x	AF1Y_CYCLOSPORIN
x	AF1Y_FK506
x	AF1Y_AZATHIOPRINE
x	AF1Y_MYCOPHENOLATE
x	AF1Y_PREDNISOLONE
x	AF1Y_IMMUNO_DRUG_OTHER
x	AF1Y_IMMUNO_DRUG_OTHER_TEXT
	AF5Y_LIFESTYLE
	AF5Y_DAYS_INPATIENT
	AF5Y_READMISSIONS
	AF5Y_PROTOCOL
	AF5Y_ACUTE_REJECTION
	AF5Y_CHRONIC_REJECTION
	AF5Y_BILIARY_COMP
	AF5Y_VASCULAR_COMP
	AF5Y_DISEASE_RECUR
	AF5Y_INFECTION
	AF5Y_READMIT_OTHER
	AF5Y_BILIRUBIN
	AF5Y_ALK_PHOSPHATE
	AF5Y_ALT_SGOT
	AF5Y_AST_SGPT
	AF5Y_BLOOD_UREA
	AF5Y_DYSFUNCTION
	AF5Y_TUMOUR
	AF5Y_CYCLOSPORIN
	AF5Y_FK506
	AF5Y_AZATHIOPRINE
	AF5Y_MYCOPHENOLATE
	AF5Y_PREDNISOLONE
	AF5Y_IMMUNO_DRUG_OTHER
	AF5Y_IMMUNO_DRUG_OTHER_TEXT

For peer review only

X	COF
X	LOST_FUP
	laststat
	last_graft_no
	GSTATUS
	RCOD
	RCOD2
	tsurv
	tcens
X	gsurv
X	gcens
	PSURV
	PCENS

For peer review only

For peer review only

Liver
United Kingdom
Description

Anonymised recipient identification number
Anonymised registration identification number
Letters of the first part of the recipients postcode
Recipient 2010 IMD deprivation score based on postcode (England)
Recipient 2010 IMD deprivation ranking order, 1 being the most deprived (England)
Recipient sex
Recipient age
Recipient age group (Adult or Paediatric)
Recipient blood group
Recipient weight at registration (kg)
Recipient height at registration (cm)
Recipient ethnicity
Primary indication for liver transplant
Secondary indication for liver transplant
Terriary indication for liver transplant
Liver failure grading
Lifestyle activity score prior to transplant
Inpatient prior to tranplant
Ventilated prior to transplant
Renal support prior to transplant
Ascites prior to transplant
Diuretic therapy prior to transplant
Encephalopathy grade prior to transplant
Previous abdominal surgery prior to transplant
ICP monitor prior to transplant
Oesophageal varices prior to transplant
Oesophageal varices prior to transplant indicate shunt
Pyrexial prior to transplant
Sepsis confirmed prior to transplant
If sepesis prior to transplant, where?
Hb prior to transplant (gm/dl)
White blood cells prior to transplant (x10^9/l)
Platelets prior to transplant (x10^9/l)
Recipient creatinine prior to transplant (μmol/l)
Recipient creatinine (μmol/l) prior to transplant after applying the Royal Free correction factor
Albumin prior to transplant (g/l)
Urea prior to transplant (mmol/l)
INR prior to transplant
Bilirubin prior to transplant (μmol/l)
Sodium prior to transplant (mmol/l)
Recipient UK End-stage Liver Disease score prior to transplant
Recipient Model for End-stage Liver Disease score prior to transplant
Potassium prior to transplant (mmol/l)
pH prior to transplant
Oxygen prior to transplant (Kpa)
Recipient CMV test result at registration
Most recent recipient HCV test result
Recipient HBV DNA test result at transplant
Recipient HBS AG test result at transplant

Recipient HBE AG test result at transplant
Recipient HCV RNA test result at transplant
Recipient Anti HBCLGM test result at transplant
Recipient Anti HBS test result at transplant
Recipient Anti HBE test result at transplant
Recipient Anti HCV test result at transplant
Recipient Anti DELTA test result at transplant
Recipient Anti CMV test result at transplant
Recipient Anti HSV test result at transplant
Recipient Anti HIV test result at transplant
Anonymised transplant identification number
Year of transplant
Re-registered after transplant indicator
Year of re-registration
Re-transplanted indicator
Year of next transplant
Type of transplant
Multi-organ transplant indicator
Transplant method
Lobe/segment of liver transplanted
Organ transplanted
Liver and small bowel combined transplant indicator
Urgency status at registration
Super-urgent indicator
Liver completeness of liver transplanted
Recipient transplant number (counts all solid organ transplants)
Number of liver transplants (counts liver transplants only)
Nation of transplant centre
Country of transplant centre
Anonymised donor identification number
Year of donation
Type of donor
Donor type group
Donor sex
Donor age
Donor age group (Adult or Paediatric)
Donor blood group
Donors BMI
Donor cause of death
Donor nation
Donor country
Donor CMV test result
Kidney retrieved
Pancreas retrieved
Small bowel retrieved
Heart retrieved
Lung retrieved
Donated more than one type of solid organ
Preservation fluid
Organ appearance
Steatosis
Capsular damage
Hepatic artery

For peer review only - http://bmjopen.bmj.com/site/about/guidelines.xhtml

Cold ischaemic time
Veno-venous bypass time
Operative repurfusion time
Biliary anastomosis
Hepatic artery anastomosis
Anti-fibrinolytic therapy used?
Intraoperative death?
Number of intraoperative units of blood
Number of intraoperative units of plasma
Number of intraoperative units of platelets
Number of intraoperative units of cryoprecipitate
Number of post-operative units of blood within 48 hours
Number of post-operative units of plasma within 48 hours
Number of post-operative units of platelets within 48 hours
Number of post-operative units of cryoprecipitate within 48 hours
Common bile duct
Common hepatic artery
Number of hepatic arteries
Iliac Artery
Iliac Vein
Left hepatic from left gastric and right hepatic from SMA
Left hepatic from left gastric
Lymph node
Main portal vein
Does the retrieved liver look normal
Aortic Patch
Right hepatic from SMA
Spleen
Vena Cava
Lifestyle activity score at 3 month post transplant
Number of treated rejection episodes since transplant at 3 month form
Number of days ventilated post-transplant
Number of days in ITU post-transplant
Renal status post-transplant
Complications hepatic artery thrombosis
Complications portal vein thrombosis
Complications IVC/hepatic vein occlusion
Complications haemorrhage requiring reoperation
Complications biliary tract leaks
Complications biliary tract stricture
Complications biliary tract stricture treatment
Post-operative CMV infection
Post-operative fungal infection
Post-operative sputum sepsis
Post-operative blood sepsis
Post-operative urine sepsis
Post-operative ascities sepsis
Post-operative wound sepsis
Post-operative other sepsis
Immunosuppression at 3 months: Cyclosporin A
Immunosuppression at 3 months: Azathioprine
Immunosuppression at 3 months: Mycophenolate mofetil
Immunosuppression at 3 months: Prednisolone

Immunosuppression at 3 months: FK 506
Immunosuppression at 3 months: Other
Immunosuppression at 3 months: Other text
Lifestyle activity score at 1 year post transplant
Total number of days as an inpatient during the last year (excluding transplant admission)
Number of readmissions over the last year
Number of readmissions if protocol was the primary reason for readmittance
Number of readmissions if acute rejection was the primary reason for readmittance
Number of readmissions if chronic rejection was the primary reason for readmittance
Number of readmissions if biliary complications was the primary reason for readmittance
Number of readmissions if vascular complications was the primary reason for readmittance
Number of readmissions if disease recurrence was the primary reason for readmittance
Number of readmissions if infection was the primary reason for readmittance
Number of readmissions if other was the primary reason for readmittance
Bilirubin at 1 year (μmol/l)
Alk phosphatase at 1 year (IU/L)
ALT/SGOT at 1 year (IU/L)
AST/SGPT at 1 year (IU/L)
Blood urea at 1 year (mmol/l)
Is renal dysfunction present at 1 year
Tumour been detected at 1 year
Immunosuppression at 1 year: Cyclosporin A
Immunosuppression at 1 year: FK 506
Immunosuppression at 1 year: Azathioprine
Immunosuppression at 1 year: Mycophenolate mofetil
Immunosuppression at 1 year: Prednisolone
Immunosuppression at 1 year: Other
Immunosuppression at 1 year: Other text
Lifestyle activity score at 5 years post transplant
Total number of days as an inpatient during the last year at 5 years
Number of readmissions over the last year at 5 years
Total number of readmissions if protocol was the primary reason for readmittance over the last year at 5 years
Total number of readmissions if acute rejection was the primary reason for readmittance over the last year at 5 y
Total number of readmissions if chronic rejection was the primary reason for readmittance over the last year at 5
Total number of readmissions if biliary complications was the primary reason for readmittance over the last year
Total number of readmissions if vascular complications was the primary reason for readmittance over the last ye
Total number of readmissions if disease recurrence was the primary reason for readmittance over the last year a
Total number of readmissions if infection was the primary reason for readmittance over the last year at 5 years
Total number of readmissions if other was the primary reason for readmittance over the last year at 5 years
Bilirubin at 5 years (μmol/l)
Alk phosphatase at 5 years (IU/L)
ALT/SGOT at 5 years (IU/L)
AST/SGPT at 5 years (IU/L)
Blood urea at 5 years (mmol/l)
Is renal dysfunction present at 5 years
Tumour been detected at 5 years
Immunosuppression at 5 years: Cyclosporin A
Immunosuppression at 5 years: FK 506
Immunosuppression at 5 years: Azathioprine
Immunosuppression at 5 years: Mycophenolate mofetil
Immunosuppression at 5 years: Prednisolone
Immunosuppression at 5 years: Other
Immunosuppression at 5 years: Other text

Cause of failure
Lost to follow up
Graft status of latest graft
Graft number of latest graft
Graft status
Primary recipient cause of death
Secondary recipient cause of death
Transplant survival time (days)
Transplant censoring indicator
Graft survival time (days)
Graft censoring indicator
Patient survival time (days)
Patient censoring indicator

For peer review only

Reference
see reference liver disease
see reference liver disease
see reference liver disease
1=Hyperacute, 2=Acute, 3=Subacute, 4=Not acute
1=Able to carry out normal activity without restriction, 2=Only restricted in physically strenuous activity, 3=Can m
1=No, 2=Yes, 8=Not reported, 9=Unknown
1=No, 2=Yes, 8=Not reported, 9=Unknown
1=Haemodialysis, 2=Filtration, 3=Not required
1=No, 2=Yes, 8=Not reported, 9=Unknown
1=No, 2=Yes, 8=Not reported, 9=Unknown
0=Not present, 1=Compromised. Altered mood/behaviour. Psychometric defects. 2=Drowsy. Inappropriate beha
1=No, 2=Yes, 8=Not reported, 9=Unknown
1=Normal pressure, 2=Raised pressure, 3=Not used
1=No previous variceal bleed, 2=Previous variceal bleed, 3=Not present
1=Portosystemic shunt, 2=Previous variceal bleed, 3=Not present
1=No, 2=Yes, 8=Not reported, 9=Unknown
1=No, 2=Yes, 8=Not reported, 9=Unknown
1=Sputum, 2=Blood, 3=Urine, 4=Ascities/Drain fluid, 5=Wound, 7=other
1=Negative, 2=Positive, 6=results awaited, 7=Not tested, 8=Not reported, 9=Unknown
1=Negative, 2=Positive, 6=results awaited, 7=Not tested, 8=Not reported, 9=Unknown
1=Negative, 2=Positive, 6=results awaited, 7=Not tested, 8=Not reported, 9=Unknown
1=Negative, 2=Positive, 6=results awaited, 7=Not tested, 8=Not reported, 9=Unknown

1=Negative, 2=Positive, 6=results awaited, 7=Not tested, 8=Not reported, 9=Unknown
1=Negative, 2=Positive, 6=results awaited, 7=Not tested, 8=Not reported, 9=Unknown
1=Negative, 2=Positive, 6=results awaited, 7=Not tested, 8=Not reported, 9=Unknown
1=Negative, 2=Positive, 6=results awaited, 7=Not tested, 8=Not reported, 9=Unknown
1=Negative, 2=Positive, 6=results awaited, 7=Not tested, 8=Not reported, 9=Unknown
1=Negative, 2=Positive, 6=results awaited, 7=Not tested, 8=Not reported, 9=Unknown
1=Negative, 2=Positive, 6=results awaited, 7=Not tested, 8=Not reported, 9=Unknown
1=Negative, 2=Positive, 6=results awaited, 7=Not tested, 8=Not reported, 9=Unknown
1=Negative, 2=Positive, 6=results awaited, 7=Not tested, 8=Not reported, 9=Unknown
1=Negative, 2=Positive, 6=results awaited, 7=Not tested, 8=Not reported, 9=Unknown

0=No, 1=Yes

0=No, 1=Yes

40=Liver, 80=IF liver-only, 83=IF liver/bowel/pancreas, 84=IF multivisceral, 110=Liver & kidney, 115=Liver, kidne
0=No, 1=Yes
1=Orthotopic, 2=Heterotopic
40=Whole liver, 41=Left liver lobe, 42=Right liver lobe, 46= Left lateral liver segment

0=No, 1=Yes
0=No, 1=Yes
Null=registered transplant, 91= No active record, 92=Transplanted whilst suspended, 93=Transplanted after tran
0=Whole, 1=Reduced, 2=Split

1=DBD, 2=DCD, 5=Living related, 6=Living, unrelated, 7=Domino 13=Living unrelated - Altruistic

see reference donor COD

1=Negative, 2=Positive, 6=results awaited, 7=Not tested, 8=Not reported, 9=Unknown
0=No, 1=Yes, 9=Unknown
0=No, 1=Yes, 9=Unknown
0=No, 1=Yes, 9=Unknown
0=No, 1=Yes, 9=Unknown
0=No, 1=Yes, 9=Unknown
0=No, 1=Yes, 9=Unknown
see reference perfusate used
1=Healthy, 2=Suboptimal
1=No, 2=Yes, 8=Not reported, 9=Unknown
1=No, 2=Yes, 8=Not reported, 9=Unknown
1=Single, 2=Accessory

1=No stent, 2=T-tube, 3=Roux, 4=Stent
1=Single 2=Multiple
1=No, 2=Yes, 8=Not reported, 9=Unknown
1=No, 2=Yes, 8=Not reported, 9=Unknown

1=No, 2=Yes, 8=Not reported, 9=Unknown
1=No, 2=Yes, 8=Not reported, 9=Unknown
1=No, 2=Yes, 8=Not reported, 9=Unknown
1=No, 2=Yes, 8=Not reported, 9=Unknown
1=No, 2=Yes, 8=Not reported, 9=Unknown
1=No, 2=Yes, 8=Not reported, 9=Unknown
1=No, 2=Yes, 8=Not reported, 9=Unknown
1=No, 2=Yes, 8=Not reported, 9=Unknown
1=No, 2=Yes, 8=Not reported, 9=Unknown
1=No, 2=Yes, 8=Not reported, 9=Unknown
1=No, 2=Yes, 8=Not reported, 9=Unknown
1=No, 2=Yes, 8=Not reported, 9=Unknown
1=No, 2=Yes, 8=Not reported, 9=Unknown
1=No, 2=Yes, 8=Not reported, 9=Unknown
1=Able to carry out normal activity without restriction, 2=Only restricted in physically strenuous activity, 3=Can m

1=No or minor renal impairment, 2=Required transient renal filtration, 3=Short-term dialysis, 4=Long-term dialysi
1=No, 2=Yes, 8=Not reported, 9=Unknown
1=No, 2=Yes, 8=Not reported, 9=Unknown
1=No, 2=Yes, 8=Not reported, 9=Unknown
1=No, 2=Yes, 8=Not reported, 9=Unknown
1=No leak, 2=Leak not treated, 3=Leak requiring reoperation, 4=Other radiological intervention
1=No, 2=Yes, 8=Not reported, 9=Unknown
1=Reoperated, 2=Balloon dilatation, 3=Stenting
1=No, 2=Yes, 8=Not reported, 9=Unknown
1=No, 2=Yes, 8=Not reported, 9=Unknown
1=No, 2=Yes, 8=Not reported, 9=Unknown
1=No, 2=Yes, 8=Not reported, 9=Unknown
1=No, 2=Yes, 8=Not reported, 9=Unknown
1=No, 2=Yes, 8=Not reported, 9=Unknown
1=No, 2=Yes, 8=Not reported, 9=Unknown
1=No, 2=Yes, 8=Not reported, 9=Unknown
1=No, 2=Yes, 8=Not reported, 9=Unknown
1=No, 2=Yes, 8=Not reported, 9=Unknown
1=No, 2=Yes, 8=Not reported, 9=Unknown
1=No, 2=Yes, 8=Not reported, 9=Unknown

1=No, 2=Yes, 8=Not reported, 9=Unknown
1=No, 2=Yes, 8=Not reported, 9=Unknown

1=Able to carry out normal activity without restriction, 2=Only restricted in physically strenuous activity, 3=Can m

1=No, 2=Yes, 8=Not reported, 9=Unknown
1=No, 2=Yes, 8=Not reported, 9=Unknown
1=No, 2=Yes, 8=Not reported, 9=Unknown
1=No, 2=Yes, 8=Not reported, 9=Unknown
1=No, 2=Yes, 8=Not reported, 9=Unknown
1=No, 2=Yes, 8=Not reported, 9=Unknown
1=No, 2=Yes, 8=Not reported, 9=Unknown
1=No, 2=Yes, 8=Not reported, 9=Unknown

1=Able to carry out normal activity without restriction, 2=Only restricted in physically strenuous activity, 3=Can m

1=No, 2=Yes, 8=Not reported, 9=Unknown
1=No, 2=Yes, 8=Not reported, 9=Unknown
1=No, 2=Yes, 8=Not reported, 9=Unknown
1=No, 2=Yes, 8=Not reported, 9=Unknown
1=No, 2=Yes, 8=Not reported, 9=Unknown
1=No, 2=Yes, 8=Not reported, 9=Unknown
1=No, 2=Yes, 8=Not reported, 9=Unknown
1=No, 2=Yes, 8=Not reported, 9=Unknown

For peer review only - http://bmjopen.bmj.com/site/about/guidelines.xhtml

see reference liver cause of failure
1=No, 2=Yes, 8=Not reported, 9=Unknown
1=Still alive, graft functioning, 2=Graft failed, 3=Death with a functioning graft, 4=Death only reported

1=Still alive, graft functioning, 2=Graft failed, 3=Death with a functioning graft, 4=Death only reported
see reference recipient COD
see reference recipient COD

0=Censored, 1=Event (Graft failure or death)

0=Censored, 1=Event (Graft failure)

0=Censored, 1=Event (Death of recipient)

For peer review only

care. Unable to do any form of work. 4=Only capable of limited self care. Confined mostly to bed or c[illegible]

aking and obeying simple commmands. Inarticulate speech. Marked confusion. 4= Coma. Cannot be

For peer review only

ung & liver, 140=Liver & pancreas, 160=Heart & liver, 190=Lung & liver

ter removal, 95=Transplanted after death reported

For peer review only

care. Unable to do any form of work. 4=Only capable of limited self care. Confined mostly to bed or cl

For peer review only

care. Unable to do any form of work. 4=Only capable of limited self care. Confined mostly to bed or cl

care. Unable to do any form of work. 4=Only capable of limited self care. Confined mostly to bed or cl

For peer review only

For peer review only

For peer review only

hair, 5=Completely reliant on nursing/medical care, 7=Aged five years or less

aroused

For peer review only

For peer review only

hair, 5=Completely reliant on nursing/medical care, 7=Aged five years or less

For peer review only

hair, 5=Completely reliant on nursing/medical care, 7=Aged five years or less

hair, 5=Completely reliant on nursing/medical care, 7=Aged five years or less

For peer review only

	**Activity/Organ(s):**
	**Geographical area:**
	**Mark data items required with an X**
**X**	**Variable**
	ATX_ID
	ARECIP_ID
	ADONOR_ID
X	rhosp
	NHS_GRP
X	RETHNIC
X	RSEX
X	rage
	rpaed
	RAGE_GRP2
X	rbg
X	rheight
X	rweight
X	RBMI
X	pcd
X	reg_primary_disease
	reg_prev_heart_surgery
	reg_prev_thoracotomy
	reg_prev_sudden_death
	reg_antiarrhythmics
	reg_aicd
	reg_hypertension
	reg_vascular_disease
	reg_cerebrovascular
	reg_cholesterol
	reg_diabetes
x	reg_prev_malignancy
	reg_prednisolone
x	reg_smoker
	reg_bilirubin
	reg_creatinine
	reg_home_oxygen
	reg_nyha_class
	reg_pa_systolic
	reg_pa_mean
	reg_pcw
	reg_cardiac_output
	reg_ejection_fraction
	reg_fev1
	reg_fvc
	reg_vo2_max
	reg_six_min_walk
	reg_in_hospital
	reg_ventilated
	reg_inotropes
	reg_iabp
	reg_vad
	reg_tah
	reg_ecmo

review only

X	CMV_TX
X	HCV_TX
X	HBV_TX
	HIV_TX
X	rwtime
X	uwtime
X	suwtime
X	tx_yr
	f_yr
	retunit
X	TX_TYPE
X	ORG_TXD
X	multi_tx
X	organ
X	tx
	het
X	GRAFT_NO
X	gno_hrt
X	gno_lng
X	ALLTX_NO
	urgent
X	dtype
	dgrp
	DD_YR
	dhosp
X	DCOD
X	dage
	dpaed
	dage_grp2
X	dsex
X	dheight
X	dweight
X	dethnic
X	DBMI
X	dbg
	ABOmatch
X	dcmv
	DHBSAG
	DHBCAB
X	DHCV
	DHIV
X	DEBV
	DHTLV
	DTOXO
	DSYPHILIS
	DPAST_ALCOHOL_ABUSE
	DPAST_CARDIO_DISEASE
	DPAST_DIABETES
	DPAST_FAMILY_DIABETES
	DPAST_FAMILY_DIABETES_TYPE
	DPAST_DRUG_ABUSE
	DPAST_HYPERTENSION
	DPAST_LIVER_DISEASE

review only

For peer review only - http://bmjopen.bmj.com/site/about/guidelines.xhtml

	DPAST_OTHER
x	DPAST_SMOKER
x	DPAST_SMOKER_AMOUNT
x	DPAST_TUMOUR
	DPAST_UTI
	DPAST_ALLERGY
	DHYPOTENSION
	DCARDIAC_ARREST
	DRESPIRATORY_ARREST
	HEART_PERFUSATE
	LUNG_PERFUSATE
	HEART_PERFUSION_METHOD
	LUNG_PERFUSION_METHOD
	FIRST_LUNG
	IT
	WIT
	PRE_OP_INFECTION
	NYHA
	IN_HOSP
	RVENT
	INOTROPES
	IABP
	ECMO
	VAD
	TAH
	RCREAT
	DAYS_TX_HDU
	DAYS_TX_ITU
	DAYS_TX_HOSP
	THEATRE
	HAEMOFILTRATION
	IABP_POST_OP
	OTHER_POST_OP
X	REJECTIONS
	INFECTIONS
	ORGANISMS
	infect1_TX
	infect2_TX
	infect3_TX
x	CYCLOSPORIN_TX
x	FK506_TX
x	AZATHIOPRINE_TX
x	CORTICOSTEROIDS_TX
x	SIROLIMUS_TX
x	ALG_induction_TX
x	ALG_rejection_TX
x	OKT3_induction_TX
x	OKT3_rejection_TX
	TLI_TX
	METHOTREXATE_TX
	IMMUNO_DRUG_OTHER_TX
	IMMUNO_TEXT_TX
	RCOD

review only

X	gstatus
X	cof
X	LOST_FUP
	vtl_stat
	re_tx
	tsurv
	tcens
X	gsurv
X	gcens
	psurv
	pcens
	HOSPITAL_ADMISSIONS_j
	REJECTION_EPISODES_j
	AIRWAY_COMPLICATIONS_j
	INFECTIONS_j
	FEV1_1_j
	FEV1_2_j
	NYHA_CLASS_j
	ORGANISM_TEXT_j
	INFECT_1_j
	INFECT_2_j
	INFECT_3_j
	CREATININE_j

r review only

Cardiothoracic:
United Kingdom
Description
Anonymised transplant identification number
Anonymised recipient identification number
Anonymised donor identification number
Anonymised unit that received and transplanted the organ
Recipient NHS group
Recipient ethnicity
Recipient sex
Recipient age
Recipient paediatric (<16) or adult
Recipient age in 10 year increments
Recipient blood group
Height of recipient (cm)
Weight of recipient (kg)
Recipient BMI
Primary cause of cardiothoracic disease
Primary disease at registration
Number of previous open heart surgery operations at registration
Previous thoracotomy at registration
Previous sudden death episode at registration
Antiarrhythmics (excluding digoxin) at registration
AICD at registration
Hypertension requring treatment in the last 5 years at registration
Peripheral vascular disease with intervention performed or planned at registration
Cerebrovascular disease at registration
Cholesterol (mmol/l) at registration
Diabetes at registration
Previous malignancy at registration
Daily dose of prednisolone (mg) at registration
Smoker more than 5 a day within 6 months at registration
Bilirubin (µmol/l) at registration
Creatinine (µmol/l) at registration
Home oxygen
NYHA class at registration
PA systolic (mm Hg) at registration
PA mean (mm Hg) at registration
PCW or LAP (mm Hg) at registration
Cardiac output (l/min) at registration
Ejection fraction (%) at registration
FEV1 (litres) at registration
FVC (litres) at registration
VO2 max (ml/kg/min) at registration
6 minute walk test (m) at registration
In hospital at registration
Patient on ventilator whilst in hopsital at registration
Patient on intopes whilst in hopsital at registration
IABP whilst in hospital at registration
VAD whilst in hospital at registration
TAH whilst in hospital at registration
ECMO whilst in hospital at registration

Recipient CMV status at transplant
Recipient HCV status at transplant
Recipient HBV status at transplant
Recipient HIV status at transplant
Routine wait time (days)
Urgent wait time (days)
Super-urgent wait time (days)
Year of transplant
Financial year of transplant
Anonymised unit that retrieved the organ
Type of transplant
Type of cardiothoracic transplant
Multi-organ transplant indicator
Organ transplanted
Indicator organ transplanted
Heart transplanted was heterotopic or orthotopic
Number of Heart/lung transplants
Number of heart transplants
Number of lung transplants
Number of all transplants
Urgency status of transplant
Donor type
Donor type group
Year of donation
Anonymised donation centre
Donor cause of death
Donor age
Donor paediatric (<16) or adult
Donor age in 10 year increments
Donor sex
Donor height (cm)
Donor weight (kg)
Donor ethnicity
Donor BMI
Donor blood group
Donor vs recipient blood group
Donor CMV test result
Donor HBSAG test result
Donor HBCAB test result
Donor HCV test result
Donor HIV test result
Donor EBV test result
Donor HTLV test result
Donor Toxo test result
Donor syphilis test result
Donor history of alcohol abuse
Donor cardio disease
Donor diabetes
Donors family history of diabetes
Donors family history type of diabete
Donor history of drug abuse
Donor hypertension
Donor liver disease

Donor past other
Donor smoker
Donor smoker amount (number smoked per day)
Donor tumour
Donor UTI
Donor allergy
Donor hypotension (less than 70mmHg systolic)
Donor cardiac arrest
Donor respiratory arrest
Heart perfusion fluid
Lung perfusion fluid
Heart perfusion method
Lung perfusion method
First lung perfused
Total ischaemic time hours from cross clamp of donor to reperfusion time (hours)
Warm ischemia time (minutes)
Infection requiring antibiotics in the last 6 weeks at transplant
NYHA class at transplant
In hospital at transplant
Patient on ventilator whilst in hospital at transplant
Patient on intopes whilst in hospital at transplant
IABP whilst in hospital at transplant
ECMO whilst in hospital at transplant
VAD whilst in hospital at transplant
TAH whilst in hospital at transplant
Creatinine (μmol/l) at transplant
Number of days spent in HDU after transplant
Number of days spent in ITU after transplant
Number of days spent in hospital after transplant
Complications - return to theatre
Complications - haemofiltration/haemodialysis
Complications - IABP post-op
Complications - other mechanical assistance post-op
Number of treated rejection episodes at transplant
Number of serious infection episodes at transplant
If infection at transplant what was the organism
Primary site of infection at transplant
Secondary site of infection at transplant
Teritary site of infection at transplant
Cyclosporin immunosuppression at transplant
FK506 immunosuppression at transplant
Azathioprine immunosuppression at transplant
Corticosteroids immunosuppression at transplant
Sirolimus immunosuppression at transplant
ALG/ATG antibody inducation at transplant
ALG/ATG antibody for rejection at transplant
OKT3 antibody inducation at transplant
OKT3 antibody for rejection at transplant
TLI immunosuppression at transplant
Methotrexate immunosuppression at transplant
Other immunosuppression at transplant
Other immunosuppression text at transplant
Recipient cause of death

Most recent outcome of graft
Cause of failure
Lost to follow up
Vital status of recipient
Whether patient was re-transplanted
Transplant survival time (days)
Transplant censoring indicator
Graft survival time (days)
Graft censoring indicator
Patient survival time (days)
Patient censoring indicator
Number of hospital admissions in follow-up period j (j=3, 12, 24, 36, … corresponding to months post-transplant)
Number of rejection episodes in follow-up period j (j=3, 12, 24, 36, … corresponding to months post-transplant)
Airway complications in follow-up period j (j=3, 12, 24, 36, … corresponding to months post-transplant)
Number of serious infection episodes in follow-up period j (j=3, 12, 24, 36, … corresponding to months post-trans
First of two best FEV litres at follow up period j (j=3, 12, 24, 36, … corresponding to months post-transplant)
Second of two best FEV litres follow up period j (j=3, 12, 24, 36, … corresponding to months post-transplant)
NYHA Class at follow up period j (j=3, 12, 24, 36, … corresponding to months post-transplant)
If infection in follow-up period j (j=3, 12, 24, 36, … corresponding to months post-transplant), what was the orgar
Primary site of infection in follow-up period j (j=3, 12, 24, 36, … corresponding to months post-transplant)
Secondary site of infection in follow-up period j (j=3, 12, 24, 36, … corresponding to months post-transplant)
Teritary site of infection in follow-up period j (j=3, 12, 24, 36, … corresponding to months post-transplant)
Creatinine (μmol/l) at follow up period j (j=3, 12, 24, 36, … corresponding to months post-transplant)

eview only

Reference

1=Group 1, 2=Group 2, 8=Not reported, 9=Unknown

NR=Not reported, UKN=Unknown

888=Not reported
888.8=Not reported

see reference CARDIO PRIMARY DISEASE
see reference CARDIO PRIMARY DISEASE
88=Not reported, 99=Unknown
1=No, 2=Yes, 8=Not reported 9=Unknown
1=No, 2=Yes, 8=Not reported 9=Unknown
1=No, 2=Yes, 8=Not reported 9=Unknown
1=No, 2=Yes, 8=Not reported 9=Unknown
1=No, 2=Yes, 8=Not reported 9=Unknown
1=No, 2=Yes, 8=Not reported 9=Unknown
1=No, 2=Yes, permanent disability, 3=Yes, transient disability, 8=Not reported 9=Unknown
88.8=Not reported, 99.9=Unknown
1=No, 2=Yes, insulin dependent, 3=Yes, not insulin dependent, 8=Not reported, 9=Unknown
1=No, 2=Yes, 8=Not reported 9=Unknown
888.8=Not reported, 999.9=Unknown
1=No, 2=Yes, 8=Not reported 9=Unknown
8888=Not reported, 9999=Uknown
8888=Not reported, 9999=Uknown
1=No, 2=Yes, 8=Not reported 9=Unknown
1=I, 2=II, 3=III, 4=IV, 8=Not reported, 9=Unknown
888=Not reported, 999=Unknown
888=Not reported, 999=Unknown
88=Not reported, 99=Unknown
88.8=Not reported, 99.9=Unknown
88=Not reported, 99=Unknown
88.88=Not reported, 9.99=Unknown
88.88=Not reported, 9.99=Unknown
88=Not reported, 99=Unknown
888=Not reported, 999=Unknown
1=No, 2=Yes, 8=Not reported 9=Unknown
1=No, 2=Yes, 8=Not reported 9=Unknown
1=No, 2=Yes, 8=Not reported 9=Unknown
1=No, 2=Yes, 8=Not reported 9=Unknown
1=None, 2=Left, 3=Right, 4=Both 8=Not reported, 9=Unknown
1=No, 2=Yes, 8=Not reported 9=Unknown
1=No, 2=Yes, 8=Not reported 9=Unknown

For peer review only - http://bmjopen.bmj.com/site/about/guidelines.xhtml

1=Negative, 2=Positive, 3=Indeterminate, 4=Repeat reactive, 5=Confirmed positive, 6=results awaited, 7=Not te
1=Negative, 2=Positive, 3=Indeterminate, 4=Repeat reactive, 5=Confirmed positive, 6=results awaited, 7=Not te
1=Negative, 2=Positive, 3=Indeterminate, 4=Repeat reactive, 5=Confirmed positive, 6=results awaited, 7=Not te
1=Negative, 2=Positive, 3=Indeterminate, 4=Repeat reactive, 5=Confirmed positive, 6=results awaited, 7=Not te

30=Heart, 60=Lung, 63=Double lung, 65=Bilateral single lung, 66=Partial lung, 70=Heart/lung, 120=Heart, lung &
35=Orthotropic heart, 36=Heterotopic heart, 61=Left lung, 62=Right lung, 63=Double lung, 65=Bilateral single lu
0=Single organ transplant, 1=Multi-organ transplant
30=Heart, 61=Left lung, 62=Right lung, 63=Lung pair
H=Heart, L=Lungs, HL=Heart/Lungs
0=Orthotopic, 1=Heterotopic

0=Routine/Non-urgent, 1=Urgent, 2=Super-urgent
1=DBD, 2=DCD, 5=Living related, 6=Living, unrelated, 7=Domino 13=Living unrelated - Altruistic

see reference DONOR COD

NR=Not reported, UKN=Unknown
888=Not reported, 999=Unknown
888=Not reported, 999=Unknown

1=Identical, 2=Compatible, 3=Incompatible
1=Negative, 2=Positive, 3=Indeterminate, 4=Repeat reactive, 5=Confirmed positive, 6=results awaited, 7=Not te
1=Negative, 2=Positive, 3=Indeterminate, 4=Repeat reactive, 5=Confirmed positive, 6=results awaited, 7=Not te
1=Negative, 2=Positive, 3=Indeterminate, 4=Repeat reactive, 5=Confirmed positive, 6=results awaited, 7=Not te
1=Negative, 2=Positive, 3=Indeterminate, 4=Repeat reactive, 5=Confirmed positive, 6=results awaited, 7=Not te
1=Negative, 2=Positive, 3=Indeterminate, 4=Repeat reactive, 5=Confirmed positive, 6=results awaited, 7=Not te
1=Negative, 2=Positive, 3=Indeterminate, 4=Repeat reactive, 5=Confirmed positive, 6=results awaited, 7=Not te
1=Negative, 2=Positive, 3=Indeterminate, 4=Repeat reactive, 5=Confirmed positive, 6=results awaited, 7=Not te
1=Negative, 2=Positive, 3=Indeterminate, 4=Repeat reactive, 5=Confirmed positive, 6=results awaited, 7=Not te
1=Negative, 2=Positive, 3=Indeterminate, 4=Repeat reactive, 5=Confirmed positive, 6=results awaited, 7=Not te
1=No, 2=Yes, 8=Not reported, 9=Unknown
1=No, 2=Yes, 8=Not reported, 9=Unknown
1=No, 2=Yes, 8=Not reported, 9=Unknown
1=No, 2=Yes, 8=Not reported, 9=Unknown
3=Type 1, 4=Type 2, 5=Type 1 & 2, 8=Not reported, 9=Unknown
1=No, 2=Yes, 8=Not reported, 9=Unknown
1=No, 2=Yes, 8=Not reported, 9=Unknown
1=No, 2=Yes, 8=Not reported, 9=Unknown

1=No, 2=Yes, 8=Not reported, 9=Unknown
1=No, 2=Yes, 8=Not reported, 9=Unknown
99=Unknown
1=No, 2=Yes, 8=Not reported, 9=Unknown
1=No, 2=Yes, 8=Not reported, 9=Unknown
1=No, 2=Yes, 8=Not reported, 9=Unknown
1=No, 2=Yes, 8=Not reported 9=Unknown
1=No, 2=Yes, 8=Not reported 9=Unknown
1=No, 2=Yes, 8=Not reported 9=Unknown
see reference PERFUSATE USED
see reference PERFUSATE USED
1=Flush, 2=Core-cooling, 3=Immersion, 6=Not perfused/not applicable, 7=Other, 8=Missing, 9=Unknown
1=Flush, 2=Core-cooling, 3=Immersion, 6=Not perfused/not applicable, 7=Other, 8=Missing, 9=Unknown
61=Left, 62=Right

1=No, 2=Yes, 8=Not reported 9=Unknown
1=I, 2=II, 3=III, 4=IV, 8=Not reported, 9=Unknown
1=No, 2=Yes, 8=Not reported 9=Unknown
1=No, 2=Yes, 8=Not reported 9=Unknown
1=No, 2=Yes, 8=Not reported 9=Unknown
1=No, 2=Yes, 8=Not reported 9=Unknown
1=No, 2=Yes, 8=Not reported 9=Unknown
1=None, 2=Left, 3=Right, 4=Both, 8=Not reported, 9=Unknown
1=No, 2=Yes, 8=Not reported, 9=Unknown
8888=Not reported, 9999=Uknown

1=No, 2=Yes, 8=Not reported 9=Unknown
1=No, 2=Yes, 8=Not reported 9=Unknown
1=No, 2=Yes, 8=Not reported 9=Unknown
1=No, 2=Yes, 8=Not reported 9=Unknown
88=Not reported, 99=Unknown
88=Not reported, 99=Unknown

1=No, 2=Yes, 8=Not reported, 9=Unknown
1=No, 2=Yes, 8=Not reported, 9=Unknown
1=No, 2=Yes, 8=Not reported, 9=Unknown
1=No, 2=Yes, 8=Not reported, 9=Unknown
1=No, 2=Yes, 8=Not reported, 9=Unknown
1=No, 2=Yes, 8=Not reported, 9=Unknown
1=No, 2=Yes, 8=Not reported, 9=Unknown
1=No, 2=Yes, 8=Not reported, 9=Unknown
1=No, 2=Yes, 8=Not reported, 9=Unknown
1=No, 2=Yes, 8=Not reported, 9=Unknown
1=No, 2=Yes, 8=Not reported, 9=Unknown
1=No, 2=Yes, 8=Not reported, 9=Unknown

see reference RECIPIENT COD

1=Functioning, 2=Failed, 3=Died with functioning graft, 4=Death only reported, 8=Not reported, 9=Unknown
see reference CARDIOTHORACIC CAUSES OF FAILURE
1=No, 2=Yes, 8=Not reported, 9=Unknown
1=Alive, 2=Dead, 8=Missing
0=No, 1=Yes
-100=Not reported
0=Censored, 1=Event (Graft failure or death)
-100=Not reported
0=Censored, 1=Event (Graft failure)
-100=Not reported, Will be missing for regrafts
0=Censored, 1=Event (Death of recipient)

1=No, 2=Yes, 8=Not reported, 9=Unknown

9.99=Unknown
9.99=Unknown
1=I, 2=II, 3=III, 4=IV, 8=Not reported, 9=Unknown

eview only

For peer review only

ey & liver, 180=Heart, lung & kidney, 190=Lung & liver

For peer review only

	**Activity/Organ(s):**
	**Geographical area:**
	**Mark data items required with an X**
**X**	**Variable**
	ATX_ID
	ADONOR_ID
	ARECIP_ID
	DTYPE
	SOLID
	DAGE
	DSEX
	DCOD
	RAGE
	RSEX
	TX_YR
	GTYPE
	DIAG
	GNO_OPERATED
	GNO_FELLOW
	LOGMAR0
	LOGMAR1
	GSURV
	GCENS

For peer review only

Ocular tissue transplant dataset
United Kingdom
**Description**
Anonymised transplant identification number
Anonymised donor identification number
Anonymised recipient identification number
Donor type
Solid organs donated
Donor age
Donor sex
Donor cause of death
Recipient age
Recipient sex
Calendar year of transplant
Graft type
Indication for ocular tissue transplant
Graft number in operated eye
Number of previous grafts in fellow eye
LogMAR acuity at transplant
LogMAR acuity at one-year (8-16 months post-transplant)
Graft survival (days)
Graft censor

For peer review only

Reference

1=DBD, 2=DCD, 10=Tissue only
0=No, 1=Yes

1=Male, 2=Female, 8=Not reported, 9=Unknown
see reference donor COD

1=Male, 2=Female, 9=Unknown

1=Penetrating keratoplasty (PK), 2=Superficial lamellar, 3=Deep anterior lamellar keratoplasty (DALK), 5=sclera/cornea in ethanol, 6=stem cell allograft, 7=Other, 10=Descemet's membrane endothelial keratoplasty
see Recipient Diagnosis

0=Censored, 1=Event (graft failure)

For peer review only

	**Activity/Organ(s):**
	**Time period:**
	**Geographical area:**
	**Mark data items required with an X**
**X**	**Variable**
	ATX_ID
	ADONOR_ID
	ARECIP_ID
	DTYPE
	DAGE_GRP
	DSEX
	DCOD_GRP
	RAGE_GRP
	RSEX
	TX_YR
	TX_TYPE
	AMM_1998
	BMM_1998
	DRMM_1998
	KID_PD_GRP
	KID_CIT_MINS
	KID_GSURV
	KID_GCENS
	KID_PSURV
	KID_PCENS
	KID_TSURV
	KID_TCENS
	PAN_PD_GRP
	PAN_CIT_MINS
	ISLET_URGENCY_STATUS
	PAN_GSURV
	PAN_GCENS
	PAN_PSURV
	PAN_PCENS
	PAN_TSURV
	PAN_TCENS
	LIV_PD_GRP
	LIV_CIT_MINS
	LIV_URGENCY_STATUS
	LIV_GSURV
	LIV_GCENS
	LIV_PSURV
	LIV_PCENS
	LIV_TSURV
	LIV_TCENS
	CARDIO_PD_GRP
	CARDIO_IT_MINS
	CARDIO_URGENCY_STATUS
	LNG_GSURV
	LNG_GCENS
	LNG_PSURV
	LNG_PCENS
	LNG_TSURV

For peer review only

LNG_TCENS
HRT_GSURV
HRT_GCENS
HRT_PSURV
HRT_PCENS
HRT_TSURV
HRT_TCENS

For peer review only

Public all organ transplant dataset
1 January 1995 - 31 December 2016
United Kingdom
**Description**
Anonymised transplant identification number
Anonymised Donor identification number
Anonymised Recipient identification number
Donor type
Donor age at donation
Sex of donor
Donor cause of death
Recipient age at transplant
Sex of recipient
Calendar year of transplant
Transplant type
Number of mismatches at A locus
Number of mismatches at B locus
Number of mismatches at DR locus
Primary renal disease group
Kidney cold ischaemia time (mins)
Graft survival time (days) for kidney transplant
Graft survival censoring indicator for kidney transplant
Patient survival time (days) for kidney transplant
Patient survival censoring indicator for kidney transplant
Transplant survival time (days) for kidney transplant
Transplant survival censoring indicator for kidney transplant
Primary renal disease group
Pancreas cold ischaemia time (mins)
Islet urgency status of recipient
Graft survival time (days) for pancreas transplant
Graft survival censoring indicator for pancreas transplant
Patient survival time (days) for pancreas transplant
Patient survival censoring indicator for pancreas transplant
Transplant survival time (days) for pancreas transplant
Transplant survival censoring indicator for pancreas transplant
Primary liver disease group
Liver cold ischaemia time (mins)
Urgency status of recipient
Graft survival time (days) for liver transplant
Graft survival censoring indicator for liver transplant
Patient survival time (days) for liver transplant
Patient survival censoring indicator for liver transplant
Transplant survival time (days) for liver transplant
Transplant survival censoring indicator for liver transplant
Primary cardio disease group
Cardio ischaemia time (mins)
Urgency status of recipient
Graft survival time (days) for lung transplant
Graft survival censoring indicator for lung transplant
Patient survival time (days) for lung transplant
Patient survival censoring indicator for lung transplant
Transplant survival time (days) for lung transplant

For peer review only

Transplant survival censoring indicator for lung transplant
Graft survival time (days) for heart transplant
Graft survival censoring indicator for heart transplant
Patient survival time (days) for heart transplant
Patient survival censoring indicator for heart transplant
Transplant survival time (days) for heart transplant
Transplant survival censoring indicator for heart transplant

For peer review only

Reference
See donor type in Appendix 1
See donor age group in Appendix 2
1=Male, 2=Female, 8=Not reported, 9=Unknown
See donor cause of death codes in Appendix 3
See recipient age group in Appendix 2
1=Male, 2=Female, 8=Not reported, 9=Unknown
See transplant type in Appendix 5
0=zero mismatches, 1=1 mismatch, 2=2 mismatches, 7=Not tested, 9=Unknown
0=zero mismatches, 1=1 mismatch, 2=2 mismatches, 7=Not tested, 9=Unknown
0=zero mismatches, 1=1 mismatch, 2=2 mismatches, 7=Not tested, 9=Unknown
See Primary renal disease code in Appendix 4
0=Censored, 1=Event (graft failure)
0=Censored, 1=Event (death of patient)
0=Censored, 1=Event (graft failure or death of patient)
See Primary renal disease code in Appendix 4
0=Routine, 1=Priority
0=Censored, 1=Event (graft failure)
0=Censored, 1=Event (death of patient)
0=Censored, 1=Event (graft failure or death of patient)
See Primary liver disease code in Appendix 4
0=Elective, 1=Super Urgent
0=Censored, 1=Event (graft failure)
0=Censored, 1=Event (death of patient)
0=Censored, 1=Event (graft failure or death of patient)
See Primary cardiothoracic disease code in Appendix 4
0=Elective, 1=Urgent
0=Censored, 1=Event (graft failure)
0=Censored, 1=Event (death of patient)

For peer review only

0=Censored, 1=Event (graft failure or death of patient)

0=Censored, 1=Event (graft failure)

0=Censored, 1=Event (death of patient)

0=Censored, 1=Event (graft failure or death of patient)

For peer review only

References

DONOR COD

0 Living donor
10 Intracranial haemorrhage
11 Intracranial thrombosis
12 Brain tumour
13 Hypoxic brain damage - all causes
19 Intracranial - type unclassified (CVA)
20 Trauma RTA - car
21 Trauma RTA - motorbike
22 Trauma RTA - pushbike
23 Trauma RTA - pedestrian
24 Trauma RTA - Other
29 Trauma RTA - unknown type
30 Other trauma - known or suspected suicide
31 Other trauma - accident
39 Other trauma - unknown cause
40 Cardiac arrest
41 Myocardial infarction
42 Aneurysm
42 Aortic Aneurysm
43 Ischaemic heart disease
44 Congestive heart failure
45 Pulmonary embolism
49 Cardiovascular - type unclassified
50 Chronic pulmonary disease
51 Pneumonia
52 Asthma
53 Respiratory failure
54 Carbon monoxide poisoning
59 Respiratory - type unclassified (inc smoke inhalation)
60 Cancer (other than brain tumour)
70 Meningitis
71 Septicaemia
72 Infections - type unclassified
73 Acute blood loss/hypovolaemia
74 Liver failure (not self poisoning)
75 Renal failure
76 Multi-organ failure
77 Sudden Infant Death Syndrome (SIDS)
78 Burns
80 Alcohol poisoning
81 Paracetamol overdose
82 Other drug overdose, please specify
85 Self poisoning - type unclassified
88 Not reported
90 OTHER
98 Other, please specify
99 Unknown

RECIPIENT COD

500 Cause of death uncertain/not determined
511 Myocardial ischaemia and infarction

512 Hyperkalaemia
513 Haemorrhagic pericarditis
514 Other causes of cardiac failure
515 Sudden unexplained cardiac death
516 Hypertensive cardiac failure
517 Hypokalaemia
518 Fluid overload
519 Elevated pvr/pulmonary hypertension
520 Airway dehiscence
521 Pulmonary embolus
522 Cerebro-vascular accident
523 Gastro-intestinal haemorrhage
524 Haemorrhage from graft site
525 Haemorrhage from vascular access or dialysis circuit
526 Haemorrhage from ruptured vascular aneurysm (not code 22 or 23)
527 Haemorrhage from surgery (not code 23,24 or 26)
528 Other haemorrhage (not codes 23-27)
529 Mesenteric infarction
530 Pulmonary infection (protozoal)
531 Pulmonary infection (bacterial)
532 Pulmonary infection (viral)
533 Pulmonary infection (fungal)
534 Infections elsewhere (except viral hepatitis)
535 Septicaemia
536 Tuberculosis (lung)
537 Tuberculosis (elsewhere)
538 Generalized viral infection
539 Peritonitis (not code 70)
541 Liver - due to hepatitis b virus
542 Liver - other viral hepatitis
543 Liver - drug toxicity
544 Cirrhosis - not viral
545 Cystic liver disease
546 Liver failure - cause unknown
547 Renal failure
548 Recurrent primary disease - benign
549 Recurrent primary disease - malignant
551 Patient refused further treatment
552 Suicide
553 Therapy ceased for any other reason
554 Esrf treatment withdrawn for medical reasons
561 Uraemia caused by graft failure
562 Pancreatitis
563 Bone marrow depression
564 Cachexia
566 Lymphoid malignant disease possibly induced by immunosuppressive therapy
567 Lymphoid malignant disease not induced by immunosuppressive therapy
568 Malignant disease: lymphoproliferative disorders (except 66)
569 Dementia
570 Sclerosing (or adhesive) peritoneal disease
571 Perforation of peptic ulcer
572 Perforation of colon
573 Non-lymphoid malignant disease possibly induced by immunosuppressive therapy

574 Non-lymphoid malignant disease not induced by immunosuppressive therapy
575 Early graft dysfunction
576 Cardiac tamponade
577 Ards
578 Respiratory failure
579 Multi-system failure
581 Accident related to treatment
582 Accident unrelated to treatment
590 Donor organ failure
595 Other identified cause of death
598 Other identified cause of death
599 Unknown
600 Peritonitis (bacterial, with peritoneal dialysis
601 Peritonitis (fungal, with peritoneal dialysis
602 Peritonitis (due to other cause, with peritoneal dialysis
888 Cod not requested

Table A HLA mismatch groups

Level	HLA mismatch summary
1	0
2	[0 DR and 0/1 B]
3	[0 DR and 2 B] or [1 DR and 0/1 B]
4	[1 DR and 2 B] or [2 DR]

PANCREAS/RENAL DISEASE

200 Chronic renal failure, aetiology uncertain
210 Glomerulonephritis, histologically not examined
211 Severe nephrotic syndrome with focal sclerosis
212 IgA nephropathy
213 Dense deposit disease
214 Membranous nephropathy
215 Membrano - proliferative glomerulonephritis
216 Rapidly progressive GN without systemic disease
217 Focal segmental glomerulosclerosis with nephrotic syndrome in adults
219 Glomerulonephritis, histologically examined
220 Pyelonephritis/Interstitial nephritis - cause not specified
221 Pyelonephritis/Interstitial nephritis associated with neurogenic bladder
222 Pyelonephritis/Interstitial nephritis due to con obs uropathy with/without V-U reflux
223 Pyelonephritis/Interstitial nephritis due to acquired obstructive uropathy
224 Pyelonephritis/Interstitial nephritis due to V-U reflux without obstruction
225 Pyelonephritis/Interstitial nephritis due to urolithiasis
229 Pyelonephritis/Interstitial nephritis due to other cause
230 Tubulo Interstitial Nephritis (Not Pyelonephritis)
231 Nephropathy due to analgesic drugs
232 Nephropathy due to cis-platinum
233 Nephropathy due to cyclosporin A
239 Nephropathy caused by other specific drug
240 Cystic kidney disease - type unspecified

241 Polycystic kidneys, adult type (dominant type)
242 Polycystic kidneys, infantile (recessive type)
243 Medullary cystic disease, including nephronophthisis
249 Cystic kidney disease - other specified type
250 Hereditary/Familial nephropathy - type unspecified
251 Hereditary nephritis with nerve deafness (Alports syndrome)
252 Cystinosis
253 Primary oxalosis
254 "Fabrys disease"
259 Hereditary nephropathy - other
260 Congenital renal hypoplasia - type unspecified
261 Oligomeganephronic hypoplasia
262 Segmental renal hypoplasia
263 Congenital renal dysplasia with or without urinary tract malformation
266 Syndrome of agenesis of abdominal muscles
270 Renal vascular disease - type unspecified
271 Renal vascular disease - malignant hypertension
272 Renal vascular disease - hypertension
273 Renal vascular disease - polyarteritis
274 "Wegeners granulomatosis"
275 Ischaemic renal disease/ cholesterol embolism
279 Renal vascular disease - classified
280 Diabetes - insulin dependent (Type I)
281 Diabetes - non-insulin dependent (Type II)
282 Myelomatosis/Light chain deposit disease
283 Amyloid
284 Lupus erythematosus
285 Henoch-Schonlein purpura
286 "Goodpastures Syndrome"
287 Systemic sclerosis (Scleroderma)
288 Haemolytic Uraemic Syndrome (inc Moschowitz Syndrome)
289 Multi-system disease - other
290 Cortical or tubular necrosis
291 Tuberculosis
292 Gout
293 Nephrocalcinosis & hypercalcaemic nephropathy
294 Balkan nephropathy
295 Kidney tumour
296 Traumatic or surgical loss of kidney
298 Other identified renal disorders
299 Unknown
888 Not reported

PANCREAS CAUSE OF FAILURE

0 Graft still functioning
201 Vascular thrombosis
202 Infection
203 Bleeding
204 Anastomotic leak
205 Pancreatitis
206 Primary non-function (no detectable C-peptide between 7 and 28 days)
207 Patient declined medication
208 Hyperacute rejection

209 Acute rejection
210 Chronic rejection
211 Preservation/Procurement problem
212 Instant blood mediated inflammatory reaction (IBMIR)
213 Poor engraftment
214 Functional decline (unknown cause)
215 Recurrent autoimmunity
216 Hepatitis / Steatosis
217 Cholangitis
290 Death with a functioning graft
298 Other
299 Unknown

RELATIONSHIP

0 Son or daughter
1 Cadaveric donor, no relationship
2 Mother or father
3 Sibling (2 shared haplotypes)
4 Sibling (1 shared haplotype)
5 Sibling (0 shared haplotype)
6 Sibling (genetic relationship unknown)
7 Monozygotic (identical) twin
8 Dizygotic (non-identical) twin
9 Other living related - please specify
10 Obligatory removal of organ
11 Living non-related donor - spouse
12 Living non-related donor - partner
15 Pooled donor
16 Altruistic non-directed donor
19 Other living non-related donor - please specify
20 Living donor, relationship unspecified
88 Missing
99 Unknown
103 Default relationship - update required

PERFUSATE USED

0 Not known
1 Sacks
2 Perfudex
3 Dextran 40
4 Gelin
6 Hartmans
7 Manitol
8 Rheomacrodex
10 Euro collins
20 Wisconson/u (UW solution)
21 U.W.(commercial)
22 Modified U.W.(high Na+)
23 Modified U.W.(high k+)
27 Other liver preservation fluid
28 Not reported
29 Unknown

30 Marshalls/hoc
40 PBS
50 St thomas
60 Papworth solution
66 Not perfused/not applicable
70 Machine preservation - PPF
79 Machine preservation - other
88 Not reported
95 Other
98 Other
99 Unknown

RENAL CAUSES OF FAILURE

0 Graft still functioning
201 Hyperacute rejection
202 Rejection while taking immunosuppressive drug(s)
203 Rejection after stopping all immunosuppressive drugs
204 Recurrent primary renal disease
205 Vascular or ureteric operative problems (excluding vascular thrombosis)
206 Vascular (arterial or venous) thrombosis
207 Infection of graft
208 Removal of functioning graft
209 Non-viable kidney
290 Recipient died, graft was functioning at time of death
295,298 Other
299 Unknown

CARDIO PRIMARY DISEASE

310 Coronary Heart Disease
311 Dilated Cardiomyopathy - type unspecified
313 Valvular Heart Disease
314 Congenital Heart Disease
319 Other Heart Disease
320 Primary Pulmonary Hypertension
321 Eisenmenger's Syndrome - type unspecified
322 Cystic Fibrosis
323 Fibrosing Lung Disease
324 Inhalation
325 Alpha-1-Antitrypsin deficiency
326 Sarcoid
327 Emphysema
328 Bronchiectasis
330 Immediate Graft Failure
331 Acute Rejection
332 Coronary Occlusive Disease
333 Hyper acute rejection
334 Chronic rejection
335 Non-specific graft failure
337 Bronchiolitis obliterans
338 Allograft vascular disease
341 Dilated Cardiomyopathy - idiopathic
342 Dilated Cardiomyopathy - familial

343 Dilated Cardiomyopathy - myocarditis
344 Dilated Cardiomyopathy - alcohol
345 Dilated Cardiomyopathy - post partum
346 Dilated Cardiomyopathy - adriamycin
349 Dilated Cardiomyopathy - other, specify
350 Hypertrophic Cardiomyopathy
351 Restrictive Cardiomyopathy
361 Eisenmenger's Syndrome - ASD
362 Eisenmenger's Syndrome - VSD
363 Eisenmenger's Syndrome - PDA
364 Eisenmenger's Syndrome - other
370 Other Congenital Heart/Lung Disease
398 Other
399 Unknown
888 Not reported

CARDIOTHORACIC CAUSES OF FAILURE

0 Graft still functioning
310 Rejection
311 Early allograft dysfunction
312 Acute rejection
320 Infection
330 Coronary occlusive disease
331 Obliterative bronchiolitis
390 Recipient died, graft was functioning at time of death
395 Other identified cause of death
398 Other
399 Unknown

LIVER CAUSES OF FAILURE

0 GRAFT STILL FUNCTIONING
410 ACUTE REJECTION
420 CHRONIC REJECTION
430 PRIMARY NON-FUNCTION
440 ACUTE VASCULAR OCCLUSION
441 VASCULAR OCCLUSION
450 NON-THROMBOTIC INFARCTION
460 DUCTOPENIC REJECTION
470 RECURRENT DISEASE
480 BILIARY COMPLICATIONS
490 RECIP. DIED, GRAFT STILL FUNCTIONING AT T.O.D.
495 OTHER
498 Other, please specify
499 UNKNOWN

LIVER PRIMARY DISEASE

400 Liver disease unknown
410 Chronic liver failure cause unknown
411 Primary biliary cirrhosis
412 Autoimmune chronic active liver disease
413 Hepatitis B cirrhosis

For peer review only

414 Primary sclerosing cholangitis
415 Alpha-1-antitrypsin deficiency
416 Budd-Chiari syndrome (not code 27)
417 Cryptogenic cirrhosis
418 Secondary biliary cirrhosis
419 Alcoholic liver disease
420 Biliary atresia
421 Congenital hepatic fibrosis
422 Wilson's Disease
423 Congenital biliary disease
424 Hepatitis C cirrhosis
425 Paediatric cholestatic liver disease, please specify
426 Non-alcoholic fatty liver disease
427 Acute hepatic failure - Budd-Chiari syndrome
428 Acute hepatic failure - other virus
430 Acute hepatic failure - serologically indeterminate
431 FHF - Viral
432 FHF - Drug induced
433 FHF - Toxin induced
434 Acute hepatic failure - Wilson's disease
435 Acute hepatic failure - HAV
436 Acute hepatic failure - HBV
437 Acute Hepatic Failure - paracetamol hepatotoxicity
438 Acute Hepatic Failure - other drug toxicity
439 Acute Hepatic Failure - other, please specify
440 Malignancy (CA) not classifiable
441 Hepatocellular carcinoma - non-cirrhotic
442 Hepatocellular carcinoma - cirrhotic
443 Cholangiocarcinoma
444 Hepatoblastoma
445 Secondary hepatic malignancy
446 Other mesenchymal tumours
447 Other primary hepatic malignancy, please specify
448 Benign liver tumour
450 Other metabolic liver disease, please specify
451 Cystic fibrosis
452 Primary oxalosis
453 Graft versus host disease
454 Hyperlipidaemia
455 Trauma
456 Tyrosinaemia
457 Familial amyloidosis
458 Familial cholestasis
459 Neonatal hepatitis
460 Polycystic liver disease
461 Hereditary haemochromatosis
462 Glycogen storage disease
463 Progressive familial intrahepatic cholestasis
464 Neonatal haemochromatosis
465 Niemann-Pick type C
466 Hypercholesterolaemia
467 Urea cycle disorder
468 Crigler-Najjar syndrome

For peer review only

469 Cholesterol ester storage disease
470 Alper's syndrome
471 Acute rejection
472 Chronic rejection
473 Primary non-function
474 Acute vascular occlusion (artery plus vein)
475 Non-thrombotic infarction
476 Ductopenic rejection
477 Recurrent disease
478 Biliary complications
479 Hepatic artery thrombosis
480 Early graft dysfunction
481 Acute vascular occlusion - venous
482 Acute vascular occlusion - artery and venous
483 Mitochondrial disease
484 Alagilles syndrome
485 Neonatal sclerosing cholangitis
486 Other neonatal cholestasis
498 Other, please specify
499 Unknown
888 Not reported

OCULAR TISSUE RECIPIENT DIAGNOSIS

110 KERATOCONUS
111 KERATOGLOBUS
117 OTHER ECTASIAS
120 FUCHS' DYSTROPHY
127 OTHER DYSTROPHIES
131 PSEUDOPHAKIC CORNEAL OEDEMA
132 APHAKIC CORNEAL OEDEMA
133 COMPLICATIONS OF REFRACTIVE SURGERY
137 OTHER PREVIOUS OCULAR SURGERY
141 VIRAL INFECTION
142 BACTERIAL INFECTION
143 FUNGAL INFECTION
144 PROTOZOAN INFECTION
147 OTHER INFECTION
151 MECHANICAL INJURY
152 CHEMICAL INJURY
157 OTHER INJURY
163 RHEUMATOID ARTHRITIS
167 OTHER ULCERATIVE KERATITIS
171 REGRAFT - GRAFT FAILURE
172 REGRAFT - REJECTION
173 REGRAFT - ENDOTHELIAL FAILURE
174 REGRAFT - HIGH ASTIGMATISM
176 REGRAFT - ENDOPHTHALMITIS
177 REGRAFT - OTHER CAUSE OF FAILURE
181 INTERSTITIAL KERATITIS
182 DERMOID OPACIFICATION
183 NEOPLASIA
184 PTERYGIUM
187 OTHER OPACIFICATION

191 ADULT APHAKIA
192 PAEDIATRIC APHAKIA
193 PRIMARY ORBITAL IMPLANT
194 SECONDARY ORBITAL IMPLANT
195 REPAIR OF LOCALISED SCLERAL DEFECT
198 OTHER
199 NOT REPORTED

For peer review only

r review only

r review only

HLA mismatch combinations included
0
100, 010, 110, 200, 210
020, 120, 220, 001, 101, 201, 011, 111, 211
021, 121, 221, 002, 102, 202, 012, 112, 212, 022, 122,

view only

Public Health England

Protecting and improving the nation's health

NCRAS Cancer Registration Da

Access to cancer registration data

The NCRAS data dictionary contains attribute lists that are c
for Data Release (ODR). Prospective applicants are advisec
To access the support available through the NCRAS analyti

Data availability

The following datasets are available for request:

Tables
Cancer registration (patient table)
Cancer registration (tumour table)
Cancer registration (treatment table)
Index of Multiple Deprivation_Income domain
Route to diagnosis
National Radiotherapy Dataset (RTDS)
Systemic Anti-Cancer Therapy Dataset
HES admitted care
HES outpatient
HES accident and emergency
NBOCAP
Cancer Patient Experience Survey
Cancer Waiting Times
Quality of Life of Cancer Survivors in England: (Breast, Colorectal, Prostate, Non-Hodgkin's Lymphoma)
Lung Cancer Data Audit (LUCADA)
Quality of Life of Colorectal Cancer Survivors in England: Patient Reported Outcome Measures Survey (PROMS)

Selecting data fields

In using these attribute lists, users are advised requests for
the body of the associated protocol will not be disclosed. Ap

- all relevant coding classifications (i.e. ICD 10 C61 and
- all relevant geographies (i.e. All England, patients res
- time period(s) (temporality)

For peer review only

Privacy by design and default

In assessing applications to access PHE data, the ODR wor intended purpose. As part of this approach we have highligh fields. Prospective applicants are encouraged to apply priva in our privacy principles include:

**Aggregation**	**Age bands** will be suppli anonymity checks. **Ethnic categorisation** sl **Geographic data** should
**Partial data removal**	**Dates** (such as date of bi possible to provide event sensitive data.
**Masking**	**Provider, Consultant ar** data to be observed whil
**De-duplication**	Only one **deprivation in** Where similar items are a

The ODR encourages applicants to apply data minimisation have any questions please contact the Data Access and Co

For more information on NCRAS datasets, you can also rea
[PHE cancer data sets, linkage and availability]

First published: June 2018

© Crown copyright 2018
Re-use of Crown copyright material (excluding logos) is allo
www.nationalarchives.gov.uk/doc/open-government-licence/
for terms and conditions.

For peer review only - http://bmjopen.bmj.com/site/about/guidelines.xhtml

For peer review only - http://bmjopen.bmj.com/site/about/guidelines.xhtml

For peer review only - http://bmjopen.bmj.com/site/about/guidelines.xhtml

ly

For peer review only - http://bmjopen.bmj.com/site/about/guidelines.xhtml

ly

For peer review only - http://bmjopen.bmj.com/site/about/guidelines.xhtml

ly

ly

For peer review only - http://bmjopen.bmj.com/site/about/guidelines.xhtml

For peer review only - http://bmjopen.bmj.com/site/about/guidelines.xhtml

For peer review only - http://bmjopen.bmj.com/site/about/guidelines.xhtml

r peer review only

ata Dictionary v3.4

designed to support the co-production of a data specification for a specific project, ahead of submis
d to seek expert support from a site, geographic or asset specific analyst to confirm and validate the
cal network, please contact ODR@phe.gov.uk

Coverage	Available as a discrete dataset (not linked)
1 January 1985 - 31 December 2016	x
1 January 1985 - 31 December 2016	x
1 January 1985 - 31 December 2016	x
To latest available date	
1 January 2006 - 31 December 2015	
1 April 2009 - 31 March 2016	
1 April 2012 - 30 June 2016	
1 April 1997 - 31 December 2015	*Please contact the NHS Digital Data Access Request Service (DARs)*
1 April 1997 - 31 December 2015	
1 April 1997 - 31 December 2015	
1 April 2006 - 31 March 2013	
Please see relevant sheet regarding each wave of the survey	*Please contact Quality Health*
1 January 2009 - 31 December 2014	*Please contact NHS England*
July 2006 - July 2010	x
1 January 2005 - 31st December 2013	x
1 January 2010 - 31 December 2011	x

data must be limited to the minimum necessary to accomplish the intended purpose. Fields that fai
plicants are further advised to summarise the following requirements in the 'Request Summary' tab

l D07)
siding within CCG, patients treated at NHS Trust)

ːk to 'Privacy by Design and Default' principles to ensure that any data released is the minimum ne
ıted items in this data dictionary where these principles have been applied, including a number of c
cy by design and default practices when identifying the data required for the project. Examples of t

ied instead of precise age. These bandings may be in the standard 5yr banding or broader groups

hould be as broad as possible, full ethnic categorisation breakdown often causes data to fail k-anoı
ǀ be at the highest level to allow analysis to be undertaken.

irth) will, by default, be supplied as month and year only. Precise dates will require additional justifi
t intervals, please consider if this would allow the intended analysis to be undertaken without the ne

ıd GP Practice codes data will be pseudonymised by default. The aim of this measure is to allow
lst ensuring there is no reputational risk for organisations or individuals identified within the data

dex will only be supplied, multiple indices allow the identification of the geographies to which the in
available in multiple data sources, by default, **data will only be supplied from a single source**.

techniques for your proposed project, to ensure your application can be processed as quickly as p
nfidentiality Manager assigned to your application or the ODR directly at ODR@phe.gov.uk

d the 'PHE cancer data sets, linkage and availability' document.

wed under the terms of the Open Government Licence, visit
/version/3/

For peer review only - http://bmjopen.bmj.com/site/about/guidelines.xhtml

For peer review only - http://bmjopen.bmj.com/site/about/guidelines.xhtml

r peer review only

ssion to the PHE Office
eir data requirements.

Available as linked resource only
x
x
x
x
x
x
x
x
x
x

il to be justified within
):

cessary for the
commonly derived
he data items included

if required to pass k-<br>nymity checks.
ication. It may be<br>eed to release
variation within the
dices apply.

ossible. Should you

r peer review only

r peer review only

For peer review only

Provide a summary of the data required details below - and then select items you require in the appropriate workshe

All applicants must select and justify each data item required as part of the ODR request application.

Summary of request - Please provide a summary of the data being requested, outlining which of the available c

We are requesting data from 'CANCER REG-PATIENT TABLE', 'CANCER REG-TUMOUR TABLE', 'CANCER REG-TREATMENT TABLE', SACT and RTDS. The entire cohort is 1 Jan 1985 to 31 Dec 2016, with data required up to latest available. We understand that some cohorts (especially SACT and RTDS) do not encompass this entire period but we are requesting everything that is available.

Cancer Sites/Morphologies – Please provide the cancer sites and/or morphologies required for the request separated by commas and the coding system used. If combinations of site/ morphology are required please separate site and morphology with hyphens. If all codes within a tumour site grouping are required an 'x' may be used to suffix the 3 character grouping. (For example: C18x, C19x, C20x, C44 – 80903, C44 – 81703, C56.1, C56.2)

All cancer sites and morphologies requested.

Geography or treatment provider criteria – Please provide us with the geography for the data provided, if data are required for all of England please state this. If data are required for particular geographies/provider please state the geography level, the required geographies and how these geographies should be applied to the data. (For example: CCGs 07X, 08V, 08B defined by patient treatment within trust located in one of these CCGs)

We require data for all of England.

Time period criteria - individual years or a range of years. Time period should also describe which dataset time period applies to, e.g. all patients with a diagnosis date between 2000-2010 or patients with any inpatient HES activity in the trusts defined above in 2015. Please also indicate clearly if the date is diagnostic date, treatment date, event date or a

All patients with any diagnosis between 1st January 1985 and 31st December 2017

For peer review only

Introduction

To achieve comprehensive registration for all registerable tumours, the NCRA
treatment from diagnosis. The CAS AV 2016 tables includes data on the patie

Data item selection

Please be aware that selecting patient identifiable data items – **the data item**
statutory regulation covering your organisation. Sensitive patient demographic
applied to render the data anonymised to k-3 anonymity. Privacy by design ar
key explaining how these alternative can be selected is included below:

Key:

**Items in red**	Identifiable fields that require additional permissions to access
	Sensitive items that require additional justification in your protoc
	Privacy by design and default' alternatives to the sensitive items

Item number	Data item
1	Pseudonymised patient ID
2	**NHS number**
3	Alias check flag - patient
4	**Date of Birth**
5	Month of birth
6	Year of birth
7	Date of Birth check flag - patient
8	Sex
9	Ethnicity
10	Ethnic group
11	Broad ethnic group
12	Vital status of the patient
13	**Date of death of the patient**
14	Month of death of the patient
15	Year of death of the patient
16	Days from another event to date to death
17	Date of death imputed flag
18	Embarkation flag
19	Date of embarkation
20	Month of embarkation
21	Year of embarkation
22	Days from another event to embarkation
23	As provided with death notification
24	As provided with death notification

**25**	As provided with death notification
**26**	As provided with death notification
**27**	As provided with death notification
**28**	Code of the location (type) where the patient died, e.g. patients home, hospice etc.
**29**	Description of the location (type) where the patient died, e.g. patients home, hospice etc.
**30**	Code of institution at which death takes place
**31**	Indicates whether a post-mortem took place
**32**	Count of every tumour assigned to this PatientID.
**33**	Count of every tumour assigned to this PatientID in range C00-97 excl C44

************************ **End of sheet** ***********************

For peer review only

AS brings together data from more than 500 local and regional datasets to build a picture of an individual's ent, their diagnosis, tumour and treatment events.

s marked below in red – will require you to have patient consent, section 251 approval, or the appropriate c data, event dates and geographies will be treated as de-personalised, unless anonymisation techniques are nd default principles have also been applied to some of these data to provide a less disclosive alternative. The

(as outlined above)
col to access
s highlighted in dark grey

Field name	Request data item	Justification
**Av_patient**		
PATIENTID	**X**	This will be done by NHS Digital
**NHSNUMBER**	**X**	Necessary for record linkage by NHS Digital
ALIASFLAG		
**BIRTHDATEBEST**	X	Necessary for record linkage by NHS Digital
MONTH_DOB		
YEAR_DOB		
BIRTHDATEFLAG		
SEX	X	Necessary for record linkage by NHS Digital but also to investigate cancer risk by baseline demographics.
ETHNICITY	X	To investigate cancer risk by baseline demographics.
ETHNICITYNAME	X	To investigate cancer risk by baseline demographics.
Option to group ethnicities (e.g. white/ non-white/ unknown)		
VITALSTATUS		
**DEATHDATEBEST**	X	To investigate cancer mortality risk.
MONTH_DOD		
YEAR_DOD		
Option to provide number of days from another event to death (e.g. days from diagnosis to death)	X	To investigate days from first diagnosis to death.
DEATHDATEFLAG		
EMBARKATION		
EMBARKATIONDATE		
Month of embarkation		
Year of embarkation		
Option to provide number of days from another event to embarkation (e.g. days from diagnosis to embarkation		
DEATHCAUSECODE_1A	X	To investigate cancer mortality aetiology (cancer versus other causes)
DEATHCAUSECODE_1B	X	To investigate cancer mortality aetiology (cancer versus other causes)

DEATHCAUSECODE_1C	X	To investigate cancer mortality aetiology (cancer versus other causes)
DEATHCAUSECODE_2	X	To investigate cancer mortality aetiology (cancer versus other causes)
DEATHCAUSECODE_UNDERLYING	X	To investigate cancer mortality aetiology (cancer versus other causes)
DEATHLOCATIONCODE		
DEATHLOCATIONDESC		
SITECODEOFDEATH		
POSTMORTEM		
TUMOURCOUNT	X	To understand overall cancer burden.
BIGTUMOURCOUNT	X	To understand overall cancer burden.

For peer review only

view only

Introduction

To achieve comprehensive registration for all registerable tumours, the NCRS brings tog
diagnosis. The CAS AV 2016 includes data on the patient, their diagnosis, tumour and tr

Data item selection

Please be aware that selecting patient identifiable data items – **the data items marked** regulation covering your organisation. Sensitive patient demographic data, event dates a anonymised to k-3 anonymity. Privacy by design and default principles have also been a be selected is included below:

Key:

**Items in red**	Identifiable fields that require additional permissions to access (as outline
	Sensitive items that require additional justification in your protocol to acce
	Privacy by design and default' alternatives to the sensitive items highlight

Item number	Data item
1	Pseudonymised tumour ID
2	Pseudonymised patient ID
3	**ONS ID**
4	**NHS Number**
5	**Date of Birth**
6	Month of birth
7	Year of birth
8	Age at diagnosis
9	Age at diagnosis in 5 year age bands (0-4 etc.)
10	Sex
11	**Postcode at Diagnosis**
12	Broader geographic area/ IMD quintile
13	Ethnicity
14	Broad ethnic group
15	Earliest date when the diagnosis may have taken place
16	Latest date when the diagnosis may have taken place
17	Diagnosis date
18	Month of diagnosis
19	Year of diagnosis
20	Days from another event to date to diagnosis
21	Date of diagnosis imputed flag
22	Financial year of diagnosis
23	Basis of diagnosis of the tumour
24	Diagnosis death certificate only
25	Site of neoplasm (4-character ICD-10-O2 code)
26	Site of neoplasm (3-character ICD-10-O2 code)
27	Site of the cancer
28	Description of the code in SITE_CODED
29	3 digit version of SITE_CODED
30	The coding system used to register the tumour

**31**	Description of coding system used in registration
**32**	Morphology
**33**	Morphology of the cancer, in the ICD-10-O2 system
**34**	Behaviour of the cancer, in the ICD-10-O2 system
**35**	Numeric behaviour code
**36**	Description of behaviour code
**37**	Histology code
**38**	Description of histology code
**39**	Grade of tumour
**40**	Size of the largest dimension of the tumour, in mm
**41**	Number of nodes excised
**42**	Number of nodes involved
**43**	Laterality
**44**	Multifocal
**45**	Oestrogen receptor status of the tumour
**46**	Oestrogen receptor score of the tumour.
**47**	Progesterone receptor status of the tumour
**48**	Progesterone receptor score of the tumour
**49**	HER2 status of the tumour
**50**	Nottingham Prognostic Index Score
**51**	Dukes' stage
**52**	FIGO stage
**53**	Clark's stage
**54**	Breslow thickness of tumour
**55**	Gleason primary pattern
**56**	Gleason secondary pattern
**57**	Gleason tertiary pattern
**58**	Combined Gleason primary and secondary scores
**59**	T stage (pre-treatment)
**60**	N stage (pre-treatment)
**61**	M stage (pre-treatment)
**62**	Stage at diagnosis derived from imaging
**63**	System used to record imaging stage at diagnosis
**64**	T stage (pathology)
**65**	N stage (pathology)
**66**	M stage (pathology)
**67**	Pathological stage at diagnosis
**68**	System used to record pathological stage at diagnosis
**69**	Pathological stage at diagnosis (pre-treatment)
**70**	T stage flagged by the registry as the 'best' T stage
**71**	N stage flagged by the registry as the 'best' N stage
**72**	M stage flagged by the registry as the 'best' M stage
**73**	Best 'registry' stage at diagnosis of the tumour
**74**	System used to record best registry stage at diagnosis
**75**	Code for the place where the diagnosis episode took place
**76**	Pseudonymised diagnosis provider code
**77**	Description of DIAGNOSISPROVIDER_CODE
**78**	Code for the Trust at diagnosis
**79**	Pseudonymised diagnosis trust code
**80**	Name of the trust at diagnosis
**81**	Tumour registration status
**82**	Excision margin
**83**	Screen detected cancer
**84**	Screening status of the tumour
**85**	Description of SCREENINGSTATUSCOSD_CODE
**86**	Full detailed screening status of the tumour
**87**	Description of SCREENINGSTATUSFULL_CODE
**88**	Date of first recorded event in treatment table
**89**	Month of first recorded event in treatment table
**90**	Year of first recorded event in treatment table

For peer review only

**91**	Days from another event to first recorded event
**92**	Trust code of first recorded event in treatment table
**93**	Pseudonymised trust code of first event
**94**	Name of trust for first recorded event in treatment table
**95**	Date of first recorded surgery in treatment table
**96**	Month of first recorded surgery in treatment table
**97**	Year of first recorded surgery in treatment table
**98**	Days from another event to first recorded surgery in treatment table
**99**	Trust code of first recorded surgery in treatment table
**100**	Pseudonymised trust code of first recorded surgery
**101**	Name of trust for first recorded surgery in treatment table
**102**	2011 Lower Super Output Area
**103**	2001 Lower Super Output Area
**104**	2011 Middle Super Output Area
**105**	2001 Middle Super Output Area
**106**	Clinical Commissioning Group code (at diagnosis)
**107**	Name of the Clinical Commissioning Group
**108**	Primary Care Trust code the patient was resident in when the tumour was diagnosed
**109**	Name of the Primary Care Trust the patient was resident in when the tumour was diagnosed
**110**	Local Authority Unitary Authority code the patient was resident in when the tumour was diagnosed
**111**	Name of the Local Authority Unitary Authority the patient was resident in when the tumour was diagnosed
**112**	Upper tier Local Authority code the patient was resident in when the tumour was diagnosed
**113**	Name of the upper tier Local Authority the patient was resident in when the tumour was diagnosed
**114**	Strategic Clinical Network code the patient was resident in when the tumour was diagnosed
**115**	Name of the Strategic Clinical Network the patient was resident in when the tumour was diagnosed
**116**	Cancer network code the patient was resident in when the tumour was diagnosed
**117**	Name of the cancer network the patient was resident in when the tumour was diagnosed
**118**	County code the patient was resident in when the tumour was diagnosed
**119**	Name of the county the patient was resident in when the tumour was diagnosed
**120**	Government office region code the patient was resident in when the tumour was diagnosed
**121**	Name of the government office region the patient was resident in when the tumour was diagnosed
**122**	Cancer registry catchment area code the patient was resident in when the tumour was diagnosed
**123**	Name of the cancer registry catchment area the patient was resident in when the tumour was diagnosed
**124**	Country code the patient was resident in when the tumour was diagnosed
**125**	Name of the country the patient was resident in when the tumour was diagnosed
**126**	Cancer registry code which finalised the case and was responsible for sending it to ONS if it was an in-region case

For peer review only

**127**	Name of the cancer registry which finalised the case and was responsible for sending it to ONS if it was an in-region case

************************* **End of sheet** *************************

For peer review only

jether data from more than 500 local and regional datasets to build a picture of an individual's treatment from reatment events.

below in red – will require you to have patient consent, section 251 approval, or the appropriate statutory and geographies will be treated as de-personalised, unless anonymisation techniques are applied to render the data applied to some of these data to provide a less disclosive alternative. The key explaining how these alternative can

ed above)
ess
ed in dark grey

Field name	Request data item	Justification
**Av_tumour**		
TUMOURID		
PATIENTID		
**ONSID**		
**NHSNUMBER**	X	Necessary for record linkage by NHS Digital
**BIRTHDATEBEST**	X	Necessary for record linkage by NHS Digital
MONTH_DOB		
YEAR_DOB		
AGE	X	To investigate cancer risk by baseline demographics.
FIVEYEARAGEBAND	X	To investigate cancer risk by baseline demographics.
SEX	X	Necessary for record linkage by NHS Digital but also to investigate cancer risk by baseline demographics.
**POSTCODE**	X	Necessary for record linkage by NHS Digital
Option to provide geography as deprivation score or aggregate to larger geographic areas such as MSOA or county.	X	IMD quintile to investigate cancer risk b
ETHNICITY	X	To investigate cancer risk by baseline demographics.
Option to group ethnicities (e.g. white/ non-white/ unknown)		
DIAGNOSISDATE1	X	For time survival analyses and determir
DIAGNOSISDATE2	X	For time survival analyses and determir
DIAGNOSISDATEBEST	X	For time survival analyses and determir
DIAGNOSISMONTH		
DIAGNOSISYEAR		
Option to provide number of days from another event to diagnosis (e.g. days from birth to diagnosis)	X	From birth to date of diagnosis
DIAGNOSISDATEFLAG		
FINANCIALYEAR		
BASISOFDIAGNOSIS		
DCO		
SITE_ICD10_O2	X	To better understand cancer morpholog
SITE_ICD10_O2_3CHAR	X	To better understand cancer morpholog
SITE_CODED	X	To better understand cancer morpholog
SITE_CODED_DESC	X	To better understand cancer morpholog
SITE_CODED_3CHAR	X	To better understand cancer morpholog
CODING_SYSTEM	X	To better understand cancer morpholog

CODING_SYSTEM_DESC	X	To better understand cancer morpholog
MORPH_CODED	X	To better understand cancer morpholog
MORPH_ICD10_O2	X	To better understand cancer morpholog
BEHAVIOUR_ICD10_O2	X	To better understand cancer morpholog
BEHAVIOUR_CODED	X	To better understand cancer morpholog
BEHAVIOUR_CODED_DESC	X	To better understand cancer morpholog
HISTOLOGY_CODED	X	To better understand cancer morpholog
HISTOLOGY_CODED_DESC	X	To better understand cancer morpholog
GRADE	X	To better understand cancer morpholog
TUMOURSIZE	X	To better understand cancer morpholog
Nodes_excised_new	X	To better understand cancer morpholog
nodes_involved_new	X	To better understand cancer morpholog
LATERALITY	X	To better understand cancer morpholog
MULTIFOCAL	X	To better understand cancer morpholog
ER_STATUS	X	To better understand cancer morpholog
ER_SCORE	X	To better understand cancer morpholog
PR_STATUS	X	To better understand cancer morpholog
PR_SCORE	X	To better understand cancer morpholog
HER2_STATUS	X	To better understand cancer morpholog
NPI	X	To better understand cancer morpholog
DUKES	X	To better understand cancer morpholog
FIGO	X	To better understand cancer morpholog
CLARKS	X	To better understand cancer morpholog
BRESLOW	X	To better understand cancer morpholog
GLEASON_PRIMARY	X	To better understand cancer morpholog
GLEASON_SECONDARY	X	To better understand cancer morpholog
GLEASON_TERTIARY	X	To better understand cancer morpholog
GLEASON_COMBINED	X	To better understand cancer morpholog
T_IMG	X	To better understand cancer morpholog
N_IMG	X	To better understand cancer morpholog
M_IMG	X	To better understand cancer morpholog
STAGE_IMG	X	To better understand cancer morpholog
STAGE_IMG_SYSTEM	X	To better understand cancer morpholog
T_PATH	X	To better understand cancer morpholog
N_PATH	X	To better understand cancer morpholog
M_PATH	X	To better understand cancer morpholog
STAGE_PATH	X	To better understand cancer morpholog
STAGE_PATH_SYSTEM	X	To better understand cancer morpholog
STAGE_PATH_PRETREATED	X	To better understand cancer morpholog
T_BEST	X	To better understand cancer morpholog
N_BEST	X	To better understand cancer morpholog
M_BEST	X	To better understand cancer morpholog
STAGE_BEST	X	To better understand cancer morpholog
STAGE_BEST_SYSTEM	X	To better understand cancer morpholog
DIAGNOSISPROVIDER_CODE		
DIAGNOSISPROVIDER_CODE (pseudonymised)		
DIAGNOSISPROVIDER_NAME		
DIAGNOSISTRUST_CODE	X	To understand cancer burden and deliv
DIAGNOSISTRUST_CODE (pseudonymised)		
DIAGNOSISTRUST_NAME	X	To understand cancer burden and deliv
STATUSOFREGISTRATION	X	To better understand cancer status
EXCISIONMARGIN	X	To better understand cancer status
SCREENDETECTED	X	To understand screening success for p
SCREENINGSTATUSCOSD_CODE	X	To understand screening success for p
SCREENINGSTATUSCOSD_NAME	X	To understand screening success for p
SCREENINGSTATUSFULL_CODE	X	To understand screening success for p
SCREENINGSTATUSFULL_NAME	X	To understand screening success for p
DATE_FIRST_EVENT	X	To understand cancer burden and deliv
Month of first recorded event in treatment table	X	To understand cancer burden and deliv
Year of first recorded event in treatment table	X	To understand cancer burden and deliv

Option to provide number of days from another event to the first recorded event in the treatment table (e.g. days from diagnosis to first treatment event)	X	Days from diagnosis to first treatment e
TRUSTCODE_FIRST_EVENT	X	To understand cancer burden and deliv
TRUSTNAME_FIRST_EVENT	X	To understand cancer burden and deliv
DATE_FIRST_SURGERY	X	To better understand cancer treatment
Month of first recorded surgery in treatment table	X	To better understand cancer treatment
Year of first recorded surgery in treatment table	X	To better understand cancer treatment
Option to provide number of days from another event to the first recorded surgery (e.g. days from diagnosis to first recorded surgery)	X	Days from diagnosis to first recorded su
TRUSTCODE_FIRST_SURGERY	X	To understand cancer burden and deliv
TRUSTCODE_FIRST_SURGERY (pseudonymised)		
TRUSTNAME_FIRST_SURGERY	X	To understand cancer burden and deliv
LSOA11_CODE		
LSOA01_CODE		
MSOA11_CODE		
MSOA01_CODE		
CCG_CODE		
CCG_NAME		
PCT_CODE		
PCT_NAME		
LAUA_CODE		
LAUA_NAME		
UTLA_CODE		
UTLA_NAME		
SCN_CODE		
SCN_NAME		
CNET_CODE		
CNET_NAME		
COUNTY_CODE		
COUNTY_NAME		
GOR_CODE		
GOR_NAME		
CREG_CODE		
CREG_NAME		
CTRY_CODE		
CTRY_NAME		
CENTRE		

For peer review only

CENTRENAME		

For peer review only

ɔy baseline demographics

ne time from date of diagnosis from date of transplant
ne time from date of diagnosis from date of transplant
ne time from date of diagnosis from date of transplant

ɡy
ɡy
ɡy
ɡy
ɡy
ɡy

For peer review only

gy
gy
gy
gy
gy
gy
gy
gy
gy
gy
gy
gy
gy
gy
gy
gy
gy
gy
gy
gy
gy
gy
gy
gy
gy
gy
gy
gy
gy
gy
gy
gy
gy
gy
gy
gy
gy
gy
gy
gy
gy
gy

ery of care post-transplant

ery of care post-transplant

ost-transplant cancer detection
ost-transplant cancer detection
ost-transplant cancer detection
ost-transplant cancer detection
ost-transplant cancer detection
ery of care post-transplant
ery of care post-transplant (in case above not available)
ery of care post-transplant (in case above not available)

event
very of care post-transplant

very of care post-transplant

(if above not available)
(if above not available)

urgery
very of care post-transplant

very of care post-transplant

For peer review only

For peer review only

For peer review only

For peer review only

Introduction

To achieve comprehensive registration for all registerable tumours, the NCRS bri
treatment from diagnosis. The CAS AV 2016 includes data on the patient, their di

Data item selection

Please be aware that selecting patient identifiable data items – **the data items m**
statutory regulation covering your organisation. Sensitive patient demographic da
applied to render the data anonymised to k-3 anonymity. Privacy by design and c
key explaining how these alternative can be selected is included below:

Key:

Items in red Identifiable fields that require additional permissions to access (as
Sensitive items that require additional justification in your protocol t
Privacy by design and default' alternatives to the sensitive items hi

Item number	Data item
1	Pseudonymised event ID
2	Pseudonymised patient ID
3	Pseudonymised tumour ID
4	Age at diagnosis
5	Age at diagnosis in 5 year age bands (0-4 etc.)
6	Age at diagnosis in x year age bands
7	Sex
8	Diagnosis date
9	Month of diagnosis
10	Year of diagnosis
11	Days from another event to date to diagnosis
12	Number of tumours affected by this event
13	Type of event code
14	Description of the event
15	Date the event took place
16	Month the event took place
17	Year the event took place
18	Days from another event to this event
19	Treatment provider (organisation code)
20	Pseudonymised treatment provider code
21	Name of the organisation where the event took place
22	Code of the NHS Trust where the event took place
23	Pseudonymised NHS Trust code where the event took place
24	Name of the NHS Trust where the event took place
25	Consultant code
26	Consultant code (pseudonymised by default)
27	Consultant name
28	Cancer registry catchment area code the patient was resident in when the tumour was diagnosed
29	Treatment within 6 months of diagnosis - check flag
30	Treatment six months from date of diagnosis - check flag

**31**	Operations, procedures and interventions (OPCS-4)
**32**	Name of the operations, procedures and interventions
**33**	Radiotherapy code
**34**	Radiotherapy description
**35**	Imaging code – internal coding system
**36**	Description of imaging
**37**	Site on body where imaging occurred
**38**	Size in millimetres of the diameter of a lesion (histology)

************************** **End of sheet** **************************

For peer review only

ings together data from more than 500 local and regional datasets to build a picture of an individual's
iagnosis, tumour and treatment events.

arked below in red – will require you to have patient consent, section 251 approval, or the appropriate
ata, event dates and geographies will be treated as de-personalised, unless anonymisation techniques are
default principles have also been applied to some of these data to provide a less disclosive alternative. The

outlined above)
to access
ghlighted in dark grey

Field name	Request data item	Justification
**AV_treatment**		
EVENTID		
TUMOURID		
PATIENTID		
AGE	X	To investigate cancer risk by baseline demographics.
FIVEYEARAGEBAND	X	To investigate cancer risk by baseline demographics.
Option to provide age in broad categories (e.g. =<45, 46-55, 56-65, >65)		
SEX	X	To investigate cancer risk by baseline demographics.
DIAGNOSISDATEBEST	X	**To determine time span from transplant**
DIAGNOSISMONTH	X	**To determine time span from transplant (in case**
DIAGNOSISYEAR	X	**To determine time span from transplant (in case**
Option to provide number of days from another event to diagnosis (e.g. days from birth to diagnosis)	X	**Days from birth to diagnosis**
NUMBER_OF_TUMOURS	X	To better understand cancer morphology, nature ar
EVENTCODE	X	To better understand cancer morphology, nature ar
EVENTDESC	X	To better understand cancer morphology, nature ar
EVENTDATE	X	To better understand cancer morphology, nature ar
Month of the year the event took place	X	To better understand cancer morphology, nature ar
EVENTYEAR	X	To better understand cancer morphology, nature ar
Option to provide number of days from another recorded event to this event (e.g. days from diagnosis to event)	X	**Days from birth to event**
PROVIDERCODE	X	To understand cancer burden and delivery of care p
PROVIDERCODE (pseudonymised)		
PROVIDERDESC		
TRUST_CODE	X	To understand cancer burden and delivery of care p
TRUST_CODE (pseudonymised)		
TRUST_NAME	X	To understand cancer burden and delivery of care p
PRACTITIONERCODE		
PRACTITIONERCODE (pseudonymised)		
PRACTITIONERDESC		
CREG_CODE		
WITHIN_SIX_MONTHS_FLAG	X	To better understand cancer treatment
SIX_MONTHS_AFTER_FLAG	X	To better understand cancer treatment

OPCS4_CODE	X	To better understand cancer treatment
OPCS4_NAME	X	To better understand cancer treatment
RADIOCODE	X	To better understand cancer treatment
RADIODESC	X	To better understand cancer treatment
IMAGINGCODE	X	To better understand cancer treatment
IMAGINGDESC	X	To better understand cancer treatment
IMAGINGSITE	X	To better understand cancer treatment
LESIONSIZE	X	To better understand cancer treatment

For peer review only

e above not available)
e above not available)

nd burden
nd burden
nd burden
nd burden
nd burden
nd burden

post-transplant

post-transplant

post-transplant

For peer review only

For peer review only

For peer review only

Introduction

The Income Deprivation Domain measures the proportion of the population experiencin[illegible] those that are in work but who have low earnings (and who satisfy the respective mean[illegible]

Data items selection

Please be aware that selecting patient identifiable data items – **the data items marked** covering your organisation. Sensitive patient demographic data, event dates and geogr[illegible] 3 anonymity.

Note: only one deprivation quintile year can be selected for each application to reduce t[illegible]

Item number	Data item
1	Pseudonymised patient ID
2	Pseudonymised tumour ID
3	Measure of deprivation at small area level made up from the income domain in 2004, quintiles are calculated from populations
4	Measure of deprivation at small area level made up from the income domain in 2007, quintiles are calculated from populations
5	Measure of deprivation at small area level made up from the income domain in 2010, quintiles are calculated from populations
6	Measure of deprivation at small area level made up from the income domain in 2015, quintiles are calculated from populations

****

For peer review only

g deprivation relating to low income. The
s tests).

below in red – will require you to have p
raphies will be treated as de-personalised

the risk of deductive disclosure based on

Field name	Request data item
PATIENTID	
TUMOURID	
IMD2004	
IMD2007	
IMD2010	
quintile_2015	X

************* **End of sheet** *****************

For peer review only

definition of low income used includes both those people that are out-of-work, and

ɔatient consent, section 251 approval, or the appropriate statutory regulation
ɬ, unless anonymisation techniques are applied to render the data anonymised to k-

low level geographies.

Justification
IMD quintile to investigate cancer risk by baseline demographics

****

For peer review only

Introduction

Inpatient HES contains data on all inpatient admissions at NHS hospitals in Engla
paid for the care they deliver. HES data is designed to enable secondary use, that

Data items selection

Please be aware that selecting patient identifiable data items – **the data items ma**
appropriate statutory regulation covering your organisation. Sensitive patient dem
anonymisation techniques are applied to render the data anonymised to k-3 anony
provide a less disclosive alternative. The key explaining how these alternative can
registration data.

Key:

Items in red Identifiable fields that require additional permissions to access (as ou
Sensitive items that require additional justification in your protocol to
Privacy by design and default' alternatives to the sensitive items high

Item number	Data item
1	Pseudonymised patient ID
2	Administrative & legal status of patient
3	Administrative category
4	Age at start of episode
5	**Date of birth - patient**
6	Month of birth
7	Year of birth
8	Ethnic category
9	Broad ethnic group
10	**Local patient identifier**
11	**NHS number**
12	**HES patient identifier**
13	Postcode district of patient residence
14	**Postcode of patient residence**
15	Broader geographic area/ IMD quintile
16	Sex of patient
17	Date of admission
18	Month of admission
19	Year of admission
20	Days from another event to admission
21	Date of decision to admit
22	Month of decision to admit
23	Year of decision to admit
24	Days from another event to date of decision to admit
25	Method of admission
26	Source of admission
27	First regular day or night admission
28	Waiting time
29	Class of patient

**30**	Date of discharge
**31**	Month of discharge
**32**	Year of discharge
**33**	Days from another event to date of discharge
**34**	Destination on discharge
**35**	Method of discharge
	**Ep**
**36**	Bed days within the year
**37**	Beginning of spell
**38**	Date episode ended
**39**	Month the episode ended
**40**	Year the episode ended
**41**	Days from another event to date episode ended
**42**	Date episode started
**43**	Month the episode started
**44**	Year the episode started
**45**	Days from another event to date episode started
**46**	Duration of spell
**47**	End of spell
**48**	Episode duration
**49**	Episode order
**50**	Episode type
**51**	Hospital provider spell number (pseudonymised by default)
**52**	All diagnosis codes
**53**	Primary diagnosis (3 characters) (derived)
**54**	Primary diagnosis (4 characters)
**55**	All operative procedure codes
**56**	Date of operations
**57**	Month of operations
**58**	Year of operations
**59**	Days from another event to date of operation
**60**	Operation status code
**61**	Intended management
**62**	Main specialty
**63**	Treatment specialty
	**Health**
**64**	Dominant procedure
**65**	Healthcare resource group (Applied HRG code from 2006-07 onwards)
**66**	NHS-generated HRG code
**67**	NHS-generated HRG code version number
**68**	SUS generated core spell HRG
**69**	SUS generated HRG
**70**	SUS generated HRG version number
**71**	SUS generated spell ID
**72**	Commissioner code
**73**	Commissioner code status

For peer review only - http://bmjopen.bmj.com/site/about/guidelines.xhtml

**74**	Commissioner's regional office
**75**	Commissioner's strategic health authority
**76**	Commissioning serial number
**77**	Health authority where patients GP was registered
**78**	Primary care group
**79**	Primary care trust of responsibility - historic
**80**	Primary care trust of responsibility - current
**81**	Primary care trust area where patient's GP was registered
**82**	Provider code - 5 character
**83**	Pseudonymised provider code - 5 character
**84**	Provider code - 3 character
**85**	Pseudonymised provider code - 3 character
**86**	Provider code - treatment centre
**87**	Site code of treatment
**88**	Pseudonymised site code of treatment
**89**	Provider type
**90**	Regional office area where patient's GP was registered
**91**	Strategic health authority area where patient's GP was registered
**92**	Broader geographical area where patient's GP was registered
**93**	Census output area 2001
**94**	Census output area 2001 (6 character)
**95**	County of residence
**96**	Local Authority district
**97**	Local authority district & current electoral ward
**98**	Electoral ward in 91
**99**	Government office region of residence
**100**	Government office region of treatment
**101**	Health authority of residence
**102**	Health authority of treatment
**103**	Patient's health authority/PCT of residence provide by NHS
**104**	Patient's primary care trust of residence - historic
**105**	Patient's primary care trust of residence - current
**106**	Patients strategic health authority of residence - historic
**107**	Patients strategic health authority of residence - current
**108**	Primary care trust area of treatment
**109**	Region of treatment
**110**	Regional office of residence
**111**	Strategic health authority area of treatment
**112**	Code of GP practice
**113**	Code of GP practice (Pseudonymised by default)
**114**	Consultant code
**115**	Consultant code (pseudonymised by default)
**116**	Code of patient's registered or referring general medical practitioner
**117**	Code of patient's registered or referring general medical practitioner
**118**	Person referring patient
**119**	Referring organisation Code
**120**	Referring organisation Code (pseudonymised)
**121**	Record Identifier (pseudonymised by default)
**122**	Datayear

************************* **End of sheet**

For peer review only

For

For

For

For peer review only - http://bmjopen.bmj.com/site/about/guidelines.xhtml

Fo

Fo

For

Fo

For peer review only - http://bmjopen.bmj.com/site/about/guidelines.xhtml

For peer review only - http://bmjopen.bmj.com/site/about/guidelines.xhtml

For

ınd. This data is collected during a patient's time at hospital and is submitted to allow hospitals to be
: is use for non-clinical purposes, of this administrative data.

ırked below in red – will require you to have patient consent, section 251 approval, or the
ıographic data, event dates and geographies will be treated as de-personalised, unless
ymity. Privacy by design and default principles have also been applied to some of these data to
ı be selected is included below. **HES data will only be supplied as a linked table to cancer**

ıtlined above)
access
ılighted in dark grey

Field name	Request data item	Justification
**Patient**		
PATIENTID		
category		
admincat		
startage		
**dob**		
Month of birth		
Year of birth		
ethnos		
Option to group ethnicities (e.g. white/ non-white/ unknown)		
**lopatid**		
**newnhsno**		
**encrypted_hesid**		
postdist		
**homeadd**		
Option to provide geography as deprivation score or aggregate to larger geographic areas such as MSOA or county.		
sex		
**Admissions**		
admidate		
Month of admission		
Year of admission		
Option to provide number of days from another event to date of admission (e.g. days from diagnosis to admission)		
elecdate		
Month of decision to admit		
Year of decision to admit		
Option to provide number of days from another event to date of decision to admit (e.g. days from decision to admit to admission)		
admimeth		
admisorc		
firstreg		
elecdur		
classpat		

**Discharges**		
disdate		
Month of discharge		
Year of discharge		
Option to provide number of days from another event to date of discharge (e.g. days from admission to discharge)		
disdest		
dismeth		
**pisodes and Spells**		
bedyear		
spelbgin		
epiend		
Month the episode ended		
Year the episode ended		
Option to provide number of days from another event to date episode ended (e.g. days from diagnosis to date episode ended)		
epistart		
Month the episode started		
Year the episode started		
Option to provide number of days from another event to date episode ended (e.g. days from diagnosis to date episode started)		
speldur		
spelend		
epidur		
epiorder		
epitype		
provspno		
**Clinical**		
diag_nn		
diag3_01		
diag_4		
opertn_nn		
opdate_nn		
Month of operations		
Year of operations		
Option to provide number of days from another event to date of operation (e.g. days from admission to date of operation)		
operstat		
intmanig		
mainspef		
tretspef		
**ıcare Resource Groups**		
domproc		
hrg40		
hrgnhs		
hrgnhsvn		
suscorehrg		
sushrg		
sushrgvers		
susspellid		
**Organisation**		
purcode		
purval		

purro		
purstha		
csnum		
gppracha		
pcgcode		
pctcode		
pctcode06		
gpprpct		
procode		
pseudoprocode		
procode3		
pseudoprocode3		
procodet		
sitetret		
pseudositetret		
protype		
gppracro		
gpprstha		
Option to provide a broader geographic area that the patient's GP was registered (e.g. country or country)		
**Geographical**		
oacode		
oacode6		
rescty		
resladst		
resladst_currward		
ward91		
resgor		
gortreat		
resha		
hatreat		
pctnhs		
respct		
respct06		
resstha		
resstha06		
pcttreat		
rotreat		
Resro		
sthatret		
**Practitioner**		
gpprac		
pseudogpprac		
consult		
pseudoconsult		
reggmp		
pseudoreggmp		
referrer		
referorg		
referorg		
**Systems Data**		
epikeyanon		
datayear		

****

For peer review only

For peer review only

Introduction

Outpatient HES contains data on all outpatient appointments at NHS hospitals in En
care they deliver. HES data is designed to enable secondary use, that is use for nor

Data item selection

Please be aware that selecting patient identifiable data items – **the data items marl**
regulation covering your organisation. Sensitive patient demographic data, event da
data anonymised to k-3 anonymity. Privacy by design and default principles have al
alternative can be selected is included below. **HES data will only be supplied as a**

Key:

**Items in red**	Identifiable fields that require additional permissions to access (as ou
	Sensitive items that require additional justification in your protocol to
	Privacy by design and default' alternatives to the sensitive items high

Item number	Data Item
1	Pseudonymised patient ID
2	Administrative category
3	Ethnic category
4	Broad ethnic group
5	**Local patient identifier**
6	**Patient ID - HES generated**
7	Appointment date
8	Month of appointment
9	Year of appointment
10	Days from another event to appointment date
11	Attendance identifier
12	Attendance type
13	Attended or did not attend
14	First attendance
15	Last DNA or patient cancelled date
16	Medical staff type seeing patient
17	Outcome of attendance
18	Priority type
19	Referral request received date
20	Service type requested
21	Source of referral for outpatients
22	Days waiting
23	Waiting/waiting calculation indicator also known as waiting quality indicator
24	All diagnosis codes
25	Primary diagnosis - 4 character
26	Primary diagnosis - 3 character (derived)
27	All operation codes
28	Main operation
29	Main operation - 3 character (derived)
30	Operation Status code
31	Main Specialty
32	Treatment Specialty

For peer review only

**33**	NHS generated HRG code
**34**	NHS generated HRG code version number
**35**	SUS generated HRG
**36**	SUS generated HRG version number
**37**	Commissioner code (pseudonymised by default)
**38**	Provider code - treatment
**39**	Pseudonymised provider code
**40**	Provider type
**41**	Patients census output area (2001) (10 character)
**42**	Patients census output area (2001) (6 character)
**43**	County of residence
**44**	Government office region of residence
**45**	Government office region of treatment
**46**	Patients electoral ward in 1991
**47**	Patients Primary Care Trust of residence - current
**48**	Patients Primary Care Trust of residence - historic
**49**	Patients Strategic Health Authority of Residence - current
**50**	Patients Strategic Health Authority of Residence - historic
**51**	Code of GP practice
**52**	Code of GP practice (Pseudonymised by default)
**53**	Consultant Code
**54**	Consultant Code (pseudonymised by default)
**55**	Code of patient's registered or referring general medical practitioner
**56**	Code of patient's registered or referring general medical practitioner (Pseudonymised)
**57**	Person referring patient
**58**	Referring organisation Code
**59**	Pseudonymised referring organisation code
**58**	Record Identifier (pseudonymised by default)
**59**	Datayear

************************** **End of**

For peer review only - http://bmjopen.bmj.com/site/about/guidelines.xhtml

For

For peer review only - http://bmjopen.bmj.com/site/about/guidelines.xhtml

For

For

For

For

For

For r

For

For peer review only - http://bmjopen.bmj.com/site/about/guidelines.xhtml

For peer review only - http://bmjopen.bmj.com/site/about/guidelines.xhtml

For peer review only

ıgland. This data is collected during a patient's time at hospital and is submitted to allow hospitals to be paid for the
ı-clinical purposes, of this administrative data.

ked below in red – will require you to have patient consent, section 251 approval, or the appropriate statutory
ates and geographies will be treated as de-personalised, unless anonymisation techniques are applied to render the
ılso been applied to some of these data to provide a less disclosive alternative. The key explaining how these
ı linked table to cancer registration data.

ıtlined above)
access
ılighted in dark grey

Field Name	Request Data Item	Justification
**Patient**		
PATIENTID		
Admincat		
ethnos		
Option to group ethnicities (e.g. white/ non-white/ unknown)		
**lopatid**		
**encrypted_hesid**		
**Appointment**		
apptdate		
Month of appointment		
Year of appointment		
Option to provide number of days from another event to the appt date (e.g. days from diagnosis to appointment)		
attendid		
atentype		
attended		
firstatt		
DNAdate		
stafftyp		
outcome		
priority		
reqdate		
servtype		
refsourc		
waiting		
wait_ind		
**Clinical**		
diag_nn		
diag_4		
diag3		
opertn_nn		
opertn_01		
opertn3		
operstat		
mainspef		
tretspef		
**Healthcare Resource Groups**		

hrgnhs		
hrgnhsvn		
sushrg		
sushrgvers		Available from 2009/10
**Organisation**		
purcode		
procodet		
procodet (pseudonymised)		
protype		
**Geographical**		
oacode01		
oacode6		
rescty		
resgor		
gortreat		
ward91		
respct06		
respct		
resstha06		
resstha		
**Practitioner**		
gpprac		
pseudogpprac		
consult		
pseudoconsult		
reggmp		
pseudoreggmp		
referrer		
referorg		
referorg (pseudonymised)		
**Systems Data**		
attendkeyanon		
datayear		

sheet *************************

ew only

For peer review only - http://bmjopen.bmj.com/site/about/guidelines.xhtml

ew only

For peer review only

For peer review only

For peer review only

For peer review only

For peer review only

For peer review only

For peer review only

For peer review only

For peer review only

For peer review only

For peer review only

For peer review only

For peer review only

For peer review only

For peer review only

Introduction

Accident and Emergency HES contains data on all A&E attendances at NHS hc
for the care they deliver. HES data is designed to enable secondary use, that is

Data items selection

Please be aware that selecting patient identifiable data items – **the data items**
regulation covering your organisation. Sensitive patient demographic data, eve
data anonymised to k-3 anonymity. Privacy by design and default principles ha
alternative can be selected is included below. **HES data will only be supplied**

Key:

**Items in red**	Identifiable fields that require additional permissions to access
	Sensitive items that require additional justification in your proto
	Privacy by design and default' alternatives to the sensitive item

Item number	Data item
**1**	Pseudonymised patient ID
**2**	Ethnic category
**3**	**Local patient identifier**
**4**	**Patient ID - HES generated**
**5**	Arrival mode
**6**	Attendance category
**7**	Attendance disposal
**8**	Department type
**9**	Duration to assessment
**10**	Duration to treatment
**11**	Duration to conclusion
**12**	Duration to departure
**13**	Incident location type
**14**	Patient group
**15**	Source of referral
**16**	Arrival date
**17**	Day of the week of the arrival
**18**	Arrival on a: weekday / weekend
**19**	Days from arrival date to another event
**20**	Arrival time
**21**	Arrival time occurring in the: morning / afternoon / evening
**22**	A&E diagnosis
**23**	A&E diagnosis - 2 character
**24**	A&E diagnosis - Anatomical area
**25**	A&E diagnosis - Anatomical side
**26**	A&E investigation
**27**	A&E investigation - 2 character
**28**	A&E treatment
**29**	A&E treatment - 2 character

For peer review

**30**	2001 Census output area
**31**	2001 Census output area (6 character)
**32**	County of residence
**33**	Current electoral ward
**34**	Current PCT of residence
**35**	Current SHA of residence
**36**	Government Office Region of residence
**37**	Health authority of residence
**38**	Historic PCT of residence
**39**	Historic SHA of residence
**40**	LA district of residence
**41**	Region of residence
**42**	Government Office Region of treatment
**43**	Health Authority of treatment
**44**	PCT of treatment
**45**	Region of treatment
**46**	SHA of treatment
**47**	Dominant procedure
**48**	Trust derived HRG value
**49**	Version no. of trust derived HRG
**50**	SUS generated HRG (available 2009-2010)
**51**	SUS generated HRG version number (Available 2009 - 2010)
**52**	Provider code 3 - character
**53**	Provider code 3 - character (pseudonymised)
**54**	Provider code 5 - character
**55**	Pseudonymised Provider code 5 - character
**56**	Provider code - treatment
**57**	Pseudonymised treatment provider code
**58**	Provider type
**59**	Org code of patient path ID issuer
**60**	RTT period start
**61**	RTT period status
**62**	RTTP period end
**63**	Duration of wait (referral to treatment period)
**64**	GP practice code
**65**	GP practice code (pseuondymised by default)
**66**	Record Identifier (pseudonymised by default)
**67**	Datayear

************************* **End of sheet** *************************

For r

For r

For r

For peer review only - http://bmjopen.bmj.com/site/about/guidelines.xhtml

For r

For peer review only - http://bmjopen.bmj.com/site/about/guidelines.xhtml

For r

For peer review only - http://bmjopen.bmj.com/site/about/guidelines.xhtml

For r

For peer review only - http://bmjopen.bmj.com/site/about/guidelines.xhtml

For peer review only - http://bmjopen.bmj.com/site/about/guidelines.xhtml

ospitals in England. This data is collected during a patient's time at hospital and is submitted to allow hospitals to be paid
; use for non-clinical purposes, of this administrative data.

marked below in red – will require you to have patient consent, section 251 approval, or the appropriate statutory
ent dates and geographies will be treated as de-personalised, unless anonymisation techniques are applied to render the
ave also been applied to some of these data to provide a less disclosive alternative. The key explaining how these
as a linked table to cancer registration data.

(as outlined above)
ocol to access
ns highlighted in dark grey

Field name	Request data item	Justification
**Patient**		
PATIENTID		
ethnos		
**lopatid**		
**encrypted_hesid**		
**Attendances**		
aearrivalmode		
aeattendcat		
aeattenddisp		
aedepttype		
initdur		
tretdur		
concldur		
depdur		
aeincloctype		
aepatgroup		
aerefsource		
arrivaldate		
Option to provide the day of the week the A&E arrival took place		
Option to provide whether the A&E arrival was on a weekday or at the weekend		
Option to provide number of days from arrival to another event (e.g. days from A&E arrival to diagnosis)		
arrivaltime		
Option to provide the part of the day the patient arrived		
**Clinical diagnosis**		
diag_n		
diag2_n		
diaga_n		
diags_n		
**Clinical Investigation**		
invest_n		
invest2_n		
**Clinical treatment**		
treat_n		
treat2_n		
**Residence**		

oacode		
oacode6		
rescty		
currward		
respct06		
resstha06		
resgor		
resha		
respct02		
resstha02		
resladst		
resro		
**Treatment**		
gortreat		
hatreat		
pcttreat		
rotreat		
sthatret		
**HRG data**		
domproc		
hrgnhs		
hrgnhsvn		
sushrg		
sushrgvers		
**Organisation data**		
procode3		
procode3 (pseudonymised)		
procode		
procode (pseudonymised)		
procodet		
procodet (pseudonymised)		
protype		
**Patient Pathway**		
orgpppid		
rttperstart		
rttperstat		
rttperend		
waitdays		
**Practitioner Data**		
gpprac		
gpprac (pseudonymised)		
**System Data**		
aekeyanon		
datayear		

For peer review only - http://bmjopen.bmj.com/site/about/guidelines.xhtml

For peer review only

For peer review only

For peer review only

For peer review only

For peer review only

For peer review only

For peer review only

For peer review only

For peer review only

For peer review only

For peer review only

For peer review only

For peer review only

For peer review only

For peer review only

Introduction

Routes to Diagnosis: cancer registration data are combined with Administrative Hospital Episo cancer screening programmes. Using these datasets cancers registered in England which wer Routes to Diagnosis. The methodology is described in detail in the British Journal of Cancer ar journey using multiple routine datasets'.

Data items selection

Please select your required data items from the list below. If you require more details on the m http://www.ncin.org.uk/publications/routes_to_diagnosis or http://www.nature.com/bjc/journal/v

Please be aware that selecting patient identifiable data items – **the data items marked below** approval, or the appropriate statutory regulation covering your organisation. Sensitive patient as de-personalised, unless anonymisation techniques are applied to render the data anonymis

Item number	Data item	Field name
		**Route to diagnosis**
**1**	Tumour level pseudo ID (for linkage)	TUMOURID
**2**	Route to diagnosis code (the code assigned to a route for the purpose of the algorithm)	ROUTE_CODE
**3**	Finalised route to diagnosis (the published route with all datasets types accounted for)	FINAL_ROUTE

************************* **End of sheet** *************************

For peer review only

de Statistics data, Cancer Waiting Times data and data from the
e diagnosed in 2006 to 2015 are categorised into one of eight
ticle 'Routes to Diagnosis for cancer - Determining the patient

neaning and definition of specific data items please visit:
107/n8/full/bjc2012408a.html

in red – will require you to have patient consent, section 251
demographic data, event dates and geographies will be treated
sed to k-3 anonymity.

Request data item	Justification

For peer review only

Introduction

Systemic Anti-Cancer Therapy Data Set collects clinical management on patien
acute inpatient, daycase, outpatient settings and delivery in the community. It cov
designed to collect information on all drug treatments with an anti-cancer effect, in

Data validation is an important aspect of the programme. This is in three stages co
and validity of their data before inclusion for subsequent analysis.

Coverage

Data is available from April 2012 to September 2016 inclusive. The data is linked a

Data item selection

Please be aware that selecting patient identifiable data items – **the data items ma**
your organisation. Sensitive patient demographic data, event dates and geographi
anonymity. Privacy by design and default principles have also been applied to son

Key:

Items in red Identifiable fields that require additional permissions to access (as c
Sensitive items that require additional justification in your protocol to
Privacy by design and default' alternatives to the sensitive items hig

**Item number**	**Data item**
**1**	Pseudonymised patient ID
**2**	**NHS number**
**3**	NHS number status indicator code
**4**	**Date of birth**
**5**	Month of birth
**6**	Year of birth
**7**	Gender code (current)
**8**	Ethnicity
**9**	Broad ethnic group
**10**	Postcode
**11**	Broader geographic area/ IMD quintile
**12**	General medical practice code (patient registration)
**13**	**Consultant code (initiated SACT)**
**14**	Consultant code (pseudonymised)
**15**	Care professional main speciality code (start SACT)
**16**	Organisation code
**17**	Organisation code (pseudonymised)
**18**	Primary diagnosis (on SACT initiation)
**19**	Morphology (ICD-O on SACT initiation)
**20**	Pre- treatment (final) TNM stage
**21**	SACT programme number
**22**	Anti-cancer regimen number
**23**	Drug treatment intent
**24**	Regimen analysis grouping
**25**	Regimen grouping (benchmark reports)
**26**	Patient's height (metres (m))
**27**	Patient's weight (kilograms (kg))
**28**	Performance Status (Adult)
**29**	Performance Status (Young Person)
**30**	Co-morbidity adjustment indicator

31	Decision to treat date (Drug regimen)
32	Month of decision to treat (Drug regimen)
33	Year of decision to treat (Drug regimen)
34	Days from another event to decision to treat date
35	Start date (Drug regimen)
36	Month of start date for drug regimen
37	Year of start date for drug regimen
38	Days from another event to drug regimen start date
39	Clinical trial indicator
40	Chemo-radiation indicator
41	Number of planned systemic anti-cancer therapy cycles
41	Cycle identifier
42	Start date (Cycle)
43	Month of start date of cycle
44	Year of start date of cycle
45	Days from another event to start date of cycle
46	Patient's Weight (Kilograms (kg))
47	Performance Status (Adult)
48	Performance Status (Young Person)
49	Primary procedure (OPCS)
50	Drug analysis grouping
51	Actual dose
52	SACT drug route of administration
53	SACT administration date
54	Organisation code (provider)
55	Pseudonymised organisation code (provider)
56	Primary procedure (OPCS)
57	Start date (Final therapy)
58	Month of final therapy
59	Year of final therapy
60	Days from another event to start date of final therapy
61	Regimen modification indicator (dose reduction)
62	Regimen modification indicator (time delay)
63	Regimen modification indicator (days reduced)
64	Planned treatment change reason

************************* End of sheet *************************

ıts receiving cancer chemotherapy in or funded by the NHS in England. It relates to all canc
/ers chemotherapy treatment for all solid tumour and haematological malignancies and thos
ı all treatment settings, including traditional cytotoxic chemotherapy and all newer agents.

overing data quality, confirmation of activity levels and an initial clinical analysis. Trusts are r

at a patient level and can be linked to the latest available Cancer Reg- Patient Table

arked below in red – will require you to have patient consent, section 251 approval, or the a
ıies will be treated as de-personalised, unless anonymisation techniques are applied to rend
ıe of these data to provide a less disclosive alternative. The key explaining how these alterr

ɔutlined above)
o access
ȷhlighted in dark grey

Field name	Request data item
**Demographics and consultant**	
PATIENTID	
**NHS_Number**	X
NHS_Number_Status	
**Date_Of_Birth**	X
MONTH_DOB	
YEAR_DOB	
Gender_Current	X
Ethnicity	X
Option to group ethnicities (e.g. white/ non-white/ unknown)	
Postcode	X
Option to provide geography as deprivation score or aggregate to larger geographic areas such as MSOA or county.	X
GP_Practice_Code	
**Consultant_GMC_Code_Clean**	
Consultant_GMC_Code (pseudonymised)	
Consultant_Speciality_Code	
Organisation_Code_of_Provider	
Organisation_Code_of_Provider (pseudonymised)	
**Clinical status**	
Primary_Diagnosis	X
Morphology_clean	X
Stage_at_Start	X
**Programme and regimen**	
Programme_Number	X
Regimen_Number	X
Intent_of_Treatment	X
Analysis_Group	X
Benchmark_Group	X
Height_At_Start_of_Regimen	X
Weight_At_Start_of_Regimen	X
Performance_Status_at_Start_of_Regimen_Clean	X
Performance_Status_at_Start_of_Regimen_Clean	X
Comorbidity_Adjustment	X

Date_Decision_To_Treat	X
Month of decision to treat	X
Year of decision to treat	X
Option to provide number of days from another event to the date of the decision to treat (e.g. days from diagnosis to date of decision to treat)	X
Start_Date_of_Regimen	X
Month of start date of drug regimen	X
Year of start date of drug regimen	X
Option to provide number of days from another event to the start date of the drug regimen (e.g. days from date of decision to treat to start date of regimen)	X
Clinical_Trial	X
Chemo_Radiation	X
Number_of_Cycles_Planned	X
**Cycle**	
Cycle_Number	X
Start_Date_of_Cycle	X
Month of start date of cycle	X
Year of start date of cycle	X
Option to provide number of days from another event to the start date of the cycle (e.g. days from diagnosis to start date of cycle)	X
Weight_At_Start_Of_Cycle	X
Performance_Status_At_Start_Of_Cycle_Clean	X
Performance_Status_At_Start_Of_Cycle_Clean	X
OPCS_Procurement_Code	X
**Drug details**	
Drug_Group	X
Actual_Dose_Per_Administration	X
Administration_Route	
Administration_Date	X
Organisation_Code_of_Drug_Provider	
Organisation_Code_of_Drug_Provider (pseudonymised)	
OPCS_Delivery_Code	
**Outcome**	
Date_of_Final_Treatment	X
Month of final therapy	X
Year of final therapy	X
Option to provide number of days from another event to the start date of the final therapy (e.g. days from diagnosis to start date of final therapy)	X
Regimen_Modification_Dose_Reduction	X
Regimen_Modification_Time_Delay	X
Regimen_Modification_Stopped_Early	X
Regimen_Outcome_Summary	X

ɔer patients, both adult and paediatric, in
e in clinical trials. The dataset has been

required to check the quality, quantity

appropriate statutory regulation covering
er the data anonymised to k-3
ıative can be selected is included below:

Justification
To better understand cancer morphology and related treatment
To better understand cancer morphology and related treatment
To better understand cancer morphology and related treatment
To better understand cancer morphology and related treatment
To better understand cancer morphology and related treatment
To better understand cancer morphology and related treatment
To better understand cancer morphology and related treatment
To better understand cancer morphology and related treatment
To better understand cancer morphology and related treatment
To better understand cancer morphology and related treatment

To better understand cancer morphology and related treatment
To better understand cancer morphology and related treatment (if above not available)
To better understand cancer morphology and related treatment (if above not available)
Days from diagnosis to date of decision to treat
To understand cancer treatment
To understand cancer treatment
To understand cancer treatment
To understand cancer treatment
To understand cancer treatment
To understand cancer treatment
To understand cancer treatment
To understand cancer treatment
To understand cancer treatment
To understand cancer treatment
To understand cancer treatment
To understand cancer treatment
To understand cancer treatment
To understand cancer treatment
To understand cancer treatment
To understand cancer treatment
To understand cancer treatment
To understand cancer treatment
To understand cancer treatment
To understand cancer treatment

For peer review only

For peer review only

peer review only

For peer review only

For peer review only

Introduction

A **Radiotherapy** EPISODE is a continuous period of care for radic
Treatment given concurrently or consecutively to multiple anatomi
If the treatment plan changes during treatment (e.g.: because a ne
Treatment given to separate unrelated PRIMARY tumour sites will

Inclusions:

All radiotherapy of the following types:
- Teletherapy
- Brachytherapy given using automated remote afterloading machi
- All other brachytherapy given for the treatment of malignant dise

delivered in England to patients in NHS facilities, or in private facil

Exclusions:

- Brachytherapy delivered using other than automated remote afte
- Radiotherapy delivered using unsealed sources
- Radiotherapy delivered outside of England
- Radiotherapy delivered in a non-NHS setting and not funded by t
- Non-therapeutic exposures delivered using a radiotherapy machi
- Non-patient exposures (e.g. dosimetry exposures, blood or tissu

Coverage

Data is available from 01/04/2009 – 31/03/2016. The data is linked

Data item selection

Please be aware that selecting patient identifiable data items – **the**
demographic data, event dates and geographies will be treated as
these data to provide a less disclosive alternative. The key explain

Key:

**Items in red**	Identifiable fields that require additional permissions
	Sensitive items that require additional justification ir
	Privacy by design and default' alternatives to the se

Item number	Name
1	Pseudonymised patient ID
2	ATTENDANCE IDENTIFIER (Pseudonymised)
3	ORGANISATION CODE (LOCAL PATIENT IDENTIFIER)
4	PATIENT ID (Pseudonymised)
	A Radiotherapy EPISODE is a co<br>Treatment given con<br>If the treatment plan changes during treatment
5	RADIOTHERAPY EPISODE IDENTIFIER (Pseudonymised)
6	APPOINTMENT DATE
7	Month of appointment
8	Year of appointment
9	Days from another event to appointment date

**10**	DECISION TO TREAT DATE (RADIOTHERAPY TREATMENT EPISODE)
**11**	Month of decision to treat date
**12**	Year of decision to treat date
**13**	Days from another event to decision to treat date
**14**	EARLIEST CLINICALLY APPROPRIATE DATE
**15**	Month of earliest clinically appropriate date
**16**	Year of earliest clinically appropriate date
**17**	Days from another event to decision to earliest clinically appropriate date
**18**	RADIOTHERAPY PRIORITY
**19**	TREATMENT START DATE (RADIOTHERAPY TREATMENT EPISODE)
**20**	Month of treatment start date
**21**	Year of treatment start date
**22**	Days from another event to treatment start date
**23**	RADIOTHERAPY DIAGNOSIS (ICD)
**24**	RADIOTHERAPY INTENT
A radiotl	
**25**	PRESCRIPTION IDENTIFIER (Pseudonymised)
**26**	RADIOTHERAPY TREATMENT REGION
**27**	ANATOMICAL TREATMENT SITE (RADIOTHERAPY)
**28**	NUMBER OF TELETHERAPY FIELDS
**29**	RADIOTHERAPY PRESCRIBED DOSE
**30**	PRESCRIBED FRACTIONS
**31**	RADIOTHERAPY ACTUAL DOSE
**32**	ACTUAL FRACTIONS
**33**	RADIOTHERAPY TREATMENT MODALITY
**34**	RADIOTHERAPY FIELD IDENTIFIER (Pseudonymised)

For peer review only

35	MACHINE IDENTIFIER
36	MACHINE IDENTIFIER (pseudonymised by default)
37	RADIOISOTOPE
38	RADIOTHERAPY BEAM TYPE
39	RADIOTHERAPY BEAM ENERGY
40	TIME OF EXPOSURE
41	ORGANISATION CODE (CODE OF PROVIDER)
42	ORGANISATION CODE (CODE OF PROVIDER) - pseudonymised by default
43	PROCEDURE (OPCS)
44	PROCEDURE DATE
45	Month of procedure
46	Year of procedure
47	Days from another event to procedure date

************************* **End of sheet** *************************

otherapy (from start to finish) including all preparation, planning and delivery of tre
cal sites associated with the same primary tumour will form part of the same radi
ew symptom arises which also requires radiotherapy) then all of the treatment del
l form separate episodes.

ines
ase
ities where delivery is funded by the NHS, from 1st April 2016.

erloading, for the treatment of non-malignant disease

the NHS
ines (e.g.: imaging)
es, animals)

d at a patient level and can be linked to the latest available Cancer Reg- Patient T

e data items marked below in red – will require you to have patient consent, se
s de-personalised, unless anonymisation techniques are applied to render the dat
ning how these alternative can be selected is included below:

s to access (as outlined above)
n your protocol to access
ensitive items highlighted in dark grey

Description
**RTDS LINKAGE**<br>**Patient information**<br>The following data items are required for patient link
Project specific patient ID
A sequential number or time of day used to enable an attendance to be uniquely identified.
This is the ORGANISATION CODE of the ORGANISATION that assigned the LOCAL PATIENT IDENTIFIER
Patient ID from AV_TUMOUR on CAS
**RADIOTHERAPY EPISODE**<br>To carry the details of the Episode of radiotherapy being giv<br>ntinuous period of care for radiotherapy (from start to finish) including all prepara<br>ncurrently or consecutively to multiple anatomical sites associated with the same<br>(e.g.: because a new symptom arises which also requires radiotherapy) then all o<br>Treatment given to separate unrelated PRIMARY tumour sites w
Any identifier that is unique for each radiotherapy episode.
Date when PATIENT is to be seen by or be in contact with one or more CARE PROFESSIONALS.
Derived from APPOINTMENT DATE field
Derived from APPOINTMENT DATE field
Derived from APPOINTMENT DATE field

only

The date on which it was decided that the PATIENT required a specific Planned Cancer Treatment. This is the date that the consultation between the PATIENT and the clinician took place and a Planned Cancer Treatment was agreed.
Derived from DECISION TO TREAT DATE field
Derived from DECISION TO TREAT DATE field
Derived from DECISION TO TREAT DATE field
This is the first date that the patient would have been available to start radiotherapy.
Derived from EARLIEST CLINICALLY APPROPRIATE DATE field
Derived from EARLIEST CLINICALLY APPROPRIATE DATE field
Derived from EARLIEST CLINICALLY APPROPRIATE DATE field
The priority for this course of therapy as classified by the requesting clinician.
The start of a stay, an episode, period covered by a plan or other time period. This may be used to calculate the length of the period, or to classify by financial year or other time-based criterion.
Derived from TREATMENT START DATE field
Derived from TREATMENT START DATE field
Derived from TREATMENT START DATE field
This is the PATIENT DIAGNOSIS for:<br>• Patients with cancer, the primary tumour diagnosis code or<br>• non-cancer diagnoses, the main condition being treated during the episode of radiotherapy<br>Note: The definition of this field is different from that of the Primary Diagnosis in CDS.
The intent of the delivered beam radiation.
**RADIOTHERAPY PRESCRIPTION**<br>**To carry the details of each Prescription of radiotherapy bein**<br>herapy prescription specifies a radiotherapy field arrangement, dose and fractiona
Any identifier that is unique for each radiotherapy prescription.
The specific area to be treated with radiotherapy.
The part of the body to which the RADIOTHERAPY ACTUAL DOSE is administered.
The prescribed number of fields of a Teletherapy Treatment Course.
The total prescribed absorbed radiation dose in Grays
The prescribed number of Fractions or hyperfractionation of a Teletherapy Treatment Course
The total actual absorbed radiation dose given in Grays. *This item may be omitted from all but the ultimate fraction for this prescription.*
The total number of Fractions or hyperfractionation of a Teletherapy Treatment Course administered. *This item may be omitted from all but the ultimate fraction for this prescription.*
The type of treatment delivered during a RADIOTHERAPY PRESCRIPTION.
**RADIOTHERAPY EXPOSURE**<br>**To carry the details of each radiotherapy exposure delive**<br>A Radiotherapy Exposure is the delivery of one treatment field as pa
An identifier that is unique for each radiotherapy exposure.

For peer review only

A unique code ascribed to the radiotherapy equipment used to treat this exposure.<br>This identifier is made up of:<br>Five character NACS site code (R----)<br>Two character equipment type code (LA/CO/KV/OT)<br>Four digit unique sequence number (issued by RTDS).
A pseudonymised code ascribed to the radiotherapy equipment used to treat this exposure.<br>This identifier is made up of:<br>Five character NACS site code (R----)<br>Two character equipment type code (LA/CO/KV/OT)<br>Four digit unique sequence number (issued by RTDS).
The type of radioactive source used to deliver radiotherapy with brachytherapy. To record the isotope in standard scientific notation (e.g.: I123 or Ir192)
The prescribed type of beam of a Teletherapy Treatment Course.
Beam energy in MeV/MV/MVp. Record kVp energies as decimals (e.g. 250kV = 0.25MV). Only for multi-modality machines.
Time when the exposure was initiated
**OPCDS PROCEDURES**
This is the ORGANISATION CODE of the ORGANISATION acting as a Health Care Provider.
This is a pseudonymised ORGANISATION CODE of the ORGANISATION acting as a Health Care Provider.
**Start of repeating Item**
Procedure carried out and recorded for CDS or HES purposes.
The date of the occurrence of the CLINICAL INTERVENTION.
Derived from PROCEDURE DATE field
Derived from PROCEDURE DATE field
Derived from PROCEDURE DATE field
**End of repeating item**

eatment as covered in the treatment intention.
iotherapy episode.
livered concurrently or consecutively will form part of the same episode.

Table

ction 251 approval, or the appropriate statutory regulation covering your organisation. Sensitive patient ta anonymised to k-3 anonymity. Privacy by design and default principles have also been applied to some of

Variable name	Request Data Item	Justification
kage purposes.		
PATIENTID		
OPCDS_LINKAGE.ATTENDID		
OPCDS_LINKAGE.ORGCODEPROVIDER		
OPCDS_LINKAGE.PATIENTID		
ven at this attendance.<br>ition, planning and delivery of treatment as covered in the treatment intention.<br>primary tumour will form part of the same radiotherapy episode.<br>of the treatment delivered concurrently or consecutively will form part of the same episode.<br>vill form separate episodes.		
RTDS_EPISODES.RADIOTHERAPYEPISODEID	X	
RTDS_EPISODES.APPTDATE	X	
Month of appointment	X	
Year of appointment	X	
Option to provide number of days from another event to the appointment date (e.g. days from diagnosis to appointment date)	X	Days from diagnosis to appointment date

RTDS_EPISODES.DECISIONTOTREATDATE	X	
Month of decision to treat date	X	
Year of decision to treat date	X	
Option to provide number of days from another event to the d		Days from diagnosis to decision to treat
RTDS_EPISODES.EARLIESTCLINAPPROPRIATEDATE		
Month of earliest clinically appropriate date		
Year of earliest clinically appropriate date		
Option to provide number of days from another event to the earliest clinically appropriate date (e.g. days from diagnosis to earliest clinically appropriate date)		
RTDS_EPISODES.RADIOTHERAPYPRIORITY	X	To better understand cancer treatment
RTDS_EPISODES.TREATMENTSTARTDATE	X	To better understand cancer treatment
Month of treatment start date	X	To better understand cancer treatment
Year of treatment start date	X	To better understand cancer treatment
Option to provide number of days from another event to the treatment date (e.g. days from diagnosis to treatment start date)	X	To better understand cancer treatment
RTDS_EPISODES.RADIOTHERAPYDIAGNOSISID	X	To better understand cancer treatment
RTDS_EPISODES.RADIOTHERAPYINTENT	X	To better understand cancer treatment
**g given at this attendance.**<br>ation for treatment to an 'ICRU-like' reference point.		
RTDS_PRESCRIPTIONS.PRESCRIPTIONID	X	To better understand cancer treatment
RTDS_PRESCRIPTIONS.RTTREATMENTREGION	X	To better understand cancer treatment
RTDS_PRESCRIPTIONS.RTTREATMENTANATOMICALSITE	X	To better understand cancer treatment
RTDS_PRESCRIPTIONS.NUMBEROFTELETHERAPYFIELDS	X	To better understand cancer treatment
RTDS_PRESCRIPTIONS.RTPRESCRIBEDDOSE	X	To better understand cancer treatment
RTDS_PRESCRIPTIONS.PRESCRIBEDFRACTIONS	X	To better understand cancer treatment
RTDS_PRESCRIPTIONS.RTACTUALDOSE	X	To better understand cancer treatment
RTDS_PRESCRIPTIONS.ACTUALFRACTIONS	X	To better understand cancer treatment
RTDS_PRESCRIPTIONS.RTTREATMENTMODALITY	X	To better understand cancer treatment
**ered at this attendance.**<br>art of a Fraction of Radiotherapy		
RTDS_EXPOSURES.RADIOTHERAPYFIELDID		

RTDS_EXPOSURES.MACHINEID		
RTDS_EXPOSURES.MACHINEID (pseudonymised)		
RTDS_EXPOSURES.RADIOISOTOPE		
RTDS_EXPOSURES.RADIOTHERAPYBEAMTYPE		
RTDS_EXPOSURES.RADIOTHERAPYBEAMENERGY		
RTDS_EXPOSURES.TIMEOFEXPOSURE		
OPCDS_PROCEDURES.ORGCODEPROVIDER		
OPCDS_PROCEDURES.ORGCODEPROVIDER (pseudonymised)		
OPCDS_PROCEDURES.PRIMARYPROCEDUREOPCS	X	To better understand cancer treatment
OPCDS_PROCEDURES.PROCEDUREDATE		
Month of procedure		
Year of procedure		
Option to provide number of days from another event to the treatment date (e.g. days from diagnosis to procedure date)		

Introduction:

The National Cancer Patient Experience Survey (CPES), commissioned by NHS England through Quality Health i Trust in England providing adult cancer services as inpatients or day cases, and discharged within a specified thre through diagnosis and treatment and to the ongoing management of their cancer.

Data coverage:
Wave 1 - 2010: Patients discharged between 01/01/10 – 31/03/10
Wave 2 - 2011/12: Patients discharged between 01/09/11 – 30/11/11
Wave 3 - 2013: Patients discharged between 01/09/12 – 30/09/12
Wave 4 - 2014: Patients discharged between 01/09/13 – 30/11/13
Wave 5 - 2015: Patients discharged between 01/04/2015 - 30/06/2015

Data items selection:

Please be aware that selecting patient identifiable data items – **the data items marked below in red** – will require event dates and geographies will be treated as de-personalised, unless anonymisation techniques are applied to r alternative. The key explaining how these alternative can be selected is included below:

Key:
Items in red Identifiable fields that require additional permissions to access (as outlined above)
Sensitive items that require additional justification in your protocol to access
Privacy by design and default' alternatives to the sensitive items highlighted in dark grey

	**W** Pati
**Item number**	**Data item**
**1**	Pseudonymised patient ID
**2**	Pseudonymised tumourID
**3**	How many times did you see your GP about the health problem caused by cancer
**4**	How long did you have to wait before your first appointment after your GP told you that you would need to see a hospital doctor
**5**	How do you feel about the length of time you had to wait before your first appointment with a hospital doctor
**6**	How long did you have to wait before your first appointment with a hospital doctor
**7**	Did your health get worse, better or stay the same while you were waiting for first appointment
**8**	In the last 12 months have you had diagnostics tests
**9**	Did staff give complete explanation of purpose of test(s)
**10**	Did staff explain completely what would be done during test
**11**	Were you given easy to understand written information about test
**12**	Were you given complete explanation of test results in understandable way
**13**	Who first told you that you had cancer
**14**	Were you told they could bring a friend when first told they had cancer
**15**	How do you feel about the way you were told that you had cancer
**16**	Did you understand the explanation of what was wrong with you
**17**	Were you given written information about the type of cancer you had

**18**	Before your cancer treatment started, were you given a choice of different types of treatment
**19**	Were the possible side effects of treatments explained in a way you could understand
**20**	Were you given written information about the side effects of treatments
**21**	Were you involved as much as you wanted to be in decisions about your care and treatment
**22**	Were you given the name of the CNS in charge of your care
**23**	How easy is it for you to contact your CNS
**24**	Did your CNS listen carefully to you the last time you spoke
**25**	How often do you get understandable answers from you CNS
**26**	The last time you saw or spoke to your CNS, how do you feel about the time you spent with them
**27**	Did hospital staff give information about support/self-help groups
**28**	Did hospital staff give information about financial help or any benefits you might be entitled to
**29**	Did hospital staff tell you that you could get free prescriptions
**30**	Have you had an operation in the last 12 months
**31**	Was your admission date changed to a later date by the hospital
**32**	Did staff give complete explanation of what would be done
**33**	Were you given written information about the operation
**34**	Did staff explain how operation had gone in understandable way
**35**	Have you had an operation or stayed overnight for cancer care
**36**	When you had important questions how often did you get understandable answers
**37**	Did you have confidence and trust in the doctors treating you
**38**	Do you think the doctors treating you knew enough about how to treat your cancer
**39**	Did doctors talk in front of you as if you weren't there
**40**	Did your friends and family have enough opportunity to talk to a doctor if they wanted to
**41**	When you had important questions to ask a ward nurse how often did you get understandable answers
**42**	Did you have confidence and trust in the ward nurses treating you
**43**	Did ward nurses talk in front of you as if you weren't there
**44**	In your opinion were there enough nurses on duty to care for you in hospital
**45**	Did you ever think that doctors or nurses were deliberately not telling you certain things you wanted to know
**46**	Did you ever get conflicting information from your treatment team
**47**	Were you given enough privacy when discussing your condition or treatment
**48**	Were you given enough privacy when being examined or treated
**49**	Do you think the hospital staff did everything they could to help control your pain
**50**	Were you treated with respect and dignity by hospital staff
	**Inf**
**51**	Were you given clear written information about what you should and should not do after you left hospital
**52**	Did staff tell you who to contact if you were worried about your condition or treatment
**53**	Were your family given all the information they needed to help care for you at home

For peer review only

54	After leaving hospital were you given enough care and help from health and social services
55	During the past 12 months have you had radiotherapy
56	Did hospital staff do everything possible to control the side effects of radiotherapy
57	During the past 12 months have you had chemotherapy
58	Did hospital staff do everything possible to control the side effects of chemotherapy
59	As an OP or day case did staff do everything they could to help control your pain
60	As an OP or day case were you given enough emotional support from staff
61	In the last 12 months have you had an OP appointment with a cancer doctor
62	How long after your appointed time did your OP appointment start
63	How was the length of your appointment with your cancer doctor
64	Did the doctor have the correct documents
65	Was your GP given enough information about your condition and treatment you had at the hospital
66	Did the practice staff do everything they could to support you while you were having cancer treatment
67	Did the different people treating and caring for you work well together to give you the best possible care
68	How much information were you given about your condition and treatment
69	Did you ever feel in your NHS care over the last year like a 'set of cancer symptoms'
70a	What year were you born
70b	Age bands
71	Gender
72a-g	Do you suffer from any of the following long-standing conditions
73	How long is it since you were treated for this cancer
74	**Sexual Orientation**
75	Can we send you a survey in the future to ask about your health and healthcare
76a	Ethnic Group
76b	Broader ethnic group

	**Wa[illegible]** Pati[illegible]
**Item number**	**Data item**
1	Pseudonymised patient ID
2	Pseudonymised tumourID
3	How many times did you see your GP about the health problem caused by cancer
4	How do you feel about the length of time you had to wait before your first appointment with a hospital doctor
5	How long did you have to wait before your first appointment with a hospital doctor

For peer review only

6	Did your health get worse, better or stay the same while you were waiting for first appointment
7	In the last 12 months have you had diagnostics tests
8	Did staff give complete explanation of purpose of test(s)
9	Did staff explain completely what would be done during test
10	Were you given easy to understand written information about test
11	Were you given complete explanation of test results in understandable way
12	Who first told you that you had cancer
13	Were you told they could bring a friend when first told they had cancer
14	How do you feel about the way you were told that you had cancer
15	Did you understand the explanation of what was wrong with you
16	Were you given written information about the type of cancer you had
17	Before your cancer treatment started, were you given a choice of different types of treatment
18	Do you think you were taken into account when teams were discussing which treatment you should have
19	Were the possible side effects of treatments explained in a way you could understand
20	Were you given written information about the side effects of treatments
21	Were you involved as much as you wanted to be in decisions about your care and treatment
22	Were you given the name of the CNS in charge of your care
23	How easy is it for you to contact your CNS
24	Did your CNS listen carefully to you the last time you spoke
25	How often do you get understandable answers from you CNS
26	Did hospital staff give information about support/self-help groups
27	Did hospital staff give information about financial help or any benefits you might be entitled to
28	Did hospital staff tell you that you could get free prescriptions
29	Taking part in cancer research discussed with patient
30	If yes, were you glad to have been asked
31	If no, would you have liked to have been asked
32	Have you had an operation in the last 12 months
33	Was your admission date changed to a later date by the hospital
34	Did staff give complete explanation of what would be done
35	Were you given written information about the operation
36	Did staff explain how operation had gone in understandable way
37	Have you had an operation or stayed overnight for cancer care

For peer review only

38	When you had important questions how often did you get understandable answers
39	Did you have confidence and trust in the doctors treating you
40	Did doctors talk in front of you as if you weren't there
41	Did your friends and family have enough opportunity to talk to a doctor if they wanted to
42	When you had important questions to ask a ward nurse how often did you get understandable answers
43	Did you have confidence and trust in the ward nurses treating you
44	Did ward nurses talk in front of you as if you weren't there
45	In your opinion were there enough nurses on duty to care for you in hospital
46	Did you ever think that doctors or nurses were deliberately not telling you certain things you wanted to know
47	Did you ever get conflicting information from your treatment team
48	Were you asked what name you prefer to be called by
49	Were you given enough privacy when discussing your condition or treatment
50	Were you given enough privacy when being examined or treated
51	Were you able to discuss any worries or fears with staff during your hospital visit
52	Do you think the hospital staff did everything they could to help control your pain
53	Were you treated with respect and dignity by hospital staff
	**Inf**
54	Were you given clear written information about what you should and should not do after you left hospital
55	Did staff tell you who to contact if you were worried about your condition or treatment
56	Were your family given all the information they needed to help care for you at home
57	After leaving hospital were you given enough care and help from health and social services
58	Did hospital staff do everything possible to control the side effects of radiotherapy
59	Did hospital staff do everything possible to control the side effects of chemotherapy
60	As an OP or day case did staff do everything they could to help control your pain
61	As an OP or day case were you given enough emotional support from staff
62	In the last 12 months have you had an OP appointment with a cancer doctor
63	How long after your appointed time did your OP appointment start
64	How was the length of your appointment with your cancer doctor
65	Did the doctor have the correct documents
66	Was your GP given enough information about your condition and treatment you had at the hospital
67	Did the practice staff do everything they could to support you while you were having cancer treatment
68	Did the different people treating and caring for you work well together to give you the best possible care
69	How much information were you given about your condition and treatment
70	Have you been offered a written assessment and care plan
71	Did you ever feel in your NHS care over the last year like a 'set of cancer symptoms'
72	Overall, how would you rate you care
73	What year were you born
74	Age bands
75	Gender

**76**	**Sexual Orientation**
**77a-g**	Do you suffer from any of the following long-standing conditions
**78**	How long is it since you were treated for this cancer
**79**	Can we send you a survey in the future to ask about your health and healthcare
**80a**	Ethnic Group
**80b**	Broader ethnic group

	**W** Pati
**Item number**	**Data item**
**1**	Pseudonymised patient ID
**2**	Pseudonymised tumourID
**3**	How many times did you see your GP about the health problem caused by cancer
**4**	How do you feel about the length of time you had to wait before your first appointment with a hospital doctor
**5**	How long did you have to wait before your first appointment with a hospital doctor
**6**	Did your health get worse, better or stay the same while you were waiting for first appointment
**7**	In the last 12 months have you had diagnostics tests
**8**	Did staff give complete explanation of purpose of test(s)
**9**	Did staff explain completely what would be done during test
**10**	Were you given easy to understand written information about test
**11**	Were you given complete explanation of test results in understandable way
**12**	Who first told you that you had cancer
**13**	Were you told they could bring a friend when first told they had cancer
**14**	How do you feel about the way you were told that you had cancer
**15**	Did you understand the explanation of what was wrong with you
**16**	Were you given written information about the type of cancer you had
**17**	Before your cancer treatment started, were you given a choice of different types of treatment
**18**	Do you think you were taken into account when teams were discussing which treatment you should have
**19**	Were the possible side effects of treatments explained in a way you could understand
**20**	Were you given written information about the side effects of treatments

For peer review only

21	Were you told about side effects that could affect in the future
22	Were you involved as much as you wanted to be in decisions about your care and treatment
23	Were you given the name of the CNS in charge of your care
24	How easy is it for you to contact your CNS
25	Did your CNS listen carefully to you the last time you spoke
26	How often do you get understandable answers from you CNS
27	Did hospital staff give information about support/self-help groups
28	Did hospital staff give information about the impact cancer could have on work life or education
29	Did hospital staff give information about financial help or any benefits you might be entitled to
30	Did hospital staff tell you that you could get free prescriptions
31	Have you seen information about cancer research in the hospital
32	Taking part in cancer research discussed with patient
33	Patient has taken part in cancer research
34	Have you had an operation in the last 12 months
35	Did staff give complete explanation of what would be done
36	Were you given written information about the operation
37	Did staff explain how operation had gone in understandable way
38	Have you had an operation or stayed overnight for cancer care
39	When you had important questions how often did you get understandable answers
40	Did you have confidence and trust in the doctors treating you
41	Did doctors talk in front of you as if you weren't there
42	Did your friends and family have enough opportunity to talk to a doctor if they wanted to
43	When you had important questions to ask a ward nurse how often did you get understandable answers
44	Did you have confidence and trust in the ward nurses treating you
45	Did ward nurses talk in front of you as if you weren't there
46	In your opinion were there enough nurses on duty to care for you in hospital
47	Did you ever think that doctors or nurses were deliberately not telling you certain things you wanted to know
48	Did you ever get conflicting information from your treatment team
49	Were you asked what name you prefer to be called by
50	Were you given enough privacy when discussing your condition or treatment
51	Were you given enough privacy when being examined or treated
52	Were you able to discuss any worries or fears with staff during your hospital visit
53	Do you think the hospital staff did everything they could to help control your pain
54	Were you treated with respect and dignity by hospital staff
55	Were you given clear written information about what you should and should not do after you left hospital
56	Did staff tell you who to contact if you were worried about your condition or treatment
57	Were your family given all the information they needed to help care for you at home
58	After leaving hospital were you given enough care and help from health and social services

For peer review only

59	Did hospital staff do everything possible to control the side effects of radiotherapy
60	Did hospital staff do everything possible to control the side effects of chemotherapy
61	As an OP or day case did staff do everything they could to help control your pain
62	As an OP or day case were you given enough emotional support from staff
63	In the last 12 months have you had an OP appointment with a cancer doctor
64	Did the doctor have the correct documents
65	Was your GP given enough information about your condition and treatment you had at the hospital
66	Did the practice staff do everything they could to support you while you were having cancer treatment
67	Did the different people treating and caring for you work well together to give you the best possible care
68	Have you been treated by a physiotherapist following your cancer
69	Have you been treated by a Occupational Therapist following your cancer
70	Have you been treated by a Dietician following your cancer
71	Have you been treated by a Speech and Language Therapist following your cancer
72	Have you been treated by a Lymphoedema Specialist following your cancer
73	How much information were you given about your condition and treatment
74	Have you been offered a written assessment and care plan
75	Did you ever feel in your NHS care over the last year like a 'set of cancer symptoms'
76	Overall, how would you rate you care
71	What year were you born
72a	Age
72b	Age bands
73	Gender
74	**Sexual Orientation**
75	Employment Status
76	Do you suffer from any of the following long-standing conditions
77	How long is it since you were treated for this cancer
78	How has your cancer responded to treatment
79	Can we send you a survey in the future to ask about your health and healthcare
80a	Ethnic Group
80b	Broader ethnic group

	**W**<br>Pati
**Item number**	**Data item**
1	Pseudonymised patient ID
2	Pseudonymised tumourID

For peer review only

**3**	How many times did you see your GP about the health problem caused by cancer
**4**	How do you feel about the length of time you had to wait before your first appointment with a hospital doctor
**5**	How long did you have to wait before your first appointment with a hospital doctor
**6**	Did your health get worse, better or stay the same while you were waiting for first appointment
**7**	In the last 12 months have you had diagnostics tests
**8**	Did staff give complete explanation of purpose of test(s)
**9**	Did staff explain completely what would be done during test
**10**	Were you given easy to understand written information about test
**11**	Were you given complete explanation of test results in understandable way
**12**	Who first told you that you had cancer
**13**	Were you told they could bring a friend when first told they had cancer
**14**	How do you feel about the way you were told that you had cancer
**15**	Did you understand the explanation of what was wrong with you
**16**	Were you given written information about the type of cancer you had
**17**	Before your cancer treatment started, were you given a choice of different types of treatment
**18**	Do you think you were taken into account when teams were discussing which treatment you should have
**19**	Were the possible side effects of treatments explained in a way you could understand
**20**	Were you given written information about the side effects of treatments
**21**	Were you told about side effects that could affect in the future
**22**	Were you involved as much as you wanted to be in decisions about your care and treatment
**23**	Were you given the name of the CNS in charge of your care
**24**	How easy is it for you to contact your CNS
**25**	Did your CNS listen carefully to you the last time you spoke
**26**	How often do you get understandable answers from you CNS
**27**	Did hospital staff give information about support/self-help groups
**28**	Did hospital staff give information about the impact cancer could have on work life or education
**29**	Did hospital staff give information about financial help or any benefits you might be entitled to
**30**	Did hospital staff tell you that you could get free prescriptions
**31**	Have you seen information about cancer research in the hospital
**32**	Taking part in cancer research discussed with patient
**33**	Patient has taken part in cancer research

For peer review only

34	Have you had an operation in the last 12 months
35	Did staff give complete explanation of what would be done
36	Were you given written information about the operation
37	Did staff explain how operation had gone in understandable way
38	Have you had an operation or stayed overnight for cancer care
39	When you had important questions how often did you get understandable answers
40	Did you have confidence and trust in the doctors treating you
41	Did doctors talk in front of you as if you weren't there
42	Did your friends and family have enough opportunity to talk to a doctor if they wanted to
43	When you had important questions to ask a ward nurse how often did you get understandable answers
44	Did you have confidence and trust in the ward nurses treating you
45	Did ward nurses talk in front of you as if you weren't there
46	In your opinion were there enough nurses on duty to care for you in hospital
47	Did you ever think that doctors or nurses were deliberately not telling you certain things you wanted to know
48	Did you ever get conflicting information from your treatment team
49	Were you asked what name you prefer to be called by
50	Were you given enough privacy when discussing your condition or treatment
51	Were you given enough privacy when being examined or treated
52	Were you able to discuss any worries or fears with staff during your hospital visit
53	Do you think the hospital staff did everything they could to help control your pain
54	Were you treated with respect and dignity by hospital staff
55	Were you given clear written information about what you should and should not do after you left hospital
56	Did staff tell you who to contact if you were worried about your condition or treatment
57	Were your family given all the information they needed to help care for you at home
58	After leaving hospital were you given enough care and help from health and social services
59	Did hospital staff do everything possible to control the side effects of radiotherapy
60	Did hospital staff do everything possible to control the side effects of chemotherapy
61	As an OP or day case did staff do everything they could to help control your pain
62	As an OP or day case were you given enough emotional support from staff
63	In the last 12 months have you had an OP appointment with a cancer doctor
64	Did the doctor have the correct documents
65	Was your GP given enough information about your condition and treatment you had at the hospital
66	Did the practice staff do everything they could to support you while you were having cancer treatment
67	Did the different people treating and caring for you work well together to give you the best possible care
68	Have you been treated by a physiotherapist following your cancer
69	Have you been treated by a Occupational Therapist following your cancer
70	Have you been treated by a Dietician following your cancer

For peer review only

**71**	Have you been treated by a Speech and Language Therapist following your cancer
**72**	Have you been treated by a Lymphoedema Specialist following your cancer
**73**	How much information were you given about your condition and treatment
**74**	Have you been offered a written assessment and care plan
**75**	Did you ever feel in your NHS care over the last year like a 'set of cancer symptoms'
**76**	Overall, how would you rate you care
**77**	What year were you born
**78a**	Age
**78b**	Age bands
**79**	Gender
**80**	**Sexual Orientation**
**81**	Employment Status
**82**	Do you suffer from any of the following long-standing conditions
**83**	How long is it since you were treated for this cancer
**84**	Is this the first time you have been treated for cancer
**85**	How has you cancer responded to treatment
**86**	Can we send you a survey in the future to ask about your health and healthcare
**87a**	Ethnic Group
**87b**	Broader ethnic group

	**W**<br>Pati
**Item number**	**Data item**
**1**	Pseudonymised patient ID
**2**	Pseudonymised tumourID
**3**	Before you were told you needed to go to hospital about cancer, how many times did you see your GP (family doctor) about the health problem caused by cancer?
**4**	How do you feel about the length of time you had to wait before your first appointment with a hospital doctor?
**5**	How long was it from the time you first thought something might be wrong with you until you first saw a GP or other doctor?
**6**	In the last 12 months, have you had a diagnostic test(s) for cancer such as an endoscopy, biopsy, mammogram, or scan at one of the hospitals named in the covering letter?
**7**	Beforehand, did you have all the information you needed about your test?
**8**	Overall, how did you feel about the length of time you had to wait for your test to be done?
**9**	Were the results of the test explained in a way you could understand?

For peer review only

**10**	When you were first told that you had cancer, had you been told you could bring a family member or friend with you?
**11**	How do you feel about the way you were told you had cancer?
**12**	Did you understand the explanation of what was wrong with you?
**13**	When you were told you had cancer, were you given written information about the type of cancer you had?
**14**	Before your cancer treatment started, were your treatment options explained to you?
**15**	Were the possible side effects of treatment(s) explained in a way you could understand?
**16**	Were you offered practical advice and support in dealing with the side effects of your treatment(s)?
**17**	Before you started your treatment(s), were you also told about any side effects of the treatment that could affect you in the future rather than straight away?
**18**	Were you involved as much as you wanted to be in decisions about your care and treatment?
**19**	Were you given the name of a Clinical Nurse Specialist who would support you through your treatment?
**20**	How easy or difficult has it been for you to contact your Clinical Nurse Specilaist?
**21**	When you have had important questions to ask your Clinical Nurse Specialist, how often have you got answers you could understand?
**22**	Did hospital staff give you information about support or self-help groups for people with cancer?
**23**	Did hospital staff discuss with you or give you information about the impact cancer could have on your day to day activities (for example, your work life or education)?
**24**	Did hospital staff give you information about how to get financial help or any benefits you might be entitled to?
**25**	Did hospital staff tell you that you could get free prescriptions?
**26**	During the last 12 months, have you had an operation (such as removal of a tumour or lump) at one of the hospitals named in the covering letter?
**27**	Beforehand, did you have all the information you needed about your operation?
**28**	After the operation, did a member of staff explain how it had gone in a way you could understand?
**29**	During the last 12 months, have you had an operation or stayed overnight for cancer care at one of the hospitals named in the covering letter?
**30**	Did groups of doctors and nurses talk in front of you as if you weren't there?
**31**	Did you have confidence and trust in the doctors treating you?
**32**	If your family or someone else close to you wanted to talk to a doctor, were they able to?
**33**	Did you have confidence and trust in the ward nurses treating you?
**34**	In your opinion, were there enough nurses on duty to care for you in hospital?
**35**	While you were in hospital did the doctors and nurses ask you what name you prefer to be called by?
**36**	Were you given enough privacy when discussing your condition or treatment?
**37**	During your hospital visit, did you find someone on the hospital staff to talk to about your worries and fears?
**38**	Do you think the hospital staff did everything they could to help control your pain?

For peer review only - http://bmjopen.bmj.com/site/about/guidelines.xhtml

**39**	Overall, did you feel you were treated with respect and dignity while you were in the hospital?
**40**	Were you given clear written information about what you should or should not do after leaving hospital?
**41**	Did the hospital staff tell you who to contact if you were worried about your condition or treatment after you left hospital?
**42**	During the last 12 months, have you been treated as an oupatient or day case for cancer care at one of the hospitals named in the covering letter?
**43**	While you were being treated an an outpatient or day case, did you find someone on the hospital staff to talk to about your worries and fears?
**44**	The last time you had an outpatients appointment with a cancer doctor, did they have the right documents, such as medical notes, x-rays and test results?
**45**	During the last 12 months, have you had radiotherapy at any of the hospitals named in the covering letter?
**46**	Beforehand, did you have all of the information you needed about your radiotherapy treatment?
**47**	Once you started your treatment, were you given enough information about whether your radiotherapy was working in a way you could understand?
**48**	During the last 12 months, have you had chemotherapy at any of the hospitals named in the covering letter?
**49**	Beforehand, did you have all of the information you needed about your chemotherapy treatment?
**50**	Once you started your treatment, were you given enough information about whether your chemotherapy was working in a way you could understand?
**51**	Did the doctors or nurses give your family or someone close to you all the information they needed to help care for you at home?
**52**	During your cancer treatment, were you given enough care and support from health or social services (for example, district nurses, home helps or physiotherapists)?
**53**	Once your cancer treatment finished, were you given enough care and support from health or social services (for example, district nurses, home helps or physiotherapists)?
**54**	As far as you know, was your GP given enough information about your condition and the treatment you had at the hospital?
**55**	Do you think the GPs and nurses at your general practice did everything they could to support you while you were having cancer treatment?
**56**	Did the different people treating and caring for you (such as GP, hospital doctors, hospital nurses, specialist nurses, community nurses) work well together to give you the best possible care?
**57**	Have you been given a care plan?
**58**	Overall, how would you rate the administration of your care (getting letters at the right time, doctors having the right notes/tests results, etc.)?
**59**	Overall, how do you feel about the length of time you had to wait when attending clinics and appointments for your cancer treatment?
**60**	Since your diagnosis, has anyone discussed with you whether you would like to take part in cancer research?
**61**	Overall, how would you rate your care?
**62**	How long is it since you were first treated for this cancer?
**63**	Had your cancer spread to other organs or parts of your body at the time you were first told you had cancer?
**64**	Which of the following applies?
**67**	What year were you born?
**68a**	Age
**68b**	Age bands
**69**	Are you male or female?
**70**	**Which of the following options best describes how you think of yourself? (sexual orientation)**

For peer review only

**71**	Do you have any of the following long-standing conditions?
**72**	Could we send you a survey in the future to ask about your health and healthcare?
**73**	Is English your first language?
**74a**	What is your ethnic group?
**74b**	Broader ethnic group

For peer review only

is a survey sent out to all adult cancer patients (aged 16 and over) with a primary diagnosis of cancer who have been admitt
ee month sampling period each year. The survey aims to collect information from patients about their cancer journey from th

e you to have patient consent, section 251 approval, or the appropriate statutory regulation covering your organisation. Sens
render the data anonymised to k-3 anonymity. Privacy by design and default principles have also been applied to some of th

/ave 1 2010 Cancer Patient Experience Survey
ients discharged between 01/01/2010 – 31/03/2010

Possible answers	Field Name
	PATIENTID
	TUMOURID
**Seeing your GP**	
None I did not see my GP before going to hospital, I saw my GP once, I saw my GP twice, I saw my GP 3 or 4 times, I saw my GP 5 or more times, Don't know / Can't say	Q1
I was seen the same day or next day, I was seen after 2-14 days, I was seen in 3 to 4 weeks, Waited 1 to 4 months, Waited more than 4 months, I did not see my GP before going into hospital, I chose to have a later appointment than the one I was offered, Don't know / Can't remember	Q2
I was seen as soon as I thought was necessary, I should have been seen a bit sooner, I should have been seen a lot sooner	Q3
Less than 3 months, 3-6 months, 6-12 months, More than 12 months, Don't know / Can't remember	Q4
My health got worse, My health got better, My health stayed about the same	Q5
**Diagnostic Tests**	
Yes, No	Q6
Yes completely, Yes to some extent, No but I would have liked an explanation, I did not need an explanation, Don't know / Can't remember	Q7
Yes completely, Yes to some extent, No but I would have liked an explanation, I did not need an explanation, Don't know / Can't remember	Q8
Yes and it was easy to understand, Yes but it was difficult to understand, No but I would have liked written information about the test(s), I did not need written information, Don't know / Can't remember	Q9
Yes completely, Yes to some extent, No but I would have liked an explanation, I did not need an explanation, Don't know / Can't remember	Q10
**Finding out what was wrong with you**	
A hospital doctor, A hospital nurse, A GP (family doctor), Another health professional, A friend or relative, Nobody – I worked it out for myself	Q11
Yes, No, It was not necessary, I was told by phone or letter, Don't know / Can't remember	Q12
It was done sensitively, It should have been done a bit more sensitively, It should have been done a lot more sensitively	Q13
Yes I completely understood it, Yes I understood some of it, No I did not understand it, Can't remember	Q14
Yes and it was easy to understand, Yes but it was difficult to understand, No I was not given written information about the type of cancer I had, I did not need written information, Don't know / Can't remember	Q15

For peer review only

**Deciding the best treatment for you**	
Yes, No but I would have liked a choice, I was not given a choice because only one type of treatment was suitable for me, Not sure / Can't remember	Q16
Yes definitely, Yes to some extent, No side effects were not explained, I did not need an explanation, Not sure / Can't remember	Q17
Yes and it was easy to understand, Yes but it was difficult to understand, No I was not given written information about side effects, Don't know / Can't remember	Q18
Yes definitely, Yes to some extent, No but I would like to have been more involved, Only one type of treatment was suitable for me	Q19
**Clinical Nurse Specialist**	
Yes, No, Don't know / Not sure	Q20
Easy, Sometimes easy sometimes difficult, Difficult, I have not tried to contact her/him	Q21
Yes definitely, Yes to some extent, No	Q22
All or most of the time, Some of the time, Rarely or never, I do not ask any questions	Q23
Too short, About right, Too long	Q24
**Support for people with Cancer**	
Yes, No but I would have liked information, It was not necessary, Don't know / Can't remember	Q25
Yes, No but I would have liked information, It was not necessary, Don't know / Can't remember	Q26
Yes, No but I would have liked information, It was not necessary, Don't know / Can't remember	Q27
**Operations**	
Yes, No	Q28
No, Yes it was changed once, Yes it was changed 2 or 3 times, Yes it was changed 4 times or more	Q29
Yes completely, Yes to some extent, No but I would have liked an explanation, I did not need an explanation, Don't know / Can't remember	Q30
Yes and it was easy to understand, Yes but it was difficult to understand, No I was not given written information about my operation, Don't know / Can't remember	Q31
Yes completely, Yes to some extent, No but I would have liked an explanation, I did not need an explanation`	Q32
**Hospital doctors**	
Yes, No	Q33
All or most of the time, Some of the time, Rarely or never, I did not ask any questions	Q34
In all of them, In some of them, In none of them	Q35
Yes definitely, Yes to some extent, No, Don't know / Not sure	Q36
Yes often, Yes sometimes, No	Q37
Yes definitely, Yes to some extent, No, No family or friends were involved, My family did not want or need information, I did not want my family or friends to talk to a doctor	Q38
**Ward Nurses**	
All or most of the time, Some of the time, Rarely or never, I did not ask any questions	Q39
In all of them, In some of them, In none of them	Q40
Yes often, Yes sometimes, No	Q41
There were always or nearly always enough on duty, There were sometimes enough on duty, There were rarely or never enough on duty	Q42
**Hospital care and treatment**	
Often, Sometimes, Only once, Never	Q43
Often, Sometimes, Only once, Never	Q44
Yes always, Yes sometimes, No	Q45
Yes always, Yes sometimes, No	Q46
All of the time, Some of the time, Not at all, I did not have any pain	Q47
Always, Most of the time, Some of the time, Never	Q48
**formation given to you before you left hospital**	
Yes, No, Can't remember	Q49
Yes, No, Don't know / Can't remember	Q50
Yes definitely, Yes to some extent, No, No family or friends were involved, My family or friends did not want or need information, I did not want my family or friends to be given information	Q51
**Arranging home support**	

For peer review only

Yes definitely, Yes to some extent, No, I did not need help from health or social services, Don't know / Can't remember	Q52
**Hospital care as day patient/ outpatient**	
Yes, No	Q53
Yes definitely, Yes to some extent, No they could have done more, I have not had any side effects from radiotherapy	Q54
Yes, No	Q55
Yes definitely, Yes to some extent, No they could have done more, I have not had side effects from chemotherapy	Q56
Yes definitely, Yes to some extent, No they could have done more, I did not have any pain	Q57
Yes definitely, Yes to some extent, No I would have liked more support, I did not need emotional support from staff	Q58
**Outpatient appointments with doctors**	
Yes, No	Q59
Seen on time or early, Waited up to 5 minutes, Waited 6 - 15 minutes, Waited 16 - 30 minutes, Waited 31 - 60 minutes, Waited 1 to 2 hours, Waited more than 2 hours, Don't know / Can't remember	Q60
Too short, About right, Too long	Q61
Yes, No, Don't know / Can't remember	Q62
**Care from your general practice**	
Yes, No, Don't know / Can't remember	Q63
Yes definitely, Yes to some extent, No they could have done more, My general practice was not involved	Q64
**Your overall NHS care**	
Yes always, Yes most of the time, Yes some of the time, No never, Don't know	Q65
Not enough, The right amount, Too much	Q66
Yes often, Yes sometimes, No	Q67
**About you**	
	Q68
	Q71_AGE_BAND
Male, Female	q69
Deafness or severe hearing impairment, Blindness or partially sighted, A long-standing physical condition, A learning disability, A mental health condition, A long-standing illness such as HIV diabetes chronic heart disease or epilepsy, No I do not have a longstanding condition	Q70A, Q70B, Q70C, Q70D, Q70E,Q70F, Q70G
Less than 1 year, 1 to 5 years, More than 5 years, Don't know / Can't remember	Q71
**Heterosexual / straight (opposite sex), Bisexual (both sexes), Gay or Lesbian (same sex), Other, Prefer not to answer**	**Q72**
Yes and I understand that this does not mean that I would have to take part in the future survey, No I would prefer you not to contact me again	Q73
British, Irish, Any other White background, White and Black Caribbean, White and Black African, White and Asian, Any other mixed background, Indian, Pakistani, Bangladeshi, Any other Asian background, Caribbean, African, Any other Black background, Chinese, Any other ethnic group	Q74
Option to group ethnicities (e.g. white/ non-white/ unknown)	

ve 2 2011/12 Cancer Patient Experience Survey
ients discharged between 01/09/2011 – 30/11/2011

	**Field Name**
	PATIENTID
	TUMOURID
**Seeing your GP**	
None I did not see my GP before going to hospital, I saw my GP once, I saw my GP twice, I saw my GP 3 or 4 times, I saw my GP 5 or more times, Don't know / Can't say	Q1
I was seen as soon as I thought was necessary, I should have been seen a bit sooner, I should have been seen a lot sooner	Q2
Less than 3 months, Don't know / Can't remember, More than 12 months, 6-12 months, 3-6 months	Q3

My health stayed about the same, My health got better, My health got worse	Q4
**Diagnostic tests**	
Yes, No	Q5
Yes completely, Don't know / Can't remember, I did not need an explanation, No but I would have liked an explanation, Yes to some extent	Q6
Yes to some extent, Yes completely, No but I would have liked an explanation, I did not need an explanation, Don't know / Can't remember	Q7
Yes but it was difficult to understand, Yes and it was easy to understand, No but I would have liked written information about the test(s), Don't know / Can't remember, I did not need written information	Q8
I did not need an explanation, No but I would have liked an explanation, Yes completely, Yes to some extent, Don’t know / Can’t remember	Q9
**Finding out what was wrong with you**	
A GP (family doctor), Another health professional, A hospital nurse, A hospital doctor, A friend or relative, Nobody – I worked it out for myself	Q10
No, Yes, It was not necessary, Don’t know / Can’t remember, I was told by phone or letter	Q11
It was done sensitively, It should have been done a bit more sensitively, It should have been done a lot more sensitively	Q12
Yes I completely understood it, No I did not understand it, Yes I understood some of it, Can’t remember	Q13
I did not need written information, No I was not given written information about the type of cancer I had, Yes but it was difficult to understand, Yes and it was easy to understand, Don’t know / Can’t remember	Q14
**Deciding the best treatment for you**	
Not sure / Can’t remember, I was not given a choice because only one type of treatment was suitable for me, No but I would have liked a choice, Yes	Q15
I didn't know my treatment was being discussed by a team of doctors / nurses, No my views were not taken into account, Yes to some extent, Not sure / Can't remember, Yes definitely	Q16
Yes definitely, Yes to some extent, Not sure / Can’t remember, I did not need an explanation, No side effects were not explained	Q17
Yes and it was easy to understand, Yes but it was difficult to understand, No I was not given written information about side effects, Don’t know / Can’t remember	Q18
No but I would like to have been more involved, Yes to some extent, Yes definitely, Not sure / Can't remember	Q19
**Clinical Nurse Specialist**	
Don’t know / Not sure, Yes, No	Q20
Difficult, Sometimes easy sometimes difficult, Easy, I have not tried to contact her/him	Q21
Yes definitely, Yes to some extent, No	Q22
All or most of the time, Some of the time, Rarely or never, I do not ask any questions	Q23
**Support for people with cancer**	
No but I would have liked information, Yes, It was not necessary, Don’t know / Can’t remember	Q24
It was not necessary, No but I would have liked information, Yes, Don't know / Can't remember	Q25
It was not necessary, No but I would have liked information, Yes, Don’t know / Can’t remember	Q26
Yes, No, Don't know / Can't remember	Q27
Yes, No	Q28
Yes, No	Q29
**Operations**	
Yes, No	Q30
No, Yes it was changed once, Yes it was changed 2 or 3 times, Yes it was changed 4 times or more	Q31
Yes completely, Yes to some extent, Don't know / Can't remember, I did not need an explanation, No but I would have liked an explanation	Q32
No I was not given written information about my operation, Yes but it was difficult to understand, Yes and it was easy to understand, Don’t know / Can’t remember	Q33
Yes completely, Yes to some extent, No but I would have liked an explanation, I did not need an explanation`	Q34
**Hospital doctors**	
Yes, No	Q35

Rarely or never, Some of the time, All or most of the time, I did not ask any questions	Q36
In all of them, In some of them, In none of them	Q37
Yes often, Yes sometimes, No	Q38
No, Yes to some extent, Yes definitely, No family or friends were involved, I did not want my family or friends to talk to a doctor, My family did not want or need information	Q39
**Ward Nurses**	
Some of the time, All or most of the time, Rarely or never, I did not ask any questions	Q40
In some of them, In all of them, In none of them	Q41
Yes often, Yes sometimes, No	Q42
There were sometimes enough on duty, There were always or nearly always enough on duty, There were rarely or never enough on duty	Q43
**Hospital care and treatment**	
Often, Never, Only once, Sometimes	Q44
Sometimes, Often, Only once, Never	Q45
Only some of them did, Yes all of them did, None of them did	Q46
Yes always, Yes sometimes, No	Q47
Yes always, Yes sometimes, No	Q48
As much as I wanted, Most of the time, I did not have any worries or fears, Not at all but would have liked to, Some of the time	Q49
All of the time, Some of the time, Not at all, I did not have any pain	Q50
Always, Most of the time, Never, Some of the time	Q51
**formation given to you before you left hospital**	
No, Yes, Can't remember	Q52
Yes, No, Don't know / Can't remember	Q53
No, Yes to some extent, Yes definitely, No family or friends were involved, My family or friends did not want or need information, I did not want my family or friends to be given information	Q54
**Arranging home support**	
Yes definitely, Yes to some extent, No, I did not need help from health or social services, Don't know / Can't remember	Q55
**Hospital care as a day patient/ outpatient**	
No they could have done more, Yes to some extent, Yes definitely, I have not had any side effects from radiotherapy, I have not had radiotherapy	Q56
Yes definitely, Yes to some extent, No they could have done more, I have not had any side effects from chemotherapy, I have not had chemotherapy	Q57
Yes definitely, Yes to some extent, I did not have any pain, No they could have done more	Q58
Yes definitely, No I would have liked more support, Yes to some extent, I did not need emotional support from staff	Q59
**Outpatient appointments with doctors**	
Yes, No	Q60
Seen on time or early, Waited 6 - 15 minutes, Waited up to 5 minutes, Waited 16 - 30 minutes, Waited 31 - 60 minutes, Waited 1 to 2 hours, Waited more than 2 hours, Don't know / Can't remember	Q61
Too short, Too long, About right	Q62
Don't know / Can't remember, Yes, No	Q63
**Care from your general practice**	
Yes, No, Don't know / Can't remember	Q64
Yes to some extent, No they could have done more, My general practice was not involved, Yes definitely	Q65
**Your overall NHS care**	
Don't know, Yes most of the time, Yes some of the time, No never, Yes always	Q66
The right amount, Too much, Not enough	Q67
Yes, No, Don't know / Can't remember	Q68
Yes often, Yes sometimes, No	Q69
Excellent, Very good, Good, Fair, Poor	Q70
**About you**	
	Q71
	Q71_AGE_BAND
Male, Female	Q72

For peer review only

**Heterosexual / straight (opposite sex), Prefer not to answer, Other, Gay or Lesbian (same sex), Bisexual (both sexes)**	**Q73**
Deafness or severe hearing impairment, Blindness or partially sighted, A long-standing physical condition, A learning disability, A mental health condition, A long-standing illness such as HIV diabetes chronic heart disease or epilepsy, No I do not have a longstanding condition	Q74A, Q74B, Q74C, Q74D, Q74E, Q74F, Q74G
1 to 5 years, Less than 1 year, More than 5 years, Don't know / Can't remember	Q75
Yes and I understand that this does not mean that I would have to take part in the future survey, No I would prefer you not to contact me again	Q76
British, Any other ethnic group, Any other White background, White and Black Caribbean, White and Black African, White and Asian, Any other mixed background, Indian, Pakistani, Bangladeshi, Any other Asian background, Caribbean, African, Any other Black background, Chinese, Irish	Q77
Option to group ethnicities (e.g. white/ non-white/ unknown)	

/ave 3 2013 Cancer Patient Experience Survey
ients discharged between 01/09/2012 – 30/09/2012

	**Field Name**
	PATIENTID
	TUMOURID
**Seeing your GP**	
None - I did not see my GP before going to hospital, I saw my GP once, I saw my GP twice, I saw my GP 3 or 4 times, I saw my GP 5 or more times, Don't know / can't say	Q1
I was seen as soon as I thought was necessary, I should have been seen a bit sooner, I should have been seen a lot sooner	Q2
Less than 3 months, 3-6 months, 6-12 months, More than 12 months, Don't know / can't remember	Q3
My health got worse, My health got better, My health stayed about the same	Q4
**Diagnostic tests**	
Yes, No	Q5
Yes, completely, Yes, to some extent, No, but I would have liked an explanation, I did not need an explanation, Don't know / can't remember	Q6
Yes, completely, Yes, to some extent, No, but I would have liked an explanation, I did not need an explanation, Don't know / can't remember	Q7
Yes, and it was easy to understand, Yes, but it was difficult to understand, No, but I would have liked written information about the test(s), I did not need written information, Don't know / can't remember	Q8
Yes, completely, Yes, to some extent, No, but I would have liked an explanation, I did not need an explanation, Don't know / can't remember	Q9
**Finding out what was wrong with you**	
A hospital doctor, A hospital nurse, A GP (family doctor), Another health professional, A friend or relative, Nobody ? I worked it out for myself	Q10
Yes, No, It was not necessary, I was told by phone or letter, Don't know / can't remember	Q11
It was done sensitively, It should have been done a bit more sensitively, It should have been done a lot more sensitively	Q12
Yes, I completely understood it, Yes, I understood some of it, No, I did not understand it, Can't remember	Q13
Yes, and it was easy to understand, Yes, but it was difficult to understand, No, I was not given written information about the type of cancer I had, I did not need written information, Don't know / can't remember	Q14
**Deciding the best treatment for you**	
Yes, No, but I would have liked a choice, I was given a choice because only one type of treatment was suitable for me, Not sure / can't remember	Q15
Yes, definitely, Yes, to some extent, No, my views were not taken into account, I didn't know my treatment was being discussed by a team of doctors / nurses, Not sure / can't remember	Q16
Yes, definitely, Yes, to some extent, No, side effects were not explained, I did not need an explanation, Not sure / can't remember	Q17
Yes, and it was easy to understand, Yes, but it was difficult to understand, No, I was not given written information about side effects, Don't know / can't remember	Q18

For peer review only

Yes, definitely, Yes, to some extent, No, future side effects were not explained, I did not need an explanation, Not sure / can't remember	Q19
Yes, definitely, Yes, to some extent, No, but I would like to have been more involved, Not sure / can't remember	Q20
**Clinical Nurse Specialist**	
Yes, No, Don't know / not sure	Q21
Easy, Sometimes easy, sometimes difficult, Difficult, I have not tried to contact her/him	Q22
Yes, definitely, Yes, to some extent, No	Q23
All or most of the time, Some of the time, Rarely or never, I do not ask any questions	Q24
**Support for people with cancer**	
Yes, No, but I would have liked information, It was necessary, Don't know / can't remember	Q25
Yes, No, but I would have liked a discussion or information, It was not necessary / relevant to me, Don't know / can't remember	Q26
Yes, No, but I would have liked information, It was not necessary, Don't know / can't remember	Q27
Yes, No, but I would have liked information, It was not necessary, Don't know / can't remember	Q28
Yes, No	Q29
Yes, No, Don't know / can't remember	Q30
Yes, No	Q31
**Operations**	
Yes, No	Q32
Yes, completely, Yes, to some extent, No, but I would have liked an explanation, I did not need an explanation, Don't know / can't remember	Q33
Yes, and it was easy to understand, Yes, but it was difficult to understand, No, I was not given written information about my operation, Don't know / can't remember	Q34
Yes, completely, Yes, to some extent, No, but I would have liked an explanation, I did not need an explanation	Q35
**Hospital doctors**	
Yes, No	Q36
All or most of the time, Some of the time, Rarely or never, I did not ask any questions	Q37
In all of them, In some of them, In none of them	Q38
Yes, often, Yes, sometimes, No	Q39
Yes, definitely, Yes, to some extent, No, No family or friends were involved, My family did not want or need information, I did not want my family or friends to talk to a doctor	Q40
**Ward nurses**	
All or most of the time, Some of the time, Rarely or never, I did not ask any questions	Q41
In all of them, In some of them, In none of them	Q42
Yes, often, Yes, sometimes, No	Q43
There were always or nearly always enough on duty, There were sometimes enough on duty, There were rarely or never enough on duty	Q44
**Hospital care and treatment**	
Often, Sometimes, Only once, Never	Q45
Often, Sometimes, Only once, Never	Q46
Yes, all of them did, Only some of them did, None of them did	Q47
Yes, always, Yes, sometimes, No	Q48
Yes, sometimes, Yes, sometimes, No	Q49
As much as I wanted, Most of the time, Some of the time, Not at all, but would have liked to, I did not have any worries or fears	Q50
All of the time, Some of the time, Not at all, I did not have any pain	Q51
Always, Most of the time, Some of the time, Never	Q52
Yes, No, Can't remember	Q53
Yes, No, Don't know / can't remember	Q54
Yes, definitely, Yes, to some extent, No, No family or friends were involved, My family or friends did not want or need information, I did not want my family or friends to be given information	Q55
**Arranging home support**	
Yes, definitely, Yes, to some extent, No, I did not need help from health or social services, Don't know / can't remember	Q56

For peer review only

**Hospital care as day patient/ outpatient**	
Yes, definitely, Yes, to some extent, No, they could have done more, I have not had any side effects from radiotherapy, I have not had radiotherapy	Q57
Yes, definitely, Yes, to some extent, No, they could have done more, I have not had any side effects from chemotherapy, I have not had chemotherapy	Q58
Yes, definitely, Yes, to some extent, No, they could have done more, I did not have any pain	Q59
Yes, definitely, Yes, to some extent, No, I would have liked more support, I did not need emotional support from staff	Q60
**Outpatient appointments with doctors**	
Yes, No	Q61
Yes, No, Don't know / can't remember	Q62
**Care from your general practice**	
Yes, No, Don't know / can't remember	Q63
Yes, definitely, Yes, to some extent, No, they could have done more, My general practice was not involved	Q64
**Your overall NHS care**	
Yes, always, Yes, most of the time, Yes, some of the time, No, never, Don't know	Q65
Physiotherapist	Q66A
Occupational therapist (OT)	Q66B
Dietician	Q66C
Speech and language therapist	Q66D
Lymphoedema specialist	Q66E
Not enough, The right amount, Too much	Q67
Yes, No, Don't know / can't remember	Q68
Yes, often, Yes, sometimes, No	Q69
Excellent, Very good, Good, Fair, Poor	Q70
**About you**	
	Q71
	Q71_AGE
	Q71_AGE_BAND
Male, Female	Q72
**Heterosexual / straight (opposite sex), Bisexual (both sexes), Gay or Lesbian (same sex), Other, Prefer not to answer**	**Q73**
Full time employment, Part time employment, Homemaker, Student (in education), Retired, Unemployed - and seeking work, Unemployed - unable to work for health reasons, Other	Q74
Deafness or severe hearing impairment, Deafness or severe hearing impairment, Blindness or partially sighted, A long-standing physical condition, A learning disability, A mental health condition, A long-standing illness, such as cancer, HIV, diabetes, chronic heart disease, or epilepsy, No, I do not have a long-standing condition	Q75A, Q75B, Q75C, Q75D, Q75E, Q75F, Q75G
Less than 1 year, 1 to 5 years, More than 5 years, Don't know / can't remember	Q76
My cancer has responded fully to treatment (I have no signs or symptoms of cancer), My cancer has been treated but is still present, My cancer has not been treated at all, My cancer has come back after it was originally treated, My original cancer responded but I now have a new cancer, I am not certain what is happening with my cancer	Q77
Yes, and I understand that this does not mean that I would have to take part in the future survey, No, I would prefer you not to contact me again	Q78
British, Irish, Any other White background, White and Black Caribbean, White and Black African, White and Asian, Any other mixed background, Indian, Pakistani, Bangladeshi, Any other Asian background, Caribbean, African, Any other Black background, Chinese, Any other ethnic background	Q79
Option to group ethnicities (e.g. white/ non-white/ unknown)	

/ave 4 2014 Cancer Patient Experience Survey
ients discharged between 01/09/2013 – 30/11/2013

	**Field Name**
	PATIENTID
	TUMOURID

For peer review only

**Seeing your GP**	
None - I didn't see my GP before going to hospital, I saw my GP once, I saw my GP twice, I saw my GP 3 or 4 times, I saw my GP 5 or more times, Don't know / can't say	Q1
I was seen as soon as I thought was necessary, I should have been seen a bit sooner, I should have been seen a lot sooner	Q2
Less than 3 months, 3-6 months, 6-12 months, More than 12 months, Don't know / can't remember	Q3
My health got worse, My health got better, My health stayed about the same	Q4
Yes, No	Q5
**Diagnostic tests**	
Yes, completely, Yes, to some extent, No, but I would have liked an explanation, I did not need an explanation, Don't know / can't remember	Q6
Yes, completely, Yes, to some extent, No, but I would have liked an explanation, I did not need an explanation, Don't know / can't remember	Q7
Yes, and it was easy to understand, Yes, but it was difficult to understand, No, but I would have liked written information about the test(s), I did not need written information, Don't know / can't remember	Q8
Yes, completely, Yes, to some extent, No, but I would have liked an explanation, I did not need an explanation, Don't know / can't remember	Q9
**Finding out what was wrong with you**	
A hospital doctor, A hospital nurse, A GP (family doctor), Another health professional, A friend or relative, Nobody – I worked it out for myself	Q10
Yes, No, It was not necessary, I was told by phone or letter, Don't know / can't remember	Q11
It was done sensitively, It should have been done a bit more sensitively, It should have been done a lot more sensitively	Q12
Yes, I completely understood it, Yes, I understood some of it, No, I did not understand it, Can't remember	Q13
Yes, and it was easy to understand, Yes, but it was difficult to understand, No, I was not given written information about the type of cancer I had, I did not need written information, Don't know / can't remember	Q14
**Deciding the best treatment for you**	
Yes, No, but I would have liked a choice, I was given a choice because only one type of treatment was suitable for me, Not sure / can't remember	Q15
Yes, definitely, Yes, to some extent, No, my views were not taken into account, I didn't know my treatment was being discussed by a team of doctors / nurses, Not sure / can't remember	Q16
Yes, definitely, Yes, to some extent, No, side effects were not explained, I did not need an explanation, Not sure / can't remember	Q17
Yes, and it was easy to understand, Yes, but it was difficult to understand, No, I was not given written information about side effects, Don't know / can't remember	Q18
Yes, definitely, Yes, to some extent, No, future side effects were not explained, I did not need an explanation, Not sure / can't remember	Q19
Yes, definitely, Yes, to some extent, No, but I would like to have been more involved, Not sure / can't remember	Q20
**Clinical Nurse Specialist**	
Yes, No, Don't know / not sure	Q21
Easy, Sometimes easy, sometimes difficult, Difficult, I have not tried to contact her/him	Q22
Yes, definitely, Yes, to some extent, No	Q23
All or most of the time, Some of the time, Rarely or never, I do not ask any questions	Q24
**Support for people with cancer**	
Yes, No, but I would have liked information, It was necessary, Don't know / can't remember	Q25
Yes, No, but I would have liked a discussion or information, It was not necessary / relevant to me, Don't know / can't remember	Q26
Yes, No, but I would have liked information, It was not necessary, Don't know / can't remember	Q27
Yes, No, but I would have liked information, It was not necessary, Don't know / can't remember	Q28
Yes, No	Q29
Yes, No, Don't know / can't remember	Q30
Yes, No	Q31
**Operations**	

For peer review only

Yes, No	Q32
Yes, completely, Yes, to some extent, No, but I would have liked an explanation, I did not need an explanation, Don't know / can't remember	Q33
Yes, and it was easy to understand, Yes, but it was difficult to understand, No, I was not given written information about my operation, Don't know / can't remember	Q34
Yes, completely, Yes, to some extent, No, but I would have liked an explanation, I did not need an explanation	Q35
**Hospital doctors**	
Yes, No	Q36
All or most of the time, Some of the time, Rarely or never, I did not ask any questions	Q37
In all of them, In some of them, In none of them	Q38
Yes, often, Yes, sometimes, No	Q39
Yes, definitely, Yes, to some extent, No, No family or friends were involved, My family did not want or need information, I did not want my family or friends to talk to a doctor	Q40
**Ward Nurses**	
All or most of the time, Some of the time, Rarely or never, I did not ask any questions	Q41
In all of them, In some of them, In none of them	Q42
Yes, often, Yes, sometimes, No	Q43
There were always or nearly always enough on duty, There were sometimes enough on duty, There were rarely or never enough on duty	Q44
**Hospital care and treatment**	
Often, Sometimes, Only once, Never	Q45
Often, Sometimes, Only once, Never	Q46
Yes, all of them did, Only some of them did, None of them did	Q47
Yes, always, Yes, sometimes, No	Q48
Yes, sometimes, Yes, sometimes, No	Q49
As much as I wanted, Most of the time, Some of the time, Not at all, but would have liked to, I did not have any worries or fears	Q50
All of the time, Some of the time, Not at all, I did not have any pain	Q51
Always, Most of the time, Some of the time, Never	Q52
Yes, No, Can't remember	Q53
Yes, No, Don't know / can't remember	Q54
**Home care and support**	
Yes, definitely, Yes, to some extent, No, No family or friends were involved, My family or friends did not want or need information, I did not want my family or friends to be given information	Q55
Yes, definitely, Yes, to some extent, No, I did not need help from health or social services, Don't know / can't remember	Q56
**Hospital care as day patient/ outpatient**	
Yes, definitely, Yes, to some extent, No, they could have done more, I have not had any side effects from radiotherapy, I have not had radiotherapy	Q57
Yes, definitely, Yes, to some extent, No, they could have done more, I have not had any side effects from chemotherapy, I have not had chemotherapy	Q58
Yes, definitely, Yes, to some extent, No, they could have done more, I did not have any pain	Q59
Yes, definitely, Yes, to some extent, No, I would have liked more support, I did not need emotional support from staff	Q60
**Outpatient appointments with doctors**	
Yes, No	Q61
Yes, No, Don't know / can't remember	Q62
**Care from your general practice**	
Yes, No, Don't know / can't remember	Q63
Yes, definitely, Yes, to some extent, No, they could have done more, My general practice was not involved	Q64
**Your overall NHS care**	
Yes, always, Yes, most of the time, Yes, some of the time, No, never, Don't know	Q65
Physiotherapist	Q66A
Occupational therapist (OT)	Q66B
Dietician	Q66C

For peer review only

Speech and language therapist	Q66D
Lymphoedema specialist	Q66E
Not enough, The right amount, Too much	Q67
Yes, No, Don't know / can't remember	Q68
Yes, often, Yes, sometimes, No	Q69
Excellent, Very good, Good, Fair, Poor	Q70
**About you**	
	Q71
	Q71_AGE
	Q71_AGE_BAND
Male, Female	Q72
**Heterosexual / straight (opposite sex), Bisexual (both sexes), Gay or Lesbian (same sex), Other, Prefer not to answer**	**Q73**
Full time employment, Part time employment, Homemaker, Student (in education), Retired, Unemployed - and seeking work, Unemployed - unable to work for health reasons, Other	Q74
Deafness or severe hearing impairment, Blindness or partially sighted, A long-standing physical condition, A learning disability, A mental health condition, A long-standing illness, such as cancer, HIV, diabetes, chronic heart disease, or epilepsy, No, I do not have a long-standing condition	Q75A, Q75B,Q75C, Q75D, Q75E, Q75F, Q75G
Less than 1 year, 1 to 5 years, More than 5 years, Don't know / can't remember	Q76
Yes, this is the first time I have been treated for cancer, No, I have been treated for the same type of cancer before but it has now come back, No, I have been treated for a different type of cancer before	Q77
Treatment has not yet started for this cancer, I am in the course of treatment and I can't tell yet how my cancer has responded, The treatment has been effective and I have no signs or symptoms of cancer, I have finished the course of treatment but my cancer is still present, My cancer is being treated again because it has not responded fully to treatment, I am not in active treatment but I am on "Watch and Wait", My cancer has not been treated at all	Q78
Yes, and I understand that this does not mean that I would have to take part in the future survey, No, I would prefer you not to contact me again	Q79
English / Welsh / Scottish / Northern Irish / British, Irish, Gypsy or Irish Traveller, Any other White background, White and Black Caribbean, White and Black African, White and Asian, Any other Mixed / multiple ethnic background, Indian, Pakistani, Bangladeshi, Chinese, Any other Asian background, African, Caribbean, Any other Black / African / Caribbean background	Q80
Option to group ethnicities (e.g. white/ non-white/ unknown)	

/ave 5 2015 Cancer Patient Experience Survey
ients discharged between 01/04/2015 - 30/06/2015

	**Field Name**
	PATIENTID
	TUMOURID
**Seeing your GP**	
None - I went straight to hospital, None - I went to hospital following a cancer screening appointment , I saw my GP once, I saw my GP twice, I saw my GP 3 or 4 times, I saw my GP 5 or more times , Don't know / can't remember	Q1
I was seen as soon as I thought was necessary, I should have been seen a bit sooner, I should have been seen a lot sooner	Q2
Less than 3 months, 3-6 months, 6-12 months , More than 12 months, Don't know / can't remember	Q3
**Diagnostic tests**	
Yes, No	Q4
Yes, No - I would have liked more written information, No- I would have liked more verbal information, I did not need / want any information, Don't know / can't remember	Q5
It was about right, It was a little too long, It was much too long, Don't know / can't remember	Q6
Yes- completely, Yes- to some extent, No - I did not understand the explanation, I did not have an explanation but would have liked one, I did not need an explanation, Don't know / can't remember	Q7

For peer review only

**Finding out what was wrong with you**	
Yes, No, It was not necessary, I was told by phone or letter, Don't know / can't remember	Q8
It was done sensitively, It should have been done a bit more sensitively, It should have been done a lot more sensitively	Q9
Yes - I completely understood it, Yes-, I understood some of it, No- I did not understand it, Don't know / can't remember	Q10
Yes- and it was easy to understand, Yes- but it was difficult to understand, No- I was not given written information about the type of cancer I had, I did not need written information, Don't know / can't remember	Q11
**Deciding the best treatment for you**	
Yes- completely, Yes- to some extent, No, There was only one type of treatment that was suitable for me, Don't know / can't remember	Q12
Yes – definitely, Yes- to some extent, No- side effects were not explained, I did not need an explanation, Don't know / can't remember	Q13
Yes –definitely, Yes- to some extent, No- I was not offered any practical advice or support, Don't know / can't remember	Q14
Yes – definitely, Yes- to some extent, No- future side effects were not explained, I did not need an explanation, Don't know / can't remember	Q15
Yes- definitely, Yes- to some extent, No- but I would like to have been more involved, Don't know / can't remember	Q16
**Clinical Nurse Specialist**	
Yes, No , Don't know / can't remember	Q17
Very easy, Quite easy, Neither easy nor difficult, Quite difficult, Very difficult, I have not tried to contact my Clinical Nurse Specialist	Q18
All or most of the time, Some of the time, Rarely or never, I have not asked any questions	Q19
**Support for people with cancer**	
Yes, No- but I would have liked information, It was not necessary, Don't know / can't remember	Q20
Yes, No - but I would have liked a discussion or information, It was not necessary / relevant to me, Don't know / can't remember	Q21
Yes, No- but I would have liked information, It was not necessary, Don't know / can't remember	Q22
Yes, No- but I would have liked information, It was not necessary, Don't know / can't remember	Q23
**Operations**	
Yes, No	Q24
Yes, No - I would have liked more written information, No - I would have liked more verbal information, I did not need / want information	Q25
Yes - completely, Yes - to some extent, No- but I would have liked an explanation, I did not need an explanation	Q26
**Hospital care as an inpatient**	
Yes, No	Q27
Yes – often, Yes – sometimes, No	Q28
Yes- in all of them, Yes- in some of them, No- in none of them	Q29
Yes- definitely, Yes- to some extent, No- No family or friends were involved, My family did not want to talk to a doctor, I did not want my family or friends to talk to a doctor	Q30
Yes - in all of them, Yes -in some of them, No, in none of them	Q31
There were always or nearly always enough on duty, There were sometimes enough on duty, There were rarely or never enough on duty	Q32
Yes - all of them did, Only some of them did, None of them did	Q33
Yes –always, Yes – sometimes, No	Q34
Yes – definitely, Yes - to some extent, No, I had no worries or fears	Q35
Yes – definitely, Yes - to some extent, No, I did not have any pain	Q36

For peer review only

Yes – always, Yes – sometimes, No	Q37
Yes, No, Don't know/can't remember	Q38
Yes, No, Don't know/can't remember	Q39
**Hospital care as a day patient / outpatient**	
Yes, No	Q40
Yes – definitely, Yes - to some extent, No, I had no worries or fears	Q41
Yes, No, I didn't have an appointment with a cancer doctor, Don't know / can't remember	Q42
Yes, No	Q43
Yes – completely, Yes - to some extent, No, I did not need any information	Q44
Yes – completely, Yes - to some extent, No, It is too early to know if my radiotherapy is working, I did not need any information	Q45
Yes, No	Q46
Yes – completely, Yes - to some extent, No, I did not need any information	Q47
Yes – completely, Yes - to some extent, No, It is too early to know if my chemotherapy is working, I did not need any information	Q48
**Home Care Support**	
Yes –definitely, Yes - to some extent, No, No family or friends were involved, My family or friends did not want or need information, I did not want my family or friends to be involved	Q49
Yes – definitely, Yes - to some extent, No, I did not need help from health or social services, Don't know / can't remember	Q50
Yes – definitely, Yes - to some extent, No, I did not need help from health or social services, Don't know / can't remember	Q51
Yes, No, Don't know/can't remember	Q52
Yes – definitely, Yes - to some extent, No -they could have done more,My general practice was not involved	Q53
Yes – always, Yes - most of the time, Yes- some of the time, No – never, Don't know / can't remember	Q54
Yes, No, I don't know what a care plan is, Don't know/can't remember	Q55
Very good, Good, Neither good nor bad, Quite bad, Very bad, Don't know / can't remember	Q56
It was much too long, It was a little too long, It was about right, Don't know / can't remember	Q57
Yes, No, No, but I would have liked them to, Don't know / can't remember	Q58
Scale 1-10	Q59
Less than 1 year, 1 to 5 years, More than 5 years, Don't know/ can't remember	Q60
Yes, No, Don't know, Does not apply to my type of cancer	Q61
My cancer has been taken out / treated, without any sign of further problem; My cancer was taken out / treated without any sign of further problem, but has since come back / spread to other parts of my body; None of the above options apply to my type of cancer; I would prefer not to say; I don't know	Q62
**About You**	
	Q63
	Q63_AGE
	Q63_AGE_BAND
Male, Female	Q64
**Heterosexual or Straight, Gay or Lesbian, Bisexual, Other, Prefer not to say**	**Q65**

Deafness or severe hearing impairment, Blindness or partially sighted, A long-standing physical condition, A learning disability, A mental health condition, A long-standing illness (such as HIV, diabetes, chronic heart disease, or epilepsy)	Q66
Yes - and I understand that this does NOT mean that I would have to take part in the future survey, No - I would prefer you not to contact me again	Q67
Yes, No	Q68
English / Welsh / Scottish / Northern Irish / British, Irish, Gypsy or Irish Traveller, Any other White background, White and Black Caribbean, White and Black African, White and Asian, Any other Mixed / multiple ethnic background, Indian, Pakistani, Bangladeshi, Chinese, Any other Asian background, African, Caribbean, Any other Black / African / Caribbean background, Arab, Any other ethnic group	Q69
Option to group ethnicities (e.g. white/ non-white/ unknown)	

************************* **End of sheet** *************************

For peer review only

ted to an acute or specialist NHS
eir initial GP visit prior to diagnosis,

sitive patient demographic data,
nese data to provide a less disclosive

Request Field	Justification

Request I	Justification

**Request I**	**Justification**

Request I	Justification

Request I	Justification

For peer review only - http://bmjopen.bmj.com/site/about/guidelines.xhtml

For peer review only - http://bmjopen.bmj.com/site/about/guidelines.xhtml

For peer review only

Introduction

The National Cancer Waiting Times Monitoring Data Set s

- A maximum two week wait from an urgent GP referral fo
- A maximum one month (31-day) wait from diagnosis (CA
- A maximum two month (62-day) wait from urgent GP ref
- A maximum one month (31-day) wait from urgent GP ref
- A maximum 62-day wait from referral from a cancer Scre
- A maximum 62-day wait from a CONSULTANTS decisio
- A maximum 31-day wait for all subsequent treatments fo
- A maximum two week wait from referral for breast sympt

Data Coverage

2009-2015 CWT treatment data (linked to CAS AV tables

Data items selection

Please be aware that selecting patient identifiable data ite organisation. Sensitive patient demographic data, event d design and default principles have also been applied to sc

Key:

**Items in red**	Identifiable fields that require additional per
	Sensitive items that require additional justifi
	Privacy by design and default' alternatives t

Item Number	Data Item
**1**	Pseudonymised patient ID
**2**	**NHS Number**
**3**	**Patient Pathway Identifier**
**4**	Organisation code (patient pathway identifier issuer)
**5**	Pseudonymised organisation code
**6**	Source of referral for out-patients
**7**	Priority type code
**8**	Decision to refer date (cancer or breast symptoms)
**9**	Month of decision to refer
**10**	Year of decision to refer
**11**	Days from another event to decision to refer date
**12**	Cancer referral to treatment period start date
**13**	Month of cancer referral to treatment period
**14**	Year of cancer referral to treatment period
**15**	Days from another event to treatment period start date

**16**	Two week wait cancer or symptomatic breast referral type
**17**	Consultant upgrade date
**18**	Month of consultant upgrade
**19**	Year of consultant upgrade
**20**	Days from another event to consultant upgrade date
**21**	Site code (of provider consultant update)
**22**	Site code (of provider consultant update) (Pseudonymised)
**23**	Date first seen
**24**	Month first seen
**25**	Year first seen
**26**	Days from another event to date first seen
**27**	Site code (of provider first seen)
**28**	Site code (of provider first seen) (pseudonymised)
**29**	Waiting time adjustment (first seen)
**30**	Multidisciplinary team discussion indicator
**31**	Multidisciplinary team discussion date (cancer)
**32**	Month of MDT discussion
**33**	Year of MDT discussion
**34**	Days from another event to multidisciplinary team discussion date
**54**	Cancer or symptomatic breast referral patient status
**55**	Primary diagnosis (ICD)
**56**	Metastatic site
**57**	Tumour laterality
**58**	Cancer treatment period start date
**59**	Month of cancer treatment period
**60**	Year of cancer treatment period
**61**	Days from another event to cancer treatment period start date
**62**	Site code (of provider cancer decision to treat)
**63**	Pseudonymised site code (of provider cancer decision to treat)
**64**	Referral request received date (inter-provider transfer)
**65**	Month of referral request received

**66**	Year of referral request received
**67**	Days from another event to referral request received date
**68**	Practice code (at cancer treatment period start date)
**69**	Pseudonymised practice code (at cancer treatment period start date)
**70**	Cancer site grouping (broad)
**71**	Cancer site grouping (specific)
**72**	Duplicate flag
**73**	Two week wait detection indicator (same site)
**74**	Two week wait detection indicator (any site)
**75**	Treatment start date (cancer)
**76**	Month treatment started
**77**	Year treatment started
**78**	Days from another event to the treatment start date
**79**	Site code (of provider cancer treatment start date)
**80**	Pseudonymised site code (of provider cancer treatment start date)
**81**	Cancer treatment event type
**82**	Cancer treatment modality
**83**	Clinical trial indicator
**84**	Cancer care setting (treatment)
**85**	Radiotherapy intent
**86**	Radiotherapy priority
**87**	Waiting time adjustment (treatment)

************************* **End of sheet** ********

review only

supports the continued management and monitc

ɔr suspected cancer to DATE FIRST SEEN by a
ANCER TREATMENT PERIOD START DATE) t
ferral for suspected cancer to First Definitive Tre
ferral for suspected cancer to First Definitive Tre
eening Programme to first treatment for all canc
ɔn to upgrade the urgency of a PATIENT they su
ɔr new cases of primary and recurrent cancer wh
toms (where cancer is not initially suspected) to

for 2009-2014 registrations)

ems – **the data items marked below in red** – w
dates and geographies will be treated as de-pers
ome of these data to provide a less disclosive al

rmissions to access (as outlined above)
fication in your protocol to access
to the sensitive items highlighted in dark grey

**Field Name**
PATIENTID
**NHSNO**
**PPI**
ORG_PPI
ORG_PPI (pseudonymised)
One occu
REF_SOURCE
PRIORITY_TYPE
DEC_TO_REF_DATE
Derived from DEC_TO_REF_DATE
Derived from DEC_TO_REF_DATE
Derived from DEC_TO_REF_DATE
CRTP_DATE
Derived from CRTP_DATE field
Derived from CRTP_DATE field
Derived from CRTP_DATE field

eer review only

REF_TYPE
CONS_UPGRADE_DATE
Derived from CONS_UPGRADE_DATE
Derived from CONS_UPGRADE_DATE
Derived from CONS_UPGRADE_DATE
ORG_CONS_UPGRADE
ORG_CONS_UPGRADE (pseudonymised)
DATE_FIRST_SEEN
Derived from DATE_FIRST_SEEN field
Derived from DATE_FIRST_SEEN field
Derived from DATE_FIRST_SEEN field
ORG_FIRST_SEEN
ORG_FIRST_SEEN (pseudonymised)
WTA_FIRST_SEEN
MDT_IND
MDT_DATE
Derived from MDT_DATE field
Derived from MDT_DATE field
Derived from MDT_DATE field
PATIENT_STATUS
DIAG
METS_SITE
LATERALITY
TREAT_PERIOD_START
Derived from TREAT_PERIOD_START
Derived from TREAT_PERIOD_START
Derived from TREAT_PERIOD_START
ORG_DEC_TO_TREAT
ORG_DEC_TO_TREAT (pseudonymised)
REF_REQUEST_RECEIVED
Derived from REF_REQUEST_RECEIVED

For peer review only

Derived from REF_REQUEST_RECEIVED
Derived from REF_REQUEST_RECEIVED
**DERIVED INF**
practice_code
practice_code (pseudonymised)
diag_group_broad
diag_group_specific
dup_flag
detected_same_site
detected_any_site
One occur
TREAT_START
Derived from TREAT_START field
Derived from TREAT_START field
Derived from TREAT_START field
ORG_TREAT_START
ORG_TREAT_START (pseudonymised)
CTE_TYPE
MODALITY
CLIN_TRIAL
CARE_SETTING
RADIO_INTENT
RADIO_PRIORITY
WTA_TREAT

****

›ring of the following waiting times:

specialist for all suspected cancers
to First Definitive Treatment for all cancers
›atment for all cancers
›atment for children's cancers, testicular cancers and acute leukaemia
ers
ıspect to have cancer to first treatment for all cancers
here an Anti-Cancer Drug Regimen, surgery or Radiotherapy is the chosen CANCER TREATMENT MODALITY;
DATE FIRST SEEN

ill require you to have patient consent, section 251 approval, or the appropriate statutory regulation covering your
sonalised, unless anonymisation techniques are applied to render the data anonymised to k-3 anonymity. Privacy by
lternative. The key explaining how these alternative can be selected is included below:

Definition	Request Data Item
**PATIENT AND PATHWAY IDENTIFICATION**<br>To carry Patient and Pathway details.<br>One occurrence of this group is required.	
Project specific patient ID	
**The NHS NUMBER, the primary identifier of a PERSON, is a unique identifier for a PATIENT within the NHS in England and Wales.**	
**An identifier, which together with the ORGANISATION CODE / ORGANISATION IDENTIFIER of the issuer, uniquely identifies a PATIENT PATHWAY.**	
The ORGANISATION CODE of the Organisation issuing the PATIENT PATHWAY IDENTIFIER.	
The pseudonymised ORGANISATION CODE issuing the PATIENT PATHWAY IDENTIFIER.	
**OUTPATIENT SERVICES**<br>To carry Outpatient Services details.<br>rrence of this group is required if applicable to the scenario.	
The source of referral of each Consultant Out-Patient Episode.	
The priority of a request for SERVICES.	
The DATE on which a decision was made to refer the PATIENT to Secondary Care with either suspected cancer, or as an urgent symptomatic breast referral.	
Month of decision to refer	
Year of decision to refer	
Option to provide number of days from another event to decision to refer date (e.g. number of days from decision to refer date to diagnosis)	
The Start Date of a Cancer Referral To Treatment Period.	
Month of cancer referral to treatment period	
Year of cancer referral to treatment period	
Number of days from another event to decision to refer date (e.g. number of days from a cancer referral to diagnosis)	

The site where cancer is suspected by the GENERAL MEDICAL PRACTITIONER or GENERAL DENTAL PRACTITIONER on referral, or, for PATIENTS who are upgraded to an urgent breast cancer PATIENT PATHWAY, identifies that the PATIENT was initially referred on the basis of exhibited (non-cancer) breast symptoms.	
The DATE that the CONSULTANT responsible for the care of the PATIENT (or an authorised member of the CONSULTANT team as defined by local policy) decided that the PATIENT should be upgraded onto an urgent Cancer PATIENT PATHWAY.	
Month of consultant upgrade	
Year of consultant upgrade	
Option to provide number of days from another event to decision to consultant upgrade date (e.g. number of days from decision to consultant upgrade date to diagnosis)	
The ORGANISATION SITE CODE of the Organisation acting as Health Care Provider when a decision is made to upgrade the PATIENT to an urgent Cancer PATIENT PATHWAY.	
The pseudonymised ORGANISATION SITE CODE of the Organisation acting as Health Care Provider when a decision is made to upgrade the PATIENT to an urgent Cancer PATIENT PATHWAY.	
The date that the PATIENT is first seen in the Trust that receives the first referral.	
Month first seen	
Year first seen	
Option to provide number of days from a different event to the date first seen (e.g. days from date first seen to diagnosis)	
The ORGANISATION SITE CODE of the Health Care Provider at the first contact with the PATIENT.	
The pseudonymised ORGANISATION SITE CODE of the Health Care Provider at the first contact with the PATIENT.	
The number of days that should be removed from the derived waiting time between the CANCER REFERRAL TO TREATMENT PERIOD START DATE and DATE FIRST SEEN.	
**MULTIDISCIPLINARY TEAM ACTIVITY**<br>To carry Multidisciplinary Team Activity details.<br>One occurrence of this group is required.	
An indication of whether the PATIENT's CARE PLAN was discussed at a Multidisciplinary Team Meeting.	
The date on which the PATIENT's Cancer Care Plan was discussed at a Multidisciplinary Team Meeting and a treatment planning decision was made.	
Month of MDT discussion	
Year of MDT discussion	
Option to provide number of days from a different event to the MDT discussion date (e.g. days from MDT discussion date to diagnosis)	
**PATIENT STATUS AND DIAGNOSIS**<br>To carry Patient Status and Diagnosis details.<br>One occurrence of this group is required.	
Recorded to enable tracking of the status of REFERRAL REQUESTS for PATIENTS referred with a suspected cancer, or referred with breast symptoms with cancer not originally suspected.	
The International Classification of Diseases (ICD) code used to identify the PRIMARY DIAGNOSIS.	
The METASTATIC SITE at the point of treatment.	
The side of the body for a Tumour relating to paired organs within a PATIENT.	
The DATE when a Cancer Treatment Period is started.	
Month of cancer treatment period	
Year of cancer treatment period	
Option to provide number of days from a different event to the cancer treatment period start date (e.g. days from diagnosis to cancer treatment period start date)	
The ORGANISATION SITE CODE of the Organisation acting as Health Care Provider where the decision to treat the PATIENT was made which initiated a Cancer Care Plan with one or more Planned Cancer Treatments.	
The pseudonymised ORGANISATION SITE CODE of the Organisation acting as Health Care Provider where the decision to treat the PATIENT was made which initiated a Cancer Care Plan with one or more Planned Cancer Treatments.	
The date the Organisation where the PATIENT is being transferred to, received the SERVICE REQUEST from the referring Organisation.	
Month of referral request received	

Year of referral request received	
Option to provide number of days from a different event to the referral request received date (e.g. days from the referral request received date to diagnosis)	
**FORMATION ABOUT PATIENT PATHWAY AND DIAGNOSIS**	
GP practice code for the patient's registered GP practice at the treatment period start date (according to a patient trace)	
Pseudonymised GP practice code for the patient's registered GP practice at the treatment period start date (according to a patient trace)	
Broad cancer site grouping for the primary diagnosis recorded in the CWT data	
Specific cancer site grouping for the primary diagnosis recorded in the CWT data	
Indicator of a possible duplicate CWT treatment record	
For first treatment records, indicator for whether the cancer diagnosis resulted from an urgent GP referral for suspected cancer (sometimes known as 'two week wait referral') for the same broad type of suspected cancer as the recorded diagnosis	
For first treatment records, indicator for whether the cancer diagnosis resulted from an urgent GP referral for suspected cancer (sometimes known as 'two week wait referral') for the any type of suspected cancer	
**TREATMENT EVENTS** To carry Treatment Event details. rrence of this group is required if applicable to the scenario.	
The Start Date of the first, second or subsequent cancer treatment given to a PATIENT who is receiving care for a cancer condition, with a PRIMARY DIAGNOSIS (ICD) code within the range C00 to C97 or D05 as defined by NHS England.	
Month treatment started	
Year treatment started	
Option to provide number of days from a different event to the treatment start date (e.g. days from diagnosis to the treatment start date)	
The ORGANISATION SITE CODE of the Organisation where the TREATMENT START DATE FOR CANCER is recorded.	
The pseudonymised ORGANISATION SITE CODE of the Organisation where the TREATMENT START DATE FOR CANCER is recorded.	
The stage of treatment reached during a Cancer PATIENT PATHWAY for primary, recurrent or metastatic cancer.	
The type of treatment or care which was delivered in a Cancer Treatment Period.	
An indicator recording whether an individual episode of care is being delivered to a PATIENT as part of a CLINICAL TRIAL.	
The type of care setting where the cancer care relating to the TREATMENT START DATE FOR CANCER took place.	
The intent of the delivered beam radiation for PATIENTS with a cancer PRIMARY DIAGNOSIS (ICD), as defined by NHS England.	
The priority for the Radiotherapy Treatment Course as classified by the requesting clinician.	
The number of days that should be removed from the derived waiting time between CANCER TREATMENT PERIOD START DATE and TREATMENT START DATE FOR CANCER.	

For peer review only

Justification

er review only

Introduction

The **National Bowel Cancer Audit (NBOCA)** was established to describe and
established and the 2015 Annual Report includes data on over 30,000 patients
The audit is commissioned by the Healthcare Quality Improvement Partnership
Clinical Audit Support Unit within the Health and Social Care Information Centr
The 2015 annual report will be published in December 2015; this includes data

Inclusion:
The audit includes all new diagnoses of primary bowel cancer within the currer
• Aged 18 or older at the date of diagnosis
• Diagnosed in the audit period (April-March)
• Diagnosed with colorectal cancer, with one of the follow ICD10 codes C18-C2

Exclusion:
• Recurrent cancer – the audit only reports on primary cancer diagnoses
• Primary cancer without a colorectal site
• Sarcomas
• B cell lymphoma
• Neuroendocrine / Carcinoid tumours
• Cloacogenic carcinoma
• Melanoma

Coverage

The audit follows patients diagnosed between:
01/04/2006 - 31/03/2013 (the vital status of each patient can be followed up wi

Data item selection

Please be aware that selecting patient identifiable data items – **the data items**
covering your organisation. Sensitive patient demographic data, event dates ar
3 anonymity. Privacy by design and default principles have also been applied t
below:

Key:

**Items in red**	Identifiable fields that require additional permissions to acce
	Sensitive items that require additional justification in your pı
	Privacy by design and default' alternatives to the sensitive i

Item number	Name
**1**	Pseudonymised patient ID
**2**	**NHSNumber**
**3**	**DateOfBirth**
**4**	DateOfBirth_YYYY
**5**	DateOfBirth_MM
**6**	Days from a number of options
**7**	**Surname**
**8**	**Forename**
**9**	**Postcode (at diagnosis)**
**10**	Postcode (at diagnosis) - 1st part of postcode
**11**	Gender

**12**	OrganisationFirstSeen
**13**	Pseudonymised code for OrganisationFirstSeen
**14**	DateOfDiagnosis
**15**	DateOfDiagnosis_YYYY
**16**	DateOfDiagnosis_mm
**17**	Days from a different event to date of diagnosis
**18**	SourceOfReferral
**19**	ClinicalNurseSpecialistIndication
**20**	ICD10MajorSite
**21**	SynchronousCancer
**22**	PerformanceStatus
**23**	CPEXAnaerobicThreshold
**24**	CarePlanIntent
**25**	PlannedCancerTreatmentType
**26**	NoCancerTreatmentReason
**27**	TCategory
**28**	NCategory
**29**	MCategory
**30**	MonitoringIntent
**31**	ProviderOrganisation
**32**	ProviderOrganisation (pseudonymised)
**33**	ASAGrade
**34**	CancerTreatmentCurability
**35**	CPEXAnaerobicThreshold
**36**	BMI
**37**	DateOfSurgery
**38**	DateOfSurgery_YYYY
**39**	DateOfSurgery_MM
**40**	Days from a different event to date of surgery
**41**	SurgicalUrgencyModeOfOperation
**42**	**Consultant code**

For peer review only

43	Consultant code (pseudonymised)
44	PrimaryProcedure
45	SurgicalAccess
46	RoboticSurgery
47	ImmediatePostoperativeCare
48	StatusOfCircumferentialExcisionMargin
49	NumberOfNodesExamined
50	NumberOfNodesPositive
51	FinalPathologyTCategory
52	FinalPathologyNCategory
53	FinalPathologyRadiologyMCategory
54	SizeOfSpecimen
55	TumourType
56	DifferentiationByWorstArea
57	LocalInvasionPolypoidTumours
58	LocalInvasionSessileTumours
59	CancerVascularOrLymphaticInvasion
60	PreOpInitialProviderOrganisation
61	PreOpInitialProviderOrganisation (pseudonymised)
62	PreOpInitialCancerTreatmentModality
63	PostOpProviderOrganisation
64	PostOpProviderOrganisation (pseudonymised)
65	PostOpCancerTreatmentModality

************************* **End of sheet** *************************

For peer review only

For p

For peer review only - http://bmjopen.bmj.com/site/about/guidelines.xhtml

For p

For p

For p

For p

For peer review only

d compare the diagnosis, care and outcomes of patients diagnosed with bowel cancer in Engla
s. The Audit's overall aim is to measure the quality of care and survival of patients with bowel c
o (HQIP), has been developed by the Association of Coloproctology of Great Britain and Irelan
re on behalf of the Clinical Effectiveness Unit (CEU) of the Royal College of Surgeons of Engla
a on patients diagnosed from 1 April 2013 to 31 March 2014.In 2016 annual report will be publi

nt reporting period. Included patients must be;

20;

th linkage to Cancer Registration data)

s marked below in red – will require you to have patient consent, section 251 approval, or the
nd geographies will be treated as de-personalised, unless anonymisation techniques are appl
to some of these data to provide a less disclosive alternative. The key explaining how these a

ess (as outlined above)
rotocol to access
items highlighted in dark grey

Description	Request Data Item
**Patient**	
PATIENTID	
**Mandatory Field. Must be valid number - those without a valid number (e.g. non-English nationals) should have had a temporary NHS number created.**	
**Mandatory Field. dd/mm/yyyy format**	
Mandatory Field. yyyy format - year	
Mandatory Field. mm format - month	
Option to provide number of days from a selected date	
**That part of a person's name which is used to describe family, clan, tribal group, or marital association.**	
**The forename or given name of a person.**	
**A person's postcode at diagnosis.**	
A person's postcode at diagnosis - first part of postcode	
A person's gender at time of diagnosis	
**Tumour**	

The ORGANISATION SITE CODE of the Health Care Provider at the first contact with the PATIENT.	
The pseudonymised ORGANISATION SITE CODE of the Health Care Provider at the first contact with the PATIENT.	
Record the date when cancer was confirmed or diagnosis agreed. This will typically be the date of the pathology report which confirms the cancer.	
Date of Diagnosis - year	
Date of Diagnosis - month	
Option to provide number of days from a selected date	
Record the source of referral of a patient referred to secondary care for the investigation of suspected cancer.	
Record if and when the patient saw an appropriate site specific clinical nurse specialist.	
Record the diagnosis that represents the main cancer site for which the patient is receiving care	
Record any synchronous tumours. Multiple responses are possible. Synchronous tumours are identified as discrete tumours apparently not in continuity with other primary cancers originating in the same site or tissue	
The patient's performance status, to be the value presented at the MDT meeting prior to the beginning of treatment.	
Record the anaerobic threshold. Value to be between 0-99 with 1 decimal place.	
The intention of the treatment being planned.	
The treatment(s) that are planned for the patient	
The reason no treatment(s) are planned for the patient	
The 'T' part of the TNM classification used to describe the clinico-radiological stage of the tumour prior to treatment. 5th edition of TNM is used for colorectal cancer	
The 'N' part of the TNM classification used to describe the clinico-radiological stage of the tumour prior to treatment. 5th edition of TNM is used for colorectal cancer	
The 'M' part of the TNM classification used to describe the clinico-radiological stage of the tumour prior to treatment. 5th edition of TNM is used for colorectal cancer	
The purpose of monitoring a patient. Note: in the Cancer Outcomes and Services Dataset this may only be used for First Definitive Treatment.	
**Surgery**	
Surgery Provider Organisation Code	
Pseudonymised Surgery Provider Organisation Code	
Record the ASA Grade as determined at the time of surgery. Data item used for risk adjustment	
The surgeons' opinion of the completeness of the excision based on clinical findings at the time of operation which should not be revised in the light of subsequent histopathology reporting. Curative means no local tumour was visibly present after the surgical procedure. Palliative means that visible tumour was left behind after the surgical resection	
Record the anaerobic threshold. Value to be between 0-99 with 1 decimal place.	
Estimate of a patient's Body Mass Index (BMI) at diagnosis.	
Record the date when the primary procedure took place.	
Record the date when the primary procedure took place. - year	
Record the date when the primary procedure took place. - month	
Option to provide number of days from a selected date	
Record the mode of surgery performed	
**The General Medical Council (GMC) Code for the consultant who was actually in overall charge of the patient's surgical treatment during the time the patient was in hospital undergoing surgery.**	

For peer review

Pseudonymised GMC code for consultant	
Record the procedure that best describes resection of the primary colorectal cancer.	
Record the access used to perform the operation	
Record any use of the robot in the operation	
Site of discharge form theatre recovery	
**Pathology**	
Record if the edge of the tumour is 1 mm or less from the circumferential resection margin (i.e. margin involved) Circumferential margin refers to the completeness of the surgeon's resection margin in the opinion of the histopathologist. In parts of the colon where it is completely surrounded by peritoneum, recording of the circumferential resection margin (CRM) is not appropriate.	
The number of local and regional nodes examined.	
The number of local and regional nodes reported as being positive for the presence of Tumour metastases.	
T CATEGORY (PATHOLOGICAL) is the Union for International Cancer Control (UICC) code which classifies the size and extent of the primary Tumour based on the evidence from a pathological examination.	
N CATEGORY (PATHOLOGICAL) is the Union for International Cancer Control (UICC) code which classifies the absence or presence and extent of regional lymph node metastases based on the evidence from a pathological examination.	
For 97% of patients the M stage is a radiological NOT a pathological judgement.<br>M CATEGORY (PATHOLOGICAL) is the Union for International Cancer Control (UICC) code which classifies the absence or presence of distant metastases based on the evidence from a pathological examination.	
The size in millimetres of the diameter of a lesion, largest if more than one, if the histology of a SAMPLE proves to be invasive.	
Tumour Type from pathology report e.g., Adenocarcinoma	
The definitive grade of the Tumour based on the evidence from a pathological examination.	
For local excision specimens<br>Polypoid tumours - Haggitt level	
For local excision specimens<br>Sessile tumours - Kikuchi level	
An indication of the presence or absence of unequivocal tumour in lymphatic and/or vascular spaces.	
**Chemoradiotherapy**	
Chemoradiotherapy Provider Organisation Code	
Pseudonymised Pre-op Chemoradiotherapy Provider Organisation Code	
The type of treatment or care which was delivered in a Cancer Treatment Period. This is the first oncology therapy given, or where applicable that give BEFORE Surgical resection of the primary colorectal cancer.	
Chemoradiotherapy Provider Organisation Code	
Pseudonymised Post-op Chemoradiotherapy Provider Organisation Code	
The type of treatment or care which was delivered in a Cancer Treatment Period. This is oncology therapy given AFTER Surgical resection of the primary colorectal cancer.	

peer review only

and and Wales; it is now well
cancer in England and Wales.
nd (ACPGBI) and is managed by the
land.
blished in summer 2016.

e appropriate statutory regulation
lied to render the data anonymised to k-
lternative can be selected is included

Justification

For peer review only - http://bmjopen.bmj.com/site/about/guidelines.xhtml

peer review only

For peer review only

For peer review only

For peer review only

peer review only

For peer review only

For peer review only

For peer review only

For peer review only

For peer review only

For peer review only

For peer review only

For peer review only

Introduction

The Quality of Life of Cancer Survivors in England: Pilot Survey (2011) was cor
The survey was conducted by Quality Health in conjunction with cancer registri
prostate cancer and non-Hodgkin's lymphoma (NHL)) and at four different time
Responses were obtained (n=3300) of individuals approach to participant, givin
Assessment of Cancer Therapy) items and SDI (Social Difficulties Inventory).
https://www.gov.uk/government/uploads/system/uploads/attachment_data/file/2

The survey was repeated in 2012 for the same cohort of patients, with an 85%

Data Coverage

Routinely linked to incident cancer cases from July 2006 - July 2010

Data items selection:

Please be aware that selecting patient identifiable data items – the data items r
Sensitive patient demographic data, event dates and geographies will be treate
principles have also been applied to some of these data to provide a less disclo

Key:

**Items in red**	Identifiable fields that require additional permissions to access (
	Sensitive items that require additional justification in your protoc
	Privacy by design and default' alternatives to the sensitive items

Breast cancer PROMS

Question number	Question text
The questions in this	
**N/A**	Pseudonymised patient ID
**01a**	What treatments have you received for your breast cancer?
**01b**	What treatments have you received for your breast cancer?
**01c**	What treatments have you received for your breast cancer?
**01d**	What treatments have you received for your breast cancer?
**2**	How long is it since you completed your initial treatment for breast cancer?
**3**	How has your breast cancer responded to treatment?
**04a**	If you have had breast surgery, do any of the following apply to you?
**04b**	If you have had breast surgery, do any of the following apply to you?
**04c**	If you have had breast surgery, do any of the following apply to you?
**04d**	If you have had breast surgery, do any of the following apply to you?
**04e**	If you have had breast surgery, do any of the following apply to you?
**5**	Mobility (NCSI)
**6**	Self-Care (NCSI)

**7**	Usual Activities (NCSI)
**8**	Pain / Discomfort (NCIS)
**9**	Anxiety / Depression
	The ques
**10**	I have been short of breath.
**11**	I am self conscious about the way I dress.
**12**	One or both of my arms are swollen or tender.
**13**	I feel sexually attractive.
**14**	I am bothered by hair loss.
**15**	I worry that other members of my family might someday get the same illness I have.
**16**	I worry about the effect of stress on my illness.
**17**	I am bothered by a change in weight.
**18**	I have certain parts of my body where I experience pain.
**19**	In the past week, on how many days have you done a total of 30 minutes or more of physical activity, which was enough to raise your heart rate?
	The questic
**20**	Have you had any difficulty in maintaining your independence?
**21**	Have you had any difficulty in carrying out your domestic chores?
**22**	Have you had any difficulty with managing your own personal care?
**23**	Have you had any difficulty with looking after those who depend on you?
**24**	Have any of those close to you (e.g. partner, children, parents) had any difficulty with the support available to them?
**25**	Have you had any difficulty with benefits?
**26**	Have you had any financial difficulties?
**27**	Have you had any difficulty with financial services?
**28**	Have you had any difficulty concerning your work?
**29**	Have you had any difficulty with planning for your own or your family's future?
**30**	Have you had any difficulty with communicating with those closest to you?
**31**	Have you had any difficulty with communicating with others?
**32**	Have you had any difficulty concerning sexual matters?
**33**	Have you had any difficulty concerning plans to have a family?
**34**	Have you had any difficulty concerning your appearance or body image?
**35**	Have you felt isolated?
**36**	Have you had any difficulty with getting around?
**37**	Have you had any difficulty with where you live?
**38**	Have you had any difficulty in carrying out your recreational activities?
**39**	Have you had any difficulty with your plans to travel or take a holiday?
**40**	Have you had any difficulty with any other area of your everyday life?
**41**	I have fears about my cancer spreading.

For peer review only

**42**	I have fears about my cancer coming back.
**43**	I have fears about death and dying.
**44**	I experience memory loss.
**45**	I have trouble sleeping.
**46**	I have trouble concentrating.
**47**	I always feel tired.
**48**	I experience mood swings.
**49**	I am often irritable.
**50**	Do you have an up-to-date written care plan?
**51**	Do you have a named nurse who you can contact if you have a worry about your cancer care?
**52**	Do you know who to contact if you have a concern about any aspect of living with or after cancer?
**53**	Do you think that hospital staff did everything they could to support you following your cancer treatment?
**54**	Do you think that GPs and nurses at your general practice do everything they can to support you following your cancer treatment?
**55**	Following your initial cancer treatment have you been given enough care and help from health and social services (for example, district nurses, home helps or occupational therapists)?
**56a**	Would it have been helpful to have had more advice or information on any of the following issues:
**56b**	Would it have been helpful to have had more advice or information on any of the following issues:
**56c**	Would it have been helpful to have had more advice or information on any of the following issues:
**56d**	Would it have been helpful to have had more advice or information on any of the following issues:
**56e**	Would it have been helpful to have had more advice or information on any of the following issues:
**56f**	Would it have been helpful to have had more advice or information on any of the following issues:
**56g**	Would it have been helpful to have had more advice or information on any of the following issues:
**56h**	Would it have been helpful to have had more advice or information on any of the following issues:
**56i**	Would it have been helpful to have had more advice or information on any of the following issues:
	If you are helping someon
**57**	What year were you born?
**58**	Are you male or female?
**59**	**Which of the following best describes your sexual orientation?**
**60**	Which statement best describes your living arrangements?
**61**	Do you have a long standing health condition? (NCSI)
**62a**	Which, if any, of the following conditions do you have?
**62b**	Which, if any, of the following conditions do you have?
**62c**	Which, if any, of the following conditions do you have?
**62d**	Which, if any, of the following conditions do you have?
**62e**	Which, if any, of the following conditions do you have?
**62f**	Which, if any, of the following conditions do you have?
**62g**	Which, if any, of the following conditions do you have?
**62h**	Which, if any, of the following conditions do you have?
**62i**	Which, if any, of the following conditions do you have?
**62j**	Which, if any, of the following conditions do you have?
**62k**	Which, if any, of the following conditions do you have?

For peer review only

62l	Which, if any, of the following conditions do you have?
62m	Which, if any, of the following conditions do you have?
62n	Which, if any, of the following conditions do you have?
62o	Which, if any, of the following conditions do you have?
62p	Which, if any, of the following conditions do you have?
62q	Which, if any, of the following conditions do you have?
62r	Which, if any, of the following conditions do you have?
63	What was your employment status before you were diagnosed with cancer?
64	What is your employment status currently?
65	If you are currently employed at the moment, are you:
66a	To which of these ethnic groups would you say you belong?
66b	Broad ethnic group
**66_Other**	**To which of these ethnic groups would you say you belong?**

Colorectal cancer PROMS

**Question number**	**Question text**
	The questions in this
N/A	Pseudonymised patient ID
01a	What treatments have you received for your colorectal / GI cancer?
01b	What treatments have you received for your colorectal / GI cancer?
01c	What treatments have you received for your colorectal / GI cancer?
2	How long is it since you completed your initial treatment for colorectal / GI cancer?
3	How has your colorectal / GI cancer responded to treatment?
4	If you have a stoma (e.g. colostomy) is it:
5	Mobility (NCSI)
6	Self-Care (NCSI)
7	Usual Activities (NCSI)
8	Pain / Discomfort (NCIS)
9	Anxiety / Depression
	The ques
10	I have swelling or cramps in my stomach area.

**11**	I am losing weight.
**12**	I have control of my bowels.
**13**	I can digest my food well.
**14**	I have diarrhoea.
**15**	I have a good appetite.
**16**	I like the appearance of my body.
**17**	I have difficulty urinating.
**18**	I urinate more frequently than usual.
**19**	I leak urine.
**20**	Do you have an ostomy appliance / stoma?
**21**	I am embarrassed by my ostomy appliance / stoma.
**22**	Caring for my ostomy appliance / stoma is difficult.
**23**	Do you have any difficulty in controlling your bowels (e.g. any accidents)?
**24**	If yes, how often do you have difficulties?
**25**	In the past week, on how many days have you done a total of 30 minutes or more of physical activity, which was enough to raise your heart rate?
	The questio
**26**	Have you had any difficulty in maintaining your independence?
**27**	Have you had any difficulty in carrying out your domestic chores?
**28**	Have you had any difficulty with managing your own personal care?
**29**	Have you had any difficulty with looking after those who depend on you?
**30**	Have any of those close to you (e.g. partner, children, parents) had any difficulty with the support available to them?
**31**	Have you had any difficulty with benefits?
**32**	Have you had any financial difficulties?
**33**	Have you had any difficulty with financial services?
**34**	Have you had any difficulty concerning your work?
**35**	Have you had any difficulty with planning for your own or your family's future?
**36**	Have you had any difficulty with communicating with those closest to you?
**37**	Have you had any difficulty with communicating with others?
**38**	Have you had any difficulty concerning sexual matters?
**39**	Have you had any difficulty concerning plans to have a family?
**40**	Have you had any difficulty concerning your appearance or body image?
**41**	Have you felt isolated?
**42**	Have you had any difficulty with getting around?
**43**	Have you had any difficulty with where you live?
**44**	Have you had any difficulty in carrying out your recreational activities?
**45**	Have you had any difficulty with your plans to travel or take a holiday?
**46**	Have you had any difficulty with any other area of your everyday life?
**47**	I have fears about my cancer spreading.
**48**	I have fears about my cancer coming back.
**49**	I have fears about death and dying.
**50**	I experience memory loss.
**51**	I have trouble sleeping.

For peer review only

**52**	I have trouble concentrating.
**53**	I always feel tired.
**54**	I experience mood swings.
**55**	I am often irritable.
**56**	Do you have an up-to-date written care plan?
**57**	Do you have a named nurse who you can contact if you have a worry about your cancer care?
**58**	Do you know who to contact if you have a concern about any aspect of living with or after cancer?
**59**	Do you think that hospital staff did everything they could to support you following your cancer treatment?
**60**	Do you think that GPs and nurses at your general practice do everything they can to support you following your cancer treatment?
**61**	Following your initial cancer treatment have you been given enough care and help from health and social services (for example, district nurses, home helps or occupational therapists)?
**62a**	Would it have been helpful to have had more advice or information on any of the following issues:
**62b**	Would it have been helpful to have had more advice or information on any of the following issues:
**62c**	Would it have been helpful to have had more advice or information on any of the following issues:
**62d**	Would it have been helpful to have had more advice or information on any of the following issues:
**62e**	Would it have been helpful to have had more advice or information on any of the following issues:
**62f**	Would it have been helpful to have had more advice or information on any of the following issues:
**62g**	Would it have been helpful to have had more advice or information on any of the following issues:
**62h**	Would it have been helpful to have had more advice or information on any of the following issues:
**62i**	Would it have been helpful to have had more advice or information on any of the following issues:
	If you are helping someon
**63**	What year were you born?
**64**	Are you male or female?
**65**	**Which of the following best describes your sexual orientation?**
**66**	Which statement best describes your living arrangements?
**67**	Do you have a long standing health condition? (NCSI)
**68a**	Which, if any, of the following conditions do you have?
**68b**	Which, if any, of the following conditions do you have?
**68c**	Which, if any, of the following conditions do you have?
**68d**	Which, if any, of the following conditions do you have?
**68e**	Which, if any, of the following conditions do you have?
**68f**	Which, if any, of the following conditions do you have?
**68g**	Which, if any, of the following conditions do you have?
**68h**	Which, if any, of the following conditions do you have?
**68i**	Which, if any, of the following conditions do you have?
**68j**	Which, if any, of the following conditions do you have?
**68k**	Which, if any, of the following conditions do you have?
**68l**	Which, if any, of the following conditions do you have? Learning difficulty
**68m**	Which, if any, of the following conditions do you have?
**68n**	Which, if any, of the following conditions do you have?
**68o**	Which, if any, of the following conditions do you have?
**68p**	Which, if any, of the following conditions do you have?

For peer review only

**68q**	Which, if any, of the following conditions do you have?
**68r**	Which, if any, of the following conditions do you have?
**69**	What was your employment status before you were diagnosed with cancer?
**70**	What is your employment status currently?
**71**	If you are currently employed at the moment, are you:
**66a**	To which of these ethnic groups would you say you belong?
**66b**	Broad ethnic group
**72_Other**	**To which of these ethnic groups would you say you belong?**

Non-Hodgkin's Lymphoma PROMS

Question number	Question text
	The questions in this
**N/A**	Pseudonymised patient ID
**01a**	What treatments have you received for your Non Hodgkin's Lymphoma (NHL)?
**01b**	What treatments have you received for your Non Hodgkin's Lymphoma (NHL)?
**01c**	What treatments have you received for your Non Hodgkin's Lymphoma (NHL)? Surgery
**01d**	What treatments have you received for your Non Hodgkin's Lymphoma (NHL)?
**01e**	What treatments have you received for your Non Hodgkin's Lymphoma (NHL)? Stem cell transplant
**2**	How long is it since you completed your initial treatment for NHL?
**3**	How has your NHL responded to treatment?
**4**	Mobility (NCSI)
**5**	Self-Care (NCSI)
**6**	Usual Activities (NCSI)
**7**	Pain / Discomfort (NCIS)
**8**	Anxiety / Depression
	The ques
**9**	I have certain parts of my body where I experience pain.
**10**	I am bothered by lumps or swelling in certain parts of my body.

**11**	I am bothered by fevers.
**12**	I have night sweats.
**13**	I am bothered by itching.
**14**	I have trouble sleeping at night.
**15**	I get tired easily.
**16**	I am losing weight.
**17**	I have a loss of appetite.
**18**	I have trouble concentrating [7 days]
**19**	I worry about getting infections.
**20**	I worry that I might get new symptoms of my illness.
**21**	I feel isolated from others because of my illness or treatment.
**22**	I have emotional ups and downs.
**23**	Because of my illness, I have difficulty planning for the future.
**24**	In the past week, on how many days have you done a total of 30 minutes or more of physical activity, which was enough to raise your heart rate?
	The questio
**25**	Have you had any difficulty in maintaining your independence?
**26**	Have you had any difficulty in carrying out your domestic chores?
**27**	Have you had any difficulty with managing your own personal care?
**28**	Have you had any difficulty with looking after those who depend on you?
**29**	Have any of those close to you (e.g. partner, children, parents) had any difficulty with the support available to them?
**30**	Have you had any difficulty with benefits?
**31**	Have you had any financial difficulties?
**32**	Have you had any difficulty with financial services?
**33**	Have you had any difficulty concerning your work?
**34**	Have you had any difficulty with planning for your own or your family's future?
**35**	Have you had any difficulty with communicating with those closest to you?
**36**	Have you had any difficulty with communicating with others?
**37**	Have you had any difficulty concerning sexual matters?
**38**	Have you had any difficulty concerning plans to have a family?
**39**	Have you had any difficulty concerning your appearance or body image?
**40**	Have you felt isolated?
**41**	Have you had any difficulty with getting around?
**42**	Have you had any difficulty with where you live?
**43**	Have you had any difficulty in carrying out your recreational activities?
**44**	Have you had any difficulty with your plans to travel or take a holiday?
**45**	Have you had any difficulty with any other area of your everyday life?
**46**	I have fears about my cancer spreading.
**47**	I have fears about my cancer coming back.
**48**	I have fears about death and dying.
**49**	I experience memory loss.
**50**	I have trouble sleeping.

For peer review only

**51**	I have trouble concentrating.
**52**	I always feel tired.
**53**	I experience mood swings.
**54**	I am often irritable.
**55**	Do you have an up-to-date written care plan?
**56**	Do you have a named nurse who you can contact if you have a worry about your cancer care?
**57**	Do you know who to contact if you have a concern about any aspect of living with or after cancer?
**58**	Do you think that hospital staff did everything they could to support you following your cancer treatment?
**59**	Do you think that GPs and nurses at your general practice do everything they can to support you following your cancer treatment?
**60**	Following your initial cancer treatment have you been given enough care and help from health and social services (for example, district nurses, home helps or occupational therapists)?
**61a**	Would it have been helpful to have had more advice or information on any of the following issues:
**61b**	Would it have been helpful to have had more advice or information on any of the following issues:
**61c**	Would it have been helpful to have had more advice or information on any of the following issues: Financial help or benefits
**61d**	Would it have been helpful to have had more advice or information on any of the following issues: Free prescriptions
**61e**	Would it have been helpful to have had more advice or information on any of the following issues:
**61f**	Would it have been helpful to have had more advice or information on any of the following issues:
**61g**	Would it have been helpful to have had more advice or information on any of the following issues:
**61h**	Would it have been helpful to have had more advice or information on any of the following issues:
**61i**	Would it have been helpful to have had more advice or information on any of the following issues:
	If you are helping someone
**62**	What year were you born?
**63**	Are you male or female?
**64**	**Which of the following best describes your sexual orientation?**
**65**	Which statement best describes your living arrangements?
**66**	Do you have a long standing health condition? (NCSI)
**67a**	Which, if any, of the following conditions do you have?
**67b**	Which, if any, of the following conditions do you have?
**67c**	Which, if any, of the following conditions do you have?
**67d**	Which, if any, of the following conditions do you have?
**67e**	Which, if any, of the following conditions do you have?
**67f**	Which, if any, of the following conditions do you have?
**67g**	Which, if any, of the following conditions do you have?
**67h**	Which, if any, of the following conditions do you have?
**67i**	Which, if any, of the following conditions do you have?
**67j**	Which, if any, of the following conditions do you have?
**67k**	Which, if any, of the following conditions do you have?
**67l**	Which, if any, of the following conditions do you have?
**67m**	Which, if any, of the following conditions do you have? Liver disease

For peer review only

67n	Which, if any, of the following conditions do you have?
67o	Which, if any, of the following conditions do you have?
67p	Which, if any, of the following conditions do you have?
67q	Which, if any, of the following conditions do you have?
67r	Which, if any, of the following conditions do you have?
68	What was your employment status before you were diagnosed with cancer?
69	What is your employment status currently?
70	If you are currently employed at the moment, are you:
66a	To which of these ethnic groups would you say you belong?
66b	Broad ethnic group
**71_Other**	**To which of these ethnic groups would you say you belong?**

Prostate cancer PROMS

**Question number**	**Question text**
	The questions in this
**N/A**	Pseudonymised patient ID
**01a**	What treatments have you received for your prostate cancer?
**01b**	What treatments have you received for your prostate cancer?
**01c**	What treatments have you received for your prostate cancer?
**01d**	What treatments have you received for your prostate cancer?
**01e**	What treatments have you received for your prostate cancer?
**01f**	What treatments have you received for your prostate cancer?
**01g**	What treatments have you received for your prostate cancer?
**2**	How long is it since you completed your initial treatment for prostate cancer?
**3**	How has your prostate cancer responded to treatment?
**4**	Mobility (NCSI)
**5**	Self-Care (NCSI)
**6**	Usual Activities (NCSI)
**7**	Pain / Discomfort (NCIS)

**8**	Anxiety / Depression
The ques	
**9**	I am losing weight.
**10**	I have a good appetite.
**11**	I have aches and pains that bother me
**12**	I have certain parts of my body where I experience pain.
**13**	My pain keeps me from doing things I want to do
**14**	I am satisfied with my present comfort level
**15**	I have trouble moving my bowels
**16**	I have difficulty urinating.
**17**	I urinate more frequently than usual.
**18**	My problems with urinating limit my activities
**19**	I am able to have and maintain an erection
**20**	I leak urine.
**21**	Do you have any difficulty in controlling your bowels (e.g. any accidents)?
**22**	If yes, how often do you have difficulties?
**23**	In the past week, on how many days have you done a total of 30 minutes or more of physical activity, which was enough to raise your heart rate?
The questio	
**24**	Have you had any difficulty in maintaining your independence?
**25**	Have you had any difficulty in carrying out your domestic chores?
**26**	Have you had any difficulty with managing your own personal care?
**27**	Have you had any difficulty with looking after those who depend on you?
**28**	Have any of those close to you (e.g. partner, children, parents) had any difficulty with the support available to them?
**29**	Have you had any difficulty with benefits?
**30**	Have you had any financial difficulties?
**31**	Have you had any difficulty with financial services?
**32**	Have you had any difficulty concerning your work?
**33**	Have you had any difficulty with planning for your own or your family's future?
**34**	Have you had any difficulty with communicating with those closest to you?
**35**	Have you had any difficulty with communicating with others?
**36**	Have you had any difficulty concerning sexual matters?
**37**	Have you had any difficulty concerning plans to have a family?
**38**	Have you had any difficulty concerning your appearance or body image?
**39**	Have you felt isolated?
**40**	Have you had any difficulty with getting around?
**41**	Have you had any difficulty with where you live?
**42**	Have you had any difficulty in carrying out your recreational activities?
**43**	Have you had any difficulty with your plans to travel or take a holiday?
**44**	Have you had any difficulty with any other area of your everyday life?
**45**	I have fears about my cancer spreading.

For peer review only

**46**	I have fears about my cancer coming back.
**47**	I have fears about death and dying.
**48**	I experience memory loss.
**49**	I have trouble sleeping.
**50**	I have trouble concentrating.
**51**	I always feel tired.
**52**	I experience mood swings.
**53**	I am often irritable.
**54**	Do you have an up-to-date written care plan?
**55**	Do you have a named nurse who you can contact if you have a worry about your cancer care?
**56**	Do you know who to contact if you have a concern about any aspect of living with or after cancer?
**57**	Do you think that hospital staff did everything they could to support you following your cancer treatment?
**58**	Do you think that GPs and nurses at your general practice do everything they can to support you following your cancer treatment?
**59**	Following your initial cancer treatment have you been given enough care and help from health and social services (for example, district nurses, home helps or occupational therapists)?
**60a**	Would it have been helpful to have had more advice or information on any of the following issues:
**60b**	Would it have been helpful to have had more advice or information on any of the following issues:
**60c**	Would it have been helpful to have had more advice or information on any of the following issues:
**60d**	Would it have been helpful to have had more advice or information on any of the following issues:
**60e**	Would it have been helpful to have had more advice or information on any of the following issues:
**60f**	Would it have been helpful to have had more advice or information on any of the following issues:
**60g**	Would it have been helpful to have had more advice or information on any of the following issues:
**60h**	Would it have been helpful to have had more advice or information on any of the following issues:
**60i**	Would it have been helpful to have had more advice or information on any of the following issues:
	If you are helping someon
**61**	What year were you born?
**62**	Are you male or female?
**63**	**Which of the following best describes your sexual orientation?**
**64**	Which statement best describes your living arrangements?
**65**	Do you have a long standing health condition? (NCSI)
**66a**	Which, if any, of the following conditions do you have?
**66b**	Which, if any, of the following conditions do you have?
**66c**	Which, if any, of the following conditions do you have?
**66d**	Which, if any, of the following conditions do you have?
**66e**	Which, if any, of the following conditions do you have?
**66f**	Which, if any, of the following conditions do you have?
**66g**	Which, if any, of the following conditions do you have?
**66h**	Which, if any, of the following conditions do you have?
**66i**	Which, if any, of the following conditions do you have?
**66j**	Which, if any, of the following conditions do you have? High blood pressure
**66k**	Which, if any, of the following conditions do you have?

**66l**	Which, if any, of the following conditions do you have?
**66m**	Which, if any, of the following conditions do you have? Liver disease
**66n**	Which, if any, of the following conditions do you have? Long term back problems
**66o**	Which, if any, of the following conditions do you have? Long-standing mental health problem
**66p**	Which, if any, of the following conditions do you have?
**66q**	Which, if any, of the following conditions do you have?
**66r**	Which, if any, of the following conditions do you have?
**67**	What was your employment status before you were diagnosed with cancer?
**68**	What is your employment status currently?
**69**	If you are currently employed at the moment, are you:
**66a**	To which of these ethnic groups would you say you belong?
**66b**	Broad ethnic group
**70_Other**	**To which of these ethnic groups would you say you belong?**
	************************** **End of sheet** **************************

mmissioned by the Department of Health as part of the National Cancer Survivorship Initiative (NCSI).
ies. The survey measures overall quality of life of representative samples of cancer survivors with four different
points after diagnosis (approximately one, two, three or five years).
ng a response rate of 66%. Outcome questions in the survey are made up of three instruments: the EQ-5D (Eur

267042/9284-TSO-2900701-PROMS-1.pdf

response rate

marked below in red – will require you to have patient consent, section 251 approval, or the appropriate statutor
ed as de-personalised, unless anonymisation techniques are applied to render the data anonymised to k-3 anor
osive alternative. The key explaining how these alternative can be selected is included below:

(as outlined above)
col to access
s highlighted in dark grey

**Possible answers**	**Request Data Item**
**Your health today**<br>s section are all about your health and how you feel on the day you complete this questionnaire.	
PATIENTID	
Radiotherapy	
Chemotherapy (excluding hormone treatment)	
Hormone treatment	
Surgery	
I am still having my initial treatment, It is less than 3 months since my initial treatment, It is between 3 and 12 months since my initial treatment, It is between 1 and 5 years since my initial treatment, It is more than 5 years since my initial treatment, Don't know / can't remember	
My breast cancer has responded fully to treatment (I am in remission), My breast cancer has been treated but is still present, My breast cancer has not been treated at all, My breast cancer has come back after it was originally treated, I am not certain what is happening with my breast cancer	
I have had a lumpectomy	
I have had a mastectomy	
I have had breast reconstruction	
I am awaiting or considering breast reconstruction	
None of these apply to me	
I have no problems in walking about, I have slight problems in walking about, I have moderate problems in walking about, I have severe problems in walking about, I am unable to walk about	
I have no problems washing or dressing myself, I have slight problems washing or dressing myself, I have moderate problems washing or dressing myself, I have severe problems washing or dressing myself, I am unable to wash or dress myself	

I have no problems doing my usual activities, I have slight problems doing my usual activities, I have moderate problems doing my usual activities, I have severe problems doing my usual activities, I am unable to do my usual activities	
I have no pain or discomfort, I have slight pain or discomfort, I have moderate pain or discomfort, I have severe pain or discomfort, I have extreme pain or discomfort	
I am not anxious or depressed, I am slightly anxious or depressed, I am moderately anxious or depressed, I am severely anxious or depressed, I am extremely anxious or depressed	
**Your health and wellbeing in the last 7 days**<br>stions in this section are about your health and how you have felt in the past 7 days.	
Not at all, A little bit, Somewhat, Quite a bit, Very much	
Not at all, A little bit, Somewhat, Quite a bit, Very much	
Not at all, A little bit, Somewhat, Quite a bit, Very much	
Not at all, A little bit, Somewhat, Quite a bit, Very much	
Not at all, A little bit, Somewhat, Quite a bit, Very much	
Not at all, A little bit, Somewhat, Quite a bit, Very much	
Not at all, A little bit, Somewhat, Quite a bit, Very much	
Not at all, A little bit, Somewhat, Quite a bit, Very much	
Not at all, A little bit, Somewhat, Quite a bit, Very much	
None, 1 day, 2 days, 3 days, 4 days, 5 days, 6 days, 7 days	
**Your health and wellbeing in the last month**<br>ons in this section are about your health and how you have felt during the past month.	
No difficulty, A little, Quite a bit, Very much, Does not apply	
No difficulty, A little, Quite a bit, Very much, Does not apply	
No difficulty, A little, Quite a bit, Very much, Does not apply	
No difficulty, A little, Quite a bit, Very much, Does not apply	
No difficulty, A little, Quite a bit, Very much, Does not apply	
No difficulty, A little, Quite a bit, Very much, Does not apply	
No difficulty, A little, Quite a bit, Very much, Does not apply	
No difficulty, A little, Quite a bit, Very much, Does not apply	
No difficulty, A little, Quite a bit, Very much, Does not apply	
No difficulty, A little, Quite a bit, Very much, Does not apply	
No difficulty, A little, Quite a bit, Very much, Does not apply	
No difficulty, A little, Quite a bit, Very much, Does not apply	
No difficulty, A little, Quite a bit, Very much, Does not apply	
No difficulty, A little, Quite a bit, Very much, Does not apply	
No difficulty, A little, Quite a bit, Very much, Does not apply	
No difficulty, A little, Quite a bit, Very much, Does not apply	
No difficulty, A little, Quite a bit, Very much, Does not apply	
No difficulty, A little, Quite a bit, Very much, Does not apply	
No difficulty, A little, Quite a bit, Very much, Does not apply	
No difficulty, A little, Quite a bit, Very much, Does not apply	
No difficulty, A little, Quite a bit, Very much, Does not apply	
Strongly Agree, Agree, Neither agree nor disagree, Disagree, Strongly disagree, Does not apply to me	

For peer review only

Strongly Agree, Agree, Neither agree nor disagree, Disagree, Strongly disagree, Does not apply to me	
Strongly Agree, Agree, Neither agree nor disagree, Disagree, Strongly disagree	
Strongly Agree, Agree, Neither agree nor disagree, Disagree, Strongly disagree	
Strongly Agree, Agree, Neither agree nor disagree, Disagree, Strongly disagree	
Strongly Agree, Agree, Neither agree nor disagree, Disagree, Strongly disagree	
Strongly Agree, Agree, Neither agree nor disagree, Disagree, Strongly disagree	
Strongly Agree, Agree, Neither agree nor disagree, Disagree, Strongly disagree	
Strongly Agree, Agree, Neither agree nor disagree, Disagree, Strongly disagree	
**Overall support and care**	
Yes definitely, Yes I think so, No, I do not need a care plan, Don't know	
Yes, No, Don't know	
Yes definitely, Yes I think so, No	
Yes all of the time, Only some of the time, Never, I did not need any support	
Yes all of the time, Only some of the time, Never, My general practice is not involved, I do not need any support	
Yes definitely, Yes to some extent, No, I did not need help from health or social services, Don't know / can't remember	
Diet and lifestyle	
Physical activity and exercise	
Financial help or benefits	
Free prescriptions	
Returning to or staying in work	
Information / advice for family / friends / carer	
The physical aspects of living with and after cancer	
The psychological or emotional aspects of living with and after cancer	
I have all the information and advice I need	
**About you**<br>e to complete this questionnaire, please make sure this information is the patient's and not your own.	
In format YYYY	
Male, Female	
**Heterosexual / straight (opposite sex), Bisexual (both sexes), Gay or Lesbian (same sex), Other, Prefer not to answer**	
I live with partner / spouse / family / friends, I live alone, I live in a nursing home hospital or other long term care home, Other	
Yes, No, Don't know / can't say	
Alzheimer's disease or dementia	
Angina	
Arthritis	
Asthma or other chronic chest problem	
Blindness or visual impairment	
Deafness or hearing impairment	
Diabetes	
Epilepsy	
Heart condition	
High blood pressure	
Kidney disease	

Learning difficulty	
Liver disease	
Long term back problems	
Long-standing mental health problem	
Long-standing neurological problem	
Another long-standing condition	
I do not have any of these conditions	
Full time employment, Part time employment, Homemaker, Student (in education), Retired, Unemployed – and seeking work, Unemployed – unable to work for health reasons, Other	
Full time employment, Part time employment, Homemaker, Student (in education), Retired, Unemployed – and seeking work, Unemployed – unable to work for health reasons, Other	
Not working at all, Working less hours than usual, Working your usual hours, Working more hours than usual, This question does not apply to me	
British, Irish, Any other White background, White and Black Caribbean, White and Black African, White and Asian, Any other mixed background, Indian, Pakistani, Bangladeshi, Any other Asian background, Caribbean, African, Any other Black background, Chinese, Any other ethnic group	
Option to group ethnicities (e.g. white/ non-white/ unknown)	
**Free text**	

**Possible answers**	**Request Data Item**
**Your health today**<br>s section are all about your health and how you feel on the day you complete this questionnaire.	
PATIENTID	
Radiotherapy	
Chemotherapy	
Surgery	
I am still having my initial treatment, It is less than 3 months since my initial treatment, It is between 3 and 12 months since my initial treatment, It is between 1 and 5 years since my initial treatment, It is more than 5 years since my initial treatment, Don't know / can't remember	
My colorectal / GI cancer has responded fully to treatment (I am in remission), My colorectal / GI cancer has been treated but is still present, My colorectal / GI cancer has not been treated at all, My colorectal / GI cancer has come back after it was originally treated, I am not certain what is happening with my colorectal / GI cancer	
Still present, Reversed, This does not apply to me	
I have no problems in walking about, I have slight problems in walking about, I have moderate problems in walking about, I have severe problems in walking about, I am unable to walk about	
I have no problems washing or dressing myself, I have slight problems washing or dressing myself, I have moderate problems washing or dressing myself, I have severe problems washing or dressing myself, I am unable to wash or dress myself	
I have no problems doing my usual activities, I have slight problems doing my usual activities, I have moderate problems doing my usual activities, I have severe problems doing my usual activities, I am unable to do my usual activities	
I have no pain or discomfort, I have slight pain or discomfort, I have moderate pain or discomfort, I have severe pain or discomfort, I have extreme pain or discomfort	
I am not anxious or depressed, I am slightly anxious or depressed, I am moderately anxious or depressed, I am severely anxious or depressed, I am extremely anxious or depressed	
**Your health and wellbeing in the last 7 days**<br>stions in this section are about your health and how you have felt in the past 7 days.	
Not at all, A little bit, Somewhat, Quite a bit, Very much	

Not at all, A little bit, Somewhat, Quite a bit, Very much	
Not at all, A little bit, Somewhat, Quite a bit, Very much	
Not at all, A little bit, Somewhat, Quite a bit, Very much	
Not at all, A little bit, Somewhat, Quite a bit, Very much	
Not at all, A little bit, Somewhat, Quite a bit, Very much	
Not at all, A little bit, Somewhat, Quite a bit, Very much	
Not at all, A little bit, Somewhat, Quite a bit, Very much	
Not at all, A little bit, Somewhat, Quite a bit, Very much	
Not at all, A little bit, Somewhat, Quite a bit, Very much	
No, Yes	
Not at all, A little bit, Somewhat, Quite a bit, Very much	
Not at all, A little bit, Somewhat, Quite a bit, Very much	
No, Yes	
Monthly, Weekly, Daily, Constantly, It varies	
None, 1 day, 2 days, 3 days, 4 days, 5 days, 6 days, 7 days	
**Your health and wellbeing in the last month** ons in this section are about your health and how you have felt during the past month.	
No difficulty, A little, Quite a bit, Very much, Does not apply	
No difficulty, A little, Quite a bit, Very much, Does not apply	
No difficulty, A little, Quite a bit, Very much, Does not apply	
No difficulty, A little, Quite a bit, Very much, Does not apply	
No difficulty, A little, Quite a bit, Very much, Does not apply	
No difficulty, A little, Quite a bit, Very much, Does not apply	
No difficulty, A little, Quite a bit, Very much, Does not apply	
No difficulty, A little, Quite a bit, Very much, Does not apply	
No difficulty, A little, Quite a bit, Very much, Does not apply	
No difficulty, A little, Quite a bit, Very much, Does not apply	
No difficulty, A little, Quite a bit, Very much, Does not apply	
No difficulty, A little, Quite a bit, Very much, Does not apply	
No difficulty, A little, Quite a bit, Very much, Does not apply	
No difficulty, A little, Quite a bit, Very much, Does not apply	
No difficulty, A little, Quite a bit, Very much, Does not apply	
No difficulty, A little, Quite a bit, Very much, Does not apply	
No difficulty, A little, Quite a bit, Very much, Does not apply	
No difficulty, A little, Quite a bit, Very much, Does not apply	
No difficulty, A little, Quite a bit, Very much, Does not apply	
No difficulty, A little, Quite a bit, Very much, Does not apply	
No difficulty, A little, Quite a bit, Very much, Does not apply	
Strongly Agree, Agree, Neither agree nor disagree, Disagree, Strongly disagree, Does not apply to me	
Strongly Agree, Agree, Neither agree nor disagree, Disagree, Strongly disagree, Does not apply to me	
Strongly Agree, Agree, Neither agree nor disagree, Disagree, Strongly disagree	
Strongly Agree, Agree, Neither agree nor disagree, Disagree, Strongly disagree	
Strongly Agree, Agree, Neither agree nor disagree, Disagree, Strongly disagree	

For peer review only

Strongly Agree, Agree, Neither agree nor disagree, Disagree, Strongly disagree	
Strongly Agree, Agree, Neither agree nor disagree, Disagree, Strongly disagree	
Strongly Agree, Agree, Neither agree nor disagree, Disagree, Strongly disagree	
Strongly Agree, Agree, Neither agree nor disagree, Disagree, Strongly disagree	
**Overall support and care**	
Yes definitely, Yes I think so, No, I do not need a care plan, Don't know	
Yes, No, Don't know	
Yes definitely, Yes I think so, No	
Yes all of the time, Only some of the time, Never, I did not need any support	
Yes all of the time, Only some of the time, Never, My general practice is not involved, I do not need any support	
Yes definitely, Yes to some extent, No, I did not need help from health or social services, Don't know / can't remember	
Diet and lifestyle	
Physical activity and exercise	
Financial help or benefits	
Free prescriptions	
Returning to or staying in work	
Information / advice for family / friends / carer	
The physical aspects of living with and after cancer	
The psychological or emotional aspects of living with and after cancer	
I have all the information and advice I need	
**About you**<br>e to complete this questionnaire, please make sure this information is the patient's and not your own.	
In format YYYY	
Male, Female	
**Heterosexual / straight (opposite sex), Bisexual (both sexes), Gay or Lesbian (same sex), Other, Prefer not to answer**	
I live with partner / spouse / family / friends, I live alone, I live in a nursing home hospital or other long term care home, Other	
Yes, No, Don't know / can't say	
Alzheimer's disease or dementia	
Angina	
Arthritis	
Asthma or other chronic chest problem	
Blindness or visual impairment	
Deafness or hearing impairment	
Diabetes	
Epilepsy	
Heart condition	
High blood pressure	
Kidney disease	
Learning difficulty	
Liver disease	
Long term back problems	
Long-standing mental health problem	
Long-standing neurological problem	

For peer review only

Another long-standing condition	
I do not have any of these conditions	
Full time employment, Part time employment, Homemaker, Student (in education), Retired, Unemployed – and seeking work, Unemployed – unable to work for health reasons, Other	
Full time employment, Part time employment, Homemaker, Student (in education), Retired, Unemployed – and seeking work, Unemployed – unable to work for health reasons, Other	
Not working at all, Working less hours than usual, Working your usual hours, Working more hours than usual, This question does not apply to me	
British, Irish, Any other White background, White and Black Caribbean, White and Black African, White and Asian, Any other mixed background, Indian, Pakistani, Bangladeshi, Any other Asian background, Caribbean, African, Any other Black background, Chinese, Any other ethnic group	
Option to group ethnicities (e.g. white/ non-white/ unknown)	
**Free text**	

**Possible answers**	**Request Data Item**
**Your health today**<br>s section are all about your health and how you feel on the day you complete this questionnaire.	
PATIENTID	
Radiotherapy	
Chemotherapy	
Surgery	
Antibody therapy	
Stem cell transplant	
I am still having my initial treatment, It is less than 3 months since my initial treatment, It is between 3 and 12 months since my initial treatment, It is between 1 and 5 years since my initial treatment, It is more than 5 years since my initial treatment, Don't know / can't remember	
My NHL has responded fully to treatment (I am in remission), My NHL has been treated but is still present, My NHL has not been treated at all, My NHL has come back after it was originally treated, I am not certain what is happening with my NHL	
I have no problems in walking about, I have slight problems in walking about, I have moderate problems in walking about, I have severe problems in walking about, I am unable to walk about	
I have no problems washing or dressing myself, I have slight problems washing or dressing myself, I have moderate problems washing or dressing myself, I have severe problems washing or dressing myself, I am unable to wash or dress myself	
I have no problems doing my usual activities, I have slight problems doing my usual activities, I have moderate problems doing my usual activities, I have severe problems doing my usual activities, I am unable to do my usual activities	
I have no pain or discomfort, I have slight pain or discomfort, I have moderate pain or discomfort, I have severe pain or discomfort, I have extreme pain or discomfort	
I am not anxious or depressed, I am slightly anxious or depressed, I am moderately anxious or depressed, I am severely anxious or depressed, I am extremely anxious or depressed	
**Your health and wellbeing in the last 7 days**<br>stions in this section are about your health and how you have felt in the past 7 days.	
Not at all, A little bit, Somewhat, Quite a bit, Very much	
Not at all, A little bit, Somewhat, Quite a bit, Very much	

Not at all, A little bit, Somewhat, Quite a bit, Very much	
Not at all, A little bit, Somewhat, Quite a bit, Very much	
Not at all, A little bit, Somewhat, Quite a bit, Very much	
Not at all, A little bit, Somewhat, Quite a bit, Very much	
Not at all, A little bit, Somewhat, Quite a bit, Very much	
Not at all, A little bit, Somewhat, Quite a bit, Very much	
Not at all, A little bit, Somewhat, Quite a bit, Very much	
Not at all, A little bit, Somewhat, Quite a bit, Very much	
Not at all, A little bit, Somewhat, Quite a bit, Very much	
Not at all, A little bit, Somewhat, Quite a bit, Very much	
Not at all, A little bit, Somewhat, Quite a bit, Very much	
Not at all, A little bit, Somewhat, Quite a bit, Very much	
Not at all, A little bit, Somewhat, Quite a bit, Very much	
None, 1 day, 2 days, 3 days, 4 days, 5 days, 6 days, 7 days	
**Your health and wellbeing in the last month**<br>ons in this section are about your health and how you have felt during the past month.	
No difficulty, A little, Quite a bit, Very much, Does not apply	
No difficulty, A little, Quite a bit, Very much, Does not apply	
No difficulty, A little, Quite a bit, Very much, Does not apply	
No difficulty, A little, Quite a bit, Very much, Does not apply	
No difficulty, A little, Quite a bit, Very much, Does not apply	
No difficulty, A little, Quite a bit, Very much, Does not apply	
No difficulty, A little, Quite a bit, Very much, Does not apply	
No difficulty, A little, Quite a bit, Very much, Does not apply	
No difficulty, A little, Quite a bit, Very much, Does not apply	
No difficulty, A little, Quite a bit, Very much, Does not apply	
No difficulty, A little, Quite a bit, Very much, Does not apply	
No difficulty, A little, Quite a bit, Very much, Does not apply	
No difficulty, A little, Quite a bit, Very much, Does not apply	
No difficulty, A little, Quite a bit, Very much, Does not apply	
No difficulty, A little, Quite a bit, Very much, Does not apply	
No difficulty, A little, Quite a bit, Very much, Does not apply	
No difficulty, A little, Quite a bit, Very much, Does not apply	
No difficulty, A little, Quite a bit, Very much, Does not apply	
No difficulty, A little, Quite a bit, Very much, Does not apply	
No difficulty, A little, Quite a bit, Very much, Does not apply	
No difficulty, A little, Quite a bit, Very much, Does not apply	
Strongly Agree, Agree, Neither agree nor disagree, Disagree, Strongly disagree, Does not apply to me	
Strongly Agree, Agree, Neither agree nor disagree, Disagree, Strongly disagree, Does not apply to me	
Strongly Agree, Agree, Neither agree nor disagree, Disagree, Strongly disagree	
Strongly Agree, Agree, Neither agree nor disagree, Disagree, Strongly disagree	
Strongly Agree, Agree, Neither agree nor disagree, Disagree, Strongly disagree	

Strongly Agree, Agree, Neither agree nor disagree, Disagree, Strongly disagree	
Strongly Agree, Agree, Neither agree nor disagree, Disagree, Strongly disagree	
Strongly Agree, Agree, Neither agree nor disagree, Disagree, Strongly disagree	
Strongly Agree, Agree, Neither agree nor disagree, Disagree, Strongly disagree	
**Overall support and care**	
Yes definitely, Yes I think so, No, I do not need a care plan, Don't know	
Yes, No, Don't know	
Yes definitely, Yes I think so, No	
Yes all of the time, Only some of the time, Never, I did not need any support	
Yes all of the time, Only some of the time, Never, My general practice is not involved, I do not need any support	
Yes definitely, Yes to some extent, No, I did not need help from health or social services, Don't know / can't remember	
Diet and lifestyle	
Physical activity and exercise	
Financial help or benefits	
Free prescriptions	
Returning to or staying in work	
Information / advice for family / friends / carer	
The physical aspects of living with and after cancer	
The psychological or emotional aspects of living with and after cancer	
I have all the information and advice I need	
**About you**<br>e to complete this questionnaire, please make sure this information is the patient's and not your own.	
In format YYYY	
Male, Female	
**Heterosexual / straight (opposite sex), Bisexual (both sexes), Gay or Lesbian (same sex), Other, Prefer not to answer**	
I live with partner / spouse / family / friends, I live alone, I live in a nursing home hospital or other long term care home, Other	
Yes, No, Don't know / can't say	
Alzheimer's disease or dementia	
Angina	
Arthritis	
Asthma or other chronic chest problem	
Blindness or visual impairment	
Deafness or hearing impairment	
Diabetes	
Epilepsy	
Heart condition	
High blood pressure	
Kidney disease	
Learning difficulty	
Liver disease	

For peer review

Long term back problems	
Long-standing mental health problem	
Long-standing neurological problem	
Another long-standing condition	
I do not have any of these conditions	
Full time employment, Part time employment, Homemaker, Student (in education), Retired, Unemployed – and seeking work, Unemployed – unable to work for health reasons, Other	
Full time employment, Part time employment, Homemaker, Student (in education), Retired, Unemployed – and seeking work, Unemployed – unable to work for health reasons, Other	
Not working at all, Working less hours than usual, Working your usual hours, Working more hours than usual, This question does not apply to me	
British, Irish, Any other White background, White and Black Caribbean, White and Black African, White and Asian, Any other mixed background, Indian, Pakistani, Bangladeshi, Any other Asian background, Caribbean, African, Any other Black background, Chinese, Any other ethnic group	
Option to group ethnicities (e.g. white/ non-white/ unknown)	
Free text	

Possible answers	Request Data Item
**Your health today** s section are all about your health and how you feel on the day you complete this questionnaire.	
PATIENTID	
Radiotherapy	
Surgery	
Hormone Treatment	
Chemotherapy	
HIFU	
Cryotherapy	
Active surveillance	
I am still having my initial treatment, It is less than 3 months since my initial treatment, It is between 3 and 12 months since my initial treatment, It is between 1 and 5 years since my initial treatment, It is more than 5 years since my initial treatment, Don't know / can't remember	
My prostate cancer has responded fully to treatment (I am in remission), My prostate cancer has been treated but is still present, My prostate cancer has not been treated at all, My prostate cancer has come back after it was originally treated, I am not certain what is happening with my prostate cancer	
I have no problems in walking about, I have slight problems in walking about, I have moderate problems in walking about, I have severe problems in walking about, I am unable to walk about	
I have no problems washing or dressing myself, I have slight problems washing or dressing myself, I have moderate problems washing or dressing myself, I have severe problems washing or dressing myself, I am unable to wash or dress myself	
I have no problems doing my usual activities, I have slight problems doing my usual activities, I have moderate problems doing my usual activities, I have severe problems doing my usual activities, I am unable to do my usual activities	
I have no pain or discomfort, I have slight pain or discomfort, I have moderate pain or discomfort, I have severe pain or discomfort, I have extreme pain or discomfort	

For peer review only

I am not anxious or depressed, I am slightly anxious or depressed, I am moderately anxious or depressed, I am severely anxious or depressed, I am extremely anxious or depressed	
**Your health and wellbeing in the last 7 days**<br>stions in this section are about your health and how you have felt in the past 7 days.	
Not at all, A little bit, Somewhat, Quite a bit, Very much	
Not at all, A little bit, Somewhat, Quite a bit, Very much	
Not at all, A little bit, Somewhat, Quite a bit, Very much	
Not at all, A little bit, Somewhat, Quite a bit, Very much	
Not at all, A little bit, Somewhat, Quite a bit, Very much	
Not at all, A little bit, Somewhat, Quite a bit, Very much	
Not at all, A little bit, Somewhat, Quite a bit, Very much	
Not at all, A little bit, Somewhat, Quite a bit, Very much	
Not at all, A little bit, Somewhat, Quite a bit, Very much	
Not at all, A little bit, Somewhat, Quite a bit, Very much	
Not at all, A little bit, Somewhat, Quite a bit, Very much	
Not at all, A little bit, Somewhat, Quite a bit, Very much	
No, Yes	
Monthly, Weekly, Daily, Constantly, It varies	
None, 1 day, 2 days, 3 days, 4 days, 5 days, 6 days, 7 days	
**Your health and wellbeing in the last month**<br>ons in this section are about your health and how you have felt during the past month.	
No difficulty, A little, Quite a bit, Very much, Does not apply	
No difficulty, A little, Quite a bit, Very much, Does not apply	
No difficulty, A little, Quite a bit, Very much, Does not apply	
No difficulty, A little, Quite a bit, Very much, Does not apply	
No difficulty, A little, Quite a bit, Very much, Does not apply	
No difficulty, A little, Quite a bit, Very much, Does not apply	
No difficulty, A little, Quite a bit, Very much, Does not apply	
No difficulty, A little, Quite a bit, Very much, Does not apply	
No difficulty, A little, Quite a bit, Very much, Does not apply	
No difficulty, A little, Quite a bit, Very much, Does not apply	
No difficulty, A little, Quite a bit, Very much, Does not apply	
No difficulty, A little, Quite a bit, Very much, Does not apply	
No difficulty, A little, Quite a bit, Very much, Does not apply	
No difficulty, A little, Quite a bit, Very much, Does not apply	
No difficulty, A little, Quite a bit, Very much, Does not apply	
No difficulty, A little, Quite a bit, Very much, Does not apply	
No difficulty, A little, Quite a bit, Very much, Does not apply	
No difficulty, A little, Quite a bit, Very much, Does not apply	
No difficulty, A little, Quite a bit, Very much, Does not apply	
No difficulty, A little, Quite a bit, Very much, Does not apply	
No difficulty, A little, Quite a bit, Very much, Does not apply	
Strongly Agree, Agree, Neither agree nor disagree, Disagree, Strongly disagree, Does not apply to me	

For peer review only

Strongly Agree, Agree, Neither agree nor disagree, Disagree, Strongly disagree, Does not apply to me	
Strongly Agree, Agree, Neither agree nor disagree, Disagree, Strongly disagree	
Strongly Agree, Agree, Neither agree nor disagree, Disagree, Strongly disagree	
Strongly Agree, Agree, Neither agree nor disagree, Disagree, Strongly disagree	
Strongly Agree, Agree, Neither agree nor disagree, Disagree, Strongly disagree	
Strongly Agree, Agree, Neither agree nor disagree, Disagree, Strongly disagree	
Strongly Agree, Agree, Neither agree nor disagree, Disagree, Strongly disagree	
Strongly Agree, Agree, Neither agree nor disagree, Disagree, Strongly disagree	
**Overall support and care**	
Yes definitely, Yes I think so, No, I do not need a care plan, Don't know	
Yes, No, Don't know	
Yes definitely, Yes I think so, No	
Yes all of the time, Only some of the time, Never, I did not need any support	
Yes all of the time, Only some of the time, Never, My general practice is not involved, I do not need any support	
Yes definitely, Yes to some extent, No, I did not need help from health or social services, Don't know / can't remember	
Diet and lifestyle	
Physical activity and exercise	
Financial help or benefits	
Free prescriptions	
Returning to or staying in work	
Information / advice for family / friends / carer	
The physical aspects of living with and after cancer	
The psychological or emotional aspects of living with and after cancer	
I have all the information and advice I need	
**About you**	
e to complete this questionnaire, please make sure this information is the patient's and not your own.	
In format YYYY	
Male, Female	
**Heterosexual / straight (opposite sex), Bisexual (both sexes), Gay or Lesbian (same sex), Other, Prefer not to answer**	
I live with partner / spouse / family / friends, I live alone, I live in a nursing home hospital or other long term care home, Other	
Yes, No, Don't know / can't say	
Alzheimer's disease or dementia	
Angina	
Arthritis	
Asthma or other chronic chest problem	
Blindness or visual impairment	
Deafness or hearing impairment	
Diabetes	
Epilepsy	
Heart condition	
High blood pressure	
Kidney disease	

Learning difficulty	
Liver disease	
Long term back problems	
Long-standing mental health problem	
Long-standing neurological problem	
Another long-standing condition	
I do not have any of these conditions	
Full time employment, Part time employment, Homemaker, Student (in education), Retired, Unemployed – and seeking work, Unemployed – unable to work for health reasons, Other	
Full time employment, Part time employment, Homemaker, Student (in education), Retired, Unemployed – and seeking work, Unemployed – unable to work for health reasons, Other	
Not working at all, Working less hours than usual, Working your usual hours, Working more hours than usual, This question does not apply to me	
British, Irish, Any other White background, White and Black Caribbean, White and Black African, White and Asian, Any other mixed background, Indian, Pakistani, Bangladeshi, Any other Asian background, Caribbean, African, Any other Black background, Chinese, Any other ethnic group	
Option to group ethnicities (e.g. white/ non-white/ unknown)	

For peer review only

tumour types (breast, colorectal and

roqol 5 level), FACT (Functional

ry regulation covering your organisation.
nymity. Privacy by design and default

Justification

iew only

For peer review only

For peer review only

Justification

For peer review only

For peer review only

For peer review only - http://bmjopen.bmj.com/site/about/guidelines.xhtml

Justification

For peer review only

For peer review only

For peer review only

Justification

For peer review only

For peer review only - http://bmjopen.bmj.com/site/about/guidelines.xhtml

Introduction

The **National Lung Cancer Audit (LUCADA)** looks at the care delivered during refe compiled to meet the requirements of audit, and are not to be confused with the data the primary treatment package, assessing against standards and bringing about nec as it emerges.

Coverage: The audit follows patients diagnosed between: 01/01/2015 - 31/12/2013

Data item selection

Please be aware that selecting patient identifiable data items – **the data items mark** Sensitive patient demographic data, event dates and geographies will be treated as have also been applied to some of these data to provide a less disclosive alternative

Key:

Items in red Identifiable fields that require additional permissions to access (as out
Sensitive items that require additional justification in your protocol to a
Privacy by design and default' alternatives to the sensitive items highli

Item number	Data Item
1	Pseudonymised patient ID
2	Patient identifier
3	**NHS Number**
4	**Surname**
5	**Forenames**
6	**Postcode at diagnosis**
7	Postcode at diagnosis - 1st part of postcode
8	Sex
9	**Date of birth**
10	Date of birth - year
11	Date of birth - month
12	Age at diagnosis
13	Age at diagnosis in 5 year age bands (0-4 etc.)
14	Organisation code of the trust which provided the data
15	Pseudonymised organisation code of trust providing data
16	Registry Code
	This s
17	Source of referral (for cancer)
18	Cancer referral decision date
19	Cancer referral decision date - year
20	Cancer referral decision date - month
21	Days from a different date to cancer referral decision date
22	Date first seen
23	Date first seen - year
24	Date first seen - month
25	Days from a different date to date first seen
26	NCDS Lung Cancer Specialist Referral Date
27	NCDS Lung Cancer Specialist Referral Date - year
28	NCDS Lung Cancer Specialist Referral Date - month
29	Days from a different date to date of specialist referral
30	Place first seen
31	Pseudonymised place first seen
This section should be used to record details of the lung cancer diagnosis. If the ma	
32	Date of diagnosis
33	Date of diagnosis - year
34	Date of diagnosis - month
35	Days from a different date to date of diagnosis

**36**	Place of diagnosis
**37**	Pseudonymised code of place of diagnosis
**38**	Primary site diagnosis
**39**	Laterality
**40**	Basis of diagnosis
	The TNM staging data items record the definitive pre-treatment TNM stage. TNM<br>cancer care plan meeting prior to treatment and based on the clinical and patholo
**41**	TNM Classification Version Number
**42**	Staging M category
**43**	Staging N category
**44**	Staging T category
**45**	T category (Final pre treatment)
**46**	N category (Final pre treatment)
**47**	M category (Final pre treatment)
**48**	Pre-treatment stage group
**49**	Site-specific classification
**50**	Pre Treatment Histology
**51**	Lung cancer morphology
**52**	Did the patient have a staging procedure
**53**	Did patient have mediastinal sampling
**54**	Did the patient receive mediastinoscopy or mediastinomy
**55**	Did the patient have an FNA
**56**	Did the patient have any other staging procedure
**57**	Staging procedure performed but method unknown
	Following a diagnosis of cancer, a cancer care plan will be drawn up for the patient's
**58**	MDT discussion indicator
**59**	MDT discussion date
**60**	MDT discussion date - year
**61**	MDT discussion date - month
**62**	Days from a different date to date of MDT discussion
**63**	Cancer care plan intent
**64**	Care plan agreed date
**65**	Care plan agreed date- year
**66**	Care plan agreed date - month
**67**	Days from a different date to date the care plan was agreed
**68**	Intention of the treatment which is planned for the patient at this point in time.
**69**	Type of chemotherapy treatment discussed at the MDT
**70**	FEV1 (Absolute)
**71**	FEV1 (Percentage of predicted)
**72**	Performance status
	If a pat
**73**	Any significant comorbidity which prevented original treatment plan discussed at the MDT
	If co-morbidity is selected as the rea
**74**	Dementia/Cerebrovascular disease
**75**	Cardiovascular disease
**76**	Renal failure
**77**	Other malignancy
**78**	Severe weight loss
**79**	Other significant co-morbidity
**80**	The date comorbidity was recorded
**81**	The date comorbidity - year
**82**	The date comorbidity - month
**83**	Days from a different date to date the comorbidity was recorded

This section describes the key investigations used in establishing a diagnosis of lun	
**84**	Was a CT scan performed
**85**	Date of CT scan
**86**	Date of CT scan - year
**87**	Date of CT scan - month
**88**	Days from a different date to date of CT scan
**89**	Was a PET / PET CT scan performed
**90**	Date of PET / PET CT scan
**91**	Date of PET / PET CT scan - year
**92**	Date of PET / PET CT scan - month
**93**	Days from a different date to date of PET / PET CT scan
**94**	Was a bronchoscopy performed
**95**	Date of bronchoscopy
**96**	Date of bronchoscopy - year
**97**	Date of bronchoscopy - mm
**98**	Days from a different date to date of bronchoscopy
**99**	Was a CT guided biopsy performed
**100**	Date of CT guided biopsy
**101**	Date of CT guided biopsy - year
**102**	Date of CT guided biopsy - month
**103**	Days from a different date to date of CT guided biopsy
**104**	Was an other diagnostic biopsy performed
**105**	Date of other diagnostic biopsy
**106**	Date of other diagnostic biopsy - year
**107**	Date of other diagnostic biopsy - month
**108**	Days from a different date to date of other diagnostic biopsy
This section is about the input of the Lung Cancer Nurse Specialist into the lung ca receives the	
**109**	Assessed by lung cancer nurse specialist
**110**	Date of assessment by lung cancer nurse specialist
**111**	Date of assessment by lung cancer nurse specialist - year
**112**	Date of assessment by lung cancer nurse specialist - month
**113**	Days from a different date to date of assessment by CNS
**114**	How was the patient assessed by lung cancer nurse specialist
**115**	At what stage (s) in the patient journey was the patient assessed by lung cancer nurse specialist
**116**	Lung cancer nurse specialist present when patient received diagnosis
Use this	
**117**	Clinical Trial Status
**118**	Planned treatment type
**119**	Planned treatment
For Lung Cancer patients (including clinical diagnoses) Record here any surgical pro	
**120**	Hospital site code (of surgery)
**121**	Pseudonymised hospital site code (of surgery)
**122**	Date of decision to operate (surgery)
**123**	Date of decision to operate (surgery) - year
**124**	Date of decision to operate (surgery) - month
**125**	Days from a different date to date of decision to operate
**126**	Date of surgery
**127**	Date of surgery - year
**128**	Date of surgery - month
**129**	Days from a different date to date of surgery
**130**	Main surgical procedure (OPSC)

Recorded here are details relating to the completeness of the surgical resection a[illegible]	
**131**	Excision margins – R Classification
**132**	Path Stage T
**133**	Path Stage N
**134**	Path Stage M
**135**	T category (Pathological)
**136**	N category(Pathological)
**137**	M category (Pathological)
**138**	Overall Pathological TNM Stage grouping
**139**	Investigation results date (Date Specimen Reported)
**140**	Investigation results date (Date Specimen Reported) - year
**141**	Investigation results date (Date Specimen Reported) - month
**142**	Days from a different date to investigation results date
**143**	Histology
**144**	Site-specific classification
**145**	Hospital site code (of chemotherapy)
**146**	Pseudonymised hospital site code (of chemotherapy)
**147**	Date of decision to treat
**148**	Date of decision to treat - year
**149**	Date of decision to treat - month
**150**	Days from a different date to date of decision to treat
**151**	Drug treatment start date
**152**	Drug treatment start date - year
**153**	Drug treatment start date - month
**154**	Days from a different date to drug treatment start date
**155**	Chemotherapy treatment given
**156**	Hospital site code (of teletherapy)
**157**	Pseudonymised hospital site code (of teletherapy)
**158**	Date of decision to treat
**159**	Date of decision to treat - year
**160**	Date of decision to treat - month
**161**	Days from a different date to date of decision to treat
**162**	Radiotherapy treatment given
**163**	Radiotherapy anatomical treatment site
**164**	Teletherapy start date
**165**	Teletherapy start date - year
**166**	Teletherapy start date - month
**167**	Days from a different date to teletherapy start date
**168**	Hospital site code (of brachytherapy)
**169**	Pseudonymised hospital site code (of brachytherapy)
**170**	Date of decision to treat (of brachytherapy)
**171**	Date of decision to treat (of brachytherapy) - year
**172**	Date of decision to treat (of brachytherapy) - month
**173**	Days from a different date to date of decision to treat
**174**	Start date (brachytherapy treatment course)
**175**	Start date (brachytherapy treatment course) - year
**176**	Start date (brachytherapy treatment course) - month
**177**	Days from a different date to brachytherapy start date
**178**	Site code (palliative treatment course)
**179**	Pseudonymised site code (palliative treatment course)
**180**	Palliative care provider type
**181**	Palliative care community provider type
**182**	Date of decision to treat (palliative care)
**183**	Date of decision to treat (palliative care) - year

**184**	Date of decision to treat (palliative care) - month
**185**	Days from a different date to date of decision to treat
**186**	Specialist Palliative Care Start Date
**187**	Specialist Palliative Care Start Date - year
**188**	Specialist Palliative Care Start Date - month
**189**	Days from a different date to palliative care start date
**190**	Palliative care interventional treatment given
**191**	Date interventional treatment given
**192**	Date interventional treatment given - year
**193**	Date interventional treatment given - month
**194**	Days from a different date to date of interventional treatment
**195**	Hospital site code (of active monitoring)
**196**	Pseudonymised hospital site code (of active monitoring)
**197**	Decision to treat date (active monitoring)
**198**	Decision to treat date (active monitoring) - year
**199**	Decision to treat date (active monitoring) - month
**200**	Days from a different date to date of decision to treat
**200**	**Date of death**
**201**	Date of death - year
**202**	Date of death - month
**203**	Days from a different date to date of death
**204**	Was Death Related to Treatment?
**205**	Treatment Related Morbidity Type
**206**	Patient received prophylactic cranial radiation.
**207**	Was the original treatment plan carried out
**208**	Reason why original treatment plan failed

************************* **End of sheet** *************************

For peer review only

ɛrral, diagnosis, treatment and outcomes for people diagnosed with lung cancer and mesothelioma. The data ite
a items identified as Lung Cancer in the National Cancer dataset. The audit focuses on measuring the care giveı
ɛessary improvements. The project supports the Calman Hine recommendations, the National Cancer Plan and ‹

(the vital status of each patient can be followed up with linkage to Cancer Registration data)

ked below in red – will require you to have patient consent, section 251 approval, or the appropriate statutory re
de-personalised, unless anonymisation techniques are applied to render the data anonymised to k-3 anonymity
ɛ. The key explaining how these alternative can be selected is included below:

ɪlined above)
ɪccess
ighted in dark grey

Field name	Request Data Item
**Patient demographics**	
PATIENTID	
LUCADAID	
**NHS_NUMBER**	
**PERSON FAMILY NAME (Surname)**	
**PERSON NAME GIVEN (Forenames)**	
**POSTCODE**	
POSTCODE	
Sex	
**BIRTH_DATE**	
BIRTH_DATE_YYY	
BIRTH_DATE_MM	
AGE_AT_DIAGNOSIS	
FIVEYEARAGEBAND (Age at diagnosis in 5 year age bands (0-4 etc.))	
ORG_CODE_PROVIDER	
ORG_CODE_PROVIDER (pseudonymised)	
REGISTRY_CODE	
**Referral**<br>ɛection is used to record details about referral to a lung cancer specialist / team.	
SOURCE_OF_REFERRAL	
REFERRAL_DECISION_DATE	
REFERRAL_DECISION_DATE_YYYY	
REFERRAL_DECISION_DATE_MM	
Option to provide number of days from a selected date	
DATE_FIRST_SEEN	
DATE_FIRST_SEEN_YYYY	
DATE_FIRST_SEEN_MM	
Option to provide number of days from a selected date	
SPECIALIST_REFERRAL_DATE	
SPECIALIST_REFERRAL_DATE_YYYY	
SPECIALIST_REFERRAL_DATE_MM	
Option to provide number of days from a selected date	
ORG_FIRST_SEEN	
ORG_FIRST_SEEN (pseudonymised)	
**Diagnosis**<br>alignancy is discovered only at autopsy, or via details from a death certificate, then this section should be comple	
DIAGNOSIS_DATE	
DIAGNOSIS_DATE_YYYY	
DIAGNOSIS_DATE_MM	
Option to provide number of days from a selected date	

ORG_CODE_DIAGNOSIS	
ORG_CODE_DIAGNOSIS (pseudonymised)	
PRIMARY_DIAGNOSIS_ICD	
LATERALITY	
BASIS_OF_DIAGNOSIS	
**Pre-treatment staging** refers to the International Union Against Cancer's coding system (6th edition 1997, 7th edition 2009). This stagi ogical data available. Such evidence arises from physical examination, imaging, endoscopy, biopsy, surgical ex	
TNM_VERSION	
STAGING_CERTAINTY_FACTOR_M_CAT	
STAGING_CERTAINTY_FACTOR_N_CAT	
STAGING_CERTAINTY_FACTOR_T_CAT	
T_CAT_FINAL_PRE_TREAT	
N_CAT_FINAL_PRE_TREAT	
TNM_CAT_FINAL_PRE_TREAT	
TNM_CAT_FINAL_PRE_TREAT	
SITE_SPECIFIC_STAGING	
HISTOLOGY_SNOMED	
MORPHOLOGY_SNOMED	
STAGING_PROCEDURE_PERFORMED	
STG_PROC_MEDIASTIN	
MEDIASTINOSCOPY_MEDIASTINOTOMY	
STG_PROC_FNA	
STG_PROC_OTHER	
STG_PROC_UNKNOWN	
**Cancer care plan/ MDT details** s care. The cancer care plan will relate to this particular diagnosis. It is appreciated that a cancer care plan can c as more information about the stage of the disease is ascertained.	
MDT_DISCUSSION_IND	
MDT_DATE	
MDT_DATE_YYYY	
MDT_DATE_MM	
Option to provide number of days from a selected date	
CARE_PLAN_INTENT	
CARE_PLAN_AGREED_DATE	
CARE_PLAN_AGREED_DATE_YYYY	
CARE_PLAN_AGREED_DATE_MM	
Option to provide number of days from a selected date	
CANCER_TREATMENT_INTENT	
DRUG_TREATMENT_INTENT	
FEV1_ABSOLUTE_AMOUNT	
FEV1_PERCENTAGE	
PERFORMANCE_STATUS	
**Significant Co-morbidity / Treatment of Choice** tient did not receive the treatment of choice the reason should be recorded here	
SIGNIFICANT_COMORBIDITY	
**Co-morbidity** son why the patient did not receive the treatment of choice, the nature of the co-morbidity should be recorded	
DEMENTIA_CEREBROVASC_DISEASE	
CARDIOVASCULAR_DISEASE	
RENAL_FAILURE	
OTHER_MALIGNANCY	
SEVERE_WEIGHT_LOSS	
OTHER_SIGNIFICANT_CO_MORBIDITY	
DATE_COMORBIDITY_RECORDED	
DATE_COMORBIDITY_RECORDED_YYYY	
DATE_COMORBIDITY_RECORDED_MM	
Option to provide number of days from a selected date	

For peer review only

Key Investigations

ng cancer. Record all procedures used even if the result was negative. The purpose of this section is to establish in what order and whether there are delays in accessing certain services

CT_SCAN	
CT_SCAN_DATE	
CT_SCAN_DATE_YYYY	
CT_SCAN_DATE_MM	
Option to provide number of days from a selected date	
PET_OR_PET_CT_SCAN	
PET_OR_PET_CT_SCAN_DATE	
PET_OR_PET_CT_SCAN_DATE_YYYY	
PET_OR_PET_CT_SCAN_DATE_MM	
Option to provide number of days from a selected date	
BRONCHOSCOPY	
BRONCHOSCOPY_DATE	
BRONCHOSCOPY_DATE_YYYY	
BRONCHOSCOPY_DATE_MM	
Option to provide number of days from a selected date	
CT_GUIDED_BIOPSY	
CT_GUIDED_BIOPSY_DATE	
CT_GUIDED_BIOPSY_DATE_YYYY	
CT_GUIDED_BIOPSY_DATE_MM	
Option to provide number of days from a selected date	
DIAGNOSTIC_BIOPSY	
DIAGNOSTIC_BIOPSY_DATE	
DIAGNOSTIC_BIOPSY_DATE_YYYY	
DIAGNOSTIC_BIOPSY_DATE_MM	
Option to provide number of days from a selected date	

Nursing care

ancer patient journey and refers to any specialist nursing input that occurs between the patient being referred to eir primary treatment. It is not intended to record follow up information after treatment.

ASSESSED_NURSE_SPECIALIST	
DATE_ASSESSED_NURSE_SPECIALIST	
DATE_ASSESSED_NURSE_SPECIALIST_YYYY	
DATE_ASSESSED_NURSE_SPECIALIST_MM	
Option to provide number of days from a selected date	
HOW_ASSESSED_NURSE_SPECIALIST	
STAGE_PATH_ASSESSED_NURSE_SPEC	
NURSE_SPEC_PRESENT_AT_DIAG	

Treatment - Clinical trial

s section to record whether or not the patient is eligible for entry into a clinical trial

PATIENT_TRIAL_STATUS	
TREATMENT_PLAN_TYPE	
SUGGESTED_CANCER_TRT_PLAN	

Surgery

ocedure under taken with the intention of curing the patient. For Mesothelioma Patients, it is accepted that all su palliative

ORG_CODE_SURGERY	
ORG_CODE_SURGERY (pseudonymised)	
DTTD_SURGERY	
DTTD_SURGERY_YYYY	
DTTD_SURGERY_MM	
Option to provide number of days from a selected date	
PROCEDURE_DATE_SURGERY	
PROCEDURE_DATE_SURGERY_YYYY	
PROCEDURE_DATE_SURGERY_MM	
Option to provide number of days from a selected date	
PRIMARY_PROCEDURE_OPCS	

**Post surgery pathology**<br>nd the post operative staging based on the TNM system. Note that post-operative histology data is recorded in t<br>treatment window.	
EXCISION_MARGIN	
CERTAINTY_FACTOR_PATH_STAGE_T	
CERTAINTY_FACTOR_PATH_STAGE_N	
CERTAINTY_FACTOR_PATH_STAGE_M	
T_CAT_PATH	
N_CAT_PATH	
M_CAT_PATH	
TNM_CAT_PATH	
**Histology (Post Surgery)**	
INVESTIGATION_RESULT_DATE	
INVESTIGATION_RESULT_DATE_YYYY	
INVESTIGATION_RESULT_DATE_MM	
Option to provide number of days from a selected date	
HISTOLOGY_SNOMED_2	
POST_TRT_SITE_SPECIFIC_CLASS	
**Chemotherapy**	
ORG_CODE_ANTI_CANCER_TREAT	
ORG_CODE_ANTI_CANCER_TREAT (pseudonymised)	
DTTD_ANTI_CANCER_REGIMEN	
DTTD_ANTI_CANCER_REGIMEN_YYYY	
DTTD_ANTI_CANCER_REGIMEN_MM	
Option to provide number of days from a selected date	
START_DATE_ANTI_CANCER_REGIMEN	
START_DATE_ANTI_CANCER_REGIMEN_YYYY	
START_DATE_ANTI_CANCER_REGIMEN_MM	
Option to provide number of days from a selected date	
CT_TREATMENT_GIVEN	
Teletherapy (external beam radiotherapy).<br>This section is used to record the initial teletherapy treatment.	
ORG_CODE_TELETHERAPY	
ORG_CODE_TELETHERAPY (pseudonymised)	
DTTD_TELETHERAPY	
DTTD_TELETHERAPY_YYYY	
DTTD_TELETHERAPY_MM	
Option to provide number of days from a selected date	
RT_TREATMENT_GIVEN	
RT_ANATOMICAL_SITE	
START_DATE_TELETHERAPY	
START_DATE_TELETHERAPY_YYYY	
START_DATE_TELETHERAPY_MM	
Option to provide number of days from a selected date	
**Brachytherapy**	
ORG_CODE_BRACHYTHERAPY	
ORG_CODE_BRACHYTHERAPY (pseudonymised)	
DTTD_BRACHYTHERAPY	
DTTD_BRACHYTHERAPY_YYYY	
DTTD_BRACHYTHERAPY_MM	
Option to provide number of days from a selected date	
START_DATE_BRACHYTHERAPY	
START_DATE_BRACHYTHERAPY_YYYY	
START_DATE_BRACHYTHERAPY_MM	
Option to provide number of days from a selected date	
**Palliative care**	
ORG_CODE_PALLIATIVE	
ORG_CODE_PALLIATIVE (pseudonymised)	
PALL_CARE_PROV_TYPE	
PALL_CARE_COMM_PROV_TYPE	
DTTD_PALLIATIVE	
DTTD_PALLIATIVE_YYYY	

DTTD_PALLIATIVE_MM	
Option to provide number of days from a selected date	
START_DATE_PALLIATIVE	
START_DATE_PALLIATIVE_YYYY	
START_DATE_PALLIATIVE_MM	
Option to provide number of days from a selected date	
PALL_CARE_INT_TRT_GIVEN	
PALL_CARE_INT_TRT_GIVEN_DATE	
PALL_CARE_INT_TRT_GIVEN_DATE_YYYY	
PALL_CARE_INT_TRT_GIVEN_DATE_MM	
Option to provide number of days from a selected date	
**Active monitoring**	
ORG_CODE_ACTIVE_MONITORING	
ORG_CODE_ACTIVE_MONITORING (pseudonymised)	
DTTD_ACTIVE_MONITORING	
DTTD_ACTIVE_MONITORING_YYYY	
DTTD_ACTIVE_MONITORING_MM	
Option to provide number of days from a selected date	
**Outcome - death**	
DEATH_DATE	
DEATH_DATE_YYYY	
DEATH_DATE_MM	
Option to provide number of days from a selected date	
DEATH_RELATED_TO_TREATMENT	
TREATMENT_MORBIDITY_TYPE	
**Outcome - Prophylactic cranial irradiation**	
RCVD_PROPHY_CRANIAL_IRRAD	
ORIG_TRT_PLAN_CARRIED_OUT	
ORIG_TRT_PLAN_FAILURE_REASON	

For peer review only

ems in the LUCADA dataset have been
n to lung cancer patients from diagnosis to
other national guidance (e.g. NICE guidance)

egulation covering your organisation.
/. Privacy by design and default principles

Justification
eted with as much information as is available.

For peer review only

ing is usually agreed at the MDT meeting or<br>xploration and other relevant examinations.
change as the patient's disease progresses or

For peer review only

h what diagnostic techniques are being used,
the hospital to the point at which the patient
urgical procedures in this group of patients are

For peer review only

the histology window and not in the surgical

For peer review only

For peer review only

Introduction

The Quality of Lif
patients in Janua
cancer to treatme
Assessment of C

Coverage: Routi

Data item select

Please select you

Key:

Items in red

Item number
1
2
3
4
5
6
7
8
9
10
11
12
13
14
15
16
17
18
19
20
21
22
23
24
25
26
27
28
29
30
31
32
33
34
35
36
37
38
39

For peer review only

40
41
42
43
44
45
46
47
48
49
50
51
52
53
54
55
56
57
58
59
60
61
62
63
64
65
66
67
68
69
70
71
72
73
74
75
76
77
78
79
80
81
82
83
84
85
86

peer review only

87
88
89
90
91
92
93
94
95
96
97
98
99
100
101
102
103
104
105
106
107
108
109
110
111
112
113
114
115
116
117
118
119
120
121

For peer review only

ife of Colorectal Cancer Survivors in England: Patient Reported Outcome Measures includes responses from respond
ary 2013, who were alive 12-36 months after a diagnosis of colorectal cancer respondents (n=34,467) described differ
ent and through to their experiences of aftercare. The outcome questions in the survey are made up of three instrume
Cancer Therapy) and SDI (Social Difficulties Inventory). https://www.england.nhs.uk/wp-content/uploads/2015/03/color

inely linked to incident cancer cases from: 01/01/2010 - 31/12/2011, linked at a patient level

tion

ur required data items from the list below. If you require more details on the meaning and definition of specific data ite

Identifiable fields that require additional permissions to access (as outlined above)
Sensitive items that require additional justification in your protocol to access
Privacy by design and default' alternatives to the sensitive items highlighted in dark grey

Data Item	Field Name
**Your health today**	
Pseudonymised patient ID	PATIENTID
Q1. What treatments have you received for your bowel (colorectal) cancer?	q1_1
Q1. What treatments have you received for your bowel (colorectal) cancer?	q1_2
Q1. What treatments have you received for your bowel (colorectal) cancer?	q1_3
Q2. How long is it since you completed your initial treatment for bowel (colorectal) cancer?	q2
Q3. How has your bowel (colorectal) cancer responded to treatment?	q3
Q4. If you have a stoma (e.g. colostomy) is it:	q4
Q5. Mobility	q5
Q6. Self care	q6
Q7. Usual activities (work, study, housework, family or leisure activities)	q7
Q8. Pain/discomfort	q8
Q9. Anxiety/depression	q9
Q10. I have swelling or cramps in my stomach area	q10
Q11. I am losing weight	q11
Q12. I have control of my bowels	q12
Q13. I can digest my food well	q13
Q14. I have diarrhoea	q14
Q15. I have a good appetite	q15
Q16. I like the appearance of my body	q16
Q17. I have difficulty urinating	q17
Q18. I urinate more frequently than usual	q18
Q19. I leak urine	q19
Q20. Do you have an ostomy appliance/ stoma?	q20
Q21. I am embarrassed by my ostomy appliance/ stoma	q21
Q22. Caring for my ostomy appliance/ stoma is difficult	q22
Q23. Do you have any difficulty in controlling your bowels (e.g. any accidents)?	q23
Q24. If yes, how often do you have difficulties?	q24
Q25. In the past week, on how many days have you done a total of 30 minutes or more physical activity, which was enough to raise your heart rate?	q25
Q26. Have you had any difficulty in maintaining your independence	q26
Q27. Have you had any difficulty in carrying out your domestic chores?	q27
Q28. Have you had any difficulty with managing your own personal care?	q28
Q29. Have you had any difficulty with looking after those who depend on you?	q29
Q30. Have any of those close to you (e.g. partner, children, parents) had any difficulty with the support available to them?	q30
Q31. Have you had any difficulty with benefits?	q31
Q32. Have you had any financial difficulties?	q32
Q33. Have you had any difficulty with financial services?	q33
Q34. Have you had any difficulty concerning your work?	q34
Q35. Have you had any difficulty with planning for your own or your family's future?	q35
Q36. Have you had any difficulty with communicating with those closest to you?	q36

For peer review only

Q37. Have you had any difficulty with communicating with others?	q37
Q38. Have you had any difficulty concerning sexual matters?	q38
Q39. Have you had any difficulty concerning plans to have a family?	q39
Q40. Have you had any difficulty concerning your appearance or body image?	q40
Q41. Have you felt isolated?	q41
Q42. Have you had any difficulty with getting around?	q42
Q43. Have you had any difficulty with where you live?	q43
Q44. Have you had any difficulty in carrying out your recreational activities?	q44
Q45. Have you had any difficulty with your plans to travel or take a holiday?	q45
Q46. Have you had any difficulty with any other area of your everyday life?	q46
Q47. I have fears about my cancer spreading	q47
Q48. I have fears about my cancer coming back	q48
Q49. I have fears about death and dying	q49
Q50. I experience memory loss	q50
Q51. I have trouble sleeping	q51
Q52. I have trouble concentrating	q52
Q53. I always feel tired	q53
Q54. I experience mood swings	q54
Q55. I am often irritable	q55
Q56. Do you have an up to date written care plan?	q56
Q57. Do you have a named nurse who you can contact if you have a worry about your cancer care?	q57
Q58. Do you know who to contact if you have a concern about any aspect of living with or after cancer?	q58
Q59. Do you think that hospital staff did everything they could to support you following your cancer treatment?	q59
Q60. Do you think that GPs and nurses at your general practice do everything they can to support you following your cancer treatment?	q60
Q61. Following your initial cancer treatment have you been given enough care and help from health and social services (for example, district nurses, home helps or occupational therapists)?	q61
Q62. Do you consider yourself to be a	q62
Q63. If an ex-smoker, how long ago did you stop?	q63
Q64. Did you receive any advice or information on any of the following issues?	q64_1
Q64. Did you receive any advice or information on any of the following issues?	q64_2
Q64. Did you receive any advice or information on any of the following issues?	q64_3
Q64. Did you receive any advice or information on any of the following issues?	q64_4
Q64. Did you receive any advice or information on any of the following issues?	q64_5
Q64. Did you receive any advice or information on any of the following issues?	q64_6
Q64. Did you receive any advice or information on any of the following issues?	q64_7
Q64. Did you receive any advice or information on any of the following issues?	q64_8
Q64. Did you receive any advice or information on any of the following issues?	q64_9
Q64. Did you receive any advice or information on any of the following issues?	q64_10
Q64. Did you receive any advice or information on any of the following issues?	q64_11
Q65. Would it have been helpful to have had more advice or information on any of the following issues?	q65_1
Q65. Would it have been helpful to have had more advice or information on any of the following issues?	q65_2
Q65. Would it have been helpful to have had more advice or information on any of the following issues?	q65_3
Q65. Would it have been helpful to have had more advice or information on any of the following issues?	q65_4
Q65. Would it have been helpful to have had more advice or information on any of the following issues?	q65_5
Q65. Would it have been helpful to have had more advice or information on any of the following issues?	q65_6
Q65. Would it have been helpful to have had more advice or information on any of the following issues?	q65_7
Q65. Would it have been helpful to have had more advice or information on any of the following issues?	q65_8
Q65. Would it have been helpful to have had more advice or information on any of the following issues?	q65_9

For peer review only

Q65. Would it have been helpful to have had more advice or information on any of the following issues?	q65_10
Q66. What year were you born?	q66
Q67. Are you male or female?	q67
Q68. Do you look after, or give any help or support (not as part of your paid employment) to family members, friends, neighbours or others because of either: Long term physical or mental health/ disability, or Problems relating to old age	q68
Q69. Which of the following best describes your sexual orientation?	q69
Q70. Which statement best describes your living arrangements?	q70_1
Q70. Which statement best describes your living arrangements?	q70_2
Q70. Which statement best describes your living arrangements?	q70_3
Q70. Which statement best describes your living arrangements?	q70_4
Q71. Do you have a long standing health condition?	q71_1
Q71. Do you have a long standing health condition?	q71_2
Q71. Do you have a long standing health condition?	q71_3
Q72. Which, if any, of the following conditions do you have?	q72_1
Q72. Which, if any, of the following conditions do you have?	q72_2
Q72. Which, if any, of the following conditions do you have?	q72_3
Q72. Which, if any, of the following conditions do you have?	q72_4
Q72. Which, if any, of the following conditions do you have?	q72_5
Q72. Which, if any, of the following conditions do you have?	q72_6
Q72. Which, if any, of the following conditions do you have?	q72_7
Q72. Which, if any, of the following conditions do you have?	q72_8
Q72. Which, if any, of the following conditions do you have?	q72_9
Q72. Which, if any, of the following conditions do you have?	q72_10
Q72. Which, if any, of the following conditions do you have?	q72_11
Q72. Which, if any, of the following conditions do you have?	q72_12
Q72. Which, if any, of the following conditions do you have?	q72_13
Q72. Which, if any, of the following conditions do you have?	q72_14
Q72. Which, if any, of the following conditions do you have?	q72_15
Q72. Which, if any, of the following conditions do you have?	q72_16
Q72. Which, if any, of the following conditions do you have?	q72_17
Q72. Which, if any, of the following conditions do you have?	q72_18
Q73. What was your employment status before you were diagnosed with cancer?	q73
Q74. What is your employment status currently?	q74
Q75. If you are currently employed at the moment, are you:	q75
Q76. To which of these ethnic groups would you say you belong?	**q76**
Broad ethnic group	Option to group e

lents to a questionnaire distributed to colorectal cancer
rent parts of their journey from a diagnosis of colorectal
ents: the EQ-5D (Euroqol 5 level), FACT items (Functional
rectal-cancer-proms-report-140314.pdf

ems please visit: cancerproms.ncr.nhs.uk

**Request Data Item**	**Justification**

For peer review only - http://bmjopen.bmj.com/site/about/guidelines.xhtml

For peer review only

only

For peer review only

HOSPITAL EPISODE STATISTICS DATA

Hospital Episode Statistics Admitted Patient Care

Period
1997/98
1998/99
1999/00
2000/01
2001/02
2002/03
2003/04
2004/05
2005/06
2006/07
2007/08
2008/09
2009/10
2010/11
2011/12
2012/13
2013/14
2014/15
2015/16
2016/17
2017/18
2018/19

Other fields
[ACTIVAGE Age at activity date,
[ADMIAGE Age on admission,
[ADMIDATE Date of admission,
[ADMIFLAG Admission episode flag,
[ADMIMETH Method of admission,
[ADMISORC Source of admission,
[ADMISTAT Psychiatric history on admission,
[CLASSPAT Patient classification,
[DIAG_3_NN Diagnosis - 3 characters,
[Diag_4_01 Primary Diagnosis - 4 characters,
[DIAG_4_NN Diagnosis - 4 characters,
[DIAG_NN All Diagnosis codes,
[DISDATE Date of discharge,
[DISDEST Destination on discharge,
[DISFLAG Discharge episode flag,
[DISMETH Method of discharge,
[DISREADYDATE Discharge ready date,
[DOMPROC Trust derived dominant procedure,
[ENCRYPTED_HESID Encrypted HESID,

For peer review only

[EPIEND Date episode ended,
[EPIORDER Episode order,
[EPISTART Date episode started,
[EPISTAT Episode status,
[EPITYPE Episode type,
[ETHNOS Ethnic category,
[FAE Finished Admission Episode,
[FCEFLAG Finished consultant episode flag,
[HRGNHSVN Version No. of Trust derived HRG,
[IMD04 IMD Index of Multiple Deprivation,
[IMD04_DECILE IMD Decile Group,
[IMD04C IMD Crime Domain,
[IMD04ED IMD Education Training and Skills Domain,
[IMD04EM IMD Employment Deprivation Domain,
[IMD04HD IMD Health and Disability Domain,
[IMD04HS IMD Barriers to Housing and Service Domain,
[IMD04I IMD Income Domain,
[IMD04IA IMD Income affecting Adults Domain,
[IMD04IC IMD Income affecting Children Domain,
[IMD04LE IMD Living Environment Domain,
[IMD04RK IMD Overall Rank,
[LSOA01 Lower Super Output Area (LSOA01),
[LSOA11 Lower Super Output Area (LSOA11),
[MAINSPEF Main specialty,
[OPDATE_NN Date of operation,
[OPERSTAT Operation status code,
[OPERTN_4_01 Primary Operative procedure codes 4 character,
[OPERTN_4_NN All secondary Operative procedure codes 4 character,
[PCON_ONS Westminster parliamentary constituency (ONS),
[PROCODE3 Provider code - 3 character,
[PROCODE5 Provider code - 5 character,
[PROCODET Provider code,
[PROVSPNOPS Pseudonymised hospital provider spell number,
[RESGOR Government office region of residence,
[SEX Sex of patient,
[SITETRET Site code of treatment,
[STARTAGE Age at start of episode,
[SUSHRG SUS generated HRG,
[SUSHRGVERS SUS generated HRG version number,
[TRETSPEF Treatment specialty

Hospital Episode Statistics Outpatients

Periods

2003/04
2004/05
2005/06
2006/07

For peer review only

2007/08
2008/09
2009/10
2010/11
2011/12
2012/13
2013/14
2014/15
2015/16
2016/17
2017/18
2018/19

Other fields
[APPTDATE Appointment date,
[ATTENDED Attended or did not attend,
[ATTENDID Attendance identifier,
[CHAPTER Primary diagnosis chapter,
[DIAG_3_01 Primary diagnosis - 3 character,
[DIAG_3_NN Secondary diagnoses - 3 character,
[DIAG_4_01 Primary diagnosis - 4 character,
[DIAG_4_CONCAT 4 character concatenated diagnosis,
[DIAG_4_NN Secondary diagnosess - 4 character,
[DIAG_COUNT Count of diagnoses,
[DIAG_NN Diagnosis,
[DNADATE Last DNA or patient cancelled date,
[ENCRYPTED_HESID Encrypted HESID,
[ETHNOS Ethnic category,
[HRGNHS Trust derived HRG value,
[HRGNHSVN Version No. of Trust derived HRG,
[IMD04 IMD Index of Multiple Deprivation,
[MAINSPEF Main specialty,
[MYDOB Date of Birth - month and year,
[NODIAGS Number of Diagnosis,
[OPERTN_4_NN All Operative procedure codes,
[OPERTN_NN Operative procedure,
[PROCODE3 Provider code (3 character),
[PROCODE5 Provider code (5 character),
[SEX Sex of patient,
[STAFFTYP Medical staff type seeing patient,
[SUSHRG SUS generated HRG,
[SUSHRGVERS SUS generated HRG version number,
[TRETSPEF Treatment specialty,
[WAITING Days waiting

Civil Registration (Deaths) - Secondary Care Cut
Periods

For peer review only

Future 31/12/2016
Sensitive fields
[cause_of_death] Original Underlying Cause of Death,
[cause_of_death_non_neonatal] Non-neonatal cause of death,
[death_record_used] Death Record Used,
[dod] Date of Death,
[subsequent_activity] Subsequent activity

For peer review only

Protocol: observational cohort study exploring the Epidemiology of Cancer after solid Organ Transplantation (EpCOT)

Journal:	*BMJ Open*
Manuscript ID	bmjopen-2020-043731.R2
Article Type:	Protocol
Date Submitted by the Author:	28-Feb-2021
Complete List of Authors:	Sharif, Adnan; University Hospitals Birmingham NHS Foundation Trust, Renal Medicine<br>Peracha, Javeria; University Hospitals Birmingham NHS Foundation Trust, Renal Medicine<br>Winter, David; University of Birmingham<br>Reulen, Raoul; University of Birmingham<br>Hawkins, Mike; University of Birmingham, School of Health and Population Sciences
**Primary Subject Heading**:	Epidemiology
Secondary Subject Heading:	Surgery, Renal medicine, Oncology
Keywords:	TRANSPLANT SURGERY, TRANSPLANT MEDICINE, Transplant surgery < SURGERY, ONCOLOGY, Adult oncology < ONCOLOGY

SCHOLARONE™ Manuscripts

I, the Submitting Author has the right to grant and does grant on behalf of all authors of the Work (as defined in the below author licence), an exclusive licence and/or a non-exclusive licence for contributions from authors who are: i) UK Crown employees; ii) where BMJ has agreed a CC-BY licence shall apply, and/or iii) in accordance with the terms applicable for US Federal Government officers or employees acting as part of their official duties; on a worldwide, perpetual, irrevocable, royalty-free basis to BMJ Publishing Group Ltd ("BMJ") its licensees and where the relevant Journal is co-owned by BMJ to the co-owners of the Journal, to publish the Work in this journal and any other BMJ products and to exploit all rights, as set out in our licence.

The Submitting Author accepts and understands that any supply made under these terms is made by BMJ to the Submitting Author unless you are acting as an employee on behalf of your employer or a postgraduate student of an affiliated institution which is paying any applicable article publishing charge ("APC") for Open Access articles. Where the Submitting Author wishes to make the Work available on an Open Access basis (and intends to pay the relevant APC), the terms of reuse of such Open Access shall be governed by a Creative Commons licence – details of these licences and which Creative Commons licence will apply to this Work are set out in our licence referred to above.

Other than as permitted in any relevant BMJ Author's Self Archiving Policies, I confirm this Work has not been accepted for publication elsewhere, is not being considered for publication elsewhere and does not duplicate material already published. I confirm all authors consent to publication of this Work and authorise the granting of this licence.

For peer review only

Title. Protocol: observational cohort study exploring the Epidemiology of Cancer after solid Organ Transplantation (EpCOT)

Author. Adnan Sharif[1,2], Javeria Peracha[1], David L Winter[3], Raoul C Reulen[3], Mike M Hawkins[3]

Address.

[1]Department of Nephrology and Transplantation, Queen Elizabeth Hospital, University Hospitals Birmingham, Edgbaston, Birmingham, United Kingdom

[2]Institute of Immunology and Immunotherapy, University of Birmingham, Edgbaston, Birmingham, UK

[3]Institute of Applied Health Research, University of Birmingham, Edgbaston, Birmingham, UK

Corresponding author contact information.

Dr. Adnan Sharif

Department of Nephrology and Transplantation

Queen Elizabeth Hospital, University Hospitals Birmingham

Mindelsohn Way, Edgbaston

Birmingham, B15 2GW, United Kingdom

Phone: (0121 371 5861), Fax: (0121 371 5858), Email: adnan.sharif@uhb.nhs.uk, Twitter: @AdnanSharif1979, Orchid ID: 0000-0002-7586-9136

Word count. 4,081

For peer review only - http://bmjopen.bmj.com/site/about/guidelines.xhtml

Abstract

Introduction. Solid organ transplant patients are counselled regarding increased risk for cancer (principally due to their need for lifelong immunosuppression) and it ranks as one of their biggest self-reported worries. Post-transplantation cancer is common, associated with increased healthcare costs and emerging as a leading cause of post-transplant mortality. However, epidemiology of cancer post-transplantation remains poorly understood, with limitations including translating data from different countries and national data being siloed across different registries and/or data warehouses.

Methods and analysis. Study methodology for EpCOT involves record linkage between the UK Transplant Registry (from NHS Blood and Transplant), Hospital Episode Statistics (for secondary care episodes from NHS Digital), national cancer registry (from cancer registration data hosted by Public Health England) and the national death registry (from NHS Digital). Deterministic record linkage will be conducted by NHS Digital, with a fully anonymised linked dataset available for analysis by the research team. The study cohort will consist of up to 85,410 solid organ transplant recipients, who received a solid organ transplant in England between January 1st 1985 and December 31st 2015, with up-to-date outcome data.

Ethics and dissemination. This study has been approved by the Confidentiality Advisory Group (reference: 16/CAG/0121), Research Ethics Committee (reference: 15/YH/0320) and institutional review board (reference: RRK5471). The results of this study will be presented at national and international conferences, and manuscripts with results will be submitted for publication in high-impact peer-reviewed journals. The information produced will also be

For peer review only

used to develop national evidence-based clinical guidelines to inform risk stratification to enable risk-based clinical follow up.

Registration. This observational study is registered with clinicaltrials.org (NCT02991105).

Strengths and limitations of this study

- Population-based cohort linked dataset of all solid organ transplant recipients in England to provide comprehensive coverage
- A large, retrospective analysis using national linked datasets of solid organ transplant recipients at risk for developing cancer
- Robust and collaborative data linkage of routinely collected administrative data, for the purpose of exploring important clinical outcomes of research interest post-transplantation
- Missing variables and confounding data may limit interpretation of the analysis
- Data is England-specific and may not be translatable to other cohorts

For peer review only

For peer review only - http://bmjopen.bmj.com/site/about/guidelines.xhtml

Introduction

Solid organ transplantation is established therapy for patients with end-stage organ dysfunction, providing lifesaving (e.g. heart, lung, liver) or life enhancing (e.g. kidney, pancreas) treatment. However, cancer is now emerging as one of the leading complications after solid organ transplantation. Transplant recipients have an increased risk of cancer compared with that expected from the general population [1-3] and comparable risk to immunodeficiency states such as HIV/AIDS [4], with similar preponderance for cancers with a viral component [5]. Multi-factorial aetiology underlies cancer risk after solid organ transplantation but immunosuppression, compulsory to prevent allograft rejection, contributes significantly to cancer risk [6, 7]. Standardised incidence ratio for development of any post-transplantation cancers (excluding non-melanoma skin cancers) remain constantly increased starting from 2-years post-transplantation in the UK [3], meaning the relative excess risk of cancer increases with time. Transplant recipients are achieving better long-term survival [8] and, among kidney transplant patients with more than 20-years graft function, *de novo* cancer develops in 37% of patients [9]. This is likely due to improved post-transplant longevity leading to increased cumulative exposure to cancer risks such as immunosuppression. In summary, post-transplantation cancer is now the leading cause of death after kidney transplantation in the UK [10], death from cancer post-transplantation is in excess of that expected from the general population [11-14], it ranks as one of the biggest worries for post-transplant patients [15] and development of post-transplantation cancer is associated with increased healthcare cost [16].

For peer review only

Some reports on cancer epidemiology after solid organ transplantation focus on historical eras and practice has changed over the last decade. Immunosuppression is significantly different, especially since 2006 when dissemination of the SYMPHONY study results led to switches from ciclosporin to tacrolimus-based immunosuppression [17]. This is important as tacrolimus has been shown in liver transplant recipients to increase risk for post-transplantation cancer compared to ciclosporin [18], with an observed dose-effect relationship between tacrolimus and post-transplantation cancer [19]. Cancer risk appears decreased with sirolimus after kidney transplantation [20, 21], increased with azathioprine after solid organ transplantation [22-24] but some studies (limited to kidney transplantation) suggest no difference in cancer-risk between immunosuppressant agents [25]. Induction immunosuppression is also more common used; while T-cell depletion induction has been linked with increased risk for post-transplantation cancer [26], induction therapy can lead to reduced exposure to maintenance immunosuppression further confounding cancer risk assessment [27]. Evolving immunosuppression, changing transplant practice and selection of solid organ donors/recipients with different characteristics justifies a more contemporary and time-dependent analysis of post-transplantation cancer.

Data related to the epidemiology of post-transplantation cancer predominantly comes from outside the UK (e.g. the US) but this may not be directly translatable. Immunosuppression use, the biggest modifiable risk factor for post-transplantation cancer [28], differs between the US and UK. For example, T-cell depletion therapy is the predominant induction agent in the US versus non T-cell depletion (e.g. basiliximab) in the UK [29]. Cumulative exposure to immunosuppression will be different, with financial coverage limitations in the US extending

For peer review only - http://bmjopen.bmj.com/site/about/guidelines.xhtml

to immunosuppression provision [30] and these factors could possibly contribute to inferior post-transplant outcomes observed in the US compared to Europe [31, 32]. Kidney allograft recipients in England have more pre-transplant cancer history, more post-transplant cancer occurrence but superior overall graft survival and all-cause mortality when compared to contemporaneous recipients in New York State [33], suggesting post-transplantation cancer risk (and outcomes) are not comparable. Study cohorts from Australia and/or New Zealand are more aligned to UK practice from an immunosuppression perspective. However, they have different demographics (e.g., less minority ethnic recipients) and burden of medical co-morbidities (e.g., cause of kidney failure) that limits direct translation of outcomes [34]. With only two epidemiology studies focused on post-transplantation cancer in the UK [3, 13], further studies in the UK are urgently warranted to inform patients and professionals.

Absence of relevant epidemiological data impacts upon patient counselling regarding increased risk for post-transplantation cancer, which is accepted by transplant candidates as a recognised complication from immunosuppression. However, individual risk stratification is not possible and this has implications in the contemporary era of consent [35]. Personalised counselling is difficult as we cannot predict individual post-transplant cancer risk and screening is only available for a minority of cancers. Gaining a clearer understanding of post-transplantation cancer risk, morbidity and mortality for specific transplant groups/cohorts will aid transplant discussions. This is especially important for cancers of greater incidence and risk of adverse outcomes after solid organ transplantation (e.g., post-transplant lymphoproliferative disorders). KDIGO (Kidney Disease Improving Global Outcomes) guidelines for the care of (kidney) transplant patients report very poor level evidence when it

For peer review only - http://bmjopen.bmj.com/site/about/guidelines.xhtml

comes to cancer, with either level 2C or non-graded evidence [36]. This translates into lack of consensus on cancer screening advice post-transplantation [37], lack of evidence-based guidance on how best to manage development of post-transplant cancer [38] and overall poor outcomes post-transplantation if transplant recipients develop cancer [11-13].

To understand the epidemiology of post-transplant cancer in the UK, it is imperative to bring together multiple sources of information relating to the same solid organ transplant recipient who may develop cancer contained across different registries or datasets. Big data linkage, the process of bringing together and linking large volumes of healthcare records, can help identify factors and associations that would otherwise be difficult to robustly determine. The ability to link data can significantly increase the value that can be derived from individual datasets – which are often collected for specific regulatory purposes with considerable effort and expense – and maximise research benefit for patients. To that effect, Epidemiology of Cancer after solid Organ Transplantation (EpCOT) has the aim of exploring prespecified research questions of importance to the solid organ transplant community utilising big data record linkage of national healthcare datasets. The study protocol is registered with clinicaltrials.org (NCT02991105) and study results will be reported in accordance with STROBE guidelines [39].

Methods and Analysis

This epidemiological study will undertake record linkage between national datasets to link up the pathway from solid organ transplantation and subsequent risk for developing cancer. Deterministic record linkage will be undertaken by NHS Digital using identifiable patient data (specifically NHS number, date of birth, sex and postcode) between the following datasets:

For peer review only - http://bmjopen.bmj.com/site/about/guidelines.xhtml

(i) *UK Transplant Registry (UKTR)*

The UKTR is maintained by NHS Blood and Transplant and holds data for all solid organ transplants performed in the UK, with data submission mandatory for transplant centres. UKTR will provide data for the central study cohort of 85,410 patients who received a solid organ transplant between 1st January 1985 and 31st December 2015 in England, with donor-, recipient- and transplant-specific variables. An overview of the cohort is provided in Table 1, with key demographics shown in Table 2 and the data dictionary in Supplementary File 1. The final cohort for analysis will be determined after excluding any repeat transplant recipients (only the first transplant will be considered in the analysis), non-English residents who received their transplant in England and anyone who refused consent for their data to be used.

(ii) *Civil registration deaths (NHS Digital)*

National death registration data is held by NHS Digital. For those patients that have died, the date and underlying cause of death from death certificate codes will be obtained for any death occurring in the United Kingdom (any deaths occurring outside of the United Kingdom will be missed but date of emigration [loss to follow up] will be provided). For date of death only a flag indicating that death has occurred and the months from transplantation to death (to calculate time interval) will be processed in line with legal approval.

(iii) *National cancer registry - National Cancer Registration and Analysis Service (NCRAS) from Public Health England (PHE)*

For peer review only - http://bmjopen.bmj.com/site/about/guidelines.xhtml

NCRAS collects information about every patient diagnosed with cancer in England. All variables of cancer-related interest will be linked to our cohort (between 1st January 1986 and 31st December 2017 unless stated otherwise) and include; cancer site (C00c-C097x and D00-D48), histology, treatment, chemotherapy (between 1st April 2012 and 31st March 2018) and radiotherapy (between 1st April 2009 and 31st March 2019). The data dictionary is provided in Supplementary File 2.

(iv) Hospital Episode Statistics (HES)

HES is an administrative dataset collating information on all secondary care events in England, held by NHS Digital. From HES we can determine adverse health outcomes or procedures requiring hospitalisation (e.g. inpatient, outpatient and A&E attendance), for which the risk may be increased as a result of having had a solid organ transplant. Period at risk of adverse health outcomes would be from 1997 until the most recent date for which HES is known to be complete. The data dictionary is provided in Supplementary File 3.

Record linkage methodology

Data linkage methodology will involve UK Transplant Registry and NCRAS sending to NHS Digital only a list of identifying details for their respective cohorts. NHS Digital will link the UK Transplant Registry and NCRAS cohorts, determining which patients are common in both data sets. NHS Digital will then provide a list of pseudonymous IDs of patients common in both data sets to UK Transplant Registry and NCRAS respectively. This will facilitate extraction of clinical/health data from their data sets respectively, for provision to the University of Birmingham identifiable by the pseudonymous ID only. NHS Digital will facilitate linkage to

For peer review only - http://bmjopen.bmj.com/site/about/guidelines.xhtml

HES and 'Civil Registration – Deaths' to extract records and supply anonymised records to University of Birmingham with the common pseudonymous ID. The University of Birmingham will then facilitate linkage between the NHS Digital, UKTR and NCRAS datasets using the pseudonymous ID common to all datasets. See Figure 1 for a summary of data flows for the EpCOT study.

Data security and governance

There will be no need, requirement or possibility to re-identify individuals after record linkage by the University of Birmingham (acting as Data Processor). Received data will be stored on a separate array of disks and accessed on a mapped drive as part of the University of Birmingham campus network. Access to this mapped drive will be limited to the named University researchers who can only access the University of Birmingham campus network with a personalised username/password combination. The personal computers used to access the data will have additional virus checking/data security software installed (Malwarebytes). The separate array of disks will reside in a controlled environment with access only by accredited University IT staff. The data from this disk array will be backed up under a separate storage policy, which once deleted will make the data unavailable for restore.

The linkage, processing and analysis of the data from UKTR, NCRAS and NHS Digital will only be carried out the specified researchers within the University of Birmingham as the Data Processor who are appropriately trained in data protection and confidentiality. There will be no attempt made to link any data requested under this application to any held under other Data Sharing Agreements. This database of the pseudonymised data will be accessible only to

For peer review only - http://bmjopen.bmj.com/site/about/guidelines.xhtml

named personnel within the project and will be kept in auditable documents. In addition, analysts fulfilling database administrator role (who are substantive members of the University of Birmingham) will also have access to the database but undertake no processing. At the end of the project, the data will be destroyed in line with strict policies and procedures on the destruction of data. No record level information will leave the Data Processor or aggregated data without small number suppression in line with the NHS Digital HES Analysis Guide.

Prespecified research questions

1. To compare observed and expected risks of specific causes of deaths, in particular cancer-related death, by linking the UKTR with the national death registry to obtain underlying causes of death and determine factors related to increased risk of specific causes of death post-transplantation. General population mortality rates will be used to calculate expected number of deaths from specific causes and identify subgroups of post-transplant patients (e.g. age, sex, ethnicity, organ type, etc.) at excess risk compared with expected risk.

2. Investigate survival and causes of death after cancer in post-transplant patients versus individuals from the general population with a similar *de novo* cancer of the same age, sex, and calendar year of diagnosis.

3. Compare observed and expected risks of specific cancer types post-transplantation by linking the UKTR with the national cancer registry to obtain observed numbers of cancers and determine factors related to increased risk of specific types of cancer. General population cancer incidence rates will be used to calculate expected numbers of cancers

For peer review only - http://bmjopen.bmj.com/site/about/guidelines.xhtml

of specific type and identify subgroups of post-transplant patients at excess risk of specific cancers compared with expected.

4. Estimate risk of morbidity requiring hospitalisation both generally and that associated with development of post-transplantation cancer by linking the UKTR with Hospital Episode Statistics (HES). Risk of hospital admissions and procedures (e.g. surgery) for specific morbidities will be investigated. We will calculate expected risks for specific conditions requiring hospitalisation, enabling identification of specific subgroups of post-transplant patients at excess risk compared with expected.

Detailed research plan

1: Compare observed and expected risks of specific causes of death

Each of the patients in the UKTR enters risk at the date of transplantation and contributes person-years until the exit date. Standardised mortality ratios (SMRs) and absolute excess risks (AERs) will be calculated as O/E and [(O – E)/ py]*1000, where 'O' and 'E' are the observed and expected numbers of deaths respectively and 'py' is the total person-years at risk. To examine variation in SMRs or AERs across factors (e.g. age, sex, ethnicity, organ type, period of follow-up, calendar year of diagnosis), multivariable Poisson regression models will be utilised. Cumulative incidence of deaths from specific causes, in particular cancer, will be estimated (treating other causes of death as competing risks). All statistical analyses will use STATA 16 unless otherwise stated.

2: Investigate survival and causes of death after cancer in transplant patients

For peer review only - http://bmjopen.bmj.com/site/about/guidelines.xhtml

For peer review only

Comparators will be individuals from the general population with a similar *de novo* cancer of the same age, sex and calendar year of diagnosis. On one hand, heightened surveillance among solid organ transplant survivors should detect any cancers at an early stage and, therefore, reduce cancer-related mortality. On the other hand, previous treatment with immunosuppressants may limit treatment options for cancer and hence increase cancer-specific mortality. Survival comparisons will use Cox regression. We shall compare all-cause and cause-specific mortality of patients who have undergone a solid organ transplant and developed a cancer with that of individuals who developed a similar *de novo* cancer using Cox regression and/or Poisson regression analysis. Summary data of patients from the general population who developed cancer, without any transplant exposure after record linkage by NHS Digital, will be obtained from NCRAS [40].

3: Compare observed and expected risks of specific cancer types

Data from NCRAS will identify diagnosis of post-transplantation cancers in terms of date and site/type. The initial analysis will involve constructing a table with rows corresponding to variables relating to characteristics (e.g. age, sex, organ etc.) of all transplant patients and the columns corresponding to the cancer site/types. Observed and expected numbers in each cell of this table will identify any evidence of an excess. Poisson regression as described above will be used to determine subgroups of patients at a substantially increased risk of specific cancers.

We will also compare cancer risk for transplant patients treated before/after 2006 (introduction of tacrolimus-based regimens) to evaluate whether more recently treated patients have increased/decreased post-transplantation cancer risk.

For peer review only - http://bmjopen.bmj.com/site/about/guidelines.xhtml

4: Estimate risk of morbidity requiring hospitalisation

Two types of analyses will be possible using UKTR-HES linked data:

A) Internal analyses: Risk of specific adverse outcomes post-transplantation will be compared over the period at risk using Poisson regression, to identify particular subgroups (e.g. in terms of attained age, sex, type of transplant, age at diagnosis, period of follow-up and calendar year of diagnosis) at greatest risk of the specific adverse health outcomes.

B) External analyses: Obtaining general population HES events classified by age, sex and calendar year and dividing by appropriate general population estimates will enable external comparisons. We will use Poisson regression to compare risk of adverse health outcomes, over the period at risk, between transplant patients and the general population adjusting for attained age, sex and calendar year in the model. Patient sub-groups (defined in terms of attained age, sex, type of transplant, age at diagnosis, period of follow-up and calendar year of diagnosis) at substantial excess risk compared with expected will thus be identified.

Complementary to our analyses specified above, a data-driven methodology incorporating latest developments in Artificial Intelligence (e.g. machine learning) will be explored, enabling us to rank the importance of predictors for post-transplantation cancer if possible. By developing and applying models to approximate complex and yet-unknown interactions between clinical features, better prediction of patient outcomes could inform certain streams of post-transplantation care.

For peer review only - http://bmjopen.bmj.com/site/about/guidelines.xhtml

Analytical considerations

Adequate power for study events

In previous work by Collett et al. [3], 37,617 solid organ transplant recipients in the UK were analysed, whilst in the current proposal we include more than double (N=85,410, although final cohort after exclusions still to be determined). Using a conservative approach, assuming number of expected cancers will be twice that from previous work by Collett and colleagues (although expected number will likely be much greater as the cohort has aged), then we will be able to detect SIRs, of magnitudes as shown in Table 3, with 80% power when using a 5% significance level.

Thus, apart from cancer sites of anus, lip and Kaposi sarcoma which are the commonest occurring, we should be able to detect an SIR of 2.0 or less with at least 80% statistical power. Similar logic would apply to our analyses relating to hospitalisations and mortality.

Missing data

Missing data is a reality of both administrative and registry data. Examples of the degree of missing data from the UKTR has been indicated in Table 2, but the final amount after record linkage is undertaken will not be known and in some instances could be reduced (e.g. missing ethnicity data in UKTR may be available from HES or NCRAS which also contain this data field).

Our approach for dealing with missing data will be to only analyse the available data (i.e. ignoring the missing data) and conduct sensitivity analyses to explore whether missing data

For peer review only - http://bmjopen.bmj.com/site/about/guidelines.xhtml

could have biased our results; or, where appropriate, to impute missing data and account for the fact that missing values were imputed with uncertainty (e.g. multiple imputation).

Quality assurance for submitted data and record linkage

Record linkage will be facilitated by NHS Digital. The research team is therefore reliant on robust quality assurance mechanisms from both data senders and NHS Digital Data Linkage and Extract Services to ensure a robust and comprehensive record linkage process. We have worked very closely with all partners, carefully selecting fields from the available data dictionaries and checking their suitability and viability for record linkage. Cost recovery frameworks have been factored into our budget to ensure robust data extraction and quality assurance processes are in place. NHS Digital charges also includes list cleaning, a validation process using demographic data to ensure accuracy and improve linkage outcomes. A record linkage report will be available to check record linkage success rate.

Patient and Public involvement (PPI)

Patient involvement has been critical in the design of our research aims, to ensure our research questions are both relevant and appropriate. We have developed our research aims after discussion with key stakeholders like patients to ensure we are seeking answers to questions that they wish to be answered and further study into post-transplantation cancer meets those needs. PPI group meetings involving transplant candidates and transplant recipients, hosted as part of the Trust's initiative to ensure adequate patient and public representation in research drives, has confirmed qualitative study findings that post-transplantation cancer is a leading concern and an area where preventative strategies would

For peer review only - http://bmjopen.bmj.com/site/about/guidelines.xhtml

be overwhelmingly welcomed. Patients were very receptive to our strategy of record linkage, both using anonymised national data resources and also using linked primary/secondary care data after informed consent if required for any future extension work.

Ethics and dissemination

Approvals for record linkage

The EpCOT study research plan has been reviewed and approved by our data contributing partners NHS Digital, the UK Transplant Registry and Public Health England. Their support, collaboration and advice has been critical for the development of data flows and we plan to work in close partnership at various stages of the project. Our wide consortium of partners has demonstrated their strong support to ensure the success of this project over many years of development.

The EpCOT project has attained institutional (*RRK5471*) and ethical (*15/YH/0320*) approval. The project also has section 251 approval from the Confidentiality Advisory Group (*16/CAG/0121*), under Regulation 5 of the Health Service (Control of Patient Information) Regulations 2002, for the record linkage to proceed by NHS Digital.

Dissemination plan

The aim of this project is to produce targeted studies looking at the epidemiology of post-transplantation cancer, and this data will be disseminated through presentations at national/international congresses and submitted for publication in leading medical journals.

For peer review only - http://bmjopen.bmj.com/site/about/guidelines.xhtml

The aim is also to develop Standards of Care guidelines, working with the Standards Committee of the British Transplantation Society to provide evidence-based clinical evidence for direct patient benefit and counselling. These will be made freely available to the community at their website (https://bts.org.uk/guidelines-standards/). It is also the aim to plan patient-focused dissemination in plain English through various channels of communication for patients and the general public.

Outputs will only contain aggregate data with small numbers suppressed in line with the NHS Digital HES Analysis Guide [41]. Both NHS Blood and Transplant and Public Health England (who will provide the initial data cohorts on this project) have mechanisms for dissemination of information to both professional and general population and we will use these to share the results from EpCOT widely. We also aim to do similar research presentation at Research Open Days at both University Hospitals Birmingham and the University of Birmingham. Summary research findings are actively disseminated via social media channels (e.g. @AdnanSharif1979; @UHBResearch; @ImmunologyUoB), while tagging our collaborative partners (@NHSBT_RD; @PHE_uk; @NHSDigital). Project partners will be encouraged to disseminate their involvement through all social media channels.

Discussion

Large epidemiological studies from the US (using data from the Transplant Cancer Match Study) have recently reported post-transplant cancer epidemiology outcomes for a cohort of 221,962 solid organ transplant recipients, of whom 15,012 developed cancer [42]. One of the main findings from Noone and colleagues is cancer-related mortality increases steadily with post-transplantation time duration, reaching 15.7% of deaths (810 per 100,000 person-years)

For peer review only - http://bmjopen.bmj.com/site/about/guidelines.xhtml

after 10 or more years after transplantation. While a large cohort, one of the limitations of this dataset is the incomplete national coverage; a total of 17 cancer registries provided data regarding incident cancers (excluding nonmelanoma skin cancer) that covers approximately one-half of the US transplant population with varying years of coverage data. To the best of our knowledge, EpCOT is one of the largest population-cohort studies exploring cancer epidemiology after solid organ transplantation. Using record linkage of national datasets with extended years of coverage, this project will provide detailed information for solid organ transplant recipients with cancer-related coverage at a countrywide level.

In order to fulfil its regulatory mandate, NHS Blood and Transplant (NHSBT) collects data for all solid organ transplant recipients including long-term follow-up information. The collection of national data enables NHSBT to fulfil its statutory obligations with regard to the effective use of organs, equitable organ offering and performance monitoring of transplant centres in terms of patient and graft outcomes. Such data has tremendous analytical potential and is available for research purposes with appropriate approval. However, the exploration of certain outcomes like cancer is limited as this information is not routinely collected by NHSBT. To overcome this issue, record linkage between the transplant registry and national cancer data is essential. The methodology of EpCOT facilitates this, with the inclusion of all secondary care episodes and death registrations, to obtain a robust, comprehensive and informative population-cohort study of post-transplantation cancer. From an epidemiological perspective, this allows detailed analyses to assess crucial estimates of the impact of cancer for solid organ transplant recipients and provides coverage of the full patient journey from transplantation to cancer-related outcomes.

For peer review only - http://bmjopen.bmj.com/site/about/guidelines.xhtml

In summary, cancer is a leading cause of morbidity and mortality after solid organ transplantation. With incidence of cancer projected to increase, improving our understanding of post-transplant cancer epidemiology is essential to improve long-term patient outcomes. EpCOT will be one of the largest record linkages of solid organ transplant recipients, with contemporary and comprehensive national cancer-related outcomes, to provide answers to questions of importance to both transplant professionals and organ recipients respectively.

References:

1. Engels EA, Pfeiffer RM, Fraumeni JF, Jr., Kasiske BL, Israni AK, Snyder JJ, Wolfe RA, Goodrich NP, Bayakly AR, Clarke CA *et al*: **Spectrum of cancer risk among US solid organ transplant recipients**. *JAMA* 2011, **306**(17):1891-1901.
2. Kasiske BL, Snyder JJ, Gilbertson DT, Wang C: **Cancer after kidney transplantation in the United States**. *Am J Transplant* 2004, **4**(6):905-913.
3. Collett D, Mumford L, Banner NR, Neuberger J, Watson C: **Comparison of the incidence of malignancy in recipients of different types of organ: a UK Registry audit**. *Am J Transplant* 2010, **10**(8):1889-1896.
4. Grulich AE, van Leeuwen MT, Falster MO, Vajdic CM: **Incidence of cancers in people with HIV/AIDS compared with immunosuppressed transplant recipients: a meta-analysis**. *Lancet* 2007, **370**(9581):59-67.
5. Morath C, Mueller M, Goldschmidt H, Schwenger V, Opelz G, Zeier M: **Malignancy in renal transplantation**. *J Am Soc Nephrol* 2004, **15**(6):1582-1588.
6. Vajdic CM, van Leeuwen MT: **Cancer incidence and risk factors after solid organ transplantation**. *Int J Cancer* 2009, **125**(8):1747-1754.
7. Geissler EK: **Post-transplantation malignancies: here today, gone tomorrow?** *Nat Rev Clin Oncol* 2015, **12**(12):705-717.
8. **UK Transplant Activity Report 2019-2019. Accessed 5th September 2019 at; https://nhsbtdbe.blob.core.windows.net/umbraco-assets-corp/16469/organ-donation-and-transplantation-activity-report-2018-2019.pdf** Accessed 29th May 2018.
9. McCaughan JA, Courtney AE: **The clinical course of kidney transplant recipients after 20 years of graft function**. *Am J Transplant* 2015, **15**(3):734-740.
10. Steenkamp R, Rao A, Fraser S: **UK Renal Registry 18th Annual Report (December 2015) Chapter 5: Survival and Causes of Death in UK Adult Patients on Renal Replacement Therapy in 2014: National and Centre-specific Analyses**. *Nephron* 2016, **132 Suppl 1**:111-144.

For peer review only - http://bmjopen.bmj.com/site/about/guidelines.xhtml

11. Na R, Grulich AE, Meagher NS, McCaughan GW, Keogh AM, Vajdic CM: **De novo cancer-related death in Australian liver and cardiothoracic transplant recipients**. *Am J Transplant* 2013, **13**(5):1296-1304.
12. Acuna SA, Fernandes KA, Daly C, Hicks LK, Sutradhar R, Kim SJ, Baxter NN: **Cancer Mortality Among Recipients of Solid-Organ Transplantation in Ontario, Canada**. *JAMA Oncol* 2016, **2**(4):463-469.
13. Farrugia D, Mahboob S, Cheshire J, Begaj I, Khosla S, Ray D, Sharif A: **Malignancy-related mortality following kidney transplantation is common**. *Kidney Int* 2014, **85**(6):1395-1403.
14. Kiberd BA, Rose C, Gill JS: **Cancer mortality in kidney transplantation**. *Am J Transplant* 2009, **9**(8):1868-1875.
15. Howell M, Tong A, Wong G, Craig JC, Howard K: **Important outcomes for kidney transplant recipients: a nominal group and qualitative study**. *Am J Kidney Dis* 2012, **60**(2):186-196.
16. Dharnidharka VR, Naik AS, Axelrod D, Schnitzler MA, Xiao H, Brennan DC, Segev DL, Randall H, Chen J, Kasiske B *et al*: **Clinical and Economic Consequences of Early Cancer After Kidney Transplantation in Contemporary Practice**. *Transplantation* 2017, **101**(4):858-866.
17. Ekberg H, Tedesco-Silva H, Demirbas A, Vitko S, Nashan B, Gurkan A, Margreiter R, Hugo C, Grinyo JM, Frei U *et al*: **Reduced exposure to calcineurin inhibitors in renal transplantation**. *N Engl J Med* 2007, **357**(25):2562-2575.
18. Wimmer CD, Angele MK, Schwarz B, Pratschke S, Rentsch M, Khandoga A, Guba M, Jauch KW, Bruns C, Graeb C: **Impact of cyclosporine versus tacrolimus on the incidence of de novo malignancy following liver transplantation: a single center experience with 609 patients**. *Transpl Int* 2013, **26**(10):999-1006.
19. Carenco C, Assenat E, Faure S, Duny Y, Danan G, Bismuth M, Herrero A, Jung B, Ursic-Bedoya J, Jaber S *et al*: **Tacrolimus and the risk of solid cancers after liver transplant: a dose effect relationship**. *Am J Transplant* 2015, **15**(3):678-686.
20. Yanik EL, Siddiqui K, Engels EA: **Sirolimus effects on cancer incidence after kidney transplantation: a meta-analysis**. *Cancer Med* 2015, **4**(9):1448-1459.
21. Sharif A: **Sirolimus after kidney transplantation**. *BMJ* 2014, **349**:g6808.
22. Jiyad Z, Olsen CM, Burke MT, Isbel NM, Green AC: **Azathioprine and Risk of Skin Cancer in Organ Transplant Recipients: Systematic Review and Meta-Analysis**. *Am J Transplant* 2016, **16**(12):3490-3503.
23. Na R, Laaksonen MA, Grulich AE, Meagher NS, McCaughan GW, Keogh AM, Vajdic CM: **Iatrogenic immunosuppression and risk of non-Hodgkin lymphoma in solid organ transplantation: A population-based cohort study in Australia**. *Br J Haematol* 2016, **174**(4):550-562.
24. Na R, Laaksonen MA, Grulich AE, Meagher NS, McCaughan GW, Keogh AM, Vajdic CM: **High azathioprine dose and lip cancer risk in liver, heart, and lung transplant recipients: A population-based cohort study**. *J Am Acad Dermatol* 2016, **74**(6):1144-1152 e1146.
25. Gallagher MP, Kelly PJ, Jardine M, Perkovic V, Cass A, Craig JC, Eris J, Webster AC: **Long-term cancer risk of immunosuppressive regimens after kidney transplantation**. *J Am Soc Nephrol* 2010, **21**(5):852-858.

For peer review only - http://bmjopen.bmj.com/site/about/guidelines.xhtml

26. Hall EC, Engels EA, Pfeiffer RM, Segev DL: **Association of antibody induction immunosuppression with cancer after kidney transplantation**. *Transplantation* 2015, **99**(5):1051-1057.
27. Wiseman AC: **Induction Therapy in Renal Transplantation: Why? What Agent? What Dose? We May Never Know**. *Clin J Am Soc Nephrol* 2015, **10**(6):923-925.
28. Gutierrez-Dalmau A, Campistol JM: **Immunosuppressive therapy and malignancy in organ transplant recipients: a systematic review**. *Drugs* 2007, **67**(8):1167-1198.
29. Hardinger KL, Brennan DC, Klein CL: **Selection of induction therapy in kidney transplantation**. *Transpl Int* 2013, **26**(7):662-672.
30. Gill JS, Tonelli M: **Penny wise, pound foolish? Coverage limits on immunosuppression after kidney transplantation**. *N Engl J Med* 2012, **366**(7):586-589.
31. Ojo AO, Morales JM, Gonzalez-Molina M, Steffick DE, Luan FL, Merion RM, Ojo T, Moreso F, Arias M, Campistol JM *et al*: **Comparison of the long-term outcomes of kidney transplantation: USA versus Spain**. *Nephrol Dial Transplant* 2013, **28**(1):213-220.
32. Gondos A, Dohler B, Brenner H, Opelz G: **Kidney graft survival in Europe and the United States: strikingly different long-term outcomes**. *Transplantation* 2013, **95**(2):267-274.
33. Jackson-Spence F, Gillott H, Tahir S, Nath J, Mytton J, Evison F, Sharif A: **Cancer-related outcomes in kidney allograft recipients in England versus New York State: a comparative population-cohort analysis between 2003 and 2013**. *Cancer Med* 2017, **6**(3):563-571.
34. Au EH, Chapman JR, Craig JC, Lim WH, Teixeira-Pinto A, Ullah S, McDonald S, Wong G: **Overall and Site-Specific Cancer Mortality in Patients on Dialysis and after Kidney Transplant**. *J Am Soc Nephrol* 2019.
35. Lee A: **'Bolam' to 'Montgomery' is result of evolutionary change of medical practice towards 'patient-centred care'**. *Postgrad Med J* 2017, **93**(1095):46-50.
36. Kidney Disease: Improving Global Outcomes Transplant Work G: **KDIGO clinical practice guideline for the care of kidney transplant recipients**. *Am J Transplant* 2009, **9 Suppl 3**:S1-155.
37. Acuna SA, Huang JW, Scott AL, Micic S, Daly C, Brezden-Masley C, Kim SJ, Baxter NN: **Cancer Screening Recommendations for Solid Organ Transplant Recipients: A Systematic Review of Clinical Practice Guidelines**. *Am J Transplant* 2017, **17**(1):103-114.
38. Ajithkumar TV, Parkinson CA, Butler A, Hatcher HM: **Management of solid tumours in organ-transplant recipients**. *Lancet Oncol* 2007, **8**(10):921-932.
39. von Elm E, Altman DG, Egger M, Pocock SJ, Gotzsche PC, Vandenbroucke JP, Initiative S: **Strengthening the Reporting of Observational Studies in Epidemiology (STROBE) statement: guidelines for reporting observational studies**. *BMJ* 2007, **335**(7624):806-808.
40. Henson KE, Elliss-Brookes L, Coupland VH, Payne E, Vernon S, Rous B, Rashbass J: **Data Resource Profile: National Cancer Registration Dataset in England**. *Int J Epidemiol* 2020, **49**(1):16-16h.
41. **Hospital Episode Statistics (HES). Accessed 26th February 2021 at;** https://digital.nhs.uk/data-and-information/data-tools-and-services/data-services/hospital-episode-statistics.

For peer review only - http://bmjopen.bmj.com/site/about/guidelines.xhtml

42. Noone AM, Pfeiffer RM, Dorgan JF, Magder LS, Bromberg JS, Lynch CF, Morris CR, Pawlish KS, Engels EA: **Cancer-attributable mortality among solid organ transplant recipients in the United States: 1987 through 2014**. *Cancer* 2019, **125**(15):2647-2655.

For peer review only

Authors' Contributions:

Adnan Sharif: Substantial contributions to the conception, design of the work, seeking of funding, acquisition, analysis, interpretation of data, drafting the work, revising it critically and final approval of the version published

Javeria Peracha: Substantial contributions to the design of the work, analysis of data, interpretation of data, critical revision and final approval of the version published

David L Winter: Substantial contributions to the design of the work, acquisition of data, storage of data, critical revision and final approval of the version published

Raoul C Reulen: Substantial contributions to the conception, design of the work, acquisition, analysis, interpretation of data, revising it critically and final approval of the version published

Mike M Hawkins: Substantial contributions to the conception, design of the work, acquisition, analysis, interpretation of data, revising it critically and final approval of the version published

Funding Statement: This work was supported by a Wellcome Trust Institutional Strategic Support Fund: Digital Health Pilot Grant (419195-419186-42231786) and the Kildare Trust (no award number applicable).

Competing Interests Statement: No competing interests.

For peer review only

Table 1. Snapshot of the UK Transplant Registry cohort

	01/01/1985-31/12/1994	01/01/1995-31/12/2005	01/01/2006-31/12/2015	Total
**Kidney**	14341	16172	23436	53949
**Pancreas**	76	451	1835	2362
**Heart**	3271	2533	1457	7261
**Lung**	1160	1593	1683	4436
**Liver**	3296	6846	6925	17067
**Pancreas islets**	0	0	120	120
**Abdominal/intestinal**	4	47	164	215
**Total**	22148	27642	35620	85410

For peer review only

For peer review only - http://bmjopen.bmj.com/site/about/guidelines.xhtml

For peer review only

Table 2. Demographics of UK Transplant Registry cohort

		Kidney (n=53949)	Pancreas (n=2362)	Heart (n=7261)	Lung (n=4436)	Liver (n=17067)	Pancreas islets (n=120)	Abdominal / Intestinal (n=215)	Missing or unreported data
First graft		46347	2230	7115	4305	15241	74	201	0
Part of multi-organ transplant		2199	1994	1136	1027	441	0	137	0
Donor	Living	12475	0	0	24[1]	380	0	0	1
	DBD[2]	35310	2039	7246	4199	15551	110	215	
	DCD[3]	6164	323	14	224	1136	10	0	
Female recipient		20510	1006	2008	2062	7517	87	93	48
Ethnicity of recipient	White	33556	2060	3963	3237	12073	117	181	19029
	Asian	4641	115	269	75	1476	1	15	
	Black	2369	69	77	26	438	2	5	
	Chinese	300	5	11	2	107	0	0	
	Mixed	122	5	15	1	23	0	1	
	Other	545	19	39	9	408	0	3	
Recipien	0-9	1329	37	611	86	2028	0	98	22

For peer review only - http://bmjopen.bmj.com/site/about/guidelines.xhtml

t age (in years)	10-19	2860	20	733	392	946	0	15	
	20-29	6546	168	746	734	1176	0	21	
	30-39	9719	781	813	607	1745	10	23	
	40-49	12285	912	1556	864	3390	42	26	
	50-59	11972	397	2265	1288	4812	48	20	
	60-69	7733	47	531	464	2861	17	11	
	70-79	1464	0	3	1	106	3	1	
	80+	25	0	0	0	0	0	0	

[1]Partial solitary lung, [2]Donation after brain death, [3]Donation after cardiac death

For peer review only

Table 3. Adequate power for EpCOT study events

Site of cancer development	Estimated expected number of cancers	Minimal SIR that can be detected
All cancers excluding non-melanoma	1650	1.1
Skin: non-melanoma	294	1.1
Lung and Bronchus	252	1.1
Breast	243	1.1
Colorectal	200	1.2
Prostate	197	1.2
Bladder	64	1.3
Non-Hodgkin's lymphoma	56	1.3
Skin – malignant	53	1.4
Stomach	49	1.4
Oesophagus	44	1.4
Kidney	39	1.5
Pancreas	38	1.5

For peer review only - http://bmjopen.bmj.com/site/about/guidelines.xhtml

Leukaemia	37	1.5
Ovary	35	1.5
Uterus	31	1.5
Multiple myeloma	21	1.7
Cervix	18	1.8
Oral cavity	16	1.8
Liver	16	1.8
Hodgkin's lymphoma	10	1.9
Thyroid	9	2.0
Anus	5	3.0
Lip	2	4.0
Kaposi sarcoma	1.4	5.5

For peer review only

Figure legend

Figure 1. Flow of study data for the EpCOT project between data providers, NHS Digital and the University of Birmingham as Data Processor.

For peer review only - http://bmjopen.bmj.com/site/about/guidelines.xhtml

Figure 1. Flow diagram of data linkage process

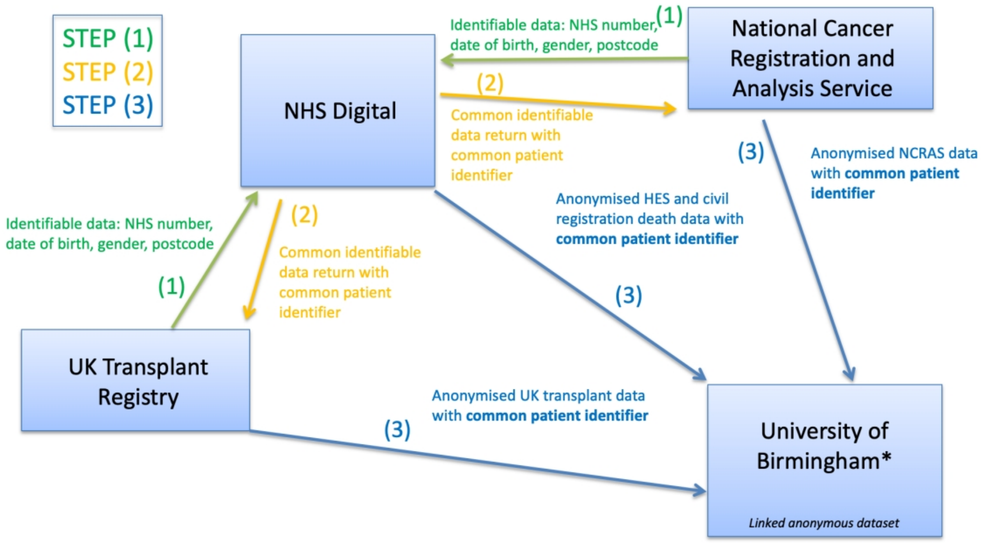

*University of Birmingham is the Data Processor. University Hospitals Birmingham NHS Foundation Trust is the Data Controller but will not receive (or access) any data at any time point.

254x190mm (200 x 200 DPI)

	**Activity/Organ(s):**
	**Geographical area:**
	**Mark data items required with an X**
**X**	**Variable**
	ARECIP_ID
	NHS_GRP
	RIMD
	RIMD_RANK
X	rhosp
X	RSEX
X	RAGE
	RAGE_GRP
	RAGE_GRP2
	PAED_AT_REG
X	RBG
	RRHESUS
x	RWEIGHT
x	RHEIGHT
X	RBMI
X	RETHNIC
X	PRD
	FPRD
	DIAB
X	WAIT_TIME
X	RCMV
X	RHCV
X	RHBV
	RHIV
X	DIAL_AT_REG
X	DIAL_AT_TX
X	DIAL_AT_TX_TYPE
	ATX_ID
X	TX_YR
	F_YR
X	TX_TYPE
	ORGAN
X	MULTI_TX
X	GRAFT_NO
X	HSP
X	CRF_TX
	rethosp
	LOCAL
	ADONOR_ID
	dd_yr
	DIMD
	DIMD_RANK
X	DTYPE
	DGRP
	DCD_CATEGORY
	RLTNSHIP
	dutilised
X	DSEX
X	DAGE

er review only

	DAGEMTHS
	DAGE_GRP
	DAGE_GRP2
x	DBG
X	DHEIGHT
X	DWEIGHT
X	DBMI
X	DETHNIC
x	DCOD
X	CIT_MINS
	CIT_HRS
	DURINE_OUTPUT_24HRS
	DURINE_OUTPUT_HR
	DURINE_PERIOD
	DURINE_PERIOD_OUTPUT
	dhosp
X	DRET_CREAT
X	DCMV
	DHBSAG
	DHBCAB
	DHCV
	DHIV
X	DEBV
	DHTLV
	DTOXO
	DSYPHILIS
	DHBCAG
	DPAST_ALCOHOL_ABUSE
	DPAST_CARDIO_DISEASE
	DPAST_DIABETES
	DPAST_FAMILY_DIABETES
	DPAST_FAMILY_DIABETES_TYPE
	DPAST_DRUG_ABUSE
	DPAST_HYPERTENSION
	DPAST_LIVER_DISEASE
	DPAST_OTHER
x	DPAST_SMOKER
x	DPAST_SMOKER_AMOUNT
x	DPAST_TUMOUR
	DPAST_UTI
	DPAST_ALLERGY
	PERSATE
	PERFQUAL
	MACHPER
	D_A_HOM
	D_B_HOM
	D_DR_HOM
	R_A_HOM
	R_B_HOM
	R_DR_HOM
	AMM_1998
	BMM_1998
	DRMM_1998

For peer review only

	AMM
	BMM
	DRMM
	mgrade
x	HLA_GRP
	matchpts
x	MATCHBTY
x	AITX_TYPE
	AITX_GRP
	AITX_FLAG
x	DGF
x	A_0
x	ALI_0
x	C_0
x	M_0
	O_0
	OKI_0
x	P_0
x	T_0
x	serum3
x	A_3
x	C_3
x	M_3
x	O_3
x	P_3
x	T_3
x	REJECTION_COUNT3
	malignancy3
	RECURRENT_DISEASE3
x	serum12
x	A_12
x	C_12
x	M_12
x	O_12
x	P_12
x	T_12
x	REJECTION_COUNT12
	malignancy12
	RECURRENT_DISEASE12
x	serum60
	A_60
	C_60
	M_60
	O_60
	P_60
	T_60
	REJECTION_COUNT60
	malignancy60
	RECURRENT_DISEASE60
x	GRAFT_OUTCOME

For peer review only - http://bmjopen.bmj.com/site/about/guidelines.xhtml

x	COF
	FCOF
	RCOD
	RCOD2
	FRCOD
	FRCOD2
	VTL_STAT
x	LAST_FUP
x	LOST_FUP
	tsurv
	tcens
x	gsurv
x	gcens
	PSURV
	PCENS

For peer review only

Kidney
United Kingdom
**Description**
Anonymised ODT recipient identification number
NHS group status
Recipient 2010 IMD deprivation score based on postcode (England)
Recipient 2010 IMD deprivation ranking order, 1 being the most deprived (England)
Anonymised unit that received and transplanted the organ
Recipient sex
Recipient age
Recipient age group, adult or paediatric
Recipient age group2, ten year increments
Does the patient have paediatric priority from registration
Recipient blood group
Recipient blood rhesus allele
Recipient weight at registration (kg)
Recipient height at registration (cm)
Recipients BMI
Recipient ethnicity
Primary renal disease
Primary renal disease in text
Was the recipient diabetic
Recipient waiting time (days)
Recipient CMV test result
Recipient HCV status
Recipient HBV status
Recipient HIV status
Dialysis status at transplant
On dialysis prior to transplant
Status of dialysis prior to transplant type
Anonymised transplant identification number
Year of transplant
Financial year of transplant
Type of transplant
Organ transplanted
Multi-organ transplant indicator
Number of kidney transplants
Flag indicating whether recipient was highly sensitised or not at time
Calculated reaction frequency at transplant
Anonymised unit that retrieved the organ
Kidney used by local centre
Anonymised donor identification number
Year of donation
Donor 2010 IMD deprivation score based on postcode (England)
Donor 2010 IMD deprivation ranking order, 1 being the most deprived (England)
Type of donor
Donor type group
Patient code for DCD donors
Relationship of donor to recipient
Donor utilised
Donor sex
Donor age

Month part of donor age (if under 3)
Donor age group, adult or paediatric
Donor age group, ten year increments
Donor blood group
Donor height (cm)
Donor weight (kg)
Donors BMI
Donor ethnicity
Donor cause of death
Cold ischaemic time (minutes)
Cold ischaemic time (hours)
Donor urine output within the last 24 hours (ml)
Donor urine output within the last hour (ml)
Donor, period or urine output (hr)
Donor urine output (ml)
Anonymised donation centre
Donor creatinine at retrieval
Donor CMV test result
Donor HBS AG test result
Donor HBCAB test result
Donor HCV test result
Donor HIV test result
Donor EBV test result
Donor HTLV test result
Donor Toxo test result
Donor syphilis test result
Donor HBCAG test result
Donor history of alcohol abuse
Donor cardio disease
Donor diabetes
Donors family history of diabetes
Donors family history type of diabete
Donor history of drug abuse
Donor hypertension
Donor liver disease
Donor past other
Donor smoker
Donor smoker amount
Donor tumour
Donor UTI
Donor allergy
Perfusate used
Perfusion quality
Machine perfusion
Donor homozygous at A locus
Donor homozygous at B locus
Donor homozygous at DR locus
Recipient homozygous at A locus
Recipient homozygous at B locus
Recipient homozygous at DR locus
Number of mismatches at A locus 1998
Number of mismatches at B locus 1998
Number of mismatches at DR locus 1998

Number of mismatches at A locus
Number of mismatches at B locus
Number of mismatches at DR locus
Matchgrade
HLA mismatch groups
Points score based on current matchability points band
Matchability
Type of antibody incompatible transplant
Group of antibody incompatible transplant
Source of antibody incompatiable transplant
Function of kidney post transplant
Azathioprine immunosuppression at transplant
ALG/ATG antibody at transplant
Ciclosporin immunosuppression at transplant
Mycophenolate immunosuppression at transplant
Other monoclonal antibody at transplant
OKT3 antibody at transplant
Predniscolone / predniscone immunosuppression at transplant
Tacrolimus immunosuppression at transplant
Serum creatinine at 3 months
Azathioprine immunosuppression at three months follow up
Ciclosporin immunosuppression at three months follow up
Mycophenolate immunosuppression at three months follow up
Other immunosuppression at three months follow up
Predniscolone / predniscone immunosuppression at three months follow up
Tacrolimus immunosuppression at three months follow up
Number of treated rejection episodes at 3 month follow up
Has the recipient developed a malignancy in the first 3 months post-transplant
Has the recipient developed evidence of a primary or recurrent renal disease (excluding infection, large vessel disease, outflow obstruction and chronic allograft nephropathy) in the graft during the frist 3 months post
Serum creatinine at 1 year
Azathioprine immunosuppression at one year follow up
Ciclosporin immunosuppression at one year follow up
Mycophenolate immunosuppression at one year follow up
Other immunosuppression at one year follow up
Predniscolone / predniscone immunosuppression at one year follow up
Tacrolimus immunosuppression at one year follow up
Number of treated rejection episodes at 1 year follow up
Has the recipient developed a malignancy in the first twelve months post-transplant
Has the recipient developed evidence of a primary or recurrent renal disease (excluding infection, large vessel disease, outflow obstruction and chronic allograft nephropathy) in the graft during the frist year post transplant.
Serum creatinine at 5 years
Azathioprine immunosuppression at five years follow up
Ciclosporin immunosuppression at five years follow up
Mycophenolate immunosuppression at five years follow up
Other immunosuppression at five years follow up
Predniscolone / predniscone immunosuppression at five years follow up
Tacrolimus immunosuppression at five years follow up
Number of treated rejection episodes within the last 12 months at 5 year follow up
Has the recipient developed a malignancy in the last 12 months at 5 years post-transplant
Has the recipient developed evidence of a primary or recurrent renal disease (excluding infection, large vessel disease, outflow obstruction and chronic allograft nephropathy) in the graft in the last 12 months at 5 years post
Last known outcome of graft

Cause of failure
Cause of failure in text
Primary recipient cause of death
Secondary recipient cause of death
Primary recipient cause of death in text
Secondary recipient cause of death in text
Recipient vital status
Last reported follow up period
Lost to follow up
Transplant survival time (days)
Transplant censoring indicator
Graft survival time (days)
Graft censoring indicator
Patient survival time (days)
Patient censoring indicator

For peer review only

Reference
1=Group 1, 2=Group 2, 8=Not reported, 9=Unknown
1=No, 2=Yes, 8=Not reported, 9=Unknown
1=Positive, 2=Negative, 8=Missing, 9=Unknown, 10=Not tested
see reference renal disease
0=No, 1=Yes
1=Negative, 2=Positive, 6=results awaited, 7=Not tested, 8=Not reported, 9=Unknown
1=Negative, 2=Positive, 6=results awaited, 7=Not tested, 8=Not reported, 9=Unknown
1=Negative, 2=Positive, 6=results awaited, 7=Not tested, 8=Not reported, 9=Unknown
1=Negative, 2=Positive, 6=results awaited, 7=Not tested, 8=Not reported, 9=Unknown
1=Haemodialysis, 2=Peritoneal dialysis, 3=Not on dialysis, 8=Not reported, 9=Unknown
1=No, 2=Yes, 8=Not reported, 9=Unknown
1=Haemodialysis, 2=Peritoneal dialysis, 3=Not on dialysis, 8=Not reported, 9=Unknown
10=Kidney, 13=En-bloc kidney, 14=Double kidney, 110=Liver & kidney,115=Liver, kidney & pancreas, 130=Kidn
10=Unspecified kidney, 11=Left kidney, 12=Right kidney
1=Kidney only, 2=Multiple organ transplant
1=No, 2=Yes, 8=Not reported, 9=Unknown
0=Imported, 1=Used by local centre
1=DBD, 2=DCD, 5=Living related, 6=Living, unrelated, 7=Domino 13=Living unrelated - Altruistic
11=After withdrawl of treatment in a patient not certified dead by brain stem tests, 12=After withdrawl of treatmer
see reference relationship
1=No, 2=Yes, 8=Not reported, 9=Unknown
1=Negative, 2=Positive, 6=results awaited, 7=Not tested, 8=Not reported, 9=Unknown

see reference donor COD

1=Negative, 2=Positive, 6=results awaited, 7=Not tested, 8=Not reported, 9=Unknown
1=Negative, 2=Positive, 6=results awaited, 7=Not tested, 8=Not reported, 9=Unknown
1=Negative, 2=Positive, 6=results awaited, 7=Not tested, 8=Not reported, 9=Unknown
1=Negative, 2=Positive, 6=results awaited, 7=Not tested, 8=Not reported, 9=Unknown
1=Negative, 2=Positive, 6=results awaited, 7=Not tested, 8=Not reported, 9=Unknown
1=Negative, 2=Positive, 6=results awaited, 7=Not tested, 8=Not reported, 9=Unknown
1=Negative, 2=Positive, 6=results awaited, 7=Not tested, 8=Not reported, 9=Unknown
1=Negative, 2=Positive, 6=results awaited, 7=Not tested, 8=Not reported, 9=Unknown
1=Negative, 2=Positive, 6=results awaited, 7=Not tested, 8=Not reported, 9=Unknown
1=Negative, 2=Positive, 6=results awaited, 7=Not tested, 8=Not reported, 9=Unknown
1=No, 2=Yes, 8=Not reported, 9=Unknown
1=No, 2=Yes, 8=Not reported, 9=Unknown
1=No, 2=Yes, 8=Not reported, 9=Unknown
1=No, 2=Yes, 8=Not reported, 9=Unknown
3=Type 1, 4=Type 2, 5=Type 1 & 2, 8=Not reported, 9=Unknown
1=No, 2=Yes, 8=Not reported, 9=Unknown
1=No, 2=Yes, 8=Not reported, 9=Unknown
1=No, 2=Yes, 8=Not reported, 9=Unknown
1=No, 2=Yes, 8=Not reported, 9=Unknown
1=No, 2=Yes, 8=Not reported, 9=Unknown

1=No, 2=Yes, 8=Not reported, 9=Unknown
1=No, 2=Yes, 8=Not reported, 9=Unknown
1=No, 2=Yes, 8=Not reported, 9=Unknown
0=No, 1=Yes
see reference perfusate used
1=No, 2=Yes Hypothermic, 3=Yes Normothermic, 8=Not reported, 9=Unknown
0=Heterozygous, 1=Homozygous, 9=Unknown
0=Heterozygous, 1=Homozygous, 9=Unknown
0=Heterozygous, 1=Homozygous, 9=Unknown
0=Heterozygous, 1=Homozygous, 9=Unknown
0=Heterozygous, 1=Homozygous, 9=Unknown
0=Heterozygous, 1=Homozygous, 9=Unknown
0=Zero mismatches, 1=One mismatch, 2=Two mismatches, 7=Not tested, 9=Unknown
0=Zero mismatches, 1=One mismatch, 2=Two mismatches, 7=Not tested, 9=Unknown
0=Zero mismatches, 1=One mismatch, 2=Two mismatches, 7=Not tested, 9=Unknown

0=Zero mismatches, 1=One mismatch, 2=Two mismatches, 7=Not tested, 9=Unknown
0=Zero mismatches, 1=One mismatch, 2=Two mismatches, 7=Not tested, 9=Unknown
0=Zero mismatches, 1=One mismatch, 2=Two mismatches, 7=Not tested, 9=Unknown
000= zero zero zero mismatches, Fave=Favourable mismatch (100,010,110), Non-fave=Non-favourable mismat
see reference Table A: HLA mismatch groups

1=HLAi, 2=ABOi, 3=Both HLAi/ABOi, 8=Not reported
HLA=HLA and HLA/ABO
FORM- Antibody incompatible form received, KTRF=Identified on kidney transplant form, ABO=Calculated from
1=Immediate, 2=Delayed, 3=Primary non-function, 8=Not reported, 9=Unknown
1=No, 2=Yes, 8=Not reported, 9=Unknown
1=No, 2=Yes, 8=Not reported, 9=Unknown
1=No, 2=Yes, 8=Not reported, 9=Unknown
1=No, 2=Yes, 8=Not reported, 9=Unknown
1=No, 2=Yes, 8=Not reported, 9=Unknown
1=No, 2=Yes, 8=Not reported, 9=Unknown
1=No, 2=Yes, 8=Not reported, 9=Unknown
1=No, 2=Yes, 8=Not reported, 9=Unknown

1=No, 2=Yes, 8=Not reported, 9=Unknown
1=No, 2=Yes, 8=Not reported, 9=Unknown
1=No, 2=Yes, 8=Not reported, 9=Unknown
1=No, 2=Yes, 8=Not reported, 9=Unknown
1=No, 2=Yes, 8=Not reported, 9=Unknown
1=No, 2=Yes, 8=Not reported, 9=Unknown

1=No, 2=Yes, 8=Not reported, 9=Unknown

1=No, 2=Yes, 8=Not reported, 9=Unknown

1=No, 2=Yes, 8=Not reported, 9=Unknown
1=No, 2=Yes, 8=Not reported, 9=Unknown
1=No, 2=Yes, 8=Not reported, 9=Unknown
1=No, 2=Yes, 8=Not reported, 9=Unknown
1=No, 2=Yes, 8=Not reported, 9=Unknown
1=No, 2=Yes, 8=Not reported, 9=Unknown

1=No, 2=Yes, 8=Not reported, 9=Unknown

1=No, 2=Yes, 8=Not reported, 9=Unknown

1=No, 2=Yes, 8=Not reported, 9=Unknown
1=No, 2=Yes, 8=Not reported, 9=Unknown
1=No, 2=Yes, 8=Not reported, 9=Unknown
1=No, 2=Yes, 8=Not reported, 9=Unknown
1=No, 2=Yes, 8=Not reported, 9=Unknown
1=No, 2=Yes, 8=Not reported, 9=Unknown

1=No, 2=Yes, 8=Not reported, 9=Unknown

1=No, 2=Yes, 8=Not reported, 9=Unknown

1=Functioning, 2=Failed, 3=Died with a functioning graft, 4=Died unknown graft function, 9=Unknown

see reference renal causes of failure

see reference recipient COD
see reference recipient COD

1=Alive, 2=Dead, 8=Not reported, 9=Unknown

1=No, 2=Yes, 8=Not reported, 9=Unknown

0=Censored, 1=Event (Graft failure or death)

0=Censored, 1=Event (Graft failure)

0=Censored, 1=Event (Death of recipient)

For peer review only

	**Activity/Organ(s):**
	**Geographical area:**
	**Mark data items required with an X**
**X**	**Variable**
	ARECIP_ID
	NHS_GRP
x	RSEX
X	RAGE
	RAGE_GRP
	RAGE_GRP2
X	RBG
	RRHESUS
X	RWEIGHT
X	RHEIGHT
X	RBMI
X	RETHNIC
X	WAIT_TIME
	PKPD
	FPKPD
X	RCMV
X	RHCV
	SURFACE_AGHBSAG
	HBSAB
	HIV
	ABOmatch
	MM_COMPLETE
	AMM
	BMM
	DRMM
	MGRADE
	HLA_GRP
X	SENSITISATION
X	DIALYSIS
	DIALYSIS_START_DATE
	MYOCARDIAL_INFARCTION
	CVA
	REG_BLIND
	LIMB_AMPUTATION
	DIABETES_CAUSE
	DIAGNOSIS_YR
	INSULIN_DOSE
	SERUM_CREATININE
	PRETX_egfr
	PIPECIGARS
x	CIGARETTES
	NODAY
	YRS_SINCE_STOP
	EXPOSURE
	ADONOR_ID
	DD_YR
X	DTYPE
X	DGRP
X	DSEX

review only

X	DAGE
	DAGE_GRP
	DAGE_GRP2
X	DBG
x	DCOD
	FDCOD
X	DHEIGHT
X	DWEIGHT
	DGIRTH
X	DBMI
X	DETHNIC
X	DCMV
	DHG
	DGLUCOSE
	DGLUCOSE_ON_ADMISSION
	DRET_CREAT
	DHCV
	DREGION
	DNATION
	DCOUNTRY
	DHBSAG
	DHBCAB
	DHIV
X	DEBV
	DHTLV
	DTOXO
	DSYPHILIS
	DHEV
	DURINE_OUTPUT_24HRS
	DURINE_OUTPUT_HR
	DURINE_PERIOD
	DURINE_PERIOD_OUTPUT
	DINSULIN
	DINSULIN_DOSE
	DCURRENT_GLUCOSE
	DINSULIN_DATE
	DINSULIN_RATE
	DPAST_ALCOHOL_ABUSE
	DALCOHOL_UNITS_AMOUNT
	DPAST_CARDIO_DISEASE
	DPAST_DIABETES
	DPAST_FAMILY_DIABETES
	DPAST_FAMILY_DIABETES_TYPE
	DPAST_DRUG_ABUSE
	DPAST_HYPERTENSION
	DPAST_LIVER_DISEASE
	DPAST_OTHER
x	DPAST_SMOKER
x	DPAST_SMOKER_AMOUNT
x	DPAST_TUMOUR
	DPAST_UTI
	DPAST_ALLERGY
	MACHINE_PRESERVATION_USED

review only

For peer review only - http://bmjopen.bmj.com/site/about/guidelines.xhtml

	WIT
	CIT
X	CIT_MINS
	ASYSTOLIC_PERIOD
	DUCT_MANAGEMENT
	QUALITY
	SENSITISATION_AT_TX
	HSP
	ATX_ID
x	TX_YR
	FYR
X	TX_TYPE
x	TX_GRP
X	PTX_NO
	HBA1C
	IFCC_HBA1C
	TMF_INITIAL_GRAFT_FUNCTION
	TMF_REJECTIONS
	TMF_RENAL_DISEASE
	TMF_INSULIN_INDEPENDENT
	TMF_INSULIN_DOSE
	TMF_BLOOD_GLUCOSE
	TMF_HBA1C
	TMF_IFCC_HBA1C
	TMF_CREATININE
x	AF1Y_REJECTIONS
x	AF1Y_RENAL_DISEASE
x	AF1Y_INSULIN_INDEPENDENT
	AF1Y_BLOOD_GLUCOSE
	AF1Y_HBA1C
	AF1Y_IFCC_HBA1C
	AF1Y_CREATININE
	AF2Y_REJECTIONS
	AF2Y_RENAL_DISEASE
	AF2Y_INSULIN_INDEPENDENT
	AF2Y_BLOOD_GLUCOSE
	AF2Y_HBA1C
	AF2Y_IFCC_HBA1C
	AF2Y_CREATININE
	AF5Y_REJECTIONS
	AF5Y_RENAL_DISEASE
	AF5Y_INSULIN_INDEPENDENT
	AF5Y_BLOOD_GLUCOSE
	AF5Y_HBA1C
	AF5Y_IFCC_HBA1C
	AF5Y_CREATININE
X	GRAFT_OUTCOME
X	COF
	FCOF
	COF_NOTE
	RCOD1
	RCOD2
	FRCOD1

review only

For peer review only - http://bmjopen.bmj.com/site/about/guidelines.xhtml

	FRCOD2
	GRAFT_FUNCTION
	RCOD_NOTE
	TSURV
	TCENS
X	GSURV
X	GCENS
	PSURV
	PCENS
	KID_DGF
	KID_OUTCOME
	KID_LFUP
	KID_GSURV
	KID_GCENS
	KID_PSURV
	KID_PCENS

For peer review only

Pancreas
**United Kingdom**
**Description**
Anonymised recipient identification number
NHS group
Recipient sex
Recipient age at transplant
Recipient paediatric or adult
Recipient age in 10 year increments
Recipient blood group
Recipient blood rhesus allele
Weight of recipient (kg)
Height of recipient (cm)
Recipient BMI
Recipient ethnicity
Waiting time for transplant (days)
Best known pancreas/renal disease at registration
Pancreas/renal diease at registration in text
Recipient CMV test result at registration
Recipient HCV test result at registration
Recipient Hepatitis B - Surface Ag(HBsAG)
Recipient if negative Hepatitis B - Surface Ag(HBsAG) then HBsAb
Recipient HIV
Donor vs Recipient blood group
Mismatch recipient
Number of mismatches at A locus
Number of mismatches at B locus
Number of mismatches at dr locus
Matchgrade
HLA mismatch groups
% sensitised against general donor population
Dialysis status of recipient at transplant
Date current dialysis started
Pre-transplant complications- Myocardial infarction
Pre-transplant complications- CVA
Pre-transplant complications- Registered blind
Pre-transplant complications- Limb amputation
Cause of diabetes
Year of diagnosis
Insulin dose prior to transplant (units/24hr)
If not on dialysis, serum creatinine prior to transplant
Pre-transplant Estimated GFR
Does the recipient smoke pipe and cigars at transplant
Does the recipient smoke cigarettes at transplant
If the recipient smokes cigarettes, how many a day at transplant?
If ex smoker how many years since stopped smoking at transplant
Total exposure in pack years at transplant
Anonymised donor identification number
Year of donation
Donor type
Donor type group
Donor sex

Donor age
Donor paediatric or adult
Donor age in 10 year increments
Donor blood group
Donor cause of death
Donor cause of death in text
Donor height (cm)
Donor weight (kg)
Donor Girth (cm)
Donor BMI
Donor ethnicity
Donor CMV test result
Donor haemoglobin at retrieval
Donor glucose at retrieval
Donor glucose on admission
Donor creatinine at retrieval
Donor HCV at retrieval
Region of donor hospital
Nation of donor hospital
Country of donor hospital
Donor HBS AG test result
Donor HBCAB test result
Donor HIV test result
Donor EBV test result
Donor HTLV test result
Donor Toxo test result
Donor syphilis test result
Donor HEV test result
Donor urine output last 24 hours (ml)
Donor urine output last hour (ml)
Donor urine last for (hrs)
Donor urine output in relation to durine_period time
Insulin adminstered to the donor
If yes what was the dose of insulin given to the donor (mg)
Donor blood glucose level mmol/l
If yes the date and time insulin was given to the donor
Donor rate of insulin infusion (ml/hr)
Donor history of alcohol abuse
Donor units of alcohol (available post January 2016)
Donor cardio disease
Donor diabetes
Donors family history of diabetes
Donors family history type of diabete
Donor history of drug abuse
Donor hypertension
Donor liver disease
Donor past other
Donor smoker
Donor smoker amount
Donor tumour
Donor UTI
Donor allergy
Machine preservation used?

Warm ischaemia time (minutes)
Cold ischaemia time (hh:mm)
Cold ischaemia time (minutes)
If donor DCD then duration of asystolic period (mins)
Type of duct management
Quality of cold perfusion
Sensitisation at transplant (%)
Flag indicating whether recipient was highly sensitised at transplant
Anonymised transplant identification number
Year of transplant
Financial year of transplant
Transplant type
Type of pancreas transplant
Number of pancreas transplants
Pre-transplant HbA1c (%) - as recorded prior to 29/1/13
Pre-transplant IFCC-HbA1c (mmol/mol) - as recorded since 29/1/13
Recipient initial graft function at 3 month fup
Number of treated rejection episodes in the first 3 months post transplant
Recurrent primary disease in pancreatic graft within the first 3 months
Has the recipient been insulin independent at all since transplant at 3 month fup
What is the insulin dose for recipient at 3 months
If insulin independent then state blood glucose (mmol/l)
If insulin independent then state HbA1c (%) - as recorded prior to 29/1/2013
If insulin independent then state IFCC-HbA1c (mmol/l) - as recorded since 29/1/2013
Serum creatinine measured between 10 and 14 weeks post-transplant
Number of treated rejection episodes in the last 12 months at 1 year fup
Recurrent primary disease in pancreatic graft within the last 12 months at 1 year fup
Has the recipient been insulin independent within the last 9 months at 1 year fup
If insulin independent then state blood glucose (mmol/l) at 1 year fup
If insulin independent then state HbA1c (%) at 1 year fup - as recorded prior to 29/1/13
If insulin independent then state IFCC-HbA1c (mmol/mol) at 1 year fup - as recorded since 29/1/13
Serum creatinine measured between 11 and 13 months post-transplant
Number of treated rejection episodes in the last 12 months at 2 year fup
Recurrent primary disease in pancreatic graft within the last 12 months at 2 year fup
Has the recipient been insulin independent within the last 12 months at 2 year fup
If insulin independent then state blood glucose (mmol/l) at 2 year fup
If insulin independent then state HbA1c (%) at 2 year fup - as recorded prior to 29/1/13
If insulin independent then state IFCC-HbA1c (mmol/mol) at 2 year fup - as recorded since 29/1/13
Serum creatinine measured at 2 years
Number of treated rejection episodes in the last 12 months at 5 years fup
Recurrent primary disease in pancreatic graft within the last 12 months at 5 years fup
Has the recipient been insulin independent within the last 12 months at 5 years fup
If insulin independent then state blood glucose (mmol/l) at 5 years fup
If insulin independent then state HbA1c (%) at 5 years fup - as recorded since 29/1/13
If insulin independent then state IFCC-HbA1c (mmol/mol) at 5 years fup - as recorded since 29/1/13
Serum creatinine measured at 5 years
Last known outcome of graft
Cause of failure
Cause of failure in text
Additional cause of failure information
Recipient primary cause of death
Recipient secondary cause of death
Recipient primary cause of death in text

Recipient secondary cause of death in text
Pancreas function at last assessment
Recipient cause of death notes
Transplant survival time (days)
Transplant censoring indicator
Graft survival time (days)
Graft censoring indicator
Patient survival time (days) from first graft only
Patient censoring indicator from first graft only
Function of kidney post transplant
Last known outcome of kidney graft
Kidney lost to fup
Kidney graft survival time (days)
Kidney graft censoring indicator
Kidney patient survival time (days)
Kidney patient censoring indicator

For peer review only

Reference
1=Group 1, 2=Group 2, 9=Unknown
1=Positive, 2=Negative, 8=Missing, 9=Unknown, 10=Not tested
see reference pancreas/renal disease
1=Negative, 2=Positive, 6=results awaited, 7=Not tested, 8=Not reported, 9=Unknown
1=Negative, 2=Positive, 6=results awaited, 7=Not tested, 8=Not reported, 9=Unknown
1=Negative, 2=Positive, 6=results awaited, 7=Not tested, 8=Not reported, 9=Unknown
1=Negative, 2=Positive, 6=results awaited, 7=Not tested, 8=Not reported, 9=Unknown
1=Negative, 2=Positive, 6=results awaited, 7=Not tested, 8=Not reported, 9=Unknown
1=Identical, 2=Compatible, 3=Incompatible, 9=Unknown match
0=Zero mismatches, 1=One mismatch, 2=Two mismatches, 7=Not tested, 9=Unknown
0=Zero mismatches, 1=One mismatch, 2=Two mismatches, 7=Not tested, 9=Unknown
0=Zero mismatches, 1=One mismatch, 2=Two mismatches, 7=Not tested, 9=Unknown
000= zero zero zero mismatches, Fave=Favourable mismatch (100,010,110), Non-fave=Non-favourable mismat
see reference Table A: HLA mismatch groups
1=Haemodialysis, 2=Peritoneal dialysis, 3=Not on dialysis, 8=Not reported, 9=Unknown
1=No, 2=Yes, 8=Not reported, 9=Unknown
1=No, 2=Yes, 8=Not reported, 9=Unknown
1=No, 2=Yes, 8=Not reported, 9=Unknown
1= No amputation, 2=Major limb amputation, 3=Digital amputation
1=Type 1, 2=Type 2, 3=Other
1=No, 2=Yes, 3=Ex-smoker, 8=Not reported, 9=Unknown
1=No, 2=Yes, 3=Ex-smoker, 8=Not reported, 9=Unknown
1=DBD, 2=DCD, 5=Living related, 6=Living, unrelated, 7=Domino 13=Living unrelated - Altruistic

see reference donor COD

1=Negative, 2=Positive, 6=results awaited, 7=Not tested, 8=Not reported, 9=Unknown

1=Negative, 2=Positive, 6=results awaited, 7=Not tested, 8=Not reported, 9=Unknown

1=Negative, 2=Positive, 6=results awaited, 7=Not tested, 8=Not reported, 9=Unknown
1=Negative, 2=Positive, 6=results awaited, 7=Not tested, 8=Not reported, 9=Unknown
1=Negative, 2=Positive, 6=results awaited, 7=Not tested, 8=Not reported, 9=Unknown
1=Negative, 2=Positive, 6=results awaited, 7=Not tested, 8=Not reported, 9=Unknown
1=Negative, 2=Positive, 6=results awaited, 7=Not tested, 8=Not reported, 9=Unknown
1=Negative, 2=Positive, 6=results awaited, 7=Not tested, 8=Not reported, 9=Unknown
1=Negative, 2=Positive, 6=results awaited, 7=Not tested, 8=Not reported, 9=Unknown
1=Negative, 2=Positive, 6=results awaited, 7=Not tested, 8=Not reported, 9=Unknown

1=No, 2=Yes, 8=Not reported, 9=Unknown

1=No, 2=Yes, 8=Not reported, 9=Unknown
1=Non-Drinker Alcohol, 2=Drinks Rarely/ Occasionally - <1 u/day, 3=Light Drinker - 1-2 u/day, 4=Moderate Drink
1=No, 2=Yes, 8=Not reported, 9=Unknown
1=No, 2=Yes, 8=Not reported, 9=Unknown
1=No, 2=Yes, 8=Not reported, 9=Unknown
3=Type 1, 4=Type 2, 5=Type 1 & 2, 8=Not reported, 9=Unknown
1=No, 2=Yes, 8=Not reported, 9=Unknown
1=No, 2=Yes, 8=Not reported, 9=Unknown
1=No, 2=Yes, 8=Not reported, 9=Unknown
1=No, 2=Yes, 8=Not reported, 9=Unknown
1=No, 2=Yes, 8=Not reported, 9=Unknown

1=No, 2=Yes, 8=Not reported, 9=Unknown
1=No, 2=Yes, 8=Not reported, 9=Unknown
1=No, 2=Yes, 8=Not reported, 9=Unknown
1=No, 2=Yes - warm, 3=Yes - cold

1=Enteric side to side, 2=Enteric - roux, 3=Bladder drainage
1=Good, 2=Fair, 3=Poor, 4=Patchy, 8=Not reported, 9=Unknown

1=No, 2=Yes, 8=Not reported, 9=Unknown

50=Pancreas only, 51=Pancreas islets, 115=Pancreas, Liver & kidney, 130=Pancreas/Kidney, 140=Pancreas &
PAK=Pancreas after kidney, PTA=Pancreas transplant alone, SPK=Simultaneous pancreas/kidney, IAK= Islets a

1=Immediate function, 2=Partial function (Insulin required), 3=Primary non-function

1=No, 2=Yes, 8=Not reported, 9=Unknown
1=No, 2=Yes, 8=Not reported, 9=Unknown

1=No, 2=Yes, 8=Not reported, 9=Unknown
1=No, 2=Yes, 8=Not reported, 9=Unknown

1=No, 2=Yes, 8=Not reported, 9=Unknown
1=No, 2=Yes, 8=Not reported, 9=Unknown

1=No, 2=Yes, 8=Not reported, 9=Unknown
1=No, 2=Yes, 8=Not reported, 12=Unknown

1=Functioning, 2=Failed, 3=Died with a functioning graft, 4=Died unknown graft function, 9=Unknown
see reference cause of failure

see reference recipient cause of death
see reference recipient cause of death

1=Insulin independent, 2=Partial function, 3=No function

0=Censored, 1=Event (Graft failure or death)

0=Censored, 1=Event (Graft failure)

0=Censored, 1=Event (Death of recipient)
1=Immediate, 2=Delayed, 3=Primary non-function, 8=Not reported, 9=Unknown
1=Functioning, 2=Failed, 3=Died with a functioning graft, 4=Died unknown graft function, 9=Unknown
1=No, 2=Yes, 8=Not reported, 9=Unknown

0=Censored, 1=Event (Graft failure)

0=Censored, 1=Event (Death of recipient)

For peer review only

	**Activity/Organ(s):**
	**Geographical area:**
	**Mark data items required with an X**
**X**	**Variable**
	ARECIP_ID
	AREGISTRATION_ID
	RPC2
	RIMD
	RIMD_RANK
X	RSEX
X	RAGE
	RAGE_GRP
X	rbg
X	RWEIGHT
X	RHEIGHT
X	RETHNIC
X	RCSPLD1
X	RCSPLD2
	RCSPLD3
X	RFAIL_GRADE
	RLIFE
	RIN_PAT
	RVENT
	RREN_SUP
	RASCITES
	RDIURETIC
	RENCEPH
	RAB_SURGERY
	RICP_MON
	ROES_VARICES
	ROES_SHUNT
	RPYREXIAL
	RSEPSIS
	RSEPSIS_SITE
	RHB
	RWBC
	RPLATELETS
	RCREAT
	RCREAT_CORR
X	RALBUMIN
	RUREA
	RINR
	RBILIRUBIN
	RSODIUM
X	UKELD
X	MELD
	RPOTASSIUM
	RPH
	ROXYGEN
X	RCMV
X	RHCV
	RHBV_DNA
	RHBS_AG

For peer review only

	RHBE_AG
	RHCV_RNA
	RANTI_HBCLGM
	RANTI_HBS
	RANTI_HBE
	RANTI_HCV
	RANTI_DELTA
	RANTI_CMV
	RANTI_HSV
	RANTI_HIV
	ATX_ID
x	TX_YR
	REREG
	rereg_yr
	RE_TXD
	nxttx_yr
X	TX_TYPE
X	MULTI_TX
	TX_METHOD
	ORGAN
	ORG_TXD
	sm_bowel
	URGENT
	su_flag
X	LIVER
	ALLTX_NO
X	GRAFT_NO
	TXNATION
	TXCOUNTRY
	ADONOR_ID
	dd_yr
X	DTYPE
	DGRP
X	DSEX
X	DAGE
	DAGE_GRP
	dbg
X	DBMI
X	DCOD
	DNATION
	DCOUNTRY
X	DCMV
	KIDDONOR
	PANDONOR
	SMBDONOR
	HRTDONOR
	LNGDONOR
	MULTIDON
	PRES_FLUID
	ORG_APP
	STEATOSIS
	CAPSULAR DAMAGE
	HEP_ARTERY

review only

	CIT
	VV_TIME
	REP_TIME
	BA
	AA
	RANTI_FIB
	IO_D
	IO_BLOOD
	IO_PLASMA
	IO_PLATELETS
	IO_CRYO
	PO_BLOOD
	PO_PLASMA
	PO_PLATELETS
	PO_CRYO
	COMMON_BILE_DUCT
	COMMON_HEP_ARTERY
	HEPATIC_ARTERY
	ILIAC_ARTERY
	ILIAC_VEIN
	LEFT_AND_RIGHT_HEPATIC
	LEFT_HEPATIC_GASTRIC
	LYMPH_NODE
	MAIN_PORTAL_VEIN
	NORMAL_ANATOMY
	PATCH
	RIGHT_HEPATIC_SMA
	SPLEEN
	VENA_CAVA
	TMF_LIFESTYLE
	TMF_REJECTION_EPISODES
	TMF_DAYS_VENTILATED
	TMF_DAYS_IN_ITU
	TMF_RENAL_STATUS
	TMF_HEPATIC_ART_THROMBOSIS
	TMF_PORTAL_VEIN_THROMBOSIS
	TMF_IVC
	TMF_HAEMORRHAGE
	TMF_BILIARY_TRACT_LEAK
	TMF_BILIARY_TRACT_STRICTURE
	TMF_BILIARY_STRICTURE_TREAT
	TMF_CMV_INFECTION
	TMF_FUNGAL_INFECTION
	TMF_SPUTUM_SEPSIS
	TMF_BLOOD_SEPSIS
	TMF_URINE_SEPSIS
	TMF_ASCITIES_SEPSIS
	TMF_WOUND_SEPSIS
	TMF_OTHER_SEPSIS
x	TMF_CYCLOSPORIN
x	TMF_AZATHIOPRINE
x	TMF_MYCOPHENOLATE
x	TMF_PREDNISOLONE

review only

x	TMF_FK506
x	TMF_IMMUNO_DRUG_OTHER
x	TMF_IMMUNO_DRUG_OTHER_TEXT
	AF1Y_LIFESTYLE
	AF1Y_DAYS_INPATIENT
	AF1Y_READMISSIONS
	AF1Y_PROTOCOL
	AF1Y_ACUTE_REJECTION
	AF1Y_CHRONIC_REJECTION
	AF1Y_BILIARY_COMP
	AF1Y_VASCULAR_COMP
	AF1Y_DISEASE_RECUR
	AF1Y_INFECTION
	AF1Y_READMIT_OTHER
	AF1Y_BILIRUBIN
	AF1Y_ALK_PHOSPHATE
	AF1Y_ALT_SGOT
	AF1Y_AST_SGPT
	AF1Y_BLOOD_UREA
	AF1Y_DYSFUNCTION
	AF1Y_TUMOUR
x	AF1Y_CYCLOSPORIN
x	AF1Y_FK506
x	AF1Y_AZATHIOPRINE
x	AF1Y_MYCOPHENOLATE
x	AF1Y_PREDNISOLONE
x	AF1Y_IMMUNO_DRUG_OTHER
x	AF1Y_IMMUNO_DRUG_OTHER_TEXT
	AF5Y_LIFESTYLE
	AF5Y_DAYS_INPATIENT
	AF5Y_READMISSIONS
	AF5Y_PROTOCOL
	AF5Y_ACUTE_REJECTION
	AF5Y_CHRONIC_REJECTION
	AF5Y_BILIARY_COMP
	AF5Y_VASCULAR_COMP
	AF5Y_DISEASE_RECUR
	AF5Y_INFECTION
	AF5Y_READMIT_OTHER
	AF5Y_BILIRUBIN
	AF5Y_ALK_PHOSPHATE
	AF5Y_ALT_SGOT
	AF5Y_AST_SGPT
	AF5Y_BLOOD_UREA
	AF5Y_DYSFUNCTION
	AF5Y_TUMOUR
	AF5Y_CYCLOSPORIN
	AF5Y_FK506
	AF5Y_AZATHIOPRINE
	AF5Y_MYCOPHENOLATE
	AF5Y_PREDNISOLONE
	AF5Y_IMMUNO_DRUG_OTHER
	AF5Y_IMMUNO_DRUG_OTHER_TEXT

For peer review only - http://bmjopen.bmj.com/site/about/guidelines.xhtml

X	COF
X	LOST_FUP
	laststat
	last_graft_no
	GSTATUS
	RCOD
	RCOD2
	tsurv
	tcens
X	gsurv
X	gcens
	PSURV
	PCENS

review only

For peer review only

**Liver**
**United Kingdom**
**Description**
Anonymised recipient identification number
Anonymised registration identification number
Letters of the first part of the recipients postcode
Recipient 2010 IMD deprivation score based on postcode (England)
Recipient 2010 IMD deprivation ranking order, 1 being the most deprived (England)
Recipient sex
Recipient age
Recipient age group (Adult or Paediatric)
Recipient blood group
Recipient weight at registration (kg)
Recipient height at registration (cm)
Recipient ethnicity
Primary indication for liver transplant
Secondary indication for liver transplant
Terriary indication for liver transplant
Liver failure grading
Lifestyle activity score prior to transplant
Inpatient prior to tranplant
Ventilated prior to transplant
Renal support prior to transplant
Ascites prior to transplant
Diuretic therapy prior to transplant
Encephalopathy grade prior to transplant
Previous abdominal surgery prior to transplant
ICP monitor prior to transplant
Oesophageal varices prior to transplant
Oesophageal varices prior to transplant indicate shunt
Pyrexial prior to transplant
Sepsis confirmed prior to transplant
If sepesis prior to transplant, where?
Hb prior to transplant (gm/dl)
White blood cells prior to transplant (x10^9/l)
Platelets prior to transplant (x10^9/l)
Recipient creatinine prior to transplant (μmol/l)
Recipient creatinine (μmol/l) prior to transplant after applying the Royal Free correction factor
Albumin prior to transplant (g/l)
Urea prior to transplant (mmol/l)
INR prior to transplant
Bilirubin prior to transplant (μmol/l)
Sodium prior to transplant (mmol/l)
Recipient UK End-stage Liver Disease score prior to transplant
Recipient Model for End-stage Liver Disease score prior to transplant
Potassium prior to transplant (mmol/l)
pH prior to transplant
Oxygen prior to transplant (Kpa)
Recipient CMV test result at registration
Most recent recipient HCV test result
Recipient HBV DNA test result at transplant
Recipient HBS AG test result at transplant

Recipient HBE AG test result at transplant
Recipient HCV RNA test result at transplant
Recipient Anti HBCLGM test result at transplant
Recipient Anti HBS test result at transplant
Recipient Anti HBE test result at transplant
Recipient Anti HCV test result at transplant
Recipient Anti DELTA test result at transplant
Recipient Anti CMV test result at transplant
Recipient Anti HSV test result at transplant
Recipient Anti HIV test result at transplant
Anonymised transplant identification number
Year of transplant
Re-registered after transplant indicator
Year of re-registration
Re-transplanted indicator
Year of next transplant
Type of transplant
Multi-organ transplant indicator
Transplant method
Lobe/segment of liver transplanted
Organ transplanted
Liver and small bowel combined transplant indicator
Urgency status at registration
Super-urgent indicator
Liver completeness of liver transplanted
Recipient transplant number (counts all solid organ transplants)
Number of liver transplants (counts liver transplants only)
Nation of transplant centre
Country of transplant centre
Anonymised donor identification number
Year of donation
Type of donor
Donor type group
Donor sex
Donor age
Donor age group (Adult or Paediatric)
Donor blood group
Donors BMI
Donor cause of death
Donor nation
Donor country
Donor CMV test result
Kidney retrieved
Pancreas retrieved
Small bowel retrieved
Heart retrieved
Lung retrieved
Donated more than one type of solid organ
Preservation fluid
Organ appearance
Steatosis
Capsular damage
Hepatic artery

Cold ischaemic time
Veno-venous bypass time
Operative repurfusion time
Biliary anastomosis
Hepatic artery anastomosis
Anti-fibrinolytic therapy used?
Intraoperative death?
Number of intraoperative units of blood
Number of intraoperative units of plasma
Number of intraoperative units of platelets
Number of intraoperative units of cryoprecipitate
Number of post-operative units of blood within 48 hours
Number of post-operative units of plasma within 48 hours
Number of post-operative units of platelets within 48 hours
Number of post-operative units of cryoprecipitate within 48 hours
Common bile duct
Common hepatic artery
Number of hepatic arteries
Iliac Artery
Iliac Vein
Left hepatic from left gastric and right hepatic from SMA
Left hepatic from left gastric
Lymph node
Main portal vein
Does the retrieved liver look normal
Aortic Patch
Right hepatic from SMA
Spleen
Vena Cava
Lifestyle activity score at 3 month post transplant
Number of treated rejection episodes since transplant at 3 month form
Number of days ventilated post-transplant
Number of days in ITU post-transplant
Renal status post-transplant
Complications hepatic artery thrombosis
Complications portal vein thrombosis
Complications IVC/hepatic vein occlusion
Complications haemorrhage requiring reoperation
Complications biliary tract leaks
Complications biliary tract stricture
Complications biliary tract stricture treatment
Post-operative CMV infection
Post-operative fungal infection
Post-operative sputum sepsis
Post-operative blood sepsis
Post-operative urine sepsis
Post-operative ascities sepsis
Post-operative wound sepsis
Post-operative other sepsis
Immunosuppression at 3 months: Cyclosporin A
Immunosuppression at 3 months: Azathioprine
Immunosuppression at 3 months: Mycophenolate mofetil
Immunosuppression at 3 months: Prednisolone

Immunosuppression at 3 months: FK 506
Immunosuppression at 3 months: Other
Immunosuppression at 3 months: Other text
Lifestyle activity score at 1 year post transplant
Total number of days as an inpatient during the last year (excluding transplant admission)
Number of readmissions over the last year
Number of readmissions if protocol was the primary reason for readmittance
Number of readmissions if acute rejection was the primary reason for readmittance
Number of readmissions if chronic rejection was the primary reason for readmittance
Number of readmissions if biliary complications was the primary reason for readmittance
Number of readmissions if vascular complications was the primary reason for readmittance
Number of readmissions if disease recurrence was the primary reason for readmittance
Number of readmissions if infection was the primary reason for readmittance
Number of readmissions if other was the primary reason for readmittance
Bilirubin at 1 year (μmol/l)
Alk phosphatase at 1 year (IU/L)
ALT/SGOT at 1 year (IU/L)
AST/SGPT at 1 year (IU/L)
Blood urea at 1 year (mmol/l)
Is renal dysfunction present at 1 year
Tumour been detected at 1 year
Immunosuppression at 1 year: Cyclosporin A
Immunosuppression at 1 year: FK 506
Immunosuppression at 1 year: Azathioprine
Immunosuppression at 1 year: Mycophenolate mofetil
Immunosuppression at 1 year: Prednisolone
Immunosuppression at 1 year: Other
Immunosuppression at 1 year: Other text
Lifestyle activity score at 5 years post transplant
Total number of days as an inpatient during the last year at 5 years
Number of readmissions over the last year at 5 years
Total number of readmissions if protocol was the primary reason for readmittance over the last year at 5 years
Total number of readmissions if acute rejection was the primary reason for readmittance over the last year at 5 y
Total number of readmissions if chronic rejection was the primary reason for readmittance over the last year at 5
Total number of readmissions if biliary complications was the primary reason for readmittance over the last year
Total number of readmissions if vascular complications was the primary reason for readmittance over the last ye
Total number of readmissions if disease recurrence was the primary reason for readmittance over the last year a
Total number of readmissions if infection was the primary reason for readmittance over the last year at 5 years
Total number of readmissions if other was the primary reason for readmittance over the last year at 5 years
Bilirubin at 5 years (μmol/l)
Alk phosphatase at 5 years (IU/L)
ALT/SGOT at 5 years (IU/L)
AST/SGPT at 5 years (IU/L)
Blood urea at 5 years (mmol/l)
Is renal dysfunction present at 5 years
Tumour been detected at 5 years
Immunosuppression at 5 years: Cyclosporin A
Immunosuppression at 5 years: FK 506
Immunosuppression at 5 years: Azathioprine
Immunosuppression at 5 years: Mycophenolate mofetil
Immunosuppression at 5 years: Prednisolone
Immunosuppression at 5 years: Other
Immunosuppression at 5 years: Other text

Cause of failure
Lost to follow up
Graft status of latest graft
Graft number of latest graft
Graft status
Primary recipient cause of death
Secondary recipient cause of death
Transplant survival time (days)
Transplant censoring indicator
Graft survival time (days)
Graft censoring indicator
Patient survival time (days)
Patient censoring indicator

For peer review only

Reference
see reference liver disease
see reference liver disease
see reference liver disease
1=Hyperacute, 2=Acute, 3=Subacute, 4=Not acute
1=Able to carry out normal activity without restriction, 2=Only restricted in physically strenuous activity, 3=Can m
1=No, 2=Yes, 8=Not reported, 9=Unknown
1=No, 2=Yes, 8=Not reported, 9=Unknown
1=Haemodialysis, 2=Filtration, 3=Not required
1=No, 2=Yes, 8=Not reported, 9=Unknown
1=No, 2=Yes, 8=Not reported, 9=Unknown
0=Not present, 1=Compromised. Altered mood/behaviour. Psychometric defects. 2=Drowsy. Inappropriate beha
1=No, 2=Yes, 8=Not reported, 9=Unknown
1=Normal pressure, 2=Raised pressure, 3=Not used
1=No previous variceal bleed, 2=Previous variceal bleed, 3=Not present
1=Portosystemic shunt, 2=Previous variceal bleed, 3=Not present
1=No, 2=Yes, 8=Not reported, 9=Unknown
1=No, 2=Yes, 8=Not reported, 9=Unknown
1=Sputum, 2=Blood, 3=Urine, 4=Ascities/Drain fluid, 5=Wound, 7=other
1=Negative, 2=Positive, 6=results awaited, 7=Not tested, 8=Not reported, 9=Unknown
1=Negative, 2=Positive, 6=results awaited, 7=Not tested, 8=Not reported, 9=Unknown
1=Negative, 2=Positive, 6=results awaited, 7=Not tested, 8=Not reported, 9=Unknown
1=Negative, 2=Positive, 6=results awaited, 7=Not tested, 8=Not reported, 9=Unknown

1=Negative, 2=Positive, 6=results awaited, 7=Not tested, 8=Not reported, 9=Unknown
1=Negative, 2=Positive, 6=results awaited, 7=Not tested, 8=Not reported, 9=Unknown
1=Negative, 2=Positive, 6=results awaited, 7=Not tested, 8=Not reported, 9=Unknown
1=Negative, 2=Positive, 6=results awaited, 7=Not tested, 8=Not reported, 9=Unknown
1=Negative, 2=Positive, 6=results awaited, 7=Not tested, 8=Not reported, 9=Unknown
1=Negative, 2=Positive, 6=results awaited, 7=Not tested, 8=Not reported, 9=Unknown
1=Negative, 2=Positive, 6=results awaited, 7=Not tested, 8=Not reported, 9=Unknown
1=Negative, 2=Positive, 6=results awaited, 7=Not tested, 8=Not reported, 9=Unknown
1=Negative, 2=Positive, 6=results awaited, 7=Not tested, 8=Not reported, 9=Unknown
1=Negative, 2=Positive, 6=results awaited, 7=Not tested, 8=Not reported, 9=Unknown

0=No, 1=Yes

0=No, 1=Yes

40=Liver, 80=IF liver-only, 83=IF liver/bowel/pancreas, 84=IF multivisceral, 110=Liver & kidney, 115=Liver, kidne
0=No, 1=Yes
1=Orthotopic, 2=Heterotopic
40=Whole liver, 41=Left liver lobe, 42=Right liver lobe, 46= Left lateral liver segment

0=No, 1=Yes
0=No, 1=Yes
Null=registered transplant, 91= No active record, 92=Transplanted whilst suspended, 93=Transplanted after tran
0=Whole, 1=Reduced, 2=Split

1=DBD, 2=DCD, 5=Living related, 6=Living, unrelated, 7=Domino 13=Living unrelated - Altruistic

see reference donor COD

1=Negative, 2=Positive, 6=results awaited, 7=Not tested, 8=Not reported, 9=Unknown
0=No, 1=Yes, 9=Unknown
0=No, 1=Yes, 9=Unknown
0=No, 1=Yes, 9=Unknown
0=No, 1=Yes, 9=Unknown
0=No, 1=Yes, 9=Unknown
0=No, 1=Yes, 9=Unknown
see reference perfusate used
1=Healthy, 2=Suboptimal
1=No, 2=Yes, 8=Not reported, 9=Unknown
1=No, 2=Yes, 8=Not reported, 9=Unknown
1=Single, 2=Accessory

1=No stent, 2=T-tube, 3=Roux, 4=Stent
1=Single 2=Multiple
1=No, 2=Yes, 8=Not reported, 9=Unknown
1=No, 2=Yes, 8=Not reported, 9=Unknown

1=No, 2=Yes, 8=Not reported, 9=Unknown
1=No, 2=Yes, 8=Not reported, 9=Unknown
1=No, 2=Yes, 8=Not reported, 9=Unknown
1=No, 2=Yes, 8=Not reported, 9=Unknown
1=No, 2=Yes, 8=Not reported, 9=Unknown
1=No, 2=Yes, 8=Not reported, 9=Unknown
1=No, 2=Yes, 8=Not reported, 9=Unknown
1=No, 2=Yes, 8=Not reported, 9=Unknown
1=No, 2=Yes, 8=Not reported, 9=Unknown
1=No, 2=Yes, 8=Not reported, 9=Unknown
1=No, 2=Yes, 8=Not reported, 9=Unknown
1=No, 2=Yes, 8=Not reported, 9=Unknown
1=No, 2=Yes, 8=Not reported, 9=Unknown
1=No, 2=Yes, 8=Not reported, 9=Unknown
1=Able to carry out normal activity without restriction, 2=Only restricted in physically strenuous activity, 3=Can m

1=No or minor renal impairment, 2=Required transient renal filtration, 3=Short-term dialysis, 4=Long-term dialysi
1=No, 2=Yes, 8=Not reported, 9=Unknown
1=No, 2=Yes, 8=Not reported, 9=Unknown
1=No, 2=Yes, 8=Not reported, 9=Unknown
1=No, 2=Yes, 8=Not reported, 9=Unknown
1=No leak, 2=Leak not treated, 3=Leak requiring reoperation, 4=Other radiological intervention
1=No, 2=Yes, 8=Not reported, 9=Unknown
1=Reoperated, 2=Balloon dilatation, 3=Stenting
1=No, 2=Yes, 8=Not reported, 9=Unknown
1=No, 2=Yes, 8=Not reported, 9=Unknown
1=No, 2=Yes, 8=Not reported, 9=Unknown
1=No, 2=Yes, 8=Not reported, 9=Unknown
1=No, 2=Yes, 8=Not reported, 9=Unknown
1=No, 2=Yes, 8=Not reported, 9=Unknown
1=No, 2=Yes, 8=Not reported, 9=Unknown
1=No, 2=Yes, 8=Not reported, 9=Unknown
1=No, 2=Yes, 8=Not reported, 9=Unknown
1=No, 2=Yes, 8=Not reported, 9=Unknown
1=No, 2=Yes, 8=Not reported, 9=Unknown
1=No, 2=Yes, 8=Not reported, 9=Unknown

1=No, 2=Yes, 8=Not reported, 9=Unknown
1=No, 2=Yes, 8=Not reported, 9=Unknown

1=Able to carry out normal activity without restriction, 2=Only restricted in physically strenuous activity, 3=Can m

1=No, 2=Yes, 8=Not reported, 9=Unknown
1=No, 2=Yes, 8=Not reported, 9=Unknown
1=No, 2=Yes, 8=Not reported, 9=Unknown
1=No, 2=Yes, 8=Not reported, 9=Unknown
1=No, 2=Yes, 8=Not reported, 9=Unknown
1=No, 2=Yes, 8=Not reported, 9=Unknown
1=No, 2=Yes, 8=Not reported, 9=Unknown
1=No, 2=Yes, 8=Not reported, 9=Unknown

1=Able to carry out normal activity without restriction, 2=Only restricted in physically strenuous activity, 3=Can m

1=No, 2=Yes, 8=Not reported, 9=Unknown
1=No, 2=Yes, 8=Not reported, 9=Unknown
1=No, 2=Yes, 8=Not reported, 9=Unknown
1=No, 2=Yes, 8=Not reported, 9=Unknown
1=No, 2=Yes, 8=Not reported, 9=Unknown
1=No, 2=Yes, 8=Not reported, 9=Unknown
1=No, 2=Yes, 8=Not reported, 9=Unknown
1=No, 2=Yes, 8=Not reported, 9=Unknown

see reference liver cause of failure
1=No, 2=Yes, 8=Not reported, 9=Unknown
1=Still alive, graft functioning, 2=Graft failed, 3=Death with a functioning graft, 4=Death only reported

1=Still alive, graft functioning, 2=Graft failed, 3=Death with a functioning graft, 4=Death only reported
see reference recipient COD
see reference recipient COD

0=Censored, 1=Event (Graft failure or death)

0=Censored, 1=Event (Graft failure)

0=Censored, 1=Event (Death of recipient)

For peer review only

care. Unable to do any form of work. 4=Only capable of limited self care. Confined mostly to bed or cl

aking and obeying simple commmands. Inarticulate speech. Marked confusion. 4= Coma. Cannot be

For peer review only

ung & liver, 140=Liver & pancreas, 160=Heart & liver, 190=Lung & liver

ter removal, 95=Transplanted after death reported

For peer review only

care. Unable to do any form of work. 4=Only capable of limited self care. Confined mostly to bed or cl

For peer review only

care. Unable to do any form of work. 4=Only capable of limited self care. Confined mostly to bed or cl

care. Unable to do any form of work. 4=Only capable of limited self care. Confined mostly to bed or cl

For peer review only

For peer review only

For peer review only

hair, 5=Completely reliant on nursing/medical care, 7=Aged five years or less

aroused

For peer review only

For peer review only

hair, 5=Completely reliant on nursing/medical care, 7=Aged five years or less

For peer review only

hair, 5=Completely reliant on nursing/medical care, 7=Aged five years or less

hair, 5=Completely reliant on nursing/medical care, 7=Aged five years or less

For peer review only

	**Activity/Organ(s):**
	**Geographical area:**
	**Mark data items required with an X**
**X**	**Variable**
	ATX_ID
	ARECIP_ID
	ADONOR_ID
X	rhosp
	NHS_GRP
X	RETHNIC
X	RSEX
X	rage
	rpaed
	RAGE_GRP2
X	rbg
X	rheight
X	rweight
X	RBMI
X	pcd
X	reg_primary_disease
	reg_prev_heart_surgery
	reg_prev_thoracotomy
	reg_prev_sudden_death
	reg_antiarrhythmics
	reg_aicd
	reg_hypertension
	reg_vascular_disease
	reg_cerebrovascular
	reg_cholesterol
	reg_diabetes
x	reg_prev_malignancy
	reg_prednisolone
x	reg_smoker
	reg_bilirubin
	reg_creatinine
	reg_home_oxygen
	reg_nyha_class
	reg_pa_systolic
	reg_pa_mean
	reg_pcw
	reg_cardiac_output
	reg_ejection_fraction
	reg_fev1
	reg_fvc
	reg_vo2_max
	reg_six_min_walk
	reg_in_hospital
	reg_ventilated
	reg_inotropes
	reg_iabp
	reg_vad
	reg_tah
	reg_ecmo

review only

X	CMV_TX
X	HCV_TX
X	HBV_TX
	HIV_TX
X	rwtime
X	uwtime
X	suwtime
X	tx_yr
	f_yr
	retunit
X	TX_TYPE
X	ORG_TXD
X	multi_tx
X	organ
X	tx
	het
X	GRAFT_NO
X	gno_hrt
X	gno_lng
X	ALLTX_NO
	urgent
X	dtype
	dgrp
	DD_YR
	dhosp
X	DCOD
X	dage
	dpaed
	dage_grp2
X	dsex
X	dheight
X	dweight
X	dethnic
X	DBMI
X	dbg
	ABOmatch
X	dcmv
	DHBSAG
	DHBCAB
X	DHCV
	DHIV
X	DEBV
	DHTLV
	DTOXO
	DSYPHILIS
	DPAST_ALCOHOL_ABUSE
	DPAST_CARDIO_DISEASE
	DPAST_DIABETES
	DPAST_FAMILY_DIABETES
	DPAST_FAMILY_DIABETES_TYPE
	DPAST_DRUG_ABUSE
	DPAST_HYPERTENSION
	DPAST_LIVER_DISEASE

review only

	DPAST_OTHER
x	DPAST_SMOKER
x	DPAST_SMOKER_AMOUNT
x	DPAST_TUMOUR
	DPAST_UTI
	DPAST_ALLERGY
	DHYPOTENSION
	DCARDIAC_ARREST
	DRESPIRATORY_ARREST
	HEART_PERFUSATE
	LUNG_PERFUSATE
	HEART_PERFUSION_METHOD
	LUNG_PERFUSION_METHOD
	FIRST_LUNG
	IT
	WIT
	PRE_OP_INFECTION
	NYHA
	IN_HOSP
	RVENT
	INOTROPES
	IABP
	ECMO
	VAD
	TAH
	RCREAT
	DAYS_TX_HDU
	DAYS_TX_ITU
	DAYS_TX_HOSP
	THEATRE
	HAEMOFILTRATION
	IABP_POST_OP
	OTHER_POST_OP
X	REJECTIONS
	INFECTIONS
	ORGANISMS
	infect1_TX
	infect2_TX
	infect3_TX
x	CYCLOSPORIN_TX
x	FK506_TX
x	AZATHIOPRINE_TX
x	CORTICOSTEROIDS_TX
x	SIROLIMUS_TX
x	ALG_induction_TX
x	ALG_rejection_TX
x	OKT3_induction_TX
x	OKT3_rejection_TX
	TLI_TX
	METHOTREXATE_TX
	IMMUNO_DRUG_OTHER_TX
	IMMUNO_TEXT_TX
	RCOD

r review only

X	gstatus
X	cof
X	LOST_FUP
	vtl_stat
	re_tx
	tsurv
	tcens
X	gsurv
X	gcens
	psurv
	pcens
	HOSPITAL_ADMISSIONS_j
	REJECTION_EPISODES_j
	AIRWAY_COMPLICATIONS_j
	INFECTIONS_j
	FEV1_1_j
	FEV1_2_j
	NYHA_CLASS_j
	ORGANISM_TEXT_j
	INFECT_1_j
	INFECT_2_j
	INFECT_3_j
	CREATININE_j

r review only

**Cardiothoracic:**
**United Kingdom**
**Description**
Anonymised transplant identification number
Anonymised recipient identification number
Anonymised donor identification number
Anonymised unit that received and transplanted the organ
Recipient NHS group
Recipient ethnicity
Recipient sex
Recipient age
Recipient paediatric (<16) or adult
Recipient age in 10 year increments
Recipient blood group
Height of recipient (cm)
Weight of recipient (kg)
Recipient BMI
Primary cause of cardiothoracic disease
Primary disease at registration
Number of previous open heart surgery operations at registration
Previous thoracotomy at registration
Previous sudden death episode at registration
Antiarrhythmics (excluding digoxin) at registration
AICD at registration
Hypertension requring treatment in the last 5 years at registration
Peripheral vascular disease with intervention performed or planned at registration
Cerebrovascular disease at registration
Cholesterol (mmol/l) at registration
Diabetes at registration
Previous malignancy at registration
Daily dose of prednisolone (mg) at registration
Smoker more than 5 a day within 6 months at registration
Bilirubin (μmol/l) at registration
Creatinine (μmol/l) at registration
Home oxygen
NYHA class at registration
PA systolic (mm Hg) at registration
PA mean (mm Hg) at registration
PCW or LAP (mm Hg) at registration
Cardiac output (l/min) at registration
Ejection fraction (%) at registration
FEV1 (litres) at registration
FVC (litres) at registration
VO2 max (ml/kg/min) at registration
6 minute walk test (m) at registration
In hospital at registration
Patient on ventilator whilst in hopsital at registration
Patient on intopes whilst in hopsital at registration
IABP whilst in hospital at registration
VAD whilst in hospital at registration
TAH whilst in hospital at registration
ECMO whilst in hospital at registration

Recipient CMV status at transplant
Recipient HCV status at transplant
Recipient HBV status at transplant
Recipient HIV status at transplant
Routine wait time (days)
Urgent wait time (days)
Super-urgent wait time (days)
Year of transplant
Financial year of transplant
Anonymised unit that retrieved the organ
Type of transplant
Type of cardiothoracic transplant
Multi-organ transplant indicator
Organ transplanted
Indicator organ transplanted
Heart transplanted was heterotopic or orthotopic
Number of Heart/lung transplants
Number of heart transplants
Number of lung transplants
Number of all transplants
Urgency status of transplant
Donor type
Donor type group
Year of donation
Anonymised donation centre
Donor cause of death
Donor age
Donor paediatric (<16) or adult
Donor age in 10 year increments
Donor sex
Donor height (cm)
Donor weight (kg)
Donor ethnicity
Donor BMI
Donor blood group
Donor vs recipient blood group
Donor CMV test result
Donor HBSAG test result
Donor HBCAB test result
Donor HCV test result
Donor HIV test result
Donor EBV test result
Donor HTLV test result
Donor Toxo test result
Donor syphilis test result
Donor history of alcohol abuse
Donor cardio disease
Donor diabetes
Donors family history of diabetes
Donors family history type of diabete
Donor history of drug abuse
Donor hypertension
Donor liver disease

Donor past other
Donor smoker
Donor smoker amount (number smoked per day)
Donor tumour
Donor UTI
Donor allergy
Donor hypotension (less than 70mmHg systolic)
Donor cardiac arrest
Donor respiratory arrest
Heart perfusion fluid
Lung perfusion fluid
Heart perfusion method
Lung perfusion method
First lung perfused
Total ischaemic time hours from cross clamp of donor to reperfusion time (hours)
Warm ischemia time (minutes)
Infection requiring antibiotics in the last 6 weeks at transplant
NYHA class at transplant
In hospital at transplant
Patient on ventilator whilst in hospital at transplant
Patient on intopes whilst in hospital at transplant
IABP whilst in hospital at transplant
ECMO whilst in hospital at transplant
VAD whilst in hospital at transplant
TAH whilst in hospital at transplant
Creatinine (μmol/l) at transplant
Number of days spent in HDU after transplant
Number of days spent in ITU after transplant
Number of days spent in hospital after transplant
Complications - return to theatre
Complications - haemofiltration/haemodialysis
Complications - IABP post-op
Complications - other mechanical assistance post-op
Number of treated rejection episodes at transplant
Number of serious infection episodes at transplant
If infection at transplant what was the organism
Primary site of infection at transplant
Secondary site of infection at transplant
Teritary site of infection at transplant
Cyclosporin immunosuppression at transplant
FK506 immunosuppression at transplant
Azathioprine immunosuppression at transplant
Corticosteroids immunosuppression at transplant
Sirolimus immunosuppression at transplant
ALG/ATG antibody inducation at transplant
ALG/ATG antibody for rejection at transplant
OKT3 antibody inducation at transplant
OKT3 antibody for rejection at transplant
TLI immunosuppression at transplant
Methotrexate immunosuppression at transplant
Other immunosuppression at transplant
Other immunosuppression text at transplant
Recipient cause of death

Most recent outcome of graft
Cause of failure
Lost to follow up
Vital status of recipient
Whether patient was re-transplanted
Transplant survival time (days)
Transplant censoring indicator
Graft survival time (days)
Graft censoring indicator
Patient survival time (days)
Patient censoring indicator
Number of hospital admissions in follow-up period j (j=3, 12, 24, 36, … corresponding to months post-transplant)
Number of rejection episodes in follow-up period j (j=3, 12, 24, 36, … corresponding to months post-transplant)
Airway complications in follow-up period j (j=3, 12, 24, 36, … corresponding to months post-transplant)
Number of serious infection episodes in follow-up period j (j=3, 12, 24, 36, … corresponding to months post-trans
First of two best FEV litres at follow up period j (j=3, 12, 24, 36, … corresponding to months post-transplant)
Second of two best FEV litres follow up period j (j=3, 12, 24, 36, … corresponding to months post-transplant)
NYHA Class at follow up period j (j=3, 12, 24, 36, … corresponding to months post-transplant)
If infection in follow-up period j (j=3, 12, 24, 36, … corresponding to months post-transplant), what was the orgar
Primary site of infection in follow-up period j (j=3, 12, 24, 36, … corresponding to months post-transplant)
Secondary site of infection in follow-up period j (j=3, 12, 24, 36, … corresponding to months post-transplant)
Teritary site of infection in follow-up period j (j=3, 12, 24, 36, … corresponding to months post-transplant)
Creatinine (µmol/l) at follow up period j (j=3, 12, 24, 36, … corresponding to months post-transplant)

eview only

Reference

1=Group 1, 2=Group 2, 8=Not reported, 9=Unknown

NR=Not reported, UKN=Unknown

888=Not reported
888.8=Not reported

see reference CARDIO PRIMARY DISEASE
see reference CARDIO PRIMARY DISEASE
88=Not reported, 99=Unknown
1=No, 2=Yes, 8=Not reported 9=Unknown
1=No, 2=Yes, 8=Not reported 9=Unknown
1=No, 2=Yes, 8=Not reported 9=Unknown
1=No, 2=Yes, 8=Not reported 9=Unknown
1=No, 2=Yes, 8=Not reported 9=Unknown
1=No, 2=Yes, 8=Not reported 9=Unknown
1=No, 2=Yes, permanent disability, 3=Yes, transient disability, 8=Not reported 9=Unknown
88.8=Not reported, 99.9=Unknown
1=No, 2=Yes, insulin dependent, 3=Yes, not insulin dependent, 8=Not reported, 9=Unknown
1=No, 2=Yes, 8=Not reported 9=Unknown
888.8=Not reported, 999.9=Unknown
1=No, 2=Yes, 8=Not reported 9=Unknown
8888=Not reported, 9999=Uknown
8888=Not reported, 9999=Uknown
1=No, 2=Yes, 8=Not reported 9=Unknown
1=I, 2=II, 3=III, 4=IV, 8=Not reported, 9=Unknown
888=Not reported, 999=Unknown
888=Not reported, 999=Unknown
88=Not reported, 99=Unknown
88.8=Not reported, 99.9=Unknown
88=Not reported, 99=Unknown
88.88=Not reported, 9.99=Unknown
88.88=Not reported, 9.99=Unknown
88=Not reported, 99=Unknown
888=Not reported, 999=Unknown
1=No, 2=Yes, 8=Not reported 9=Unknown
1=No, 2=Yes, 8=Not reported 9=Unknown
1=No, 2=Yes, 8=Not reported 9=Unknown
1=No, 2=Yes, 8=Not reported 9=Unknown
1=None, 2=Left, 3=Right, 4=Both 8=Not reported, 9=Unknown
1=No, 2=Yes, 8=Not reported 9=Unknown
1=No, 2=Yes, 8=Not reported 9=Unknown

1=Negative, 2=Positive, 3=Indeterminate, 4=Repeat reactive, 5=Confirmed positive, 6=results awaited, 7=Not te
1=Negative, 2=Positive, 3=Indeterminate, 4=Repeat reactive, 5=Confirmed positive, 6=results awaited, 7=Not te
1=Negative, 2=Positive, 3=Indeterminate, 4=Repeat reactive, 5=Confirmed positive, 6=results awaited, 7=Not te
1=Negative, 2=Positive, 3=Indeterminate, 4=Repeat reactive, 5=Confirmed positive, 6=results awaited, 7=Not te

30=Heart, 60=Lung, 63=Double lung, 65=Bilateral single lung, 66=Partial lung, 70=Heart/lung, 120=Heart, lung &
35=Orthotropic heart, 36=Heterotopic heart, 61=Left lung, 62=Right lung, 63=Double lung, 65=Bilateral single lu
0=Single organ transplant, 1=Multi-organ transplant
30=Heart, 61=Left lung, 62=Right lung, 63=Lung pair
H=Heart, L=Lungs, HL=Heart/Lungs
0=Orthotopic, 1=Heterotopic

0=Routine/Non-urgent, 1=Urgent, 2=Super-urgent
1=DBD, 2=DCD, 5=Living related, 6=Living, unrelated, 7=Domino 13=Living unrelated - Altruistic

see reference DONOR COD

NR=Not reported, UKN=Unknown
888=Not reported, 999=Unknown
888=Not reported, 999=Unknown

1=Identical, 2=Compatible, 3=Incompatible
1=Negative, 2=Positive, 3=Indeterminate, 4=Repeat reactive, 5=Confirmed positive, 6=results awaited, 7=Not te
1=Negative, 2=Positive, 3=Indeterminate, 4=Repeat reactive, 5=Confirmed positive, 6=results awaited, 7=Not te
1=Negative, 2=Positive, 3=Indeterminate, 4=Repeat reactive, 5=Confirmed positive, 6=results awaited, 7=Not te
1=Negative, 2=Positive, 3=Indeterminate, 4=Repeat reactive, 5=Confirmed positive, 6=results awaited, 7=Not te
1=Negative, 2=Positive, 3=Indeterminate, 4=Repeat reactive, 5=Confirmed positive, 6=results awaited, 7=Not te
1=Negative, 2=Positive, 3=Indeterminate, 4=Repeat reactive, 5=Confirmed positive, 6=results awaited, 7=Not te
1=Negative, 2=Positive, 3=Indeterminate, 4=Repeat reactive, 5=Confirmed positive, 6=results awaited, 7=Not te
1=Negative, 2=Positive, 3=Indeterminate, 4=Repeat reactive, 5=Confirmed positive, 6=results awaited, 7=Not te
1=Negative, 2=Positive, 3=Indeterminate, 4=Repeat reactive, 5=Confirmed positive, 6=results awaited, 7=Not te
1=No, 2=Yes, 8=Not reported, 9=Unknown
1=No, 2=Yes, 8=Not reported, 9=Unknown
1=No, 2=Yes, 8=Not reported, 9=Unknown
1=No, 2=Yes, 8=Not reported, 9=Unknown
3=Type 1, 4=Type 2, 5=Type 1 & 2, 8=Not reported, 9=Unknown
1=No, 2=Yes, 8=Not reported, 9=Unknown
1=No, 2=Yes, 8=Not reported, 9=Unknown
1=No, 2=Yes, 8=Not reported, 9=Unknown

1=No, 2=Yes, 8=Not reported, 9=Unknown
1=No, 2=Yes, 8=Not reported, 9=Unknown
99=Unknown
1=No, 2=Yes, 8=Not reported, 9=Unknown
1=No, 2=Yes, 8=Not reported, 9=Unknown
1=No, 2=Yes, 8=Not reported, 9=Unknown
1=No, 2=Yes, 8=Not reported 9=Unknown
1=No, 2=Yes, 8=Not reported 9=Unknown
1=No, 2=Yes, 8=Not reported 9=Unknown
see reference PERFUSATE USED
see reference PERFUSATE USED
1=Flush, 2=Core-cooling, 3=Immersion, 6=Not perfused/not applicable, 7=Other, 8=Missing, 9=Unknown
1=Flush, 2=Core-cooling, 3=Immersion, 6=Not perfused/not applicable, 7=Other, 8=Missing, 9=Unknown
61=Left, 62=Right

1=No, 2=Yes, 8=Not reported 9=Unknown
1=I, 2=II, 3=III, 4=IV, 8=Not reported, 9=Unknown
1=No, 2=Yes, 8=Not reported 9=Unknown
1=No, 2=Yes, 8=Not reported 9=Unknown
1=No, 2=Yes, 8=Not reported 9=Unknown
1=No, 2=Yes, 8=Not reported 9=Unknown
1=No, 2=Yes, 8=Not reported 9=Unknown
1=None, 2=Left, 3=Right, 4=Both, 8=Not reported, 9=Unknown
1=No, 2=Yes, 8=Not reported, 9=Unknown
8888=Not reported, 9999=Uknown

1=No, 2=Yes, 8=Not reported 9=Unknown
1=No, 2=Yes, 8=Not reported 9=Unknown
1=No, 2=Yes, 8=Not reported 9=Unknown
1=No, 2=Yes, 8=Not reported 9=Unknown
88=Not reported, 99=Unknown
88=Not reported, 99=Unknown

1=No, 2=Yes, 8=Not reported, 9=Unknown
1=No, 2=Yes, 8=Not reported, 9=Unknown
1=No, 2=Yes, 8=Not reported, 9=Unknown
1=No, 2=Yes, 8=Not reported, 9=Unknown
1=No, 2=Yes, 8=Not reported, 9=Unknown
1=No, 2=Yes, 8=Not reported, 9=Unknown
1=No, 2=Yes, 8=Not reported, 9=Unknown
1=No, 2=Yes, 8=Not reported, 9=Unknown
1=No, 2=Yes, 8=Not reported, 9=Unknown
1=No, 2=Yes, 8=Not reported, 9=Unknown
1=No, 2=Yes, 8=Not reported, 9=Unknown
1=No, 2=Yes, 8=Not reported, 9=Unknown

see reference RECIPIENT COD

1=Functioning, 2=Failed, 3=Died with functioning graft, 4=Death only reported, 8=Not reported, 9=Unknown
see reference CARDIOTHORACIC CAUSES OF FAILURE
1=No, 2=Yes, 8=Not reported, 9=Unknown
1=Alive, 2=Dead, 8=Missing
0=No, 1=Yes
-100=Not reported
0=Censored, 1=Event (Graft failure or death)
-100=Not reported
0=Censored, 1=Event (Graft failure)
-100=Not reported, Will be missing for regrafts
0=Censored, 1=Event (Death of recipient)

1=No, 2=Yes, 8=Not reported, 9=Unknown

9.99=Unknown
9.99=Unknown
1=I, 2=II, 3=III, 4=IV, 8=Not reported, 9=Unknown

eview only

For peer review only

ey & liver, 180=Heart, lung & kidney, 190=Lung & liver

For peer review only

	Activity/Organ(s):
	Geographical area:
	Mark data items required with an X
X	Variable
	ATX_ID
	ADONOR_ID
	ARECIP_ID
	DTYPE
	SOLID
	DAGE
	DSEX
	DCOD
	RAGE
	RSEX
	TX_YR
	GTYPE
	DIAG
	GNO_OPERATED
	GNO_FELLOW
	LOGMAR0
	LOGMAR1
	GSURV
	GCENS

For peer review only

Ocular tissue transplant dataset
United Kingdom
**Description**
Anonymised transplant identification number
Anonymised donor identification number
Anonymised recipient identification number
Donor type
Solid organs donated
Donor age
Donor sex
Donor cause of death
Recipient age
Recipient sex
Calendar year of transplant
Graft type
Indication for ocular tissue transplant
Graft number in operated eye
Number of previous grafts in fellow eye
LogMAR acuity at transplant
LogMAR acuity at one-year (8-16 months post-transplant)
Graft survival (days)
Graft censor

For peer review only

**Reference**
1=DBD, 2=DCD, 10=Tissue only
0=No, 1=Yes
1=Male, 2=Female, 8=Not reported, 9=Unknown
see reference donor COD
1=Male, 2=Female, 9=Unknown
1=Penetrating keratoplasty (PK), 2=Superficial lamellar, 3=Deep anterior lamellar keratoplasty (DALK), 5=sclera/cornea in ethanol, 6=stem cell allograft, 7=Other, 10=Descemet's membrane endothelial keratoplasty
see Recipient Diagnosis
0=Censored, 1=Event (graft failure)

For peer review only

	**Activity/Organ(s):**
	**Time period:**
	**Geographical area:**
	**Mark data items required with an X**
**X**	**Variable**
	ATX_ID
	ADONOR_ID
	ARECIP_ID
	DTYPE
	DAGE_GRP
	DSEX
	DCOD_GRP
	RAGE_GRP
	RSEX
	TX_YR
	TX_TYPE
	AMM_1998
	BMM_1998
	DRMM_1998
	KID_PD_GRP
	KID_CIT_MINS
	KID_GSURV
	KID_GCENS
	KID_PSURV
	KID_PCENS
	KID_TSURV
	KID_TCENS
	PAN_PD_GRP
	PAN_CIT_MINS
	ISLET_URGENCY_STATUS
	PAN_GSURV
	PAN_GCENS
	PAN_PSURV
	PAN_PCENS
	PAN_TSURV
	PAN_TCENS
	LIV_PD_GRP
	LIV_CIT_MINS
	LIV_URGENCY_STATUS
	LIV_GSURV
	LIV_GCENS
	LIV_PSURV
	LIV_PCENS
	LIV_TSURV
	LIV_TCENS
	CARDIO_PD_GRP
	CARDIO_IT_MINS
	CARDIO_URGENCY_STATUS
	LNG_GSURV
	LNG_GCENS
	LNG_PSURV
	LNG_PCENS
	LNG_TSURV

For peer review only

LNG_TCENS
HRT_GSURV
HRT_GCENS
HRT_PSURV
HRT_PCENS
HRT_TSURV
HRT_TCENS

For peer review only

Public all organ transplant dataset
1 January 1995 - 31 December 2016
United Kingdom
**Description**
Anonymised transplant identification number
Anonymised Donor identification number
Anonymised Recipient identification number
Donor type
Donor age at donation
Sex of donor
Donor cause of death
Recipient age at transplant
Sex of recipient
Calendar year of transplant
Transplant type
Number of mismatches at A locus
Number of mismatches at B locus
Number of mismatches at DR locus
Primary renal disease group
Kidney cold ischaemia time (mins)
Graft survival time (days) for kidney transplant
Graft survival censoring indicator for kidney transplant
Patient survival time (days) for kidney transplant
Patient survival censoring indicator for kidney transplant
Transplant survival time (days) for kidney transplant
Transplant survival censoring indicator for kidney transplant
Primary renal disease group
Pancreas cold ischaemia time (mins)
Islet urgency status of recipient
Graft survival time (days) for pancreas transplant
Graft survival censoring indicator for pancreas transplant
Patient survival time (days) for pancreas transplant
Patient survival censoring indicator for pancreas transplant
Transplant survival time (days) for pancreas transplant
Transplant survival censoring indicator for pancreas transplant
Primary liver disease group
Liver cold ischaemia time (mins)
Urgency status of recipient
Graft survival time (days) for liver transplant
Graft survival censoring indicator for liver transplant
Patient survival time (days) for liver transplant
Patient survival censoring indicator for liver transplant
Transplant survival time (days) for liver transplant
Transplant survival censoring indicator for liver transplant
Primary cardio disease group
Cardio ischaemia time (mins)
Urgency status of recipient
Graft survival time (days) for lung transplant
Graft survival censoring indicator for lung transplant
Patient survival time (days) for lung transplant
Patient survival censoring indicator for lung transplant
Transplant survival time (days) for lung transplant

For peer review only

Transplant survival censoring indicator for lung transplant
Graft survival time (days) for heart transplant
Graft survival censoring indicator for heart transplant
Patient survival time (days) for heart transplant
Patient survival censoring indicator for heart transplant
Transplant survival time (days) for heart transplant
Transplant survival censoring indicator for heart transplant

For peer review only

Reference

See donor type in Appendix 1
See donor age group in Appendix 2
1=Male, 2=Female, 8=Not reported, 9=Unknown
See donor cause of death codes in Appendix 3
See recipient age group in Appendix 2
1=Male, 2=Female, 8=Not reported, 9=Unknown

See transplant type in Appendix 5
0=zero mismatches, 1=1 mismatch, 2=2 mismatches, 7=Not tested, 9=Unknown
0=zero mismatches, 1=1 mismatch, 2=2 mismatches, 7=Not tested, 9=Unknown
0=zero mismatches, 1=1 mismatch, 2=2 mismatches, 7=Not tested, 9=Unknown
See Primary renal disease code in Appendix 4

0=Censored, 1=Event (graft failure)

0=Censored, 1=Event (death of patient)

0=Censored, 1=Event (graft failure or death of patient)
See Primary renal disease code in Appendix 4

0=Routine, 1=Priority

0=Censored, 1=Event (graft failure)

0=Censored, 1=Event (death of patient)

0=Censored, 1=Event (graft failure or death of patient)
See Primary liver disease code in Appendix 4

0=Elective, 1=Super Urgent

0=Censored, 1=Event (graft failure)

0=Censored, 1=Event (death of patient)

0=Censored, 1=Event (graft failure or death of patient)
See Primary cardiothoracic disease code in Appendix 4

0=Elective, 1=Urgent

0=Censored, 1=Event (graft failure)

0=Censored, 1=Event (death of patient)

For peer review only

0=Censored, 1=Event (graft failure or death of patient)

0=Censored, 1=Event (graft failure)

0=Censored, 1=Event (death of patient)

0=Censored, 1=Event (graft failure or death of patient)

For peer review only

References

DONOR COD

0 Living donor
10 Intracranial haemorrhage
11 Intracranial thrombosis
12 Brain tumour
13 Hypoxic brain damage - all causes
19 Intracranial - type unclassified (CVA)
20 Trauma RTA - car
21 Trauma RTA - motorbike
22 Trauma RTA - pushbike
23 Trauma RTA - pedestrian
24 Trauma RTA - Other
29 Trauma RTA - unknown type
30 Other trauma - known or suspected suicide
31 Other trauma - accident
39 Other trauma - unknown cause
40 Cardiac arrest
41 Myocardial infarction
42 Aneurysm
42 Aortic Aneurysm
43 Ischaemic heart disease
44 Congestive heart failure
45 Pulmonary embolism
49 Cardiovascular - type unclassified
50 Chronic pulmonary disease
51 Pneumonia
52 Asthma
53 Respiratory failure
54 Carbon monoxide poisoning
59 Respiratory - type unclassified (inc smoke inhalation)
60 Cancer (other than brain tumour)
70 Meningitis
71 Septicaemia
72 Infections - type unclassified
73 Acute blood loss/hypovolaemia
74 Liver failure (not self poisoning)
75 Renal failure
76 Multi-organ failure
77 Sudden Infant Death Syndrome (SIDS)
78 Burns
80 Alcohol poisoning
81 Paracetamol overdose
82 Other drug overdose, please specify
85 Self poisoning - type unclassified
88 Not reported
90 OTHER
98 Other, please specify
99 Unknown

RECIPIENT COD

500 Cause of death uncertain/not determined
511 Myocardial ischaemia and infarction

512 Hyperkalaemia
513 Haemorrhagic pericarditis
514 Other causes of cardiac failure
515 Sudden unexplained cardiac death
516 Hypertensive cardiac failure
517 Hypokalaemia
518 Fluid overload
519 Elevated pvr/pulmonary hypertension
520 Airway dehiscence
521 Pulmonary embolus
522 Cerebro-vascular accident
523 Gastro-intestinal haemorrhage
524 Haemorrhage from graft site
525 Haemorrhage from vascular access or dialysis circuit
526 Haemorrhage from ruptured vascular aneurysm (not code 22 or 23)
527 Haemorrhage from surgery (not code 23,24 or 26)
528 Other haemorrhage (not codes 23-27)
529 Mesenteric infarction
530 Pulmonary infection (protozoal)
531 Pulmonary infection (bacterial)
532 Pulmonary infection (viral)
533 Pulmonary infection (fungal)
534 Infections elsewhere (except viral hepatitis)
535 Septicaemia
536 Tuberculosis (lung)
537 Tuberculosis (elsewhere)
538 Generalized viral infection
539 Peritonitis (not code 70)
541 Liver - due to hepatitis b virus
542 Liver - other viral hepatitis
543 Liver - drug toxicity
544 Cirrhosis - not viral
545 Cystic liver disease
546 Liver failure - cause unknown
547 Renal failure
548 Recurrent primary disease - benign
549 Recurrent primary disease - malignant
551 Patient refused further treatment
552 Suicide
553 Therapy ceased for any other reason
554 Esrf treatment withdrawn for medical reasons
561 Uraemia caused by graft failure
562 Pancreatitis
563 Bone marrow depression
564 Cachexia
566 Lymphoid malignant disease possibly induced by immunosuppressive therapy
567 Lymphoid malignant disease not induced by immunosuppressive therapy
568 Malignant disease: lymphoproliferative disorders (except 66)
569 Dementia
570 Sclerosing (or adhesive) peritoneal disease
571 Perforation of peptic ulcer
572 Perforation of colon
573 Non-lymphoid malignant disease possibly induced by immunosuppressive therapy

574 Non-lymphoid malignant disease not induced by immunosuppressive therapy
575 Early graft dysfunction
576 Cardiac tamponade
577 Ards
578 Respiratory failure
579 Multi-system failure
581 Accident related to treatment
582 Accident unrelated to treatment
590 Donor organ failure
595 Other identified cause of death
598 Other identified cause of death
599 Unknown
600 Peritonitis (bacterial, with peritoneal dialysis
601 Peritonitis (fungal, with peritoneal dialysis
602 Peritonitis (due to other cause, with peritoneal dialysis
888 Cod not requested

Table A HLA mismatch groups

Level	HLA mismatch summary
1	0
2	[0 DR and 0/1 B]
3	[0 DR and 2 B] or [1 DR and 0/1 B]
4	[1 DR and 2 B] or [2 DR]

PANCREAS/RENAL DISEASE

200 Chronic renal failure, aetiology uncertain
210 Glomerulonephritis, histologically not examined
211 Severe nephrotic syndrome with focal sclerosis
212 IgA nephropathy
213 Dense deposit disease
214 Membranous nephropathy
215 Membrano - proliferative glomerulonephritis
216 Rapidly progressive GN without systemic disease
217 Focal segmental glomerulosclerosis with nephrotic syndrome in adults
219 Glomerulonephritis, histologically examined
220 Pyelonephritis/Interstitial nephritis - cause not specified
221 Pyelonephritis/Interstitial nephritis associated with neurogenic bladder
222 Pyelonephritis/Interstitial nephritis due to con obs uropathy with/without V-U reflux
223 Pyelonephritis/Interstitial nephritis due to acquired obstructive uropathy
224 Pyelonephritis/Interstitial nephritis due to V-U reflux without obstruction
225 Pyelonephritis/Interstitial nephritis due to urolithiasis
229 Pyelonephritis/Interstitial nephritis due to other cause
230 Tubulo Interstitial Nephritis (Not Pyelonephritis)
231 Nephropathy due to analgesic drugs
232 Nephropathy due to cis-platinum
233 Nephropathy due to cyclosporin A
239 Nephropathy caused by other specific drug
240 Cystic kidney disease - type unspecified

241 Polycystic kidneys, adult type (dominant type)
242 Polycystic kidneys, infantile (recessive type)
243 Medullary cystic disease, including nephronophthisis
249 Cystic kidney disease - other specified type
250 Hereditary/Familial nephropathy - type unspecified
251 Hereditary nephritis with nerve deafness (Alports syndrome)
252 Cystinosis
253 Primary oxalosis
254 "Fabrys disease"
259 Hereditary nephropathy - other
260 Congenital renal hypoplasia - type unspecified
261 Oligomeganephronic hypoplasia
262 Segmental renal hypoplasia
263 Congenital renal dysplasia with or without urinary tract malformation
266 Syndrome of agenesis of abdominal muscles
270 Renal vascular disease - type unspecified
271 Renal vascular disease - malignant hypertension
272 Renal vascular disease - hypertension
273 Renal vascular disease - polyarteritis
274 "Wegeners granulomatosis"
275 Ischaemic renal disease/ cholesterol embolism
279 Renal vascular disease - classified
280 Diabetes - insulin dependent (Type I)
281 Diabetes - non-insulin dependent (Type II)
282 Myelomatosis/Light chain deposit disease
283 Amyloid
284 Lupus erythematosus
285 Henoch-Schonlein purpura
286 "Goodpastures Syndrome"
287 Systemic sclerosis (Scleroderma)
288 Haemolytic Uraemic Syndrome (inc Moschowitz Syndrome)
289 Multi-system disease - other
290 Cortical or tubular necrosis
291 Tuberculosis
292 Gout
293 Nephrocalcinosis & hypercalcaemic nephropathy
294 Balkan nephropathy
295 Kidney tumour
296 Traumatic or surgical loss of kidney
298 Other identified renal disorders
299 Unknown
888 Not reported

PANCREAS CAUSE OF FAILURE

0 Graft still functioning
201 Vascular thrombosis
202 Infection
203 Bleeding
204 Anastomotic leak
205 Pancreatitis
206 Primary non-function (no detectable C-peptide between 7 and 28 days)
207 Patient declined medication
208 Hyperacute rejection

209 Acute rejection
210 Chronic rejection
211 Preservation/Procurement problem
212 Instant blood mediated inflammatory reaction (IBMIR)
213 Poor engraftment
214 Functional decline (unknown cause)
215 Recurrent autoimmunity
216 Hepatitis / Steatosis
217 Cholangitis
290 Death with a functioning graft
298 Other
299 Unknown

RELATIONSHIP

0 Son or daughter
1 Cadaveric donor, no relationship
2 Mother or father
3 Sibling (2 shared haplotypes)
4 Sibling (1 shared haplotype)
5 Sibling (0 shared haplotype)
6 Sibling (genetic relationship unknown)
7 Monozygotic (identical) twin
8 Dizygotic (non-identical) twin
9 Other living related - please specify
10 Obligatory removal of organ
11 Living non-related donor - spouse
12 Living non-related donor - partner
15 Pooled donor
16 Altruistic non-directed donor
19 Other living non-related donor - please specify
20 Living donor, relationship unspecified
88 Missing
99 Unknown
103 Default relationship - update required

PERFUSATE USED

0 Not known
1 Sacks
2 Perfudex
3 Dextran 40
4 Gelin
6 Hartmans
7 Manitol
8 Rheomacrodex
10 Euro collins
20 Wisconson/u (UW solution)
21 U.W.(commercial)
22 Modified U.W.(high Na+)
23 Modified U.W.(high k+)
27 Other liver preservation fluid
28 Not reported
29 Unknown

30 Marshalls/hoc
40 PBS
50 St thomas
60 Papworth solution
66 Not perfused/not applicable
70 Machine preservation - PPF
79 Machine preservation - other
88 Not reported
95 Other
98 Other
99 Unknown

RENAL CAUSES OF FAILURE

0 Graft still functioning
201 Hyperacute rejection
202 Rejection while taking immunosuppressive drug(s)
203 Rejection after stopping all immunosuppressive drugs
204 Recurrent primary renal disease
205 Vascular or ureteric operative problems (excluding vascular thrombosis)
206 Vascular (arterial or venous) thrombosis
207 Infection of graft
208 Removal of functioning graft
209 Non-viable kidney
290 Recipient died, graft was functioning at time of death
295,298 Other
299 Unknown

CARDIO PRIMARY DISEASE

310 Coronary Heart Disease
311 Dilated Cardiomyopathy - type unspecified
313 Valvular Heart Disease
314 Congenital Heart Disease
319 Other Heart Disease
320 Primary Pulmonary Hypertension
321 Eisenmenger's Syndrome - type unspecified
322 Cystic Fibrosis
323 Fibrosing Lung Disease
324 Inhalation
325 Alpha-1-Antitrypsin deficiency
326 Sarcoid
327 Emphysema
328 Bronchiectasis
330 Immediate Graft Failure
331 Acute Rejection
332 Coronary Occlusive Disease
333 Hyper acute rejection
334 Chronic rejection
335 Non-specific graft failure
337 Bronchiolitis obliterans
338 Allograft vascular disease
341 Dilated Cardiomyopathy - idiopathic
342 Dilated Cardiomyopathy - familial

343 Dilated Cardiomyopathy - myocarditis
344 Dilated Cardiomyopathy - alcohol
345 Dilated Cardiomyopathy - post partum
346 Dilated Cardiomyopathy - adriamycin
349 Dilated Cardiomyopathy - other, specify
350 Hypertrophic Cardiomyopathy
351 Restrictive Cardiomyopathy
361 Eisenmenger's Syndrome - ASD
362 Eisenmenger's Syndrome - VSD
363 Eisenmenger's Syndrome - PDA
364 Eisenmenger's Syndrome - other
370 Other Congenital Heart/Lung Disease
398 Other
399 Unknown
888 Not reported

CARDIOTHORACIC CAUSES OF FAILURE

0 Graft still functioning
310 Rejection
311 Early allograft dysfunction
312 Acute rejection
320 Infection
330 Coronary occlusive disease
331 Obliterative bronchiolitis
390 Recipient died, graft was functioning at time of death
395 Other identified cause of death
398 Other
399 Unknown

LIVER CAUSES OF FAILURE

0 GRAFT STILL FUNCTIONING
410 ACUTE REJECTION
420 CHRONIC REJECTION
430 PRIMARY NON-FUNCTION
440 ACUTE VASCULAR OCCLUSION
441 VASCULAR OCCLUSION
450 NON-THROMBOTIC INFARCTION
460 DUCTOPENIC REJECTION
470 RECURRENT DISEASE
480 BILIARY COMPLICATIONS
490 RECIP. DIED, GRAFT STILL FUNCTIONING AT T.O.D.
495 OTHER
498 Other, please specify
499 UNKNOWN

LIVER PRIMARY DISEASE

400 Liver disease unknown
410 Chronic liver failure cause unknown
411 Primary biliary cirrhosis
412 Autoimmune chronic active liver disease
413 Hepatitis B cirrhosis

For peer review only

414 Primary sclerosing cholangitis
415 Alpha-1-antitrypsin deficiency
416 Budd-Chiari syndrome (not code 27)
417 Cryptogenic cirrhosis
418 Secondary biliary cirrhosis
419 Alcoholic liver disease
420 Biliary atresia
421 Congenital hepatic fibrosis
422 Wilson's Disease
423 Congenital biliary disease
424 Hepatitis C cirrhosis
425 Paediatric cholestatic liver disease, please specify
426 Non-alcoholic fatty liver disease
427 Acute hepatic failure - Budd-Chiari syndrome
428 Acute hepatic failure - other virus
430 Acute hepatic failure - serologically indeterminate
431 FHF - Viral
432 FHF - Drug induced
433 FHF - Toxin induced
434 Acute hepatic failure - Wilson's disease
435 Acute hepatic failure - HAV
436 Acute hepatic failure - HBV
437 Acute Hepatic Failure - paracetamol hepatotoxicity
438 Acute Hepatic Failure - other drug toxicity
439 Acute Hepatic Failure - other, please specify
440 Malignancy (CA) not classifiable
441 Hepatocellular carcinoma - non-cirrhotic
442 Hepatocellular carcinoma - cirrhotic
443 Cholangiocarcinoma
444 Hepatoblastoma
445 Secondary hepatic malignancy
446 Other mesenchymal tumours
447 Other primary hepatic malignancy, please specify
448 Benign liver tumour
450 Other metabolic liver disease, please specify
451 Cystic fibrosis
452 Primary oxalosis
453 Graft versus host disease
454 Hyperlipidaemia
455 Trauma
456 Tyrosinaemia
457 Familial amyloidosis
458 Familial cholestasis
459 Neonatal hepatitis
460 Polycystic liver disease
461 Hereditary haemochromatosis
462 Glycogen storage disease
463 Progressive familial intrahepatic cholestasis
464 Neonatal haemochromatosis
465 Niemann-Pick type C
466 Hypercholesterolaemia
467 Urea cycle disorder
468 Crigler-Najjar syndrome

For peer review only

469 Cholesterol ester storage disease
470 Alper's syndrome
471 Acute rejection
472 Chronic rejection
473 Primary non-function
474 Acute vascular occlusion (artery plus vein)
475 Non-thrombotic infarction
476 Ductopenic rejection
477 Recurrent disease
478 Biliary complications
479 Hepatic artery thrombosis
480 Early graft dysfunction
481 Acute vascular occlusion - venous
482 Acute vascular occlusion - artery and venous
483 Mitochondrial disease
484 Alagilles syndrome
485 Neonatal sclerosing cholangitis
486 Other neonatal cholestasis
498 Other, please specify
499 Unknown
888 Not reported

OCULAR TISSUE RECIPIENT DIAGNOSIS

110 KERATOCONUS
111 KERATOGLOBUS
117 OTHER ECTASIAS
120 FUCHS' DYSTROPHY
127 OTHER DYSTROPHIES
131 PSEUDOPHAKIC CORNEAL OEDEMA
132 APHAKIC CORNEAL OEDEMA
133 COMPLICATIONS OF REFRACTIVE SURGERY
137 OTHER PREVIOUS OCULAR SURGERY
141 VIRAL INFECTION
142 BACTERIAL INFECTION
143 FUNGAL INFECTION
144 PROTOZOAN INFECTION
147 OTHER INFECTION
151 MECHANICAL INJURY
152 CHEMICAL INJURY
157 OTHER INJURY
163 RHEUMATOID ARTHRITIS
167 OTHER ULCERATIVE KERATITIS
171 REGRAFT - GRAFT FAILURE
172 REGRAFT - REJECTION
173 REGRAFT - ENDOTHELIAL FAILURE
174 REGRAFT - HIGH ASTIGMATISM
176 REGRAFT - ENDOPHTHALMITIS
177 REGRAFT - OTHER CAUSE OF FAILURE
181 INTERSTITIAL KERATITIS
182 DERMOID OPACIFICATION
183 NEOPLASIA
184 PTERYGIUM
187 OTHER OPACIFICATION

For peer review only - http://bmjopen.bmj.com/site/about/guidelines.xhtml

191 ADULT APHAKIA
192 PAEDIATRIC APHAKIA
193 PRIMARY ORBITAL IMPLANT
194 SECONDARY ORBITAL IMPLANT
195 REPAIR OF LOCALISED SCLERAL DEFECT
198 OTHER
199 NOT REPORTED

For peer review only

r review only

r review only

HLA mismatch combinations included

0
100, 010, 110, 200, 210
020, 120, 220, 001, 101, 201, 011, 111, 211
021, 121, 221, 002, 102, 202, 012, 112, 212, 022, 122,

view only

Public Health England

Protecting and improving the nation's health

NCRAS Cancer Registration Da

Access to cancer registration data

The NCRAS data dictionary contains attribute lists that are c
for Data Release (ODR). Prospective applicants are advisec
To access the support available through the NCRAS analyti

Data availability

The following datasets are available for request:

Tables
Cancer registration (patient table)
Cancer registration (tumour table)
Cancer registration (treatment table)
Index of Multiple Deprivation_Income domain
Route to diagnosis
National Radiotherapy Dataset (RTDS)
Systemic Anti-Cancer Therapy Dataset
HES admitted care
HES outpatient
HES accident and emergency
NBOCAP
Cancer Patient Experience Survey
Cancer Waiting Times
Quality of Life of Cancer Survivors in England: (Breast, Colorectal, Prostate, Non-Hodgkin's Lymphoma)
Lung Cancer Data Audit (LUCADA)
Quality of Life of Colorectal Cancer Survivors in England: Patient Reported Outcome Measures Survey (PROMS)

For peer review only

Selecting data fields

In using these attribute lists, users are advised requests for
the body of the associated protocol will not be disclosed. Ap

- all relevant coding classifications (i.e. ICD 10 C61 and
- all relevant geographies (i.e. All England, patients res
- time period(s) (temporality)

Privacy by design and default

In assessing applications to access PHE data, the ODR wor intended purpose. As part of this approach we have highligh fields. Prospective applicants are encouraged to apply priva in our privacy principles include:

**Aggregation**	**Age bands** will be suppli anonymity checks.<br>**Ethnic categorisation** sl<br>**Geographic data** should
**Partial data removal**	**Dates** (such as date of bi possible to provide event sensitive data.
**Masking**	**Provider, Consultant ar** data to be observed whil
**De-duplication**	Only one **deprivation in**<br>Where similar items are a

The ODR encourages applicants to apply data minimisation have any questions please contact the Data Access and Co

For more information on NCRAS datasets, you can also rea
[PHE cancer data sets, linkage and availability]

First published: June 2018

© Crown copyright 2018
Re-use of Crown copyright material (excluding logos) is allo
www.nationalarchives.gov.uk/doc/open-government-licence/
for terms and conditions.

For peer review only - http://bmjopen.bmj.com/site/about/guidelines.xhtml

For peer review only - http://bmjopen.bmj.com/site/about/guidelines.xhtml

For peer review only - http://bmjopen.bmj.com/site/about/guidelines.xhtml

For peer review only - http://bmjopen.bmj.com/site/about/guidelines.xhtml

For peer review only - http://bmjopen.bmj.com/site/about/guidelines.xhtml

For peer review only - http://bmjopen.bmj.com/site/about/guidelines.xhtml

For peer review only - http://bmjopen.bmj.com/site/about/guidelines.xhtml

For peer review only - http://bmjopen.bmj.com/site/about/guidelines.xhtml

ly

ly

For peer review only - http://bmjopen.bmj.com/site/about/guidelines.xhtml

For peer review only - http://bmjopen.bmj.com/site/about/guidelines.xhtml

For peer review only - http://bmjopen.bmj.com/site/about/guidelines.xhtml

For peer review only - http://bmjopen.bmj.com/site/about/guidelines.xhtml

r peer review only

ata Dictionary v3.4

designed to support the co-production of a data specification for a specific project, ahead of submis
d to seek expert support from a site, geographic or asset specific analyst to confirm and validate the
cal network, please contact ODR@phe.gov.uk

Coverage	Available as a discrete dataset (not linked)
1 January 1985 - 31 December 2016	x
1 January 1985 - 31 December 2016	x
1 January 1985 - 31 December 2016	x
To latest available date	
1 January 2006 - 31 December 2015	
1 April 2009 - 31 March 2016	
1 April 2012 - 30 June 2016	
1 April 1997 - 31 December 2015	*Please contact the NHS Digital Data Access Request Service (DARs)*
1 April 1997 - 31 December 2015	
1 April 1997 - 31 December 2015	
1 April 2006 - 31 March 2013	
Please see relevant sheet regarding each wave of the survey	*Please contact Quality Health*
1 January 2009 - 31 December 2014	*Please contact NHS England*
July 2006 - July 2010	x
1 January 2005 - 31st December 2013	x
1 January 2010 - 31 December 2011	x

data must be limited to the minimum necessary to accomplish the intended purpose. Fields that fai
plicants are further advised to summarise the following requirements in the 'Request Summary' tab

D07)
iding within CCG, patients treated at NHS Trust)

rk to 'Privacy by Design and Default' principles to ensure that any data released is the minimum ne
nted items in this data dictionary where these principles have been applied, including a number of c
cy by design and default practices when identifying the data required for the project. Examples of t

ied instead of precise age. These bandings may be in the standard 5yr banding or broader groups

hould be as broad as possible, full ethnic categorisation breakdown often causes data to fail k-anon
l be at the highest level to allow analysis to be undertaken.

irth) will, by default, be supplied as month and year only. Precise dates will require additional justifi
t intervals, please consider if this would allow the intended analysis to be undertaken without the ne

nd GP Practice codes** data will be pseudonymised by default. The aim of this measure is to allow
lst ensuring there is no reputational risk for organisations or individuals identified within the data

dex will only be supplied, multiple indices allow the identification of the geographies to which the in
available in multiple data sources, by default, **data will only be supplied from a single source**.

techniques for your proposed project, to ensure your application can be processed as quickly as p
nfidentiality Manager assigned to your application or the ODR directly at ODR@phe.gov.uk

d the 'PHE cancer data sets, linkage and availability' document.

wed under the terms of the Open Government Licence, visit
/version/3/

For peer review only - http://bmjopen.bmj.com/site/about/guidelines.xhtml

For peer review only

ssion to the PHE Office
eir data requirements.

Available as linked resource only
x
x
x
x
x
x
x
x
x
x

il to be justified within
):

ɛcessary for the
commonly derived
the data items included

if required to pass k-<br>nymity checks.
ication. It may be<br>eed to release
variation within the
ndices apply.

possible. Should you

For peer review only - http://bmjopen.bmj.com/site/about/guidelines.xhtml

For peer review only

For peer review only - http://bmjopen.bmj.com/site/about/guidelines.xhtml

For peer review only - http://bmjopen.bmj.com/site/about/guidelines.xhtml

For peer review only - http://bmjopen.bmj.com/site/about/guidelines.xhtml

r peer review only

For peer review only

Provide a summary of the data required details below - and then select items you require in the appropriate workshe

All applicants must select and justify each data item required as part of the ODR request application.

Summary of request - Please provide a summary of the data being requested, outlining which of the available d

We are requesting data from 'CANCER REG-PATIENT TABLE', 'CANCER REG-TUMOUR TABLE', 'CANCER REG-TREATMENT TABLE', SACT and RTDS. The entire cohort is 1 Jan 1985 to 31 Dec 2016, with data required up to latest available. We understand that some cohorts (especially SACT and RTDS) do not encompass this entire period but we are requesting everything that is available.

Cancer Sites/Morphologies – Please provide the cancer sites and/or morphologies required for the request separated by commas and the coding system used. If combinations of site/ morphology are required please separate site and morphology with hyphens. If all codes within a tumour site grouping are required an 'x' may be used to suffix the 3 character grouping. (For example: C18x, C19x, C20x, C44 – 80903, C44 – 81703, C56.1, C56.2)

All cancer sites and morphologies requested.

Geography or treatment provider criteria – Please provide us with the geography for the data provided, if data are required for all of England please state this. If data are required for particular geographies/provider please state the geography level, the required geographies and how these geographies should be applied to the data. (For example: CCGs 07X, 08V, 08B defined by patient treatment within trust located in one of these CCGs)

We require data for all of England.

Time period criteria - individual years or a range of years. Time period should also describe which dataset time period applies to, e.g. all patients with a diagnosis date between 2000-2010 or patients with any inpatient HES activity in the trusts defined above in 2015. Please also indicate clearly if the date is diagnostic date, treatment date, event date or a

All patients with any diagnosis between 1st January 1985 and 31st December 2017

For peer review only

Introduction

To achieve comprehensive registration for all registerable tumours, the NCRA
treatment from diagnosis. The CAS AV 2016 tables includes data on the patie

Data item selection

Please be aware that selecting patient identifiable data items – **the data item**
statutory regulation covering your organisation. Sensitive patient demographic
applied to render the data anonymised to k-3 anonymity. Privacy by design ar
key explaining how these alternative can be selected is included below:

Key:

Items in red Identifiable fields that require additional permissions to access
Sensitive items that require additional justification in your protoc
Privacy by design and default' alternatives to the sensitive items

Item number	Data item
1	Pseudonymised patient ID
2	**NHS number**
3	Alias check flag - patient
4	**Date of Birth**
5	Month of birth
6	Year of birth
7	Date of Birth check flag - patient
8	Sex
9	Ethnicity
10	Ethnic group
11	Broad ethnic group
12	Vital status of the patient
13	**Date of death of the patient**
14	Month of death of the patient
15	Year of death of the patient
16	Days from another event to date to death
17	Date of death imputed flag
18	Embarkation flag
19	Date of embarkation
20	Month of embarkation
21	Year of embarkation
22	Days from another event to embarkation
23	As provided with death notification
24	As provided with death notification

**25**	As provided with death notification
**26**	As provided with death notification
**27**	As provided with death notification
**28**	Code of the location (type) where the patient died, e.g. patients home, hospice etc.
**29**	Description of the location (type) where the patient died, e.g. patients home, hospice etc.
**30**	Code of institution at which death takes place
**31**	Indicates whether a post-mortem took place
**32**	Count of every tumour assigned to this PatientID.
**33**	Count of every tumour assigned to this PatientID in range C00-97 excl C44

************************ **End of sheet** ***********************

For peer review only

AS brings together data from more than 500 local and regional datasets to build a picture of an individual's ent, their diagnosis, tumour and treatment events.

ıs marked below in red – will require you to have patient consent, section 251 approval, or the appropriate c data, event dates and geographies will be treated as de-personalised, unless anonymisation techniques are nd default principles have also been applied to some of these data to provide a less disclosive alternative. The

(as outlined above)
col to access
s highlighted in dark grey

Field name	Request data item	Justification
**Av_patient**		
PATIENTID	**X**	This will be done by NHS Digital
**NHSNUMBER**	**X**	Necessary for record linkage by NHS Digital
ALIASFLAG		
**BIRTHDATEBEST**	X	Necessary for record linkage by NHS Digital
MONTH_DOB		
YEAR_DOB		
BIRTHDATEFLAG		
SEX	X	Necessary for record linkage by NHS Digital but also to investigate cancer risk by baseline demographics.
ETHNICITY	X	To investigate cancer risk by baseline demographics.
ETHNICITYNAME	X	To investigate cancer risk by baseline demographics.
Option to group ethnicities (e.g. white/ non-white/ unknown)		
VITALSTATUS		
**DEATHDATEBEST**	X	To investigate cancer mortality risk.
MONTH_DOD		
YEAR_DOD		
Option to provide number of days from another event to death (e.g. days from diagnosis to death)	X	To investigate days from first diagnosis to death.
DEATHDATEFLAG		
EMBARKATION		
EMBARKATIONDATE		
Month of embarkation		
Year of embarkation		
Option to provide number of days from another event to embarkation (e.g. days from diagnosis to embarkation		
DEATHCAUSECODE_1A	X	To investigate cancer mortality aetiology (cancer versus other causes)
DEATHCAUSECODE_1B	X	To investigate cancer mortality aetiology (cancer versus other causes)

DEATHCAUSECODE_1C	X	To investigate cancer mortality aetiology (cancer versus other causes)
DEATHCAUSECODE_2	X	To investigate cancer mortality aetiology (cancer versus other causes)
DEATHCAUSECODE_UNDERLYING	X	To investigate cancer mortality aetiology (cancer versus other causes)
DEATHLOCATIONCODE		
DEATHLOCATIONDESC		
SITECODEOFDEATH		
POSTMORTEM		
TUMOURCOUNT	X	To understand overall cancer burden.
BIGTUMOURCOUNT	X	To understand overall cancer burden.

For peer review only

view only

Introduction

To achieve comprehensive registration for all registerable tumours, the NCRS brings tog
diagnosis. The CAS AV 2016 includes data on the patient, their diagnosis, tumour and tr

Data item selection

Please be aware that selecting patient identifiable data items – **the data items marked**
regulation covering your organisation. Sensitive patient demographic data, event dates a
anonymised to k-3 anonymity. Privacy by design and default principles have also been a
be selected is included below:

Key:

**Items in red**	Identifiable fields that require additional permissions to access (as outline
	Sensitive items that require additional justification in your protocol to acce
	Privacy by design and default' alternatives to the sensitive items highlight

Item number	Data item
1	Pseudonymised tumour ID
2	Pseudonymised patient ID
3	**ONS ID**
4	**NHS Number**
5	**Date of Birth**
6	Month of birth
7	Year of birth
8	Age at diagnosis
9	Age at diagnosis in 5 year age bands (0-4 etc.)
10	Sex
11	**Postcode at Diagnosis**
12	Broader geographic area/ IMD quintile
13	Ethnicity
14	Broad ethnic group
15	Earliest date when the diagnosis may have taken place
16	Latest date when the diagnosis may have taken place
17	Diagnosis date
18	Month of diagnosis
19	Year of diagnosis
20	Days from another event to date to diagnosis
21	Date of diagnosis imputed flag
22	Financial year of diagnosis
23	Basis of diagnosis of the tumour
24	Diagnosis death certificate only
25	Site of neoplasm (4-character ICD-10-O2 code)
26	Site of neoplasm (3-character ICD-10-O2 code)
27	Site of the cancer
28	Description of the code in SITE_CODED
29	3 digit version of SITE_CODED
30	The coding system used to register the tumour

31	Description of coding system used in registration
32	Morphology
33	Morphology of the cancer, in the ICD-10-O2 system
34	Behaviour of the cancer, in the ICD-10-O2 system
35	Numeric behaviour code
36	Description of behaviour code
37	Histology code
38	Description of histology code
39	Grade of tumour
40	Size of the largest dimension of the tumour, in mm
41	Number of nodes excised
42	Number of nodes involved
43	Laterality
44	Multifocal
45	Oestrogen receptor status of the tumour
46	Oestrogen receptor score of the tumour.
47	Progesterone receptor status of the tumour
48	Progesterone receptor score of the tumour
49	HER2 status of the tumour
50	Nottingham Prognostic Index Score
51	Dukes' stage
52	FIGO stage
53	Clark's stage
54	Breslow thickness of tumour
55	Gleason primary pattern
56	Gleason secondary pattern
57	Gleason tertiary pattern
58	Combined Gleason primary and secondary scores
59	T stage (pre-treatment)
60	N stage (pre-treatment)
61	M stage (pre-treatment)
62	Stage at diagnosis derived from imaging
63	System used to record imaging stage at diagnosis
64	T stage (pathology)
65	N stage (pathology)
66	M stage (pathology)
67	Pathological stage at diagnosis
68	System used to record pathological stage at diagnosis
69	Pathological stage at diagnosis (pre-treatment)
70	T stage flagged by the registry as the ‘best’ T stage
71	N stage flagged by the registry as the ‘best’ N stage
72	M stage flagged by the registry as the ‘best’ M stage
73	Best ‘registry’ stage at diagnosis of the tumour
74	System used to record best registry stage at diagnosis
75	Code for the place where the diagnosis episode took place
76	Pseudonymised diagnosis provider code
77	Description of DIAGNOSISPROVIDER_CODE
78	Code for the Trust at diagnosis
79	Pseudonymised diagnosis trust code
80	Name of the trust at diagnosis
81	Tumour registration status
82	Excision margin
83	Screen detected cancer
84	Screening status of the tumour
85	Description of SCREENINGSTATUSCOSD_CODE
86	Full detailed screening status of the tumour
87	Description of SCREENINGSTATUSFULL_CODE
88	Date of first recorded event in treatment table
89	Month of first recorded event in treatment table
90	Year of first recorded event in treatment table

For peer review only

**91**	Days from another event to first recorded event
**92**	Trust code of first recorded event in treatment table
**93**	Pseudonymised trust code of first event
**94**	Name of trust for first recorded event in treatment table
**95**	Date of first recorded surgery in treatment table
**96**	Month of first recorded surgery in treatment table
**97**	Year of first recorded surgery in treatment table
**98**	Days from another event to first recorded surgery in treatment table
**99**	Trust code of first recorded surgery in treatment table
**100**	Pseudonymised trust code of first recorded surgery
**101**	Name of trust for first recorded surgery in treatment table
**102**	2011 Lower Super Output Area
**103**	2001 Lower Super Output Area
**104**	2011 Middle Super Output Area
**105**	2001 Middle Super Output Area
**106**	Clinical Commissioning Group code (at diagnosis)
**107**	Name of the Clinical Commissioning Group
**108**	Primary Care Trust code the patient was resident in when the tumour was diagnosed
**109**	Name of the Primary Care Trust the patient was resident in when the tumour was diagnosed
**110**	Local Authority Unitary Authority code the patient was resident in when the tumour was diagnosed
**111**	Name of the Local Authority Unitary Authority the patient was resident in when the tumour was diagnosed
**112**	Upper tier Local Authority code the patient was resident in when the tumour was diagnosed
**113**	Name of the upper tier Local Authority the patient was resident in when the tumour was diagnosed
**114**	Strategic Clinical Network code the patient was resident in when the tumour was diagnosed
**115**	Name of the Strategic Clinical Network the patient was resident in when the tumour was diagnosed
**116**	Cancer network code the patient was resident in when the tumour was diagnosed
**117**	Name of the cancer network the patient was resident in when the tumour was diagnosed
**118**	County code the patient was resident in when the tumour was diagnosed
**119**	Name of the county the patient was resident in when the tumour was diagnosed
**120**	Government office region code the patient was resident in when the tumour was diagnosed
**121**	Name of the government office region the patient was resident in when the tumour was diagnosed
**122**	Cancer registry catchment area code the patient was resident in when the tumour was diagnosed
**123**	Name of the cancer registry catchment area the patient was resident in when the tumour was diagnosed
**124**	Country code the patient was resident in when the tumour was diagnosed
**125**	Name of the country the patient was resident in when the tumour was diagnosed
**126**	Cancer registry code which finalised the case and was responsible for sending it to ONS if it was an in-region case

For peer review only

**127**	Name of the cancer registry which finalised the case and was responsible for sending it to ONS if it was an in-region case

************************* **End of sheet** *************************

For peer review only

gether data from more than 500 local and regional datasets to build a picture of an individual's treatment from reatment events.

below in red – will require you to have patient consent, section 251 approval, or the appropriate statutory and geographies will be treated as de-personalised, unless anonymisation techniques are applied to render the data applied to some of these data to provide a less disclosive alternative. The key explaining how these alternative can

d above)
ess
ed in dark grey

Field name	Request data item	Justification
**Av_tumour**		
TUMOURID		
PATIENTID		
**ONSID**		
**NHSNUMBER**	X	Necessary for record linkage by NHS Digital
**BIRTHDATEBEST**	X	Necessary for record linkage by NHS Digital
MONTH_DOB		
YEAR_DOB		
AGE	X	To investigate cancer risk by baseline demographics.
FIVEYEARAGEBAND	X	To investigate cancer risk by baseline demographics.
SEX	X	Necessary for record linkage by NHS Digital but also to investigate cancer risk by baseline demographics.
**POSTCODE**	X	Necessary for record linkage by NHS Digital
Option to provide geography as deprivation score or aggregate to larger geographic areas such as MSOA or county.	X	IMD quintile to investigate cancer risk b
ETHNICITY	X	To investigate cancer risk by baseline demographics.
Option to group ethnicities (e.g. white/ non-white/ unknown)		
DIAGNOSISDATE1	X	For time survival analyses and determir
DIAGNOSISDATE2	X	For time survival analyses and determir
DIAGNOSISDATEBEST	X	For time survival analyses and determir
DIAGNOSISMONTH		
DIAGNOSISYEAR		
Option to provide number of days from another event to diagnosis (e.g. days from birth to diagnosis)	X	From birth to date of diagnosis
DIAGNOSISDATEFLAG		
FINANCIALYEAR		
BASISOFDIAGNOSIS		
DCO		
SITE_ICD10_O2	X	To better understand cancer morpholog
SITE_ICD10_O2_3CHAR	X	To better understand cancer morpholog
SITE_CODED	X	To better understand cancer morpholog
SITE_CODED_DESC	X	To better understand cancer morpholog
SITE_CODED_3CHAR	X	To better understand cancer morpholog
CODING_SYSTEM	X	To better understand cancer morpholog

CODING_SYSTEM_DESC	X	To better understand cancer morpholog
MORPH_CODED	X	To better understand cancer morpholog
MORPH_ICD10_O2	X	To better understand cancer morpholog
BEHAVIOUR_ICD10_O2	X	To better understand cancer morpholog
BEHAVIOUR_CODED	X	To better understand cancer morpholog
BEHAVIOUR_CODED_DESC	X	To better understand cancer morpholog
HISTOLOGY_CODED	X	To better understand cancer morpholog
HISTOLOGY_CODED_DESC	X	To better understand cancer morpholog
GRADE	X	To better understand cancer morpholog
TUMOURSIZE	X	To better understand cancer morpholog
Nodes_excised_new	X	To better understand cancer morpholog
nodes_involved_new	X	To better understand cancer morpholog
LATERALITY	X	To better understand cancer morpholog
MULTIFOCAL	X	To better understand cancer morpholog
ER_STATUS	X	To better understand cancer morpholog
ER_SCORE	X	To better understand cancer morpholog
PR_STATUS	X	To better understand cancer morpholog
PR_SCORE	X	To better understand cancer morpholog
HER2_STATUS	X	To better understand cancer morpholog
NPI	X	To better understand cancer morpholog
DUKES	X	To better understand cancer morpholog
FIGO	X	To better understand cancer morpholog
CLARKS	X	To better understand cancer morpholog
BRESLOW	X	To better understand cancer morpholog
GLEASON_PRIMARY	X	To better understand cancer morpholog
GLEASON_SECONDARY	X	To better understand cancer morpholog
GLEASON_TERTIARY	X	To better understand cancer morpholog
GLEASON_COMBINED	X	To better understand cancer morpholog
T_IMG	X	To better understand cancer morpholog
N_IMG	X	To better understand cancer morpholog
M_IMG	X	To better understand cancer morpholog
STAGE_IMG	X	To better understand cancer morpholog
STAGE_IMG_SYSTEM	X	To better understand cancer morpholog
T_PATH	X	To better understand cancer morpholog
N_PATH	X	To better understand cancer morpholog
M_PATH	X	To better understand cancer morpholog
STAGE_PATH	X	To better understand cancer morpholog
STAGE_PATH_SYSTEM	X	To better understand cancer morpholog
STAGE_PATH_PRETREATED	X	To better understand cancer morpholog
T_BEST	X	To better understand cancer morpholog
N_BEST	X	To better understand cancer morpholog
M_BEST	X	To better understand cancer morpholog
STAGE_BEST	X	To better understand cancer morpholog
STAGE_BEST_SYSTEM	X	To better understand cancer morpholog
DIAGNOSISPROVIDER_CODE		
DIAGNOSISPROVIDER_CODE (pseudonymised)		
DIAGNOSISPROVIDER_NAME		
DIAGNOSISTRUST_CODE	X	To understand cancer burden and deliv
DIAGNOSISTRUST_CODE (pseudonymised)		
DIAGNOSISTRUST_NAME	X	To understand cancer burden and deliv
STATUSOFREGISTRATION	X	To better understand cancer status
EXCISIONMARGIN	X	To better understand cancer status
SCREENDETECTED	X	To understand screening success for p
SCREENINGSTATUSCOSD_CODE	X	To understand screening success for p
SCREENINGSTATUSCOSD_NAME	X	To understand screening success for p
SCREENINGSTATUSFULL_CODE	X	To understand screening success for p
SCREENINGSTATUSFULL_NAME	X	To understand screening success for p
DATE_FIRST_EVENT	X	To understand cancer burden and deliv
Month of first recorded event in treatment table	X	To understand cancer burden and deliv
Year of first recorded event in treatment table	X	To understand cancer burden and deliv

For peer review

Option to provide number of days from another event to the first recorded event in the treatment table (e.g. days from diagnosis to first treatment event)	X	Days from diagnosis to first treatment e
TRUSTCODE_FIRST_EVENT	X	To understand cancer burden and deliv
TRUSTNAME_FIRST_EVENT	X	To understand cancer burden and deliv
DATE_FIRST_SURGERY	X	To better understand cancer treatment
Month of first recorded surgery in treatment table	X	To better understand cancer treatment
Year of first recorded surgery in treatment table	X	To better understand cancer treatment
Option to provide number of days from another event to the first recorded surgery (e.g. days from diagnosis to first recorded surgery)	X	Days from diagnosis to first recorded su
TRUSTCODE_FIRST_SURGERY	X	To understand cancer burden and deliv
TRUSTCODE_FIRST_SURGERY (pseudonymised)		
TRUSTNAME_FIRST_SURGERY	X	To understand cancer burden and deliv
LSOA11_CODE		
LSOA01_CODE		
MSOA11_CODE		
MSOA01_CODE		
CCG_CODE		
CCG_NAME		
PCT_CODE		
PCT_NAME		
LAUA_CODE		
LAUA_NAME		
UTLA_CODE		
UTLA_NAME		
SCN_CODE		
SCN_NAME		
CNET_CODE		
CNET_NAME		
COUNTY_CODE		
COUNTY_NAME		
GOR_CODE		
GOR_NAME		
CREG_CODE		
CREG_NAME		
CTRY_CODE		
CTRY_NAME		
CENTRE		

For peer review only

CENTRENAME		

For peer review only

py baseline demographics

ne time from date of diagnosis from date of transplant
ne time from date of diagnosis from date of transplant
ne time from date of diagnosis from date of transplant

gy
gy
gy
gy
gy
gy

only

gy
gy
gy
gy
gy
gy
gy
gy
gy
gy
gy
gy
gy
gy
gy
gy
gy
gy
gy
gy
gy
gy
gy
gy
gy
gy
gy
gy
gy
gy
gy
gy
gy
gy
gy
gy
gy
gy
gy
gy
gy
gy
gy
gy

very of care post-transplant

very of care post-transplant

ost-transplant cancer detection
ost-transplant cancer detection
ost-transplant cancer detection
ost-transplant cancer detection
ost-transplant cancer detection
very of care post-transplant
very of care post-transplant (in case above not available)
very of care post-transplant (in case above not available)

:vent
/ery of care post-transplant

/ery of care post-transplant

(if above not available)
(if above not available)

urgery
/ery of care post-transplant

/ery of care post-transplant

For peer review only

For peer review only

For peer review only

For peer review only

Introduction

To achieve comprehensive registration for all registerable tumours, the NCRS bri
treatment from diagnosis. The CAS AV 2016 includes data on the patient, their di

Data item selection

Please be aware that selecting patient identifiable data items – **the data items m**
statutory regulation covering your organisation. Sensitive patient demographic da
applied to render the data anonymised to k-3 anonymity. Privacy by design and d
key explaining how these alternative can be selected is included below:

Key:

Items in red Identifiable fields that require additional permissions to access (as
Sensitive items that require additional justification in your protocol t
Privacy by design and default' alternatives to the sensitive items hi

Item number	Data item
1	Pseudonymised event ID
2	Pseudonymised patient ID
3	Pseudonymised tumour ID
4	Age at diagnosis
5	Age at diagnosis in 5 year age bands (0-4 etc.)
6	Age at diagnosis in x year age bands
7	Sex
8	Diagnosis date
9	Month of diagnosis
10	Year of diagnosis
11	Days from another event to date to diagnosis
12	Number of tumours affected by this event
13	Type of event code
14	Description of the event
15	Date the event took place
16	Month the event took place
17	Year the event took place
18	Days from another event to this event
19	Treatment provider (organisation code)
20	Pseudonymised treatment provider code
21	Name of the organisation where the event took place
22	Code of the NHS Trust where the event took place
23	Pseudonymised NHS Trust code where the event took place
24	Name of the NHS Trust where the event took place
25	Consultant code
26	Consultant code (pseudonymised by default)
27	Consultant name
28	Cancer registry catchment area code the patient was resident in when the tumour was diagnosed
29	Treatment within 6 months of diagnosis - check flag
30	Treatment six months from date of diagnosis - check flag

**31**	Operations, procedures and interventions (OPCS-4)
**32**	Name of the operations, procedures and interventions
**33**	Radiotherapy code
**34**	Radiotherapy description
**35**	Imaging code – internal coding system
**36**	Description of imaging
**37**	Site on body where imaging occurred
**38**	Size in millimetres of the diameter of a lesion (histology)

************************** **End of sheet** **************************

For peer review only

ings together data from more than 500 local and regional datasets to build a picture of an individual's
iagnosis, tumour and treatment events.

narked below in red – will require you to have patient consent, section 251 approval, or the appropriate
ata, event dates and geographies will be treated as de-personalised, unless anonymisation techniques are
default principles have also been applied to some of these data to provide a less disclosive alternative. The

outlined above)
to access
ghlighted in dark grey

Field name	Request data item	Justification
**AV_treatment**		
EVENTID		
TUMOURID		
PATIENTID		
AGE	X	To investigate cancer risk by baseline demographics.
FIVEYEARAGEBAND	X	To investigate cancer risk by baseline demographics.
Option to provide age in broad categories (e.g. =<45, 46-55, 56-65, >65)		
SEX	X	To investigate cancer risk by baseline demographics.
DIAGNOSISDATEBEST	X	**To determine time span from transplant**
DIAGNOSISMONTH	X	**To determine time span from transplant (in case**
DIAGNOSISYEAR	X	**To determine time span from transplant (in case**
Option to provide number of days from another event to diagnosis (e.g. days from birth to diagnosis)	X	**Days from birth to diagnosis**
NUMBER_OF_TUMOURS	X	To better understand cancer morphology, nature ar
EVENTCODE	X	To better understand cancer morphology, nature ar
EVENTDESC	X	To better understand cancer morphology, nature ar
EVENTDATE	X	To better understand cancer morphology, nature ar
Month of the year the event took place	X	To better understand cancer morphology, nature ar
EVENTYEAR	X	To better understand cancer morphology, nature ar
Option to provide number of days from another recorded event to this event (e.g. days from diagnosis to event)	X	**Days from birth to event**
PROVIDERCODE	X	To understand cancer burden and delivery of care p
PROVIDERCODE (pseudonymised)		
PROVIDERDESC		
TRUST_CODE	X	To understand cancer burden and delivery of care p
TRUST_CODE (pseudonymised)		
TRUST_NAME	X	To understand cancer burden and delivery of care p
PRACTITIONERCODE		
PRACTITIONERCODE (pseudonymised)		
PRACTITIONERDESC		
CREG_CODE		
WITHIN_SIX_MONTHS_FLAG	X	To better understand cancer treatment
SIX_MONTHS_AFTER_FLAG	X	To better understand cancer treatment

OPCS4_CODE	X	To better understand cancer treatment
OPCS4_NAME	X	To better understand cancer treatment
RADIOCODE	X	To better understand cancer treatment
RADIODESC	X	To better understand cancer treatment
IMAGINGCODE	X	To better understand cancer treatment
IMAGINGDESC	X	To better understand cancer treatment
IMAGINGSITE	X	To better understand cancer treatment
LESIONSIZE	X	To better understand cancer treatment

For peer review only

e above not available)
e above not available)

nd burden
nd burden
nd burden
nd burden
nd burden
nd burden

post-transplant

post-transplant

post-transplant

For peer review only

For peer review only

For peer review only

Introduction

The Income Deprivation Domain measures the proportion of the population experiencin
those that are in work but who have low earnings (and who satisfy the respective means

Data items selection

Please be aware that selecting patient identifiable data items – **the data items marked**
covering your organisation. Sensitive patient demographic data, event dates and geogr
3 anonymity.

Note: only one deprivation quintile year can be selected for each application to reduce t

Item number	Data item
1	Pseudonymised patient ID
2	Pseudonymised tumour ID
3	Measure of deprivation at small area level made up from the income domain in 2004, quintiles are calculated from populations
4	Measure of deprivation at small area level made up from the income domain in 2007, quintiles are calculated from populations
5	Measure of deprivation at small area level made up from the income domain in 2010, quintiles are calculated from populations
6	Measure of deprivation at small area level made up from the income domain in 2015, quintiles are calculated from populations

****

For peer review only

g deprivation relating to low income. The
s tests).

below in red – will require you to have p
raphies will be treated as de-personalised

the risk of deductive disclosure based on

Field name	Request data item
PATIENTID	
TUMOURID	
IMD2004	
IMD2007	
IMD2010	
quintile_2015	X

************* **End of sheet** *****************

For peer review only

definition of low income used includes both those people that are out-of-work, and

›atient consent, section 251 approval, or the appropriate statutory regulation
l, unless anonymisation techniques are applied to render the data anonymised to k-

low level geographies.

Justification
IMD quintile to investigate cancer risk by baseline demographics

****

Introduction

Inpatient HES contains data on all inpatient admissions at NHS hospitals in Engla
paid for the care they deliver. HES data is designed to enable secondary use, that

Data items selection

Please be aware that selecting patient identifiable data items – **the data items ma**
appropriate statutory regulation covering your organisation. Sensitive patient dem
anonymisation techniques are applied to render the data anonymised to k-3 anony
provide a less disclosive alternative. The key explaining how these alternative can
registration data.

Key:

Items in red Identifiable fields that require additional permissions to access (as ou
Sensitive items that require additional justification in your protocol to
Privacy by design and default' alternatives to the sensitive items high

Item number	Data item
1	Pseudonymised patient ID
2	Administrative & legal status of patient
3	Administrative category
4	Age at start of episode
5	**Date of birth - patient**
6	Month of birth
7	Year of birth
8	Ethnic category
9	Broad ethnic group
10	**Local patient identifier**
11	**NHS number**
12	**HES patient identifier**
13	Postcode district of patient residence
14	**Postcode of patient residence**
15	Broader geographic area/ IMD quintile
16	Sex of patient
17	Date of admission
18	Month of admission
19	Year of admission
20	Days from another event to admission
21	Date of decision to admit
22	Month of decision to admit
23	Year of decision to admit
24	Days from another event to date of decision to admit
25	Method of admission
26	Source of admission
27	First regular day or night admission
28	Waiting time
29	Class of patient

**30**	Date of discharge
**31**	Month of discharge
**32**	Year of discharge
**33**	Days from another event to date of discharge
**34**	Destination on discharge
**35**	Method of discharge
	**Ep**
**36**	Bed days within the year
**37**	Beginning of spell
**38**	Date episode ended
**39**	Month the episode ended
**40**	Year the episode ended
**41**	Days from another event to date episode ended
**42**	Date episode started
**43**	Month the episode started
**44**	Year the episode started
**45**	Days from another event to date episode started
**46**	Duration of spell
**47**	End of spell
**48**	Episode duration
**49**	Episode order
**50**	Episode type
**51**	Hospital provider spell number (pseudonymised by default)
**52**	All diagnosis codes
**53**	Primary diagnosis (3 characters) (derived)
**54**	Primary diagnosis (4 characters)
**55**	All operative procedure codes
**56**	Date of operations
**57**	Month of operations
**58**	Year of operations
**59**	Days from another event to date of operation
**60**	Operation status code
**61**	Intended management
**62**	Main specialty
**63**	Treatment specialty
	**Health**
**64**	Dominant procedure
**65**	Healthcare resource group (Applied HRG code from 2006-07 onwards)
**66**	NHS-generated HRG code
**67**	NHS-generated HRG code version number
**68**	SUS generated core spell HRG
**69**	SUS generated HRG
**70**	SUS generated HRG version number
**71**	SUS generated spell ID
**72**	Commissioner code
**73**	Commissioner code status

**74**	Commissioner's regional office
**75**	Commissioner's strategic health authority
**76**	Commissioning serial number
**77**	Health authority where patients GP was registered
**78**	Primary care group
**79**	Primary care trust of responsibility - historic
**80**	Primary care trust of responsibility - current
**81**	Primary care trust area where patient's GP was registered
**82**	Provider code - 5 character
**83**	Pseudonymised provider code - 5 character
**84**	Provider code - 3 character
**85**	Pseudonymised provider code - 3 character
**86**	Provider code - treatment centre
**87**	Site code of treatment
**88**	Pseudonymised site code of treatment
**89**	Provider type
**90**	Regional office area where patient's GP was registered
**91**	Strategic health authority area where patient's GP was registered
**92**	Broader geographical area where patient's GP was registered
**93**	Census output area 2001
**94**	Census output area 2001 (6 character)
**95**	County of residence
**96**	Local Authority district
**97**	Local authority district & current electoral ward
**98**	Electoral ward in 91
**99**	Government office region of residence
**100**	Government office region of treatment
**101**	Health authority of residence
**102**	Health authority of treatment
**103**	Patient's health authority/PCT of residence provide by NHS
**104**	Patient's primary care trust of residence - historic
**105**	Patient's primary care trust of residence - current
**106**	Patients strategic health authority of residence - historic
**107**	Patients strategic health authority of residence - current
**108**	Primary care trust area of treatment
**109**	Region of treatment
**110**	Regional office of residence
**111**	Strategic health authority area of treatment
**112**	Code of GP practice
**113**	Code of GP practice (Pseudonymised by default)
**114**	Consultant code
**115**	Consultant code (pseudonymised by default)
**116**	Code of patient's registered or referring general medical practitioner
**117**	Code of patient's registered or referring general medical practitioner
**118**	Person referring patient
**119**	Referring organisation Code
**120**	Referring organisation Code (pseudonymised)
**121**	Record Identifier (pseudonymised by default)
**122**	Datayear

************************* **End of sheet**

For peer review only - http://bmjopen.bmj.com/site/about/guidelines.xhtml

For peer review only - http://bmjopen.bmj.com/site/about/guidelines.xhtml

For peer review only - http://bmjopen.bmj.com/site/about/guidelines.xhtml

For peer review only - http://bmjopen.bmj.com/site/about/guidelines.xhtml

Fo

For peer review only - http://bmjopen.bmj.com/site/about/guidelines.xhtml

For peer review only - http://bmjopen.bmj.com/site/about/guidelines.xhtml

Fo

Fo

Fo

For peer review only - http://bmjopen.bmj.com/site/about/guidelines.xhtml

ınd. This data is collected during a patient's time at hospital and is submitted to allow hospitals to be : is use for non-clinical purposes, of this administrative data.

ırked below in red – will require you to have patient consent, section 251 approval, or the ıographic data, event dates and geographies will be treated as de-personalised, unless ymity. Privacy by design and default principles have also been applied to some of these data to ı be selected is included below. **HES data will only be supplied as a linked table to cancer**

ıtlined above)
access
ılighted in dark grey

Field name	Request data item	Justification
**Patient**		
PATIENTID		
category		
admincat		
startage		
**dob**		
Month of birth		
Year of birth		
ethnos		
Option to group ethnicities (e.g. white/ non-white/ unknown)		
**lopatid**		
**newnhsno**		
**encrypted_hesid**		
postdist		
**homeadd**		
Option to provide geography as deprivation score or aggregate to larger geographic areas such as MSOA or county.		
sex		
**Admissions**		
admidate		
Month of admission		
Year of admission		
Option to provide number of days from another event to date of admission (e.g. days from diagnosis to admission)		
elecdate		
Month of decision to admit		
Year of decision to admit		
Option to provide number of days from another event to date of decision to admit (e.g. days from decision to admit to admission)		
admimeth		
admisorc		
firstreg		
elecdur		
classpat		

**Discharges**		
disdate		
Month of discharge		
Year of discharge		
Option to provide number of days from another event to date of discharge (e.g. days from admission to discharge)		
disdest		
dismeth		
**pisodes and Spells**		
bedyear		
spelbgin		
epiend		
Month the episode ended		
Year the episode ended		
Option to provide number of days from another event to date episode ended (e.g. days from diagnosis to date episode ended)		
epistart		
Month the episode started		
Year the episode started		
Option to provide number of days from another event to date episode ended (e.g. days from diagnosis to date episode started)		
speldur		
spelend		
epidur		
epiorder		
epitype		
provspno		
**Clinical**		
diag_nn		
diag3_01		
diag_4		
opertn_nn		
opdate_nn		
Month of operations		
Year of operations		
Option to provide number of days from another event to date of operation (e.g. days from admission to date of operation)		
operstat		
intmanig		
mainspef		
tretspef		
**ncare Resource Groups**		
domproc		
hrg40		
hrgnhs		
hrgnhsvn		
suscorehrg		
sushrg		
sushrgvers		
susspellid		
**Organisation**		
purcode		
purval		

purro		
purstha		
csnum		
gppracha		
pcgcode		
pctcode		
pctcode06		
gpprpct		
procode		
pseudoprocode		
procode3		
pseudoprocode3		
procodet		
sitetret		
pseudositetret		
protype		
gppracro		
gpprstha		
Option to provide a broader geographic area that the patient's GP was registered (e.g. country or country)		
**Geographical**		
oacode		
oacode6		
rescty		
resladst		
resladst_currward		
ward91		
resgor		
gortreat		
resha		
hatreat		
pctnhs		
respct		
respct06		
ressta		
resstha06		
pcttreat		
rotreat		
Resro		
sthatret		
**Practitioner**		
gpprac		
pseudogpprac		
consult		
pseudoconsult		
reggmp		
pseudoreggmp		
referrer		
referorg		
referorg		
**Systems Data**		
epikeyanon		
datayear		

****

For peer review only - http://bmjopen.bmj.com/site/about/guidelines.xhtml

For peer review only

For peer review only

Introduction

Outpatient HES contains data on all outpatient appointments at NHS hospitals in En
care they deliver. HES data is designed to enable secondary use, that is use for nor

Data item selection

Please be aware that selecting patient identifiable data items – **the data items marl**
regulation covering your organisation. Sensitive patient demographic data, event da
data anonymised to k-3 anonymity. Privacy by design and default principles have al
alternative can be selected is included below. **HES data will only be supplied as a**

Key:

**Items in red**	Identifiable fields that require additional permissions to access (as ou
	Sensitive items that require additional justification in your protocol to
	Privacy by design and default' alternatives to the sensitive items high

Item number	Data Item
**1**	Pseudonymised patient ID
**2**	Administrative category
**3**	Ethnic category
**4**	Broad ethnic group
**5**	**Local patient identifier**
**6**	**Patient ID - HES generated**
**7**	Appointment date
**8**	Month of appointment
**9**	Year of appointment
**10**	Days from another event to appointment date
**11**	Attendance identifier
**12**	Attendance type
**13**	Attended or did not attend
**14**	First attendance
**15**	Last DNA or patient cancelled date
**16**	Medical staff type seeing patient
**17**	Outcome of attendance
**18**	Priority type
**19**	Referral request received date
**20**	Service type requested
**21**	Source of referral for outpatients
**22**	Days waiting
**23**	Waiting/waiting calculation indicator also known as waiting quality indicator
**24**	All diagnosis codes
**25**	Primary diagnosis - 4 character
**26**	Primary diagnosis - 3 character (derived)
**27**	All operation codes
**28**	Main operation
**29**	Main operation - 3 character (derived)
**30**	Operation Status code
**31**	Main Specialty
**32**	Treatment Specialty

**33**	NHS generated HRG code
**34**	NHS generated HRG code version number
**35**	SUS generated HRG
**36**	SUS generated HRG version number
**37**	Commissioner code (pseudonymised by default)
**38**	Provider code - treatment
**39**	Pseudonymised provider code
**40**	Provider type
**41**	Patients census output area (2001) (10 character)
**42**	Patients census output area (2001) (6 character)
**43**	County of residence
**44**	Government office region of residence
**45**	Government office region of treatment
**46**	Patients electoral ward in 1991
**47**	Patients Primary Care Trust of residence - current
**48**	Patients Primary Care Trust of residence - historic
**49**	Patients Strategic Health Authority of Residence - current
**50**	Patients Strategic Health Authority of Residence - historic
**51**	Code of GP practice
**52**	Code of GP practice (Pseudonymised by default)
**53**	Consultant Code
**54**	Consultant Code (pseudonymised by default)
**55**	Code of patient's registered or referring general medical practitioner
**56**	Code of patient's registered or referring general medical practitioner (Pseudonymised)
**57**	Person referring patient
**58**	Referring organisation Code
**59**	Pseudonymised referring organisation code
**58**	Record Identifier (pseudonymised by default)
**59**	Datayear

************************** **End of**

For peer review only

For peer review only - http://bmjopen.bmj.com/site/about/guidelines.xhtml

For

For

For

For

For

For

For

For

For r

For r

For peer review only - http://bmjopen.bmj.com/site/about/guidelines.xhtml

For peer review only

ıgland. This data is collected during a patient's time at hospital and is submitted to allow hospitals to be paid for the ı-clinical purposes, of this administrative data.

ked below in red – will require you to have patient consent, section 251 approval, or the appropriate statutory ates and geographies will be treated as de-personalised, unless anonymisation techniques are applied to render the ılso been applied to some of these data to provide a less disclosive alternative. The key explaining how these **ı linked table to cancer registration data.**

ıtlined above)
access
ılighted in dark grey

Field Name	Request Data Item	Justification
**Patient**		
PATIENTID		
Admincat		
ethnos		
Option to group ethnicities (e.g. white/ non-white/ unknown)		
**lopatid**		
**encrypted_hesid**		
**Appointment**		
apptdate		
Month of appointment		
Year of appointment		
Option to provide number of days from another event to the appt date (e.g. days from diagnosis to appointment)		
attendid		
atentype		
attended		
firstatt		
DNAdate		
stafftyp		
outcome		
priority		
reqdate		
servtype		
refsourc		
waiting		
wait_ind		
**Clinical**		
diag_nn		
diag_4		
diag3		
opertn_nn		
opertn_01		
opertn3		
operstat		
mainspef		
tretspef		
**Healthcare Resource Groups**		

hrgnhs		
hrgnhsvn		
sushrg		
sushrgvers		Available from 2009/10
**Organisation**		
purcode		
procodet		
procodet (pseudonymised)		
protype		
**Geographical**		
oacode01		
oacode6		
rescty		
resgor		
gortreat		
ward91		
respct06		
respct		
resstha06		
resstha		
**Practitioner**		
gpprac		
pseudogpprac		
consult		
pseudoconsult		
reggmp		
pseudoreggmp		
referrer		
referorg		
referorg (pseudonymised)		
**Systems Data**		
attendkeyanon		
datayear		

[illegible] **sheet** *************************

ew only

For peer review only - http://bmjopen.bmj.com/site/about/guidelines.xhtml

ew only

For peer review only

For peer review only

For peer review only

For peer review only

For peer review only

For peer review only

For peer review only

For peer review only

For peer review only

For peer review only

For peer review only

For peer review only

For peer review only

For peer review only

For peer review only

Introduction

Accident and Emergency HES contains data on all A&E attendances at NHS hc
for the care they deliver. HES data is designed to enable secondary use, that is

Data items selection

Please be aware that selecting patient identifiable data items – **the data items**
regulation covering your organisation. Sensitive patient demographic data, eve
data anonymised to k-3 anonymity. Privacy by design and default principles ha
alternative can be selected is included below. **HES data will only be supplied**

Key:

**Items in red**	Identifiable fields that require additional permissions to access
	Sensitive items that require additional justification in your proto
	Privacy by design and default' alternatives to the sensitive item

Item number	Data item
1	Pseudonymised patient ID
2	Ethnic category
3	**Local patient identifier**
4	**Patient ID - HES generated**
5	Arrival mode
6	Attendance category
7	Attendance disposal
8	Department type
9	Duration to assessment
10	Duration to treatment
11	Duration to conclusion
12	Duration to departure
13	Incident location type
14	Patient group
15	Source of referral
16	Arrival date
17	Day of the week of the arrival
18	Arrival on a: weekday / weekend
19	Days from arrival date to another event
20	Arrival time
21	Arrival time occurring in the: morning / afternoon / evening
22	A&E diagnosis
23	A&E diagnosis - 2 character
24	A&E diagnosis - Anatomical area
25	A&E diagnosis - Anatomical side
26	A&E investigation
27	A&E investigation - 2 character
28	A&E treatment
29	A&E treatment - 2 character

**30**	2001 Census output area
**31**	2001 Census output area (6 character)
**32**	County of residence
**33**	Current electoral ward
**34**	Current PCT of residence
**35**	Current SHA of residence
**36**	Government Office Region of residence
**37**	Health authority of residence
**38**	Historic PCT of residence
**39**	Historic SHA of residence
**40**	LA district of residence
**41**	Region of residence
**42**	Government Office Region of treatment
**43**	Health Authority of treatment
**44**	PCT of treatment
**45**	Region of treatment
**46**	SHA of treatment
**47**	Dominant procedure
**48**	Trust derived HRG value
**49**	Version no. of trust derived HRG
**50**	SUS generated HRG (available 2009-2010)
**51**	SUS generated HRG version number (Available 2009 - 2010)
**52**	Provider code 3 - character
**53**	Provider code 3 - character (pseudonymised)
**54**	Provider code 5 - character
**55**	Pseudonymised Provider code 5 - character
**56**	Provider code - treatment
**57**	Pseudonymised treatment provider code
**58**	Provider type
**59**	Org code of patient path ID issuer
**60**	RTT period start
**61**	RTT period status
**62**	RTTP period end
**63**	Duration of wait (referral to treatment period)
**64**	GP practice code
**65**	GP practice code (pseuondymised by default)
**66**	Record Identifier (pseudonymised by default)
**67**	Datayear

************************* **End of sheet** *************************

For r

For p

For r

For r

For r

For r

For r

For p

For r

For r

For r

For r

For r

ospitals in England. This data is collected during a patient's time at hospital and is submitted to allow hospitals to be paid
s use for non-clinical purposes, of this administrative data.

marked below in red – will require you to have patient consent, section 251 approval, or the appropriate statutory
ent dates and geographies will be treated as de-personalised, unless anonymisation techniques are applied to render the
ave also been applied to some of these data to provide a less disclosive alternative. The key explaining how these
as a linked table to cancer registration data.

(as outlined above)
ocol to access
ns highlighted in dark grey

Field name	Request data item	Justification
**Patient**		
PATIENTID		
ethnos		
**lopatid**		
**encrypted_hesid**		
**Attendances**		
aearrivalmode		
aeattendcat		
aeattenddisp		
aedepttype		
initdur		
tretdur		
concldur		
depdur		
aeincloctype		
aepatgroup		
aerefsource		
arrivaldate		
Option to provide the day of the week the A&E arrival took place		
Option to provide whether the A&E arrival was on a weekday or at the weekend		
Option to provide number of days from arrival to another event (e.g. days from A&E arrival to diagnosis)		
arrivaltime		
Option to provide the part of the day the patient arrived		
**Clinical diagnosis**		
diag_n		
diag2_n		
diaga_n		
diags_n		
**Clinical Investigation**		
invest_n		
invest2_n		
**Clinical treatment**		
treat_n		
treat2_n		
**Residence**		

For peer review only

oacode		
oacode6		
rescty		
currward		
respct06		
resstha06		
resgor		
resha		
respct02		
resstha02		
resladst		
resro		
**Treatment**		
gortreat		
hatreat		
pcttreat		
rotreat		
sthatret		
**HRG data**		
domproc		
hrgnhs		
hrgnhsvn		
sushrg		
sushrgvers		
**Organisation data**		
procode3		
procode3 (pseudonymised)		
procode		
procode (pseudonymised)		
procodet		
procodet (pseudonymised)		
protype		
**Patient Pathway**		
orgpppid		
rttperstart		
rttperstat		
rttperend		
waitdays		
**Practitioner Data**		
gpprac		
gpprac (pseudonymised)		
**System Data**		
aekeyanon		
datayear		

For peer review only - http://bmjopen.bmj.com/site/about/guidelines.xhtml

For peer review only - http://bmjopen.bmj.com/site/about/guidelines.xhtml

For peer review only - http://bmjopen.bmj.com/site/about/guidelines.xhtml

For peer review only

For peer review only

For peer review only

For peer review only

For peer review only

For peer review only

For peer review only

For peer review only

For peer review only

For peer review only

For peer review only

For peer review only

For peer review only

For peer review only

For peer review only

Introduction

Routes to Diagnosis: cancer registration data are combined with Administrative Hospital Episo cancer screening programmes. Using these datasets cancers registered in England which wer Routes to Diagnosis. The methodology is described in detail in the British Journal of Cancer ar journey using multiple routine datasets'.

Data items selection

Please select your required data items from the list below. If you require more details on the m http://www.ncin.org.uk/publications/routes_to_diagnosis or http://www.nature.com/bjc/journal/v

Please be aware that selecting patient identifiable data items – **the data items marked below** approval, or the appropriate statutory regulation covering your organisation. Sensitive patient as de-personalised, unless anonymisation techniques are applied to render the data anonymis

Item number	Data item	Field name
		**Route to diagnosis**
**1**	Tumour level pseudo ID (for linkage)	TUMOURID
**2**	Route to diagnosis code (the code assigned to a route for the purpose of the algorithm)	ROUTE_CODE
**3**	Finalised route to diagnosis (the published route with all datasets types accounted for)	FINAL_ROUTE

************************* **End of sheet** *************************

de Statistics data, Cancer Waiting Times data and data from the
e diagnosed in 2006 to 2015 are categorised into one of eight
ticle 'Routes to Diagnosis for cancer - Determining the patient

neaning and definition of specific data items please visit:
107/n8/full/bjc2012408a.html

in red – will require you to have patient consent, section 251
demographic data, event dates and geographies will be treated
sed to k-3 anonymity.

Request data item	Justification

Introduction

Systemic Anti-Cancer Therapy Data Set collects clinical management on patien
acute inpatient, daycase, outpatient settings and delivery in the community. It cov
designed to collect information on all drug treatments with an anti-cancer effect, in

Data validation is an important aspect of the programme. This is in three stages co
and validity of their data before inclusion for subsequent analysis.

Coverage

Data is available from April 2012 to September 2016 inclusive. The data is linked a

Data item selection

Please be aware that selecting patient identifiable data items – **the data items ma**
your organisation. Sensitive patient demographic data, event dates and geographi
anonymity. Privacy by design and default principles have also been applied to son

Key:

Items in red Identifiable fields that require additional permissions to access (as c
Sensitive items that require additional justification in your protocol to
Privacy by design and default' alternatives to the sensitive items hig

Item number	Data item
1	Pseudonymised patient ID
2	**NHS number**
3	NHS number status indicator code
4	**Date of birth**
5	Month of birth
6	Year of birth
7	Gender code (current)
8	Ethnicity
9	Broad ethnic group
10	Postcode
11	Broader geographic area/ IMD quintile
12	General medical practice code (patient registration)
13	**Consultant code (initiated SACT)**
14	Consultant code (pseudonymised)
15	Care professional main speciality code (start SACT)
16	Organisation code
17	Organisation code (pseudonymised)
18	Primary diagnosis (on SACT initiation)
19	Morphology (ICD-O on SACT initiation)
20	Pre- treatment (final) TNM stage
21	SACT programme number
22	Anti-cancer regimen number
23	Drug treatment intent
24	Regimen analysis grouping
25	Regimen grouping (benchmark reports)
26	Patient's height (metres (m))
27	Patient's weight (kilograms (kg))
28	Performance Status (Adult)
29	Performance Status (Young Person)
30	Co-morbidity adjustment indicator

31	Decision to treat date (Drug regimen)
32	Month of decision to treat (Drug regimen)
33	Year of decision to treat (Drug regimen)
34	Days from another event to decision to treat date
35	Start date (Drug regimen)
36	Month of start date for drug regimen
37	Year of start date for drug regimen
38	Days from another event to drug regimen start date
39	Clinical trial indicator
40	Chemo-radiation indicator
41	Number of planned systemic anti-cancer therapy cycles
41	Cycle identifier
42	Start date (Cycle)
43	Month of start date of cycle
44	Year of start date of cycle
45	Days from another event to start date of cycle
46	Patient's Weight (Kilograms (kg))
47	Performance Status (Adult)
48	Performance Status (Young Person)
49	Primary procedure (OPCS)
50	Drug analysis grouping
51	Actual dose
52	SACT drug route of administration
53	SACT administration date
54	Organisation code (provider)
55	Pseudonymised organisation code (provider)
56	Primary procedure (OPCS)
57	Start date (Final therapy)
58	Month of final therapy
59	Year of final therapy
60	Days from another event to start date of final therapy
61	Regimen modification indicator (dose reduction)
62	Regimen modification indicator (time delay)
63	Regimen modification indicator (days reduced)
64	Planned treatment change reason

************************* End of sheet *************************

ıts receiving cancer chemotherapy in or funded by the NHS in England. It relates to all canc
/ers chemotherapy treatment for all solid tumour and haematological malignancies and those
ı all treatment settings, including traditional cytotoxic chemotherapy and all newer agents.

overing data quality, confirmation of activity levels and an initial clinical analysis. Trusts are r

at a patient level and can be linked to the latest available Cancer Reg- Patient Table

arked below in red – will require you to have patient consent, section 251 approval, or the a
ıies will be treated as de-personalised, unless anonymisation techniques are applied to rend
ne of these data to provide a less disclosive alternative. The key explaining how these altern

outlined above)
o access
ghlighted in dark grey

Field name	Request data item
**Demographics and consultant**	
PATIENTID	
**NHS_Number**	X
NHS_Number_Status	
**Date_Of_Birth**	X
MONTH_DOB	
YEAR_DOB	
Gender_Current	X
Ethnicity	X
Option to group ethnicities (e.g. white/ non-white/ unknown)	
Postcode	X
Option to provide geography as deprivation score or aggregate to larger geographic areas such as MSOA or county.	X
GP_Practice_Code	
**Consultant_GMC_Code_Clean**	
Consultant_GMC_Code (pseudonymised)	
Consultant_Speciality_Code	
Organisation_Code_of_Provider	
Organisation_Code_of_Provider (pseudonymised)	
**Clinical status**	
Primary_Diagnosis	X
Morphology_clean	X
Stage_at_Start	X
**Programme and regimen**	
Programme_Number	X
Regimen_Number	X
Intent_of_Treatment	X
Analysis_Group	X
Benchmark_Group	X
Height_At_Start_of_Regimen	X
Weight_At_Start_of_Regimen	X
Performance_Status_at_Start_of_Regimen_Clean	X
Performance_Status_at_Start_of_Regimen_Clean	X
Comorbidity_Adjustment	X

Date_Decision_To_Treat	X
Month of decision to treat	X
Year of decision to treat	X
Option to provide number of days from another event to the date of the decision to treat (e.g. days from diagnosis to date of decision to treat)	X
Start_Date_of_Regimen	X
Month of start date of drug regimen	X
Year of start date of drug regimen	X
Option to provide number of days from another event to the start date of the drug regimen (e.g. days from date of decision to treat to start date of regimen)	X
Clinical_Trial	X
Chemo_Radiation	X
Number_of_Cycles_Planned	X
**Cycle**	
Cycle_Number	X
Start_Date_of_Cycle	X
Month of start date of cycle	X
Year of start date of cycle	X
Option to provide number of days from another event to the start date of the cycle (e.g. days from diagnosis to start date of cycle)	X
Weight_At_Start_Of_Cycle	X
Performance_Status_At_Start_Of_Cycle_Clean	X
Performance_Status_At_Start_Of_Cycle_Clean	X
OPCS_Procurement_Code	X
**Drug details**	
Drug_Group	X
Actual_Dose_Per_Administration	X
Administration_Route	
Administration_Date	X
Organisation_Code_of_Drug_Provider	
Organisation_Code_of_Drug_Provider (pseudonymised)	
OPCS_Delivery_Code	
**Outcome**	
Date_of_Final_Treatment	X
Month of final therapy	X
Year of final therapy	X
Option to provide number of days from another event to the start date of the final therapy (e.g. days from diagnosis to start date of final therapy)	X
Regimen_Modification_Dose_Reduction	X
Regimen_Modification_Time_Delay	X
Regimen_Modification_Stopped_Early	X
Regimen_Outcome_Summary	X

For peer review only - http://bmjopen.bmj.com/site/about/guidelines.xhtml

ːer patients, both adult and paediatric, in
e in clinical trials. The dataset has been

required to check the quality, quantity

appropriate statutory regulation covering
er the data anonymised to k-3
native can be selected is included below:

Justification
To better understand cancer morphology and related treatment
To better understand cancer morphology and related treatment
To better understand cancer morphology and related treatment
To better understand cancer morphology and related treatment
To better understand cancer morphology and related treatment
To better understand cancer morphology and related treatment
To better understand cancer morphology and related treatment
To better understand cancer morphology and related treatment
To better understand cancer morphology and related treatment
To better understand cancer morphology and related treatment

To better understand cancer morphology and related treatment
To better understand cancer morphology and related treatment (if above not available)
To better understand cancer morphology and related treatment (if above not available)
Days from diagnosis to date of decision to treat
To understand cancer treatment
To understand cancer treatment
To understand cancer treatment
To understand cancer treatment
To understand cancer treatment
To understand cancer treatment
To understand cancer treatment
To understand cancer treatment
To understand cancer treatment
To understand cancer treatment
To understand cancer treatment
To understand cancer treatment
To understand cancer treatment
To understand cancer treatment
To understand cancer treatment
To understand cancer treatment
To understand cancer treatment
To understand cancer treatment
To understand cancer treatment
To understand cancer treatment

For peer review only

For peer review only

peer review only

For peer review only

For peer review only

Introduction

A **Radiotherapy** EPISODE is a continuous period of care for radic
Treatment given concurrently or consecutively to multiple anatomi
If the treatment plan changes during treatment (e.g.: because a ne
Treatment given to separate unrelated PRIMARY tumour sites will

Inclusions:

All radiotherapy of the following types:

• Teletherapy
• Brachytherapy given using automated remote afterloading machi
• All other brachytherapy given for the treatment of malignant dise
delivered in England to patients in NHS facilities, or in private facil

Exclusions:

• Brachytherapy delivered using other than automated remote afte
• Radiotherapy delivered using unsealed sources
• Radiotherapy delivered outside of England
• Radiotherapy delivered in a non-NHS setting and not funded by t
• Non-therapeutic exposures delivered using a radiotherapy machi
• Non-patient exposures (e.g. dosimetry exposures, blood or tissu

Coverage

Data is available from 01/04/2009 – 31/03/2016. The data is linkec

Data item selection

Please be aware that selecting patient identifiable data items – **the**
demographic data, event dates and geographies will be treated as
these data to provide a less disclosive alternative. The key explair

Key:

**Items in red**	Identifiable fields that require additional permissions
	Sensitive items that require additional justification ir
	Privacy by design and default' alternatives to the se

Item number	Name
1	Pseudonymised patient ID
2	ATTENDANCE IDENTIFIER (Pseudonymised)
3	ORGANISATION CODE (LOCAL PATIENT IDENTIFIER)
4	PATIENT ID (Pseudonymised)
	A Radiotherapy EPISODE is a co<br>Treatment given con<br>If the treatment plan changes during treatment
5	RADIOTHERAPY EPISODE IDENTIFIER (Pseudonymised)
6	APPOINTMENT DATE
7	Month of appointment
8	Year of appointment
9	Days from another event to appointment date

**10**	DECISION TO TREAT DATE (RADIOTHERAPY TREATMENT EPISODE)
**11**	Month of decision to treat date
**12**	Year of decision to treat date
**13**	Days from another event to decision to treat date
**14**	EARLIEST CLINICALLY APPROPRIATE DATE
**15**	Month of earliest clinically appropriate date
**16**	Year of earliest clinically appropriate date
**17**	Days from another event to decision to earliest clinically appropriate date
**18**	RADIOTHERAPY PRIORITY
**19**	TREATMENT START DATE (RADIOTHERAPY TREATMENT EPISODE)
**20**	Month of treatment start date
**21**	Year of treatment start date
**22**	Days from another event to treatment start date
**23**	RADIOTHERAPY DIAGNOSIS (ICD)
**24**	RADIOTHERAPY INTENT
	A radiotl
**25**	PRESCRIPTION IDENTIFIER (Pseudonymised)
**26**	RADIOTHERAPY TREATMENT REGION
**27**	ANATOMICAL TREATMENT SITE (RADIOTHERAPY)
**28**	NUMBER OF TELETHERAPY FIELDS
**29**	RADIOTHERAPY PRESCRIBED DOSE
**30**	PRESCRIBED FRACTIONS
**31**	RADIOTHERAPY ACTUAL DOSE
**32**	ACTUAL FRACTIONS
**33**	RADIOTHERAPY TREATMENT MODALITY
**34**	RADIOTHERAPY FIELD IDENTIFIER (Pseudonymised)

For peer review only

**35**	MACHINE IDENTIFIER
**36**	MACHINE IDENTIFIER (pseudonymised by default)
**37**	RADIOISOTOPE
**38**	RADIOTHERAPY BEAM TYPE
**39**	RADIOTHERAPY BEAM ENERGY
**40**	TIME OF EXPOSURE
**41**	ORGANISATION CODE (CODE OF PROVIDER)
**42**	ORGANISATION CODE (CODE OF PROVIDER) - pseudonymised by default
**43**	PROCEDURE (OPCS)
**44**	PROCEDURE DATE
**45**	Month of procedure
**46**	Year of procedure
**47**	Days from another event to procedure date

************************* **End of sheet** *************************

otherapy (from start to finish) including all preparation, planning and delivery of tre
cal sites associated with the same primary tumour will form part of the same radi
ew symptom arises which also requires radiotherapy) then all of the treatment del
l form separate episodes.

ines
ase
ities where delivery is funded by the NHS, from 1st April 2016.

erloading, for the treatment of non-malignant disease

the NHS
ines (e.g.: imaging)
es, animals)

d at a patient level and can be linked to the latest available Cancer Reg- Patient T

e data items marked below in red – will require you to have patient consent, se
s de-personalised, unless anonymisation techniques are applied to render the dat
ning how these alternative can be selected is included below:

s to access (as outlined above)
n your protocol to access
ensitive items highlighted in dark grey

Description
**RTDS LINKAGE**<br>**Patient information**<br>The following data items are required for patient link
Project specific patient ID
A sequential number or time of day used to enable an attendance to be uniquely identified.
This is the ORGANISATION CODE of the ORGANISATION that assigned the LOCAL PATIENT IDENTIFIER
Patient ID from AV_TUMOUR on CAS
**RADIOTHERAPY EPISODE**<br>To carry the details of the Episode of radiotherapy being giv<br>ntinuous period of care for radiotherapy (from start to finish) including all prepara<br>ncurrently or consecutively to multiple anatomical sites associated with the same<br>(e.g.: because a new symptom arises which also requires radiotherapy) then all<br>Treatment given to separate unrelated PRIMARY tumour sites w
Any identifier that is unique for each radiotherapy episode.
Date when PATIENT is to be seen by or be in contact with one or more CARE PROFESSIONALS.
Derived from APPOINTMENT DATE field
Derived from APPOINTMENT DATE field
Derived from APPOINTMENT DATE field

only

The date on which it was decided that the PATIENT required a specific Planned Cancer Treatment. This is the date that the consultation between the PATIENT and the clinician took place and a Planned Cancer Treatment was agreed.
Derived from DECISION TO TREAT DATE field
Derived from DECISION TO TREAT DATE field
Derived from DECISION TO TREAT DATE field
This is the first date that the patient would have been available to start radiotherapy.
Derived from EARLIEST CLINICALLY APPROPRIATE DATE field
Derived from EARLIEST CLINICALLY APPROPRIATE DATE field
Derived from EARLIEST CLINICALLY APPROPRIATE DATE field
The priority for this course of therapy as classified by the requesting clinician.
The start of a stay, an episode, period covered by a plan or other time period. This may be used to calculate the length of the period, or to classify by financial year or other time-based criterion.
Derived from TREATMENT START DATE field
Derived from TREATMENT START DATE field
Derived from TREATMENT START DATE field
This is the PATIENT DIAGNOSIS for:<br>• Patients with cancer, the primary tumour diagnosis code or<br>• non-cancer diagnoses, the main condition being treated during the episode of radiotherapy<br>Note: The definition of this field is different from that of the Primary Diagnosis in CDS.
The intent of the delivered beam radiation.
**RADIOTHERAPY PRESCRIPTION**<br>**To carry the details of each Prescription of radiotherapy bein**<br>herapy prescription specifies a radiotherapy field arrangement, dose and fractiona
Any identifier that is unique for each radiotherapy prescription.
The specific area to be treated with radiotherapy.
The part of the body to which the RADIOTHERAPY ACTUAL DOSE is administered.
The prescribed number of fields of a Teletherapy Treatment Course.
The total prescribed absorbed radiation dose in Grays
The prescribed number of Fractions or hyperfractionation of a Teletherapy Treatment Course
The total actual absorbed radiation dose given in Grays. *This item may be omitted from all but the ultimate fraction for this prescription.*
The total number of Fractions or hyperfractionation of a Teletherapy Treatment Course administered. *This item may be omitted from all but the ultimate fraction for this prescription.*
The type of treatment delivered during a RADIOTHERAPY PRESCRIPTION.
**RADIOTHERAPY EXPOSURE**<br>**To carry the details of each radiotherapy exposure delive**<br>A Radiotherapy Exposure is the delivery of one treatment field as pa
An identifier that is unique for each radiotherapy exposure.

For peer review only

A unique code ascribed to the radiotherapy equipment used to treat this exposure.<br>This identifier is made up of:<br>Five character NACS site code (R----)<br>Two character equipment type code (LA/CO/KV/OT)<br>Four digit unique sequence number (issued by RTDS).
A pseudonymised code ascribed to the radiotherapy equipment used to treat this exposure.<br>This identifier is made up of:<br>Five character NACS site code (R----)<br>Two character equipment type code (LA/CO/KV/OT)<br>Four digit unique sequence number (issued by RTDS).
The type of radioactive source used to deliver radiotherapy with brachytherapy. To record the isotope in standard scientific notation (e.g.: I123 or Ir192)
The prescribed type of beam of a Teletherapy Treatment Course.
Beam energy in MeV/MV/MVp. Record kVp energies as decimals (e.g. 250kV = 0.25MV). Only for multi-modality machines.
Time when the exposure was initiated
**OPCDS PROCEDURES**
This is the ORGANISATION CODE of the ORGANISATION acting as a Health Care Provider.
This is a pseudonymised ORGANISATION CODE of the ORGANISATION acting as a Health Care Provider.
**Start of repeating Item**
Procedure carried out and recorded for CDS or HES purposes.
The date of the occurrence of the CLINICAL INTERVENTION.
Derived from PROCEDURE DATE field
Derived from PROCEDURE DATE field
Derived from PROCEDURE DATE field
**End of repeating item**

eatment as covered in the treatment intention.
iotherapy episode.
livered concurrently or consecutively will form part of the same episode.

Table

ection 251 approval, or the appropriate statutory regulation covering your organisation. Sensitive patient
ta anonymised to k-3 anonymity. Privacy by design and default principles have also been applied to some of

Variable name	Request Data Item	Justification
kage purposes.		
PATIENTID		
OPCDS_LINKAGE.ATTENDID		
OPCDS_LINKAGE.ORGCODEPROVIDER		
OPCDS_LINKAGE.PATIENTID		
ven at this attendance.<br>tion, planning and delivery of treatment as covered in the treatment intention.<br>primary tumour will form part of the same radiotherapy episode.<br>of the treatment delivered concurrently or consecutively will form part of the same episode.<br>will form separate episodes.		
RTDS_EPISODES.RADIOTHERAPYEPISODEID	X	
RTDS_EPISODES.APPTDATE	X	
Month of appointment	X	
Year of appointment	X	
Option to provide number of days from another event to the appointment date (e.g. days from diagnosis to appointment date)	X	Days from diagnosis to appointment date

RTDS_EPISODES.DECISIONTOTREATDATE	X	
Month of decision to treat date	X	
Year of decision to treat date	X	
Option to provide number of days from another event to the d		Days from diagnosis to decision to treat
RTDS_EPISODES.EARLIESTCLINAPPROPRIATEDATE		
Month of earliest clinically appropriate date		
Year of earliest clinically appropriate date		
Option to provide number of days from another event to the earliest clinically appropriate date (e.g. days from diagnosis to earliest clinically appropriate date)		
RTDS_EPISODES.RADIOTHERAPYPRIORITY	X	To better understand cancer treatment
RTDS_EPISODES.TREATMENTSTARTDATE	X	To better understand cancer treatment
Month of treatment start date	X	To better understand cancer treatment
Year of treatment start date	X	To better understand cancer treatment
Option to provide number of days from another event to the treatment date (e.g. days from diagnosis to treatment start date)	X	To better understand cancer treatment
RTDS_EPISODES.RADIOTHERAPYDIAGNOSISID	X	To better understand cancer treatment
RTDS_EPISODES.RADIOTHERAPYINTENT	X	To better understand cancer treatment
g given at this attendance.<br>ation for treatment to an 'ICRU-like' reference point.		
RTDS_PRESCRIPTIONS.PRESCRIPTIONID	X	To better understand cancer treatment
RTDS_PRESCRIPTIONS.RTTREATMENTREGION	X	To better understand cancer treatment
RTDS_PRESCRIPTIONS.RTTREATMENTANATOMICALSITE	X	To better understand cancer treatment
RTDS_PRESCRIPTIONS.NUMBEROFTELETHERAPYFIELDS	X	To better understand cancer treatment
RTDS_PRESCRIPTIONS.RTPRESCRIBEDDOSE	X	To better understand cancer treatment
RTDS_PRESCRIPTIONS.PRESCRIBEDFRACTIONS	X	To better understand cancer treatment
RTDS_PRESCRIPTIONS.RTACTUALDOSE	X	To better understand cancer treatment
RTDS_PRESCRIPTIONS.ACTUALFRACTIONS	X	To better understand cancer treatment
RTDS_PRESCRIPTIONS.RTTREATMENTMODALITY	X	To better understand cancer treatment
ered at this attendance.<br>art of a Fraction of Radiotherapy		
RTDS_EXPOSURES.RADIOTHERAPYFIELDID		

RTDS_EXPOSURES.MACHINEID		
RTDS_EXPOSURES.MACHINEID (pseudonymised)		
RTDS_EXPOSURES.RADIOISOTOPE		
RTDS_EXPOSURES.RADIOTHERAPYBEAMTYPE		
RTDS_EXPOSURES.RADIOTHERAPYBEAMENERGY		
RTDS_EXPOSURES.TIMEOFEXPOSURE		
OPCDS_PROCEDURES.ORGCODEPROVIDER		
OPCDS_PROCEDURES.ORGCODEPROVIDER (pseudonymised)		
OPCDS_PROCEDURES.PRIMARYPROCEDUREOPCS	X	To better understand cancer treatment
OPCDS_PROCEDURES.PROCEDUREDATE		
Month of procedure		
Year of procedure		
Option to provide number of days from another event to the treatment date (e.g. days from diagnosis to procedure date)		

Introduction:

The National Cancer Patient Experience Survey (CPES), commissioned by NHS England through Quality Health i
Trust in England providing adult cancer services as inpatients or day cases, and discharged within a specified thre
through diagnosis and treatment and to the ongoing management of their cancer.

Data coverage:
Wave 1 - 2010: Patients discharged between 01/01/10 – 31/03/10
Wave 2 - 2011/12: Patients discharged between 01/09/11 – 30/11/11
Wave 3 - 2013: Patients discharged between 01/09/12 – 30/09/12
Wave 4 - 2014: Patients discharged between 01/09/13 – 30/11/13
Wave 5 - 2015: Patients discharged between 01/04/2015 - 30/06/2015

Data items selection:

Please be aware that selecting patient identifiable data items – **the data items marked below in red** – will requir
event dates and geographies will be treated as de-personalised, unless anonymisation techniques are applied to r
alternative. The key explaining how these alternative can be selected is included below:

Key:

**Items in red**	Identifiable fields that require additional permissions to access (as outlined above)
	Sensitive items that require additional justification in your protocol to access
	Privacy by design and default' alternatives to the sensitive items highlighted in dark grey

	**W**<br>Pati
**Item number**	**Data item**
**1**	Pseudonymised patient ID
**2**	Pseudonymised tumourID
**3**	How many times did you see your GP about the health problem caused by cancer
**4**	How long did you have to wait before your first appointment after your GP told you that you would need to see a hospital doctor
**5**	How do you feel about the length of time you had to wait before your first appointment with a hospital doctor
**6**	How long did you have to wait before your first appointment with a hospital doctor
**7**	Did your health get worse, better or stay the same while you were waiting for first appointment
**8**	In the last 12 months have you had diagnostics tests
**9**	Did staff give complete explanation of purpose of test(s)
**10**	Did staff explain completely what would be done during test
**11**	Were you given easy to understand written information about test
**12**	Were you given complete explanation of test results in understandable way
**13**	Who first told you that you had cancer
**14**	Were you told they could bring a friend when first told they had cancer
**15**	How do you feel about the way you were told that you had cancer
**16**	Did you understand the explanation of what was wrong with you
**17**	Were you given written information about the type of cancer you had

18	Before your cancer treatment started, were you given a choice of different types of treatment
19	Were the possible side effects of treatments explained in a way you could understand
20	Were you given written information about the side effects of treatments
21	Were you involved as much as you wanted to be in decisions about your care and treatment
22	Were you given the name of the CNS in charge of your care
23	How easy is it for you to contact your CNS
24	Did your CNS listen carefully to you the last time you spoke
25	How often do you get understandable answers from you CNS
26	The last time you saw or spoke to your CNS, how do you feel about the time you spent with them
27	Did hospital staff give information about support/self-help groups
28	Did hospital staff give information about financial help or any benefits you might be entitled to
29	Did hospital staff tell you that you could get free prescriptions
30	Have you had an operation in the last 12 months
31	Was your admission date changed to a later date by the hospital
32	Did staff give complete explanation of what would be done
33	Were you given written information about the operation
34	Did staff explain how operation had gone in understandable way
35	Have you had an operation or stayed overnight for cancer care
36	When you had important questions how often did you get understandable answers
37	Did you have confidence and trust in the doctors treating you
38	Do you think the doctors treating you knew enough about how to treat your cancer
39	Did doctors talk in front of you as if you weren't there
40	Did your friends and family have enough opportunity to talk to a doctor if they wanted to
41	When you had important questions to ask a ward nurse how often did you get understandable answers
42	Did you have confidence and trust in the ward nurses treating you
43	Did ward nurses talk in front of you as if you weren't there
44	In your opinion were there enough nurses on duty to care for you in hospital
45	Did you ever think that doctors or nurses were deliberately not telling you certain things you wanted to know
46	Did you ever get conflicting information from your treatment team
47	Were you given enough privacy when discussing your condition or treatment
48	Were you given enough privacy when being examined or treated
49	Do you think the hospital staff did everything they could to help control your pain
50	Were you treated with respect and dignity by hospital staff
	Inf
51	Were you given clear written information about what you should and should not do after you left hospital
52	Did staff tell you who to contact if you were worried about your condition or treatment
53	Were your family given all the information they needed to help care for you at home

For peer review only

54	After leaving hospital were you given enough care and help from health and social services
55	During the past 12 months have you had radiotherapy
56	Did hospital staff do everything possible to control the side effects of radiotherapy
57	During the past 12 months have you had chemotherapy
58	Did hospital staff do everything possible to control the side effects of chemotherapy
59	As an OP or day case did staff do everything they could to help control your pain
60	As an OP or day case were you given enough emotional support from staff
61	In the last 12 months have you had an OP appointment with a cancer doctor
62	How long after your appointed time did your OP appointment start
63	How was the length of your appointment with your cancer doctor
64	Did the doctor have the correct documents
65	Was your GP given enough information about your condition and treatment you had at the hospital
66	Did the practice staff do everything they could to support you while you were having cancer treatment
67	Did the different people treating and caring for you work well together to give you the best possible care
68	How much information were you given about your condition and treatment
69	Did you ever feel in your NHS care over the last year like a 'set of cancer symptoms'
70a	What year were you born
70b	Age bands
71	Gender
72a-g	Do you suffer from any of the following long-standing conditions
73	How long is it since you were treated for this cancer
74	**Sexual Orientation**
75	Can we send you a survey in the future to ask about your health and healthcare
76a	Ethnic Group
76b	Broader ethnic group

	Wa[illegible]<br>Pati[illegible]
**Item number**	**Data item**
1	Pseudonymised patient ID
2	Pseudonymised tumourID
3	How many times did you see your GP about the health problem caused by cancer
4	How do you feel about the length of time you had to wait before your first appointment with a hospital doctor
5	How long did you have to wait before your first appointment with a hospital doctor

6	Did your health get worse, better or stay the same while you were waiting for first appointment
7	In the last 12 months have you had diagnostics tests
8	Did staff give complete explanation of purpose of test(s)
9	Did staff explain completely what would be done during test
10	Were you given easy to understand written information about test
11	Were you given complete explanation of test results in understandable way
12	Who first told you that you had cancer
13	Were you told they could bring a friend when first told they had cancer
14	How do you feel about the way you were told that you had cancer
15	Did you understand the explanation of what was wrong with you
16	Were you given written information about the type of cancer you had
17	Before your cancer treatment started, were you given a choice of different types of treatment
18	Do you think you were taken into account when teams were discussing which treatment you should have
19	Were the possible side effects of treatments explained in a way you could understand
20	Were you given written information about the side effects of treatments
21	Were you involved as much as you wanted to be in decisions about your care and treatment
22	Were you given the name of the CNS in charge of your care
23	How easy is it for you to contact your CNS
24	Did your CNS listen carefully to you the last time you spoke
25	How often do you get understandable answers from you CNS
26	Did hospital staff give information about support/self-help groups
27	Did hospital staff give information about financial help or any benefits you might be entitled to
28	Did hospital staff tell you that you could get free prescriptions
29	Taking part in cancer research discussed with patient
30	If yes, were you glad to have been asked
31	If no, would you have liked to have been asked
32	Have you had an operation in the last 12 months
33	Was your admission date changed to a later date by the hospital
34	Did staff give complete explanation of what would be done
35	Were you given written information about the operation
36	Did staff explain how operation had gone in understandable way
37	Have you had an operation or stayed overnight for cancer care

For peer review only

38	When you had important questions how often did you get understandable answers
39	Did you have confidence and trust in the doctors treating you
40	Did doctors talk in front of you as if you weren't there
41	Did your friends and family have enough opportunity to talk to a doctor if they wanted to
42	When you had important questions to ask a ward nurse how often did you get understandable answers
43	Did you have confidence and trust in the ward nurses treating you
44	Did ward nurses talk in front of you as if you weren't there
45	In your opinion were there enough nurses on duty to care for you in hospital
46	Did you ever think that doctors or nurses were deliberately not telling you certain things you wanted to know
47	Did you ever get conflicting information from your treatment team
48	Were you asked what name you prefer to be called by
49	Were you given enough privacy when discussing your condition or treatment
50	Were you given enough privacy when being examined or treated
51	Were you able to discuss any worries or fears with staff during your hospital visit
52	Do you think the hospital staff did everything they could to help control your pain
53	Were you treated with respect and dignity by hospital staff
	Inf
54	Were you given clear written information about what you should and should not do after you left hospital
55	Did staff tell you who to contact if you were worried about your condition or treatment
56	Were your family given all the information they needed to help care for you at home
57	After leaving hospital were you given enough care and help from health and social services
58	Did hospital staff do everything possible to control the side effects of radiotherapy
59	Did hospital staff do everything possible to control the side effects of chemotherapy
60	As an OP or day case did staff do everything they could to help control your pain
61	As an OP or day case were you given enough emotional support from staff
62	In the last 12 months have you had an OP appointment with a cancer doctor
63	How long after your appointed time did your OP appointment start
64	How was the length of your appointment with your cancer doctor
65	Did the doctor have the correct documents
66	Was your GP given enough information about your condition and treatment you had at the hospital
67	Did the practice staff do everything they could to support you while you were having cancer treatment
68	Did the different people treating and caring for you work well together to give you the best possible care
69	How much information were you given about your condition and treatment
70	Have you been offered a written assessment and care plan
71	Did you ever feel in your NHS care over the last year like a 'set of cancer symptoms'
72	Overall, how would you rate you care
73	What year were you born
74	Age bands
75	Gender

For peer review only

**76**	**Sexual Orientation**
**77a-g**	Do you suffer from any of the following long-standing conditions
**78**	How long is it since you were treated for this cancer
**79**	Can we send you a survey in the future to ask about your health and healthcare
**80a**	Ethnic Group
**80b**	Broader ethnic group

	W<br>Pati
**Item number**	**Data item**
**1**	Pseudonymised patient ID
**2**	Pseudonymised tumourID
**3**	How many times did you see your GP about the health problem caused by cancer
**4**	How do you feel about the length of time you had to wait before your first appointment with a hospital doctor
**5**	How long did you have to wait before your first appointment with a hospital doctor
**6**	Did your health get worse, better or stay the same while you were waiting for first appointment
**7**	In the last 12 months have you had diagnostics tests
**8**	Did staff give complete explanation of purpose of test(s)
**9**	Did staff explain completely what would be done during test
**10**	Were you given easy to understand written information about test
**11**	Were you given complete explanation of test results in understandable way
**12**	Who first told you that you had cancer
**13**	Were you told they could bring a friend when first told they had cancer
**14**	How do you feel about the way you were told that you had cancer
**15**	Did you understand the explanation of what was wrong with you
**16**	Were you given written information about the type of cancer you had
**17**	Before your cancer treatment started, were you given a choice of different types of treatment
**18**	Do you think you were taken into account when teams were discussing which treatment you should have
**19**	Were the possible side effects of treatments explained in a way you could understand
**20**	Were you given written information about the side effects of treatments

For peer review only

21	Were you told about side effects that could affect in the future
22	Were you involved as much as you wanted to be in decisions about your care and treatment
23	Were you given the name of the CNS in charge of your care
24	How easy is it for you to contact your CNS
25	Did your CNS listen carefully to you the last time you spoke
26	How often do you get understandable answers from you CNS
27	Did hospital staff give information about support/self-help groups
28	Did hospital staff give information about the impact cancer could have on work life or education
29	Did hospital staff give information about financial help or any benefits you might be entitled to
30	Did hospital staff tell you that you could get free prescriptions
31	Have you seen information about cancer research in the hospital
32	Taking part in cancer research discussed with patient
33	Patient has taken part in cancer research
34	Have you had an operation in the last 12 months
35	Did staff give complete explanation of what would be done
36	Were you given written information about the operation
37	Did staff explain how operation had gone in understandable way
38	Have you had an operation or stayed overnight for cancer care
39	When you had important questions how often did you get understandable answers
40	Did you have confidence and trust in the doctors treating you
41	Did doctors talk in front of you as if you weren't there
42	Did your friends and family have enough opportunity to talk to a doctor if they wanted to
43	When you had important questions to ask a ward nurse how often did you get understandable answers
44	Did you have confidence and trust in the ward nurses treating you
45	Did ward nurses talk in front of you as if you weren't there
46	In your opinion were there enough nurses on duty to care for you in hospital
47	Did you ever think that doctors or nurses were deliberately not telling you certain things you wanted to know
48	Did you ever get conflicting information from your treatment team
49	Were you asked what name you prefer to be called by
50	Were you given enough privacy when discussing your condition or treatment
51	Were you given enough privacy when being examined or treated
52	Were you able to discuss any worries or fears with staff during your hospital visit
53	Do you think the hospital staff did everything they could to help control your pain
54	Were you treated with respect and dignity by hospital staff
55	Were you given clear written information about what you should and should not do after you left hospital
56	Did staff tell you who to contact if you were worried about your condition or treatment
57	Were your family given all the information they needed to help care for you at home
58	After leaving hospital were you given enough care and help from health and social services

For peer review only

59	Did hospital staff do everything possible to control the side effects of radiotherapy
60	Did hospital staff do everything possible to control the side effects of chemotherapy
61	As an OP or day case did staff do everything they could to help control your pain
62	As an OP or day case were you given enough emotional support from staff
63	In the last 12 months have you had an OP appointment with a cancer doctor
64	Did the doctor have the correct documents
65	Was your GP given enough information about your condition and treatment you had at the hospital
66	Did the practice staff do everything they could to support you while you were having cancer treatment
67	Did the different people treating and caring for you work well together to give you the best possible care
68	Have you been treated by a physiotherapist following your cancer
69	Have you been treated by a Occupational Therapist following your cancer
70	Have you been treated by a Dietician following your cancer
71	Have you been treated by a Speech and Language Therapist following your cancer
72	Have you been treated by a Lymphoedema Specialist following your cancer
73	How much information were you given about your condition and treatment
74	Have you been offered a written assessment and care plan
75	Did you ever feel in your NHS care over the last year like a 'set of cancer symptoms'
76	Overall, how would you rate you care
71	What year were you born
72a	Age
72b	Age bands
73	Gender
74	**Sexual Orientation**
75	Employment Status
76	Do you suffer from any of the following long-standing conditions
77	How long is it since you were treated for this cancer
78	How has your cancer responded to treatment
79	Can we send you a survey in the future to ask about your health and healthcare
80a	Ethnic Group
80b	Broader ethnic group

	**W**<br>Pati
**Item number**	**Data item**
1	Pseudonymised patient ID
2	Pseudonymised tumourID

3	How many times did you see your GP about the health problem caused by cancer
4	How do you feel about the length of time you had to wait before your first appointment with a hospital doctor
5	How long did you have to wait before your first appointment with a hospital doctor
6	Did your health get worse, better or stay the same while you were waiting for first appointment
7	In the last 12 months have you had diagnostics tests
8	Did staff give complete explanation of purpose of test(s)
9	Did staff explain completely what would be done during test
10	Were you given easy to understand written information about test
11	Were you given complete explanation of test results in understandable way
12	Who first told you that you had cancer
13	Were you told they could bring a friend when first told they had cancer
14	How do you feel about the way you were told that you had cancer
15	Did you understand the explanation of what was wrong with you
16	Were you given written information about the type of cancer you had
17	Before your cancer treatment started, were you given a choice of different types of treatment
18	Do you think you were taken into account when teams were discussing which treatment you should have
19	Were the possible side effects of treatments explained in a way you could understand
20	Were you given written information about the side effects of treatments
21	Were you told about side effects that could affect in the future
22	Were you involved as much as you wanted to be in decisions about your care and treatment
23	Were you given the name of the CNS in charge of your care
24	How easy is it for you to contact your CNS
25	Did your CNS listen carefully to you the last time you spoke
26	How often do you get understandable answers from you CNS
27	Did hospital staff give information about support/self-help groups
28	Did hospital staff give information about the impact cancer could have on work life or education
29	Did hospital staff give information about financial help or any benefits you might be entitled to
30	Did hospital staff tell you that you could get free prescriptions
31	Have you seen information about cancer research in the hospital
32	Taking part in cancer research discussed with patient
33	Patient has taken part in cancer research

For peer review only

34	Have you had an operation in the last 12 months
35	Did staff give complete explanation of what would be done
36	Were you given written information about the operation
37	Did staff explain how operation had gone in understandable way
38	Have you had an operation or stayed overnight for cancer care
39	When you had important questions how often did you get understandable answers
40	Did you have confidence and trust in the doctors treating you
41	Did doctors talk in front of you as if you weren't there
42	Did your friends and family have enough opportunity to talk to a doctor if they wanted to
43	When you had important questions to ask a ward nurse how often did you get understandable answers
44	Did you have confidence and trust in the ward nurses treating you
45	Did ward nurses talk in front of you as if you weren't there
46	In your opinion were there enough nurses on duty to care for you in hospital
47	Did you ever think that doctors or nurses were deliberately not telling you certain things you wanted to know
48	Did you ever get conflicting information from your treatment team
49	Were you asked what name you prefer to be called by
50	Were you given enough privacy when discussing your condition or treatment
51	Were you given enough privacy when being examined or treated
52	Were you able to discuss any worries or fears with staff during your hospital visit
53	Do you think the hospital staff did everything they could to help control your pain
54	Were you treated with respect and dignity by hospital staff
55	Were you given clear written information about what you should and should not do after you left hospital
56	Did staff tell you who to contact if you were worried about your condition or treatment
57	Were your family given all the information they needed to help care for you at home
58	After leaving hospital were you given enough care and help from health and social services
59	Did hospital staff do everything possible to control the side effects of radiotherapy
60	Did hospital staff do everything possible to control the side effects of chemotherapy
61	As an OP or day case did staff do everything they could to help control your pain
62	As an OP or day case were you given enough emotional support from staff
63	In the last 12 months have you had an OP appointment with a cancer doctor
64	Did the doctor have the correct documents
65	Was your GP given enough information about your condition and treatment you had at the hospital
66	Did the practice staff do everything they could to support you while you were having cancer treatment
67	Did the different people treating and caring for you work well together to give you the best possible care
68	Have you been treated by a physiotherapist following your cancer
69	Have you been treated by a Occupational Therapist following your cancer
70	Have you been treated by a Dietician following your cancer

71	Have you been treated by a Speech and Language Therapist following your cancer
72	Have you been treated by a Lymphoedema Specialist following your cancer
73	How much information were you given about your condition and treatment
74	Have you been offered a written assessment and care plan
75	Did you ever feel in your NHS care over the last year like a 'set of cancer symptoms'
76	Overall, how would you rate you care
77	What year were you born
78a	Age
78b	Age bands
79	Gender
80	**Sexual Orientation**
81	Employment Status
82	Do you suffer from any of the following long-standing conditions
83	How long is it since you were treated for this cancer
84	Is this the first time you have been treated for cancer
85	How has you cancer responded to treatment
86	Can we send you a survey in the future to ask about your health and healthcare
87a	Ethnic Group
87b	Broader ethnic group

	W<br>Pati
**Item number**	**Data item**
1	Pseudonymised patient ID
2	Pseudonymised tumourID
3	Before you were told you needed to go to hospital about cancer, how many times did you see your GP (family doctor) about the health problem caused by cancer?
4	How do you feel about the length of time you had to wait before your first appointment with a hospital doctor?
5	How long was it from the time you first thought something might be wrong with you until you first saw a GP or other doctor?
6	In the last 12 months, have you had a diagnostic test(s) for cancer such as an endoscopy, biopsy, mammogram, or scan at one of the hospitals named in the covering letter?
7	Beforehand, did you have all the information you needed about your test?
8	Overall, how did you feel about the length of time you had to wait for your test to be done?
9	Were the results of the test explained in a way you could understand?

For peer review only

10	When you were first told that you had cancer, had you been told you could bring a family member or friend with you?
11	How do you feel about the way you were told you had cancer?
12	Did you understand the explanation of what was wrong with you?
13	When you were told you had cancer, were you given written information about the type of cancer you had?
14	Before your cancer treatment started, were your treatment options explained to you?
15	Were the possible side effects of treatment(s) explained in a way you could understand?
16	Were you offered practical advice and support in dealing with the side effects of your treatment(s)?
17	Before you started your treatment(s), were you also told about any side effects of the treatment that could affect you in the future rather than straight away?
18	Were you involved as much as you wanted to be in decisions about your care and treatment?
19	Were you given the name of a Clinical Nurse Specialist who would support you through your treatment?
20	How easy or difficult has it been for you to contact your Clinical Nurse Specilaist?
21	When you have had important questions to ask your Clinical Nurse Specialist, how often have you got answers you could understand?
22	Did hospital staff give you information about support or self-help groups for people with cancer?
23	Did hospital staff discuss with you or give you information about the impact cancer could have on your day to day activities (for example, your work life or education)?
24	Did hospital staff give you information about how to get financial help or any benefits you might be entitled to?
25	Did hospital staff tell you that you could get free prescriptions?
26	During the last 12 months, have you had an operation (such as removal of a tumour or lump) at one of the hospitals named in the covering letter?
27	Beforehand, did you have all the information you needed about your operation?
28	After the operation, did a member of staff explain how it had gone in a way you could understand?
29	During the last 12 months, have you had an operation or stayed overnight for cancer care at one of the hospitals named in the covering letter?
30	Did groups of doctors and nurses talk in front of you as if you weren't there?
31	Did you have confidence and trust in the doctors treating you?
32	If your family or someone else close to you wanted to talk to a doctor, were they able to?
33	Did you have confidence and trust in the ward nurses treating you?
34	In your opinion, were there enough nurses on duty to care for you in hospital?
35	While you were in hospital did the doctors and nurses ask you what name you prefer to be called by?
36	Were you given enough privacy when discussing your condition or treatment?
37	During your hospital visit, did you find someone on the hospital staff to talk to about your worries and fears?
38	Do you think the hospital staff did everything they could to help control your pain?

For peer review only

39	Overall, did you feel you were treated with respect and dignity while you were in the hospital?
40	Were you given clear written information about what you should or should not do after leaving hospital?
41	Did the hospital staff tell you who to contact if you were worried about your condition or treatment after you left hospital?
42	During the last 12 months, have you been treated as an oupatient or day case for cancer care at one of the hospitals named in the covering letter?
43	While you were being treated an an outpatient or day case, did you find someone on the hospital staff to talk to about your worries and fears?
44	The last time you had an outpatients appointment with a cancer doctor, did they have the right documents, such as medical notes, x-rays and test results?
45	During the last 12 months, have you had radiotherapy at any of the hospitals named in the covering letter?
46	Beforehand, did you have all of the information you needed about your radiotherapy treatment?
47	Once you started your treatment, were you given enough information about whether your radiotherapy was working in a way you could understand?
48	During the last 12 months, have you had chemotherapy at any of the hospitals named in the covering letter?
49	Beforehand, did you have all of the information you needed about your chemotherapy treatment?
50	Once you started your treatment, were you given enough information about whether your chemotherapy was working in a way you could understand?
51	Did the doctors or nurses give your family or someone close to you all the information they needed to help care for you at home?
52	During your cancer treatment, were you given enough care and support from health or social services (for example, district nurses, home helps or physiotherapists)?
53	Once your cancer treatment finished, were you given enough care and support from health or social services (for example, district nurses, home helps or physiotherapists)?
54	As far as you know, was your GP given enough information about your condition and the treatment you had at the hospital?
55	Do you think the GPs and nurses at your general practice did everything they could to support you while you were having cancer treatment?
56	Did the different people treating and caring for you (such as GP, hospital doctors, hospital nurses, specialist nurses, community nurses) work well together to give you the best possible care?
57	Have you been given a care plan?
58	Overall, how would you rate the administration of your care (getting letters at the right time, doctors having the right notes/tests results, etc.)?
59	Overall, how do you feel about the length of time you had to wait when attending clinics and appointments for your cancer treatment?
60	Since your diagnosis, has anyone discussed with you whether you would like to take part in cancer research?
61	Overall, how would you rate your care?
62	How long is it since you were first treated for this cancer?
63	Had your cancer spread to other organs or parts of your body at the time you were first told you had cancer?
64	Which of the following applies?
67	What year were you born?
68a	Age
68b	Age bands
69	Are you male or female?
70	**Which of the following options best describes how you think of yourself? (sexual orientation)**

**71**	Do you have any of the following long-standing conditions?
**72**	Could we send you a survey in the future to ask about your health and healthcare?
**73**	Is English your first language?
**74a**	What is your ethnic group?
**74b**	Broader ethnic group

For peer review only

is a survey sent out to all adult cancer patients (aged 16 and over) with a primary diagnosis of cancer who have been admitt
ee month sampling period each year. The survey aims to collect information from patients about their cancer journey from th

e you to have patient consent, section 251 approval, or the appropriate statutory regulation covering your organisation. Sens
render the data anonymised to k-3 anonymity. Privacy by design and default principles have also been applied to some of th

/ave 1 2010 Cancer Patient Experience Survey
ients discharged between 01/01/2010 – 31/03/2010

Possible answers	Field Name
	PATIENTID
	TUMOURID
**Seeing your GP**	
None I did not see my GP before going to hospital, I saw my GP once, I saw my GP twice, I saw my GP 3 or 4 times, I saw my GP 5 or more times, Don't know / Can't say	Q1
I was seen the same day or next day, I was seen after 2-14 days, I was seen in 3 to 4 weeks, Waited 1 to 4 months, Waited more than 4 months, I did not see my GP before going into hospital, I chose to have a later appointment than the one I was offered, Don't know / Can't remember	Q2
I was seen as soon as I thought was necessary, I should have been seen a bit sooner, I should have been seen a lot sooner	Q3
Less than 3 months, 3-6 months, 6-12 months, More than 12 months, Don't know / Can't remember	Q4
My health got worse, My health got better, My health stayed about the same	Q5
**Diagnostic Tests**	
Yes, No	Q6
Yes completely, Yes to some extent, No but I would have liked an explanation, I did not need an explanation, Don't know / Can't remember	Q7
Yes completely, Yes to some extent, No but I would have liked an explanation, I did not need an explanation, Don't know / Can't remember	Q8
Yes and it was easy to understand, Yes but it was difficult to understand, No but I would have liked written information about the test(s), I did not need written information, Don't know / Can't remember	Q9
Yes completely, Yes to some extent, No but I would have liked an explanation, I did not need an explanation, Don’t know / Can’t remember	Q10
**Finding out what was wrong with you**	
A hospital doctor, A hospital nurse, A GP (family doctor), Another health professional, A friend or relative, Nobody – I worked it out for myself	Q11
Yes, No, It was not necessary, I was told by phone or letter, Don’t know / Can’t remember	Q12
It was done sensitively, It should have been done a bit more sensitively, It should have been done a lot more sensitively	Q13
Yes I completely understood it, Yes I understood some of it, No I did not understand it, Can’t remember	Q14
Yes and it was easy to understand, Yes but it was difficult to understand, No I was not given written information about the type of cancer I had, I did not need written information, Don’t know / Can’t remember	Q15

**Deciding the best treatment for you**	
Yes, No but I would have liked a choice, I was not given a choice because only one type of treatment was suitable for me, Not sure / Can't remember	Q16
Yes definitely, Yes to some extent, No side effects were not explained, I did not need an explanation, Not sure / Can't remember	Q17
Yes and it was easy to understand, Yes but it was difficult to understand, No I was not given written information about side effects, Don't know / Can't remember	Q18
Yes definitely, Yes to some extent, No but I would like to have been more involved, Only one type of treatment was suitable for me	Q19
**Clinical Nurse Specialist**	
Yes, No, Don't know / Not sure	Q20
Easy, Sometimes easy sometimes difficult, Difficult, I have not tried to contact her/him	Q21
Yes definitely, Yes to some extent, No	Q22
All or most of the time, Some of the time, Rarely or never, I do not ask any questions	Q23
Too short, About right, Too long	Q24
**Support for people with Cancer**	
Yes, No but I would have liked information, It was not necessary, Don't know / Can't remember	Q25
Yes, No but I would have liked information, It was not necessary, Don't know / Can't remember	Q26
Yes, No but I would have liked information, It was not necessary, Don't know / Can't remember	Q27
**Operations**	
Yes, No	Q28
No, Yes it was changed once, Yes it was changed 2 or 3 times, Yes it was changed 4 times or more	Q29
Yes completely, Yes to some extent, No but I would have liked an explanation, I did not need an explanation, Don't know / Can't remember	Q30
Yes and it was easy to understand, Yes but it was difficult to understand, No I was not given written information about my operation, Don't know / Can't remember	Q31
Yes completely, Yes to some extent, No but I would have liked an explanation, I did not need an explanation`	Q32
**Hospital doctors**	
Yes, No	Q33
All or most of the time, Some of the time, Rarely or never, I did not ask any questions	Q34
In all of them, In some of them, In none of them	Q35
Yes definitely, Yes to some extent, No, Don't know / Not sure	Q36
Yes often, Yes sometimes, No	Q37
Yes definitely, Yes to some extent, No, No family or friends were involved, My family did not want or need information, I did not want my family or friends to talk to a doctor	Q38
**Ward Nurses**	
All or most of the time, Some of the time, Rarely or never, I did not ask any questions	Q39
In all of them, In some of them, In none of them	Q40
Yes often, Yes sometimes, No	Q41
There were always or nearly always enough on duty, There were sometimes enough on duty, There were rarely or never enough on duty	Q42
**Hospital care and treatment**	
Often, Sometimes, Only once, Never	Q43
Often, Sometimes, Only once, Never	Q44
Yes always, Yes sometimes, No	Q45
Yes always, Yes sometimes, No	Q46
All of the time, Some of the time, Not at all, I did not have any pain	Q47
Always, Most of the time, Some of the time, Never	Q48
**formation given to you before you left hospital**	
Yes, No, Can't remember	Q49
Yes, No, Don't know / Can't remember	Q50
Yes definitely, Yes to some extent, No, No family or friends were involved, My family or friends did not want or need information, I did not want my family or friends to be given information	Q51
**Arranging home support**	

For peer review only

Yes definitely, Yes to some extent, No, I did not need help from health or social services, Don't know / Can't remember	Q52
**Hospital care as day patient/ outpatient**	
Yes, No	Q53
Yes definitely, Yes to some extent, No they could have done more, I have not had any side effects from radiotherapy	Q54
Yes, No	Q55
Yes definitely, Yes to some extent, No they could have done more, I have not had side effects from chemotherapy	Q56
Yes definitely, Yes to some extent, No they could have done more, I did not have any pain	Q57
Yes definitely, Yes to some extent, No I would have liked more support, I did not need emotional support from staff	Q58
**Outpatient appointments with doctors**	
Yes, No	Q59
Seen on time or early, Waited up to 5 minutes, Waited 6 - 15 minutes, Waited 16 - 30 minutes, Waited 31 - 60 minutes, Waited 1 to 2 hours, Waited more than 2 hours, Don't know / Can't remember	Q60
Too short, About right, Too long	Q61
Yes, No, Don't know / Can't remember	Q62
**Care from your general practice**	
Yes, No, Don't know / Can't remember	Q63
Yes definitely, Yes to some extent, No they could have done more, My general practice was not involved	Q64
**Your overall NHS care**	
Yes always, Yes most of the time, Yes some of the time, No never, Don't know	Q65
Not enough, The right amount, Too much	Q66
Yes often, Yes sometimes, No	Q67
**About you**	
	Q68
	Q71_AGE_BAND
Male, Female	q69
Deafness or severe hearing impairment, Blindness or partially sighted, A long-standing physical condition, A learning disability, A mental health condition, A long-standing illness such as HIV diabetes chronic heart disease or epilepsy, No I do not have a longstanding condition	Q70A, Q70B, Q70C, Q70D, Q70E,Q70F, Q70G
Less than 1 year, 1 to 5 years, More than 5 years, Don't know / Can't remember	Q71
**Heterosexual / straight (opposite sex), Bisexual (both sexes), Gay or Lesbian (same sex), Other, Prefer not to answer**	**Q72**
Yes and I understand that this does not mean that I would have to take part in the future survey, No I would prefer you not to contact me again	Q73
British, Irish, Any other White background, White and Black Caribbean, White and Black African, White and Asian, Any other mixed background, Indian, Pakistani, Bangladeshi, Any other Asian background, Caribbean, African, Any other Black background, Chinese, Any other ethnic group	Q74
Option to group ethnicities (e.g. white/ non-white/ unknown)	

**ve 2 2011/12 Cancer Patient Experience Survey**<br>ients discharged between 01/09/2011 – 30/11/2011	
	**Field Name**
	PATIENTID
	TUMOURID
**Seeing your GP**	
None I did not see my GP before going to hospital, I saw my GP once, I saw my GP twice, I saw my GP 3 or 4 times, I saw my GP 5 or more times, Don't know / Can't say	Q1
I was seen as soon as I thought was necessary, I should have been seen a bit sooner, I should have been seen a lot sooner	Q2
Less than 3 months, Don't know / Can't remember, More than 12 months, 6-12 months, 3-6 months	Q3

For peer review only

My health stayed about the same, My health got better, My health got worse	Q4
**Diagnostic tests**	
Yes, No	Q5
Yes completely, Don't know / Can't remember, I did not need an explanation, No but I would have liked an explanation, Yes to some extent	Q6
Yes to some extent, Yes completely, No but I would have liked an explanation, I did not need an explanation, Don't know / Can't remember	Q7
Yes but it was difficult to understand, Yes and it was easy to understand, No but I would have liked written information about the test(s), Don't know / Can't remember, I did not need written information	Q8
I did not need an explanation, No but I would have liked an explanation, Yes completely, Yes to some extent, Don’t know / Can’t remember	Q9
**Finding out what was wrong with you**	
A GP (family doctor), Another health professional, A hospital nurse, A hospital doctor, A friend or relative, Nobody – I worked it out for myself	Q10
No, Yes, It was not necessary, Don’t know / Can’t remember, I was told by phone or letter	Q11
It was done sensitively, It should have been done a bit more sensitively, It should have been done a lot more sensitively	Q12
Yes I completely understood it, No I did not understand it, Yes I understood some of it, Can’t remember	Q13
I did not need written information, No I was not given written information about the type of cancer I had, Yes but it was difficult to understand, Yes and it was easy to understand, Don’t know / Can’t remember	Q14
**Deciding the best treatment for you**	
Not sure / Can’t remember, I was not given a choice because only one type of treatment was suitable for me, No but I would have liked a choice, Yes	Q15
I didn't know my treatment was being discussed by a team of doctors / nurses, No my views were not taken into account, Yes to some extent, Not sure / Can't remember, Yes definitely	Q16
Yes definitely, Yes to some extent, Not sure / Can’t remember, I did not need an explanation, No side effects were not explained	Q17
Yes and it was easy to understand, Yes but it was difficult to understand, No I was not given written information about side effects, Don’t know / Can’t remember	Q18
No but I would like to have been more involved, Yes to some extent, Yes definitely, Not sure / Can't remember	Q19
**Clinical Nurse Specialist**	
Don’t know / Not sure, Yes, No	Q20
Difficult, Sometimes easy sometimes difficult, Easy, I have not tried to contact her/him	Q21
Yes definitely, Yes to some extent, No	Q22
All or most of the time, Some of the time, Rarely or never, I do not ask any questions	Q23
**Support for people with cancer**	
No but I would have liked information, Yes, It was not necessary, Don’t know / Can’t remember	Q24
It was not necessary, No but I would have liked information, Yes, Don't know / Can't remember	Q25
It was not necessary, No but I would have liked information, Yes, Don’t know / Can’t remember	Q26
Yes, No, Don't know / Can't remember	Q27
Yes, No	Q28
Yes, No	Q29
**Operations**	
Yes, No	Q30
No, Yes it was changed once, Yes it was changed 2 or 3 times, Yes it was changed 4 times or more	Q31
Yes completely, Yes to some extent, Don't know / Can't remember, I did not need an explanation, No but I would have liked an explanation	Q32
No I was not given written information about my operation, Yes but it was difficult to understand, Yes and it was easy to understand, Don’t know / Can’t remember	Q33
Yes completely, Yes to some extent, No but I would have liked an explanation, I did not need an explanation`	Q34
**Hospital doctors**	
Yes, No	Q35

Rarely or never, Some of the time, All or most of the time, I did not ask any questions	Q36
In all of them, In some of them, In none of them	Q37
Yes often, Yes sometimes, No	Q38
No, Yes to some extent, Yes definitely, No family or friends were involved, I did not want my family or friends to talk to a doctor, My family did not want or need information	Q39
**Ward Nurses**	
Some of the time, All or most of the time, Rarely or never, I did not ask any questions	Q40
In some of them, In all of them, In none of them	Q41
Yes often, Yes sometimes, No	Q42
There were sometimes enough on duty, There were always or nearly always enough on duty, There were rarely or never enough on duty	Q43
**Hospital care and treatment**	
Often, Never, Only once, Sometimes	Q44
Sometimes, Often, Only once, Never	Q45
Only some of them did, Yes all of them did, None of them did	Q46
Yes always, Yes sometimes, No	Q47
Yes always, Yes sometimes, No	Q48
As much as I wanted, Most of the time, I did not have any worries or fears, Not at all but would have liked to, Some of the time	Q49
All of the time, Some of the time, Not at all, I did not have any pain	Q50
Always, Most of the time, Never, Some of the time	Q51
**formation given to you before you left hospital**	
No, Yes, Can't remember	Q52
Yes, No, Don't know / Can't remember	Q53
No, Yes to some extent, Yes definitely, No family or friends were involved, My family or friends did not want or need information, I did not want my family or friends to be given information	Q54
**Arranging home support**	
Yes definitely, Yes to some extent, No, I did not need help from health or social services, Don't know / Can't remember	Q55
**Hospital care as a day patient/ outpatient**	
No they could have done more, Yes to some extent, Yes definitely, I have not had any side effects from radiotherapy, I have not had radiotherapy	Q56
Yes definitely, Yes to some extent, No they could have done more, I have not had any side effects from chemotherapy, I have not had chemotherapy	Q57
Yes definitely, Yes to some extent, I did not have any pain, No they could have done more	Q58
Yes definitely, No I would have liked more support, Yes to some extent, I did not need emotional support from staff	Q59
**Outpatient appointments with doctors**	
Yes, No	Q60
Seen on time or early, Waited 6 - 15 minutes, Waited up to 5 minutes, Waited 16 - 30 minutes, Waited 31 - 60 minutes, Waited 1 to 2 hours, Waited more than 2 hours, Don't know / Can't remember	Q61
Too short, Too long, About right	Q62
Don't know / Can't remember, Yes, No	Q63
**Care from your general practice**	
Yes, No, Don't know / Can't remember	Q64
Yes to some extent, No they could have done more, My general practice was not involved, Yes definitely	Q65
**Your overall NHS care**	
Don't know, Yes most of the time, Yes some of the time, No never, Yes always	Q66
The right amount, Too much, Not enough	Q67
Yes, No, Don't know / Can't remember	Q68
Yes often, Yes sometimes, No	Q69
Excellent, Very good, Good, Fair, Poor	Q70
**About you**	
	Q71
	Q71_AGE_BAND
Male, Female	Q72

**Heterosexual / straight (opposite sex), Prefer not to answer, Other, Gay or Lesbian (same sex), Bisexual (both sexes)**	**Q73**
Deafness or severe hearing impairment, Blindness or partially sighted, A long-standing physical condition, A learning disability, A mental health condition, A long-standing illness such as HIV diabetes chronic heart disease or epilepsy, No I do not have a longstanding condition	Q74A, Q74B, Q74C, Q74D, Q74E, Q74F, Q74G
1 to 5 years, Less than 1 year, More than 5 years, Don't know / Can't remember	Q75
Yes and I understand that this does not mean that I would have to take part in the future survey, No I would prefer you not to contact me again	Q76
British, Any other ethnic group, Any other White background, White and Black Caribbean, White and Black African, White and Asian, Any other mixed background, Indian, Pakistani, Bangladeshi, Any other Asian background, Caribbean, African, Any other Black background, Chinese, Irish	Q77
Option to group ethnicities (e.g. white/ non-white/ unknown)	

/ave 3 2013 Cancer Patient Experience Survey
ients discharged between 01/09/2012 – 30/09/2012

	**Field Name**
	PATIENTID
	TUMOURID
**Seeing your GP**	
None - I did not see my GP before going to hospital, I saw my GP once, I saw my GP twice, I saw my GP 3 or 4 times, I saw my GP 5 or more times, Don't know / can't say	Q1
I was seen as soon as I thought was necessary, I should have been seen a bit sooner, I should have been seen a lot sooner	Q2
Less than 3 months, 3-6 months, 6-12 months, More than 12 months, Don't know / can't remember	Q3
My health got worse, My health got better, My health stayed about the same	Q4
**Diagnostic tests**	
Yes, No	Q5
Yes, completely, Yes, to some extent, No, but I would have liked an explanation, I did not need an explanation, Don't know / can't remember	Q6
Yes, completely, Yes, to some extent, No, but I would have liked an explanation, I did not need an explanation, Don't know / can't remember	Q7
Yes, and it was easy to understand, Yes, but it was difficult to understand, No, but I would have liked written information about the test(s), I did not need written information, Don't know / can't remember	Q8
Yes, completely, Yes, to some extent, No, but I would have liked an explanation, I did not need an explanation, Don't know / can't remember	Q9
**Finding out what was wrong with you**	
A hospital doctor, A hospital nurse, A GP (family doctor), Another health professional, A friend or relative, Nobody ? I worked it out for myself	Q10
Yes, No, It was not necessary, I was told by phone or letter, Don't know / can't remember	Q11
It was done sensitively, It should have been done a bit more sensitively, It should have been done a lot more sensitively	Q12
Yes, I completely understood it, Yes, I understood some of it, No, I did not understand it, Can't remember	Q13
Yes, and it was easy to understand, Yes, but it was difficult to understand, No, I was not given written information about the type of cancer I had, I did not need written information, Don't know / can't remember	Q14
**Deciding the best treatment for you**	
Yes, No, but I would have liked a choice, I was given a choice because only one type of treatment was suitable for me, Not sure / can't remember	Q15
Yes, definitely, Yes, to some extent, No, my views were not taken into account, I didn't know my treatment was being discussed by a team of doctors / nurses, Not sure / can't remember	Q16
Yes, definitely, Yes, to some extent, No, side effects were not explained, I did not need an explanation, Not sure / can't remember	Q17
Yes, and it was easy to understand, Yes, but it was difficult to understand, No, I was not given written information about side effects, Don't know / can't remember	Q18

Yes, definitely, Yes, to some extent, No, future side effects were not explained, I did not need an explanation, Not sure / can't remember	Q19
Yes, definitely, Yes, to some extent, No, but I would like to have been more involved, Not sure / can't remember	Q20
**Clinical Nurse Specialist**	
Yes, No, Don't know / not sure	Q21
Easy, Sometimes easy, sometimes difficult, Difficult, I have not tried to contact her/him	Q22
Yes, definitely, Yes, to some extent, No	Q23
All or most of the time, Some of the time, Rarely or never, I do not ask any questions	Q24
**Support for people with cancer**	
Yes, No, but I would have liked information, It was necessary, Don't know / can't remember	Q25
Yes, No, but I would have liked a discussion or information, It was not necessary / relevant to me, Don't know / can't remember	Q26
Yes, No, but I would have liked information, It was not necessary, Don't know / can't remember	Q27
Yes, No, but I would have liked information, It was not necessary, Don't know / can't remember	Q28
Yes, No	Q29
Yes, No, Don't know / can't remember	Q30
Yes, No	Q31
**Operations**	
Yes, No	Q32
Yes, completely, Yes, to some extent, No, but I would have liked an explanation, I did not need an explanation, Don't know / can't remember	Q33
Yes, and it was easy to understand, Yes, but it was difficult to understand, No, I was not given written information about my operation, Don't know / can't remember	Q34
Yes, completely, Yes, to some extent, No, but I would have liked an explanation, I did not need an explanation	Q35
**Hospital doctors**	
Yes, No	Q36
All or most of the time, Some of the time, Rarely or never, I did not ask any questions	Q37
In all of them, In some of them, In none of them	Q38
Yes, often, Yes, sometimes, No	Q39
Yes, definitely, Yes, to some extent, No, No family or friends were involved, My family did not want or need information, I did not want my family or friends to talk to a doctor	Q40
**Ward nurses**	
All or most of the time, Some of the time, Rarely or never, I did not ask any questions	Q41
In all of them, In some of them, In none of them	Q42
Yes, often, Yes, sometimes, No	Q43
There were always or nearly always enough on duty, There were sometimes enough on duty, There were rarely or never enough on duty	Q44
**Hospital care and treatment**	
Often, Sometimes, Only once, Never	Q45
Often, Sometimes, Only once, Never	Q46
Yes, all of them did, Only some of them did, None of them did	Q47
Yes, always, Yes, sometimes, No	Q48
Yes, sometimes, Yes, sometimes, No	Q49
As much as I wanted, Most of the time, Some of the time, Not at all, but would have liked to, I did not have any worries or fears	Q50
All of the time, Some of the time, Not at all, I did not have any pain	Q51
Always, Most of the time, Some of the time, Never	Q52
Yes, No, Can't remember	Q53
Yes, No, Don't know / can't remember	Q54
Yes, definitely, Yes, to some extent, No, No family or friends were involved, My family or friends did not want or need information, I did not want my family or friends to be given information	Q55
**Arranging home support**	
Yes, definitely, Yes, to some extent, No, I did not need help from health or social services, Don't know / can't remember	Q56

For peer review only

**Hospital care as day patient/ outpatient**	
Yes, definitely, Yes, to some extent, No, they could have done more, I have not had any side effects from radiotherapy, I have not had radiotherapy	Q57
Yes, definitely, Yes, to some extent, No, they could have done more, I have not had any side effects from chemotherapy, I have not had chemotherapy	Q58
Yes, definitely, Yes, to some extent, No, they could have done more, I did not have any pain	Q59
Yes, definitely, Yes, to some extent, No, I would have liked more support, I did not need emotional support from staff	Q60
**Outpatient appointments with doctors**	
Yes, No	Q61
Yes, No, Don't know / can't remember	Q62
**Care from your general practice**	
Yes, No, Don't know / can't remember	Q63
Yes, definitely, Yes, to some extent, No, they could have done more, My general practice was not involved	Q64
**Your overall NHS care**	
Yes, always, Yes, most of the time, Yes, some of the time, No, never, Don't know	Q65
Physiotherapist	Q66A
Occupational therapist (OT)	Q66B
Dietician	Q66C
Speech and language therapist	Q66D
Lymphoedema specialist	Q66E
Not enough, The right amount, Too much	Q67
Yes, No, Don't know / can't remember	Q68
Yes, often, Yes, sometimes, No	Q69
Excellent, Very good, Good, Fair, Poor	Q70
**About you**	
	Q71
	Q71_AGE
	Q71_AGE_BAND
Male, Female	Q72
**Heterosexual / straight (opposite sex), Bisexual (both sexes), Gay or Lesbian (same sex), Other, Prefer not to answer**	**Q73**
Full time employment, Part time employment, Homemaker, Student (in education), Retired, Unemployed - and seeking work, Unemployed - unable to work for health reasons, Other	Q74
Deafness or severe hearing impairment, Deafness or severe hearing impairment, Blindness or partially sighted, A long-standing physical condition, A learning disability, A mental health condition, A long-standing illness, such as cancer, HIV, diabetes, chronic heart disease, or epilepsy, No, I do not have a long-standing condition	Q75A, Q75B, Q75C, Q75D, Q75E, Q75F, Q75G
Less than 1 year, 1 to 5 years, More than 5 years, Don't know / can't remember	Q76
My cancer has responded fully to treatment (I have no signs or symptoms of cancer), My cancer has been treated but is still present, My cancer has not been treated at all, My cancer has come back after it was originally treated, My original cancer responded but I now have a new cancer, I am not certain what is happening with my cancer	Q77
Yes, and I understand that this does not mean that I would have to take part in the future survey, No, I would prefer you not to contact me again	Q78
British, Irish, Any other White background, White and Black Caribbean, White and Black African, White and Asian, Any other mixed background, Indian, Pakistani, Bangladeshi, Any other Asian background, Caribbean, African, Any other Black background, Chinese, Any other ethnic background	Q79
Option to group ethnicities (e.g. white/ non-white/ unknown)	

/ave 4 2014 Cancer Patient Experience Survey
ients discharged between 01/09/2013 – 30/11/2013

	**Field Name**
	PATIENTID
	TUMOURID

Seeing your GP	
None - I didn't see my GP before going to hospital, I saw my GP once, I saw my GP twice, I saw my GP 3 or 4 times, I saw my GP 5 or more times, Don't know / can't say	Q1
I was seen as soon as I thought was necessary, I should have been seen a bit sooner, I should have been seen a lot sooner	Q2
Less than 3 months, 3-6 months, 6-12 months, More than 12 months, Don't know / can't remember	Q3
My health got worse, My health got better, My health stayed about the same	Q4
Yes, No	Q5
**Diagnostic tests**	
Yes, completely, Yes, to some extent, No, but I would have liked an explanation, I did not need an explanation, Don't know / can't remember	Q6
Yes, completely, Yes, to some extent, No, but I would have liked an explanation, I did not need an explanation, Don't know / can't remember	Q7
Yes, and it was easy to understand, Yes, but it was difficult to understand, No, but I would have liked written information about the test(s), I did not need written information, Don't know / can't remember	Q8
Yes, completely, Yes, to some extent, No, but I would have liked an explanation, I did not need an explanation, Don't know / can't remember	Q9
**Finding out what was wrong with you**	
A hospital doctor, A hospital nurse, A GP (family doctor), Another health professional, A friend or relative, Nobody – I worked it out for myself	Q10
Yes, No, It was not necessary, I was told by phone or letter, Don't know / can't remember	Q11
It was done sensitively, It should have been done a bit more sensitively, It should have been done a lot more sensitively	Q12
Yes, I completely understood it, Yes, I understood some of it, No, I did not understand it, Can't remember	Q13
Yes, and it was easy to understand, Yes, but it was difficult to understand, No, I was not given written information about the type of cancer I had, I did not need written information, Don't know / can't remember	Q14
**Deciding the best treatment for you**	
Yes, No, but I would have liked a choice, I was given a choice because only one type of treatment was suitable for me, Not sure / can't remember	Q15
Yes, definitely, Yes, to some extent, No, my views were not taken into account, I didn't know my treatment was being discussed by a team of doctors / nurses, Not sure / can't remember	Q16
Yes, definitely, Yes, to some extent, No, side effects were not explained, I did not need an explanation, Not sure / can't remember	Q17
Yes, and it was easy to understand, Yes, but it was difficult to understand, No, I was not given written information about side effects, Don't know / can't remember	Q18
Yes, definitely, Yes, to some extent, No, future side effects were not explained, I did not need an explanation, Not sure / can't remember	Q19
Yes, definitely, Yes, to some extent, No, but I would like to have been more involved, Not sure / can't remember	Q20
**Clinical Nurse Specialist**	
Yes, No, Don't know / not sure	Q21
Easy, Sometimes easy, sometimes difficult, Difficult, I have not tried to contact her/him	Q22
Yes, definitely, Yes, to some extent, No	Q23
All or most of the time, Some of the time, Rarely or never, I do not ask any questions	Q24
**Support for people with cancer**	
Yes, No, but I would have liked information, It was necessary, Don't know / can't remember	Q25
Yes, No, but I would have liked a discussion or information, It was not necessary / relevant to me, Don't know / can't remember	Q26
Yes, No, but I would have liked information, It was not necessary, Don't know / can't remember	Q27
Yes, No, but I would have liked information, It was not necessary, Don't know / can't remember	Q28
Yes, No	Q29
Yes, No, Don't know / can't remember	Q30
Yes, No	Q31
**Operations**	

Yes, No	Q32
Yes, completely, Yes, to some extent, No, but I would have liked an explanation, I did not need an explanation, Don't know / can't remember	Q33
Yes, and it was easy to understand, Yes, but it was difficult to understand, No, I was not given written information about my operation, Don't know / can't remember	Q34
Yes, completely, Yes, to some extent, No, but I would have liked an explanation, I did not need an explanation	Q35
**Hospital doctors**	
Yes, No	Q36
All or most of the time, Some of the time, Rarely or never, I did not ask any questions	Q37
In all of them, In some of them, In none of them	Q38
Yes, often, Yes, sometimes, No	Q39
Yes, definitely, Yes, to some extent, No, No family or friends were involved, My family did not want or need information, I did not want my family or friends to talk to a doctor	Q40
**Ward Nurses**	
All or most of the time, Some of the time, Rarely or never, I did not ask any questions	Q41
In all of them, In some of them, In none of them	Q42
Yes, often, Yes, sometimes, No	Q43
There were always or nearly always enough on duty, There were sometimes enough on duty, There were rarely or never enough on duty	Q44
**Hospital care and treatment**	
Often, Sometimes, Only once, Never	Q45
Often, Sometimes, Only once, Never	Q46
Yes, all of them did, Only some of them did, None of them did	Q47
Yes, always, Yes, sometimes, No	Q48
Yes, sometimes, Yes, sometimes, No	Q49
As much as I wanted, Most of the time, Some of the time, Not at all, but would have liked to, I did not have any worries or fears	Q50
All of the time, Some of the time, Not at all, I did not have any pain	Q51
Always, Most of the time, Some of the time, Never	Q52
Yes, No, Can't remember	Q53
Yes, No, Don't know / can't remember	Q54
**Home care and support**	
Yes, definitely, Yes, to some extent, No, No family or friends were involved, My family or friends did not want or need information, I did not want my family or friends to be given information	Q55
Yes, definitely, Yes, to some extent, No, I did not need help from health or social services, Don't know / can't remember	Q56
**Hospital care as day patient/ outpatient**	
Yes, definitely, Yes, to some extent, No, they could have done more, I have not had any side effects from radiotherapy, I have not had radiotherapy	Q57
Yes, definitely, Yes, to some extent, No, they could have done more, I have not had any side effects from chemotherapy, I have not had chemotherapy	Q58
Yes, definitely, Yes, to some extent, No, they could have done more, I did not have any pain	Q59
Yes, definitely, Yes, to some extent, No, I would have liked more support, I did not need emotional support from staff	Q60
**Outpatient appointments with doctors**	
Yes, No	Q61
Yes, No, Don't know / can't remember	Q62
**Care from your general practice**	
Yes, No, Don't know / can't remember	Q63
Yes, definitely, Yes, to some extent, No, they could have done more, My general practice was not involved	Q64
**Your overall NHS care**	
Yes, always, Yes, most of the time, Yes, some of the time, No, never, Don't know	Q65
Physiotherapist	Q66A
Occupational therapist (OT)	Q66B
Dietician	Q66C

For peer review only

Speech and language therapist	Q66D
Lymphoedema specialist	Q66E
Not enough, The right amount, Too much	Q67
Yes, No, Don't know / can't remember	Q68
Yes, often, Yes, sometimes, No	Q69
Excellent, Very good, Good, Fair, Poor	Q70
**About you**	
	Q71
	Q71_AGE
	Q71_AGE_BAND
Male, Female	Q72
**Heterosexual / straight (opposite sex), Bisexual (both sexes), Gay or Lesbian (same sex), Other, Prefer not to answer**	**Q73**
Full time employment, Part time employment, Homemaker, Student (in education), Retired, Unemployed - and seeking work, Unemployed - unable to work for health reasons, Other	Q74
Deafness or severe hearing impairment, Blindness or partially sighted, A long-standing physical condition, A learning disability, A mental health condition, A long-standing illness, such as cancer, HIV, diabetes, chronic heart disease, or epilepsy, No, I do not have a long-standing condition	Q75A, Q75B,Q75C, Q75D, Q75E, Q75F, Q75G
Less than 1 year, 1 to 5 years, More than 5 years, Don't know / can't remember	Q76
Yes, this is the first time I have been treated for cancer, No, I have been treated for the same type of cancer before but it has now come back, No, I have been treated for a different type of cancer before	Q77
Treatment has not yet started for this cancer, I am in the course of treatment and I can't tell yet how my cancer has responded, The treatment has been effective and I have no signs or symptoms of cancer, I have finished the course of treatment but my cancer is still present, My cancer is being treated again because it has not responded fully to treatment, I am not in active treatment but I am on "Watch and Wait", My cancer has not been treated at all	Q78
Yes, and I understand that this does not mean that I would have to take part in the future survey, No, I would prefer you not to contact me again	Q79
English / Welsh / Scottish / Northern Irish / British, Irish, Gypsy or Irish Traveller, Any other White background, White and Black Caribbean, White and Black African, White and Asian, Any other Mixed / multiple ethnic background, Indian, Pakistani, Bangladeshi, Chinese, Any other Asian background, African, Caribbean, Any other Black / African / Caribbean background	Q80
Option to group ethnicities (e.g. white/ non-white/ unknown)	

/ave 5 2015 Cancer Patient Experience Survey
ients discharged between 01/04/2015 - 30/06/2015

	**Field Name**
	PATIENTID
	TUMOURID
**Seeing your GP**	
None - I went straight to hospital, None - I went to hospital following a cancer screening appointment , I saw my GP once, I saw my GP twice, I saw my GP 3 or 4 times, I saw my GP 5 or more times , Don't know / can't remember	Q1
I was seen as soon as I thought was necessary, I should have been seen a bit sooner, I should have been seen a lot sooner	Q2
Less than 3 months, 3-6 months, 6-12 months , More than 12 months, Don't know / can't remember	Q3
**Diagnostic tests**	
Yes, No	Q4
Yes, No - I would have liked more written information, No- I would have liked more verbal information, I did not need / want any information, Don't know / can't remember	Q5
It was about right, It was a little too long, It was much too long, Don't know / can't remember	Q6
Yes- completely, Yes- to some extent, No - I did not understand the explanation, I did not have an explanation but would have liked one, I did not need an explanation, Don't know / can't remember	Q7

**Finding out what was wrong with you**	
Yes, No, It was not necessary, I was told by phone or letter, Don't know / can't remember	Q8
It was done sensitively, It should have been done a bit more sensitively, It should have been done a lot more sensitively	Q9
Yes - I completely understood it, Yes-, I understood some of it, No- I did not understand it, Don't know / can't remember	Q10
Yes- and it was easy to understand, Yes- but it was difficult to understand, No- I was not given written information about the type of cancer I had, I did not need written information, Don't know / can't remember	Q11
**Deciding the best treatment for you**	
Yes- completely, Yes- to some extent, No, There was only one type of treatment that was suitable for me, Don't know / can't remember	Q12
Yes – definitely, Yes- to some extent, No- side effects were not explained, I did not need an explanation, Don't know / can't remember	Q13
Yes –definitely, Yes- to some extent, No- I was not offered any practical advice or support, Don't know / can't remember	Q14
Yes – definitely, Yes- to some extent, No- future side effects were not explained, I did not need an explanation, Don't know / can't remember	Q15
Yes- definitely, Yes- to some extent, No- but I would like to have been more involved, Don't know / can't remember	Q16
**Clinical Nurse Specialist**	
Yes, No , Don't know / can't remember	Q17
Very easy, Quite easy, Neither easy nor difficult, Quite difficult, Very difficult, I have not tried to contact my Clinical Nurse Specialist	Q18
All or most of the time, Some of the time, Rarely or never, I have not asked any questions	Q19
**Support for people with cancer**	
Yes, No- but I would have liked information, It was not necessary, Don't know / can't remember	Q20
Yes, No - but I would have liked a discussion or information, It was not necessary / relevant to me, Don't know / can't remember	Q21
Yes, No- but I would have liked information, It was not necessary, Don't know / can't remember	Q22
Yes, No- but I would have liked information, It was not necessary, Don't know / can't remember	Q23
**Operations**	
Yes, No	Q24
Yes, No - I would have liked more written information, No - I would have liked more verbal information, I did not need / want information	Q25
Yes - completely, Yes - to some extent, No- but I would have liked an explanation, I did not need an explanation	Q26
**Hospital care as an inpatient**	
Yes, No	Q27
Yes – often, Yes – sometimes, No	Q28
Yes- in all of them, Yes- in some of them, No- in none of them	Q29
Yes- definitely, Yes- to some extent, No- No family or friends were involved, My family did not want to talk to a doctor, I did not want my family or friends to talk to a doctor	Q30
Yes - in all of them, Yes -in some of them, No, in none of them	Q31
There were always or nearly always enough on duty, There were sometimes enough on duty, There were rarely or never enough on duty	Q32
Yes - all of them did, Only some of them did, None of them did	Q33
Yes –always, Yes – sometimes, No	Q34
Yes – definitely, Yes - to some extent, No, I had no worries or fears	Q35
Yes – definitely, Yes - to some extent, No, I did not have any pain	Q36

For peer review only

Yes – always, Yes – sometimes, No	Q37
Yes, No, Don't know/can't remember	Q38
Yes, No, Don't know/can't remember	Q39
**Hospital care as a day patient / outpatient**	
Yes, No	Q40
Yes – definitely, Yes - to some extent, No, I had no worries or fears	Q41
Yes, No, I didn't have an appointment with a cancer doctor, Don't know / can't remember	Q42
Yes, No	Q43
Yes – completely, Yes - to some extent, No, I did not need any information	Q44
Yes – completely, Yes - to some extent, No, It is too early to know if my radiotherapy is working, I did not need any information	Q45
Yes, No	Q46
Yes – completely, Yes - to some extent, No, I did not need any information	Q47
Yes – completely, Yes - to some extent, No, It is too early to know if my chemotherapy is working, I did not need any information	Q48
**Home Care Support**	
Yes –definitely, Yes - to some extent, No, No family or friends were involved, My family or friends did not want or need information, I did not want my family or friends to be involved	Q49
Yes – definitely, Yes - to some extent, No, I did not need help from health or social services, Don't know / can't remember	Q50
Yes – definitely, Yes - to some extent, No, I did not need help from health or social services, Don't know / can't remember	Q51
Yes, No, Don't know/can't remember	Q52
Yes – definitely, Yes - to some extent, No -they could have done more,My general practice was not involved	Q53
Yes – always, Yes - most of the time, Yes- some of the time, No – never, Don't know / can't remember	Q54
Yes, No, I don't know what a care plan is, Don't know/can't remember	Q55
Very good, Good, Neither good nor bad, Quite bad, Very bad, Don't know / can't remember	Q56
It was much too long, It was a little too long, It was about right, Don't know / can't remember	Q57
Yes, No, No, but I would have liked them to, Don't know / can't remember	Q58
Scale 1-10	Q59
Less than 1 year, 1 to 5 years, More than 5 years, Don't know/ can't remember	Q60
Yes, No, Don't know, Does not apply to my type of cancer	Q61
My cancer has been taken out / treated, without any sign of further problem; My cancer was taken out / treated without any sign of further problem, but has since come back / spread to other parts of my body; None of the above options apply to my type of cancer; I would prefer not to say; I don't know	Q62
**About You**	
	Q63
	Q63_AGE
	Q63_AGE_BAND
Male, Female	Q64
**Heterosexual or Straight, Gay or Lesbian, Bisexual, Other, Prefer not to say**	**Q65**

For peer review only

Deafness or severe hearing impairment, Blindness or partially sighted, A long-standing physical condition, A learning disability, A mental health condition, A long-standing illness (such as HIV, diabetes, chronic heart disease, or epilepsy)	Q66
Yes - and I understand that this does NOT mean that I would have to take part in the future survey, No - I would prefer you not to contact me again	Q67
Yes, No	Q68
English / Welsh / Scottish / Northern Irish / British, Irish, Gypsy or Irish Traveller, Any other White background, White and Black Caribbean, White and Black African, White and Asian, Any other Mixed / multiple ethnic background, Indian, Pakistani, Bangladeshi, Chinese, Any other Asian background, African, Caribbean, Any other Black / African / Caribbean background, Arab, Any other ethnic group	Q69
Option to group ethnicities (e.g. white/ non-white/ unknown)	

************************** **End of sheet** **************************

For peer review only

ted to an acute or specialist NHS
eir initial GP visit prior to diagnosis,

sitive patient demographic data,
nese data to provide a less disclosive

Request Field	Justification

Request I	Justification

Request I	Justification

Request I	Justification

Request I	Justification

For peer review only

Introduction

The National Cancer Waiting Times Monitoring Data Set s

- A maximum two week wait from an urgent GP referral fo
- A maximum one month (31-day) wait from diagnosis (CA
- A maximum two month (62-day) wait from urgent GP ref
- A maximum one month (31-day) wait from urgent GP ref
- A maximum 62-day wait from referral from a cancer Scre
- A maximum 62-day wait from a CONSULTANTS decisio
- A maximum 31-day wait for all subsequent treatments fo
- A maximum two week wait from referral for breast sympt

Data Coverage

2009-2015 CWT treatment data (linked to CAS AV tables

Data items selection

Please be aware that selecting patient identifiable data ite organisation. Sensitive patient demographic data, event d design and default principles have also been applied to sc

Key:

**Items in red**	Identifiable fields that require additional per
	Sensitive items that require additional justifi
	Privacy by design and default' alternatives t

Item Number	Data Item
**1**	Pseudonymised patient ID
**2**	**NHS Number**
**3**	**Patient Pathway Identifier**
**4**	Organisation code (patient pathway identifier issuer)
**5**	Pseudonymised organisation code
**6**	Source of referral for out-patients
**7**	Priority type code
**8**	Decision to refer date (cancer or breast symptoms)
**9**	Month of decision to refer
**10**	Year of decision to refer
**11**	Days from another event to decision to refer date
**12**	Cancer referral to treatment period start date
**13**	Month of cancer referral to treatment period
**14**	Year of cancer referral to treatment period
**15**	Days from another event to treatment period start date

**16**	Two week wait cancer or symptomatic breast referral type
**17**	Consultant upgrade date
**18**	Month of consultant upgrade
**19**	Year of consultant upgrade
**20**	Days from another event to consultant upgrade date
**21**	Site code (of provider consultant update)
**22**	Site code (of provider consultant update) (Pseudonymised)
**23**	Date first seen
**24**	Month first seen
**25**	Year first seen
**26**	Days from another event to date first seen
**27**	Site code (of provider first seen)
**28**	Site code (of provider first seen) (pseudonymised)
**29**	Waiting time adjustment (first seen)
**30**	Multidisciplinary team discussion indicator
**31**	Multidisciplinary team discussion date (cancer)
**32**	Month of MDT discussion
**33**	Year of MDT discussion
**34**	Days from another event to multidisciplinary team discussion date
**54**	Cancer or symptomatic breast referral patient status
**55**	Primary diagnosis (ICD)
**56**	Metastatic site
**57**	Tumour laterality
**58**	Cancer treatment period start date
**59**	Month of cancer treatment period
**60**	Year of cancer treatment period
**61**	Days from another event to cancer treatment period start date
**62**	Site code (of provider cancer decision to treat)
**63**	Pseudonymised site code (of provider cancer decision to treat)
**64**	Referral request received date (inter-provider transfer)
**65**	Month of referral request received

For peer review only - http://bmjopen.bmj.com/site/about/guidelines.xhtml

**66**	Year of referral request received
**67**	Days from another event to referral request received date
**68**	Practice code (at cancer treatment period start date)
**69**	Pseudonymised practice code (at cancer treatment period start date)
**70**	Cancer site grouping (broad)
**71**	Cancer site grouping (specific)
**72**	Duplicate flag
**73**	Two week wait detection indicator (same site)
**74**	Two week wait detection indicator (any site)
**75**	Treatment start date (cancer)
**76**	Month treatment started
**77**	Year treatment started
**78**	Days from another event to the treatment start date
**79**	Site code (of provider cancer treatment start date)
**80**	Pseudonymised site code (of provider cancer treatment start date)
**81**	Cancer treatment event type
**82**	Cancer treatment modality
**83**	Clinical trial indicator
**84**	Cancer care setting (treatment)
**85**	Radiotherapy intent
**86**	Radiotherapy priority
**87**	Waiting time adjustment (treatment)

************************* **End of sheet** ********

For peer review only - http://bmjopen.bmj.com/site/about/guidelines.xhtml

supports the continued management and monitc

›r suspected cancer to DATE FIRST SEEN by a
ANCER TREATMENT PERIOD START DATE) t
ferral for suspected cancer to First Definitive Tre
ferral for suspected cancer to First Definitive Tre
eening Programme to first treatment for all cance
›n to upgrade the urgency of a PATIENT they su
›r new cases of primary and recurrent cancer wh
toms (where cancer is not initially suspected) to

for 2009-2014 registrations)

ems – **the data items marked below in red** – w
dates and geographies will be treated as de-pers
ome of these data to provide a less disclosive al

rmissions to access (as outlined above)
fication in your protocol to access
to the sensitive items highlighted in dark grey

**Field Name**
PATIENTID
**NHSNO**
**PPI**
ORG_PPI
ORG_PPI (pseudonymised)
One occu
REF_SOURCE
PRIORITY_TYPE
DEC_TO_REF_DATE
Derived from DEC_TO_REF_DATE
Derived from DEC_TO_REF_DATE
Derived from DEC_TO_REF_DATE
CRTP_DATE
Derived from CRTP_DATE field
Derived from CRTP_DATE field
Derived from CRTP_DATE field

er review only

REF_TYPE
CONS_UPGRADE_DATE
Derived from CONS_UPGRADE_DATE
Derived from CONS_UPGRADE_DATE
Derived from CONS_UPGRADE_DATE
ORG_CONS_UPGRADE
ORG_CONS_UPGRADE (pseudonymised)
DATE_FIRST_SEEN
Derived from DATE_FIRST_SEEN field
Derived from DATE_FIRST_SEEN field
Derived from DATE_FIRST_SEEN field
ORG_FIRST_SEEN
ORG_FIRST_SEEN (pseudonymised)
WTA_FIRST_SEEN
MDT_IND
MDT_DATE
Derived from MDT_DATE field
Derived from MDT_DATE field
Derived from MDT_DATE field
PATIENT_STATUS
DIAG
METS_SITE
LATERALITY
TREAT_PERIOD_START
Derived from TREAT_PERIOD_START
Derived from TREAT_PERIOD_START
Derived from TREAT_PERIOD_START
ORG_DEC_TO_TREAT
ORG_DEC_TO_TREAT (pseudonymised)
REF_REQUEST_RECEIVED
Derived from REF_REQUEST_RECEIVED

For peer review only

Derived from REF_REQUEST_RECEIVED
Derived from REF_REQUEST_RECEIVED
**DERIVED INF**
practice_code
practice_code (pseudonymised)
diag_group_broad
diag_group_specific
dup_flag
detected_same_site
detected_any_site
One occur
TREAT_START
Derived from TREAT_START field
Derived from TREAT_START field
Derived from TREAT_START field
ORG_TREAT_START
ORG_TREAT_START (pseudonymised)
CTE_TYPE
MODALITY
CLIN_TRIAL
CARE_SETTING
RADIO_INTENT
RADIO_PRIORITY
WTA_TREAT

****

ɔring of the following waiting times:

specialist for all suspected cancers
to First Definitive Treatment for all cancers
ɛatment for all cancers
ɛatment for children's cancers, testicular cancers and acute leukaemia
ers
ɹspect to have cancer to first treatment for all cancers
here an Anti-Cancer Drug Regimen, surgery or Radiotherapy is the chosen CANCER TREATMENT MODALITY;
DATE FIRST SEEN

ɩill require you to have patient consent, section 251 approval, or the appropriate statutory regulation covering your
sonalised, unless anonymisation techniques are applied to render the data anonymised to k-3 anonymity. Privacy by
lternative. The key explaining how these alternative can be selected is included below:

**Definition**	**Request Data Item**
**PATIENT AND PATHWAY IDENTIFICATION** To carry Patient and Pathway details. One occurrence of this group is required.	
Project specific patient ID	
**The NHS NUMBER, the primary identifier of a PERSON, is a unique identifier for a PATIENT within the NHS in England and Wales.**	
**An identifier, which together with the ORGANISATION CODE / ORGANISATION IDENTIFIER of the issuer, uniquely identifies a PATIENT PATHWAY.**	
The ORGANISATION CODE of the Organisation issuing the PATIENT PATHWAY IDENTIFIER.	
The pseudonymised ORGANISATION CODE issuing the PATIENT PATHWAY IDENTIFIER.	
**OUTPATIENT SERVICES** To carry Outpatient Services details. rrence of this group is required if applicable to the scenario.	
The source of referral of each Consultant Out-Patient Episode.	
The priority of a request for SERVICES.	
The DATE on which a decision was made to refer the PATIENT to Secondary Care with either suspected cancer, or as an urgent symptomatic breast referral.	
Month of decision to refer	
Year of decision to refer	
Option to provide number of days from another event to decision to refer date (e.g. number of days from decision to refer date to diagnosis)	
The Start Date of a Cancer Referral To Treatment Period.	
Month of cancer referral to treatment period	
Year of cancer referral to treatment period	
Number of days from another event to decision to refer date (e.g. number of days from a cancer referral to diagnosis)	

The site where cancer is suspected by the GENERAL MEDICAL PRACTITIONER or GENERAL DENTAL PRACTITIONER on referral, or, for PATIENTS who are upgraded to an urgent breast cancer PATIENT PATHWAY, identifies that the PATIENT was initially referred on the basis of exhibited (non-cancer) breast symptoms.	
The DATE that the CONSULTANT responsible for the care of the PATIENT (or an authorised member of the CONSULTANT team as defined by local policy) decided that the PATIENT should be upgraded onto an urgent Cancer PATIENT PATHWAY.	
Month of consultant upgrade	
Year of consultant upgrade	
Option to provide number of days from another event to decision to consultant upgrade date (e.g. number of days from decision to consultant upgrade date to diagnosis)	
The ORGANISATION SITE CODE of the Organisation acting as Health Care Provider when a decision is made to upgrade the PATIENT to an urgent Cancer PATIENT PATHWAY.	
The pseudonymised ORGANISATION SITE CODE of the Organisation acting as Health Care Provider when a decision is made to upgrade the PATIENT to an urgent Cancer PATIENT PATHWAY.	
The date that the PATIENT is first seen in the Trust that receives the first referral.	
Month first seen	
Year first seen	
Option to provide number of days from a different event to the date first seen (e.g. days from date first seen to diagnosis)	
The ORGANISATION SITE CODE of the Health Care Provider at the first contact with the PATIENT.	
The pseudonymised ORGANISATION SITE CODE of the Health Care Provider at the first contact with the PATIENT.	
The number of days that should be removed from the derived waiting time between the CANCER REFERRAL TO TREATMENT PERIOD START DATE and DATE FIRST SEEN.	
**MULTIDISCIPLINARY TEAM ACTIVITY**<br>To carry Multidisciplinary Team Activity details.<br>One occurrence of this group is required.	
An indication of whether the PATIENT's CARE PLAN was discussed at a Multidisciplinary Team Meeting.	
The date on which the PATIENT's Cancer Care Plan was discussed at a Multidisciplinary Team Meeting and a treatment planning decision was made.	
Month of MDT discussion	
Year of MDT discussion	
Option to provide number of days from a different event to the MDT discussion date (e.g. days from MDT discussion date to diagnosis)	
**PATIENT STATUS AND DIAGNOSIS**<br>To carry Patient Status and Diagnosis details.<br>One occurrence of this group is required.	
Recorded to enable tracking of the status of REFERRAL REQUESTS for PATIENTS referred with a suspected cancer, or referred with breast symptoms with cancer not originally suspected.	
The International Classification of Diseases (ICD) code used to identify the PRIMARY DIAGNOSIS.	
The METASTATIC SITE at the point of treatment.	
The side of the body for a Tumour relating to paired organs within a PATIENT.	
The DATE when a Cancer Treatment Period is started.	
Month of cancer treatment period	
Year of cancer treatment period	
Option to provide number of days from a different event to the cancer treatment period start date (e.g. days from diagnosis to cancer treatment period start date)	
The ORGANISATION SITE CODE of the Organisation acting as Health Care Provider where the decision to treat the PATIENT was made which initiated a Cancer Care Plan with one or more Planned Cancer Treatments.	
The pseudonymised ORGANISATION SITE CODE of the Organisation acting as Health Care Provider where the decision to treat the PATIENT was made which initiated a Cancer Care Plan with one or more Planned Cancer Treatments.	
The date the Organisation where the PATIENT is being transferred to, received the SERVICE REQUEST from the referring Organisation.	
Month of referral request received	

Year of referral request received	
Option to provide number of days from a different event to the referral request received date (e.g. days from the referral request received date to diagnosis)	
**FORMATION ABOUT PATIENT PATHWAY AND DIAGNOSIS**	
GP practice code for the patient's registered GP practice at the treatment period start date (according to a patient trace)	
Pseudonymised GP practice code for the patient's registered GP practice at the treatment period start date (according to a patient trace)	
Broad cancer site grouping for the primary diagnosis recorded in the CWT data	
Specific cancer site grouping for the primary diagnosis recorded in the CWT data	
Indicator of a possible duplicate CWT treatment record	
For first treatment records, indicator for whether the cancer diagnosis resulted from an urgent GP referral for suspected cancer (sometimes known as 'two week wait referral') for the same broad type of suspected cancer as the recorded diagnosis	
For first treatment records, indicator for whether the cancer diagnosis resulted from an urgent GP referral for suspected cancer (sometimes known as 'two week wait referral') for the any type of suspected cancer	
**TREATMENT EVENTS** To carry Treatment Event details. rrence of this group is required if applicable to the scenario.	
The Start Date of the first, second or subsequent cancer treatment given to a PATIENT who is receiving care for a cancer condition, with a PRIMARY DIAGNOSIS (ICD) code within the range C00 to C97 or D05 as defined by NHS England.	
Month treatment started	
Year treatment started	
Option to provide number of days from a different event to the treatment start date (e.g. days from diagnosis to the treatment start date)	
The ORGANISATION SITE CODE of the Organisation where the TREATMENT START DATE FOR CANCER is recorded.	
The pseudonymised ORGANISATION SITE CODE of the Organisation where the TREATMENT START DATE FOR CANCER is recorded.	
The stage of treatment reached during a Cancer PATIENT PATHWAY for primary, recurrent or metastatic cancer.	
The type of treatment or care which was delivered in a Cancer Treatment Period.	
An indicator recording whether an individual episode of care is being delivered to a PATIENT as part of a CLINICAL TRIAL.	
The type of care setting where the cancer care relating to the TREATMENT START DATE FOR CANCER took place.	
The intent of the delivered beam radiation for PATIENTS with a cancer PRIMARY DIAGNOSIS (ICD), as defined by NHS England.	
The priority for the Radiotherapy Treatment Course as classified by the requesting clinician.	
The number of days that should be removed from the derived waiting time between CANCER TREATMENT PERIOD START DATE and TREATMENT START DATE FOR CANCER.	

Justification

For peer review only

er review only

For peer review only

Introduction

The **National Bowel Cancer Audit (NBOCA)** was established to describe and
established and the 2015 Annual Report includes data on over 30,000 patients
The audit is commissioned by the Healthcare Quality Improvement Partnership
Clinical Audit Support Unit within the Health and Social Care Information Centr
The 2015 annual report will be published in December 2015; this includes data

Inclusion:
The audit includes all new diagnoses of primary bowel cancer within the currer

- Aged 18 or older at the date of diagnosis
- Diagnosed in the audit period (April-March)
- Diagnosed with colorectal cancer, with one of the follow ICD10 codes C18-C2

Exclusion:

- Recurrent cancer – the audit only reports on primary cancer diagnoses
- Primary cancer without a colorectal site
- Sarcomas
- B cell lymphoma
- Neuroendocrine / Carcinoid tumours
- Cloacogenic carcinoma
- Melanoma

Coverage

The audit follows patients diagnosed between:
01/04/2006 - 31/03/2013 (the vital status of each patient can be followed up wi

Data item selection

Please be aware that selecting patient identifiable data items – **the data items**
covering your organisation. Sensitive patient demographic data, event dates ar
3 anonymity. Privacy by design and default principles have also been applied t
below:

Key:

**Items in red**	Identifiable fields that require additional permissions to acce
	Sensitive items that require additional justification in your pı
	Privacy by design and default' alternatives to the sensitive i

Item number	Name
**1**	Pseudonymised patient ID
**2**	**NHSNumber**
**3**	**DateOfBirth**
**4**	DateOfBirth_YYYY
**5**	DateOfBirth_MM
**6**	Days from a number of options
**7**	**Surname**
**8**	**Forename**
**9**	**Postcode (at diagnosis)**
**10**	Postcode (at diagnosis) - 1st part of postcode
**11**	Gender

12	OrganisationFirstSeen
13	Pseudonymised code for OrganisationFirstSeen
14	DateOfDiagnosis
15	DateOfDiagnosis_YYYY
16	DateOfDiagnosis_mm
17	Days from a different event to date of diagnosis
18	SourceOfReferral
19	ClinicalNurseSpecialistIndication
20	ICD10MajorSite
21	SynchronousCancer
22	PerformanceStatus
23	CPEXAnaerobicThreshold
24	CarePlanIntent
25	PlannedCancerTreatmentType
26	NoCancerTreatmentReason
27	TCategory
28	NCategory
29	MCategory
30	MonitoringIntent
31	ProviderOrganisation
32	ProviderOrganisation (pseudonymised)
33	ASAGrade
34	CancerTreatmentCurability
35	CPEXAnaerobicThreshold
36	BMI
37	DateOfSurgery
38	DateOfSurgery_YYYY
39	DateOfSurgery_MM
40	Days from a different event to date of surgery
41	SurgicalUrgencyModeOfOperation
42	**Consultant code**

For peer review only

**43**	Consultant code (pseudonymised)
**44**	PrimaryProcedure
**45**	SurgicalAccess
**46**	RoboticSurgery
**47**	ImmediatePostoperativeCare
**48**	StatusOfCircumferentialExcisionMargin
**49**	NumberOfNodesExamined
**50**	NumberOfNodesPositive
**51**	FinalPathologyTCategory
**52**	FinalPathologyNCategory
**53**	FinalPathologyRadiologyMCategory
**54**	SizeOfSpecimen
**55**	TumourType
**56**	DifferentiationByWorstArea
**57**	LocalInvasionPolypoidTumours
**58**	LocalInvasionSessileTumours
**59**	CancerVascularOrLymphaticInvasion
**60**	PreOpInitialProviderOrganisation
**61**	PreOpInitialProviderOrganisation (pseudonymised)
**62**	PreOpInitialCancerTreatmentModality
**63**	PostOpProviderOrganisation
**64**	PostOpProviderOrganisation (pseudonymised)
**65**	PostOpCancerTreatmentModality

************************* **End of sheet** *************************

For peer review only

For p

For p

For p

For p

For p

For p

For peer review only

d compare the diagnosis, care and outcomes of patients diagnosed with bowel cancer in Engla
s. The Audit's overall aim is to measure the quality of care and survival of patients with bowel
o (HQIP), has been developed by the Association of Coloproctology of Great Britain and Irelar
re on behalf of the Clinical Effectiveness Unit (CEU) of the Royal College of Surgeons of Engl
a on patients diagnosed from 1 April 2013 to 31 March 2014.In 2016 annual report will be publ

nt reporting period. Included patients must be;

20;

th linkage to Cancer Registration data)

s marked below in red – will require you to have patient consent, section 251 approval, or the
nd geographies will be treated as de-personalised, unless anonymisation techniques are appl
to some of these data to provide a less disclosive alternative. The key explaining how these a

ess (as outlined above)
rotocol to access
items highlighted in dark grey

Description	Request Data Item
**Patient**	
PATIENTID	
**Mandatory Field. Must be valid number - those without a valid number (e.g. non-English nationals) should have had a temporary NHS number created.**	
**Mandatory Field. dd/mm/yyyy format**	
Mandatory Field. yyyy format - year	
Mandatory Field. mm format - month	
Option to provide number of days from a selected date	
**That part of a person's name which is used to describe family, clan, tribal group, or marital association.**	
**The forename or given name of a person.**	
**A person's postcode at diagnosis.**	
A person's postcode at diagnosis - first part of postcode	
A person's gender at time of diagnosis	
**Tumour**	

The ORGANISATION SITE CODE of the Health Care Provider at the first contact with the PATIENT.	
The pseudonymised ORGANISATION SITE CODE of the Health Care Provider at the first contact with the PATIENT.	
Record the date when cancer was confirmed or diagnosis agreed. This will typically be the date of the pathology report which confirms the cancer.	
Date of Diagnosis - year	
Date of Diagnosis - month	
Option to provide number of days from a selected date	
Record the source of referral of a patient referred to secondary care for the investigation of suspected cancer.	
Record if and when the patient saw an appropriate site specific clinical nurse specialist.	
Record the diagnosis that represents the main cancer site for which the patient is receiving care	
Record any synchronous tumours. Multiple responses are possible. Synchronous tumours are identified as discrete tumours apparently not in continuity with other primary cancers originating in the same site or tissue	
The patient's performance status, to be the value presented at the MDT meeting prior to the beginning of treatment.	
Record the anaerobic threshold. Value to be between 0-99 with 1 decimal place.	
The intention of the treatment being planned.	
The treatment(s) that are planned for the patient	
The reason no treatment(s) are planned for the patient	
The 'T' part of the TNM classification used to describe the clinico-radiological stage of the tumour prior to treatment. 5th edition of TNM is used for colorectal cancer	
The 'N' part of the TNM classification used to describe the clinico-radiological stage of the tumour prior to treatment. 5th edition of TNM is used for colorectal cancer	
The 'M' part of the TNM classification used to describe the clinico-radiological stage of the tumour prior to treatment. 5th edition of TNM is used for colorectal cancer	
The purpose of monitoring a patient. Note: in the Cancer Outcomes and Services Dataset this may only be used for First Definitive Treatment.	
**Surgery**	
Surgery Provider Organisation Code	
Pseudonymised Surgery Provider Organisation Code	
Record the ASA Grade as determined at the time of surgery. Data item used for risk adjustment	
The surgeons' opinion of the completeness of the excision based on clinical findings at the time of operation which should not be revised in the light of subsequent histopathology reporting. Curative means no local tumour was visibly present after the surgical procedure. Palliative means that visible tumour was left behind after the surgical resection	
Record the anaerobic threshold. Value to be between 0-99 with 1 decimal place.	
Estimate of a patient's Body Mass Index (BMI) at diagnosis.	
Record the date when the primary procedure took place.	
Record the date when the primary procedure took place. - year	
Record the date when the primary procedure took place. - month	
Option to provide number of days from a selected date	
Record the mode of surgery performed	
**The General Medical Council (GMC) Code for the consultant who was actually in overall charge of the patient's surgical treatment during the time the patient was in hospital undergoing surgery.**	

Pseudonymised GMC code for consultant	
Record the procedure that best describes resection of the primary colorectal cancer.	
Record the access used to perform the operation	
Record any use of the robot in the operation	
Site of discharge form theatre recovery	
**Pathology**	
Record if the edge of the tumour is 1 mm or less from the circumferential resection margin (i.e. margin involved) Circumferential margin refers to the completeness of the surgeon's resection margin in the opinion of the histopathologist. In parts of the colon where it is completely surrounded by peritoneum, recording of the circumferential resection margin (CRM) is not appropriate.	
The number of local and regional nodes examined.	
The number of local and regional nodes reported as being positive for the presence of Tumour metastases.	
T CATEGORY (PATHOLOGICAL) is the Union for International Cancer Control (UICC) code which classifies the size and extent of the primary Tumour based on the evidence from a pathological examination.	
N CATEGORY (PATHOLOGICAL) is the Union for International Cancer Control (UICC) code which classifies the absence or presence and extent of regional lymph node metastases based on the evidence from a pathological examination.	
For 97% of patients the M stage is a radiological NOT a pathological judgement.<br>M CATEGORY (PATHOLOGICAL) is the Union for International Cancer Control (UICC) code which classifies the absence or presence of distant metastases based on the evidence from a pathological examination.	
The size in millimetres of the diameter of a lesion, largest if more than one, if the histology of a SAMPLE proves to be invasive.	
Tumour Type from pathology report e.g., Adenocarcinoma	
The definitive grade of the Tumour based on the evidence from a pathological examination.	
For local excision specimens<br>Polypoid tumours - Haggitt level	
For local excision specimens<br>Sessile tumours - Kikuchi level	
An indication of the presence or absence of unequivocal tumour in lymphatic and/or vascular spaces.	
**Chemoradiotherapy**	
Chemoradiotherapy Provider Organisation Code	
Pseudonymised Pre-op Chemoradiotherapy Provider Organisation Code	
The type of treatment or care which was delivered in a Cancer Treatment Period. This is the first oncology therapy given, or where applicable that give BEFORE Surgical resection of the primary colorectal cancer.	
Chemoradiotherapy Provider Organisation Code	
Pseudonymised Post-op Chemoradiotherapy Provider Organisation Code	
The type of treatment or care which was delivered in a Cancer Treatment Period. This is oncology therapy given AFTER Surgical resection of the primary colorectal cancer.	

For peer review only - http://bmjopen.bmj.com/site/about/guidelines.xhtml

peer review only

and and Wales; it is now well
cancer in England and Wales.
nd (ACPGBI) and is managed by the
land.
plished in summer 2016.

e appropriate statutory regulation
lied to render the data anonymised to k-
ilternative can be selected is included

Justification

peer review only

For peer review only

For peer review only

For peer review only

peer review only

For peer review only

For peer review only

For peer review only

For peer review only

For peer review only

For peer review only

For peer review only

For peer review only

Introduction

The Quality of Life of Cancer Survivors in England: Pilot Survey (2011) was cor
The survey was conducted by Quality Health in conjunction with cancer registri
prostate cancer and non-Hodgkin's lymphoma (NHL)) and at four different time
Responses were obtained (n=3300) of individuals approach to participant, givin
Assessment of Cancer Therapy) items and SDI (Social Difficulties Inventory).
https://www.gov.uk/government/uploads/system/uploads/attachment_data/file/2

The survey was repeated in 2012 for the same cohort of patients, with an 85%

Data Coverage

Routinely linked to incident cancer cases from July 2006 - July 2010

Data items selection:

Please be aware that selecting patient identifiable data items – the data items r
Sensitive patient demographic data, event dates and geographies will be treate
principles have also been applied to some of these data to provide a less disclo

Key:

**Items in red**	Identifiable fields that require additional permissions to access (
	Sensitive items that require additional justification in your protoc
	Privacy by design and default' alternatives to the sensitive items

Breast cancer PROMS

Question number	Question text
	The questions in this
N/A	Pseudonymised patient ID
01a	What treatments have you received for your breast cancer?
01b	What treatments have you received for your breast cancer?
01c	What treatments have you received for your breast cancer?
01d	What treatments have you received for your breast cancer?
2	How long is it since you completed your initial treatment for breast cancer?
3	How has your breast cancer responded to treatment?
04a	If you have had breast surgery, do any of the following apply to you?
04b	If you have had breast surgery, do any of the following apply to you?
04c	If you have had breast surgery, do any of the following apply to you?
04d	If you have had breast surgery, do any of the following apply to you?
04e	If you have had breast surgery, do any of the following apply to you?
5	Mobility (NCSI)
6	Self-Care (NCSI)

**7**	Usual Activities (NCSI)
**8**	Pain / Discomfort (NCIS)
**9**	Anxiety / Depression
	The ques
**10**	I have been short of breath.
**11**	I am self conscious about the way I dress.
**12**	One or both of my arms are swollen or tender.
**13**	I feel sexually attractive.
**14**	I am bothered by hair loss.
**15**	I worry that other members of my family might someday get the same illness I have.
**16**	I worry about the effect of stress on my illness.
**17**	I am bothered by a change in weight.
**18**	I have certain parts of my body where I experience pain.
**19**	In the past week, on how many days have you done a total of 30 minutes or more of physical activity, which was enough to raise your heart rate?
	The questic
**20**	Have you had any difficulty in maintaining your independence?
**21**	Have you had any difficulty in carrying out your domestic chores?
**22**	Have you had any difficulty with managing your own personal care?
**23**	Have you had any difficulty with looking after those who depend on you?
**24**	Have any of those close to you (e.g. partner, children, parents) had any difficulty with the support available to them?
**25**	Have you had any difficulty with benefits?
**26**	Have you had any financial difficulties?
**27**	Have you had any difficulty with financial services?
**28**	Have you had any difficulty concerning your work?
**29**	Have you had any difficulty with planning for your own or your family's future?
**30**	Have you had any difficulty with communicating with those closest to you?
**31**	Have you had any difficulty with communicating with others?
**32**	Have you had any difficulty concerning sexual matters?
**33**	Have you had any difficulty concerning plans to have a family?
**34**	Have you had any difficulty concerning your appearance or body image?
**35**	Have you felt isolated?
**36**	Have you had any difficulty with getting around?
**37**	Have you had any difficulty with where you live?
**38**	Have you had any difficulty in carrying out your recreational activities?
**39**	Have you had any difficulty with your plans to travel or take a holiday?
**40**	Have you had any difficulty with any other area of your everyday life?
**41**	I have fears about my cancer spreading.

For peer review only

**42**	I have fears about my cancer coming back.
**43**	I have fears about death and dying.
**44**	I experience memory loss.
**45**	I have trouble sleeping.
**46**	I have trouble concentrating.
**47**	I always feel tired.
**48**	I experience mood swings.
**49**	I am often irritable.
**50**	Do you have an up-to-date written care plan?
**51**	Do you have a named nurse who you can contact if you have a worry about your cancer care?
**52**	Do you know who to contact if you have a concern about any aspect of living with or after cancer?
**53**	Do you think that hospital staff did everything they could to support you following your cancer treatment?
**54**	Do you think that GPs and nurses at your general practice do everything they can to support you following your cancer treatment?
**55**	Following your initial cancer treatment have you been given enough care and help from health and social services (for example, district nurses, home helps or occupational therapists)?
**56a**	Would it have been helpful to have had more advice or information on any of the following issues:
**56b**	Would it have been helpful to have had more advice or information on any of the following issues:
**56c**	Would it have been helpful to have had more advice or information on any of the following issues:
**56d**	Would it have been helpful to have had more advice or information on any of the following issues:
**56e**	Would it have been helpful to have had more advice or information on any of the following issues:
**56f**	Would it have been helpful to have had more advice or information on any of the following issues:
**56g**	Would it have been helpful to have had more advice or information on any of the following issues:
**56h**	Would it have been helpful to have had more advice or information on any of the following issues:
**56i**	Would it have been helpful to have had more advice or information on any of the following issues:
	If you are helping someon
**57**	What year were you born?
**58**	Are you male or female?
**59**	**Which of the following best describes your sexual orientation?**
**60**	Which statement best describes your living arrangements?
**61**	Do you have a long standing health condition? (NCSI)
**62a**	Which, if any, of the following conditions do you have?
**62b**	Which, if any, of the following conditions do you have?
**62c**	Which, if any, of the following conditions do you have?
**62d**	Which, if any, of the following conditions do you have?
**62e**	Which, if any, of the following conditions do you have?
**62f**	Which, if any, of the following conditions do you have?
**62g**	Which, if any, of the following conditions do you have?
**62h**	Which, if any, of the following conditions do you have?
**62i**	Which, if any, of the following conditions do you have?
**62j**	Which, if any, of the following conditions do you have?
**62k**	Which, if any, of the following conditions do you have?

For peer review only

**62l**	Which, if any, of the following conditions do you have?
**62m**	Which, if any, of the following conditions do you have?
**62n**	Which, if any, of the following conditions do you have?
**62o**	Which, if any, of the following conditions do you have?
**62p**	Which, if any, of the following conditions do you have?
**62q**	Which, if any, of the following conditions do you have?
**62r**	Which, if any, of the following conditions do you have?
**63**	What was your employment status before you were diagnosed with cancer?
**64**	What is your employment status currently?
**65**	If you are currently employed at the moment, are you:
**66a**	To which of these ethnic groups would you say you belong?
**66b**	Broad ethnic group
**66_Other**	**To which of these ethnic groups would you say you belong?**

Colorectal cancer PROMS

**Question number**	**Question text**
	The questions in this
**N/A**	Pseudonymised patient ID
**01a**	What treatments have you received for your colorectal / GI cancer?
**01b**	What treatments have you received for your colorectal / GI cancer?
**01c**	What treatments have you received for your colorectal / GI cancer?
**2**	How long is it since you completed your initial treatment for colorectal / GI cancer?
**3**	How has your colorectal / GI cancer responded to treatment?
**4**	If you have a stoma (e.g. colostomy) is it:
**5**	Mobility (NCSI)
**6**	Self-Care (NCSI)
**7**	Usual Activities (NCSI)
**8**	Pain / Discomfort (NCIS)
**9**	Anxiety / Depression
	The ques
**10**	I have swelling or cramps in my stomach area.

For peer review only

**11**	I am losing weight.
**12**	I have control of my bowels.
**13**	I can digest my food well.
**14**	I have diarrhoea.
**15**	I have a good appetite.
**16**	I like the appearance of my body.
**17**	I have difficulty urinating.
**18**	I urinate more frequently than usual.
**19**	I leak urine.
**20**	Do you have an ostomy appliance / stoma?
**21**	I am embarrassed by my ostomy appliance / stoma.
**22**	Caring for my ostomy appliance / stoma is difficult.
**23**	Do you have any difficulty in controlling your bowels (e.g. any accidents)?
**24**	If yes, how often do you have difficulties?
**25**	In the past week, on how many days have you done a total of 30 minutes or more of physical activity, which was enough to raise your heart rate?
	The questic
**26**	Have you had any difficulty in maintaining your independence?
**27**	Have you had any difficulty in carrying out your domestic chores?
**28**	Have you had any difficulty with managing your own personal care?
**29**	Have you had any difficulty with looking after those who depend on you?
**30**	Have any of those close to you (e.g. partner, children, parents) had any difficulty with the support available to them?
**31**	Have you had any difficulty with benefits?
**32**	Have you had any financial difficulties?
**33**	Have you had any difficulty with financial services?
**34**	Have you had any difficulty concerning your work?
**35**	Have you had any difficulty with planning for your own or your family's future?
**36**	Have you had any difficulty with communicating with those closest to you?
**37**	Have you had any difficulty with communicating with others?
**38**	Have you had any difficulty concerning sexual matters?
**39**	Have you had any difficulty concerning plans to have a family?
**40**	Have you had any difficulty concerning your appearance or body image?
**41**	Have you felt isolated?
**42**	Have you had any difficulty with getting around?
**43**	Have you had any difficulty with where you live?
**44**	Have you had any difficulty in carrying out your recreational activities?
**45**	Have you had any difficulty with your plans to travel or take a holiday?
**46**	Have you had any difficulty with any other area of your everyday life?
**47**	I have fears about my cancer spreading.
**48**	I have fears about my cancer coming back.
**49**	I have fears about death and dying.
**50**	I experience memory loss.
**51**	I have trouble sleeping.

For peer review only

**52**	I have trouble concentrating.
**53**	I always feel tired.
**54**	I experience mood swings.
**55**	I am often irritable.
**56**	Do you have an up-to-date written care plan?
**57**	Do you have a named nurse who you can contact if you have a worry about your cancer care?
**58**	Do you know who to contact if you have a concern about any aspect of living with or after cancer?
**59**	Do you think that hospital staff did everything they could to support you following your cancer treatment?
**60**	Do you think that GPs and nurses at your general practice do everything they can to support you following your cancer treatment?
**61**	Following your initial cancer treatment have you been given enough care and help from health and social services (for example, district nurses, home helps or occupational therapists)?
**62a**	Would it have been helpful to have had more advice or information on any of the following issues:
**62b**	Would it have been helpful to have had more advice or information on any of the following issues:
**62c**	Would it have been helpful to have had more advice or information on any of the following issues:
**62d**	Would it have been helpful to have had more advice or information on any of the following issues:
**62e**	Would it have been helpful to have had more advice or information on any of the following issues:
**62f**	Would it have been helpful to have had more advice or information on any of the following issues:
**62g**	Would it have been helpful to have had more advice or information on any of the following issues:
**62h**	Would it have been helpful to have had more advice or information on any of the following issues:
**62i**	Would it have been helpful to have had more advice or information on any of the following issues:
	If you are helping someon
**63**	What year were you born?
**64**	Are you male or female?
**65**	**Which of the following best describes your sexual orientation?**
**66**	Which statement best describes your living arrangements?
**67**	Do you have a long standing health condition? (NCSI)
**68a**	Which, if any, of the following conditions do you have?
**68b**	Which, if any, of the following conditions do you have?
**68c**	Which, if any, of the following conditions do you have?
**68d**	Which, if any, of the following conditions do you have?
**68e**	Which, if any, of the following conditions do you have?
**68f**	Which, if any, of the following conditions do you have?
**68g**	Which, if any, of the following conditions do you have?
**68h**	Which, if any, of the following conditions do you have?
**68i**	Which, if any, of the following conditions do you have?
**68j**	Which, if any, of the following conditions do you have?
**68k**	Which, if any, of the following conditions do you have?
**68l**	Which, if any, of the following conditions do you have? Learning difficulty
**68m**	Which, if any, of the following conditions do you have?
**68n**	Which, if any, of the following conditions do you have?
**68o**	Which, if any, of the following conditions do you have?
**68p**	Which, if any, of the following conditions do you have?

For peer review only

**68q**	Which, if any, of the following conditions do you have?
**68r**	Which, if any, of the following conditions do you have?
**69**	What was your employment status before you were diagnosed with cancer?
**70**	What is your employment status currently?
**71**	If you are currently employed at the moment, are you:
**66a**	To which of these ethnic groups would you say you belong?
**66b**	Broad ethnic group
**72_Other**	**To which of these ethnic groups would you say you belong?**

Non-Hodgkin's Lymphoma PROMS

Question number	Question text
	The questions in this
**N/A**	Pseudonymised patient ID
**01a**	What treatments have you received for your Non Hodgkin's Lymphoma (NHL)?
**01b**	What treatments have you received for your Non Hodgkin's Lymphoma (NHL)?
**01c**	What treatments have you received for your Non Hodgkin's Lymphoma (NHL)? Surgery
**01d**	What treatments have you received for your Non Hodgkin's Lymphoma (NHL)?
**01e**	What treatments have you received for your Non Hodgkin's Lymphoma (NHL)? Stem cell transplant
**2**	How long is it since you completed your initial treatment for NHL?
**3**	How has your NHL responded to treatment?
**4**	Mobility (NCSI)
**5**	Self-Care (NCSI)
**6**	Usual Activities (NCSI)
**7**	Pain / Discomfort (NCIS)
**8**	Anxiety / Depression
	The ques
**9**	I have certain parts of my body where I experience pain.
**10**	I am bothered by lumps or swelling in certain parts of my body.

**11**	I am bothered by fevers.
**12**	I have night sweats.
**13**	I am bothered by itching.
**14**	I have trouble sleeping at night.
**15**	I get tired easily.
**16**	I am losing weight.
**17**	I have a loss of appetite.
**18**	I have trouble concentrating [7 days]
**19**	I worry about getting infections.
**20**	I worry that I might get new symptoms of my illness.
**21**	I feel isolated from others because of my illness or treatment.
**22**	I have emotional ups and downs.
**23**	Because of my illness, I have difficulty planning for the future.
**24**	In the past week, on how many days have you done a total of 30 minutes or more of physical activity, which was enough to raise your heart rate?
	The questic
**25**	Have you had any difficulty in maintaining your independence?
**26**	Have you had any difficulty in carrying out your domestic chores?
**27**	Have you had any difficulty with managing your own personal care?
**28**	Have you had any difficulty with looking after those who depend on you?
**29**	Have any of those close to you (e.g. partner, children, parents) had any difficulty with the support available to them?
**30**	Have you had any difficulty with benefits?
**31**	Have you had any financial difficulties?
**32**	Have you had any difficulty with financial services?
**33**	Have you had any difficulty concerning your work?
**34**	Have you had any difficulty with planning for your own or your family's future?
**35**	Have you had any difficulty with communicating with those closest to you?
**36**	Have you had any difficulty with communicating with others?
**37**	Have you had any difficulty concerning sexual matters?
**38**	Have you had any difficulty concerning plans to have a family?
**39**	Have you had any difficulty concerning your appearance or body image?
**40**	Have you felt isolated?
**41**	Have you had any difficulty with getting around?
**42**	Have you had any difficulty with where you live?
**43**	Have you had any difficulty in carrying out your recreational activities?
**44**	Have you had any difficulty with your plans to travel or take a holiday?
**45**	Have you had any difficulty with any other area of your everyday life?
**46**	I have fears about my cancer spreading.
**47**	I have fears about my cancer coming back.
**48**	I have fears about death and dying.
**49**	I experience memory loss.
**50**	I have trouble sleeping.

For peer review only

**51**	I have trouble concentrating.
**52**	I always feel tired.
**53**	I experience mood swings.
**54**	I am often irritable.
**55**	Do you have an up-to-date written care plan?
**56**	Do you have a named nurse who you can contact if you have a worry about your cancer care?
**57**	Do you know who to contact if you have a concern about any aspect of living with or after cancer?
**58**	Do you think that hospital staff did everything they could to support you following your cancer treatment?
**59**	Do you think that GPs and nurses at your general practice do everything they can to support you following your cancer treatment?
**60**	Following your initial cancer treatment have you been given enough care and help from health and social services (for example, district nurses, home helps or occupational therapists)?
**61a**	Would it have been helpful to have had more advice or information on any of the following issues:
**61b**	Would it have been helpful to have had more advice or information on any of the following issues:
**61c**	Would it have been helpful to have had more advice or information on any of the following issues: Financial help or benefits
**61d**	Would it have been helpful to have had more advice or information on any of the following issues: Free prescriptions
**61e**	Would it have been helpful to have had more advice or information on any of the following issues:
**61f**	Would it have been helpful to have had more advice or information on any of the following issues:
**61g**	Would it have been helpful to have had more advice or information on any of the following issues:
**61h**	Would it have been helpful to have had more advice or information on any of the following issues:
**61i**	Would it have been helpful to have had more advice or information on any of the following issues:
	If you are helping someone
**62**	What year were you born?
**63**	Are you male or female?
**64**	**Which of the following best describes your sexual orientation?**
**65**	Which statement best describes your living arrangements?
**66**	Do you have a long standing health condition? (NCSI)
**67a**	Which, if any, of the following conditions do you have?
**67b**	Which, if any, of the following conditions do you have?
**67c**	Which, if any, of the following conditions do you have?
**67d**	Which, if any, of the following conditions do you have?
**67e**	Which, if any, of the following conditions do you have?
**67f**	Which, if any, of the following conditions do you have?
**67g**	Which, if any, of the following conditions do you have?
**67h**	Which, if any, of the following conditions do you have?
**67i**	Which, if any, of the following conditions do you have?
**67j**	Which, if any, of the following conditions do you have?
**67k**	Which, if any, of the following conditions do you have?
**67l**	Which, if any, of the following conditions do you have?
**67m**	Which, if any, of the following conditions do you have? Liver disease

For peer review

**67n**	Which, if any, of the following conditions do you have?
**67o**	Which, if any, of the following conditions do you have?
**67p**	Which, if any, of the following conditions do you have?
**67q**	Which, if any, of the following conditions do you have?
**67r**	Which, if any, of the following conditions do you have?
**68**	What was your employment status before you were diagnosed with cancer?
**69**	What is your employment status currently?
**70**	If you are currently employed at the moment, are you:
**66a**	To which of these ethnic groups would you say you belong?
**66b**	Broad ethnic group
**71_Other**	**To which of these ethnic groups would you say you belong?**

Prostate cancer PROMS

**Question number**	**Question text**
	The questions in this
**N/A**	Pseudonymised patient ID
**01a**	What treatments have you received for your prostate cancer?
**01b**	What treatments have you received for your prostate cancer?
**01c**	What treatments have you received for your prostate cancer?
**01d**	What treatments have you received for your prostate cancer?
**01e**	What treatments have you received for your prostate cancer?
**01f**	What treatments have you received for your prostate cancer?
**01g**	What treatments have you received for your prostate cancer?
**2**	How long is it since you completed your initial treatment for prostate cancer?
**3**	How has your prostate cancer responded to treatment?
**4**	Mobility (NCSI)
**5**	Self-Care (NCSI)
**6**	Usual Activities (NCSI)
**7**	Pain / Discomfort (NCIS)

For peer review only

**8**	Anxiety / Depression
	The ques
**9**	I am losing weight.
**10**	I have a good appetite.
**11**	I have aches and pains that bother me
**12**	I have certain parts of my body where I experience pain.
**13**	My pain keeps me from doing things I want to do
**14**	I am satisfied with my present comfort level
**15**	I have trouble moving my bowels
**16**	I have difficulty urinating.
**17**	I urinate more frequently than usual.
**18**	My problems with urinating limit my activities
**19**	I am able to have and maintain an erection
**20**	I leak urine.
**21**	Do you have any difficulty in controlling your bowels (e.g. any accidents)?
**22**	If yes, how often do you have difficulties?
**23**	In the past week, on how many days have you done a total of 30 minutes or more of physical activity, which was enough to raise your heart rate?
	The questio
**24**	Have you had any difficulty in maintaining your independence?
**25**	Have you had any difficulty in carrying out your domestic chores?
**26**	Have you had any difficulty with managing your own personal care?
**27**	Have you had any difficulty with looking after those who depend on you?
**28**	Have any of those close to you (e.g. partner, children, parents) had any difficulty with the support available to them?
**29**	Have you had any difficulty with benefits?
**30**	Have you had any financial difficulties?
**31**	Have you had any difficulty with financial services?
**32**	Have you had any difficulty concerning your work?
**33**	Have you had any difficulty with planning for your own or your family's future?
**34**	Have you had any difficulty with communicating with those closest to you?
**35**	Have you had any difficulty with communicating with others?
**36**	Have you had any difficulty concerning sexual matters?
**37**	Have you had any difficulty concerning plans to have a family?
**38**	Have you had any difficulty concerning your appearance or body image?
**39**	Have you felt isolated?
**40**	Have you had any difficulty with getting around?
**41**	Have you had any difficulty with where you live?
**42**	Have you had any difficulty in carrying out your recreational activities?
**43**	Have you had any difficulty with your plans to travel or take a holiday?
**44**	Have you had any difficulty with any other area of your everyday life?
**45**	I have fears about my cancer spreading.

For peer review only

**46**	I have fears about my cancer coming back.
**47**	I have fears about death and dying.
**48**	I experience memory loss.
**49**	I have trouble sleeping.
**50**	I have trouble concentrating.
**51**	I always feel tired.
**52**	I experience mood swings.
**53**	I am often irritable.
**54**	Do you have an up-to-date written care plan?
**55**	Do you have a named nurse who you can contact if you have a worry about your cancer care?
**56**	Do you know who to contact if you have a concern about any aspect of living with or after cancer?
**57**	Do you think that hospital staff did everything they could to support you following your cancer treatment?
**58**	Do you think that GPs and nurses at your general practice do everything they can to support you following your cancer treatment?
**59**	Following your initial cancer treatment have you been given enough care and help from health and social services (for example, district nurses, home helps or occupational therapists)?
**60a**	Would it have been helpful to have had more advice or information on any of the following issues:
**60b**	Would it have been helpful to have had more advice or information on any of the following issues:
**60c**	Would it have been helpful to have had more advice or information on any of the following issues:
**60d**	Would it have been helpful to have had more advice or information on any of the following issues:
**60e**	Would it have been helpful to have had more advice or information on any of the following issues:
**60f**	Would it have been helpful to have had more advice or information on any of the following issues:
**60g**	Would it have been helpful to have had more advice or information on any of the following issues:
**60h**	Would it have been helpful to have had more advice or information on any of the following issues:
**60i**	Would it have been helpful to have had more advice or information on any of the following issues:
	If you are helping someon
**61**	What year were you born?
**62**	Are you male or female?
**63**	**Which of the following best describes your sexual orientation?**
**64**	Which statement best describes your living arrangements?
**65**	Do you have a long standing health condition? (NCSI)
**66a**	Which, if any, of the following conditions do you have?
**66b**	Which, if any, of the following conditions do you have?
**66c**	Which, if any, of the following conditions do you have?
**66d**	Which, if any, of the following conditions do you have?
**66e**	Which, if any, of the following conditions do you have?
**66f**	Which, if any, of the following conditions do you have?
**66g**	Which, if any, of the following conditions do you have?
**66h**	Which, if any, of the following conditions do you have?
**66i**	Which, if any, of the following conditions do you have?
**66j**	Which, if any, of the following conditions do you have? High blood pressure
**66k**	Which, if any, of the following conditions do you have?

For peer review only - http://bmjopen.bmj.com/site/about/guidelines.xhtml

**66l**	Which, if any, of the following conditions do you have?
**66m**	Which, if any, of the following conditions do you have? Liver disease
**66n**	Which, if any, of the following conditions do you have? Long term back problems
**66o**	Which, if any, of the following conditions do you have? Long-standing mental health problem
**66p**	Which, if any, of the following conditions do you have?
**66q**	Which, if any, of the following conditions do you have?
**66r**	Which, if any, of the following conditions do you have?
**67**	What was your employment status before you were diagnosed with cancer?
**68**	What is your employment status currently?
**69**	If you are currently employed at the moment, are you:
**66a**	To which of these ethnic groups would you say you belong?
**66b**	Broad ethnic group
**70_Other**	**To which of these ethnic groups would you say you belong?**
	*************************** **End of sheet** ***************************

For peer review only

mmissioned by the Department of Health as part of the National Cancer Survivorship Initiative (NCSI).
ies. The survey measures overall quality of life of representative samples of cancer survivors with four different
points after diagnosis (approximately one, two, three or five years).
ng a response rate of 66%. Outcome questions in the survey are made up of three instruments: the EQ-5D (Eur

267042/9284-TSO-2900701-PROMS-1.pdf

response rate

narked below in red – will require you to have patient consent, section 251 approval, or the appropriate statutor
ed as de-personalised, unless anonymisation techniques are applied to render the data anonymised to k-3 anor
osive alternative. The key explaining how these alternative can be selected is included below:

(as outlined above)
col to access
s highlighted in dark grey

Possible answers	Request Data Item
**Your health today**<br>s section are all about your health and how you feel on the day you complete this questionnaire.	
PATIENTID	
Radiotherapy	
Chemotherapy (excluding hormone treatment)	
Hormone treatment	
Surgery	
I am still having my initial treatment, It is less than 3 months since my initial treatment, It is between 3 and 12 months since my initial treatment, It is between 1 and 5 years since my initial treatment, It is more than 5 years since my initial treatment, Don't know / can't remember	
My breast cancer has responded fully to treatment (I am in remission), My breast cancer has been treated but is still present, My breast cancer has not been treated at all, My breast cancer has come back after it was originally treated, I am not certain what is happening with my breast cancer	
I have had a lumpectomy	
I have had a mastectomy	
I have had breast reconstruction	
I am awaiting or considering breast reconstruction	
None of these apply to me	
I have no problems in walking about, I have slight problems in walking about, I have moderate problems in walking about, I have severe problems in walking about, I am unable to walk about	
I have no problems washing or dressing myself, I have slight problems washing or dressing myself, I have moderate problems washing or dressing myself, I have severe problems washing or dressing myself, I am unable to wash or dress myself	

I have no problems doing my usual activities, I have slight problems doing my usual activities, I have moderate problems doing my usual activities, I have severe problems doing my usual activities, I am unable to do my usual activities	
I have no pain or discomfort, I have slight pain or discomfort, I have moderate pain or discomfort, I have severe pain or discomfort, I have extreme pain or discomfort	
I am not anxious or depressed, I am slightly anxious or depressed, I am moderately anxious or depressed, I am severely anxious or depressed, I am extremely anxious or depressed	
**Your health and wellbeing in the last 7 days**	
stions in this section are about your health and how you have felt in the past 7 days.	
Not at all, A little bit, Somewhat, Quite a bit, Very much	
Not at all, A little bit, Somewhat, Quite a bit, Very much	
Not at all, A little bit, Somewhat, Quite a bit, Very much	
Not at all, A little bit, Somewhat, Quite a bit, Very much	
Not at all, A little bit, Somewhat, Quite a bit, Very much	
Not at all, A little bit, Somewhat, Quite a bit, Very much	
Not at all, A little bit, Somewhat, Quite a bit, Very much	
Not at all, A little bit, Somewhat, Quite a bit, Very much	
Not at all, A little bit, Somewhat, Quite a bit, Very much	
None, 1 day, 2 days, 3 days, 4 days, 5 days, 6 days, 7 days	
**Your health and wellbeing in the last month**	
ons in this section are about your health and how you have felt during the past month.	
No difficulty, A little, Quite a bit, Very much, Does not apply	
No difficulty, A little, Quite a bit, Very much, Does not apply	
No difficulty, A little, Quite a bit, Very much, Does not apply	
No difficulty, A little, Quite a bit, Very much, Does not apply	
No difficulty, A little, Quite a bit, Very much, Does not apply	
No difficulty, A little, Quite a bit, Very much, Does not apply	
No difficulty, A little, Quite a bit, Very much, Does not apply	
No difficulty, A little, Quite a bit, Very much, Does not apply	
No difficulty, A little, Quite a bit, Very much, Does not apply	
No difficulty, A little, Quite a bit, Very much, Does not apply	
No difficulty, A little, Quite a bit, Very much, Does not apply	
No difficulty, A little, Quite a bit, Very much, Does not apply	
No difficulty, A little, Quite a bit, Very much, Does not apply	
No difficulty, A little, Quite a bit, Very much, Does not apply	
No difficulty, A little, Quite a bit, Very much, Does not apply	
No difficulty, A little, Quite a bit, Very much, Does not apply	
No difficulty, A little, Quite a bit, Very much, Does not apply	
No difficulty, A little, Quite a bit, Very much, Does not apply	
No difficulty, A little, Quite a bit, Very much, Does not apply	
No difficulty, A little, Quite a bit, Very much, Does not apply	
No difficulty, A little, Quite a bit, Very much, Does not apply	
Strongly Agree, Agree, Neither agree nor disagree, Disagree, Strongly disagree, Does not apply to me	

For peer review only

Strongly Agree, Agree, Neither agree nor disagree, Disagree, Strongly disagree, Does not apply to me	
Strongly Agree, Agree, Neither agree nor disagree, Disagree, Strongly disagree	
Strongly Agree, Agree, Neither agree nor disagree, Disagree, Strongly disagree	
Strongly Agree, Agree, Neither agree nor disagree, Disagree, Strongly disagree	
Strongly Agree, Agree, Neither agree nor disagree, Disagree, Strongly disagree	
Strongly Agree, Agree, Neither agree nor disagree, Disagree, Strongly disagree	
Strongly Agree, Agree, Neither agree nor disagree, Disagree, Strongly disagree	
Strongly Agree, Agree, Neither agree nor disagree, Disagree, Strongly disagree	
**Overall support and care**	
Yes definitely, Yes I think so, No, I do not need a care plan, Don't know	
Yes, No, Don't know	
Yes definitely, Yes I think so, No	
Yes all of the time, Only some of the time, Never, I did not need any support	
Yes all of the time, Only some of the time, Never, My general practice is not involved, I do not need any support	
Yes definitely, Yes to some extent, No, I did not need help from health or social services, Don't know / can't remember	
Diet and lifestyle	
Physical activity and exercise	
Financial help or benefits	
Free prescriptions	
Returning to or staying in work	
Information / advice for family / friends / carer	
The physical aspects of living with and after cancer	
The psychological or emotional aspects of living with and after cancer	
I have all the information and advice I need	
**About you**<br>e to complete this questionnaire, please make sure this information is the patient's and not your own.	
In format YYYY	
Male, Female	
**Heterosexual / straight (opposite sex), Bisexual (both sexes), Gay or Lesbian (same sex), Other, Prefer not to answer**	
I live with partner / spouse / family / friends, I live alone, I live in a nursing home hospital or other long term care home, Other	
Yes, No, Don't know / can't say	
Alzheimer's disease or dementia	
Angina	
Arthritis	
Asthma or other chronic chest problem	
Blindness or visual impairment	
Deafness or hearing impairment	
Diabetes	
Epilepsy	
Heart condition	
High blood pressure	
Kidney disease	

For peer review only

Learning difficulty	
Liver disease	
Long term back problems	
Long-standing mental health problem	
Long-standing neurological problem	
Another long-standing condition	
I do not have any of these conditions	
Full time employment, Part time employment, Homemaker, Student (in education), Retired, Unemployed – and seeking work, Unemployed – unable to work for health reasons, Other	
Full time employment, Part time employment, Homemaker, Student (in education), Retired, Unemployed – and seeking work, Unemployed – unable to work for health reasons, Other	
Not working at all, Working less hours than usual, Working your usual hours, Working more hours than usual, This question does not apply to me	
British, Irish, Any other White background, White and Black Caribbean, White and Black African, White and Asian, Any other mixed background, Indian, Pakistani, Bangladeshi, Any other Asian background, Caribbean, African, Any other Black background, Chinese, Any other ethnic group	
Option to group ethnicities (e.g. white/ non-white/ unknown)	
**Free text**	

**Possible answers**	**Request Data Item**
**Your health today**<br>s section are all about your health and how you feel on the day you complete this questionnaire.	
PATIENTID	
Radiotherapy	
Chemotherapy	
Surgery	
I am still having my initial treatment, It is less than 3 months since my initial treatment, It is between 3 and 12 months since my initial treatment, It is between 1 and 5 years since my initial treatment, It is more than 5 years since my initial treatment, Don't know / can't remember	
My colorectal / GI cancer has responded fully to treatment (I am in remission), My colorectal / GI cancer has been treated but is still present, My colorectal / GI cancer has not been treated at all, My colorectal / GI cancer has come back after it was originally treated, I am not certain what is happening with my colorectal / GI cancer	
Still present, Reversed, This does not apply to me	
I have no problems in walking about, I have slight problems in walking about, I have moderate problems in walking about, I have severe problems in walking about, I am unable to walk about	
I have no problems washing or dressing myself, I have slight problems washing or dressing myself, I have moderate problems washing or dressing myself, I have severe problems washing or dressing myself, I am unable to wash or dress myself	
I have no problems doing my usual activities, I have slight problems doing my usual activities, I have moderate problems doing my usual activities, I have severe problems doing my usual activities, I am unable to do my usual activities	
I have no pain or discomfort, I have slight pain or discomfort, I have moderate pain or discomfort, I have severe pain or discomfort, I have extreme pain or discomfort	
I am not anxious or depressed, I am slightly anxious or depressed, I am moderately anxious or depressed, I am severely anxious or depressed, I am extremely anxious or depressed	
**Your health and wellbeing in the last 7 days**<br>stions in this section are about your health and how you have felt in the past 7 days.	
Not at all, A little bit, Somewhat, Quite a bit, Very much	

Not at all, A little bit, Somewhat, Quite a bit, Very much	
Not at all, A little bit, Somewhat, Quite a bit, Very much	
Not at all, A little bit, Somewhat, Quite a bit, Very much	
Not at all, A little bit, Somewhat, Quite a bit, Very much	
Not at all, A little bit, Somewhat, Quite a bit, Very much	
Not at all, A little bit, Somewhat, Quite a bit, Very much	
Not at all, A little bit, Somewhat, Quite a bit, Very much	
Not at all, A little bit, Somewhat, Quite a bit, Very much	
Not at all, A little bit, Somewhat, Quite a bit, Very much	
No, Yes	
Not at all, A little bit, Somewhat, Quite a bit, Very much	
Not at all, A little bit, Somewhat, Quite a bit, Very much	
No, Yes	
Monthly, Weekly, Daily, Constantly, It varies	
None, 1 day, 2 days, 3 days, 4 days, 5 days, 6 days, 7 days	
**Your health and wellbeing in the last month**<br>ons in this section are about your health and how you have felt during the past month.	
No difficulty, A little, Quite a bit, Very much, Does not apply	
No difficulty, A little, Quite a bit, Very much, Does not apply	
No difficulty, A little, Quite a bit, Very much, Does not apply	
No difficulty, A little, Quite a bit, Very much, Does not apply	
No difficulty, A little, Quite a bit, Very much, Does not apply	
No difficulty, A little, Quite a bit, Very much, Does not apply	
No difficulty, A little, Quite a bit, Very much, Does not apply	
No difficulty, A little, Quite a bit, Very much, Does not apply	
No difficulty, A little, Quite a bit, Very much, Does not apply	
No difficulty, A little, Quite a bit, Very much, Does not apply	
No difficulty, A little, Quite a bit, Very much, Does not apply	
No difficulty, A little, Quite a bit, Very much, Does not apply	
No difficulty, A little, Quite a bit, Very much, Does not apply	
No difficulty, A little, Quite a bit, Very much, Does not apply	
No difficulty, A little, Quite a bit, Very much, Does not apply	
No difficulty, A little, Quite a bit, Very much, Does not apply	
No difficulty, A little, Quite a bit, Very much, Does not apply	
No difficulty, A little, Quite a bit, Very much, Does not apply	
No difficulty, A little, Quite a bit, Very much, Does not apply	
No difficulty, A little, Quite a bit, Very much, Does not apply	
No difficulty, A little, Quite a bit, Very much, Does not apply	
Strongly Agree, Agree, Neither agree nor disagree, Disagree, Strongly disagree, Does not apply to me	
Strongly Agree, Agree, Neither agree nor disagree, Disagree, Strongly disagree, Does not apply to me	
Strongly Agree, Agree, Neither agree nor disagree, Disagree, Strongly disagree	
Strongly Agree, Agree, Neither agree nor disagree, Disagree, Strongly disagree	
Strongly Agree, Agree, Neither agree nor disagree, Disagree, Strongly disagree	

For peer review only

Strongly Agree, Agree, Neither agree nor disagree, Disagree, Strongly disagree	
Strongly Agree, Agree, Neither agree nor disagree, Disagree, Strongly disagree	
Strongly Agree, Agree, Neither agree nor disagree, Disagree, Strongly disagree	
Strongly Agree, Agree, Neither agree nor disagree, Disagree, Strongly disagree	
**Overall support and care**	
Yes definitely, Yes I think so, No, I do not need a care plan, Don't know	
Yes, No, Don't know	
Yes definitely, Yes I think so, No	
Yes all of the time, Only some of the time, Never, I did not need any support	
Yes all of the time, Only some of the time, Never, My general practice is not involved, I do not need any support	
Yes definitely, Yes to some extent, No, I did not need help from health or social services, Don't know / can't remember	
Diet and lifestyle	
Physical activity and exercise	
Financial help or benefits	
Free prescriptions	
Returning to or staying in work	
Information / advice for family / friends / carer	
The physical aspects of living with and after cancer	
The psychological or emotional aspects of living with and after cancer	
I have all the information and advice I need	
**About you**	
e to complete this questionnaire, please make sure this information is the patient's and not your own.	
In format YYYY	
Male, Female	
**Heterosexual / straight (opposite sex), Bisexual (both sexes), Gay or Lesbian (same sex), Other, Prefer not to answer**	
I live with partner / spouse / family / friends, I live alone, I live in a nursing home hospital or other long term care home, Other	
Yes, No, Don't know / can't say	
Alzheimer's disease or dementia	
Angina	
Arthritis	
Asthma or other chronic chest problem	
Blindness or visual impairment	
Deafness or hearing impairment	
Diabetes	
Epilepsy	
Heart condition	
High blood pressure	
Kidney disease	
Learning difficulty	
Liver disease	
Long term back problems	
Long-standing mental health problem	
Long-standing neurological problem	

For peer review only

Another long-standing condition	
I do not have any of these conditions	
Full time employment, Part time employment, Homemaker, Student (in education), Retired, Unemployed – and seeking work, Unemployed – unable to work for health reasons, Other	
Full time employment, Part time employment, Homemaker, Student (in education), Retired, Unemployed – and seeking work, Unemployed – unable to work for health reasons, Other	
Not working at all, Working less hours than usual, Working your usual hours, Working more hours than usual, This question does not apply to me	
British, Irish, Any other White background, White and Black Caribbean, White and Black African, White and Asian, Any other mixed background, Indian, Pakistani, Bangladeshi, Any other Asian background, Caribbean, African, Any other Black background, Chinese, Any other ethnic group	
Option to group ethnicities (e.g. white/ non-white/ unknown)	
Free text	

Possible answers	Request Data Item
**Your health today**<br>s section are all about your health and how you feel on the day you complete this questionnaire.	
PATIENTID	
Radiotherapy	
Chemotherapy	
Surgery	
Antibody therapy	
Stem cell transplant	
I am still having my initial treatment, It is less than 3 months since my initial treatment, It is between 3 and 12 months since my initial treatment, It is between 1 and 5 years since my initial treatment, It is more than 5 years since my initial treatment, Don't know / can't remember	
My NHL has responded fully to treatment (I am in remission), My NHL has been treated but is still present, My NHL has not been treated at all, My NHL has come back after it was originally treated, I am not certain what is happening with my NHL	
I have no problems in walking about, I have slight problems in walking about, I have moderate problems in walking about, I have severe problems in walking about, I am unable to walk about	
I have no problems washing or dressing myself, I have slight problems washing or dressing myself, I have moderate problems washing or dressing myself, I have severe problems washing or dressing myself, I am unable to wash or dress myself	
I have no problems doing my usual activities, I have slight problems doing my usual activities, I have moderate problems doing my usual activities, I have severe problems doing my usual activities, I am unable to do my usual activities	
I have no pain or discomfort, I have slight pain or discomfort, I have moderate pain or discomfort, I have severe pain or discomfort, I have extreme pain or discomfort	
I am not anxious or depressed, I am slightly anxious or depressed, I am moderately anxious or depressed, I am severely anxious or depressed, I am extremely anxious or depressed	
**Your health and wellbeing in the last 7 days**<br>stions in this section are about your health and how you have felt in the past 7 days.	
Not at all, A little bit, Somewhat, Quite a bit, Very much	
Not at all, A little bit, Somewhat, Quite a bit, Very much	

Not at all, A little bit, Somewhat, Quite a bit, Very much	
Not at all, A little bit, Somewhat, Quite a bit, Very much	
Not at all, A little bit, Somewhat, Quite a bit, Very much	
Not at all, A little bit, Somewhat, Quite a bit, Very much	
Not at all, A little bit, Somewhat, Quite a bit, Very much	
Not at all, A little bit, Somewhat, Quite a bit, Very much	
Not at all, A little bit, Somewhat, Quite a bit, Very much	
Not at all, A little bit, Somewhat, Quite a bit, Very much	
Not at all, A little bit, Somewhat, Quite a bit, Very much	
Not at all, A little bit, Somewhat, Quite a bit, Very much	
Not at all, A little bit, Somewhat, Quite a bit, Very much	
Not at all, A little bit, Somewhat, Quite a bit, Very much	
Not at all, A little bit, Somewhat, Quite a bit, Very much	
None, 1 day, 2 days, 3 days, 4 days, 5 days, 6 days, 7 days	
**Your health and wellbeing in the last month**<br>ons in this section are about your health and how you have felt during the past month.	
No difficulty, A little, Quite a bit, Very much, Does not apply	
No difficulty, A little, Quite a bit, Very much, Does not apply	
No difficulty, A little, Quite a bit, Very much, Does not apply	
No difficulty, A little, Quite a bit, Very much, Does not apply	
No difficulty, A little, Quite a bit, Very much, Does not apply	
No difficulty, A little, Quite a bit, Very much, Does not apply	
No difficulty, A little, Quite a bit, Very much, Does not apply	
No difficulty, A little, Quite a bit, Very much, Does not apply	
No difficulty, A little, Quite a bit, Very much, Does not apply	
No difficulty, A little, Quite a bit, Very much, Does not apply	
No difficulty, A little, Quite a bit, Very much, Does not apply	
No difficulty, A little, Quite a bit, Very much, Does not apply	
No difficulty, A little, Quite a bit, Very much, Does not apply	
No difficulty, A little, Quite a bit, Very much, Does not apply	
No difficulty, A little, Quite a bit, Very much, Does not apply	
No difficulty, A little, Quite a bit, Very much, Does not apply	
No difficulty, A little, Quite a bit, Very much, Does not apply	
No difficulty, A little, Quite a bit, Very much, Does not apply	
No difficulty, A little, Quite a bit, Very much, Does not apply	
No difficulty, A little, Quite a bit, Very much, Does not apply	
No difficulty, A little, Quite a bit, Very much, Does not apply	
Strongly Agree, Agree, Neither agree nor disagree, Disagree, Strongly disagree, Does not apply to me	
Strongly Agree, Agree, Neither agree nor disagree, Disagree, Strongly disagree, Does not apply to me	
Strongly Agree, Agree, Neither agree nor disagree, Disagree, Strongly disagree	
Strongly Agree, Agree, Neither agree nor disagree, Disagree, Strongly disagree	
Strongly Agree, Agree, Neither agree nor disagree, Disagree, Strongly disagree	

For peer review only

Strongly Agree, Agree, Neither agree nor disagree, Disagree, Strongly disagree	
Strongly Agree, Agree, Neither agree nor disagree, Disagree, Strongly disagree	
Strongly Agree, Agree, Neither agree nor disagree, Disagree, Strongly disagree	
Strongly Agree, Agree, Neither agree nor disagree, Disagree, Strongly disagree	
**Overall support and care**	
Yes definitely, Yes I think so, No, I do not need a care plan, Don't know	
Yes, No, Don't know	
Yes definitely, Yes I think so, No	
Yes all of the time, Only some of the time, Never, I did not need any support	
Yes all of the time, Only some of the time, Never, My general practice is not involved, I do not need any support	
Yes definitely, Yes to some extent, No, I did not need help from health or social services, Don't know / can't remember	
Diet and lifestyle	
Physical activity and exercise	
Financial help or benefits	
Free prescriptions	
Returning to or staying in work	
Information / advice for family / friends / carer	
The physical aspects of living with and after cancer	
The psychological or emotional aspects of living with and after cancer	
I have all the information and advice I need	
**About you**<br>e to complete this questionnaire, please make sure this information is the patient's and not your own.	
In format YYYY	
Male, Female	
**Heterosexual / straight (opposite sex), Bisexual (both sexes), Gay or Lesbian (same sex), Other, Prefer not to answer**	
I live with partner / spouse / family / friends, I live alone, I live in a nursing home hospital or other long term care home, Other	
Yes, No, Don't know / can't say	
Alzheimer's disease or dementia	
Angina	
Arthritis	
Asthma or other chronic chest problem	
Blindness or visual impairment	
Deafness or hearing impairment	
Diabetes	
Epilepsy	
Heart condition	
High blood pressure	
Kidney disease	
Learning difficulty	
Liver disease	

For peer review

Long term back problems	
Long-standing mental health problem	
Long-standing neurological problem	
Another long-standing condition	
I do not have any of these conditions	
Full time employment, Part time employment, Homemaker, Student (in education), Retired, Unemployed – and seeking work, Unemployed – unable to work for health reasons, Other	
Full time employment, Part time employment, Homemaker, Student (in education), Retired, Unemployed – and seeking work, Unemployed – unable to work for health reasons, Other	
Not working at all, Working less hours than usual, Working your usual hours, Working more hours than usual, This question does not apply to me	
British, Irish, Any other White background, White and Black Caribbean, White and Black African, White and Asian, Any other mixed background, Indian, Pakistani, Bangladeshi, Any other Asian background, Caribbean, African, Any other Black background, Chinese, Any other ethnic group	
Option to group ethnicities (e.g. white/ non-white/ unknown)	
**Free text**	

**Possible answers**	**Request Data Item**
**Your health today**<br>s section are all about your health and how you feel on the day you complete this questionnaire.	
PATIENTID	
Radiotherapy	
Surgery	
Hormone Treatment	
Chemotherapy	
HIFU	
Cryotherapy	
Active surveillance	
I am still having my initial treatment, It is less than 3 months since my initial treatment, It is between 3 and 12 months since my initial treatment, It is between 1 and 5 years since my initial treatment, It is more than 5 years since my initial treatment, Don't know / can't remember	
My prostate cancer has responded fully to treatment (I am in remission), My prostate cancer has been treated but is still present, My prostate cancer has not been treated at all, My prostate cancer has come back after it was originally treated, I am not certain what is happening with my prostate cancer	
I have no problems in walking about, I have slight problems in walking about, I have moderate problems in walking about, I have severe problems in walking about, I am unable to walk about	
I have no problems washing or dressing myself, I have slight problems washing or dressing myself, I have moderate problems washing or dressing myself, I have severe problems washing or dressing myself, I am unable to wash or dress myself	
I have no problems doing my usual activities, I have slight problems doing my usual activities, I have moderate problems doing my usual activities, I have severe problems doing my usual activities, I am unable to do my usual activities	
I have no pain or discomfort, I have slight pain or discomfort, I have moderate pain or discomfort, I have severe pain or discomfort, I have extreme pain or discomfort	

I am not anxious or depressed, I am slightly anxious or depressed, I am moderately anxious or depressed, I am severely anxious or depressed, I am extremely anxious or depressed	
**Your health and wellbeing in the last 7 days**<br>stions in this section are about your health and how you have felt in the past 7 days.	
Not at all, A little bit, Somewhat, Quite a bit, Very much	
Not at all, A little bit, Somewhat, Quite a bit, Very much	
Not at all, A little bit, Somewhat, Quite a bit, Very much	
Not at all, A little bit, Somewhat, Quite a bit, Very much	
Not at all, A little bit, Somewhat, Quite a bit, Very much	
Not at all, A little bit, Somewhat, Quite a bit, Very much	
Not at all, A little bit, Somewhat, Quite a bit, Very much	
Not at all, A little bit, Somewhat, Quite a bit, Very much	
Not at all, A little bit, Somewhat, Quite a bit, Very much	
Not at all, A little bit, Somewhat, Quite a bit, Very much	
Not at all, A little bit, Somewhat, Quite a bit, Very much	
Not at all, A little bit, Somewhat, Quite a bit, Very much	
No, Yes	
Monthly, Weekly, Daily, Constantly, It varies	
None, 1 day, 2 days, 3 days, 4 days, 5 days, 6 days, 7 days	
**Your health and wellbeing in the last month**<br>ons in this section are about your health and how you have felt during the past month.	
No difficulty, A little, Quite a bit, Very much, Does not apply	
No difficulty, A little, Quite a bit, Very much, Does not apply	
No difficulty, A little, Quite a bit, Very much, Does not apply	
No difficulty, A little, Quite a bit, Very much, Does not apply	
No difficulty, A little, Quite a bit, Very much, Does not apply	
No difficulty, A little, Quite a bit, Very much, Does not apply	
No difficulty, A little, Quite a bit, Very much, Does not apply	
No difficulty, A little, Quite a bit, Very much, Does not apply	
No difficulty, A little, Quite a bit, Very much, Does not apply	
No difficulty, A little, Quite a bit, Very much, Does not apply	
No difficulty, A little, Quite a bit, Very much, Does not apply	
No difficulty, A little, Quite a bit, Very much, Does not apply	
No difficulty, A little, Quite a bit, Very much, Does not apply	
No difficulty, A little, Quite a bit, Very much, Does not apply	
No difficulty, A little, Quite a bit, Very much, Does not apply	
No difficulty, A little, Quite a bit, Very much, Does not apply	
No difficulty, A little, Quite a bit, Very much, Does not apply	
No difficulty, A little, Quite a bit, Very much, Does not apply	
No difficulty, A little, Quite a bit, Very much, Does not apply	
No difficulty, A little, Quite a bit, Very much, Does not apply	
No difficulty, A little, Quite a bit, Very much, Does not apply	
Strongly Agree, Agree, Neither agree nor disagree, Disagree, Strongly disagree, Does not apply to me	

For peer review only

Strongly Agree, Agree, Neither agree nor disagree, Disagree, Strongly disagree, Does not apply to me	
Strongly Agree, Agree, Neither agree nor disagree, Disagree, Strongly disagree	
Strongly Agree, Agree, Neither agree nor disagree, Disagree, Strongly disagree	
Strongly Agree, Agree, Neither agree nor disagree, Disagree, Strongly disagree	
Strongly Agree, Agree, Neither agree nor disagree, Disagree, Strongly disagree	
Strongly Agree, Agree, Neither agree nor disagree, Disagree, Strongly disagree	
Strongly Agree, Agree, Neither agree nor disagree, Disagree, Strongly disagree	
Strongly Agree, Agree, Neither agree nor disagree, Disagree, Strongly disagree	
**Overall support and care**	
Yes definitely, Yes I think so, No, I do not need a care plan, Don't know	
Yes, No, Don't know	
Yes definitely, Yes I think so, No	
Yes all of the time, Only some of the time, Never, I did not need any support	
Yes all of the time, Only some of the time, Never, My general practice is not involved, I do not need any support	
Yes definitely, Yes to some extent, No, I did not need help from health or social services, Don't know / can't remember	
Diet and lifestyle	
Physical activity and exercise	
Financial help or benefits	
Free prescriptions	
Returning to or staying in work	
Information / advice for family / friends / carer	
The physical aspects of living with and after cancer	
The psychological or emotional aspects of living with and after cancer	
I have all the information and advice I need	
**About you**	
e to complete this questionnaire, please make sure this information is the patient's and not your own.	
In format YYYY	
Male, Female	
**Heterosexual / straight (opposite sex), Bisexual (both sexes), Gay or Lesbian (same sex), Other, Prefer not to answer**	
I live with partner / spouse / family / friends, I live alone, I live in a nursing home hospital or other long term care home, Other	
Yes, No, Don't know / can't say	
Alzheimer's disease or dementia	
Angina	
Arthritis	
Asthma or other chronic chest problem	
Blindness or visual impairment	
Deafness or hearing impairment	
Diabetes	
Epilepsy	
Heart condition	
High blood pressure	
Kidney disease	

For peer review only

Learning difficulty	
Liver disease	
Long term back problems	
Long-standing mental health problem	
Long-standing neurological problem	
Another long-standing condition	
I do not have any of these conditions	
Full time employment, Part time employment, Homemaker, Student (in education), Retired, Unemployed – and seeking work, Unemployed – unable to work for health reasons, Other	
Full time employment, Part time employment, Homemaker, Student (in education), Retired, Unemployed – and seeking work, Unemployed – unable to work for health reasons, Other	
Not working at all, Working less hours than usual, Working your usual hours, Working more hours than usual, This question does not apply to me	
British, Irish, Any other White background, White and Black Caribbean, White and Black African, White and Asian, Any other mixed background, Indian, Pakistani, Bangladeshi, Any other Asian background, Caribbean, African, Any other Black background, Chinese, Any other ethnic group	
Option to group ethnicities (e.g. white/ non-white/ unknown)	

For peer review only

tumour types (breast, colorectal and

roqol 5 level), FACT (Functional

ry regulation covering your organisation.
ıymity. Privacy by design and default

Justification

ew only

For peer review only

For peer review only

Justification

view only

For peer review only

Justification

For peer review only

For peer review only

For peer review only

Justification

For peer review only

For peer review only

For peer review only

Introduction

The **National Lung Cancer Audit (LUCADA)** looks at the care delivered during refe
compiled to meet the requirements of audit, and are not to be confused with the data
the primary treatment package, assessing against standards and bringing about nec
as it emerges.

Coverage: The audit follows patients diagnosed between: 01/01/2015 - 31/12/2013

Data item selection

Please be aware that selecting patient identifiable data items – **the data items mark**
Sensitive patient demographic data, event dates and geographies will be treated as
have also been applied to some of these data to provide a less disclosive alternative

Key:

Items in red Identifiable fields that require additional permissions to access (as out
Sensitive items that require additional justification in your protocol to a
Privacy by design and default' alternatives to the sensitive items highli

Item number	Data Item
1	Pseudonymised patient ID
2	Patient identifier
3	**NHS Number**
4	**Surname**
5	**Forenames**
6	**Postcode at diagnosis**
7	Postcode at diagnosis - 1st part of postcode
8	Sex
9	**Date of birth**
10	Date of birth - year
11	Date of birth - month
12	Age at diagnosis
13	Age at diagnosis in 5 year age bands (0-4 etc.)
14	Organisation code of the trust which provided the data
15	Pseudonymised organisation code of trust providing data
16	Registry Code
	This s
17	Source of referral (for cancer)
18	Cancer referral decision date
19	Cancer referral decision date - year
20	Cancer referral decision date - month
21	Days from a different date to cancer referral decision date
22	Date first seen
23	Date first seen - year
24	Date first seen - month
25	Days from a different date to date first seen
26	NCDS Lung Cancer Specialist Referral Date
27	NCDS Lung Cancer Specialist Referral Date - year
28	NCDS Lung Cancer Specialist Referral Date - month
29	Days from a different date to date of specialist referral
30	Place first seen
31	Pseudonymised place first seen
This section should be used to record details of the lung cancer diagnosis. If the ma	
32	Date of diagnosis
33	Date of diagnosis - year
34	Date of diagnosis - month
35	Days from a different date to date of diagnosis

36	Place of diagnosis
37	Pseudonymised code of place of diagnosis
38	Primary site diagnosis
39	Laterality
40	Basis of diagnosis
The TNM staging data items record the definitive pre-treatment TNM stage. TNM cancer care plan meeting prior to treatment and based on the clinical and patholo	
41	TNM Classification Version Number
42	Staging M category
43	Staging N category
44	Staging T category
45	T category (Final pre treatment)
46	N category (Final pre treatment)
47	M category (Final pre treatment)
48	Pre-treatment stage group
49	Site-specific classification
50	Pre Treatment Histology
51	Lung cancer morphology
52	Did the patient have a staging procedure
53	Did patient have mediastinal sampling
54	Did the patient receive mediastinoscopy or mediastinomy
55	Did the patient have an FNA
56	Did the patient have any other staging procedure
57	Staging procedure performed but method unknown
Following a diagnosis of cancer, a cancer care plan will be drawn up for the patient's	
58	MDT discussion indicator
59	MDT discussion date
60	MDT discussion date - year
61	MDT discussion date - month
62	Days from a different date to date of MDT discussion
63	Cancer care plan intent
64	Care plan agreed date
65	Care plan agreed date- year
66	Care plan agreed date - month
67	Days from a different date to date the care plan was agreed
68	Intention of the treatment which is planned for the patient at this point in time.
69	Type of chemotherapy treatment discussed at the MDT
70	FEV1 (Absolute)
71	FEV1 (Percentage of predicted)
72	Performance status
If a pat	
73	Any significant comorbidity which prevented original treatment plan discussed at the MDT
If co-morbidity is selected as the rea	
74	Dementia/Cerebrovascular disease
75	Cardiovascular disease
76	Renal failure
77	Other malignancy
78	Severe weight loss
79	Other significant co-morbidity
80	The date comorbidity was recorded
81	The date comorbidity - year
82	The date comorbidity - month
83	Days from a different date to date the comorbidity was recorded

This section describes the key investigations used in establishing a diagnosis of lun	
**84**	Was a CT scan performed
**85**	Date of CT scan
**86**	Date of CT scan - year
**87**	Date of CT scan - month
**88**	Days from a different date to date of CT scan
**89**	Was a PET / PET CT scan performed
**90**	Date of PET / PET CT scan
**91**	Date of PET / PET CT scan - year
**92**	Date of PET / PET CT scan - month
**93**	Days from a different date to date of PET / PET CT scan
**94**	Was a bronchoscopy performed
**95**	Date of bronchoscopy
**96**	Date of bronchoscopy - year
**97**	Date of bronchoscopy - mm
**98**	Days from a different date to date of bronchoscopy
**99**	Was a CT guided biopsy performed
**100**	Date of CT guided biopsy
**101**	Date of CT guided biopsy - year
**102**	Date of CT guided biopsy - month
**103**	Days from a different date to date of CT guided biopsy
**104**	Was an other diagnostic biopsy performed
**105**	Date of other diagnostic biopsy
**106**	Date of other diagnostic biopsy - year
**107**	Date of other diagnostic biopsy - month
**108**	Days from a different date to date of other diagnostic biopsy
This section is about the input of the Lung Cancer Nurse Specialist into the lung ca receives the	
**109**	Assessed by lung cancer nurse specialist
**110**	Date of assessment by lung cancer nurse specialist
**111**	Date of assessment by lung cancer nurse specialist - year
**112**	Date of assessment by lung cancer nurse specialist - month
**113**	Days from a different date to date of assessment by CNS
**114**	How was the patient assessed by lung cancer nurse specialist
**115**	At what stage (s) in the patient journey was the patient assessed by lung cancer nurse specialist
**116**	Lung cancer nurse specialist present when patient received diagnosis
Use this	
**117**	Clinical Trial Status
**118**	Planned treatment type
**119**	Planned treatment
For Lung Cancer patients (including clinical diagnoses) Record here any surgical pro	
**120**	Hospital site code (of surgery)
**121**	Pseudonymised hospital site code (of surgery)
**122**	Date of decision to operate (surgery)
**123**	Date of decision to operate (surgery) - year
**124**	Date of decision to operate (surgery) - month
**125**	Days from a different date to date of decision to operate
**126**	Date of surgery
**127**	Date of surgery - year
**128**	Date of surgery - month
**129**	Days from a different date to date of surgery
**130**	Main surgical procedure (OPSC)

Recorded here are details relating to the completeness of the surgical resection a	
131	Excision margins – R Classification
132	Path Stage T
133	Path Stage N
134	Path Stage M
135	T category (Pathological)
136	N category(Pathological)
137	M category (Pathological)
138	Overall Pathological TNM Stage grouping
139	Investigation results date (Date Specimen Reported)
140	Investigation results date (Date Specimen Reported) - year
141	Investigation results date (Date Specimen Reported) - month
142	Days from a different date to investigation results date
143	Histology
144	Site-specific classification
145	Hospital site code (of chemotherapy)
146	Pseudonymised hospital site code (of chemotherapy)
147	Date of decision to treat
148	Date of decision to treat - year
149	Date of decision to treat - month
150	Days from a different date to date of decision to treat
151	Drug treatment start date
152	Drug treatment start date - year
153	Drug treatment start date - month
154	Days from a different date to drug treatment start date
155	Chemotherapy treatment given
156	Hospital site code (of teletherapy)
157	Pseudonymised hospital site code (of teletherapy)
158	Date of decision to treat
159	Date of decision to treat - year
160	Date of decision to treat - month
161	Days from a different date to date of decision to treat
162	Radiotherapy treatment given
163	Radiotherapy anatomical treatment site
164	Teletherapy start date
165	Teletherapy start date - year
166	Teletherapy start date - month
167	Days from a different date to teletherapy start date
168	Hospital site code (of brachytherapy)
169	Pseudonymised hospital site code (of brachytherapy)
170	Date of decision to treat (of brachytherapy)
171	Date of decision to treat (of brachytherapy) - year
172	Date of decision to treat (of brachytherapy) - month
173	Days from a different date to date of decision to treat
174	Start date (brachytherapy treatment course)
175	Start date (brachytherapy treatment course) - year
176	Start date (brachytherapy treatment course) - month
177	Days from a different date to brachytherapy start date
178	Site code (palliative treatment course)
179	Pseudonymised site code (palliative treatment course)
180	Palliative care provider type
181	Palliative care community provider type
182	Date of decision to treat (palliative care)
183	Date of decision to treat (palliative care) - year

**184**	Date of decision to treat (palliative care) - month
**185**	Days from a different date to date of decision to treat
**186**	Specialist Palliative Care Start Date
**187**	Specialist Palliative Care Start Date - year
**188**	Specialist Palliative Care Start Date - month
**189**	Days from a different date to palliative care start date
**190**	Palliative care interventional treatment given
**191**	Date interventional treatment given
**192**	Date interventional treatment given - year
**193**	Date interventional treatment given - month
**194**	Days from a different date to date of interventional treatment
**195**	Hospital site code (of active monitoring)
**196**	Pseudonymised hospital site code (of active monitoring)
**197**	Decision to treat date (active monitoring)
**198**	Decision to treat date (active monitoring) - year
**199**	Decision to treat date (active monitoring) - month
**200**	Days from a different date to date of decision to treat
**200**	**Date of death**
**201**	Date of death - year
**202**	Date of death - month
**203**	Days from a different date to date of death
**204**	Was Death Related to Treatment?
**205**	Treatment Related Morbidity Type
**206**	Patient received prophylactic cranial radiation.
**207**	Was the original treatment plan carried out
**208**	Reason why original treatment plan failed

************************* **End of sheet** *************************

For peer review only

ɜrral, diagnosis, treatment and outcomes for people diagnosed with lung cancer and mesothelioma. The data ite
a items identified as Lung Cancer in the National Cancer dataset. The audit focuses on measuring the care give
ɛessary improvements. The project supports the Calman Hine recommendations, the National Cancer Plan and

(the vital status of each patient can be followed up with linkage to Cancer Registration data)

ked below in red – will require you to have patient consent, section 251 approval, or the appropriate statutory re
de-personalised, unless anonymisation techniques are applied to render the data anonymised to k-3 anonymity
ɛ. The key explaining how these alternative can be selected is included below:

ɪlined above)
ɑccess
ighted in dark grey

Field name	Request Data Item
**Patient demographics**	
PATIENTID	
LUCADAID	
**NHS_NUMBER**	
**PERSON FAMILY NAME (Surname)**	
**PERSON NAME GIVEN (Forenames)**	
**POSTCODE**	
POSTCODE	
Sex	
**BIRTH_DATE**	
BIRTH_DATE_YYY	
BIRTH_DATE_MM	
AGE_AT_DIAGNOSIS	
FIVEYEARAGEBAND (Age at diagnosis in 5 year age bands (0-4 etc.))	
ORG_CODE_PROVIDER	
ORG_CODE_PROVIDER (pseudonymised)	
REGISTRY_CODE	
**Referral**<br>ɛection is used to record details about referral to a lung cancer specialist / team.	
SOURCE_OF_REFERRAL	
REFERRAL_DECISION_DATE	
REFERRAL_DECISION_DATE_YYYY	
REFERRAL_DECISION_DATE_MM	
Option to provide number of days from a selected date	
DATE_FIRST_SEEN	
DATE_FIRST_SEEN_YYYY	
DATE_FIRST_SEEN_MM	
Option to provide number of days from a selected date	
SPECIALIST_REFERRAL_DATE	
SPECIALIST_REFERRAL_DATE_YYYY	
SPECIALIST_REFERRAL_DATE_MM	
Option to provide number of days from a selected date	
ORG_FIRST_SEEN	
ORG_FIRST_SEEN (pseudonymised)	
**Diagnosis**<br>ɑlignancy is discovered only at autopsy, or via details from a death certificate, then this section should be comple	
DIAGNOSIS_DATE	
DIAGNOSIS_DATE_YYYY	
DIAGNOSIS_DATE_MM	
Option to provide number of days from a selected date	

ORG_CODE_DIAGNOSIS	
ORG_CODE_DIAGNOSIS (pseudonymised)	
PRIMARY_DIAGNOSIS_ICD	
LATERALITY	
BASIS_OF_DIAGNOSIS	
**Pre-treatment staging**<br>refers to the International Union Against Cancer's coding system (6th edition 1997, 7th edition 2009). This stagi<br>ogical data available. Such evidence arises from physical examination, imaging, endoscopy, biopsy, surgical ex	
TNM_VERSION	
STAGING_CERTAINTY_FACTOR_M_CAT	
STAGING_CERTAINTY_FACTOR_N_CAT	
STAGING_CERTAINTY_FACTOR_T_CAT	
T_CAT_FINAL_PRE_TREAT	
N_CAT_FINAL_PRE_TREAT	
TNM_CAT_FINAL_PRE_TREAT	
TNM_CAT_FINAL_PRE_TREAT	
SITE_SPECIFIC_STAGING	
HISTOLOGY_SNOMED	
MORPHOLOGY_SNOMED	
STAGING_PROCEDURE_PERFORMED	
STG_PROC_MEDIASTIN	
MEDIASTINOSCOPY_MEDIASTINOTOMY	
STG_PROC_FNA	
STG_PROC_OTHER	
STG_PROC_UNKNOWN	
**Cancer care plan/ MDT details**<br>s care. The cancer care plan will relate to this particular diagnosis. It is appreciated that a cancer care plan can<br>as more information about the stage of the disease is ascertained.	
MDT_DISCUSSION_IND	
MDT_DATE	
MDT_DATE_YYYY	
MDT_DATE_MM	
Option to provide number of days from a selected date	
CARE_PLAN_INTENT	
CARE_PLAN_AGREED_DATE	
CARE_PLAN_AGREED_DATE_YYYY	
CARE_PLAN_AGREED_DATE_MM	
Option to provide number of days from a selected date	
CANCER_TREATMENT_INTENT	
DRUG_TREATMENT_INTENT	
FEV1_ABSOLUTE_AMOUNT	
FEV1_PERCENTAGE	
PERFORMANCE_STATUS	
**Significant Co-morbidity / Treatment of Choice**<br>tient did not receive the treatment of choice the reason should be recorded here	
SIGNIFICANT_COMORBIDITY	
**Co-morbidity**<br>son why the patient did not receive the treatment of choice, the nature of the co-morbidity should be recorded	
DEMENTIA_CEREBROVASC_DISEASE	
CARDIOVASCULAR_DISEASE	
RENAL_FAILURE	
OTHER_MALIGNANCY	
SEVERE_WEIGHT_LOSS	
OTHER_SIGNIFICANT_CO_MORBIDITY	
DATE_COMORBIDITY_RECORDED	
DATE_COMORBIDITY_RECORDED_YYYY	
DATE_COMORBIDITY_RECORDED_MM	
Option to provide number of days from a selected date	

For peer review only

**Key Investigations**	
ıg cancer. Record all procedures used even if the result was negative. The purpose of this section is to establish in what order and whether there are delays in accessing certain services	
CT_SCAN	
CT_SCAN_DATE	
CT_SCAN_DATE_YYYY	
CT_SCAN_DATE_MM	
Option to provide number of days from a selected date	
PET_OR_PET_CT_SCAN	
PET_OR_PET_CT_SCAN_DATE	
PET_OR_PET_CT_SCAN_DATE_YYYY	
PET_OR_PET_CT_SCAN_DATE_MM	
Option to provide number of days from a selected date	
BRONCHOSCOPY	
BRONCHOSCOPY_DATE	
BRONCHOSCOPY_DATE_YYYY	
BRONCHOSCOPY_DATE_MM	
Option to provide number of days from a selected date	
CT_GUIDED_BIOPSY	
CT_GUIDED_BIOPSY_DATE	
CT_GUIDED_BIOPSY_DATE_YYYY	
CT_GUIDED_BIOPSY_DATE_MM	
Option to provide number of days from a selected date	
DIAGNOSTIC_BIOPSY	
DIAGNOSTIC_BIOPSY_DATE	
DIAGNOSTIC_BIOPSY_DATE_YYYY	
DIAGNOSTIC_BIOPSY_DATE_MM	
Option to provide number of days from a selected date	
**Nursing care**	
ancer patient journey and refers to any specialist nursing input that occurs between the patient being referred to eir primary treatment. It is not intended to record follow up information after treatment.	
ASSESSED_NURSE_SPECIALIST	
DATE_ASSESSED_NURSE_SPECIALIST	
DATE_ASSESSED_NURSE_SPECIALIST_YYYY	
DATE_ASSESSED_NURSE_SPECIALIST_MM	
Option to provide number of days from a selected date	
HOW_ASSESSED_NURSE_SPECIALIST	
STAGE_PATH_ASSESSED_NURSE_SPEC	
NURSE_SPEC_PRESENT_AT_DIAG	
**Treatment - Clinical trial**	
; section to record whether or not the patient is eligible for entry into a clinical trial	
PATIENT_TRIAL_STATUS	
TREATMENT_PLAN_TYPE	
SUGGESTED_CANCER_TRT_PLAN	
**Surgery**	
ocedure under taken with the intention of curing the patient. For Mesothelioma Patients, it is accepted that all su palliative	
ORG_CODE_SURGERY	
ORG_CODE_SURGERY (pseudonymised)	
DTTD_SURGERY	
DTTD_SURGERY_YYYY	
DTTD_SURGERY_MM	
Option to provide number of days from a selected date	
PROCEDURE_DATE_SURGERY	
PROCEDURE_DATE_SURGERY_YYYY	
PROCEDURE_DATE_SURGERY_MM	
Option to provide number of days from a selected date	
PRIMARY_PROCEDURE_OPCS	

**Post surgery pathology** nd the post operative staging based on the TNM system. Note that post-operative histology data is recorded in t treatment window.	
EXCISION_MARGIN	
CERTAINTY_FACTOR_PATH_STAGE_T	
CERTAINTY_FACTOR_PATH_STAGE_N	
CERTAINTY_FACTOR_PATH_STAGE_M	
T_CAT_PATH	
N_CAT_PATH	
M_CAT_PATH	
TNM_CAT_PATH	
**Histology (Post Surgery)**	
INVESTIGATION_RESULT_DATE	
INVESTIGATION_RESULT_DATE_YYYY	
INVESTIGATION_RESULT_DATE_MM	
Option to provide number of days from a selected date	
HISTOLOGY_SNOMED_2	
POST_TRT_SITE_SPECIFIC_CLASS	
**Chemotherapy**	
ORG_CODE_ANTI_CANCER_TREAT	
ORG_CODE_ANTI_CANCER_TREAT (pseudonymised)	
DTTD_ANTI_CANCER_REGIMEN	
DTTD_ANTI_CANCER_REGIMEN_YYYY	
DTTD_ANTI_CANCER_REGIMEN_MM	
Option to provide number of days from a selected date	
START_DATE_ANTI_CANCER_REGIMEN	
START_DATE_ANTI_CANCER_REGIMEN_YYYY	
START_DATE_ANTI_CANCER_REGIMEN_MM	
Option to provide number of days from a selected date	
CT_TREATMENT_GIVEN	
Teletherapy (external beam radiotherapy). This section is used to record the initial teletherapy treatment.	
ORG_CODE_TELETHERAPY	
ORG_CODE_TELETHERAPY (pseudonymised)	
DTTD_TELETHERAPY	
DTTD_TELETHERAPY_YYYY	
DTTD_TELETHERAPY_MM	
Option to provide number of days from a selected date	
RT_TREATMENT_GIVEN	
RT_ANATOMICAL_SITE	
START_DATE_TELETHERAPY	
START_DATE_TELETHERAPY_YYYY	
START_DATE_TELETHERAPY_MM	
Option to provide number of days from a selected date	
**Brachytherapy**	
ORG_CODE_BRACHYTHERAPY	
ORG_CODE_BRACHYTHERAPY (pseudonymised)	
DTTD_BRACHYTHERAPY	
DTTD_BRACHYTHERAPY_YYYY	
DTTD_BRACHYTHERAPY_MM	
Option to provide number of days from a selected date	
START_DATE_BRACHYTHERAPY	
START_DATE_BRACHYTHERAPY_YYYY	
START_DATE_BRACHYTHERAPY_MM	
Option to provide number of days from a selected date	
**Palliative care**	
ORG_CODE_PALLIATIVE	
ORG_CODE_PALLIATIVE (pseudonymised)	
PALL_CARE_PROV_TYPE	
PALL_CARE_COMM_PROV_TYPE	
DTTD_PALLIATIVE	
DTTD_PALLIATIVE_YYYY	

DTTD_PALLIATIVE_MM	
Option to provide number of days from a selected date	
START_DATE_PALLIATIVE	
START_DATE_PALLIATIVE_YYYY	
START_DATE_PALLIATIVE_MM	
Option to provide number of days from a selected date	
PALL_CARE_INT_TRT_GIVEN	
PALL_CARE_INT_TRT_GIVEN_DATE	
PALL_CARE_INT_TRT_GIVEN_DATE_YYYY	
PALL_CARE_INT_TRT_GIVEN_DATE_MM	
Option to provide number of days from a selected date	
**Active monitoring**	
ORG_CODE_ACTIVE_MONITORING	
ORG_CODE_ACTIVE_MONITORING (pseudonymised)	
DTTD_ACTIVE_MONITORING	
DTTD_ACTIVE_MONITORING_YYYY	
DTTD_ACTIVE_MONITORING_MM	
Option to provide number of days from a selected date	
**Outcome - death**	
DEATH_DATE	
DEATH_DATE_YYYY	
DEATH_DATE_MM	
Option to provide number of days from a selected date	
DEATH_RELATED_TO_TREATMENT	
TREATMENT_MORBIDITY_TYPE	
**Outcome - Prophylactic cranial irradiation**	
RCVD_PROPHY_CRANIAL_IRRAD	
ORIG_TRT_PLAN_CARRIED_OUT	
ORIG_TRT_PLAN_FAILURE_REASON	

For peer review only

ems in the LUCADA dataset have been
n to lung cancer patients from diagnosis to
other national guidance (e.g. NICE guidance)

egulation covering your organisation.
. Privacy by design and default principles

Justification
eted with as much information as is available.

For peer review only

ing is usually agreed at the MDT meeting or<br>xploration and other relevant examinations.
change as the patient's disease progresses or

For peer review only

h what diagnostic techniques are being used,
the hospital to the point at which the patient
urgical procedures in this group of patients are

peer review only

the histology window and not in the surgical

For peer review only

For peer review only

Introduction

The Quality of Lif
patients in Janua
cancer to treatme
Assessment of C

Coverage: Routi

Data item select

Please select you

Key:

Items in red

Item number
1
2
3
4
5
6
7
8
9
10
11
12
13
14
15
16
17
18
19
20
21
22
23
24
25
26
27
28
29
30
31
32
33
34
35
36
37
38
39

peer review only

40
41
42
43
44
45
46
47
48
49
50
51
52
53
54
55
56
57
58
59
60
61
62
63
64
65
66
67
68
69
70
71
72
73
74
75
76
77
78
79
80
81
82
83
84
85
86

peer review only

87
88
89
90
91
92
93
94
95
96
97
98
99
100
101
102
103
104
105
106
107
108
109
110
111
112
113
114
115
116
117
118
119
120
121

For peer review only

fe of Colorectal Cancer Survivors in England: Patient Reported Outcome Measures includes responses from respond
ary 2013, who were alive 12-36 months after a diagnosis of colorectal cancer respondents (n=34,467) described differ
ent and through to their experiences of aftercare. The outcome questions in the survey are made up of three instrume
Cancer Therapy) and SDI (Social Difficulties Inventory). https://www.england.nhs.uk/wp-content/uploads/2015/03/color

inely linked to incident cancer cases from: 01/01/2010 - 31/12/2011, linked at a patient level

tion

ur required data items from the list below. If you require more details on the meaning and definition of specific data ite

Identifiable fields that require additional permissions to access (as outlined above)
Sensitive items that require additional justification in your protocol to access
Privacy by design and default' alternatives to the sensitive items highlighted in dark grey

Data Item	Field Name
**Your health today**	
Pseudonymised patient ID	PATIENTID
Q1. What treatments have you received for your bowel (colorectal) cancer?	q1_1
Q1. What treatments have you received for your bowel (colorectal) cancer?	q1_2
Q1. What treatments have you received for your bowel (colorectal) cancer?	q1_3
Q2. How long is it since you completed your initial treatment for bowel (colorectal) cancer?	q2
Q3. How has your bowel (colorectal) cancer responded to treatment?	q3
Q4. If you have a stoma (e.g. colostomy) is it:	q4
Q5. Mobility	q5
Q6. Self care	q6
Q7. Usual activities (work, study, housework, family or leisure activities)	q7
Q8. Pain/discomfort	q8
Q9. Anxiety/depression	q9
Q10. I have swelling or cramps in my stomach area	q10
Q11. I am losing weight	q11
Q12. I have control of my bowels	q12
Q13. I can digest my food well	q13
Q14. I have diarrhoea	q14
Q15. I have a good appetite	q15
Q16. I like the appearance of my body	q16
Q17. I have difficulty urinating	q17
Q18. I urinate more frequently than usual	q18
Q19. I leak urine	q19
Q20. Do you have an ostomy appliance/ stoma?	q20
Q21. I am embarrassed by my ostomy appliance/ stoma	q21
Q22. Caring for my ostomy appliance/ stoma is difficult	q22
Q23. Do you have any difficulty in controlling your bowels (e.g. any accidents)?	q23
Q24. If yes, how often do you have difficulties?	q24
Q25. In the past week, on how many days have you done a total of 30 minutes or more physical activity, which was enough to raise your heart rate?	q25
Q26. Have you had any difficulty in maintaining your independence	q26
Q27. Have you had any difficulty in carrying out your domestic chores?	q27
Q28. Have you had any difficulty with managing your own personal care?	q28
Q29. Have you had any difficulty with looking after those who depend on you?	q29
Q30. Have any of those close to you (e.g. partner, children, parents) had any difficulty with the support available to them?	q30
Q31. Have you had any difficulty with benefits?	q31
Q32. Have you had any financial difficulties?	q32
Q33. Have you had any difficulty with financial services?	q33
Q34. Have you had any difficulty concerning your work?	q34
Q35. Have you had any difficulty with planning for your own or your family's future?	q35
Q36. Have you had any difficulty with communicating with those closest to you?	q36

For peer review only

Q37. Have you had any difficulty with communicating with others?	q37
Q38. Have you had any difficulty concerning sexual matters?	q38
Q39. Have you had any difficulty concerning plans to have a family?	q39
Q40. Have you had any difficulty concerning your appearance or body image?	q40
Q41. Have you felt isolated?	q41
Q42. Have you had any difficulty with getting around?	q42
Q43. Have you had any difficulty with where you live?	q43
Q44. Have you had any difficulty in carrying out your recreational activities?	q44
Q45. Have you had any difficulty with your plans to travel or take a holiday?	q45
Q46. Have you had any difficulty with any other area of your everyday life?	q46
Q47. I have fears about my cancer spreading	q47
Q48. I have fears about my cancer coming back	q48
Q49. I have fears about death and dying	q49
Q50. I experience memory loss	q50
Q51. I have trouble sleeping	q51
Q52. I have trouble concentrating	q52
Q53. I always feel tired	q53
Q54. I experience mood swings	q54
Q55. I am often irritable	q55
Q56. Do you have an up to date written care plan?	q56
Q57. Do you have a named nurse who you can contact if you have a worry about your cancer care?	q57
Q58. Do you know who to contact if you have a concern about any aspect of living with or after cancer?	q58
Q59. Do you think that hospital staff did everything they could to support you following your cancer treatment?	q59
Q60. Do you think that GPs and nurses at your general practice do everything they can to support you following your cancer treatment?	q60
Q61. Following your initial cancer treatment have you been given enough care and help from health and social services (for example, district nurses, home helps or occupational therapists)?	q61
Q62. Do you consider yourself to be a	q62
Q63. If an ex-smoker, how long ago did you stop?	q63
Q64. Did you receive any advice or information on any of the following issues?	q64_1
Q64. Did you receive any advice or information on any of the following issues?	q64_2
Q64. Did you receive any advice or information on any of the following issues?	q64_3
Q64. Did you receive any advice or information on any of the following issues?	q64_4
Q64. Did you receive any advice or information on any of the following issues?	q64_5
Q64. Did you receive any advice or information on any of the following issues?	q64_6
Q64. Did you receive any advice or information on any of the following issues?	q64_7
Q64. Did you receive any advice or information on any of the following issues?	q64_8
Q64. Did you receive any advice or information on any of the following issues?	q64_9
Q64. Did you receive any advice or information on any of the following issues?	q64_10
Q64. Did you receive any advice or information on any of the following issues?	q64_11
Q65. Would it have been helpful to have had more advice or information on any of the following issues?	q65_1
Q65. Would it have been helpful to have had more advice or information on any of the following issues?	q65_2
Q65. Would it have been helpful to have had more advice or information on any of the following issues?	q65_3
Q65. Would it have been helpful to have had more advice or information on any of the following issues?	q65_4
Q65. Would it have been helpful to have had more advice or information on any of the following issues?	q65_5
Q65. Would it have been helpful to have had more advice or information on any of the following issues?	q65_6
Q65. Would it have been helpful to have had more advice or information on any of the following issues?	q65_7
Q65. Would it have been helpful to have had more advice or information on any of the following issues?	q65_8
Q65. Would it have been helpful to have had more advice or information on any of the following issues?	q65_9

For peer review only

Q65. Would it have been helpful to have had more advice or information on any of the following issues?	q65_10
Q66. What year were you born?	q66
Q67. Are you male or female?	q67
Q68. Do you look after, or give any help or support (not as part of your paid employment) to family members, friends, neighbours or others because of either: Long term physical or mental health/ disability, or Problems relating to old age	q68
Q69. Which of the following best describes your sexual orientation?	q69
Q70. Which statement best describes your living arrangements?	q70_1
Q70. Which statement best describes your living arrangements?	q70_2
Q70. Which statement best describes your living arrangements?	q70_3
Q70. Which statement best describes your living arrangements?	q70_4
Q71. Do you have a long standing health condition?	q71_1
Q71. Do you have a long standing health condition?	q71_2
Q71. Do you have a long standing health condition?	q71_3
Q72. Which, if any, of the following conditions do you have?	q72_1
Q72. Which, if any, of the following conditions do you have?	q72_2
Q72. Which, if any, of the following conditions do you have?	q72_3
Q72. Which, if any, of the following conditions do you have?	q72_4
Q72. Which, if any, of the following conditions do you have?	q72_5
Q72. Which, if any, of the following conditions do you have?	q72_6
Q72. Which, if any, of the following conditions do you have?	q72_7
Q72. Which, if any, of the following conditions do you have?	q72_8
Q72. Which, if any, of the following conditions do you have?	q72_9
Q72. Which, if any, of the following conditions do you have?	q72_10
Q72. Which, if any, of the following conditions do you have?	q72_11
Q72. Which, if any, of the following conditions do you have?	q72_12
Q72. Which, if any, of the following conditions do you have?	q72_13
Q72. Which, if any, of the following conditions do you have?	q72_14
Q72. Which, if any, of the following conditions do you have?	q72_15
Q72. Which, if any, of the following conditions do you have?	q72_16
Q72. Which, if any, of the following conditions do you have?	q72_17
Q72. Which, if any, of the following conditions do you have?	q72_18
Q73. What was your employment status before you were diagnosed with cancer?	q73
Q74. What is your employment status currently?	q74
Q75. If you are currently employed at the moment, are you:	q75
Q76. To which of these ethnic groups would you say you belong?	**q76**
Broad ethnic group	Option to group e

For peer review only

lents to a questionnaire distributed to colorectal cancer
rent parts of their journey from a diagnosis of colorectal
ents: the EQ-5D (Euroqol 5 level), FACT items (Functional
rectal-cancer-proms-report-140314.pdf

ems please visit: cancerproms.ncr.nhs.uk

Request Data Item	Justification

For peer re

For peer review only

For peer review only

HOSPITAL EPISODE STATISTICS DATA

Hospital Episode Statistics Admitted Patient Care

Period

1997/98
1998/99
1999/00
2000/01
2001/02
2002/03
2003/04
2004/05
2005/06
2006/07
2007/08
2008/09
2009/10
2010/11
2011/12
2012/13
2013/14
2014/15
2015/16
2016/17
2017/18
2018/19

Other fields

[ACTIVAGE Age at activity date,
[ADMIAGE Age on admission,
[ADMIDATE Date of admission,
[ADMIFLAG Admission episode flag,
[ADMIMETH Method of admission,
[ADMISORC Source of admission,
[ADMISTAT Psychiatric history on admission,
[CLASSPAT Patient classification,
[DIAG_3_NN Diagnosis - 3 characters,
[Diag_4_01 Primary Diagnosis - 4 characters,
[DIAG_4_NN Diagnosis - 4 characters,
[DIAG_NN All Diagnosis codes,
[DISDATE Date of discharge,
[DISDEST Destination on discharge,
[DISFLAG Discharge episode flag,
[DISMETH Method of discharge,
[DISREADYDATE Discharge ready date,
[DOMPROC Trust derived dominant procedure,
[ENCRYPTED_HESID Encrypted HESID,

For peer review only

[EPIEND Date episode ended,
[EPIORDER Episode order,
[EPISTART Date episode started,
[EPISTAT Episode status,
[EPITYPE Episode type,
[ETHNOS Ethnic category,
[FAE Finished Admission Episode,
[FCEFLAG Finished consultant episode flag,
[HRGNHSVN Version No. of Trust derived HRG,
[IMD04 IMD Index of Multiple Deprivation,
[IMD04_DECILE IMD Decile Group,
[IMD04C IMD Crime Domain,
[IMD04ED IMD Education Training and Skills Domain,
[IMD04EM IMD Employment Deprivation Domain,
[IMD04HD IMD Health and Disability Domain,
[IMD04HS IMD Barriers to Housing and Service Domain,
[IMD04I IMD Income Domain,
[IMD04IA IMD Income affecting Adults Domain,
[IMD04IC IMD Income affecting Children Domain,
[IMD04LE IMD Living Environment Domain,
[IMD04RK IMD Overall Rank,
[LSOA01 Lower Super Output Area (LSOA01),
[LSOA11 Lower Super Output Area (LSOA11),
[MAINSPEF Main specialty,
[OPDATE_NN Date of operation,
[OPERSTAT Operation status code,
[OPERTN_4_01 Primary Operative procedure codes 4 character,
[OPERTN_4_NN All secondary Operative procedure codes 4 character,
[PCON_ONS Westminster parliamentary constituency (ONS),
[PROCODE3 Provider code - 3 character,
[PROCODE5 Provider code - 5 character,
[PROCODET Provider code,
[PROVSPNOPS Pseudonymised hospital provider spell number,
[RESGOR Government office region of residence,
[SEX Sex of patient,
[SITETRET Site code of treatment,
[STARTAGE Age at start of episode,
[SUSHRG SUS generated HRG,
[SUSHRGVERS SUS generated HRG version number,
[TRETSPEF Treatment specialty

Hospital Episode Statistics Outpatients

Periods

2003/04
2004/05
2005/06
2006/07

For peer review only

2007/08
2008/09
2009/10
2010/11
2011/12
2012/13
2013/14
2014/15
2015/16
2016/17
2017/18
2018/19

Other fields
[APPTDATE Appointment date,
[ATTENDED Attended or did not attend,
[ATTENDID Attendance identifier,
[CHAPTER Primary diagnosis chapter,
[DIAG_3_01 Primary diagnosis - 3 character,
[DIAG_3_NN Secondary diagnoses - 3 character,
[DIAG_4_01 Primary diagnosis - 4 character,
[DIAG_4_CONCAT 4 character concatenated diagnosis,
[DIAG_4_NN Secondary diagnosess - 4 character,
[DIAG_COUNT Count of diagnoses,
[DIAG_NN Diagnosis,
[DNADATE Last DNA or patient cancelled date,
[ENCRYPTED_HESID Encrypted HESID,
[ETHNOS Ethnic category,
[HRGNHS Trust derived HRG value,
[HRGNHSVN Version No. of Trust derived HRG,
[IMD04 IMD Index of Multiple Deprivation,
[MAINSPEF Main specialty,
[MYDOB Date of Birth - month and year,
[NODIAGS Number of Diagnosis,
[OPERTN_4_NN All Operative procedure codes,
[OPERTN_NN Operative procedure,
[PROCODE3 Provider code (3 character),
[PROCODE5 Provider code (5 character),
[SEX Sex of patient,
[STAFFTYP Medical staff type seeing patient,
[SUSHRG SUS generated HRG,
[SUSHRGVERS SUS generated HRG version number,
[TRETSPEF Treatment specialty,
[WAITING Days waiting

Civil Registration (Deaths) - Secondary Care Cut
Periods

For peer review only

Future 31/12/2016

Sensitive fields

[cause_of_death] Original Underlying Cause of Death,

[cause_of_death_non_neonatal] Non-neonatal cause of death,

[death_record_used] Death Record Used,

[dod] Date of Death,

[subsequent_activity] Subsequent activity

For peer review only
